# CRM1-dependent nuclear export of TRIM28 promotes MAVS K48-linked ubiquitination and suppresses RIG-I-mediated antiviral response

**DOI:** 10.3389/fimmu.2026.1744833

**Published:** 2026-03-24

**Authors:** Ruihua Xin, Wenshu Zou, Zhengying Qiu, Lina Huang, Xiaohan Jing, Qilin Huang, Zixiang Zhu, Jianxi Li, Mutien-Marie Garigliany

**Affiliations:** 1 Key Laboratory of Veterinary Pharmaceutical Development of Ministry of Agriculture, Technology Innovation Center of Traditional Chinese Veterinary Medicine of Gansu Province, Lanzhou Institute of Husbandry and Pharmaceutical Sciences, Chinese Academy of Agricultural Sciences, Lanzhou, China; 2 Fundamental and Applied Research for Animals & Health (FARAH), Institute of Epidemics (INDEEP), Laboratory of Pathology, Faculty of Veterinary Medicine, University of Liège, Liège, Belgium; 3 Institute of Traditional Chinese Medicine Health Industry, China Academy of Chinese Medical Sciences, Nanchang, China; 4 State Key Laboratory for Animal Disease Control and Prevention, College of Veterinary Medicine, Lanzhou University, Lanzhou Veterinary Research Institute, Chinese Academy of Agricultural Sciences, Lanzhou, China

**Keywords:** TRIM28, MAVS, ubiquitination, RIG-I signaling, nucleocytoplasmic trafficking, RNA virus replication

## Abstract

**Background:**

Ubiquitination is a pivotal post-translational mechanism regulating antiviral innate immunity. TRIM28, a member of the transcription intermediary factor 1 (TIF1) subfamily of the TRIM protein family, has been implicated in the modulation of retinoic acid-inducible gene I (RIG-I) signaling. However, the molecular basis and *in vivo* relevance remain unclear.

**Methods:**

TRIM28 overexpression and knockdown systems were used to evaluate type I interferon (IFN) responses and RNA virus replication *in vitro*, and an AAV-mediated lung-specific TRIM28 knockdown model was used for *in vivo* validation. Virus-induced nucleocytoplasmic trafficking of TRIM28 was analyzed by confocal microscopy. Protein interactions and ubiquitination events were examined by co-immunoprecipitation and in-cell ubiquitination assays. TRIM28 domain deletion constructs and MAVS lysine mutants were used to assess domain requirements and to probe functionally relevant ubiquitination sites on MAVS.

**Results:**

TRIM28 knockdown enhanced type I IFN responses and inhibited RNA virus replication *in vitro* and *in vivo*. Viral infection predominantly triggered CRM1-dependent nuclear export of TRIM28, leading to its cytoplasmic accumulation. Cytoplasmic TRIM28 associated with MAVS and promoted K48-linked polyubiquitination in an RBCC domain-dependent manner, reducing TBK1 and IRF3 activation and attenuating IFN production. Mutation of MAVS Lys136 or Lys461 impaired TRIM28-mediated ubiquitination and restored downstream signaling.

**Conclusion:**

TRIM28 functions as a negative regulator of RIG-I-mediated antiviral signaling by promoting K48-linked ubiquitination of MAVS. These findings define the TRIM28-MAVS axis as a regulatory checkpoint in innate antiviral immunity and suggest its potential as a target for antiviral intervention.

## Introduction

RNA viruses are characterized by high mutation rates and broad host adaptability, posing a persistent threat to human health and livestock production (1). The World Health Organization (WHO) estimates that seasonal influenza causes approximately 290,000 to 650,000 respiratory-related deaths annually (2, 3), while the COVID-19 pandemic has resulted in more than seven million deaths and substantial global economic losses (4–6). Their rapid genetic variability enables antiviral escape and reduces vaccine efficacy, highlighting the need for antiviral strategies that target host regulatory pathways rather than viral components alone (7, 8).

TRIM28, also known as KRAB-associated protein 1 (KAP1), is a member of the transcription intermediary factor 1 (TIF1) family (9, 10). It was initially identified as a chromatin regulator involved in maintaining genome stability through heterochromatin formation and histone modifications (9, 11). Its N-terminal RBCC motif (RING-B-box-coiled-coil) confers E3 ligase activity for both SUMO and ubiquitin, allowing TRIM28 to mediate post-translational modification (PTM) of substrate proteins and regulate signal transduction (12, 13). In our recent summary of the roles of TRIM28 in viral infection and innate immunity (14), we noted that TRIM28 not only regulates the transcription of retroviruses through its classical heterochromatin-mediated silencing function (15, 16), including human immunodeficiency virus (HIV) (17–19) and murine leukemia virus (MLV) (20, 21), but also directly modulates the replication of RNA viruses through interactions with viral proteins. For example, TRIM28 enhances SARS-CoV-2 particle assembly and immune evasion by promoting SUMOylation of the nucleocapsid protein (22), supports porcine epidemic diarrhea virus (PEDV) replication in a RING domain-dependent manner (23), and facilitates porcine reproductive and respiratory syndrome virus (PRRSV) replication by preventing ubiquitination of the GP4 glycoprotein (24). These findings indicate that TRIM28 exerts multiple regulatory roles during RNA virus replication. Moreover, TRIM28 has been shown to modulate host innate immune signaling pathways that influence antiviral outcomes (25), suggesting that its regulatory functions extend beyond viral protein targets to host defense mechanisms.

RIG-I is a cytosolic pattern recognition receptor (PRR) that detects viral RNA and activates an innate antiviral signaling pathway essential for restricting RNA virus infection (26, 27). Upon binding short double-stranded RNA or 5′-triphosphate/5′-diphosphate single-stranded RNA, RIG-I undergoes conformational activation (28) and transmits signals through the adaptor mitochondrial antiviral signaling protein (MAVS), leading to TANK-binding kinase 1 (TBK1) activation (28), phosphorylation of interferon regulatory factor 3 and 7 (IRF3/7), and induction of type I IFNs that are essential for limiting viral replication (29). Ubiquitination provides an additional layer of regulation in this pathway, with K63-linked chains promoting complex assembly and signaling (30, 31), whereas K48-linked chains target proteins for proteasomal degradation, thereby fine-tuning the antiviral response. Although TRIM28 has been implicated in modulating components of the RIG-I pathway (32–36), the specific signaling node at which TRIM28 exerts its regulatory function, and whether this regulation is functionally relevant during RNA virus infection *in vivo*, remain unclear.

Here, we demonstrate that TRIM28 suppresses type I IFN responses and promotes the replication of distinct RNA viruses, including vesicular stomatitis virus (VSV, *Rhabdoviridae*) and enterovirus 71 (EV-71, *Picornaviridae*). A lung-specific TRIM28 knockdown mouse model exhibited reduced VSV viral burden and alleviated disease severity, providing *in vivo* evidence that TRIM28 enhances RNA virus virulence. Mechanistically, viral infection induces CRM1-dependent nuclear export of TRIM28, enabling its interaction with MAVS in the cytoplasm. TRIM28 catalyzes K48-linked ubiquitination of MAVS at Lys136 and Lys461, reducing MAVS stability and impairing TBK1-IRF3 signaling, which ultimately attenuates type I IFN production. Together, our findings establish TRIM28 as a negative regulator of RIG-I-mediated antiviral immunity by promoting K48-linked ubiquitination and degradation of MAVS. Importantly, we demonstrate the functional relevance of this regulatory axis during RNA virus infection *in vivo*, providing mechanistic evidence that TRIM28 modulates host antiviral responses through MAVS. These results highlight the TRIM28-MAVS axis as a potential target for host-directed antiviral intervention.

## Materials and methods

### Cells and virus

Human embryonic kidney 293T (HEK293T) cells were kindly provided by Dr. Wei Zhang (Lanzhou Veterinary Research Institute, Chinese Academy of Agricultural Sciences, CAAS) and maintained in Dulbecco’s modified Eagle’s medium (DMEM; Gibco) supplemented with 10% fetal bovine serum (FBS; Gibco, USA), 100 U/mL penicillin, and 100 μg/mL streptomycin (Gibco). THP-1 human monocytic cells (ATCC TIB-202) were maintained in RPMI-1640 medium (Gibco) supplemented with 10% FBS, 100 U/mL penicillin, and 100 μg/mL streptomycin at 37°C with 5% CO_2_. SeV, VSV-GFP, and EV-71 were also kindly provided by Dr. Wei Zhang (Lanzhou Veterinary Research Institute, CAAS).

### Gene knockdown in HEK293T cells

Cells were transfected with a small interfering RNA (siRNA) targeting human TRIM28 (sense: 5′-AGGCAGAGAAAGCAGCCATT-3′; antisense: 5′-TGGCCTCTGTTCTGTGCCTTT-3′) using Lipofectamine RNAiMAX according to the manufacturer’s instructions. A commercially available non-targeting siRNA was used as a negative control.

### CRISPR-Cas9-mediated genome editing

TRIM28-knockout (KO) HEK293T cells were generated using CRISPR/Cas9-mediated gene editing. Single-guide RNAs (sgRNAs) targeting the human TRIM28 gene were cloned into a lentiviral vector (pLVX-U6-CMV-Cas9-P2A-Puro), and lentivirus packaging and transduction were carried out in HEK293T cells. After puromycin selection (5 μg/mL for 4 days), single-cell clones were isolated and screened by Western blot to confirm TRIM28 deletion. The KO cells were cultured under the same conditions as wild-type HEK293T cells. All gene editing procedures and lentiviral production were performed by Xibaihongcheng Biotechnology Co., Ltd. (Beijing, China).

### Animal experiments

Male C57BL/6 mice (8 weeks old, SPF grade) were housed in a specific pathogen-free (SPF) facility under controlled conditions (22 ± 2 °C, 40-60% relative humidity, 12-hour light/dark cycle) with ad libitum access to food and water. To achieve lung-specific knockdown of TRIM28, mice were administered recombinant adeno-associated virus serotype 6 carrying TRIM28-targeting shRNA (rAAV6-ZsGreen-shTRIM28) via intratracheal instillation. The effective shRNA (shRNA2) was cloned into the pAAV-U6-shRNA-CMV-ZsGreen vector. The AAV vector construction, packaging, and purification were performed by Xibeihongcheng Biotechnology Co., Ltd. (Beijing, China). Knockdown efficiency was confirmed by Western blot analysis. Experimental groups included WT and TRIM28 knockdown (KD) mice, each treated with either PBS or vesicular stomatitis virus (VSV) to assess infection-induced responses. Two weeks after AAV administration, mice were intraperitoneally injected with VSV (2×10^7^ PFU/mouse) once daily for three consecutive days. Clinical observations—including general appearance, activity level, food intake, body weight, and survival were recorded daily. At designated time points, lung and serum samples were collected for downstream molecular and histological analyses. All animal experiments were approved by the Animal Ethics Committee of the Lanzhou Institute of Husbandry and Pharmaceutical Sciences, Chinese Academy of Agricultural Sciences (Approval No. 2024-059), and conducted in accordance with institutional and local regulations.

### Antibodies

Primary antibodies against TRIM28 (KAP1, #15202-1-AP), GAPDH (#60004-1-Ig), β-actin (#66009-1-Ig), CRM1 (#66763-1-Ig), HSP60 (#15282-1-AP), Lamin B (#12987-1-AP), His-tag (#66005-1-Ig) were purchased from Proteintech (Wuhan, China). Ubiquitin (#3933S), K48-linked ubiquitin (#12805S), MAVS (#24930S), TBK1 (#38066S), phospho-TBK1 (#5483S), IRF3 (#4302S), phospho-IRF3 (#4947S), HA (#3724S), and c-Myc (#2276S) were obtained from Cell Signaling Technology (CST, USA). The anti-GFP antibody (#334077) was purchased from Abmart (Shanghai, China), and the anti-Flag antibody (A8592) from Sigma-Aldrich (USA). Mouse IgG isotype control (#A7028) was obtained from Beyotime Biotechnology (China). Fluorescent secondary antibodies, including goat anti-rabbit IgG (H+L) Alexa Fluor™ 488 (#A-11008) and goat anti-mouse IgG (H+L) Cyanine3 (#A10521), were purchased from Invitrogen (Thermo Fisher Scientific, USA). Unless otherwise specified, all primary antibodies were used at 1:1000 dilution for Western blotting (WB) and 1:100 for Immunofluorescence (IF) staining. For phosphorylation analyses, phospho-specific antibodies against TBK1 and IRF3 (p-TBK1 and p-IRF3) were used for immunoblotting.

### Reagents

Polyinosinic-polycytidylic acid (poly I:C) was obtained from InvivoGen. Selinexor (KPT-330, HY-17536) was purchased from MedChemExpress (MCE, China). Proteasome inhibitor MG132 (HY-13259), protein A/G magnetic beads (41105507), and DAPI dihydrochloride (268298) were purchased from Merck. The reverse transcription kit (PrimeScript RT reagent kit, RR037A) and qPCR master mix (TB Green Premix Ex Taq II) were from Takara (Japan). The HE staining kit (C0105M) and anti-fade mounting medium (E675011) were from Beyotime Biotechnology (China). Lipofectamine RNAiMAX (13778075), Lipofectamine 2000 (L3000015), TRIzol reagent (15596026), Pierce™ ECL Western Blotting Substrate (32106), and culture media DMEM (high glucose) and RPMI-1640 were all obtained from Thermo Fisher Scientific (USA). Human (Cat# 414101) and mouse (Cat# 424001) IFN-β ELISA kits were also from Thermo Fisher Scientific/Invitrogen. The Dual-Luciferase^®^ Reporter Assay System (E1960) was from Promega (USA). Fetal bovine serum (FBS, 10270), penicillin-streptomycin (15140-122), and GlutaMAX Supplement (35050-061) were obtained from Gibco (Thermo Fisher Scientific, USA). EndoFree Plasmid Maxi Kit and RNase A were purchased from Sangon Biotech (Shanghai, China). Non-fat dry milk (Cat# D8340) used for membrane blocking was obtained from Solarbio (Beijing, China). The SDS-PAGE gel preparation kit (125 gels) was from Epizyme (China).

### Plasmids

HA-tagged expression plasmids for RIG-I, MDA5, TBK1, IRF3, IRF7, MAVS, as well as the luciferase reporter plasmids pGL4.32-NF-κB-luc, pGL4.32-IFN-β-luc, and pRL-TK were generously provided by Dr Shasha Li (Lanzhou Veterinary Research Institute, CAAS). Ubiquitin expression constructs, including His-tagged pcDNA3.1(+)-Ubiquitin (K6, K11, K27, K29, K33, K48, K63) and MAVS point mutants (K461R and K136R), along with TRIM28 expression vectors (pcDNA3.1-Myc-TRIM28), were purchased from the Public Protein/Plasmid Library (PPL, Biogot Technology, Nanjing, China). All plasmids were propagated in Escherichia *coli* DH5α competent cells (TransGen Biotech, China) and purified using the EndoFree Plasmid Maxi Kit (Sangon Biotech, China). Plasmid sequences were verified by Sanger sequencing prior to transfection.

### TRIM28 truncation

Lentiviral vectors encoding N-terminal or C-terminal truncation mutants of TRIM28 were constructed and packaged by Xibaihongcheng Biotechnology Co., Ltd. (Beijing, China). HEK293T cells were transduced with lentiviruses according to the supplier’s instructions. Briefly, cells were seeded at an appropriate density and incubated with lentiviral particles in the presence of 8 μg/mL polybrene. After 24 h, the culture medium was replaced, and puromycin (2 μg/mL) was added for the selection of successfully transduced cells. Stable cell populations were maintained under puromycin selection, and expression of TRIM28 truncation constructs was confirmed by Western blotting.

### Co-immunoprecipitation

Cells were lysed in IP lysis buffer (containing protease and phosphatase inhibitors) on ice for 30 minutes and centrifuged to remove debris. The supernatants were incubated with specific primary antibodies overnight at 4 °C with gentle rotation. The following day, Protein A/G magnetic beads (HY-K0202, MedChemExpress) were added to the mixtures and incubated for 2–4 hours at 4 °C. Beads were then washed extensively with lysis buffer to remove nonspecific proteins. The immunoprecipitated complexes were eluted by boiling in SDS loading buffer and analyzed by SDS-PAGE and Western blotting.

### Virus titer determination

Viral titers were determined by plaque assay as previously described (37), with slight modifications. Briefly, HEK293T cells were seeded in 6-well plates and allowed to reach approximately 90% confluency. Serial 10-fold dilutions of recovered VSV samples were prepared in DMEM and added to the cells, followed by incubation at 37 °C for 1 hour with gentle rocking every 15 minutes to facilitate viral adsorption. After infection, the inoculum was removed, and cells were overlaid with 1% low-melting-point agarose dissolved in DMEM supplemented with 2% FBS. Plates were incubated at 37 °C in 5% CO_2_ for 48 hours. After incubation, a second overlay containing 0.1% crystal violet in DMEM was added to stain the monolayer and visualize plaques. Plaques were counted manually, and viral titers were calculated as plaque-forming units per milliliter (PFU/mL).

### Reverse transcription quantitative PCR

Total RNA was extracted using TRIzol reagent according to the manufacturer’s instructions. RNA concentration and purity were assessed with a micro-volume spectrophotometer. cDNA was synthesized using the PrimeScript™ RT Reagent Kit. Quantitative PCR was performed using TB Green^®^ Premix Ex Taq™ II on an Applied Biosystems real-time PCR system. Each 20 μL reaction contained qPCR master mix, gene-specific forward and reverse primers, cDNA template, and nuclease-free water. Gene expression levels were normalized to Actb and calculated using the 2^^–ΔΔCt^ method. Primer sequences are listed in **Table 1**.

**Table 1:** Primer sequences.

Genes	Forward (5′ − 3′)	Reversed (5′ − 3′)
*Human TRIM28*	TTTCATGCGTGATAGTGGCAG	GCCTCTACACAGGTCTCACAC
*Human TRIM62*	CGAGCAGCATCAGGTCACC	CCAGTTGTCGCTTGAGCAG
*Human TRIM49D1*	GGATGTGACCGTCAAAATCCG	CACCCAAGACTAAAGAGTCCCT
*Human TRIM58*	TACCAGGTAAAGCTCCAGATGG	GAAAGCCACGATGCTTCTCAA
*Human TRIM22*	CTGTCCTGTGTGTCAGACCAG	TGTGGGCTCATCTTGACCTCT
*Human TRIM67*	CGTGTCCCGAGCATGAAATG	CCTGAGATAGTTGTGCCTTGTG
*Human TRIM31*	AACCTGTCACCATCGACTGTG	TGATTGCGTTCTTCCTTACGG
*Human TRIM56*	GCCTGCATACCTACTGCCAAG	GCAGCCCATTGACGAAGAAGT
*Human TRIM24*	TGTGAAGGACACTACTGAGGTT	GCTCTGATACACGTCTTGCAG
*Human TRIM68*	GCCTGCATACCTACTGCCAAG	GCAGCCCATTGACGAAGAAGT
*Human TRIM34*	GACGCTGGATAAGTTTGCAGA	CCACCCATACGCACCATTTC
*Human INF-β*	GACATCCCTGAGGAGATTAAG	ATGTTCTGGAGCATCTCATAG
*Human ISG15*	TGGACAAATGCGACGAACC	CCCGCTCACTTGCTGCTT
*Human ISG54*	ACGGTATGCTTGGAACGATTG	AACCCAGAGTGTGGCTGATG
*Human ISG56*	TAGCCAACATGTCCTCACAGAC	TCTTCTACCACTGGTTTCATGC
*Human IL-1β*	GGGATGATGACGACCTGCTA	GCTGTTGCTTGTCTCTCCTC
*Human GAPDH*	CGGGAAGCTTGTGATCAATGG	GGCAGTGATGGCATGGACTG
*MAVS*	CAGGCCGAGCCTATCATCTG	GGGCTTTGAGCTAGTTGGCA
*EV-71*	ACGGTATGCTTGGAACGATTG	AACCCAGAGTGTGGCTGATG
*VSV*	TAGCCAACATGTCCTCACAGAC	TCTTCTACCACTGGTTTCATGC
*Mouse IFN-α*	TCAACTCTCCTCACGGTCCT	TCTGCCCTGATGATCTCCCA
*Mouse IFN-β*	TAGCCAACATGTCCTCACAGAC	TCTTCTACCACTGGTTTCATGC
*Mouse ISG15*	GGGATGATGACGACCTGCTA	GCTGTTGCTTGTCTCTCCTC
*Mouse ISG54*	TGACCCAACCACAAATGC	AGGAACTCCTTAAAGCTGCG
*Mouse ISG56*	AGAGGGAGAGAAGCAACTACA	GGGTCAGTATGTGAGAGGAAGA
*β-actin*	TGCTATGTTGCCCTAGACTTCG	GTTGGCATAGAGGTCTTTACGG

### Immunofluorescence and confocal microscopy

Cells were seeded on sterile coverslips in 24-well plates and cultured overnight. After the designated treatment or transfection, cells were washed with PBS and fixed with 4% cold paraformaldehyde for 10 min. Following three PBS washes, cells were permeabilized with 0.2% Triton X-100 for 5 min. Non-specific binding was blocked by incubating cells with 2% BSA in PBS containing 0.5% Tween-20 for 1 h at room temperature. Cells were then incubated with primary antibodies overnight at 4 °C, followed by fluorophore-conjugated secondary antibodies for 2 h at room temperature in the dark. Nuclei were stained with DAPI (Sangon Biotech, China). Coverslips were mounted with Anti-Fade Mounting Medium (Beyotime) and imaged using a confocal microscope (Leica). Acquired images were processed using Fiji (ImageJ) software.

### RNA-sequencing analysis

Total RNA was extracted from THP-1 cells with or without VSV infection using TRIzol reagent (Invitrogen). Poly(A)+ mRNA was enriched using Oligo(dT) beads and fragmented into ~200–300 bp fragments. Strand-specific cDNA libraries were constructed, PCR-amplified to generate inserts of ~300–400 bp, and sequenced on an Illumina platform to obtain 150 bp paired-end reads. After removing adaptor sequences and low-quality reads, clean reads were aligned to the human reference genome (GRCh38, Ensembl release 104.38) using HISAT2 with default parameters. Gene-level read counts were obtained using HTSeq, and differential expression analysis was performed using DESeq2. Genes with adjusted *P* < 0.05 and |log2(fold change)|≥1.5 were considered differentially expressed. The RNA-seq data generated have been deposited in the NCBI Sequence Read Archive (SRA) under the BioProject accession number PRJNA1356945.

### Co-IP and immunoblotting

Cells were lysed in RIPA buffer supplemented with protease and phosphatase inhibitors. For co-immunoprecipitation, whole-cell lysates were incubated with primary antibodies against the target proteins, followed by the addition of Protein A/G magnetic beads to pull down the immune complexes. Immunoprecipitates or whole-cell lysates were resolved by SDS-PAGE and transferred onto nitrocellulose membranes (Millipore). Membranes were probed with specific primary and HRP-conjugated secondary antibodies. Signal detection was performed using the Immobilon Western Chemiluminescent HRP Substrate (Millipore) and visualized with a Tanon imaging system. In some experiments, mitochondrial proteins were extracted using a mitochondrial isolation kit (C3601, Beyotime), and nuclear/cytoplasmic proteins were separated using a nuclear and cytoplasmic protein extraction kit (P0028, Beyotime), according to the manufacturer’s instructions, for immunoblotting analysis.

### Ubiquitination assay

HEK293T cells were transfected with plasmids encoding HA-tagged MAVS, Myc-tagged TRIM28, and His-tagged ubiquitin (wild-type or mutants). Cells were lysed in RIPA buffer (50 mM Tris-HCl, pH 7.4, 150 mM NaCl, 1% NP-40, 0.5% sodium deoxycholate, 1 mM EDTA) supplemented with protease inhibitors and 20 mM N-ethylmaleimide (Sigma-Aldrich). After adding SDS to a final concentration of 1%, lysates were boiled at 100 °C for 5 min and then diluted to 0.1% SDS. The samples were precleared with Protein A/G magnetic beads and incubated with anti-HA antibody overnight at 4 °C. Immunocomplexes were captured with Protein A/G magnetic beads, washed, eluted by boiling in SDS loading buffer, and analyzed by Western blotting using anti-His and other specific antibodies.

### Statistical analysis

Data are presented as mean ± SD from at least three independent experiments, unless otherwise stated. Statistical analysis was performed using GraphPad Prism 8. Comparisons between groups were assessed using two-tailed unpaired Student’s *t*-test or one-way ANOVA, as appropriate. Statistical significance was defined as *P* < 0.05 (*), *P* < 0.01 (**), *P* < 0.001 (***).

## Results

### TRIM28 suppresses type I IFN and pro-inflammatory cytokine induction by RNA viruses and poly(I:C)

**Figure 1:**
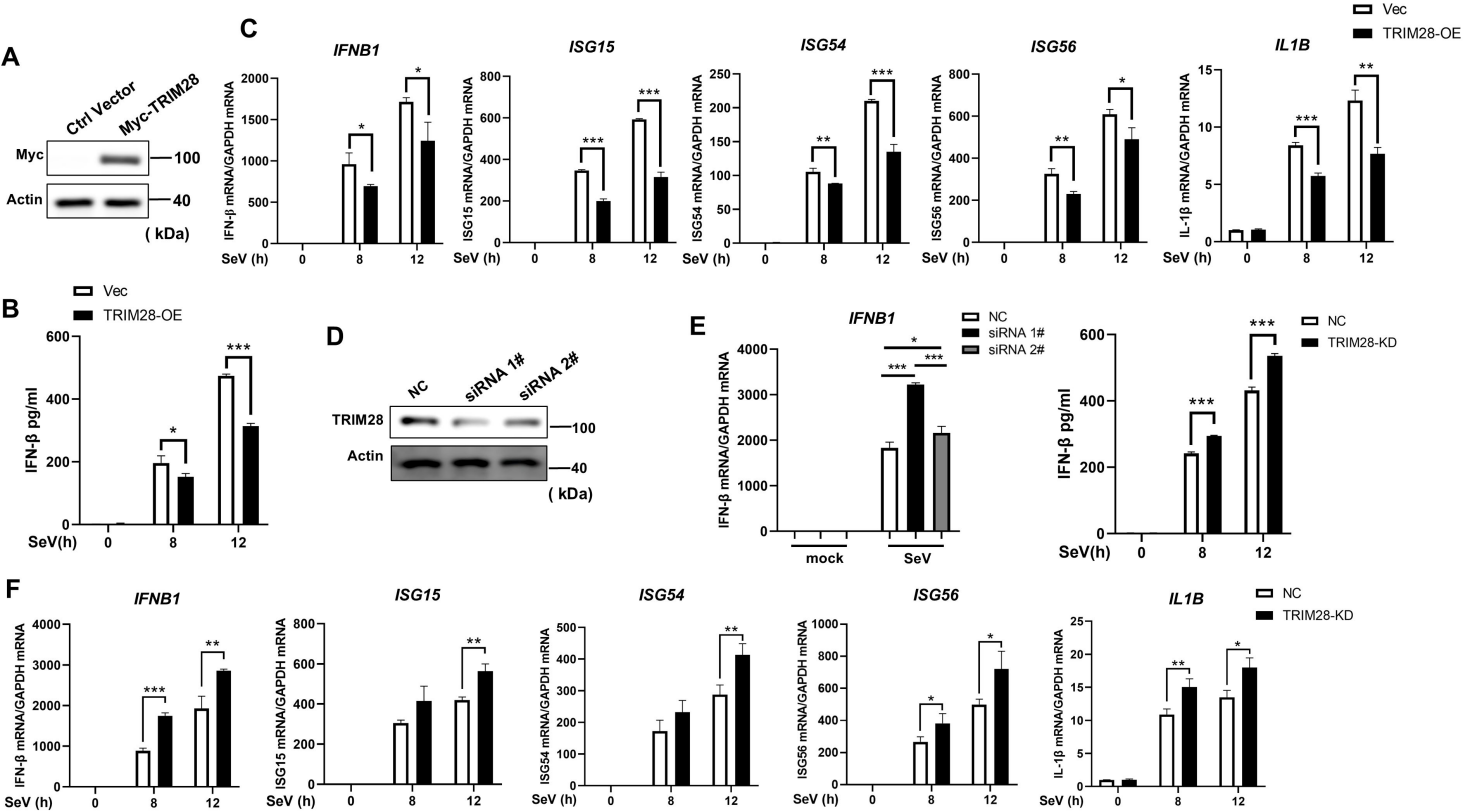
TRIM28 negatively regulates type I IFNs and proinflammatory cytokine responses during RNA virus infection. **(A)** Western blot showing ectopic expression of Myc-tagged TRIM28 in HEK293T cells transfected with empty vector (Vec) or TRIM28-expressing plasmid. **(B)** ELISA analysis of IFN-β secretion in TRIM28-overexpressing (TRIM28-OE) and Vec cells after SeV infection for the indicated times. **(C)** Time-course analysis of *IFNB1*, *ISG15*, *ISG54*, *ISG56*, and *ILB1* mRNA levels in TRIM28-OE and Vec cells following SeV infection, determined by RT-qPCR. **(D)** Western blot showing knockdown efficiency of TRIM28 in HEK293T cells transfected with two independent siRNAs (siRNA 1# and siRNA 2#) or non-targeting control siRNA (NC). **(E**, left**)** RT-qPCR analysis showing that TRIM28 knockdown enhances SeV-induced IFN-β mRNA expression, with siRNA 1# displaying a stronger effect. **(E**, right**)** ELISA quantification of IFN-β secretion in SeV-infected HEK293T cells transfected with siRNA 1# or NC. **(F)** Time-course analysis of *IFNB1*, *ISG15*, *ISG54*, *ISG56*, and *IL1B* mRNA expression in TRIM28-knockdown (TRIM28-KD) and NC cells after SeV infection. Data are presented as mean ± SD. Statistical significance was determined using two-tailed unpaired Student’s t-test; *P < 0.05, **P<0.01, ***P < 0.001.

Transcriptome profiling of SeV-infected THP-1 cells revealed a broad remodeling of the TRIM family, among which TRIM28 displayed a pronounced, time-dependent upregulation at both the mRNA and protein levels in response to either SeV or VSV infection (**Supplementary Figures 1A–G**). This inducible expression pattern suggested a functional role for TRIM28 in antiviral innate immune regulation. To determine this role, we first performed gain-of-function experiments by transfecting HEK293T cells with a Myc-tagged TRIM28 expression plasmid (**Figure 1A**). Ectopic TRIM28 expression significantly reduced SeV-induced IFN-β secretion (**Figure 1B**). Time-course analysis further showed that TRIM28 overexpression decreased the transcript levels of *IFNB1*, *ISG15*, *ISG54*, *ISG56*, and *IL1B* (**Figure 1C**). Similar inhibitory effects were observed upon SeV or poly(I:C) stimulation in a dose-dependent manner (**Supplementary Figures 2A, B**).

In contrast, TRIM28 knockdown enhanced antiviral cytokine induction. Two independent siRNAs effectively reduced TRIM28 protein levels (**Figure 1D**). Among them, siRNA #1 elicited a greater increase in SeV-induced *IFNB1* expression at the mRNA level (**Figure 1E**, left) and also enhanced IFN-β secretion, as confirmed by ELISA (**Figure 1E**, right), and was therefore selected for subsequent experiments. Time-course analysis demonstrated that TRIM28 depletion elevated the transcript levels of *IFNB1*, *ISG15*, *ISG54*, *ISG56*, and *IL1B* during SeV infection (**Figure 1F**), and similar transcriptional enhancement was observed following VSV infection (**Supplementary Figure 2C**). Together, these findings demonstrate that TRIM28 acts as a negative regulator of type I IFN and pro-inflammatory cytokine responses during RNA virus infection.

### TRIM28 promotes the replication of EV-71 and VSV

**Figure 2:**
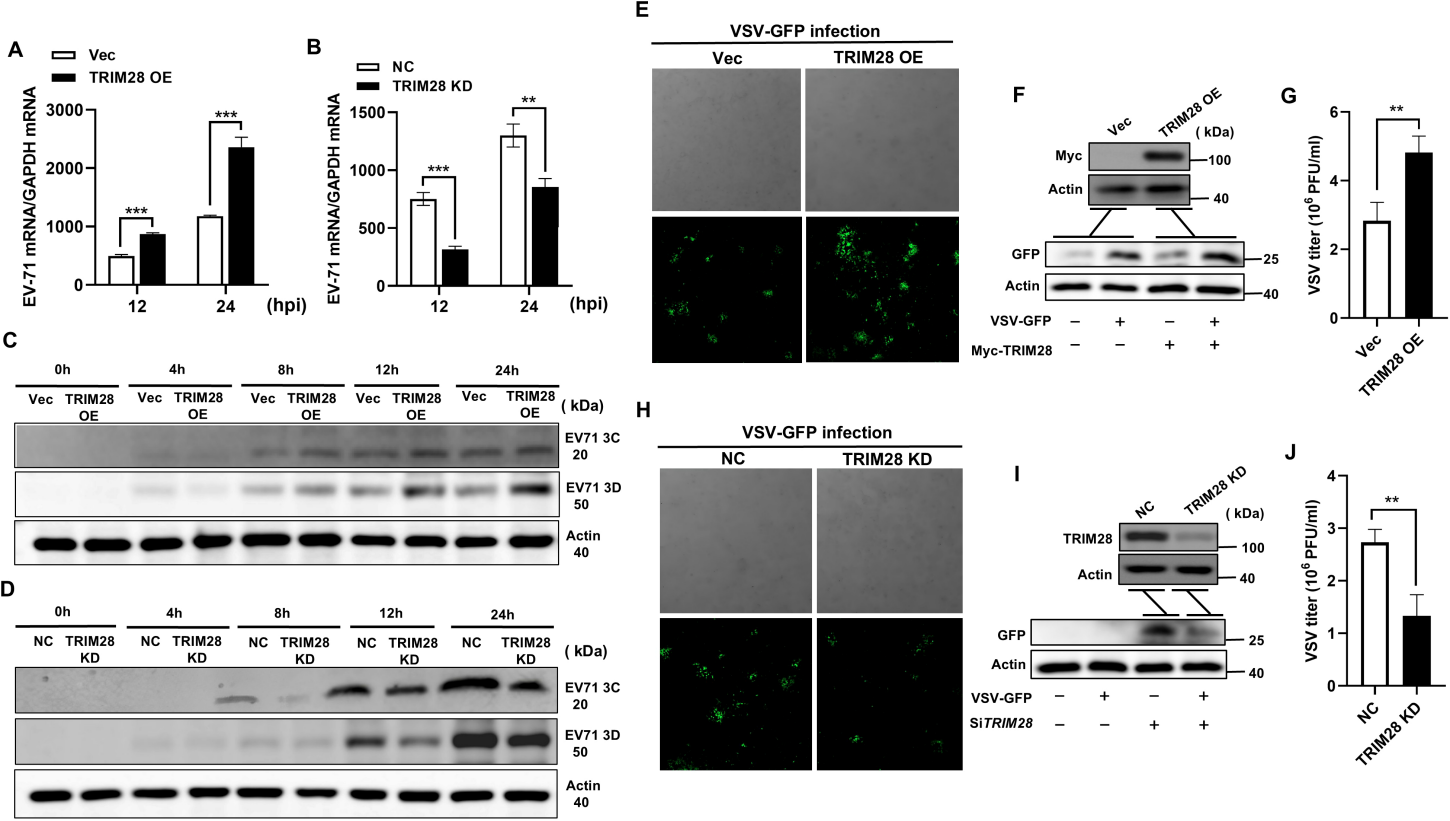
TRIM28 promotes the replication of EV-71 and VSV. **(A, B)** RT-qPCR analysis of EV-71 RNA levels in cells transfected with Vec or TRIM28-overexpressing plasmid **(A)**, and in cells transfected with NC or TRIM28-targeting siRNA **(B)**, following EV-71 infection for the indicated times. **(C, D)** Western blot analysis of EV-71 3C and 3D protein expression in TRIM28-overexpressing **(C)** or TRIM28-knockdown **(D)** cells at the indicated time points post-infection. **(E, H)** Fluorescence microscopy images showing VSV-GFP signal in TRIM28-overexpressing **(E)** or TRIM28-knockdown **(H)** HEK293T cells 12 hours after infection. **(F, I)** Western blot analysis of TRIM28 and VSV-GFP protein expression in TRIM28-overexpressing **(F)** or TRIM28-knockdown **(I)** cells following VSV-GFP infection. **(G, J)** Viral titration assays measuring VSV titers (PFU/mL) in control and TRIM28-overexpressing **(G)** or TRIM28-knockdown **(J)** cells. Data are presented as mean ± SD. Statistical significance was determined using two-tailed unpaired Student’s t-test; *P < 0.05, **P<0.01, ***P < 0.001.

To evaluate the effect of TRIM28 on RNA virus replication, we first examined the impact of TRIM28 overexpression or knockdown on enterovirus EV-71 replication. RT-qPCR analysis revealed that EV-71 RNA levels were significantly increased in TRIM28-overexpressing cells, whereas TRIM28 knockdown reduced viral RNA abundance (**Figures 2A, B**). Consistently, TRIM28 overexpression elevated the levels of EV-71 3C and 3D proteins, while TRIM28 depletion resulted in decreased expression of these viral proteins (**Figures 2C, D**). These results indicate that TRIM28 facilitates EV-71 replication.

We next examined whether TRIM28 also affects the replication of vesicular stomatitis virus (VSV). Using a VSV-GFP reporter, fluorescence microscopy revealed enhanced GFP signal in TRIM28-overexpressing cells, whereas TRIM28 knockdown markedly reduced GFP fluorescence (**Figures 2E, H**). Western blot analysis further confirmed increased VSV-GFP protein levels in TRIM28-overexpressing cells and decreased levels in TRIM28-knockdown cells (**Figures 2F, I**). Viral titration assays corroborated these findings (**Figures 2G, J**). Collectively, these results demonstrate that TRIM28 promotes the replication of RNA viruses *in vitro*.

### TRIM28 knockdown enhances type I IFN-mediated antiviral immunity *in vivo*

**Figure 3:**
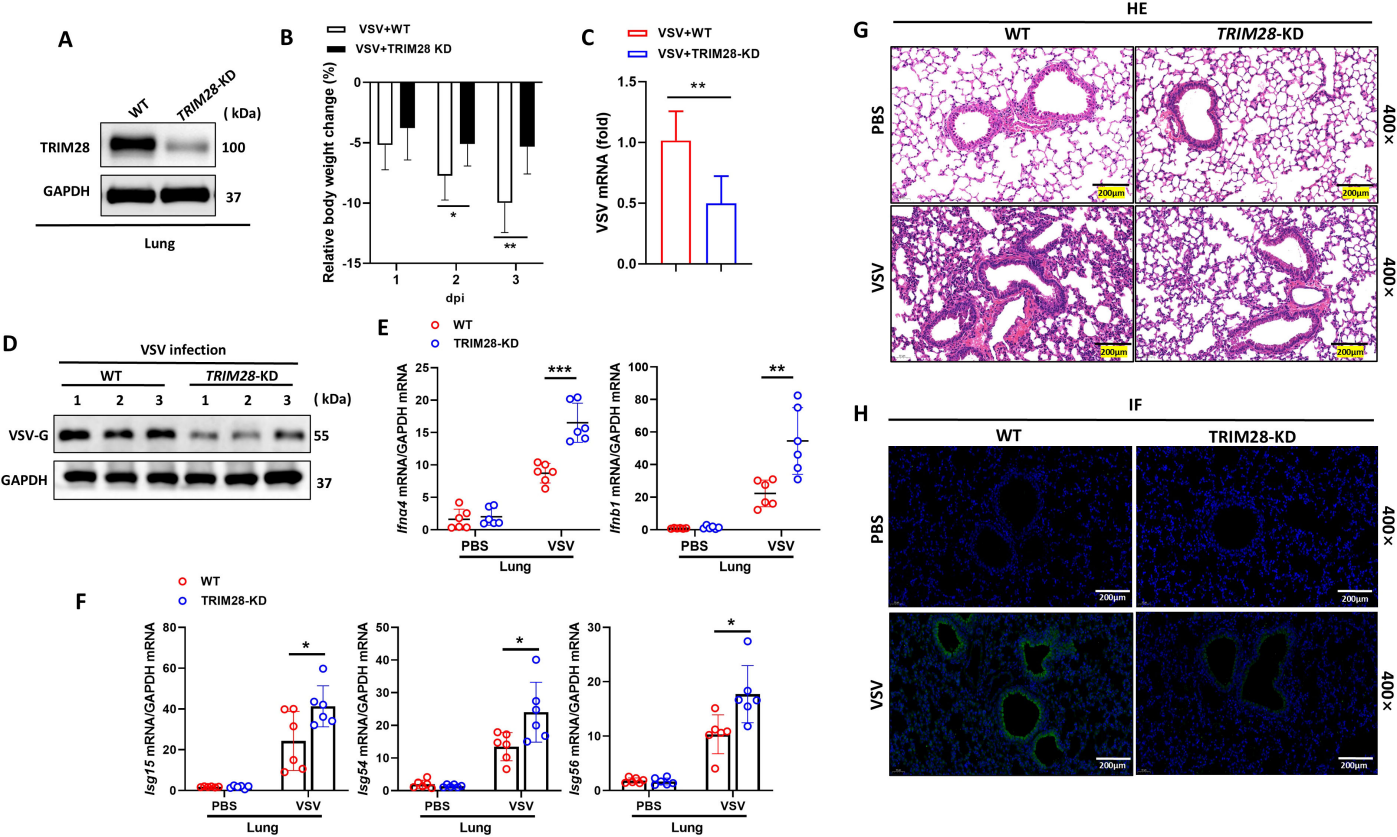
Knockdown of TRIM28 enhances antiviral innate immunity *in vivo.***(A)** Western blot analysis confirming TRIM28 knockdown in lung tissues from AAV-shRNA–treated mice. **(B)** Relative body weight change in WT and TRIM28-KD mice after VSV infection. **(C)** RT-qPCR analysis of VSV RNA levels in lung tissues at 3 dpi (n=6). **(D)** Western blot analysis of VSV-G protein expression in lung tissues from WT and TRIM28-KD mice at 3 dpi. **(E)** RT-qPCR analysis of *IFNα4* and *IFNβ1* mRNA levels in lung tissues from WT and TRIM28-KD mice treated with PBS or VSV. **(F)** Expression of interferon-stimulated genes (*Isg15*, *Isg54*, *Isg56*) in lung tissues after VSV infection. **(G)** Representative hematoxylin and eosin (H & E) staining of lung sections showing VSV-induced pathology (400×magnification; scale bar=200 μm). **(H)** Immunofluorescence imaging of VSV-GFP in lung tissues from WT and TRIM28-KD mice (400× magnification; scale bar=200 μm). Data are presented as mean ± SD. Statistical significance was determined using two-tailed unpaired Student’s t-test; *P < 0.05, **P<0.01.

We subsequently validated the role of TRIM28 in regulating antiviral responses *in vivo*. Given the embryonic lethality of global TRIM28 deletion, we generated a lung-restricted knockdown model using AAV-mediated TRIM28 shRNA via intratracheal administration. Three weeks after AAV delivery, mice were infected intraperitoneally with VSV. TRIM28 knockdown in lung tissues was confirmed by Western blot analysis (**Figure 3A**). Following infection, TRIM28-depleted mice exhibited significantly less body weight loss compared to controls (**Figure 3B**), along with reduced viral RNA levels and lower expression of the VSV-G protein in lung tissues (**Figures 3C, D**). Importantly, RT-qPCR analysis demonstrated that TRIM28 knockdown markedly increased the expression of type I IFN mRNAs (*Ifna4*, *Ifnb1*) (**Figure 3E**) and interferon-stimulated genes (*Isg15*, *Isg54*, *Isg56*) (**Figure 3F**) upon viral challenge. Histological analysis further revealed that VSV-induced lung injury was substantially alleviated in TRIM28-deficient mice. Whereas infected controls displayed severe diffuse nonsuppurative interstitial pneumonia and bronchiolitis, TRIM28 knockdown mice exhibited only minimal lesions with preservation of alveolar architecture (**Figure 3G**). Consistently, VSV-GFP infection resulted in a marked reduction of GFP fluorescence in the lungs of TRIM28-deficient mice (**Figure 3H**), indicating decreased viral replication. Taken together, these results demonstrate that TRIM28 promotes viral replication and exacerbates pulmonary pathology *in vivo*, and that this proviral effect is associated with its suppression of type I IFN signaling.

### TRIM28 inhibits TBK1 and IRF3 phosphorylation in the RIG-I signaling pathway

**Figure 4:**
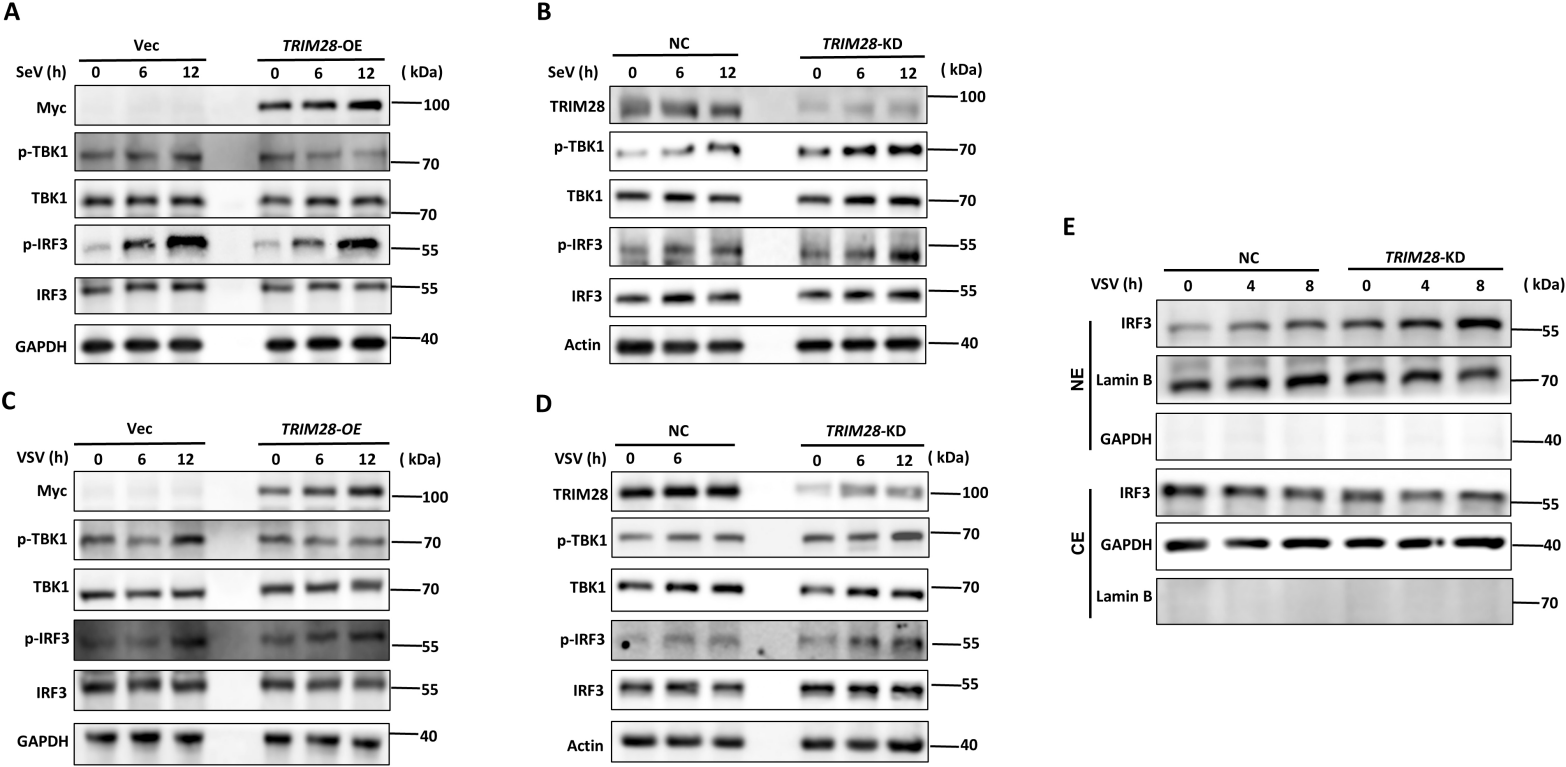
TRIM28 inhibits the phosphorylation and activation of key signaling molecules in the RIG-I pathway. **(A)** Western blot analysis of phosphorylation of TBK1 and IRF3 in TRIM28-overexpressing (TRIM28-OE) and control (Vec) HEK293T cells stimulated with SeV for the indicated times. **(B)** Phosphorylation of TBK1 and IRF3 in TRIM28-knockdown (TRIM28-KD) and NC cells following SeV infection, detected by immunoblotting. **(C, D)** Western blot analysis of TBK1, IRF3, and their phosphorylated forms in TRIM28-overexpressing **(C)** or TRIM28-knockdown **(D)** HEK293T cells after VSV stimulation for the indicated time points. **(E)** IRF3 subcellular localization in TRIM28-KD and NC cells after VSV infection. Cytoplasmic (CE) and nuclear extracts (NE) were prepared and analyzed by immunoblotting. GAPDH and Lamin B were used as cytoplasmic and nuclear markers, respectively.

Upon RNA virus infection, RIG-I-like receptors (RLRs) activate MAVS on the mitochondrial membrane, which subsequently recruits and activates TBK1, leading to IRF3 phosphorylation and type I IFN induction. To determine whether TRIM28 regulates this signaling cascade, we examined the phosphorylation of TBK1 and IRF3 in TRIM28-overexpressing and TRIM28-knockdown cells following RNA virus infection. In SeV-stimulated HEK293T cells, TRIM28 overexpression attenuated TBK1 and IRF3 phosphorylation, whereas TRIM28 knockdown enhanced their phosphorylation (**Figures 4A, B**). Similar regulatory effects were observed during VSV infection: TRIM28 deficiency increased TBK1 and IRF3 phosphorylation, while TRIM28 overexpression suppressed their activation (**Figures 4C, D**). To further assess the effect of TRIM28 on IRF3 activation, we evaluated IRF3 nuclear translocation by subcellular fractionation following VSV infection. As shown in **Figure 4E**, TRIM28 knockdown promoted nuclear accumulation of IRF3 compared to wild-type cells, indicating enhanced RIG-I signaling activity. Together, these findings demonstrate that TRIM28 negatively regulates the RIG-I signaling pathway by suppressing TBK1 and IRF3 phosphorylation and limiting IRF3 nuclear translocation.

### TRIM28 interacts with MAVS to negatively regulate the RIG-I signaling pathway

**Figure 5:**
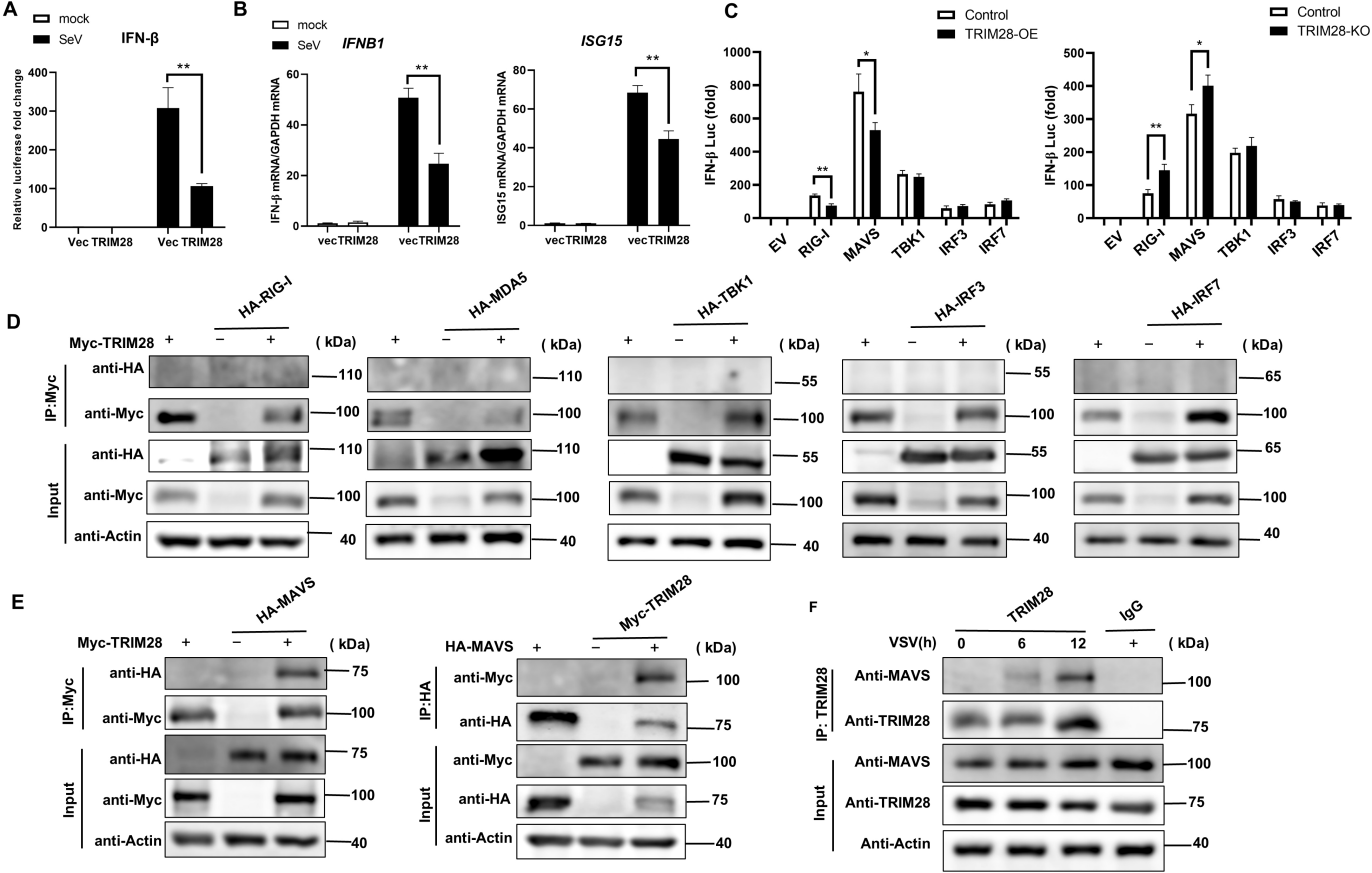
TRIM28 interacts with MAVS to inhibit the RIG-I antiviral signaling pathway. **(A)** Dual-luciferase reporter assays showing that TRIM28 overexpression suppresses SeV-induced *IFN-β* promoter activation in HEK293T cells. **(B)** qRT-PCR analysis of *IFNB1* and *ISG15* mRNA levels in HEK293T cells transfected with TRIM28 or control vector following SeV stimulation. **(C)** Luciferase reporter assays were performed to assess the impact of TRIM28 overexpression (left) or knockout (right) on *IFN-β* promoter activity in cells co-transfected with expression vectors for RIG-I, MAVS, TBK1, IRF3, or IRF7. TRIM28 selectively inhibited RIG-I- and MAVS-induced promoter activation but had minimal effect on downstream signaling components. **(D)** Co-IP experiments assessing physical interaction between Myc-tagged TRIM28 and individual HA-tagged RIG-I pathway components. TRIM28 did not interact with RIG-I, MDA5, TBK1, IRF3, or IRF7. **(E)** Reciprocal Co-IP assays showing a specific interaction between TRIM28 and MAVS under overexpression conditions. Myc-TRIM28 pulled down HA-MAVS (left) and, conversely, HA-MAVS co-precipitated Myc-TRIM28 (right). **(F)** Endogenous Co-IP analysis confirming the interaction between native TRIM28 and MAVS in VSV-infected cells, with increased binding detected upon infection. Data are presented as mean ± SD. Statistical significance was determined using two-tailed unpaired Student’s t-test; *P < 0.05, **P<0.01.

To further elucidate the molecular mechanism by which TRIM28 suppresses type I IFN production, we performed dual-luciferase reporter assays in HEK293T cells. Overexpression of TRIM28 markedly reduced SeV-induced activation of the *IFN-β* promoter (**Figure 5A**), and RT-qPCR analysis consistently demonstrated decreased *IFNB1* and *ISG15* transcript levels (**Figure 5B**). We next sought to identify the signaling node targeted by TRIM28 within the RIG-I pathway. Pathway checkpoint analysis revealed that TRIM28 inhibited RIG-I- or MAVS-induced, but not TBK1-, IRF3-, or IRF7-induced, *IFN-β* promoter activation (**Figure 5C**, left). In contrast, TRIM28 knockout enhanced *IFN-β* promoter activity in response to RIG-I and MAVS, without affecting signaling downstream of TBK1 or the IRFs (**Figure 5C**, right). These results suggest that MAVS is the key regulatory target of TRIM28 within the RIG-I signaling cascade.

To determine whether TRIM28 physically interacts with components of the RIG-I signaling cascade, co-immunoprecipitation (Co-IP) assays were performed. Myc-tagged TRIM28 was co-expressed with various HA-tagged adaptor proteins in HEK293T cells. TRIM28 did not interact with RIG-I, MDA5, TBK1, IRF3, or IRF7 (**Figure 5D**), but displayed a specific interaction with MAVS (**Figure 5E**, left). Reciprocal Co-IP assays confirmed this interaction, with Myc-TRIM28 pulling down HA-MAVS and vice versa (**Figure 5E**, right). Furthermore, endogenous Co-IP demonstrated that endogenous TRIM28 interacts with MAVS in VSV-infected cells, and the interaction became progressively stronger as infection progressed (**Figure 5F**). Together, these results identify MAVS as a specific interacting partner of TRIM28 and support a model in which TRIM28 negatively regulates RIG-I signaling by targeting MAVS to constrain downstream pathway activation.

### Viral infection triggers CRM1-dependent nuclear export of TRIM28 to the MAVS compartment

**Figure 6:**
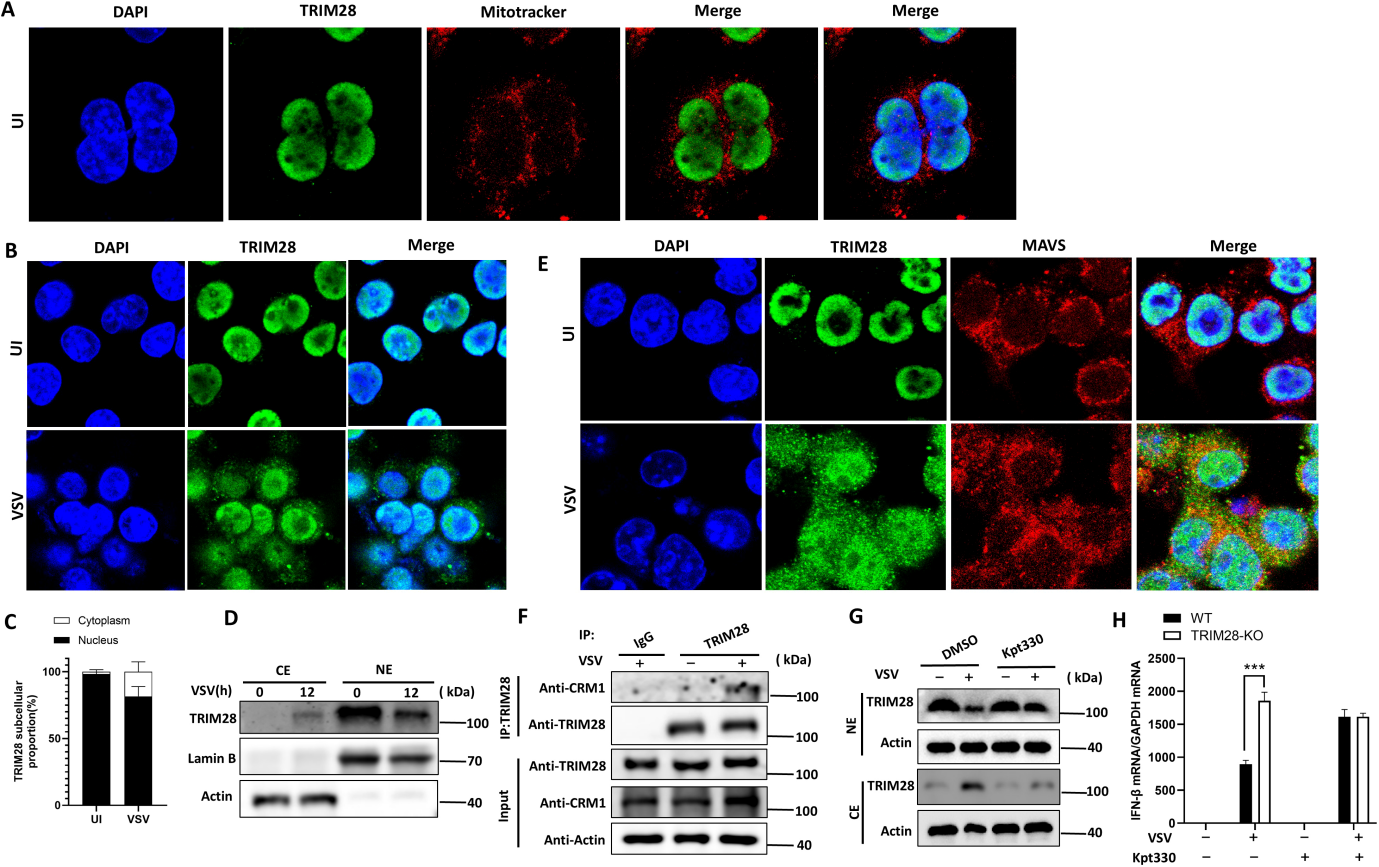
TRIM28 is exported from the nucleus via CRM1 and co-localizes with MAVS upon RNA virus infection. **(A)** Confocal microscopy showing TRIM28 localization (green) relative to nuclei (DAPI, blue) and mitochondria (Mitotracker, red) in unstimulated (UI) cells. **(B)** Immunofluorescence imaging showing TRIM28 nuclear-to-cytoplasmic translocation after VSV infection. **(C)** Quantification of TRIM28 subcellular distribution in nucleus and cytoplasm. **(D)** Western blot analysis of TRIM28 protein levels in cytoplasmic (CE) and nuclear (NE) extracts of cells treated with or without VSV for 12 h. **(E)** Confocal imaging showing colocalization of TRIM28 (green) and MAVS (red) in the cytoplasm of VSV-infected cells. **(F)** Co-IP analysis showing interaction between TRIM28 and CRM1 following VSV infection. **(G)** Treatment with the CRM1 inhibitor KPT-330 can largely block the nuclear export of TRIM28, thus retaining TRIM28 within the cell nucleus. **(H)** qRT-PCR analysis showing that blocking TRIM28 nuclear export phenocopies TRIM28 knockout, restoring *IFNB1* expression despite VSV infection. Data are presented as mean ± SD. Statistical significance was determined using two-tailed unpaired Student’s t-test; *P < 0.05, **P<0.01.

TRIM28 is predominantly localized in the nucleus under resting conditions, whereas MAVS is anchored on the outer mitochondrial membrane. To investigate how TRIM28 interacts with MAVS despite their distinct subcellular distributions, we examined TRIM28 localization by confocal microscopy. As expected, TRIM28 was mainly nuclear in unstimulated cells (**Figure 6A**). Upon VSV infection, a portion of TRIM28 redistributed from the nucleus to the cytoplasm (**Figures 6B, C**). Quantification of fluorescence intensity confirmed a reciprocal decrease in nuclear TRIM28 and a corresponding increase in cytoplasmic TRIM28 following infection (**Figure 6D**). In the cytoplasm, TRIM28 partially co-localized with MAVS (**Figure 6E**). To explore the mechanism underlying TRIM28 nuclear export, we focused on CRM1 (Exportin 1), a major nuclear export receptor that transports proteins and RNAs from the nucleus to the cytoplasm. Co-IP experiments demonstrated that TRIM28 interacts with CRM1 upon viral infection (**Figure 6F**). Notably, nuclear-cytoplasmic fractionation further showed that KPT-330 treatment markedly reduced VSV-induced redistribution of TRIM28, with increased retention of TRIM28 in the nuclear fraction and a concomitant decrease in the cytosolic fraction (**Figure 6G**). These results support that virus-triggered TRIM28 translocation is largely CRM1-dependent.

To test the functional relevance of TRIM28 nuclear export, we assessed *IFN-β* mRNA expression levels in the presence of KPT-330. VSV-induced *IFN-β* mRNA expression was significantly lower in wild-type cells compared to TRIM28-deficient cells. Importantly, KPT-330 treatment attenuated the difference between wild-type and TRIM28-deficient cells, supporting that CRM1 activity contributes to TRIM28-mediated suppression of *IFNB1* transcription (**Figure 6H**). Together, these findings demonstrate that RNA virus infection induces CRM1-dependent export of TRIM28 from the nucleus to the cytoplasm, facilitating its access to MAVS and subsequent suppression of RIG-I-mediated antiviral responses.

### TRIM28 promotes K48-linked ubiquitination of MAVS and requires its N-terminal RBCC domain

**Figure 7:**
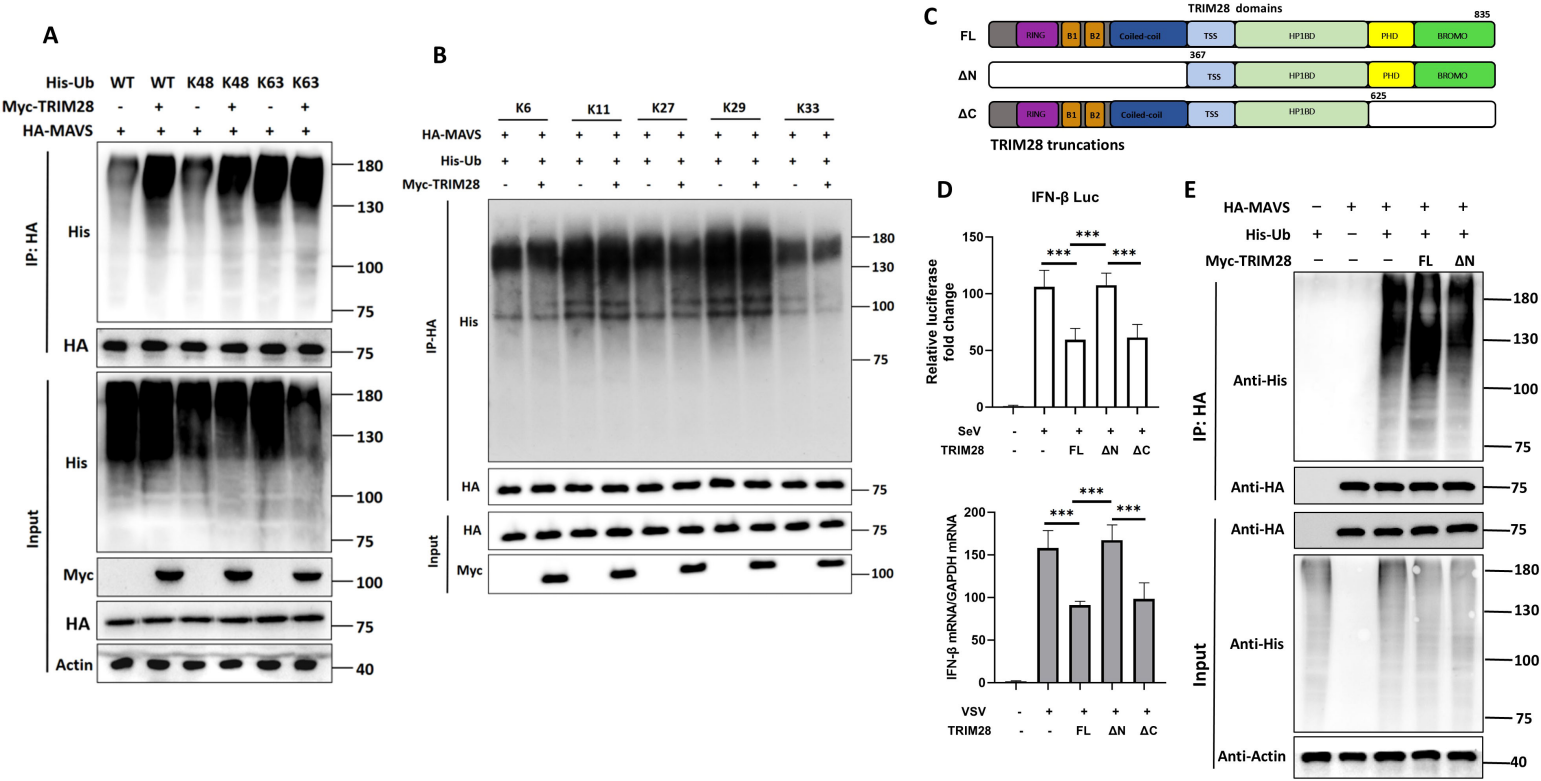
TRIM28 mediates K48-linked ubiquitination of MAVS through its N-terminal domain. **(A, B)** HEK293T cells were co-transfected with HA-MAVS, Myc-TRIM28, and the indicated ubiquitin constructs (wild-type, K48-only, or K63-only mutants in A; single-lysine mutants in B). Ubiquitination of MAVS was analyzed by immunoprecipitation (IP) with anti-HA antibody followed by immunoblotting with anti-His antibody. TRIM28 primarily induced K48-linked ubiquitination of MAVS, while other linkage types showed no comparable enhancement. **(C)** Schematic of full-length (FL) TRIM28 and truncation mutants ΔN (lacking RING-B-box-coiled-coil domain) and ΔC (lacking PHD-Bromo domain). **(D)***IFNB1* promoter activation was assessed by dual-luciferase reporter assay, and *IFN-β* mRNA expression was measured by RT-qPCR in cells transfected with TRIM28 or its truncation mutants. ΔN mutant failed to inhibit IFN-β activation. **(E)** Ubiquitination assays were performed to compare the E3 ligase activity of FL and ΔN TRIM28. *P < 0.05, **P<0.01, ***P < 0.001.

Given that TRIM28 contains an N-terminal RBCC domain characteristic of E3 ubiquitin ligases, and that it negatively regulates RIG-I signaling through interaction with MAVS, we next examined whether TRIM28 mediates MAVS ubiquitination. To this end, we co-transfected HEK293T cells with MAVS and a series of ubiquitin linkage mutants in which only a single lysine residue (K6, K11, K27, K29, K33, K48, and K63) was available for chain formation, in the presence or absence of TRIM28. The ubiquitination status of MAVS was then evaluated. The results revealed that TRIM28 specifically promoted K48-linked ubiquitination of MAVS, while exerting minimal effects on K63-linked or other lysine-linked chains (**Figures 7A, B**). Because K48-linked polyubiquitination typically targets substrates for proteasomal degradation, these findings suggest that TRIM28 may suppress MAVS signaling by facilitating its K48-dependent degradation.

To further define the functional domain responsible for this effect, we generated two TRIM28 truncated mutants: ΔN (lacking the N-terminal RING-B-box-coiled-coil domain) and ΔC (lacking the C-terminal PHD-BROMO domain) (**Figure 7C**). Dual-luciferase reporter assays and RT-qPCR analysis revealed that loss of the N-terminus (ΔN) significantly impaired the ability of TRIM28 to inhibit *IFN-β* promoter activation and *IFNB1* mRNA expression (**Figure 7D**). Consistently, the ΔN mutant failed to induce MAVS ubiquitination (**Figure 7E**). Together, these results support that TRIM28 promotes K48-linked ubiquitination of MAVS, and that the N-terminal RBCC domain is required for TRIM28-dependent MAVS ubiquitination and the inhibitory effect on IFN-β readouts in our experimental setting.

### MAVS K136 and K461 contribute to TRIM28-mediated ubiquitination and signaling repression

To identify potential ubiquitination sites on MAVS involved in TRIM28-mediated modification, we first conducted *in silico* prediction using the GPS-Uber algorithm. This analysis yielded several lysine residues with high prediction scores. Among them, K136 and K461 emerged as top-ranking candidates that had not been previously characterized. Based on their predicted scores and unexplored status, we selected these two sites for further validation.

**Figure 8:**
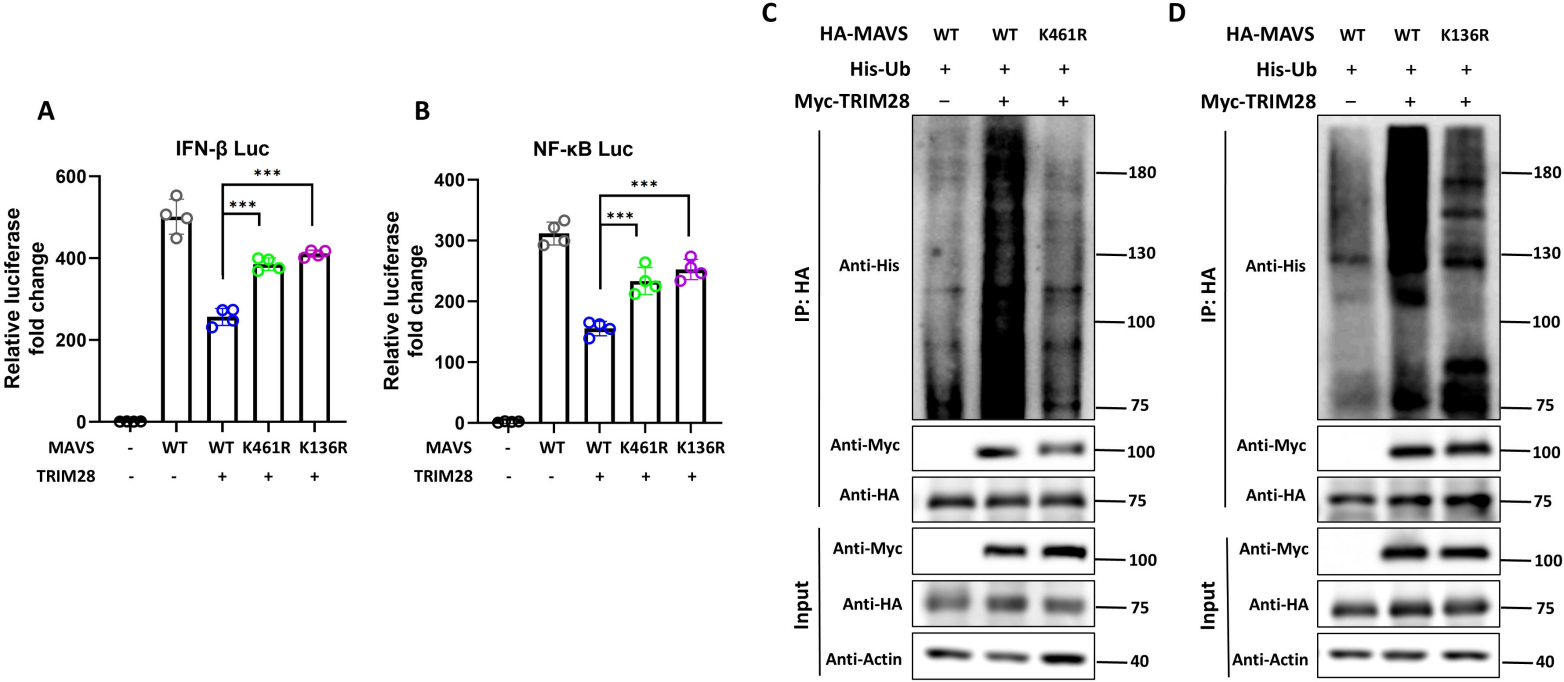
MAVS K136 and K461 is essential for TRIM28-mediate ubiquitination and suppression of antiviral responses. **(A, B)** HEK293T cells were co-transfected with wild-type or mutant MAVS (K136R or K461R), along with TRIM28, and subjected to dual-luciferase reporter assays to assess *IFN-β***(A)** and *NF-κB***(B)** promoter activity. TRIM28 significantly suppressed promoter activation induced by WT MAVS, but its inhibitory effect was attenuated in K136R- and K461R-expressing cells. **(C, D)** Ubiquitination assays were performed in HEK293T cells transfected with HA-tagged MAVS (WT or mutants), His-tagged ubiquitin, and Myc-tagged TRIM28. Cells were harvested for His pull-down, followed by immunoblotting with anti-His to detect ubiquitinated MAVS. Both K461R **(C)** and K136R **(D)** mutations markedly reduced TRIM28-mediated ubiquitination. *P < 0.05, **P<0.01.

We next generated MAVS point mutants (K136R and K461R) to evaluate their roles in TRIM28-mediated regulation of antiviral signaling. Mutation of either residue (K136R or K461R) markedly attenuated TRIM28-mediated inhibition of *IFN-β* and *NF-κB* promoter activation (**Figures 8A, B**), indicating that these residues are functionally important for TRIM28-dependent repression. To determine whether these sites are involved in TRIM28-mediated ubiquitination, we conducted ubiquitination assays using a transient overexpression system. HEK293T cells were co-transfected with plasmids expressing TRIM28, wild-type or mutant MAVS, and His-tagged ubiquitin. The results showed that both K136R and K461R mutations significantly diminished K48-linked ubiquitination of MAVS compared to the wild-type control (**Figures 8C, D**). Collectively, these findings indicate that K136 and K461 contribute to TRIM28-mediated K48-linked ubiquitination of MAVS, which is important for TRIM28-mediated restraint antiviral signaling.

## Discussion

TRIM28 exerts multifaceted functions in viral infection and host innate immunity (14). In this study, we show that TRIM28 acts as a negative regulator of RIG-I-mediated antiviral signaling. TRIM28 expression was upregulated in THP-1 cells following SeV infection and was positively correlated with virus replication. Functionally, TRIM28 overexpression suppressed virus-induced type I IFN responses, whereas its knockdown enhanced IFN production and restricted viral replication both *in vitro* and *in vivo*. Mechanistically, TRIM28 interacts with MAVS after cytoplasmic translocation and promotes its K48-linked ubiquitination, a modification typically associated with proteasomal degradation, thereby reducing downstream activation of TBK1 and IRF3/7. Together, these findings support a model in which TRIM28 attenuates MAVS-dependent signaling and acts as a negative regulator of antiviral innate immunity.

Previous studies have shown that TRIM28 is a multifunctional regulator during viral infection, not only modifying viral proteins to influence replication but also modulating key signaling molecules in host innate immune pathways (14). TRIM28-mediated SUMOylation has been reported to reduce MAVS abundance and suppress IRF7 activity, thereby attenuating RIG-I pathway activation (36, 38). In contrast, in another system, TRIM28 was shown to enhance antiviral signaling by promoting K63-linked ubiquitination of TBK1 and facilitating downstream cascade activation (34). These seemingly divergent findings suggest that the regulatory role of TRIM28 is context-dependent and closely associated with infection type and signaling node engagement. However, most of these conclusions were drawn from *in vitro* models, and the *in vivo* regulatory direction of TRIM28 during RNA virus infection has remained unclear.

In this study, using a lung-specific TRIM28 knockdown model, we demonstrated that TRIM28 functions as a negative regulator of the type I IFN pathway *in vivo*. Loss of TRIM28 in the lung enhanced type I IFN responses and reduced VSV replication and associated lung pathology, indicating that TRIM28 restrains antiviral responses during RNA virus infection. Consistently, TRIM28 deficiency also alleviated infection-induced body weight loss, reflecting reduced disease severity. TRIM28 knockdown in cultured cells likewise suppressed the replication of VSV and EV-71, indicating that this regulatory effect is applicable across different RNA viruses. In the analysis of RIG-I pathway activation, we observed that TRIM28 overexpression suppressed the phosphorylation of TBK1 and IRF3 following SeV and VSV infection, whereas TRIM28 knockdown enhanced their phosphorylation and promoted IRF3 nuclear translocation, consistent with the *in vivo* observations (36). Correspondingly, the expression of multiple ISGs was increased in TRIM28-deficient lungs, supporting that the elevated IFN response is functionally active. Together, these data support that TRIM28 negatively regulates downstream signaling of the RIG-I pathway and attenuates type I IFN responses. Therefore, we next sought to identify the specific signaling node within the RIG-I pathway through which TRIM28 exerts its inhibitory effect on antiviral responses.

MAVS is a central adaptor protein in the RIG-I-like receptor (RLR) pathway. Located on the outer mitochondrial membrane, it transduces viral RNA sensing signals from RIG-I/MDA5 to downstream TBK1 and IRF3, thereby inducing type I interferon production and mounting antiviral innate immune responses (39, 40). MAVS activity is tightly regulated by multiple post-translational modifications, including ubiquitination and SUMOylation (41). Therefore, identifying the E3 ubiquitin ligases that regulate MAVS under specific infection contexts is critical for understanding the spatiotemporal control of RIG-I signaling. TRIM28 belongs to the tripartite motif (TRIM) family of E3 ubiquitin ligases, many of which act as key modulators of innate immune signaling and can exert either positive or negative regulatory effects on antiviral responses (42). In this study, we compared the effects of TRIM28 on different signaling nodes within the RIG-I pathway. Dual-luciferase reporter assays showed that TRIM28 overexpression markedly suppressed IFNB1 promoter activation driven by RIG-I or MAVS, whereas its inhibitory effect on TBK1-, IRF3-, or IRF7-driven activation was comparatively weaker. Conversely, TRIM28 knockout enhanced IFNB1 activation specifically at the RIG-I/MAVS level. These data indicate that the suppressive activity of TRIM28 is focused at the MAVS signaling node. Consistent with this, Co-IP experiments supported an association between TRIM28 and MAVS, and the co-precipitation signal increased following VSV infection. Taken together, these results support a model in which TRIM28 primarily targets the MAVS node to attenuate RIG-I pathway activation and type I IFN responses. Notably, previous studies have reported that TRIM28 can suppress upstream RLR expression at the transcriptional/epigenetic level (43, 44): following viral infection, TRIM28 binds to and is enriched at *DDX58/IFIH1* promoter DNA, and TRIM28 deficiency leads to a reduction in promoter-associated repressive epigenetic features. Together, these findings are consistent with a transcriptional/epigenetic repressive role of TRIM28 on RIG-I/MDA5. By contrast, our study mainly observed a protein-level interaction between TRIM28 and MAVS, suggesting that TRIM28 may regulate the RLR pathway at different levels.

We therefore next asked how TRIM28 gains access to MAVS in the cytoplasm. The cytoplasmic relocalization of TRIM28 is functionally required for its regulation of the RIG-I-MAVS axis. Using a dual-luciferase reporter system combined with Co-IP assays, we identified and confirmed the interaction between TRIM28 and MAVS. Under resting conditions, TRIM28 is predominantly localized in the nucleus (10), whereas MAVS is anchored to the outer mitochondrial membrane; therefore, TRIM28 must translocate to the cytoplasm to physically access MAVS. Confocal microscopy and nuclear-cytoplasmic fractionation demonstrated that a subset of TRIM28 translocates from the nucleus to the cytoplasm upon VSV infection, where it partially co-localizes with MAVS, indicating that viral infection triggers its subcellular redistribution. Nuclear export of proteins is typically mediated by nuclear export sequences (NESs) recognized by export receptors such as CRM1 (Exportin 1), a well-characterized mediator of nucleocytoplasmic transport (45). Although TRIM28 has not yet been annotated with a confirmed NES in public databases, LocNES analysis predicts a leucine-rich region with NES-like characteristics. In our study, Co-IP experiments revealed that TRIM28 interacts with CRM1 during viral infection. Moreover, pharmacological inhibition of CRM1 using the specific inhibitor KPT-330 markedly reduced TRIM28 cytoplasmic relocalization and attenuated its suppressive effect on IFN-β expression. A recent study on the porcine reproductive and respiratory syndrome virus (PRRSV) similarly reported CRM1-dependent nuclear export of TRIM28 during infection, supporting the involvement of this pathway (46). Together, these findings indicate that CRM1 activity promotes VSV-induced TRIM28 redistribution to the cytoplasm, thereby facilitating its access to MAVS and supporting its inhibitory role in RIG-I signaling.

Ubiquitin can be polymerized through any of seven lysine residues-K6, K11, K27, K29, K33, K48, or K63, and the topological linkage specifies the post-translational fate and functional outcome of the modified protein substrate (47). K63-linked ubiquitination is generally involved in activating antiviral signaling and DNA repair pathways, whereas K48-linked chains typically target proteins for proteasomal degradation (48–50). K11-and K27-linked ubiquitination, less extensively characterized, have been implicated in mitophagy, apoptosis, and ER-associated degradation (51–54). Therefore, clarifying the specific ubiquitin linkage type catalyzed by a given E3 ligase on MAVS is essential for understanding the dynamic regulation of RIG-I signaling (55). MAVS is a key adaptor protein in the RIG-I-like receptor signaling cascade, and its activity is tightly modulated by ubiquitination events governed by various TRIM family proteins (56, 57). For example, TRIM22 enhances MAVS antiviral signaling by promoting its K63-linked polyubiquitination (58), TRIM29 promotes MAVS degradation via K29-linked ubiquitin chains (59), and TRIM44 counteracts K48-linked ubiquitination to stabilize MAVS (60). Previous work has also suggested that TRIM28 can promote K48-linked ubiquitination of MAVS (36). Consistent with these findings, our data show that TRIM28 specifically enhances K48-linked polyubiquitination of MAVS without significantly altering other ubiquitin chain types. Importantly, our study identifies Lys136 and Lys461 as functionally relevant ubiquitination sites on MAVS, and indicates that CRM1-dependent cytoplasmic relocalization of TRIM28 is required for this modification. Moreover, *in vivo* evidence from the lung-specific TRIM28 knockdown model supports that this regulatory mechanism correlates with enhanced type I IFN responses and reduced viral replication.

Although previous work has mapped TRIM28-MAVS association to C-terminal domains, how the domain-specific MAVS binding is coupled to the ubiquitination output and downstream signaling regulation may vary across experimental settings (36). The catalytic activity of TRIM family E3 ligases is known to rely on the N-terminal RING domain together with the adjacent B-box and coiled-coil motifs, which form the canonical RBCC module (25, 61, 62). In this study, we used TRIM28 truncation mutants to demonstrate that the N-terminal region is required for mediating K48-linked ubiquitination of MAVS. Loss of this domain markedly reduced TRIM28-dependent MAVS ubiquitination and substantially impaired TRIM28-mediated suppression of the RIG-I signaling pathway. These results refine the mechanistic understanding of TRIM28-mediated MAVS ubiquitination and underscore the importance of the RBCC module-particularly the RING domain-for TRIM28’s E3 ligase activity. Precise delineation of ubiquitin-acceptor sites within substrates is fundamental for dissecting degradation mechanisms, signaling dynamics, and E3 ligase specificity (63–65). Using the GPS-Uber online prediction tool (66), we identified multiple lysine residues on MAVS with high predicted scores as candidate ubiquitination acceptor sites, including K7, K10, K371, K500, K136, and K461 (36). Given that several MAVS lysines have been examined previously, we prioritized K136 and K461 for experimental validation in our system. Mutating K136 or K461 to arginine markedly reduced TRIM28-mediated K48-linked ubiquitination of MAVS and blunted its inhibitory effect on IFN signaling, indicating that these residues contribute to TRIM28-dependent MAVS regulation. These findings extend the ubiquitination site profile of MAVS and support the conclusion that TRIM28 modulates RIG-I signaling at the MAVS level.

Given that excessive type I IFN responses can contribute to immunopathology in multiple viral infections, targeted modulation of TRIM28 or its regulation of MAVS may provide a basis for fine-tuning antiviral responses, although further studies will be required to define the feasibility and specificity of such approaches.

## Conclusion

**Figure 9:**
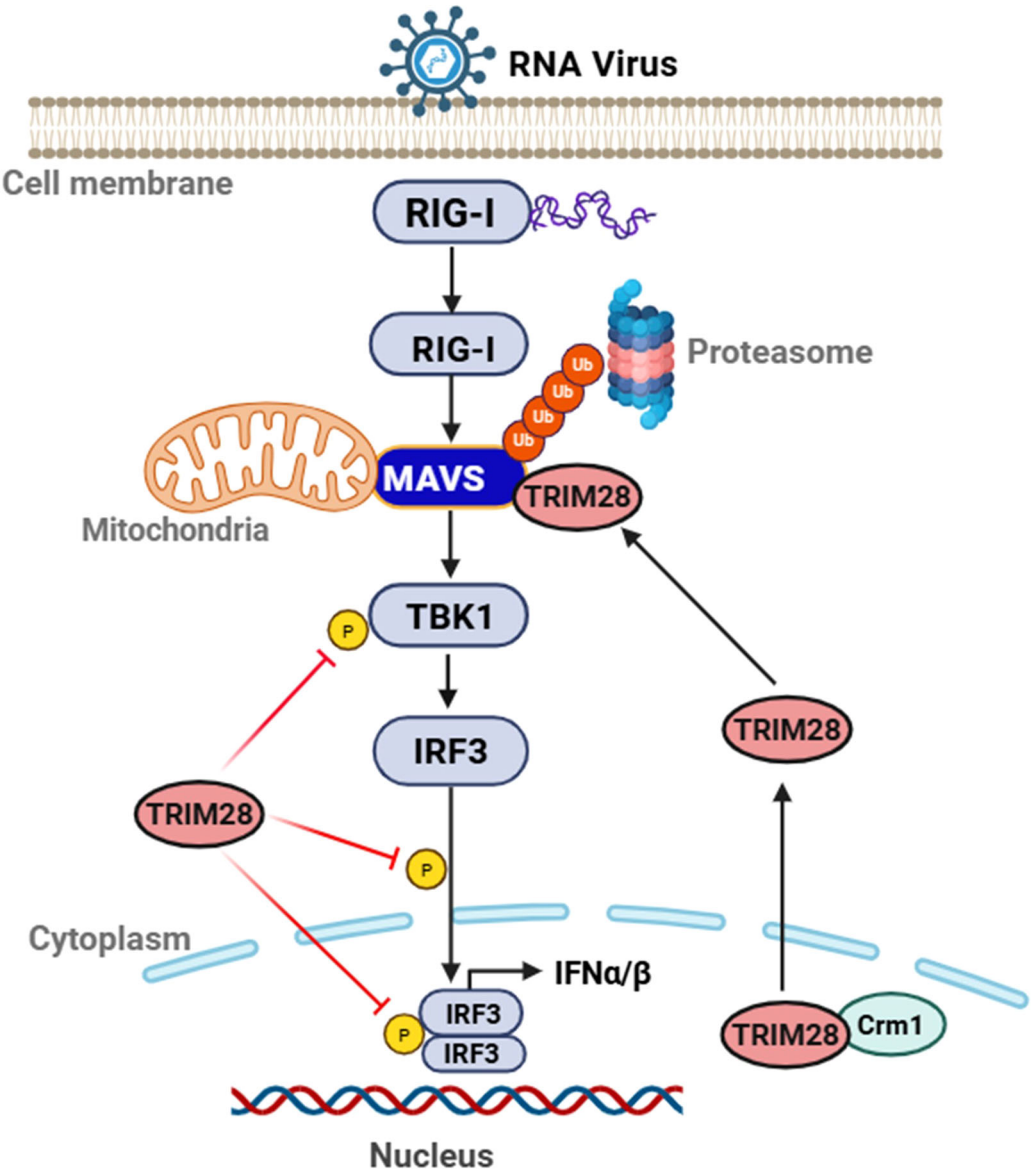
Proposed model of TRIM28-mediated regulation of the RIG-I-MAVS antiviral signaling pathway. In resting cells, TRIM28 is mainly nuclear. Upon RNA virus infection, it undergoes CRM1-dependent export from the nucleus to the cytoplasm, where it binds to MAVS and promotes K48-linked ubiquitination, leading to MAVS proteasomal degradation. This attenuates TBK1 activation and IRF3 phosphorylation, suppressing IFN-α/β production. TRIM28 also directly inhibits TBK1 and IRF3 activity. Its N-terminal RBCC domain is required for E3 ligase function. Mutation of MAVS at K136 or K461 impairs TRIM28-mediated ubiquitination and restores host antiviral signaling, underscoring the role of TRIM28 as a negative regulator of RIG-I-MAVS-mediated innate immunity.

In summary, this study identifies TRIM28 as a negative regulator of type I IFN responses during RNA virus infection. Upon infection, TRIM28 undergoes predominantly CRM1-dependent nuclear export and relocalizes to the cytoplasm, where it interacts with the mitochondrial adaptor MAVS. TRIM28 promotes K48-linked ubiquitination of MAVS in an N-terminal RBCC region-dependent manner, facilitating MAVS degradation and reducing activation of TBK1 and IRF3, thereby dampening antiviral signaling. Furthermore, we identify Lys136 and Lys461 on MAVS as functionally relevant ubiquitination sites that contribute to TRIM28-mediated suppression of IFN-β and NF-κB activation. These findings refine the current understanding of TRIM28 function in innate immune regulation by identifying MAVS as a direct ubiquitination substrate of TRIM28. Future studies exploring how TRIM28 activity is regulated across different infection contexts may help delineate its broader roles in antiviral and inflammatory responses (**Figure 9**).

## Data Availability

The datasets presented in this study can be found in online repositories. The names of the repository/repositories and accession number(s) can be found below: PRJNA1356945 (https://www.ncbi.nlm.nih.gov/bioproject/PRJNA1356945).

## Supplementary material

Supplementary Figure 1 TRIM28 is upregulated in response to viral infection in THP-1. (A) Heatmap showing the differentially expressed TRIM family genes in THP-1 following the infection with SeV for 12 h, based on RNA sequencing data from three independent biological replicates (SeV1, SeV2, SeV3) and corresponding uninfected controls (UI1, UI2, UI3). (B-C) RT-qPCR validation of representative upregulated and downregulated TRIM genes after SeV infection. (D-E) Western blot and RT-qPCR analysis showing time-dependent upregulation of TRIM28 protein (D) and mRNA (E) following SeV infection. (F-G) Western blot and RT-qPCR analysis showing TRIM28 protein (F) and mRNA (G) expression kinetics in THP-1 after VSV infection. Data are expressed as mean ± SD.

Supplementary Figure 2 TRIM28 regulates antiviral gene expression upon SeV, poly(I: C), or VSV stimulation. (A) Dose-dependent effects of TRIM28 overexpression on SeV-induced transcription of *IFNB1*, *ISG15*, *ISG54*, *ISG56*, and *IL1B* in HEK293T cells. Increasing amounts of the Myc-tagged TRIM28 plasmid were transfected, and mRNA levels were determined by RT-qPCR after 12 h of SeV infection. (B) Dose-dependent inhibition of *IFNB1, ISG15, ISG54, ISG56, and IL1B* in TRIM28-overexpressing HEK293T cells stimulated with poly(I: C) for 12 h. (C) TRIM28 knockdown (TRIM28-KD) enhanced the transcription of *IFNB1*, *ISG15*, *ISG54*, *ISG56*, and *IL1B* in HEK293T cells infected with vesicular stomatitis virus (VSV). Cells transfected with a non-targeting control siRNA (NC) were used as controls. All mRNA levels were normalized to GAPDH. Data are presented as mean ± SD from three independent experiments. *P* < 0.05, *P* < 0.01, *P* < 0.001.
