# Supplementary figures and images for: CRM1-dependent nuclear export of TRIM28 promotes MAVS K48-linked ubiquitination and suppresses RIG-I-mediated antiviral response (part 1 of 3)
Source: Front Immunol. 2026 Mar 24;17:1744833. doi: 10.3389/fimmu.2026.1744833 (PMC13053320; doi:10.3389/fimmu.2026.1744833)

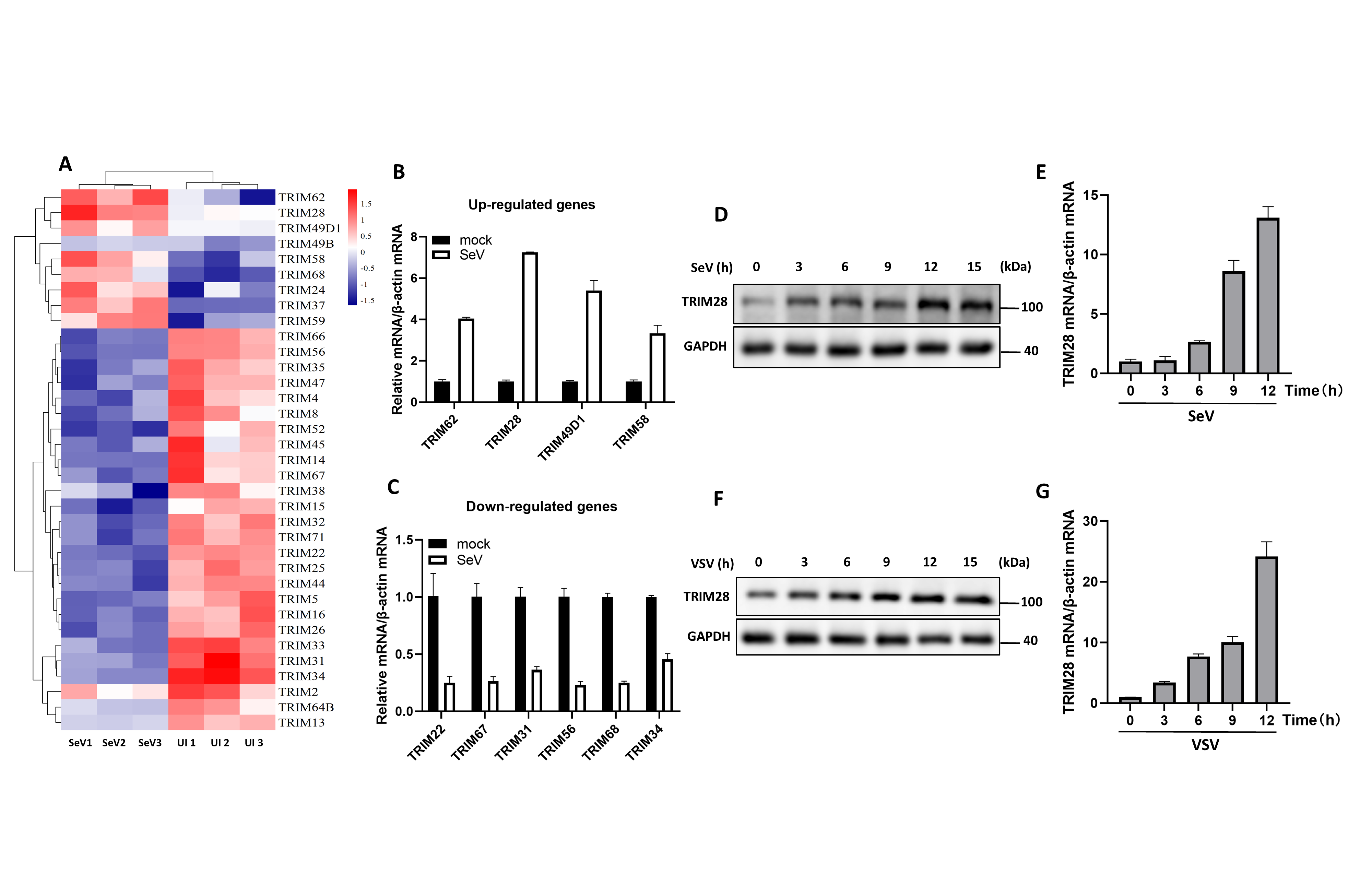

Supplement: Supplementary Figure 1 — TRIM28 is upregulated in response to viral infection in THP-1. (A) Heatmap showing the differentially expressed TRIM family genes in THP-1 following the infection with SeV for 12 h, based on RNA sequencing data from three independent biological replicates (SeV1, SeV2, SeV3) and corresponding uninfected controls (UI1, UI2, UI3). (B-C) RT-qPCR validation of representative upregulated and downregulated TRIM genes after SeV infection. (D-E) Western blot and RT-qPCR analysis showing time-dependent upregulation of TRIM28 protein (D) and mRNA (E) following SeV infection. (F-G) Western blot and RT-qPCR analysis showing TRIM28 protein (F) and mRNA (G) expression kinetics in THP-1 after VSV infection. Data are expressed as mean ± SD. [file Image1.tif]

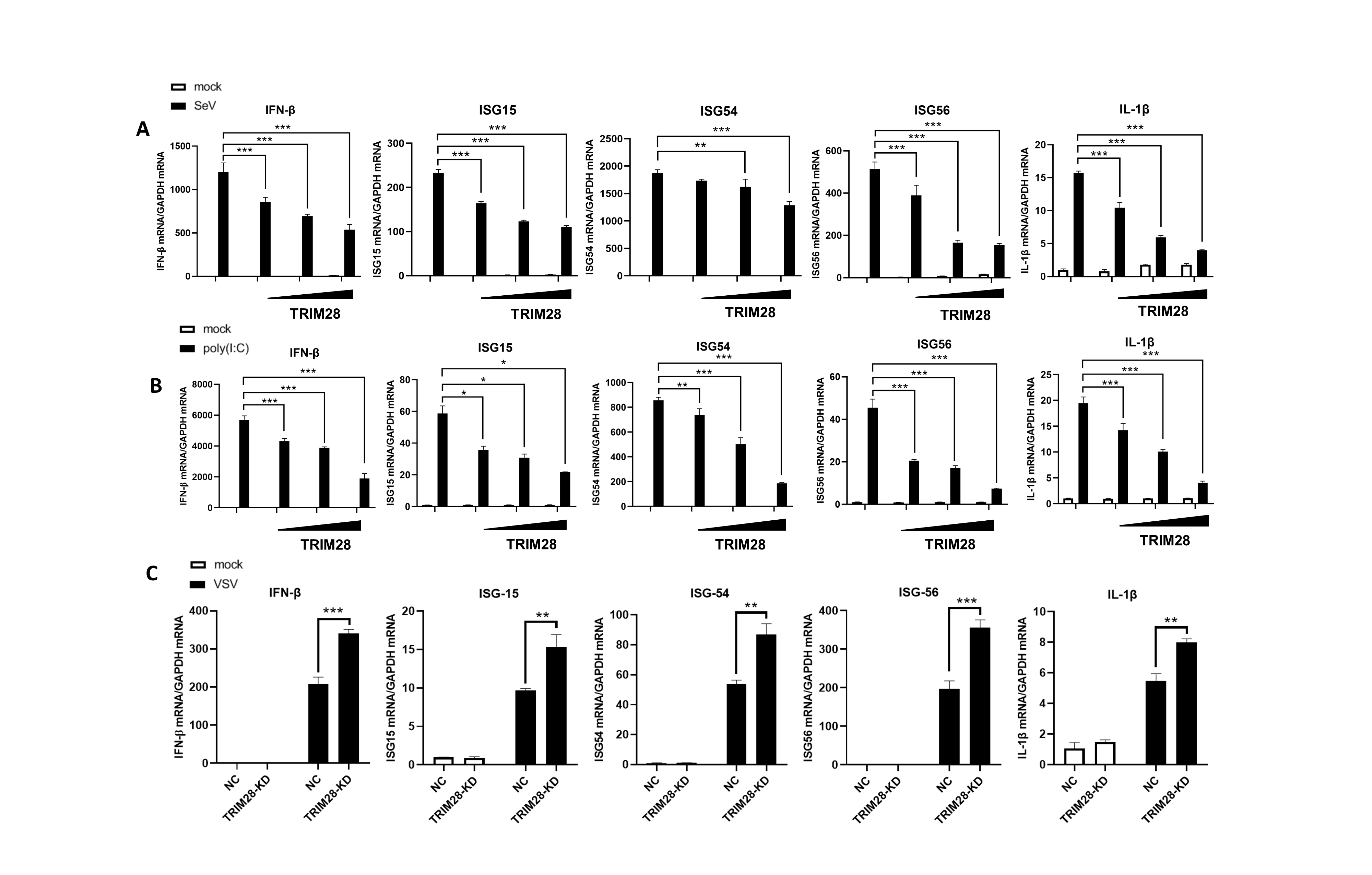

Supplement: Supplementary Figure 2 — TRIM28 regulates antiviral gene expression upon SeV, poly(I: C), or VSV stimulation. (A) Dose-dependent effects of TRIM28 overexpression on SeV-induced transcription of IFNB1, ISG15, ISG54, ISG56, and IL1B in HEK293T cells. Increasing amounts of the Myc-tagged TRIM28 plasmid were transfected, and mRNA levels were determined by RT-qPCR after 12 h of SeV infection. (B) Dose-dependent inhibition of IFNB1, ISG15, ISG54, ISG56, and IL1B in TRIM28-overexpressing HEK293T cells stimulated with poly(I: C) for 12 h. (C) TRIM28 knockdown (TRIM28-KD) enhanced the transcription of IFNB1, ISG15, ISG54, ISG56, and IL1B in HEK293T cells infected with vesicular stomatitis virus (VSV). Cells transfected with a non-targeting control siRNA (NC) were used as controls. All mRNA levels were normalized to GAPDH. Data are presented as mean ± SD from three independent experiments. P < 0.05, P < 0.01, P < 0.001. [file Image2.tif]

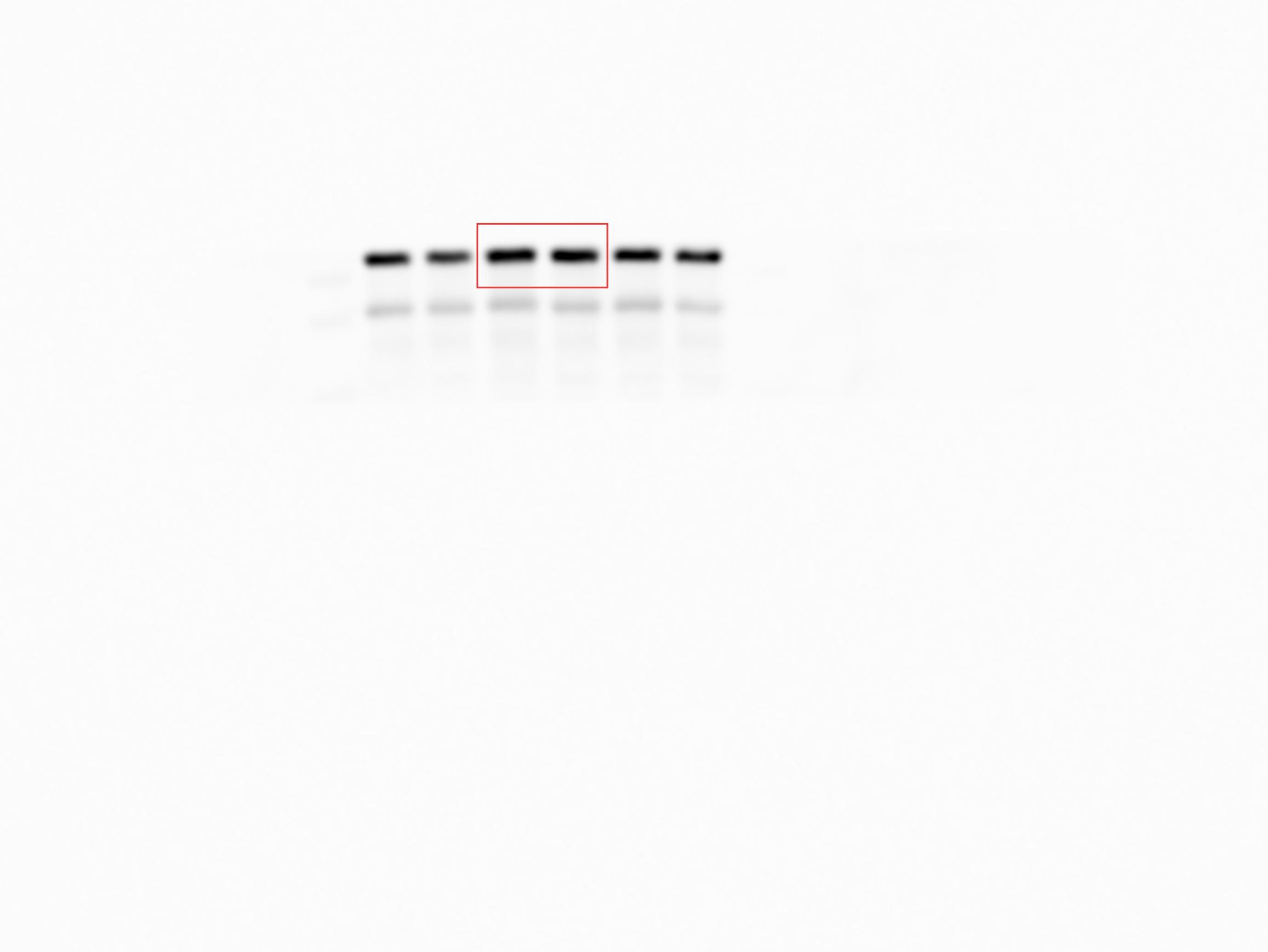

Supplement: Supplementary file 3 [file DataSheet1.zip › Fig1A Actin edited showing band.jpg]

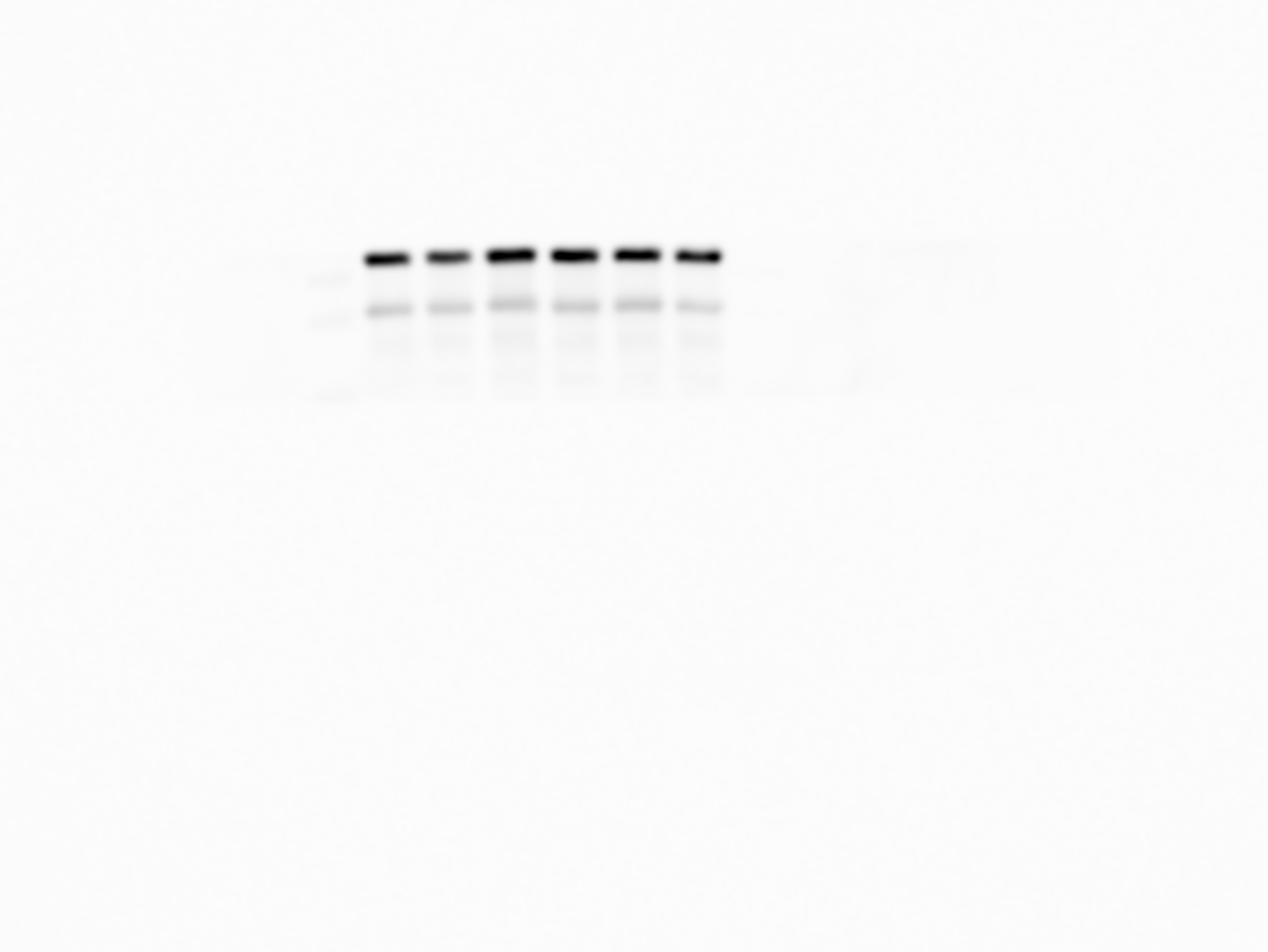

Supplement: Supplementary file 3 [file DataSheet1.zip › Fig1A Actin.tif]

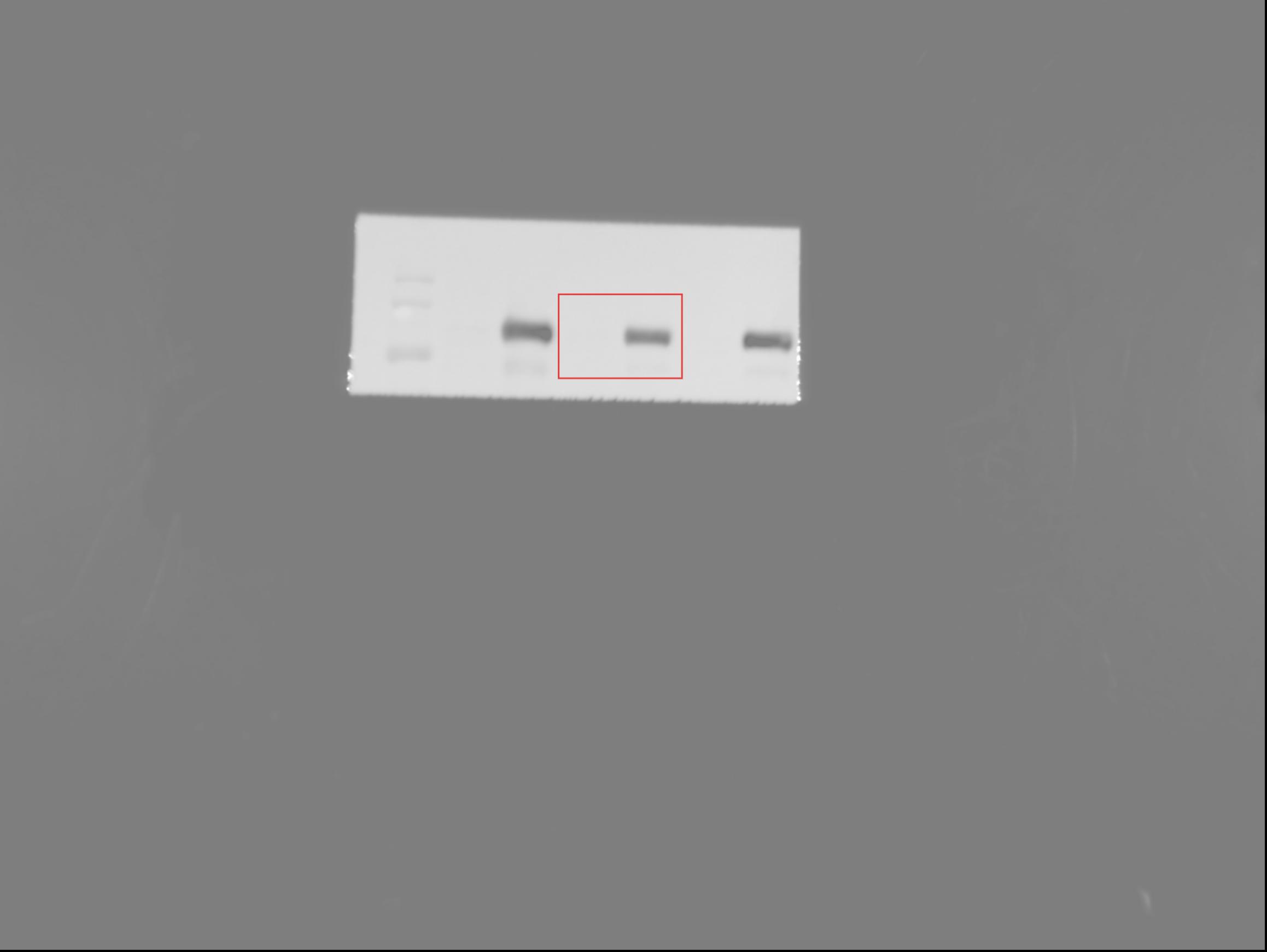

Supplement: Supplementary file 3 [file DataSheet1.zip › Fig1A Myc edited showing band.jpg]

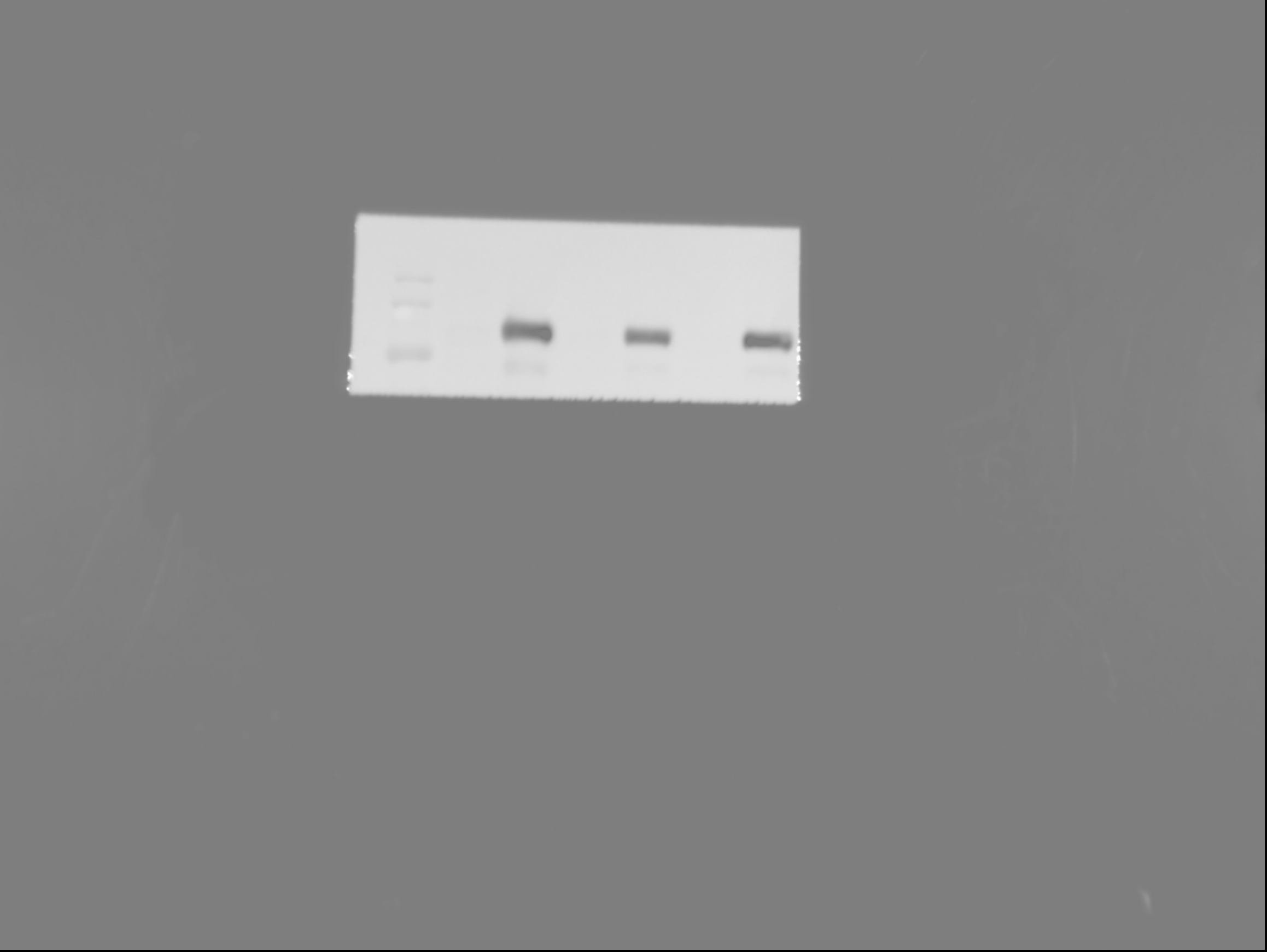

Supplement: Supplementary file 3 [file DataSheet1.zip › Fig1A Myc.tif]

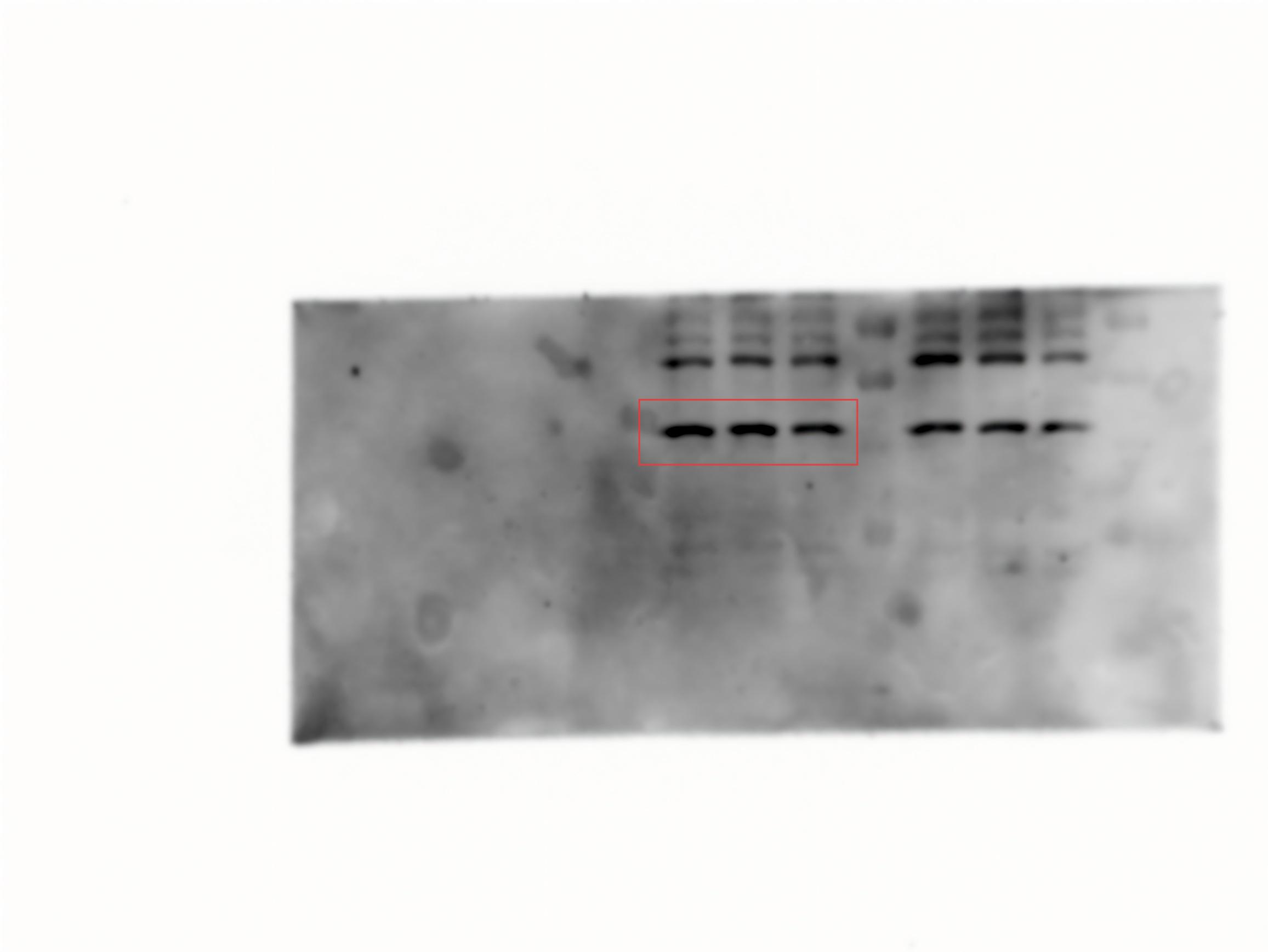

Supplement: Supplementary file 3 [file DataSheet1.zip › Fig1D Actin edited showing band.jpg]

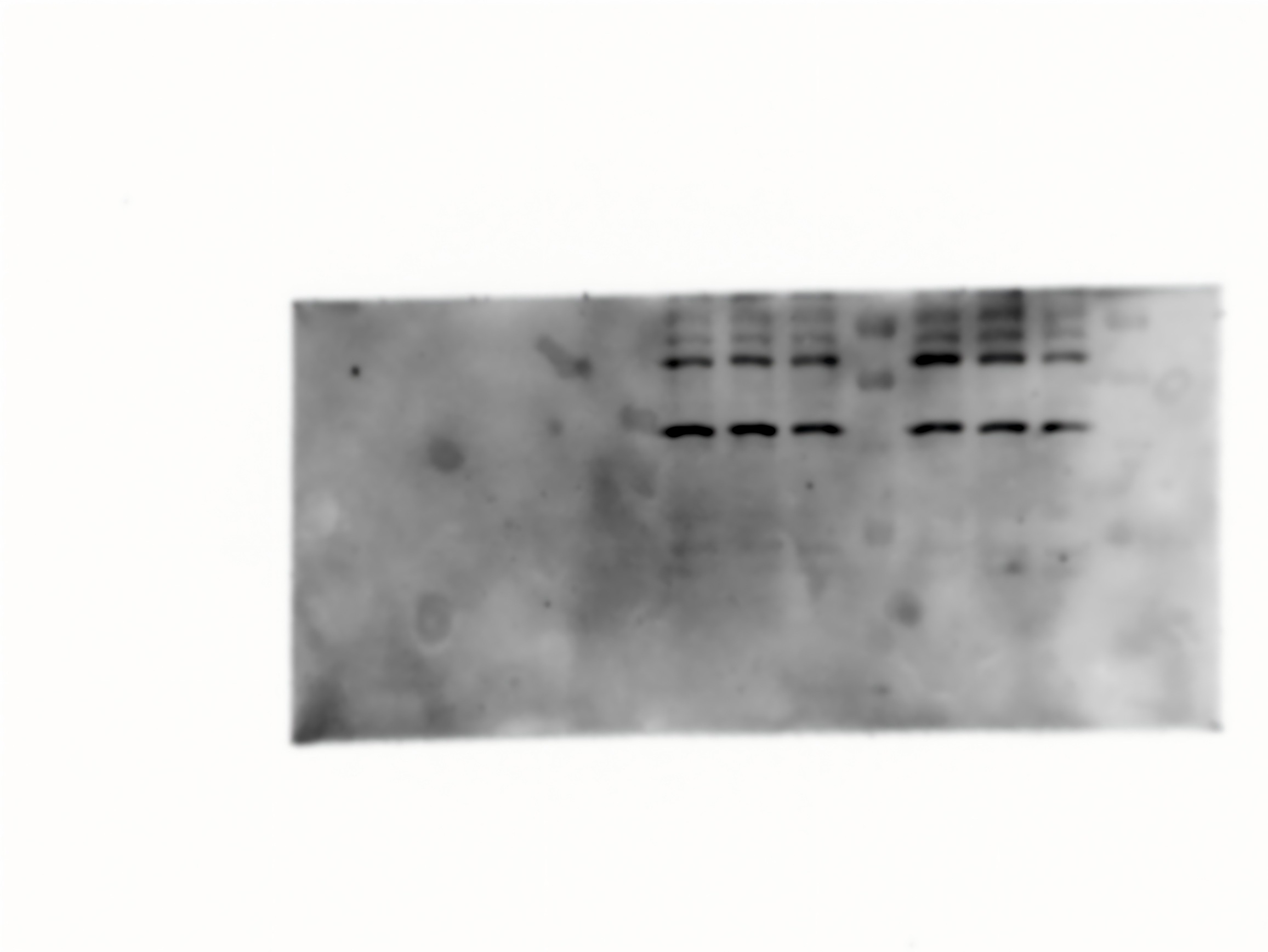

Supplement: Supplementary file 3 [file DataSheet1.zip › Fig1D Actin.jpg]

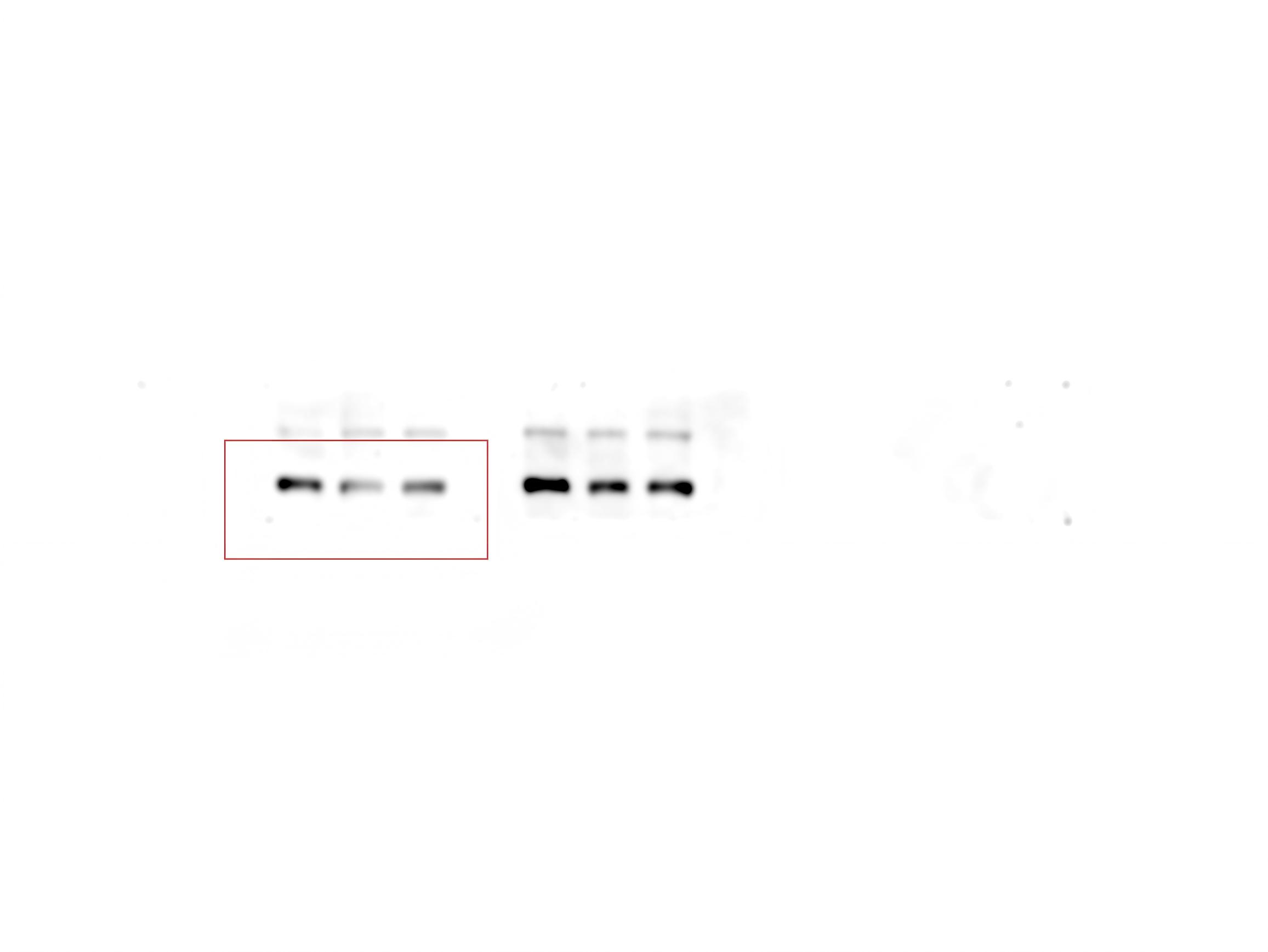

Supplement: Supplementary file 3 [file DataSheet1.zip › Fig1D TRIM28 edited showing band.jpg]

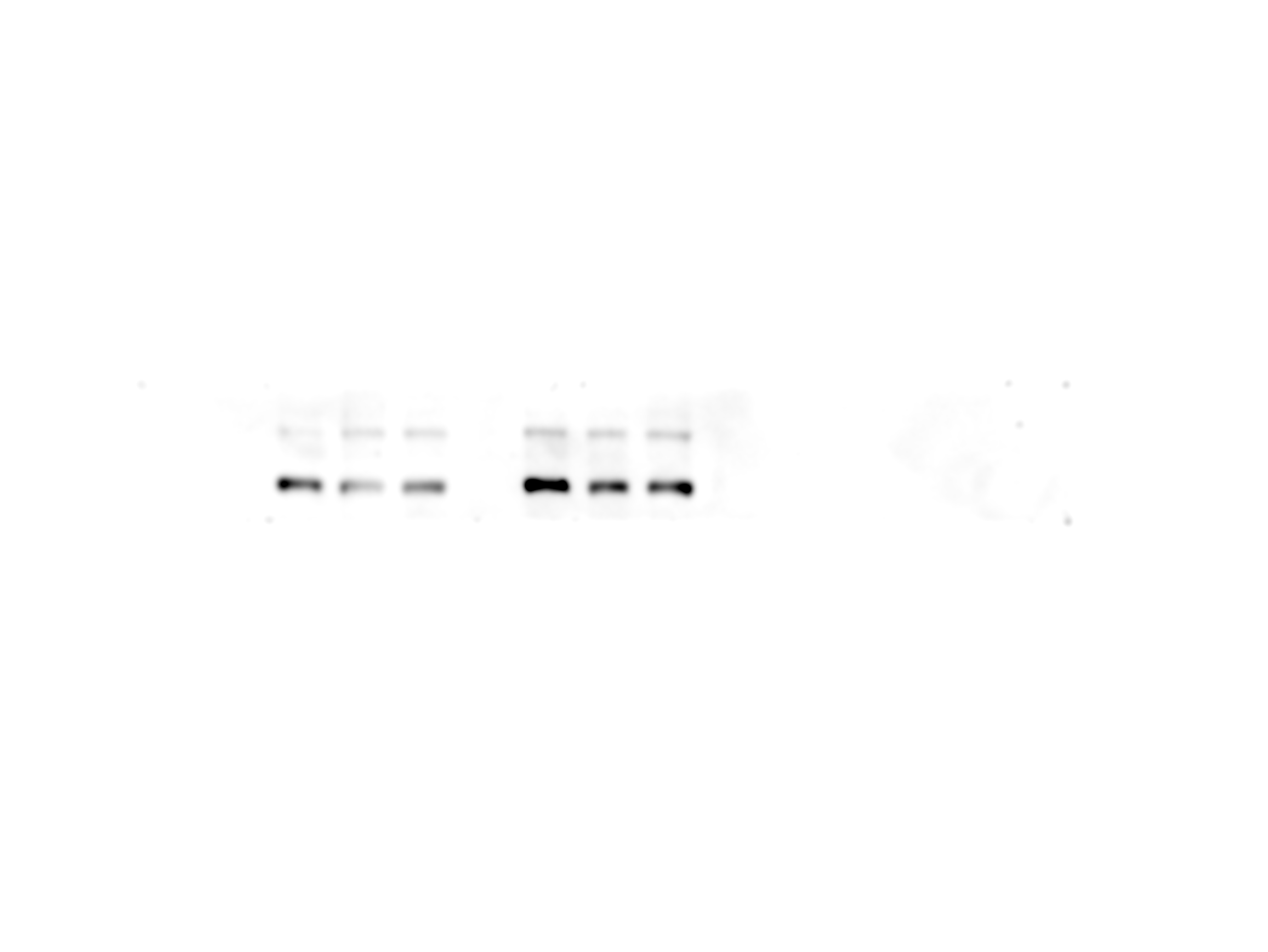

Supplement: Supplementary file 3 [file DataSheet1.zip › Fig1D TRIM28.tif]

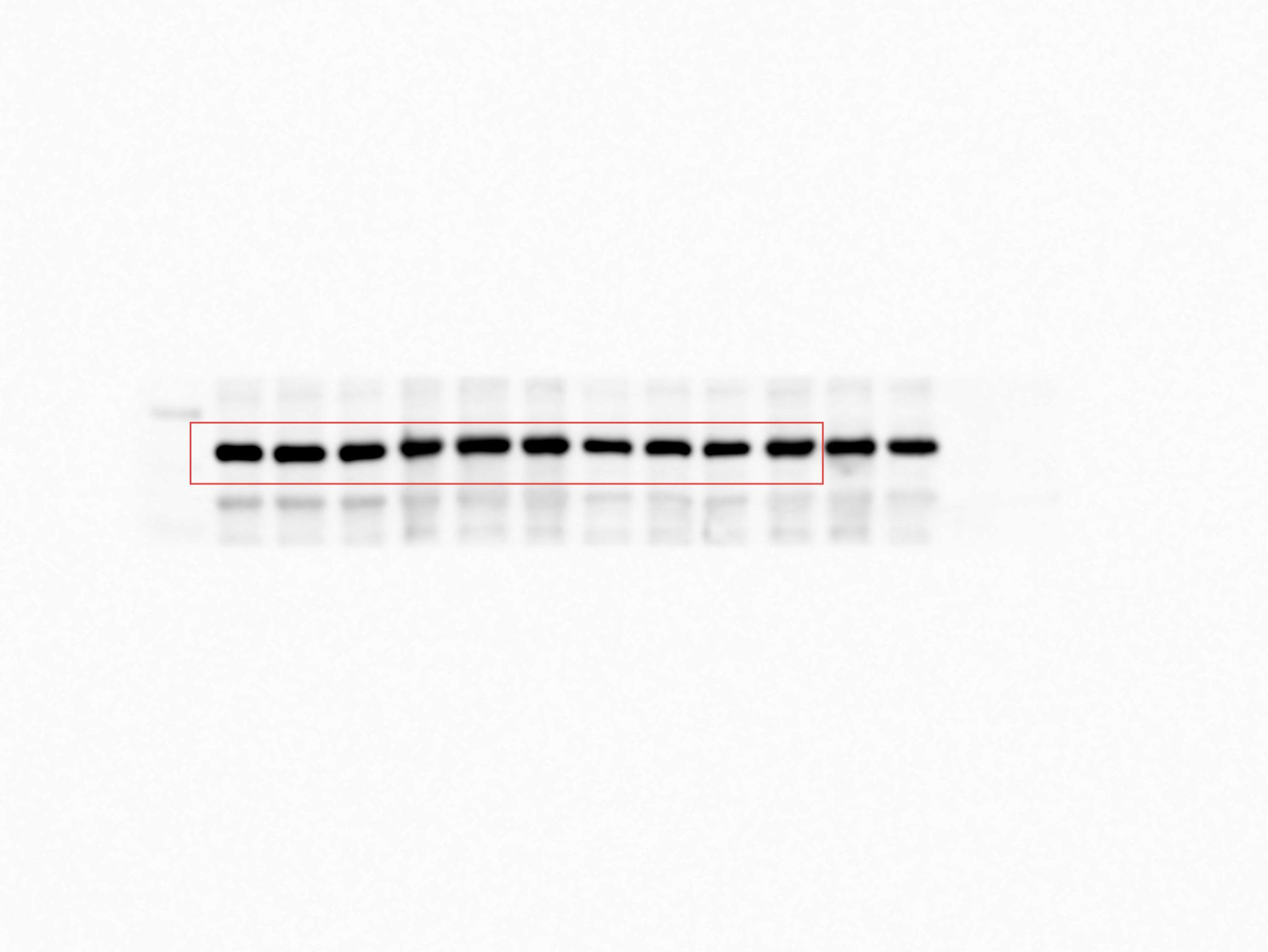

Supplement: Supplementary file 3 [file DataSheet1.zip › Fig2C ACTIN edited showing band.jpg]

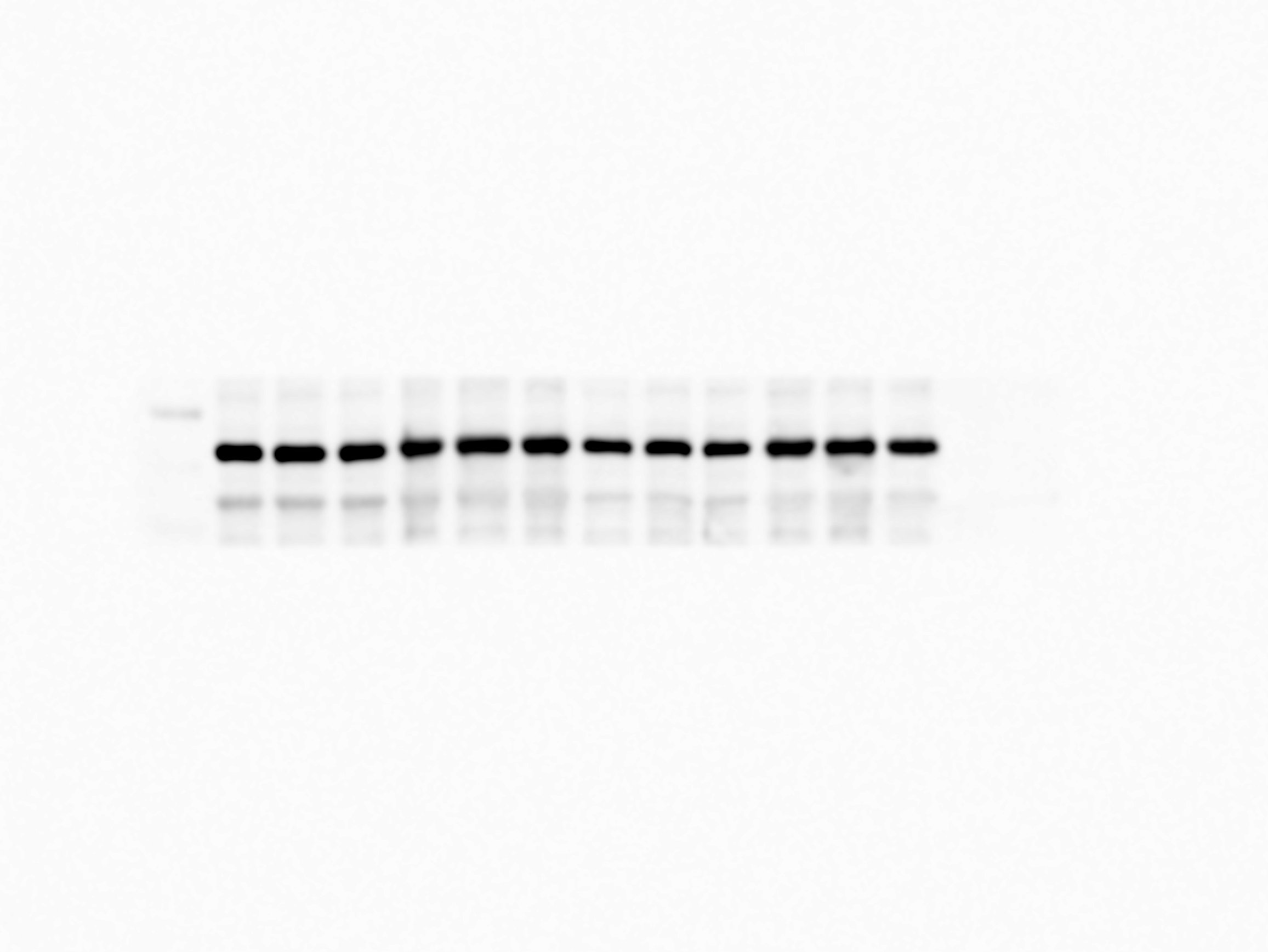

Supplement: Supplementary file 3 [file DataSheet1.zip › Fig2C ACTIN.jpg]

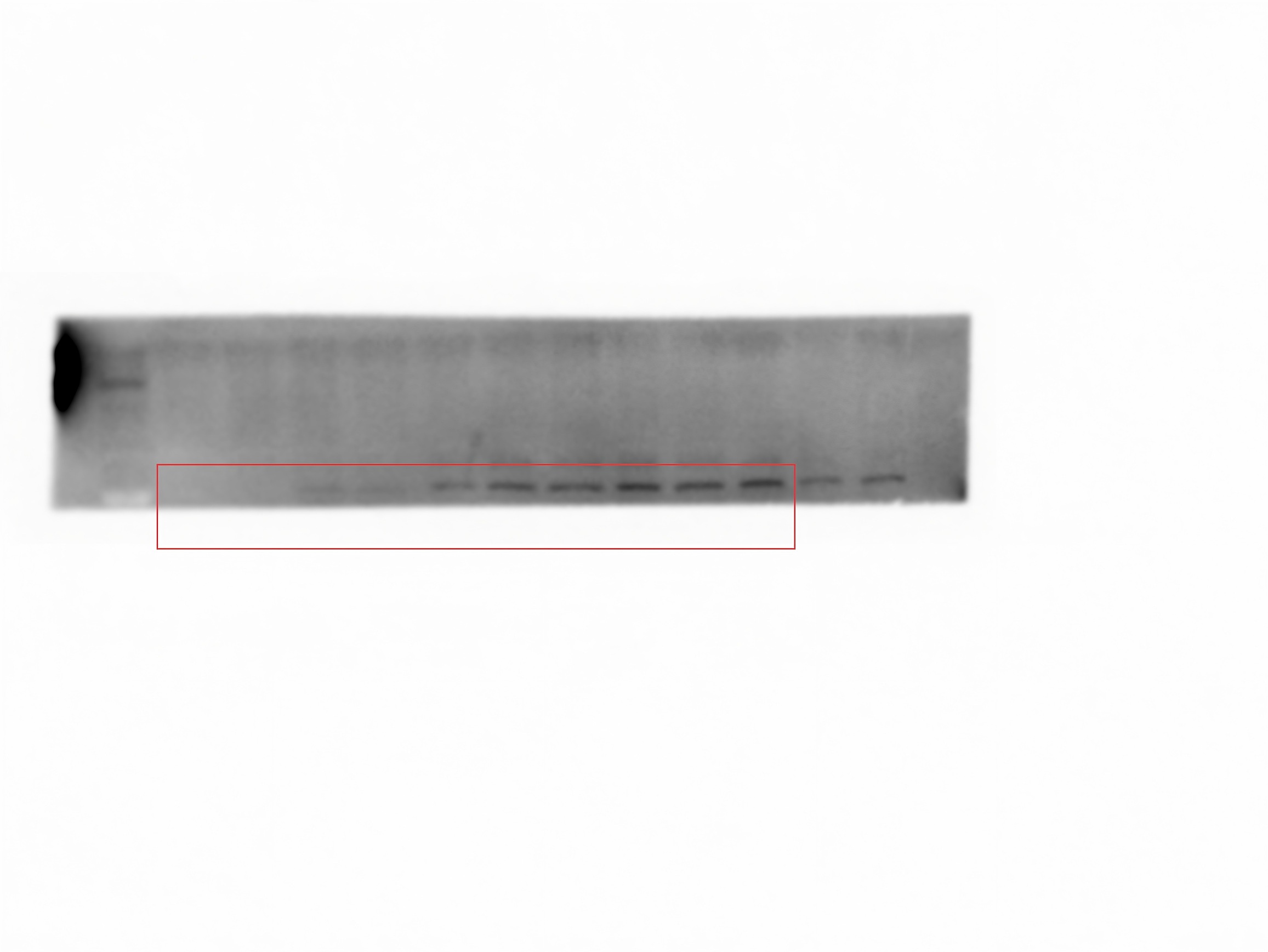

Supplement: Supplementary file 3 [file DataSheet1.zip › Fig2C EV71-3C edited showing band.jpg]

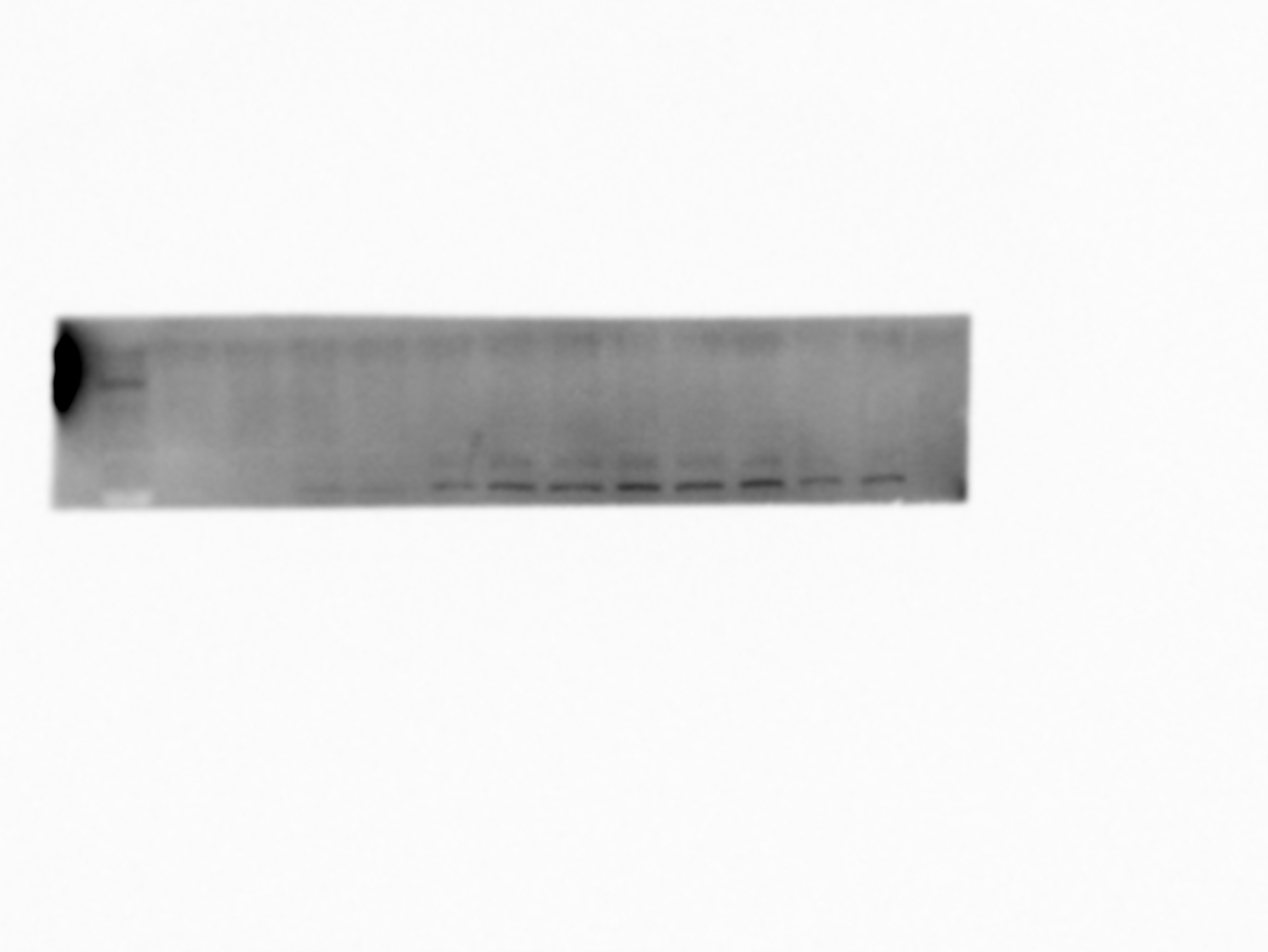

Supplement: Supplementary file 3 [file DataSheet1.zip › Fig2C EV71-3C.tif]

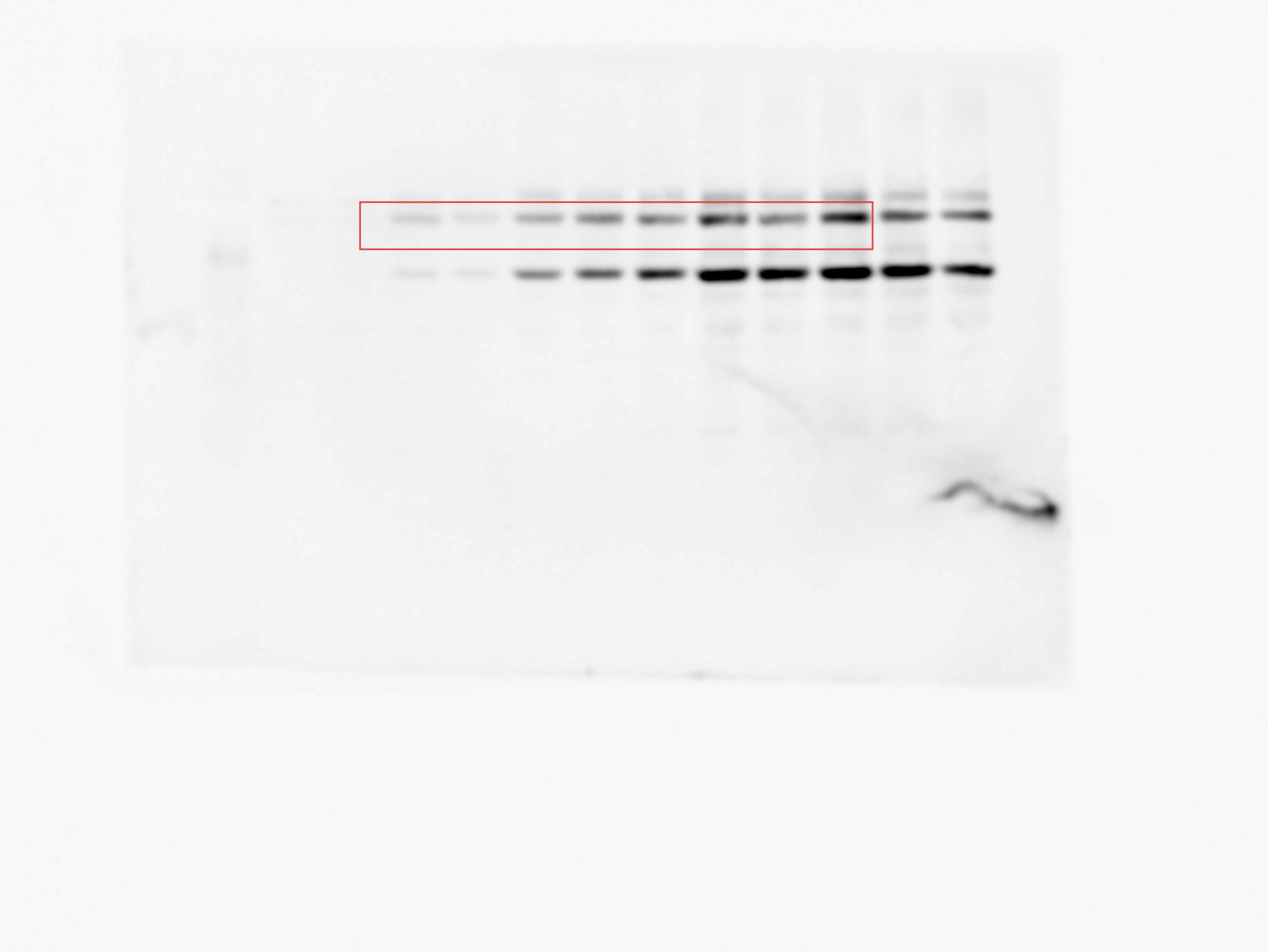

Supplement: Supplementary file 3 [file DataSheet1.zip › Fig2C EV71-3D edited showing band.jpg]

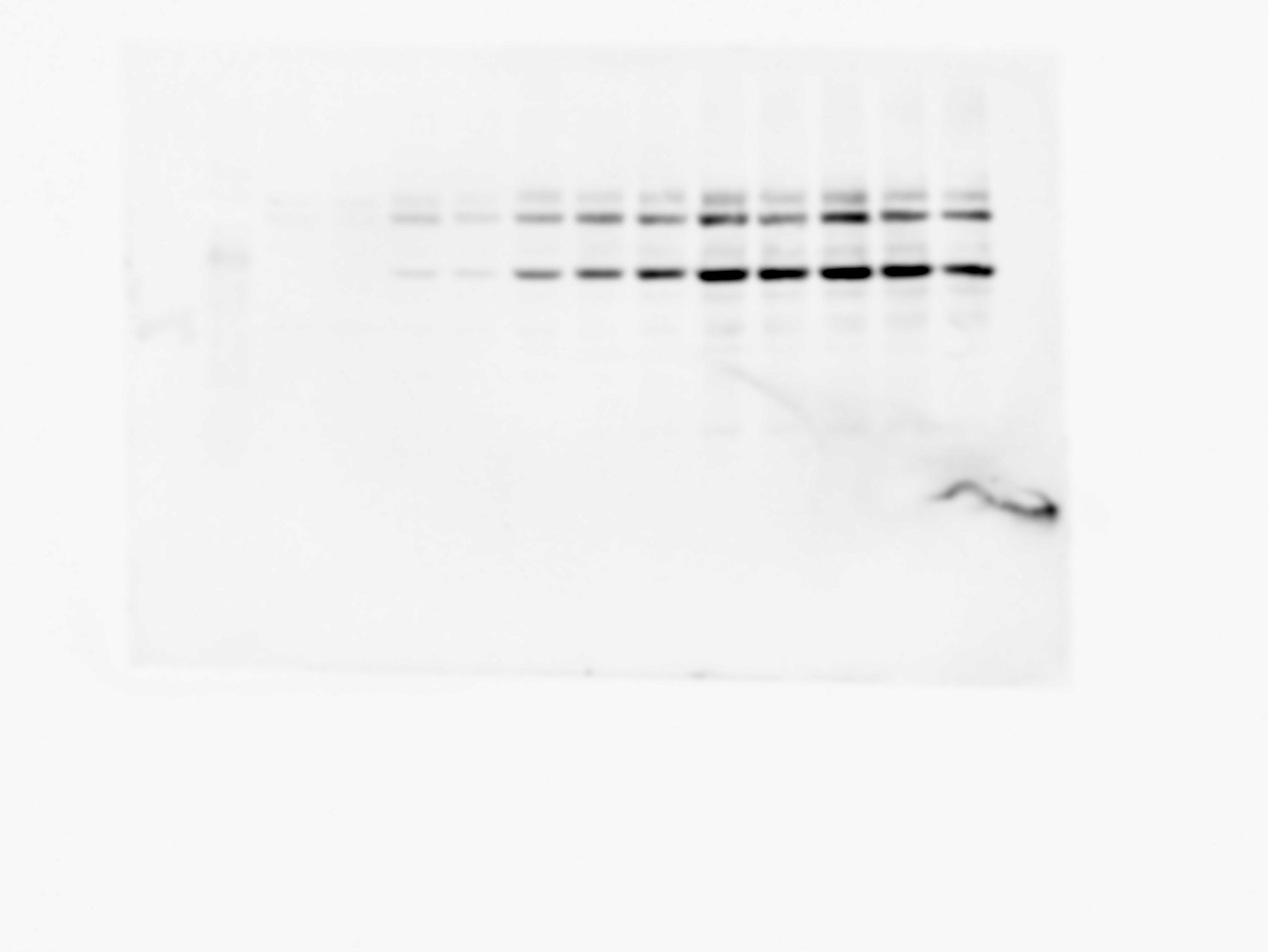

Supplement: Supplementary file 3 [file DataSheet1.zip › Fig2C EV71-3D.jpg]

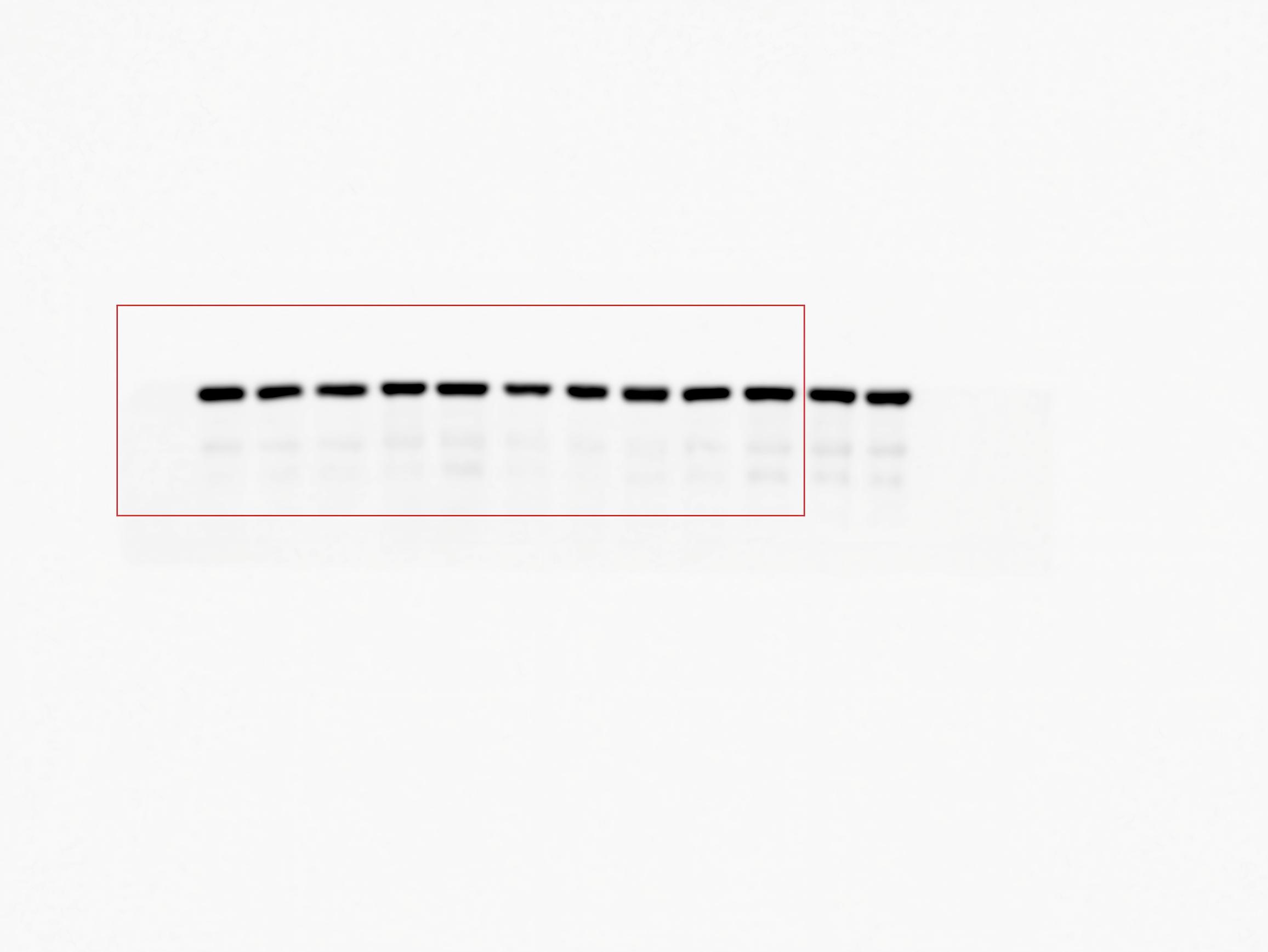

Supplement: Supplementary file 3 [file DataSheet1.zip › Fig2D Actin edited showing band.jpg]

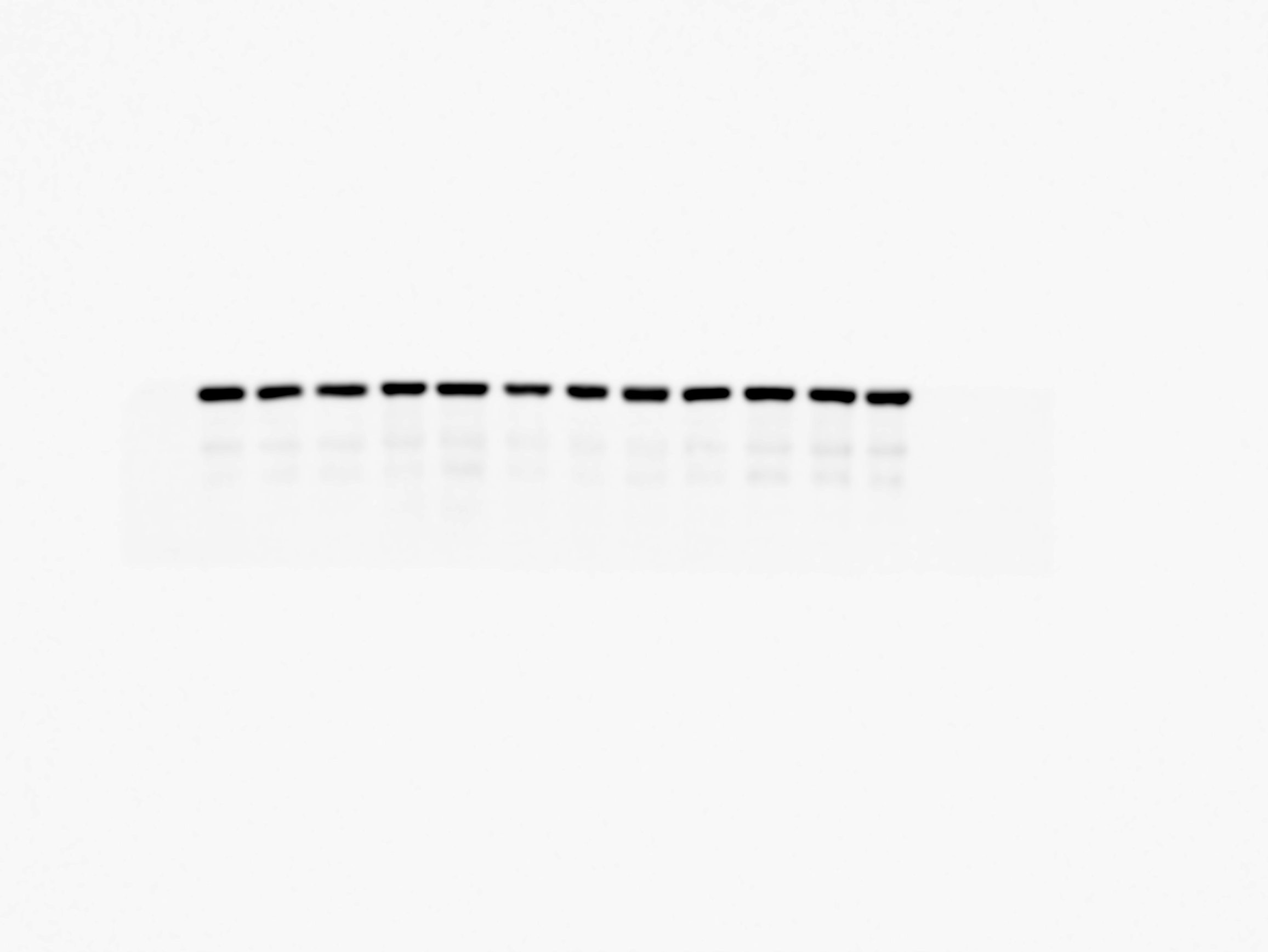

Supplement: Supplementary file 3 [file DataSheet1.zip › Fig2D Actin.jpg]

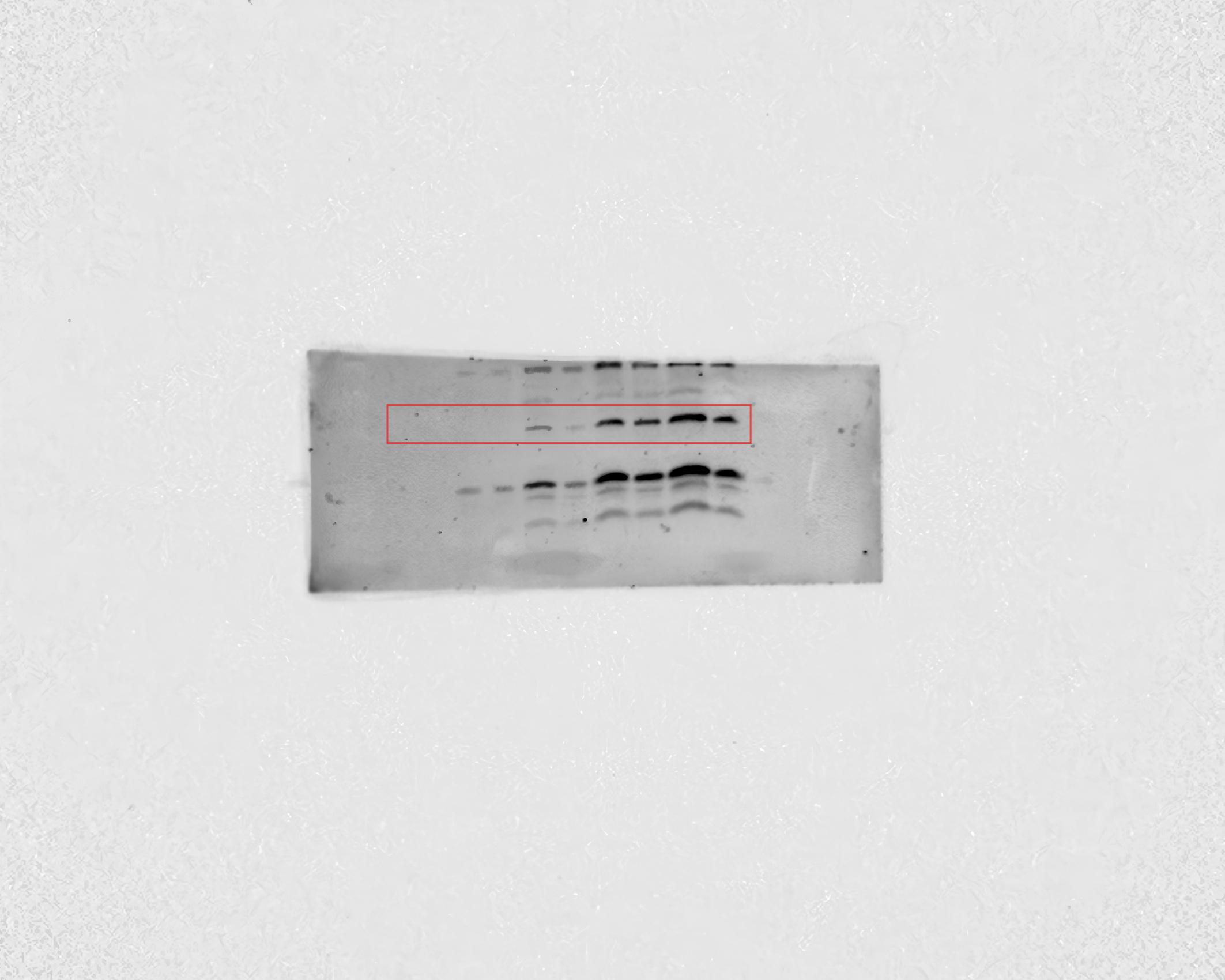

Supplement: Supplementary file 3 [file DataSheet1.zip › Fig2D EV-71 3C edited showing band.jpg]

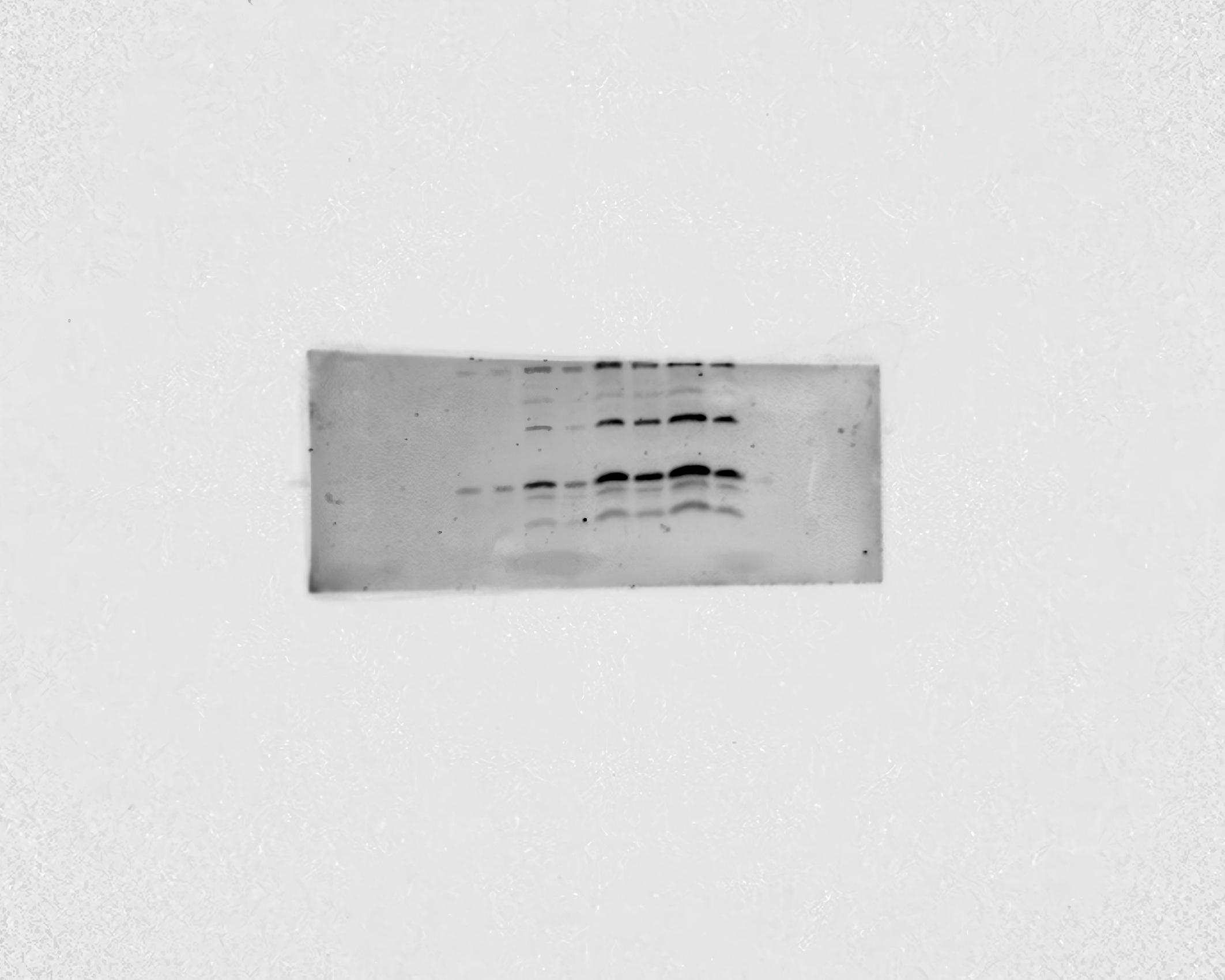

Supplement: Supplementary file 3 [file DataSheet1.zip › Fig2D EV-71 3C.jpg]

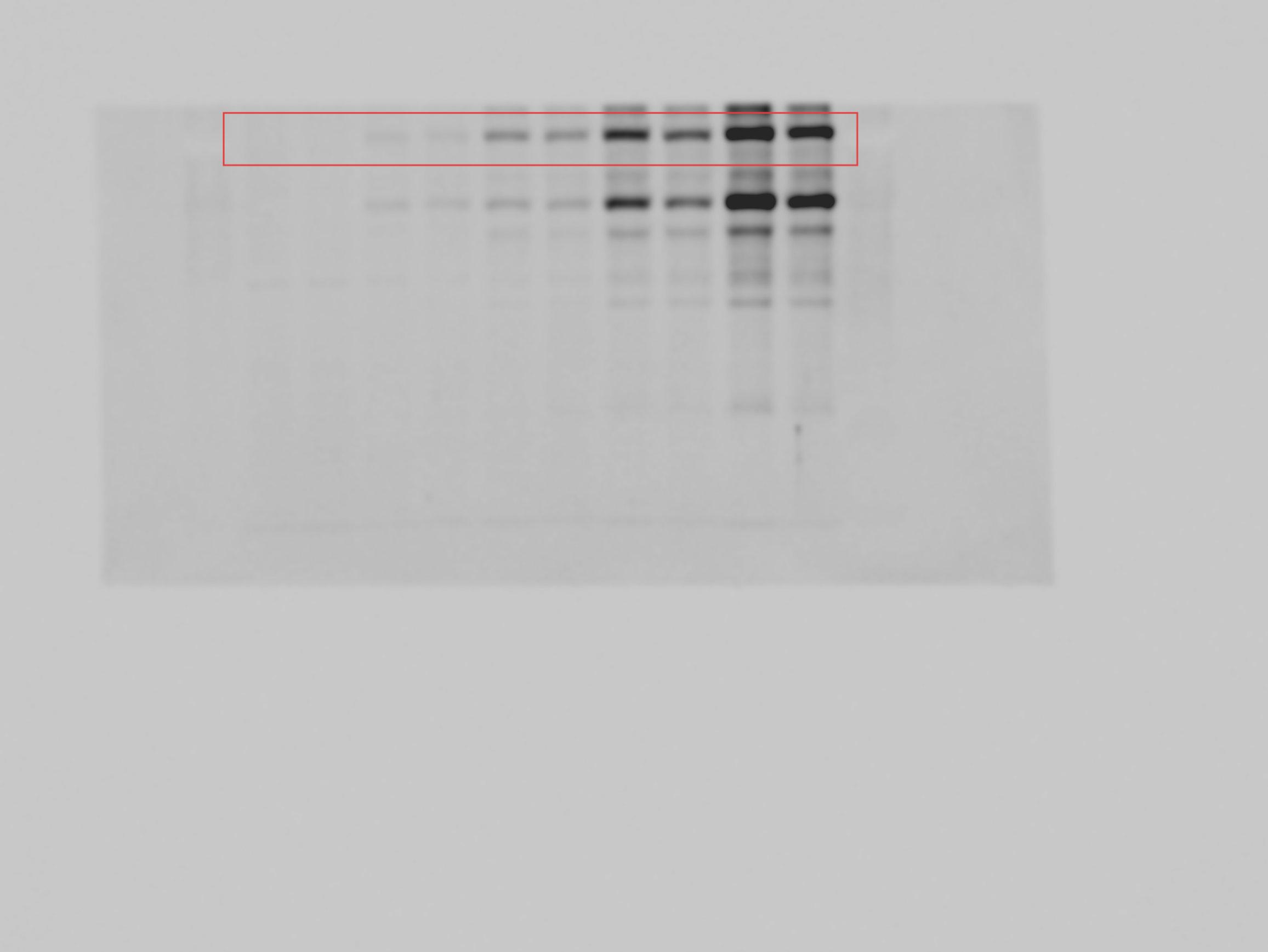

Supplement: Supplementary file 3 [file DataSheet1.zip › Fig2D EV-71 3D edited showing band.jpg]

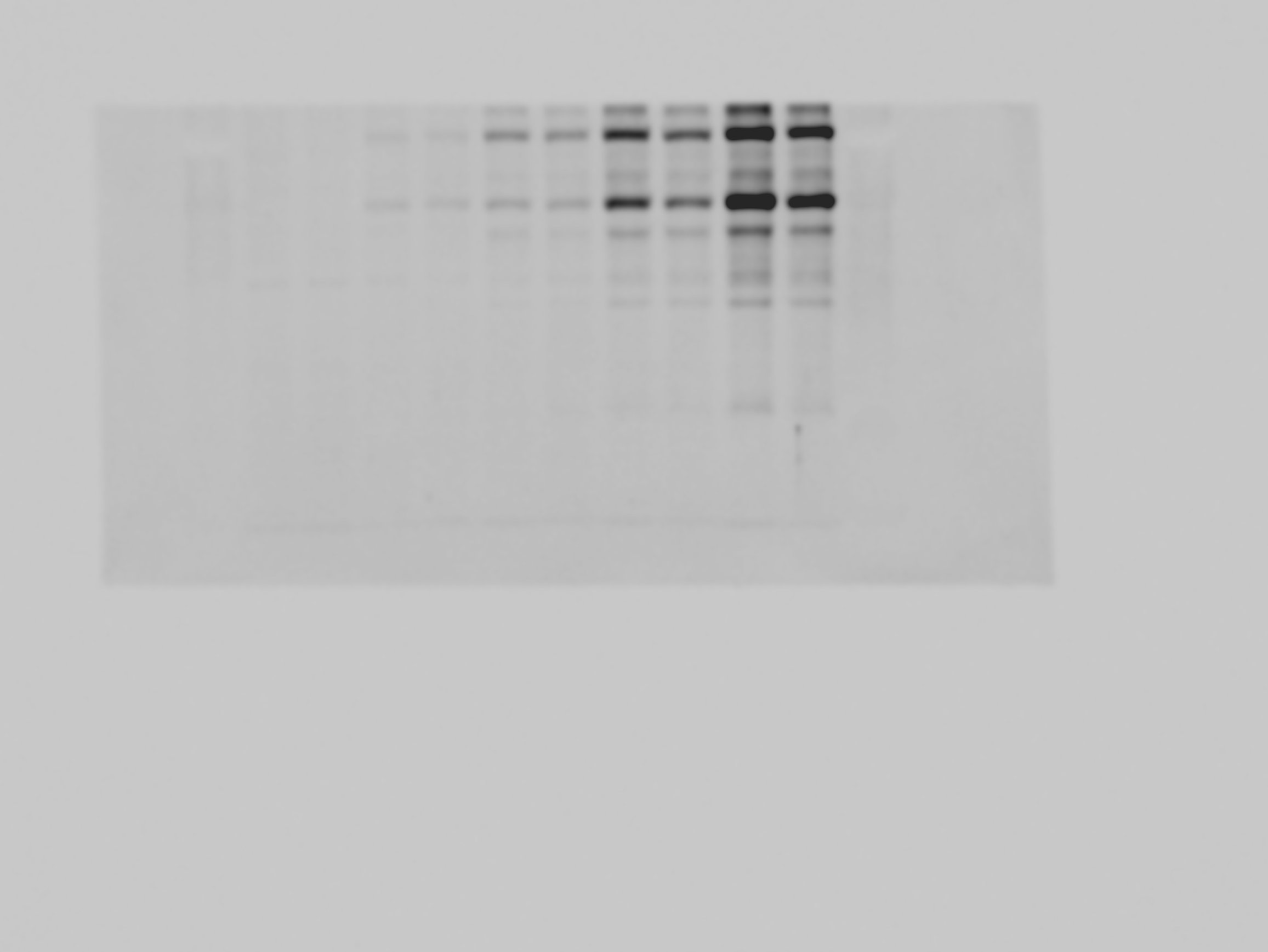

Supplement: Supplementary file 3 [file DataSheet1.zip › Fig2D EV-71 3D.tif]

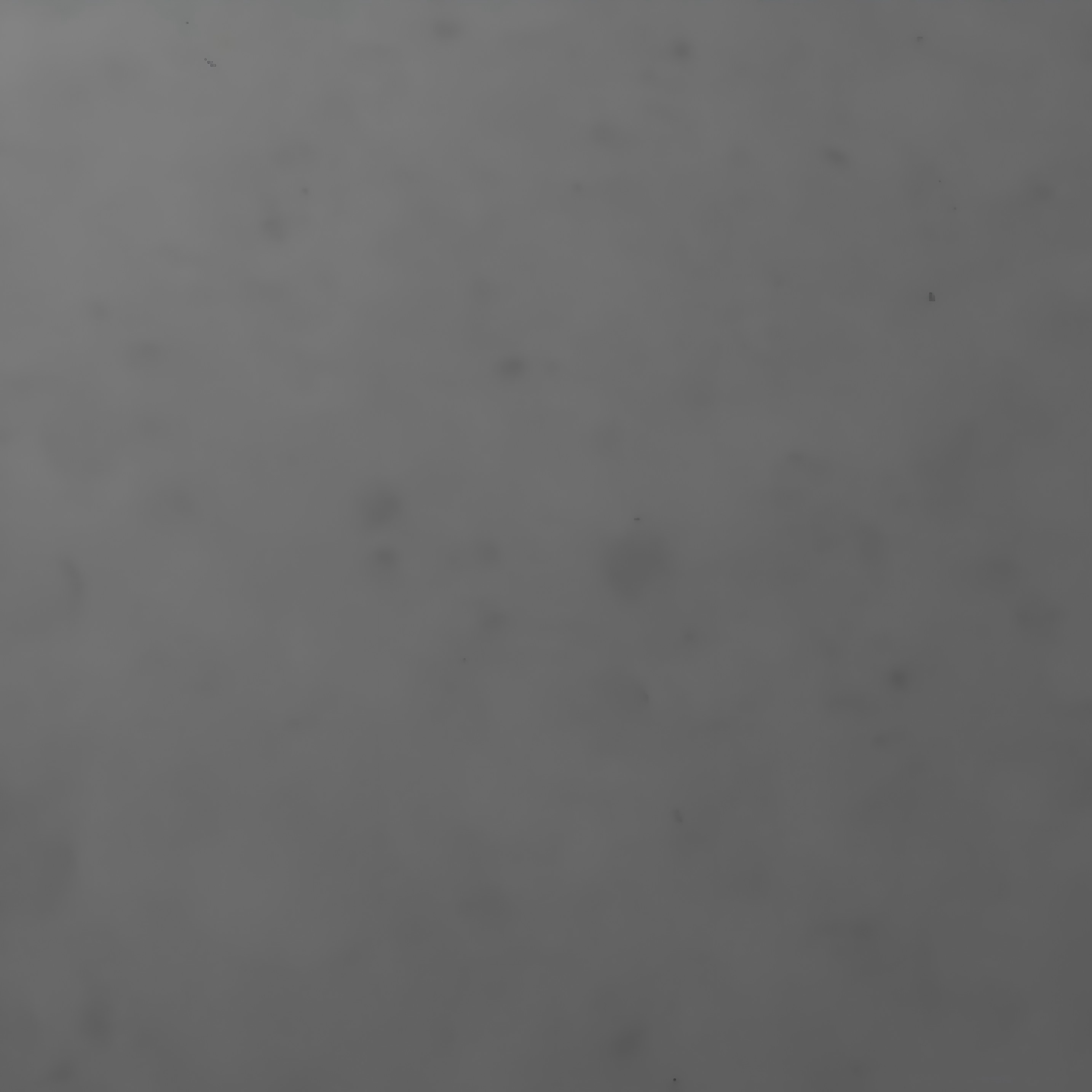

Supplement: Supplementary file 3 [file DataSheet1.zip › Fig2E TRIM28 OE1.tif]

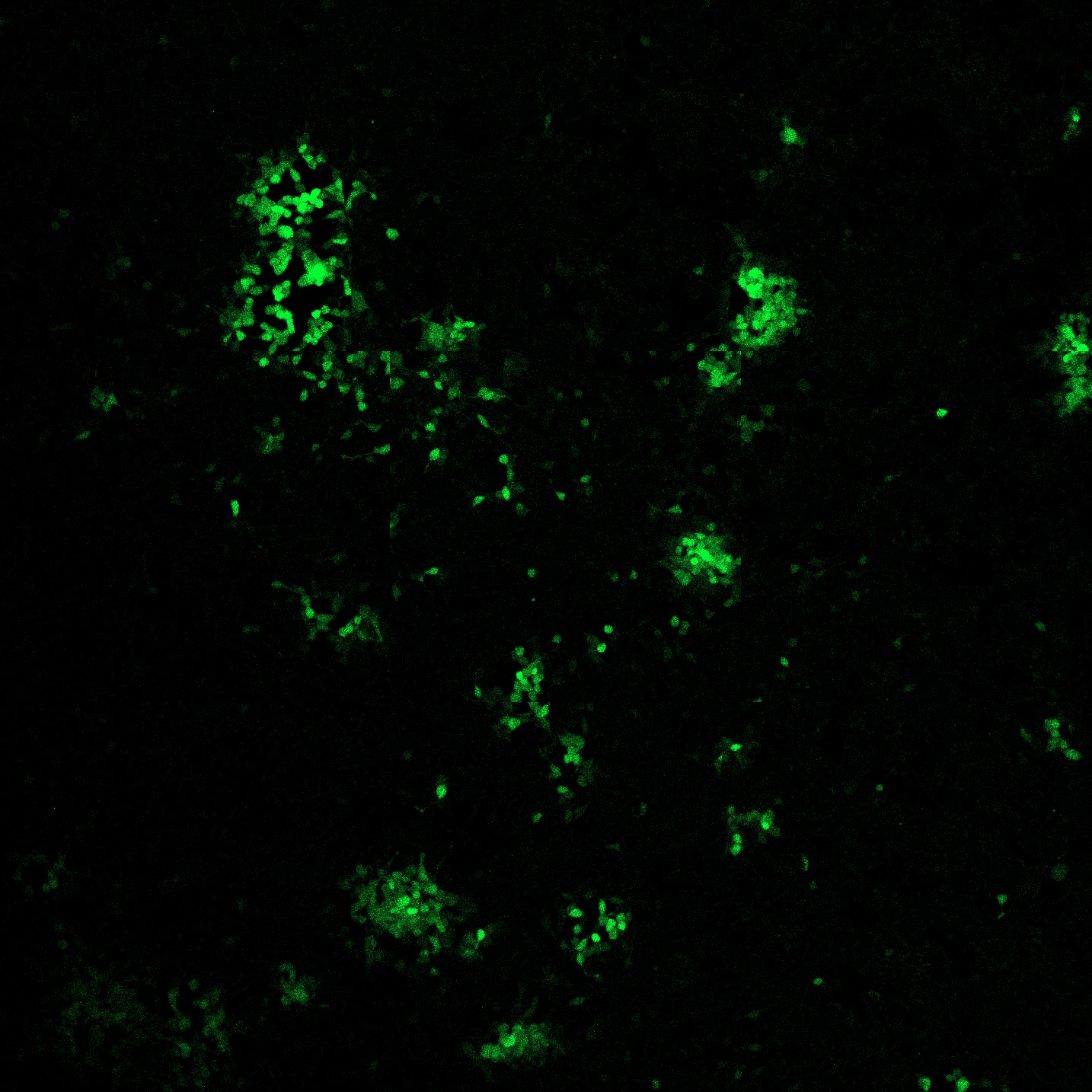

Supplement: Supplementary file 3 [file DataSheet1.zip › Fig2E TRIM28 OE2.tif]

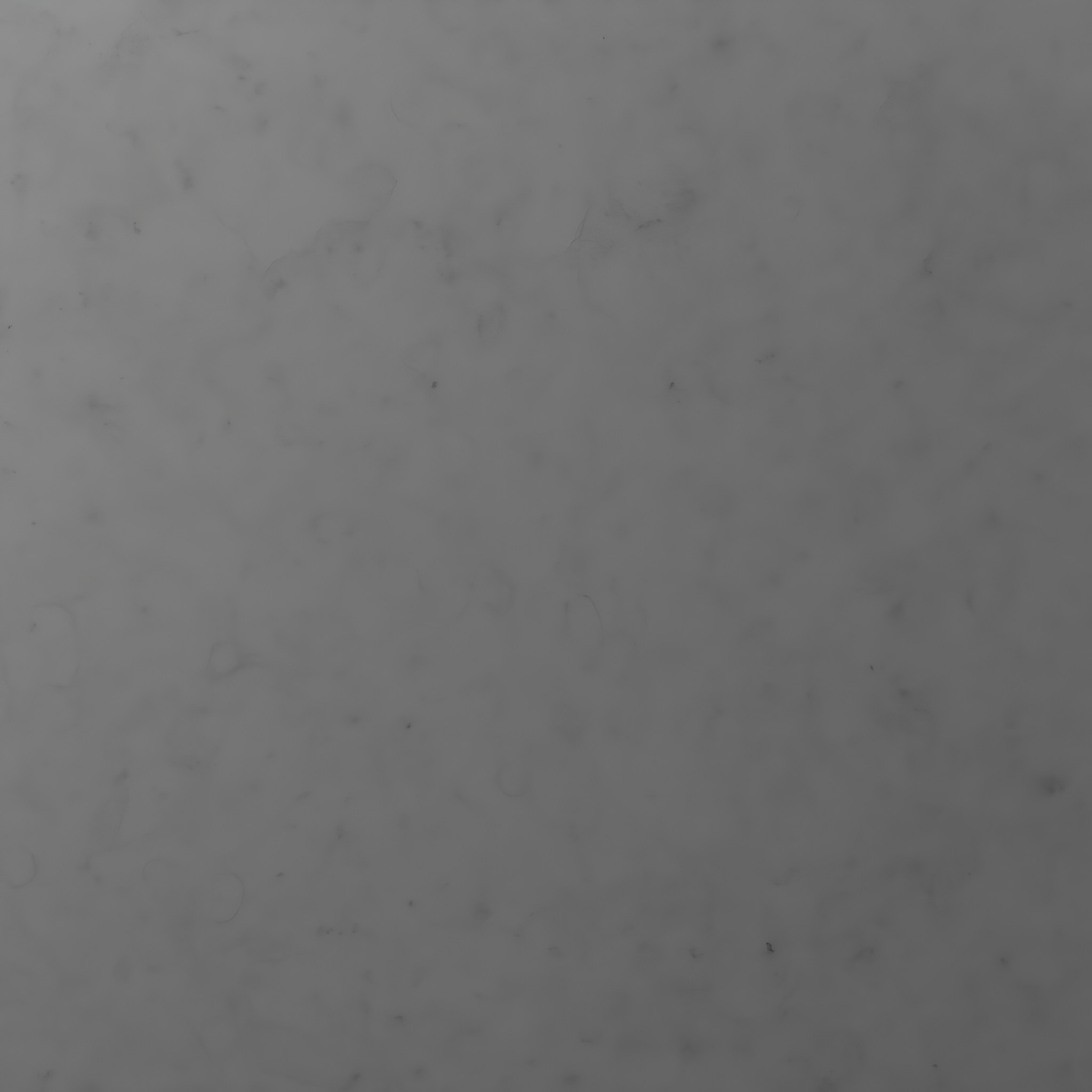

Supplement: Supplementary file 3 [file DataSheet1.zip › Fig2E Vec1.tif]

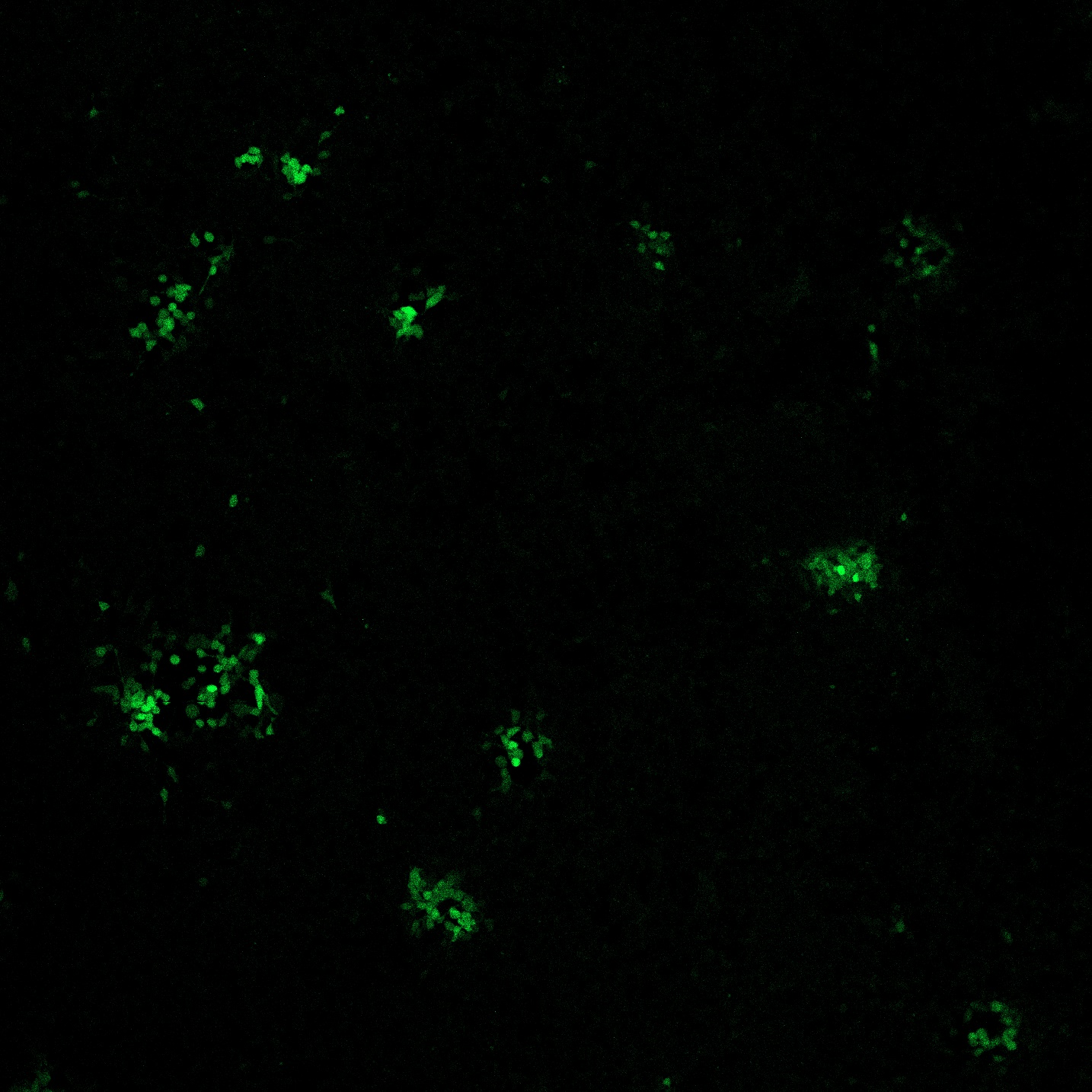

Supplement: Supplementary file 3 [file DataSheet1.zip › Fig2E Vec2.tif]

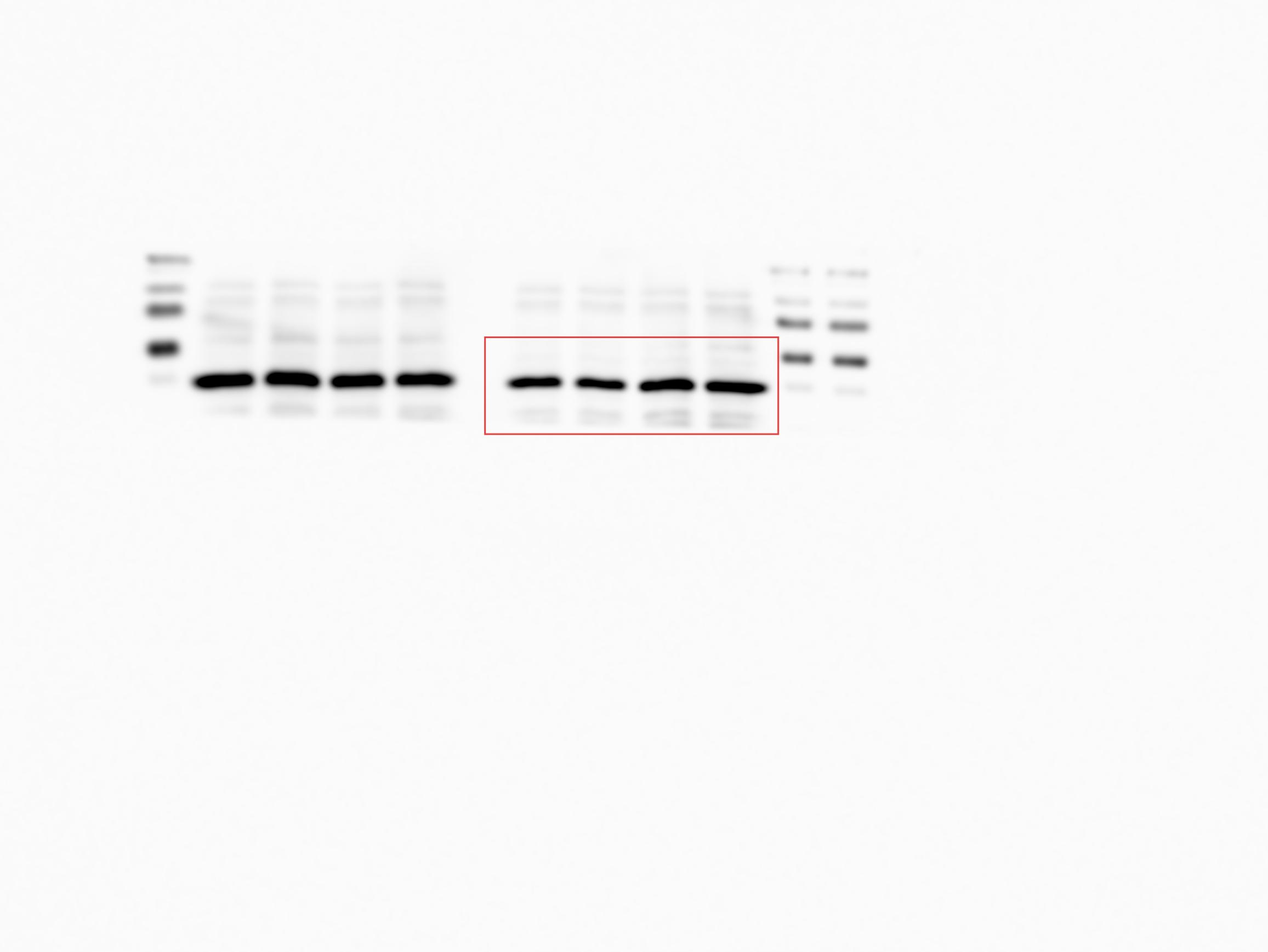

Supplement: Supplementary file 3 [file DataSheet1.zip › Fig2F Actin down edited showing band.jpg]

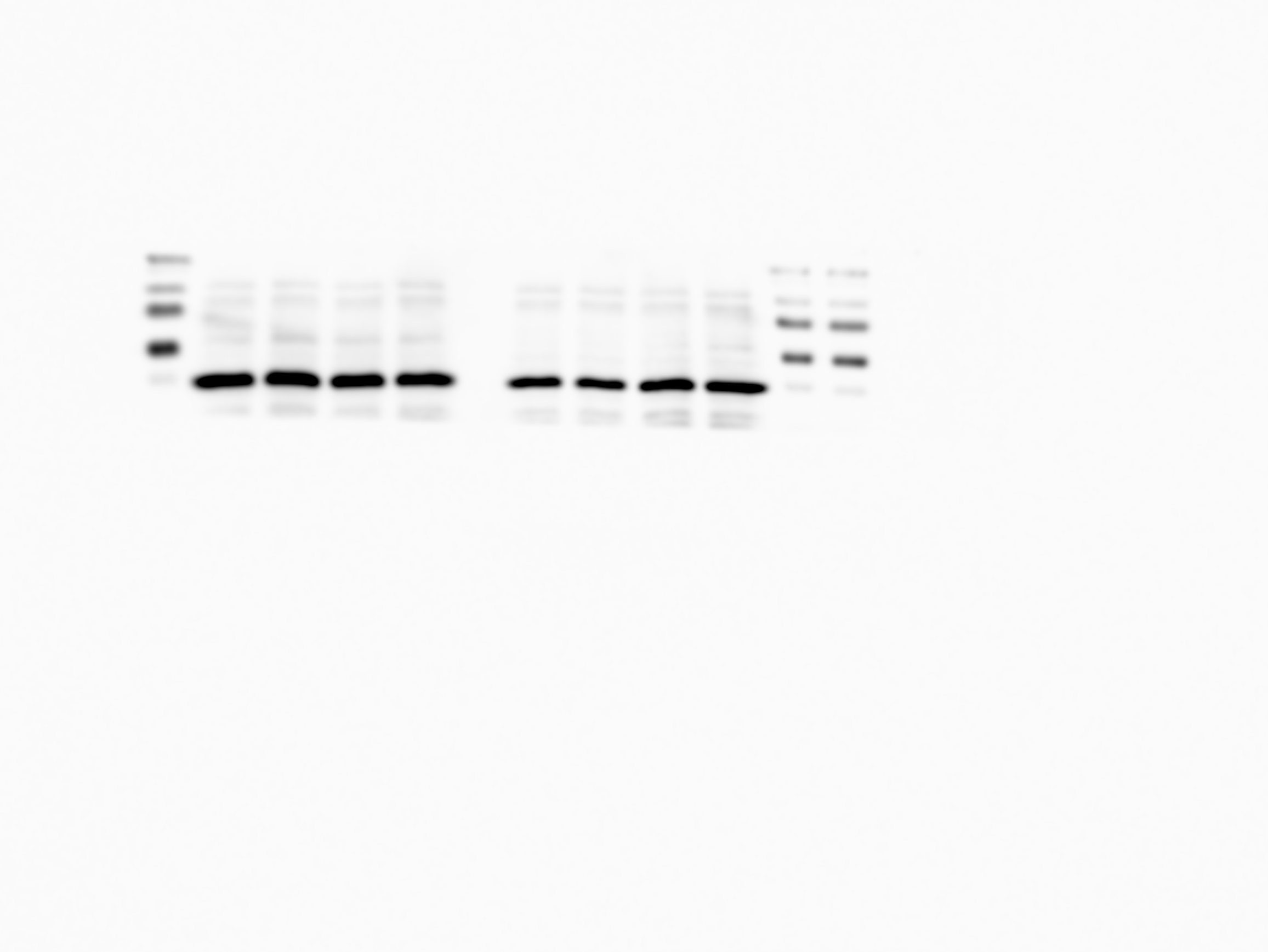

Supplement: Supplementary file 3 [file DataSheet1.zip › Fig2F Actin down.tif]

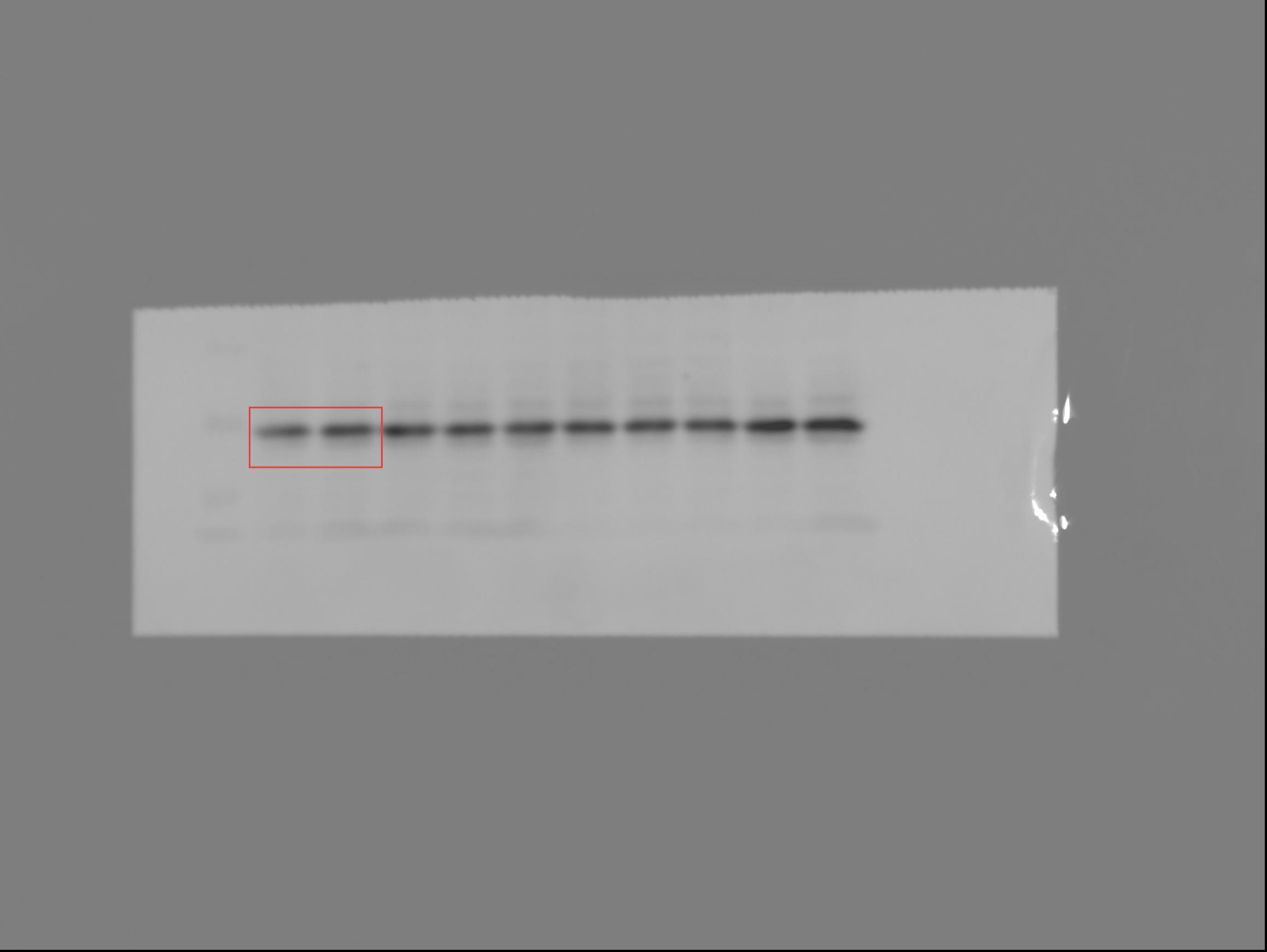

Supplement: Supplementary file 3 [file DataSheet1.zip › Fig2F Actin up edited showing band.jpg]

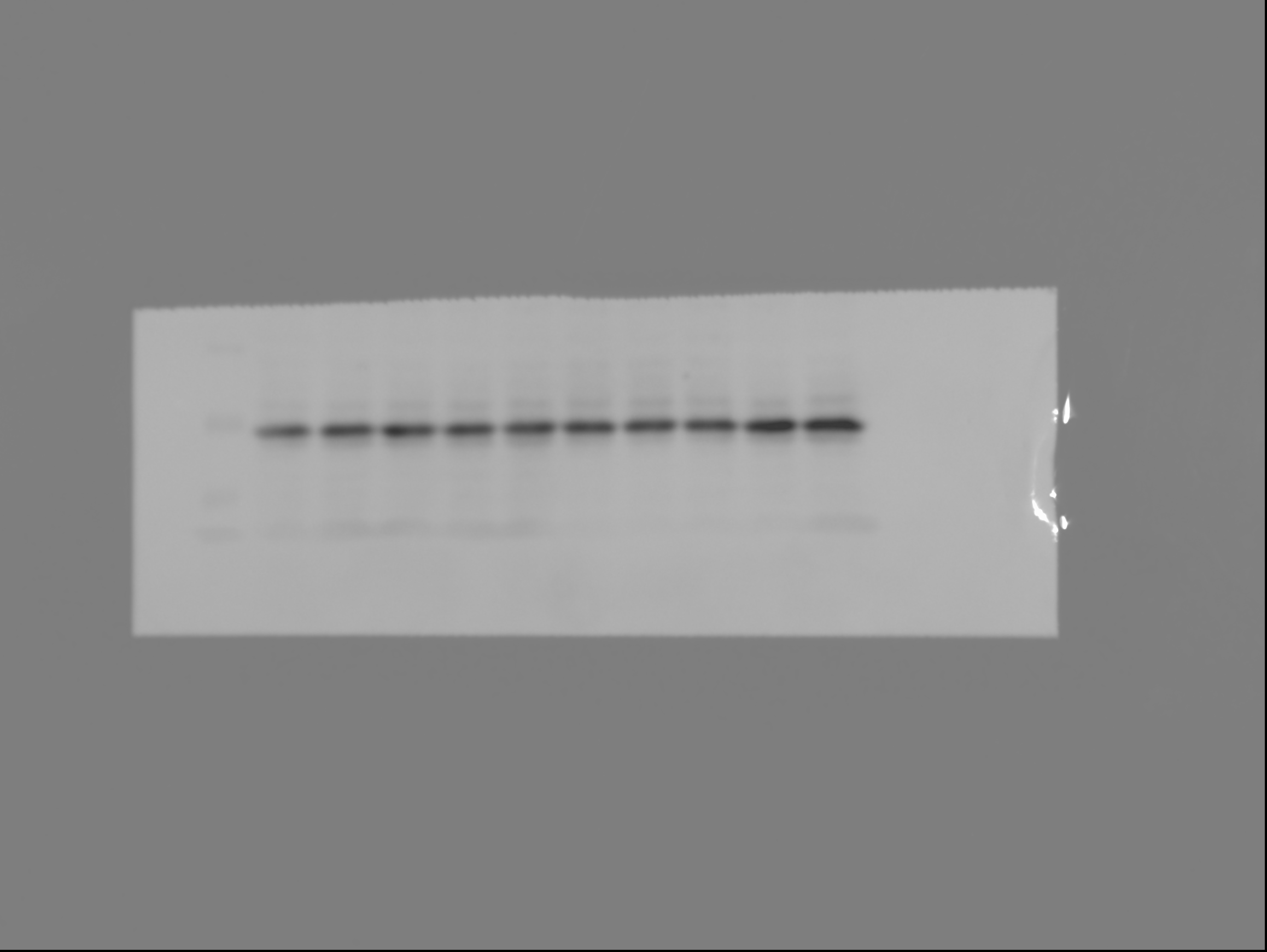

Supplement: Supplementary file 3 [file DataSheet1.zip › Fig2F Actin up.tif]

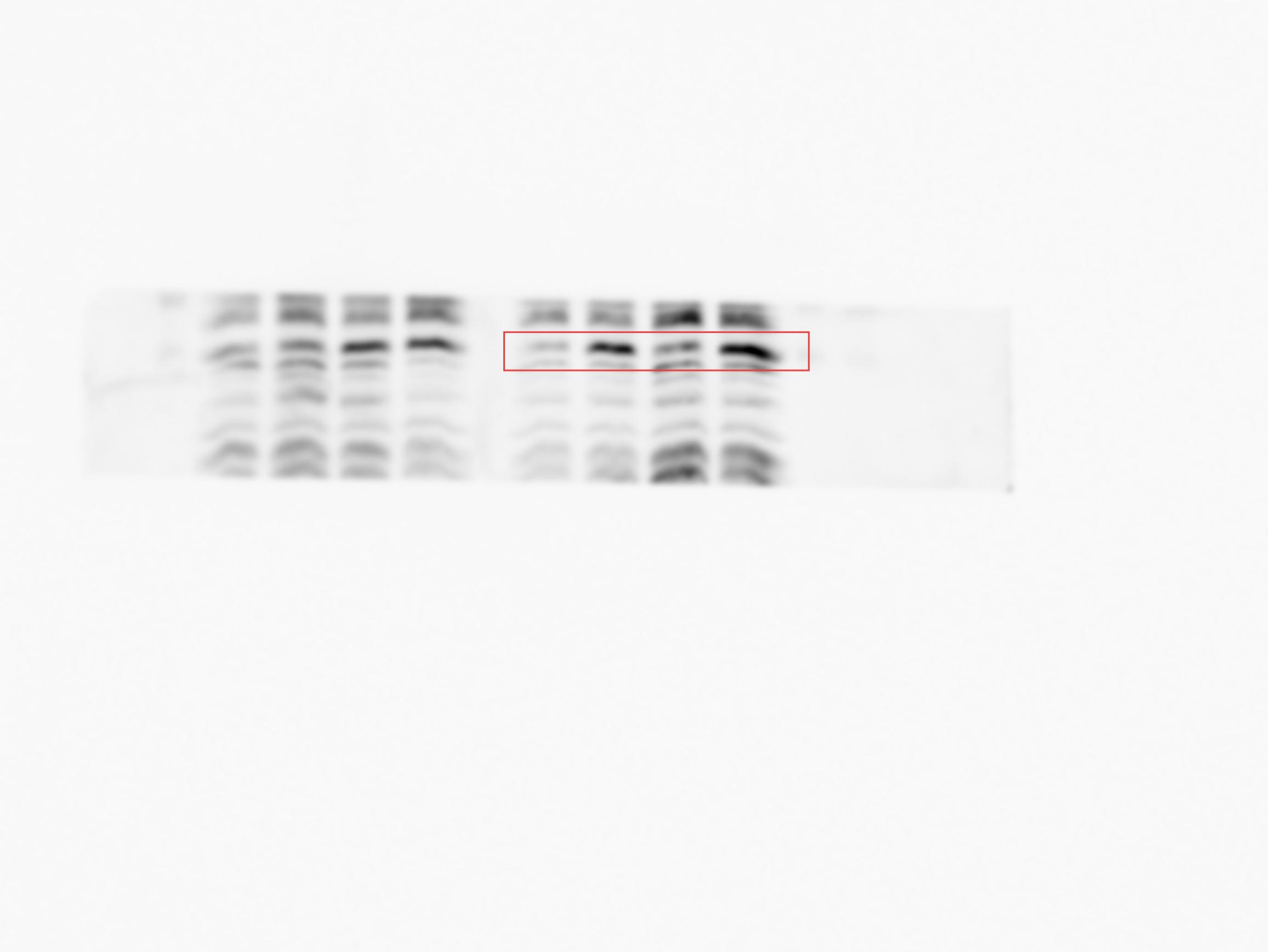

Supplement: Supplementary file 3 [file DataSheet1.zip › Fig2F GFP edited showing band.jpg]

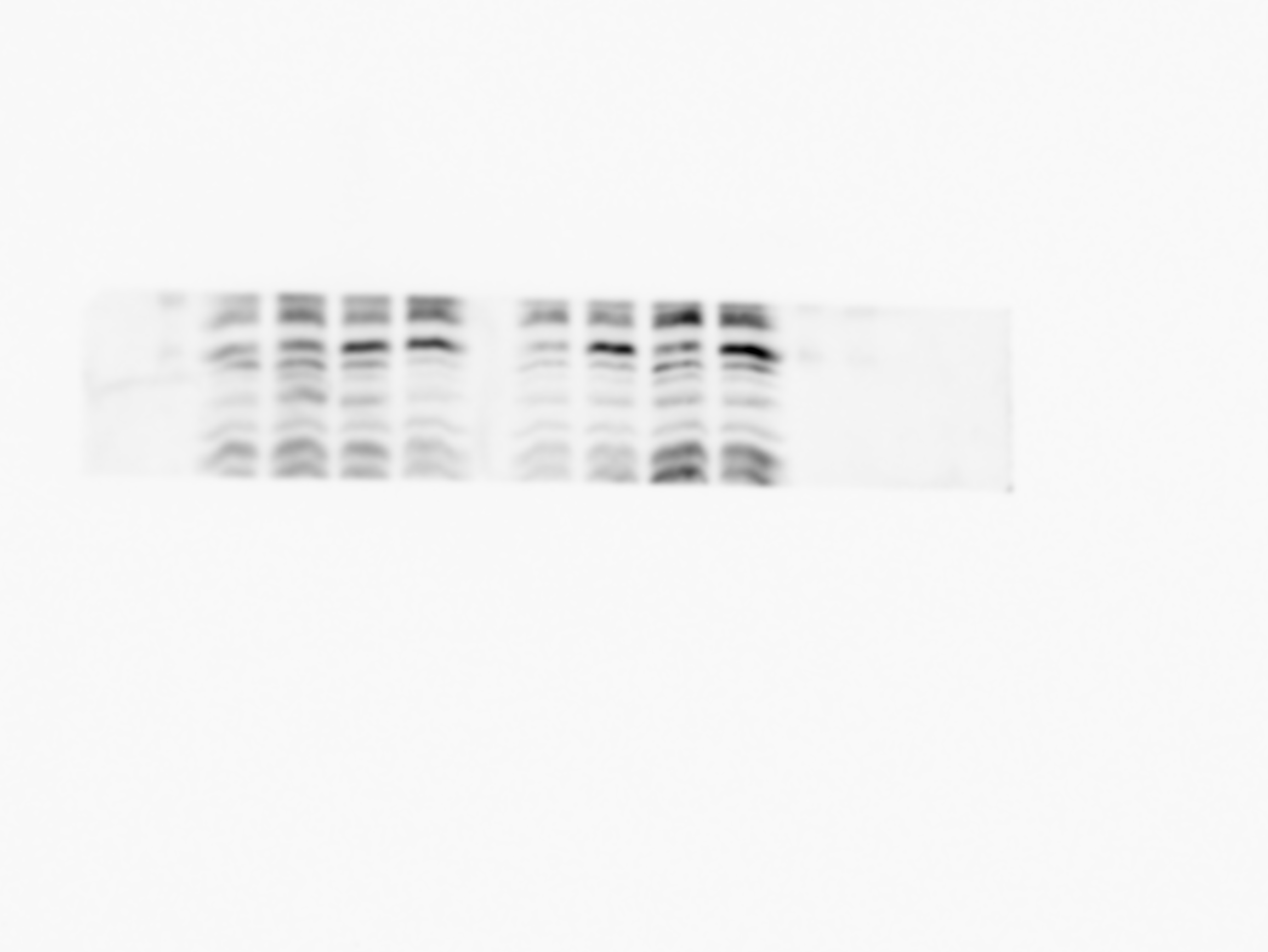

Supplement: Supplementary file 3 [file DataSheet1.zip › Fig2F GFP.tif]

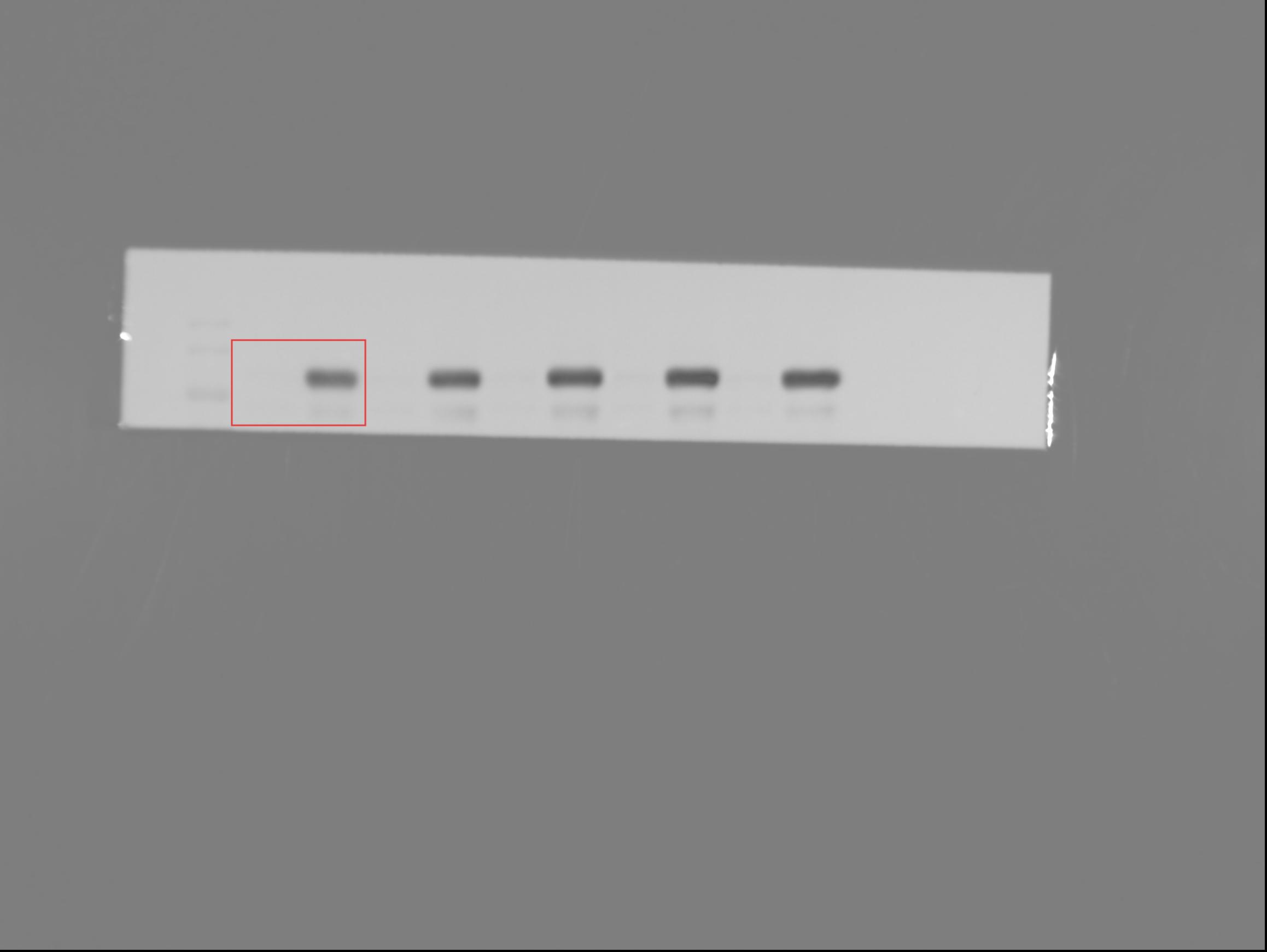

Supplement: Supplementary file 3 [file DataSheet1.zip › Fig2F Myc edited showing band.jpg]

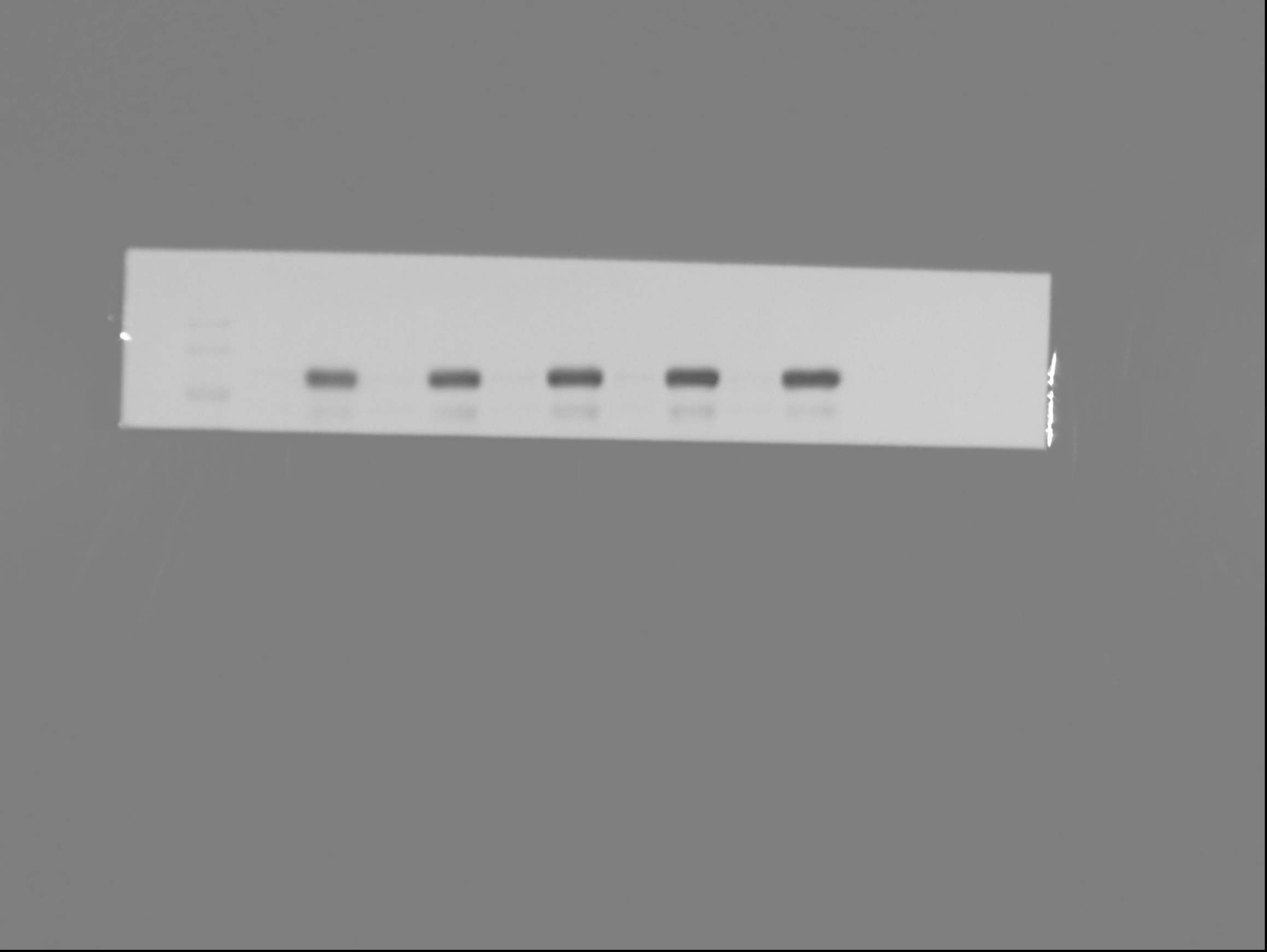

Supplement: Supplementary file 3 [file DataSheet1.zip › Fig2F Myc.tif]

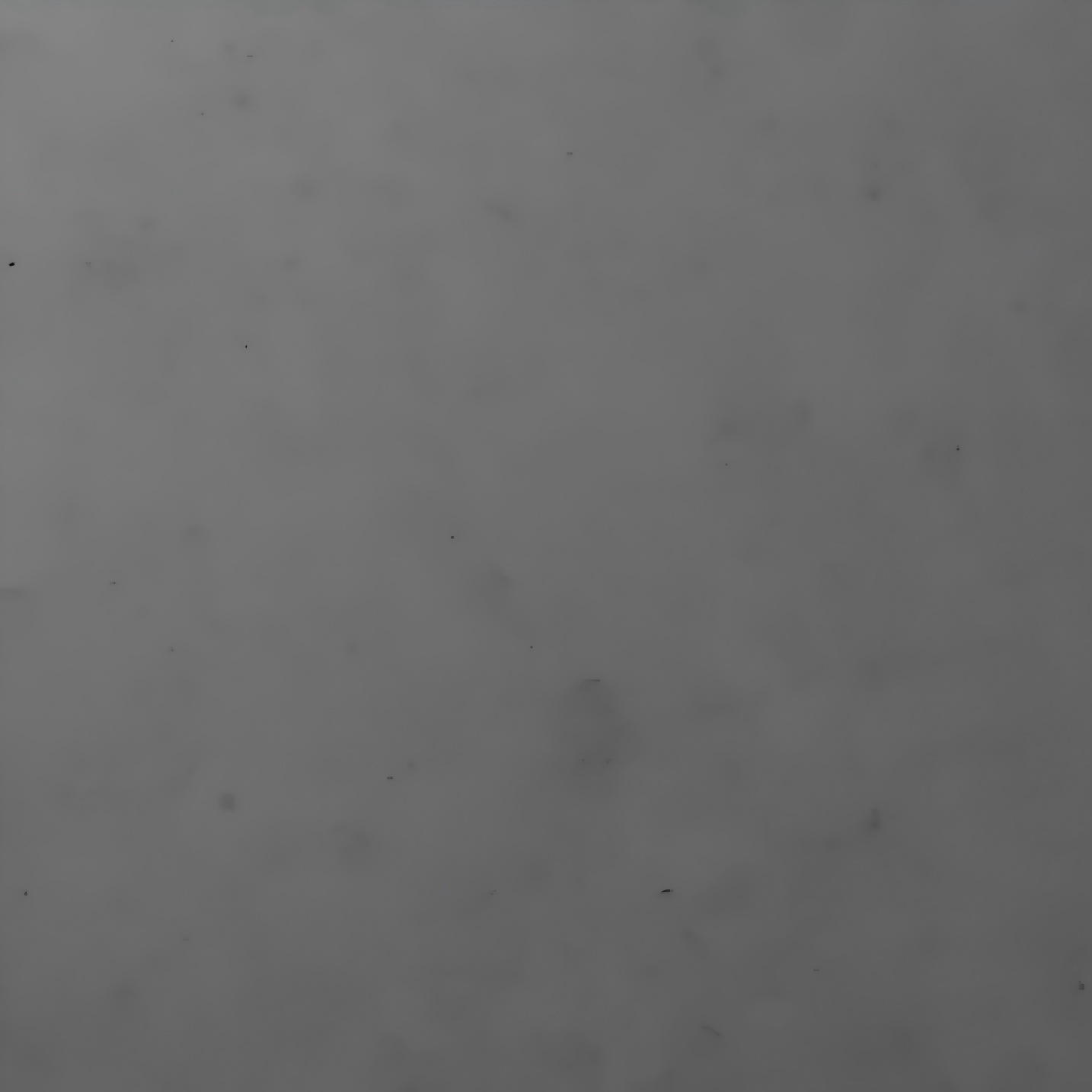

Supplement: Supplementary file 3 [file DataSheet1.zip › Fig2H NC1.tif]

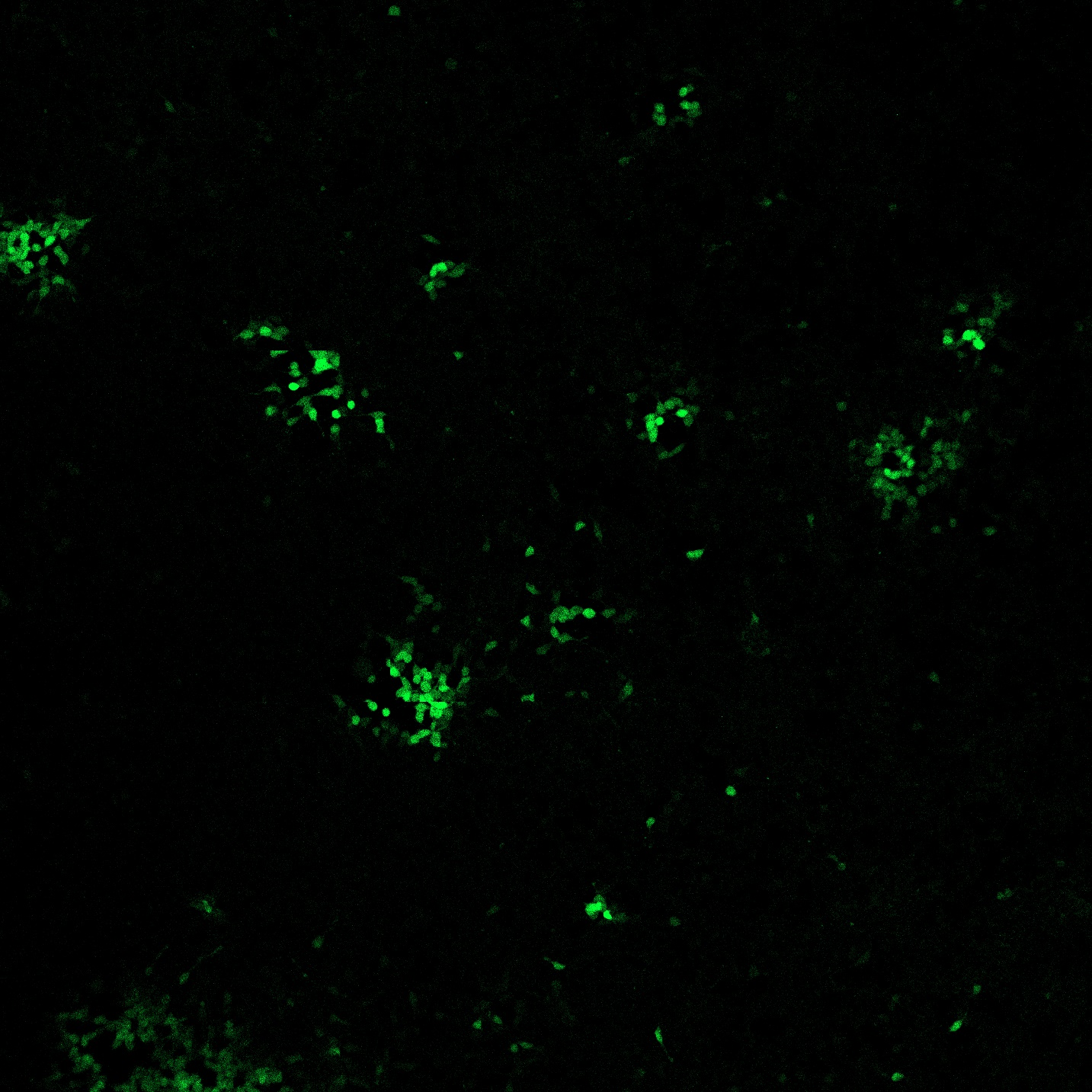

Supplement: Supplementary file 3 [file DataSheet1.zip › Fig2H NC2.tif]

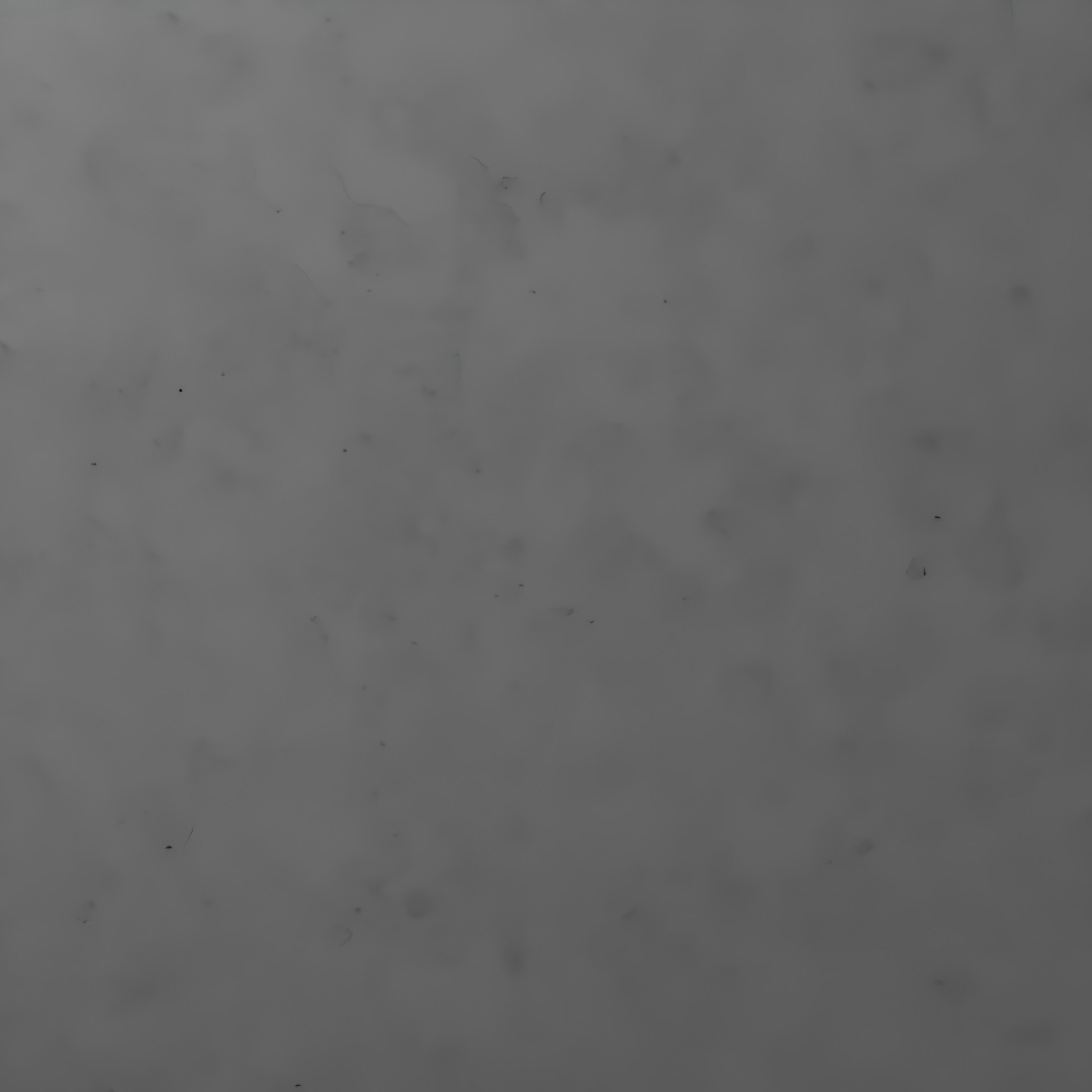

Supplement: Supplementary file 3 [file DataSheet1.zip › Fig2H TRIM28 KD1.tif]

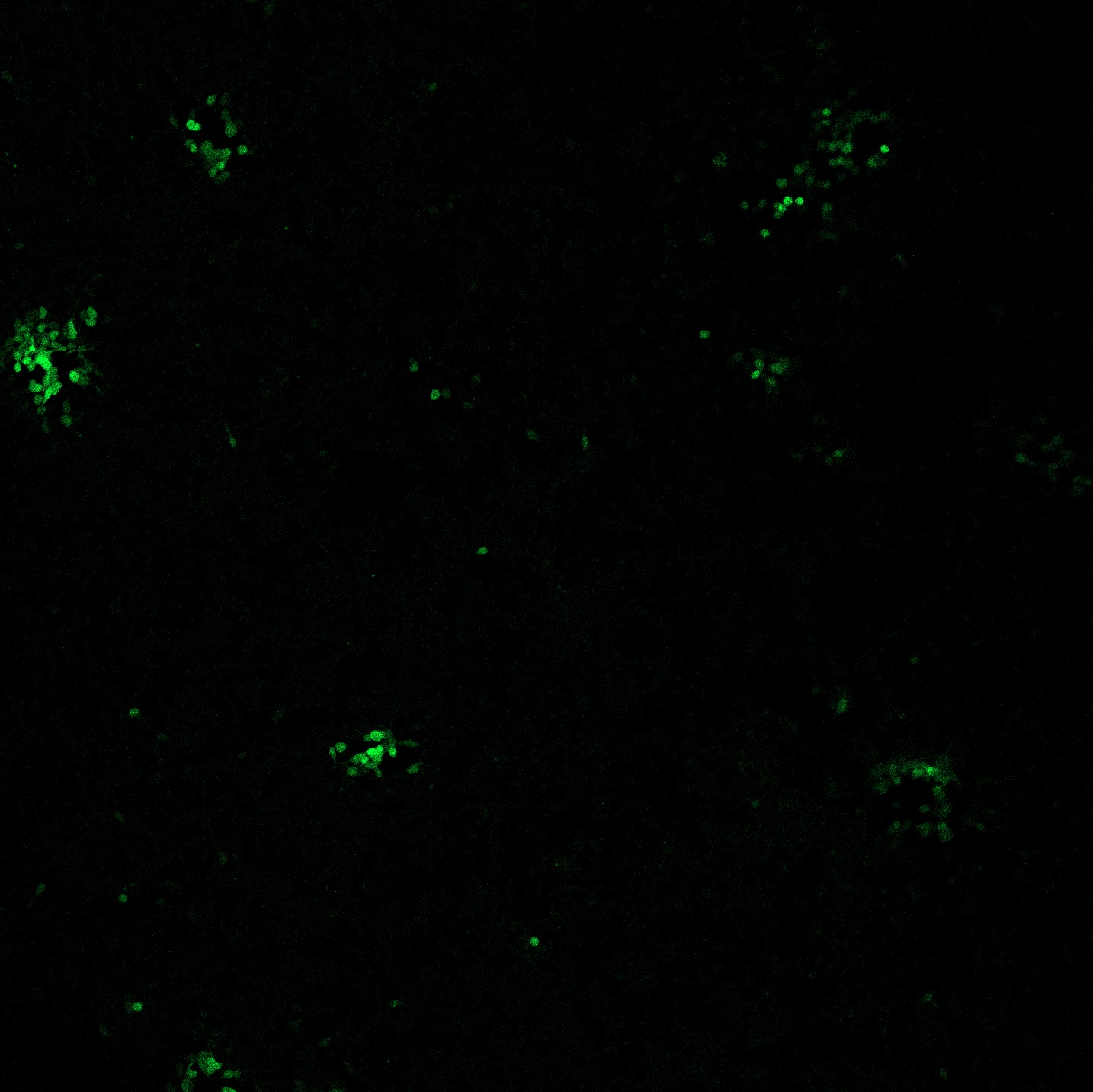

Supplement: Supplementary file 3 [file DataSheet1.zip › Fig2H TRIM28 KD2.tif]

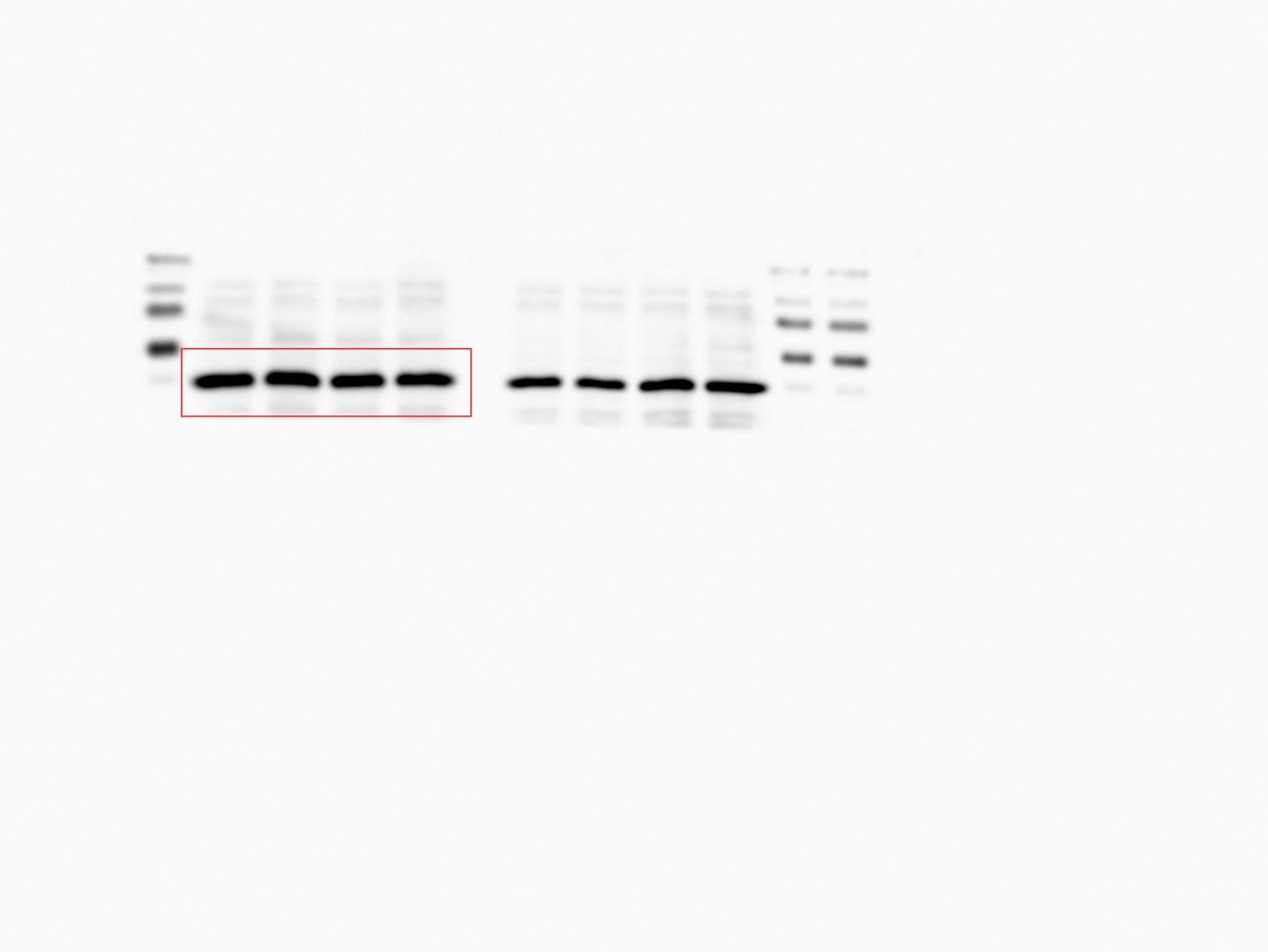

Supplement: Supplementary file 3 [file DataSheet1.zip › Fig2I Actin down edited showing band.jpg]

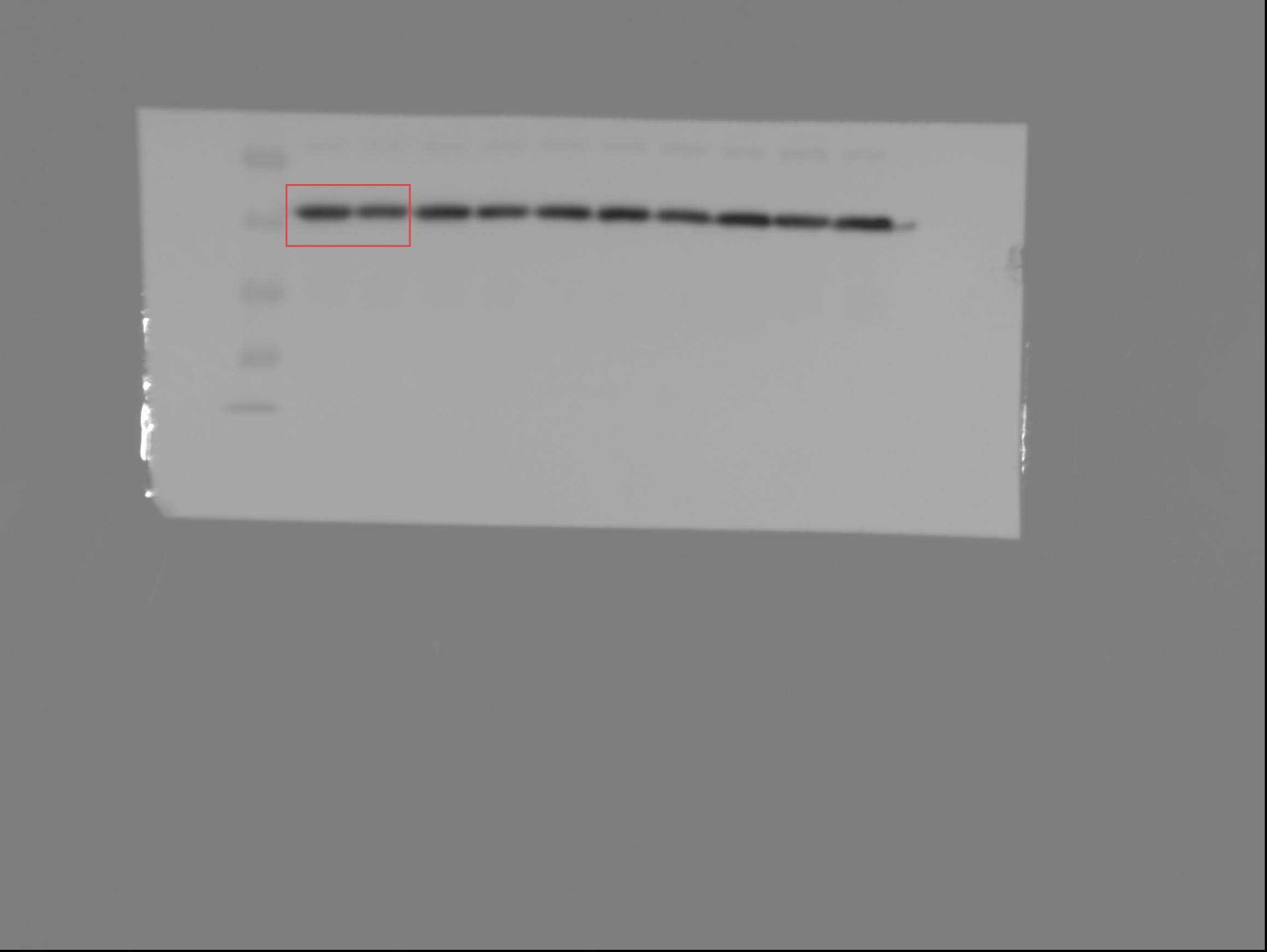

Supplement: Supplementary file 3 [file DataSheet1.zip › Fig2I Actin up edited showing band.jpg]

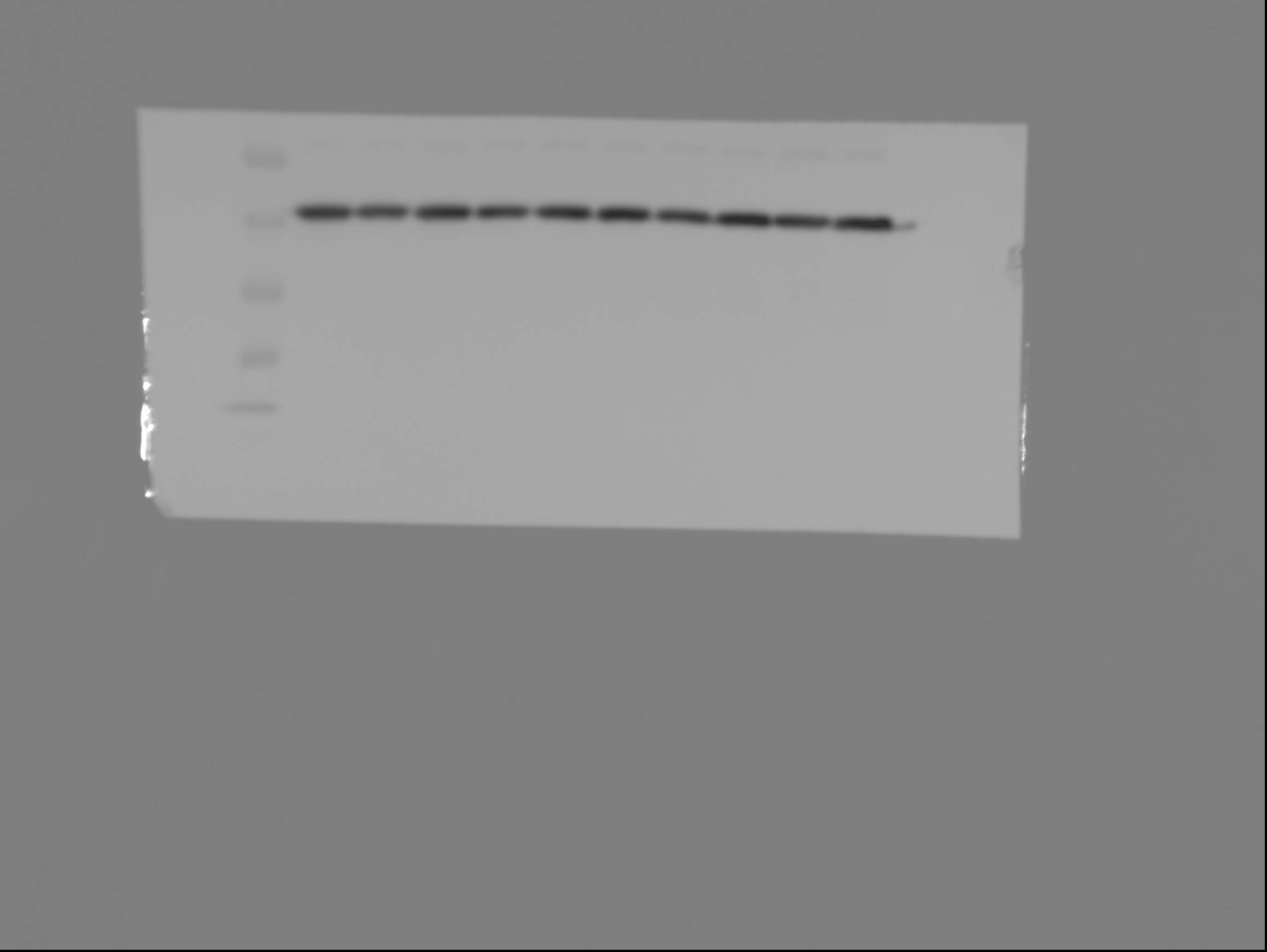

Supplement: Supplementary file 3 [file DataSheet1.zip › Fig2I Actin up.tif]

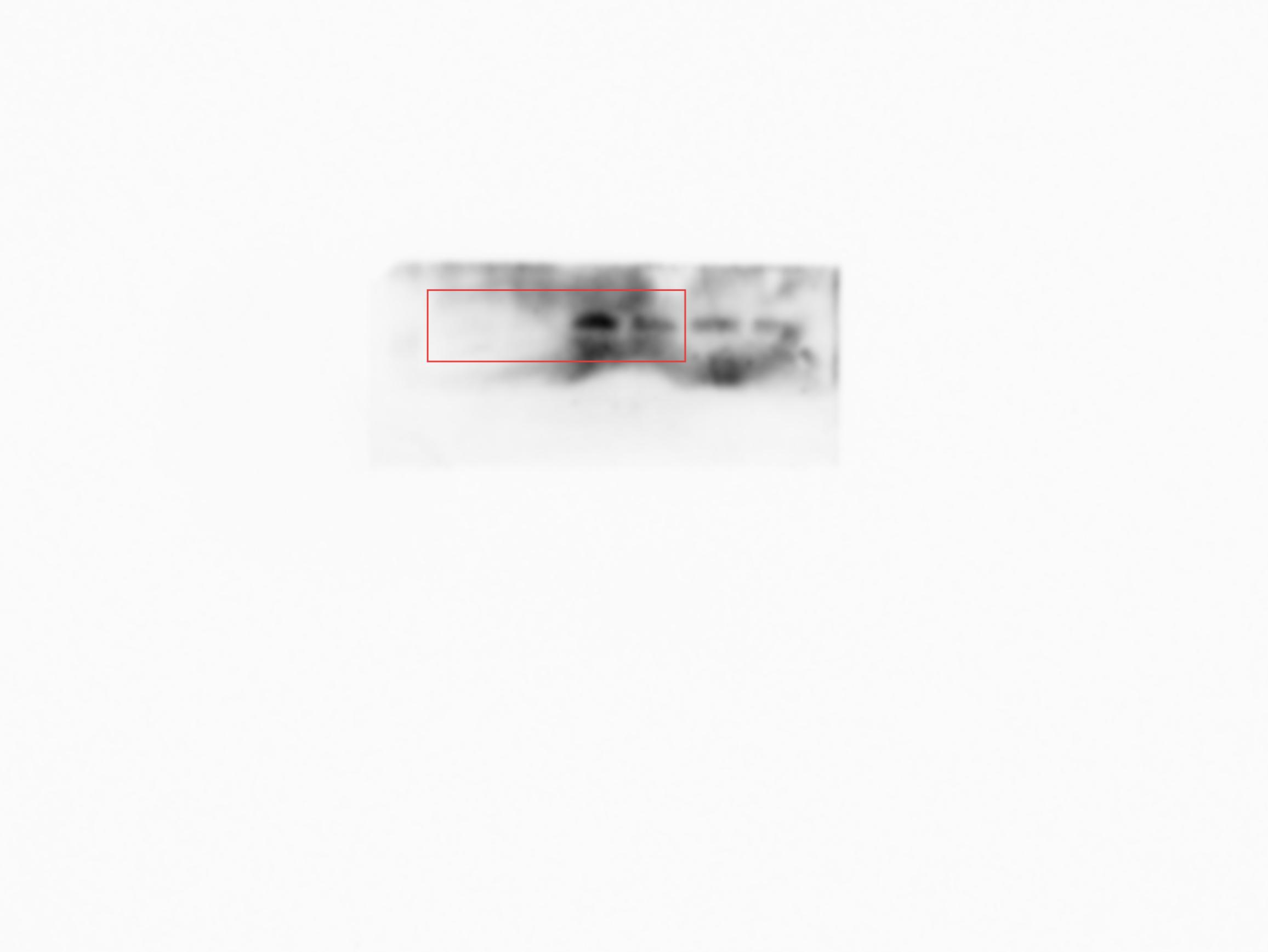

Supplement: Supplementary file 3 [file DataSheet1.zip › Fig2I GFP edited showing band.jpg]

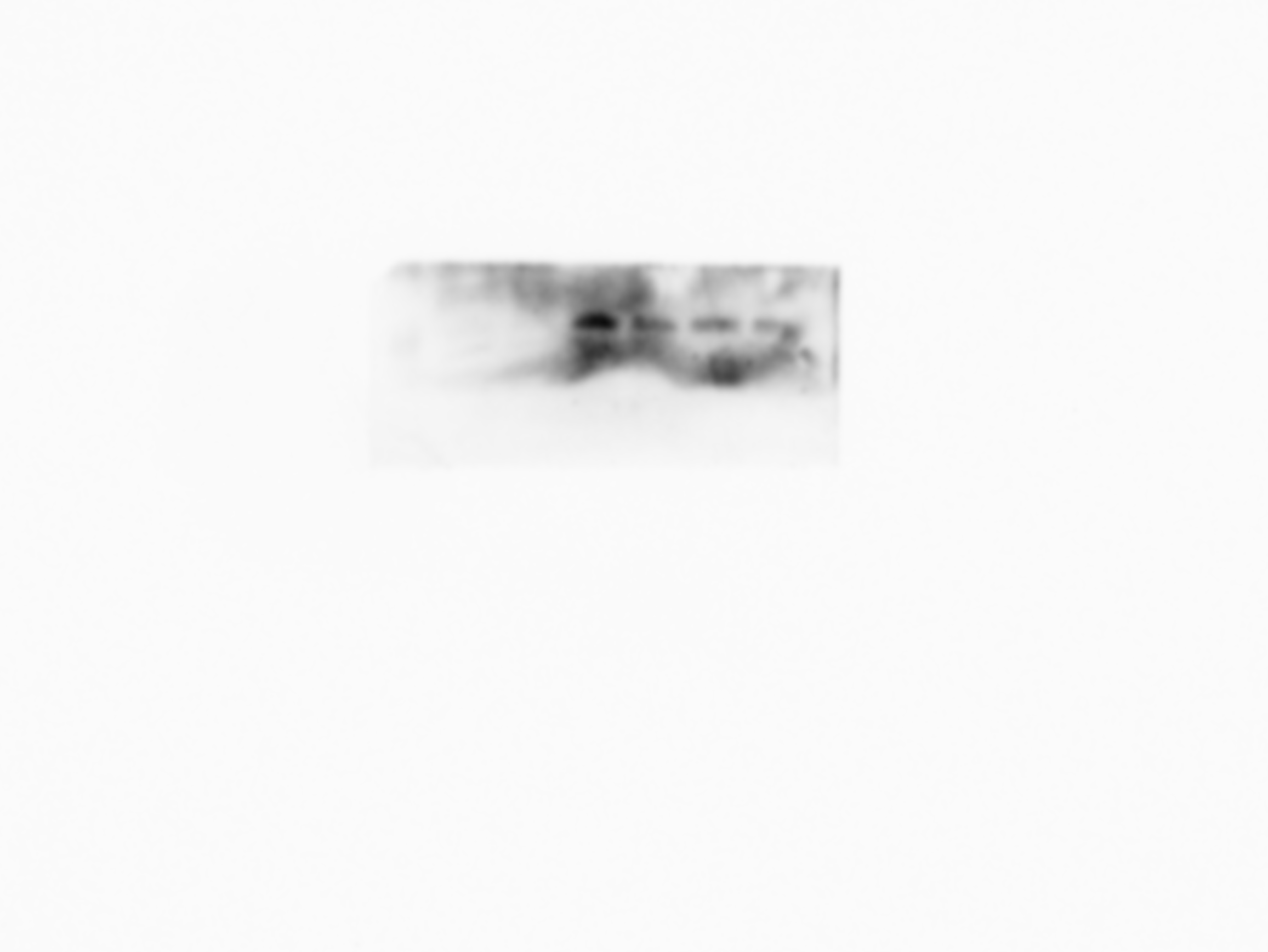

Supplement: Supplementary file 3 [file DataSheet1.zip › Fig2I GFP.tif]

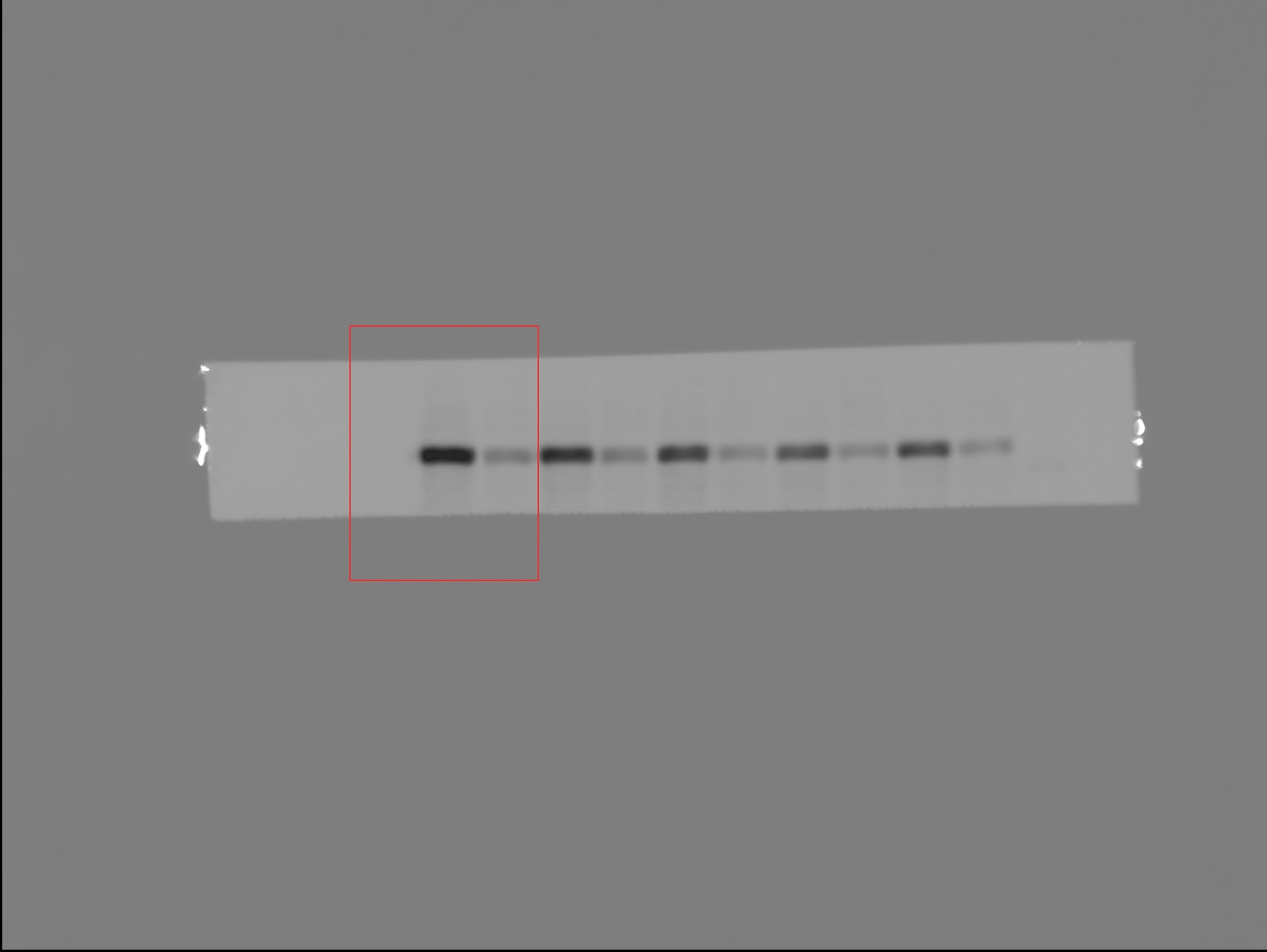

Supplement: Supplementary file 3 [file DataSheet1.zip › Fig2I TRIM28 edited showing band.jpg]

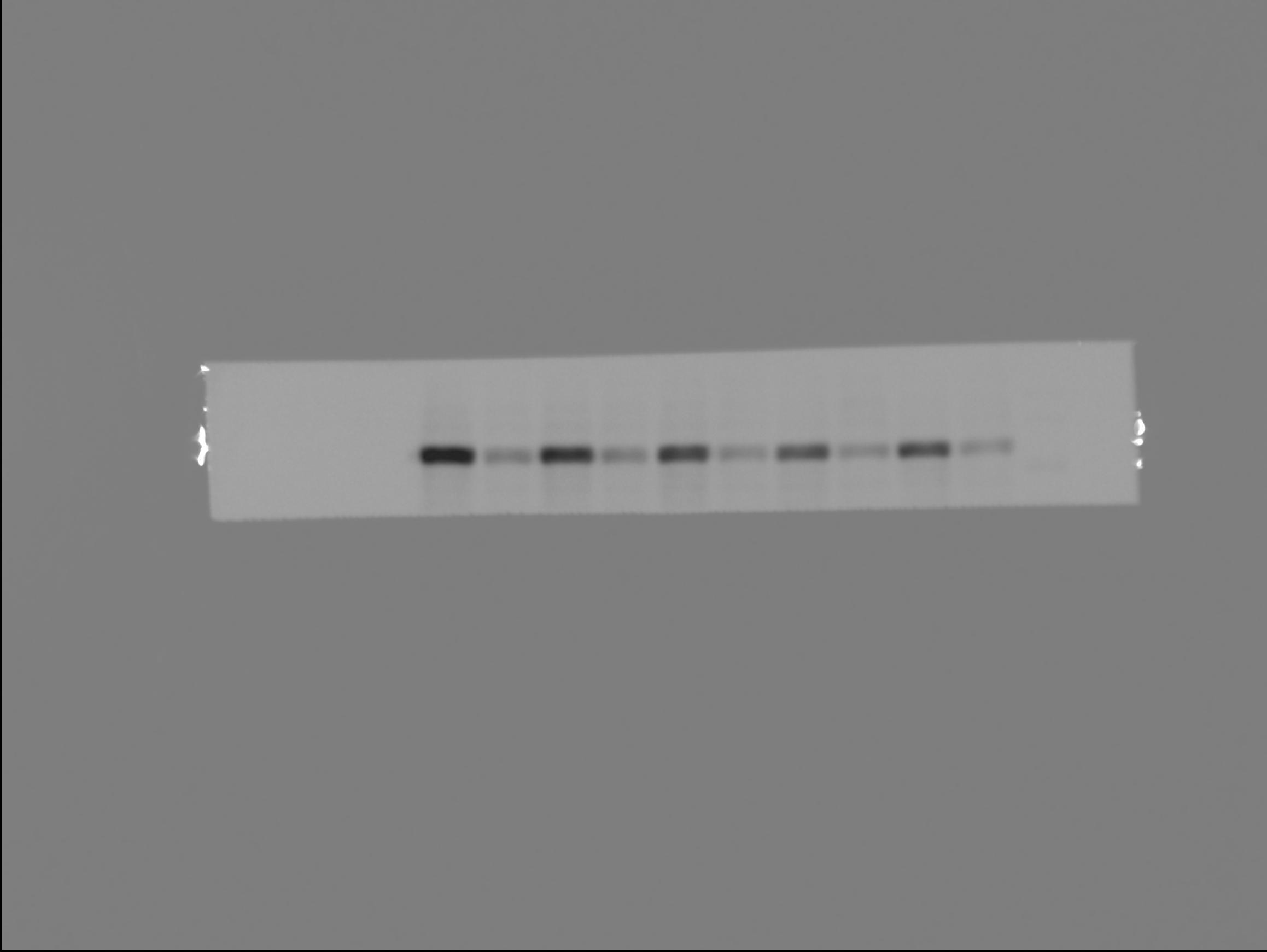

Supplement: Supplementary file 3 [file DataSheet1.zip › Fig2I TRIM28.jpg]

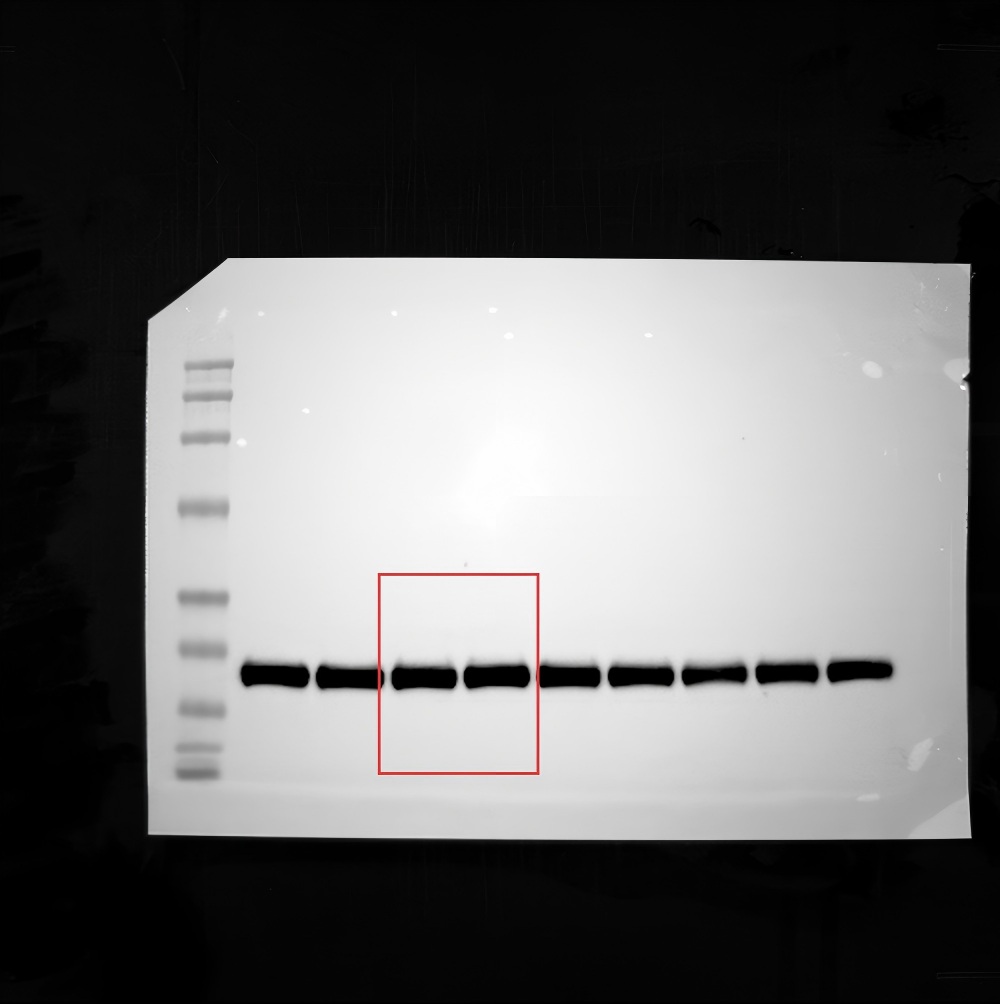

Supplement: Supplementary file 4 [file DataSheet2.zip › Fig3A GAPDH edited showing band.jpg]

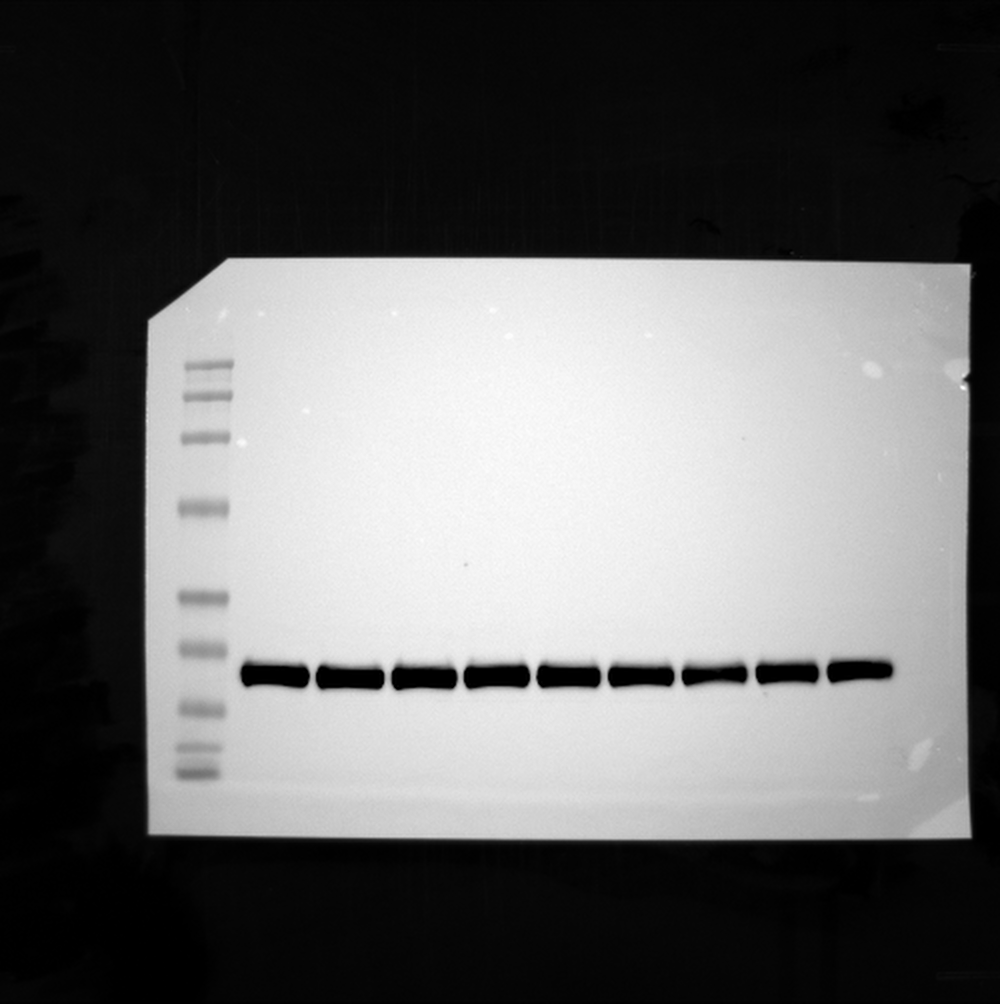

Supplement: Supplementary file 4 [file DataSheet2.zip › Fig3A GAPDH.tif]

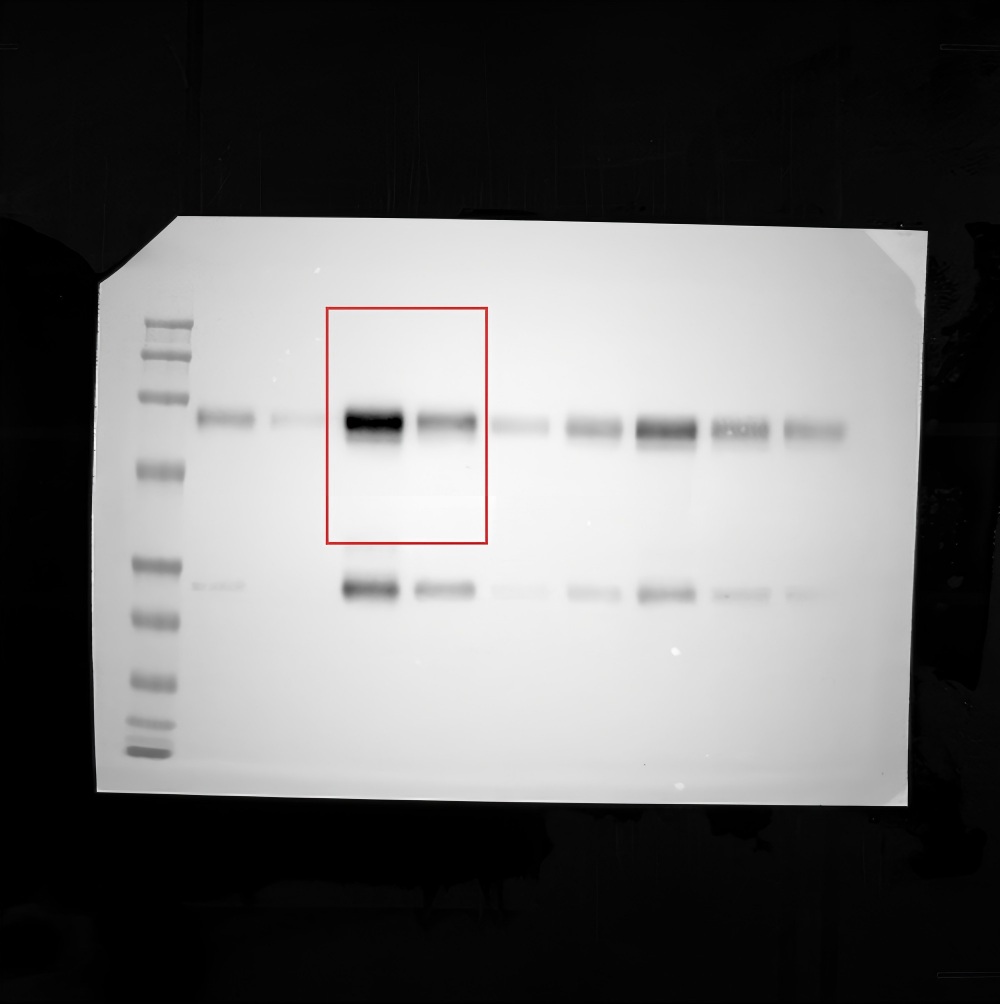

Supplement: Supplementary file 4 [file DataSheet2.zip › Fig3A TRIM28 edited showing band.jpg]

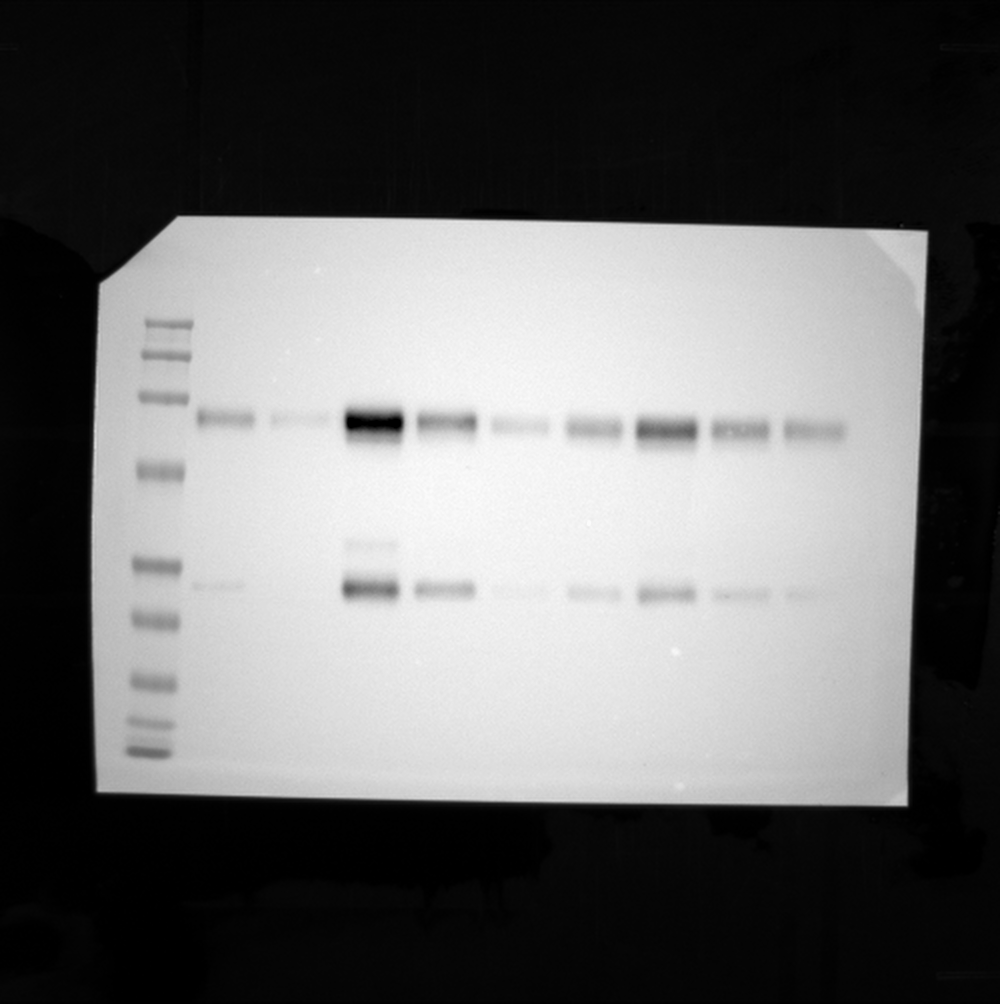

Supplement: Supplementary file 4 [file DataSheet2.zip › Fig3A TRIM28.tif]

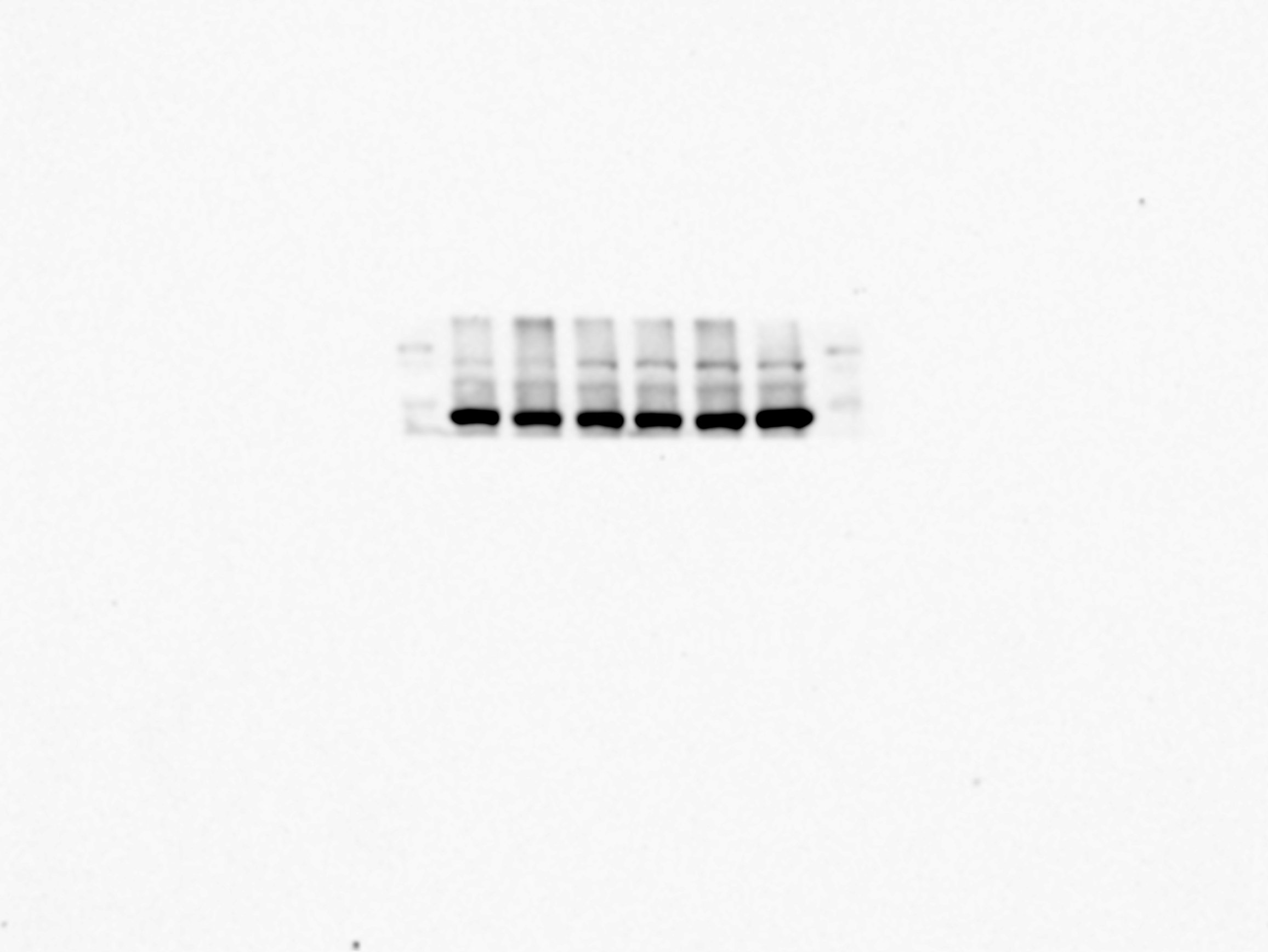

Supplement: Supplementary file 4 [file DataSheet2.zip › Fig3D GAPDH.tif]

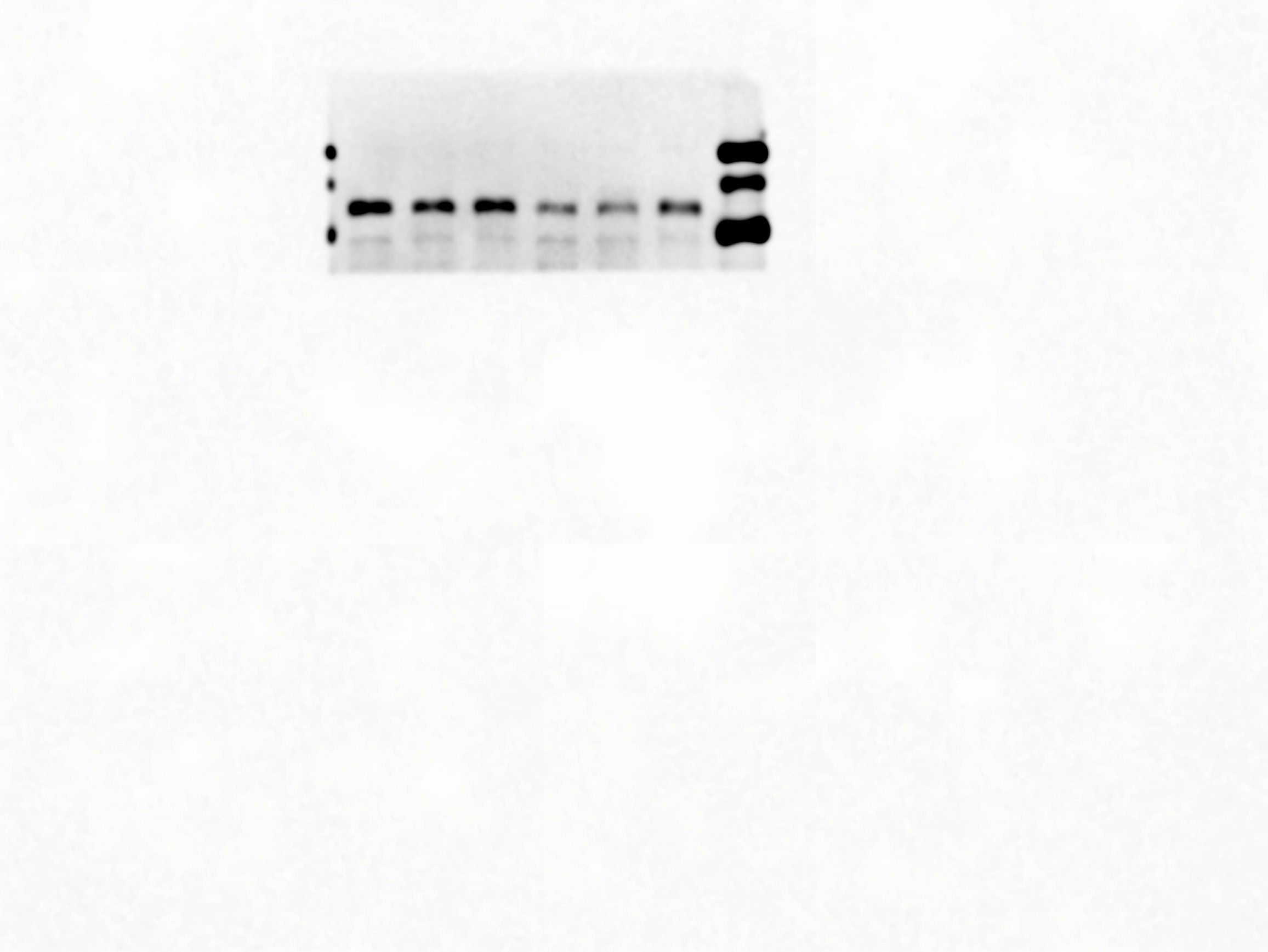

Supplement: Supplementary file 4 [file DataSheet2.zip › Fig3D VSV-G.jpg]

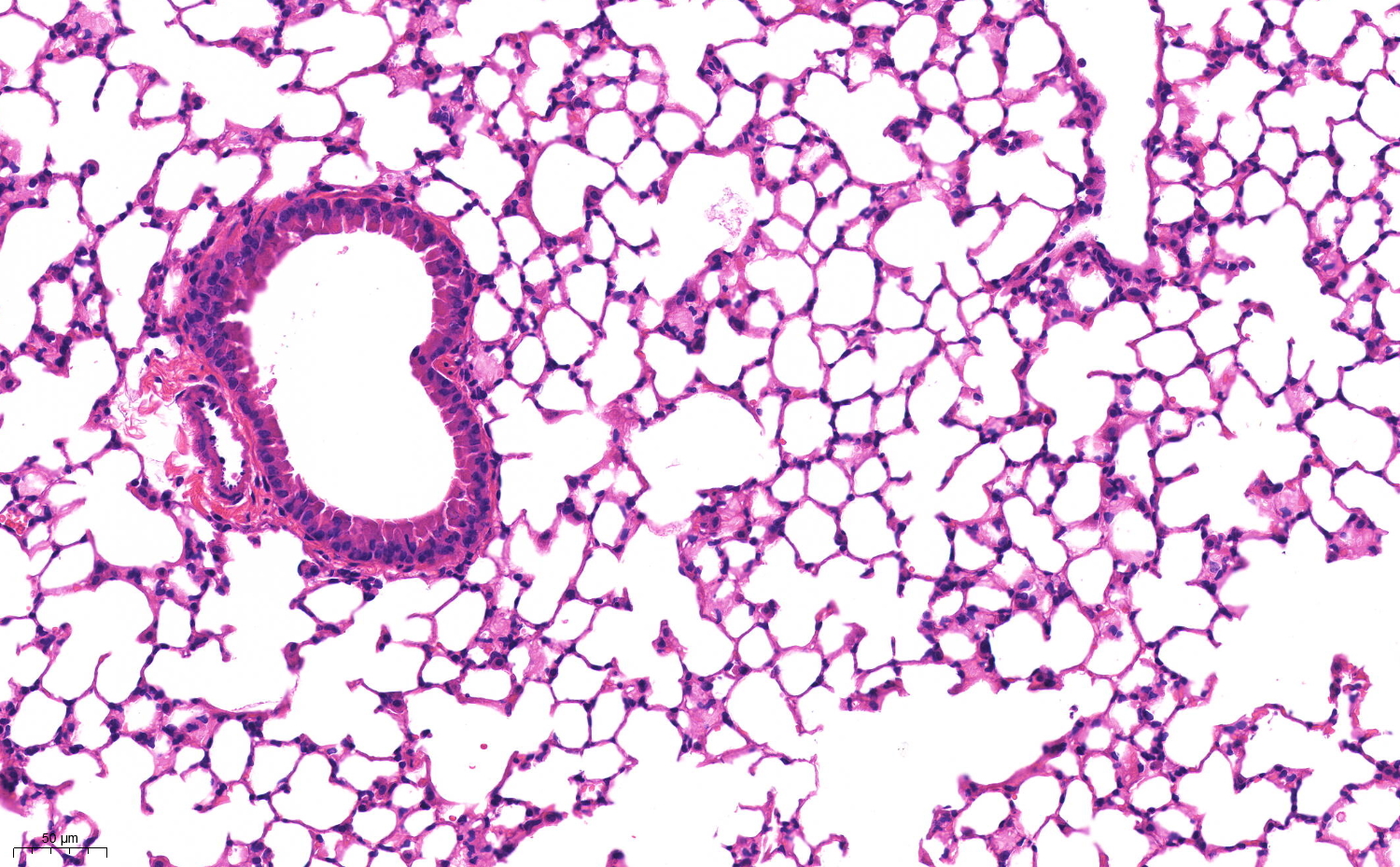

Supplement: Supplementary file 4 [file DataSheet2.zip › Fig3G TRIM28 KD-PBS 400x.jpg]

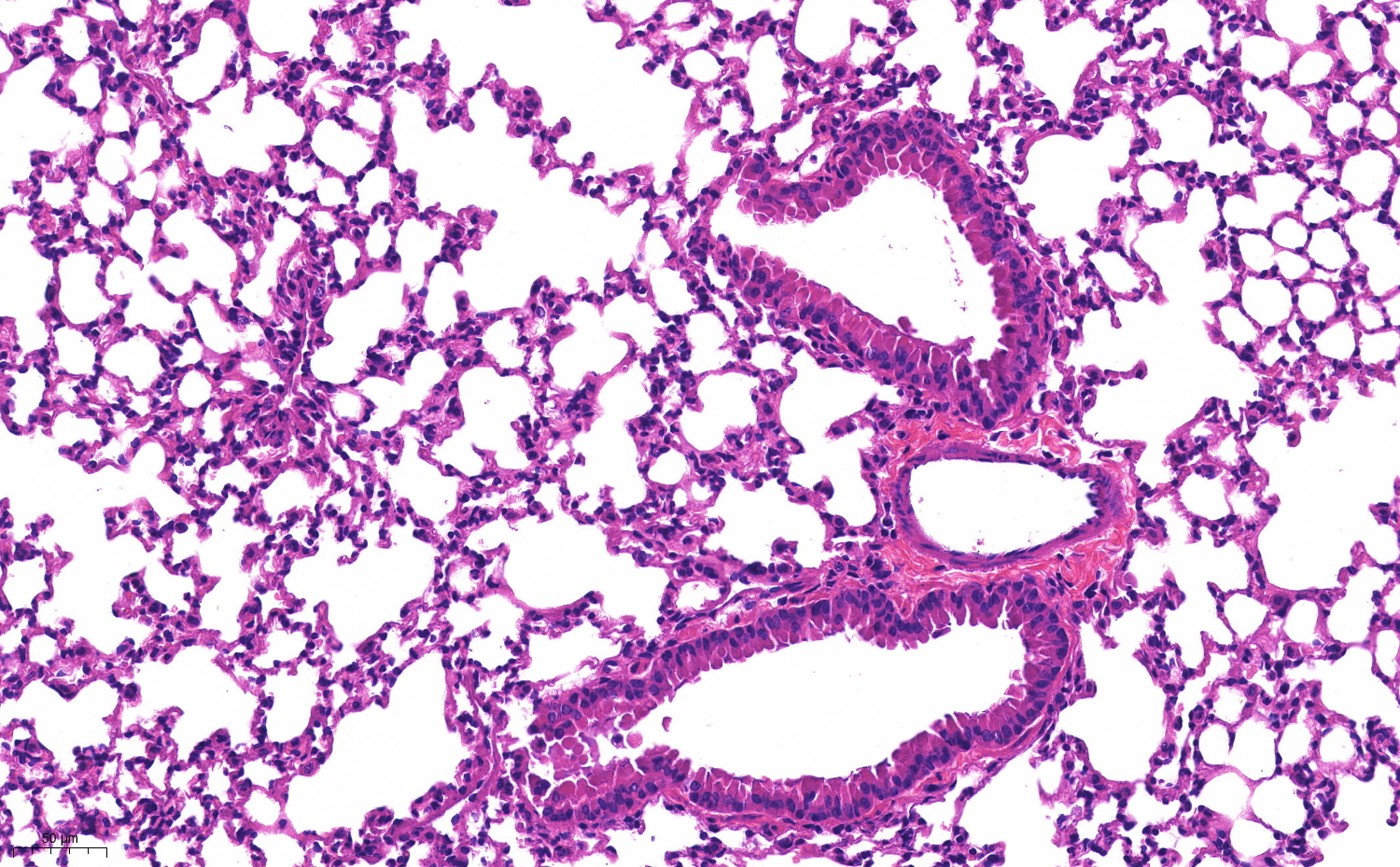

Supplement: Supplementary file 4 [file DataSheet2.zip › Fig3G TRIM28 KD-VSV 400x.jpg]

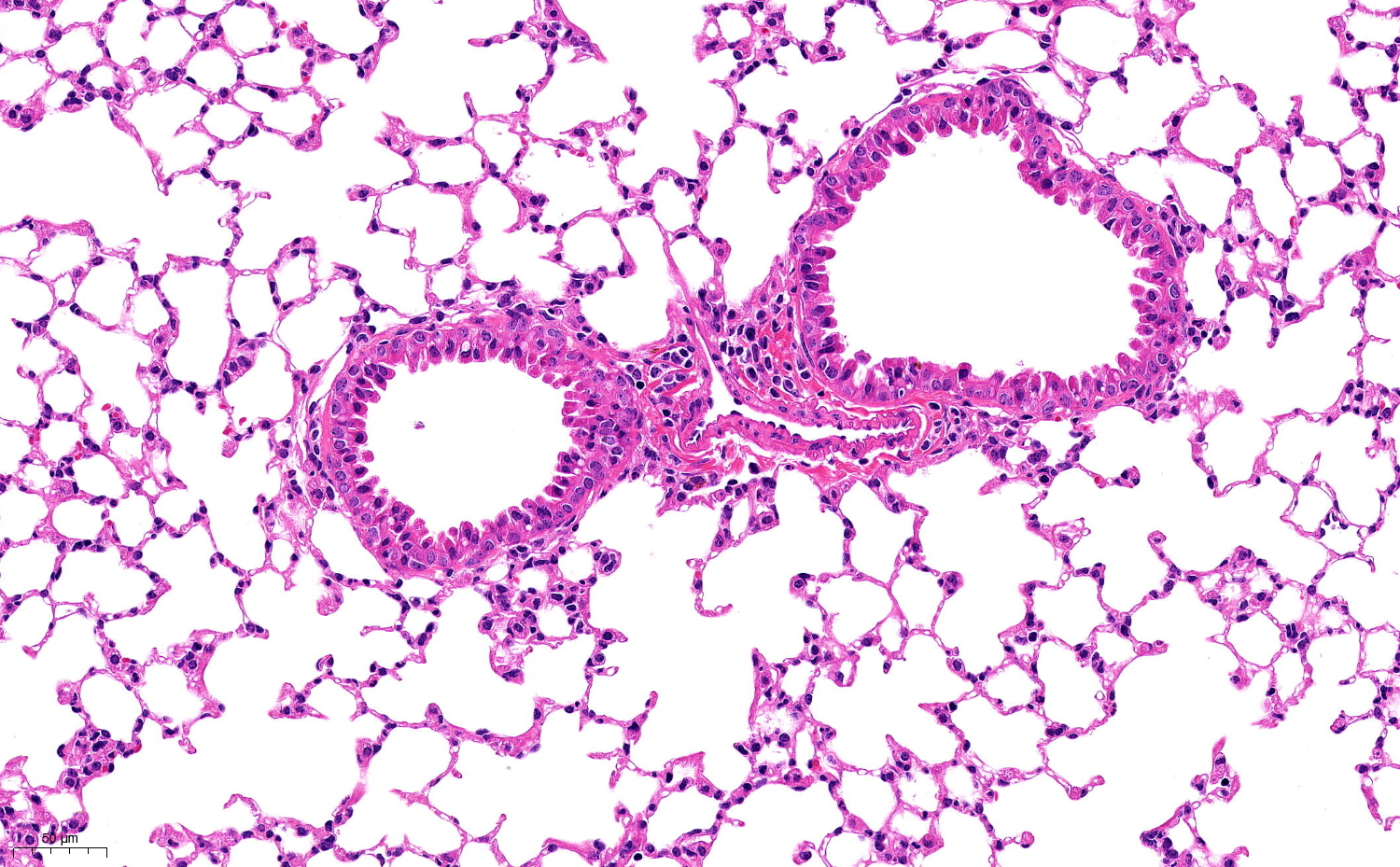

Supplement: Supplementary file 4 [file DataSheet2.zip › Fig3G WT-PBS 400x.jpg]

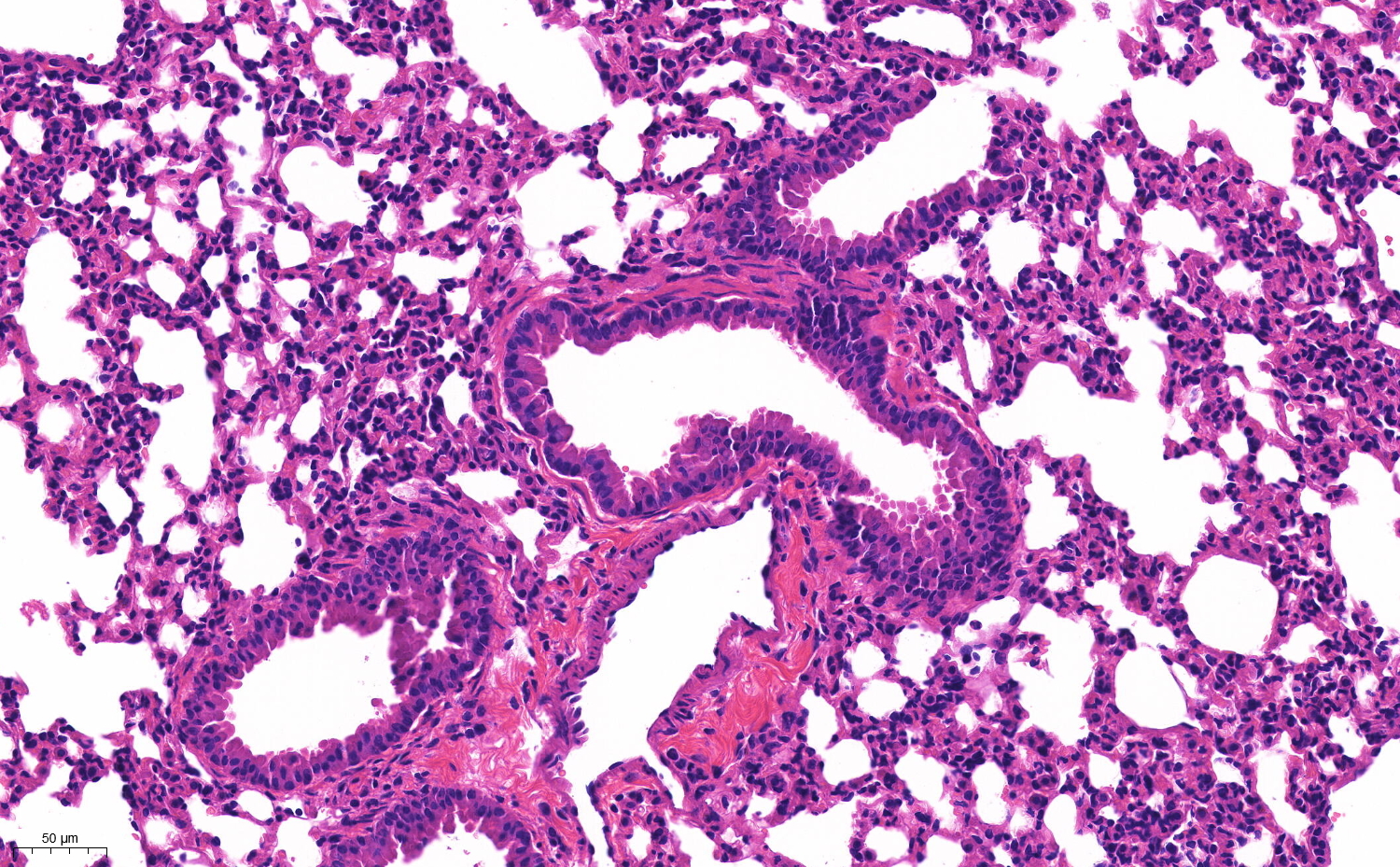

Supplement: Supplementary file 4 [file DataSheet2.zip › Fig3G WT-VSV 400x.jpg]

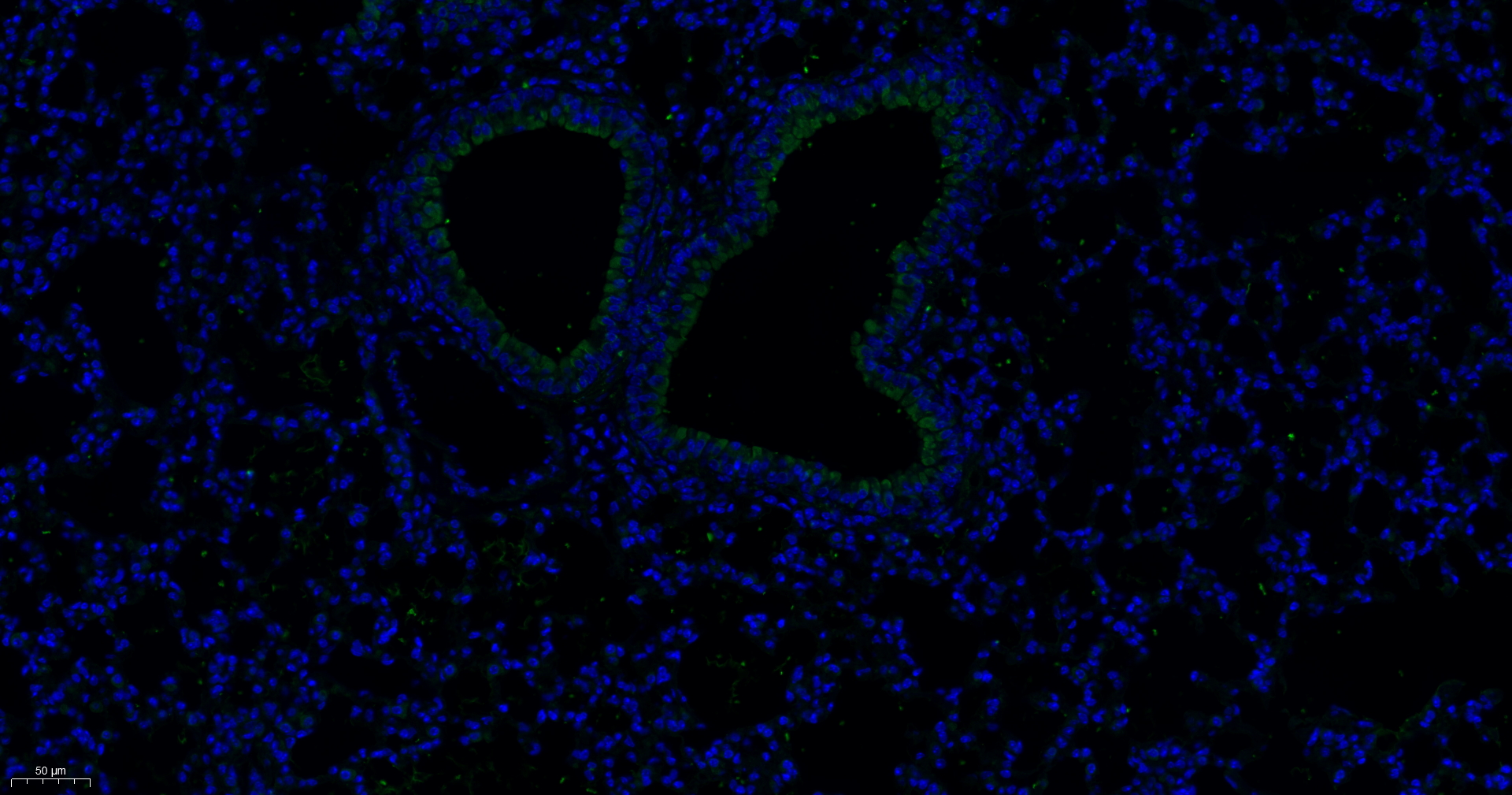

Supplement: Supplementary file 4 [file DataSheet2.zip › Fig3H TRIM28 KD-VSV 400x.jpg]

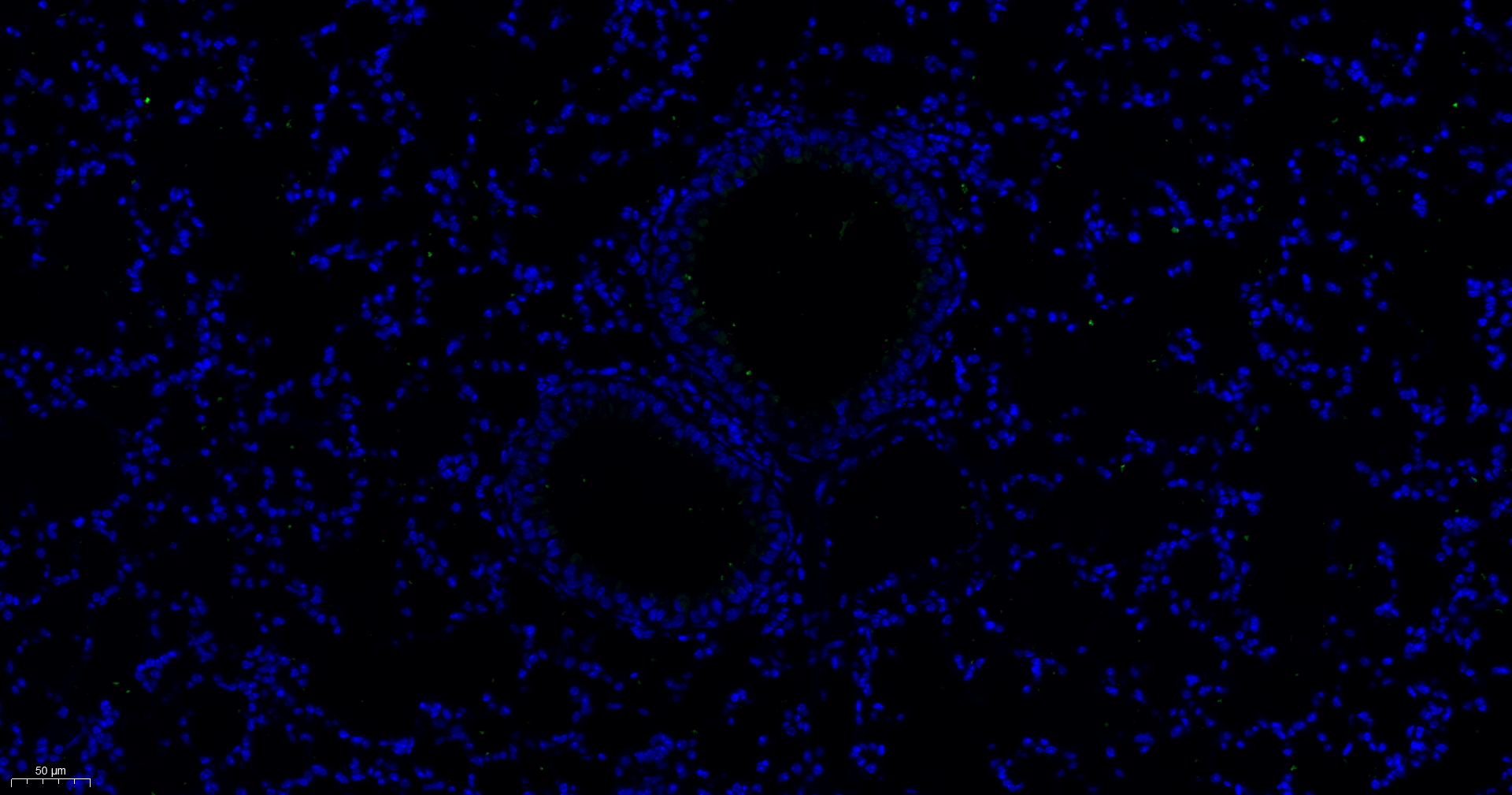

Supplement: Supplementary file 4 [file DataSheet2.zip › Fig3H WT-PBS 400x.jpg]

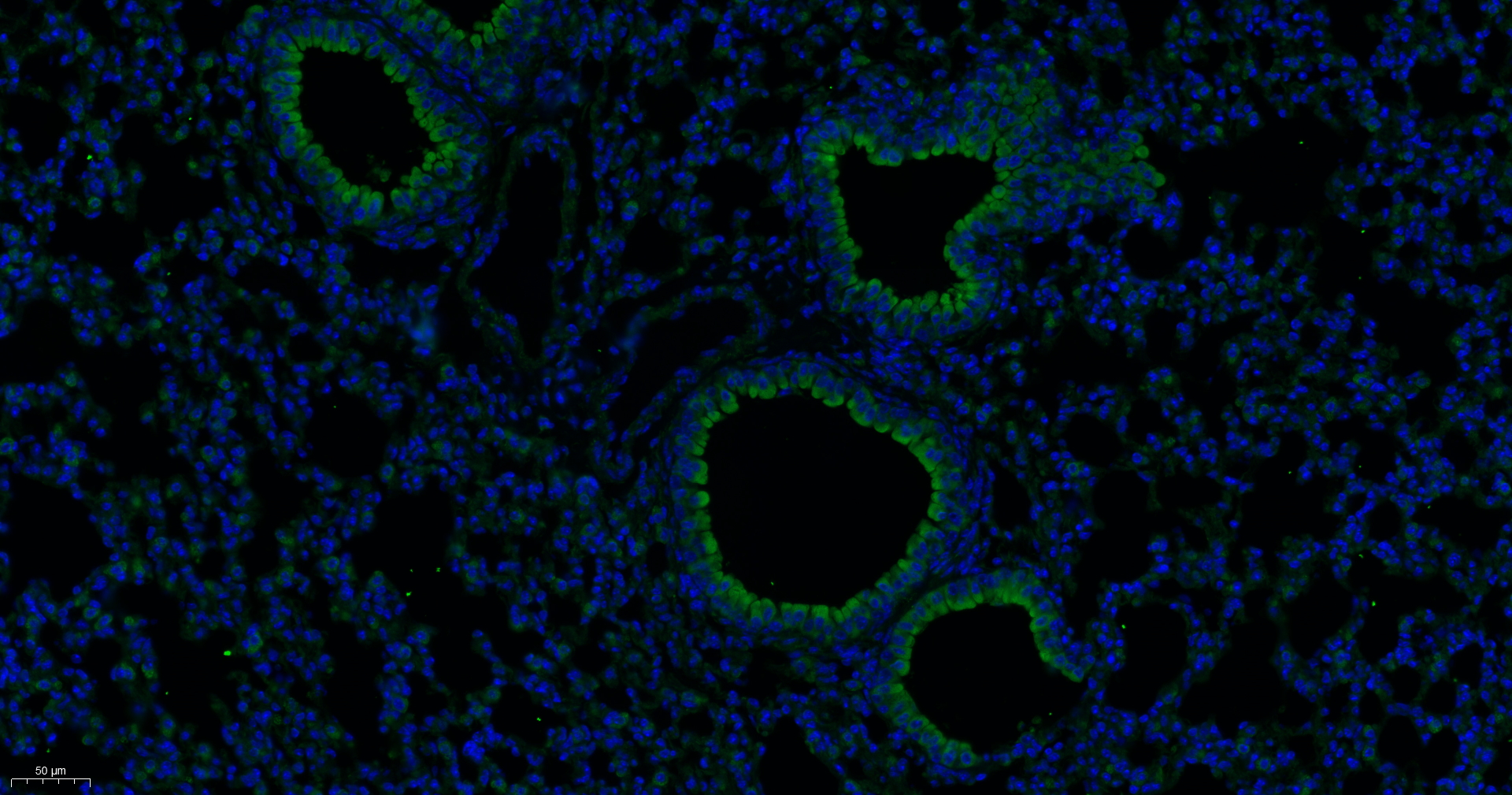

Supplement: Supplementary file 4 [file DataSheet2.zip › Fig3H WT-VSV 400x.jpg]

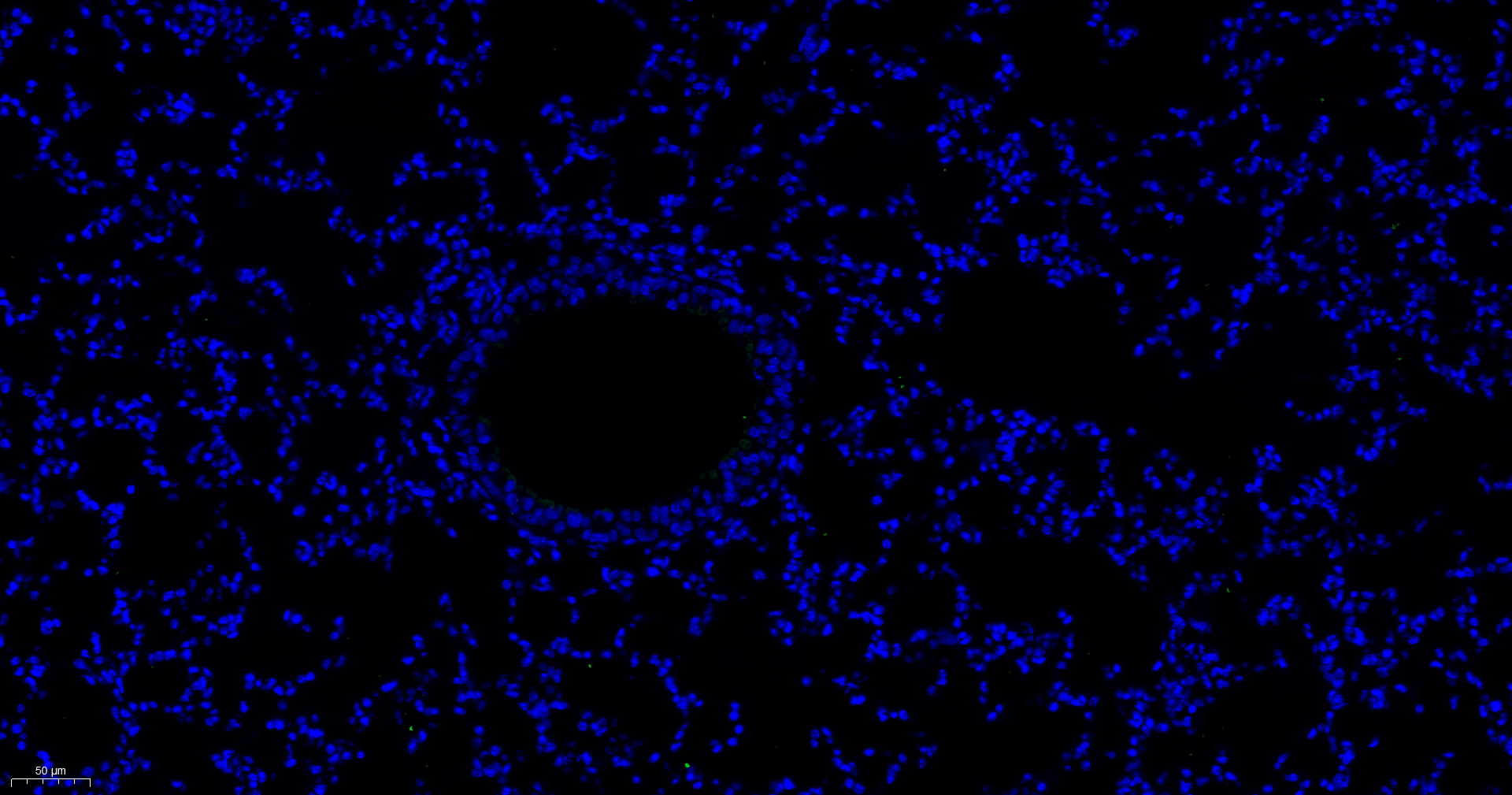

Supplement: Supplementary file 4 [file DataSheet2.zip › Fig3HTRIM28 KD-PBS 400x.jpg]

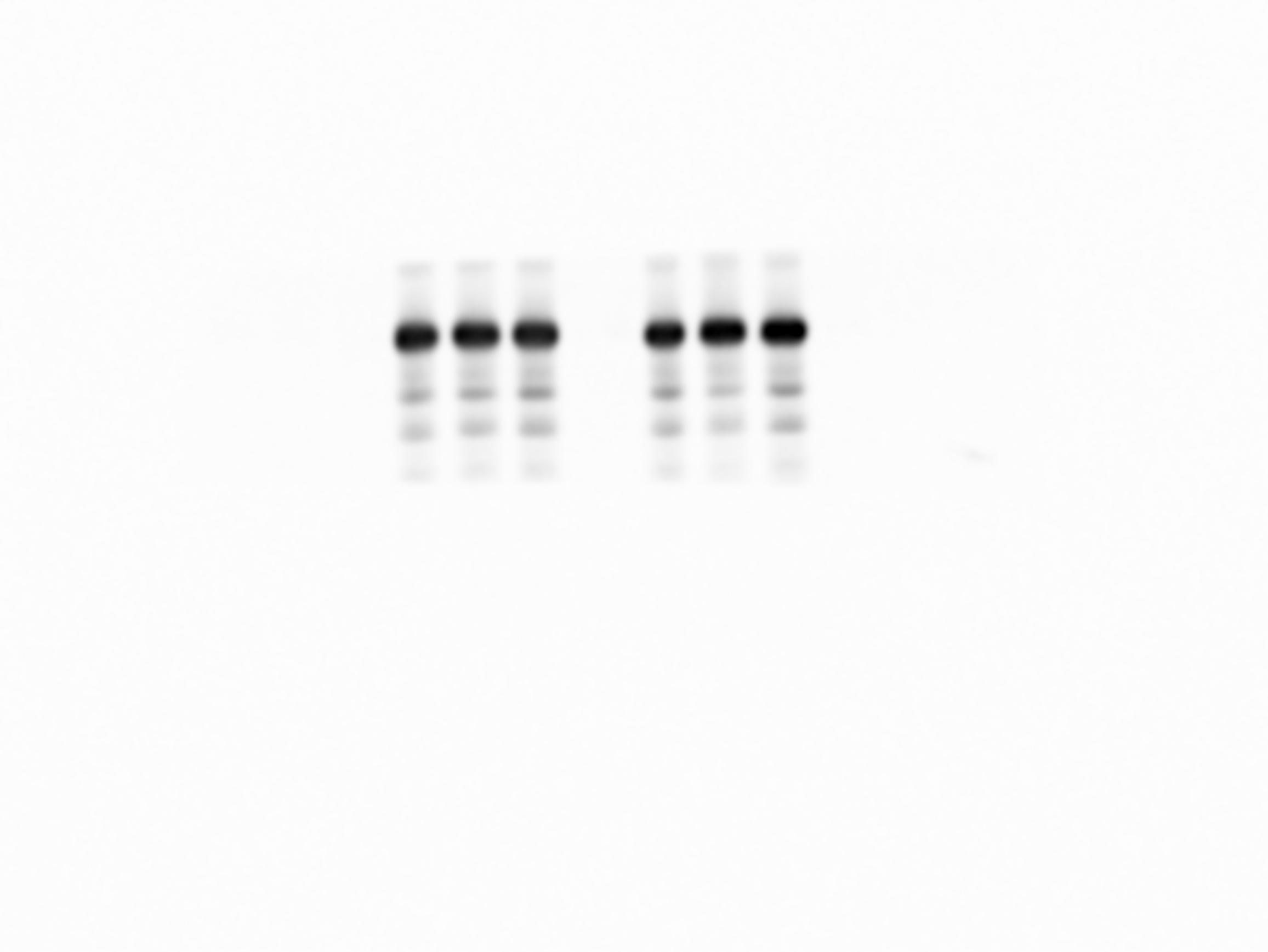

Supplement: Supplementary file 4 [file DataSheet2.zip › Fig4A GAPDH.tif]

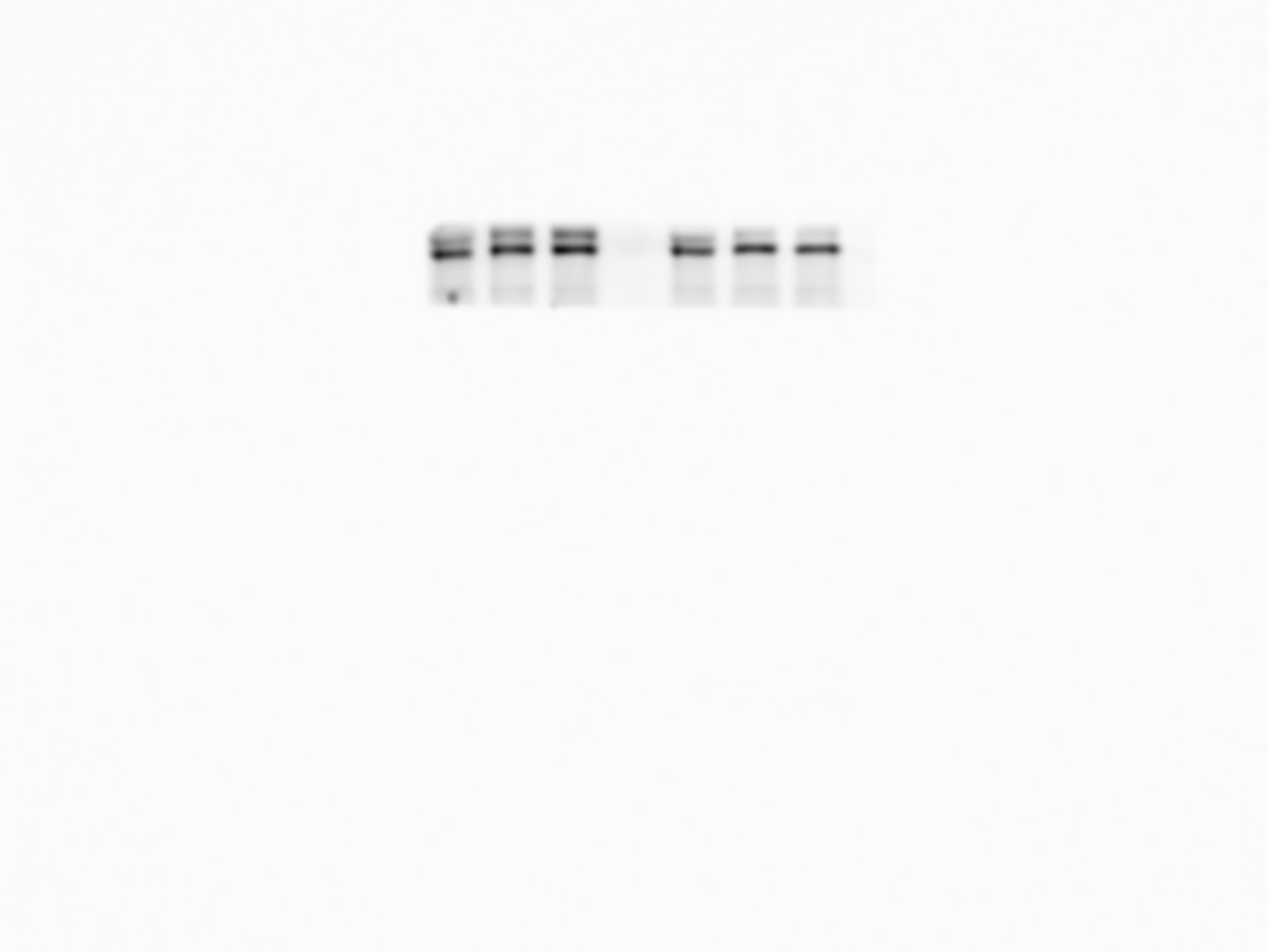

Supplement: Supplementary file 4 [file DataSheet2.zip › Fig4A IRF3.tif]

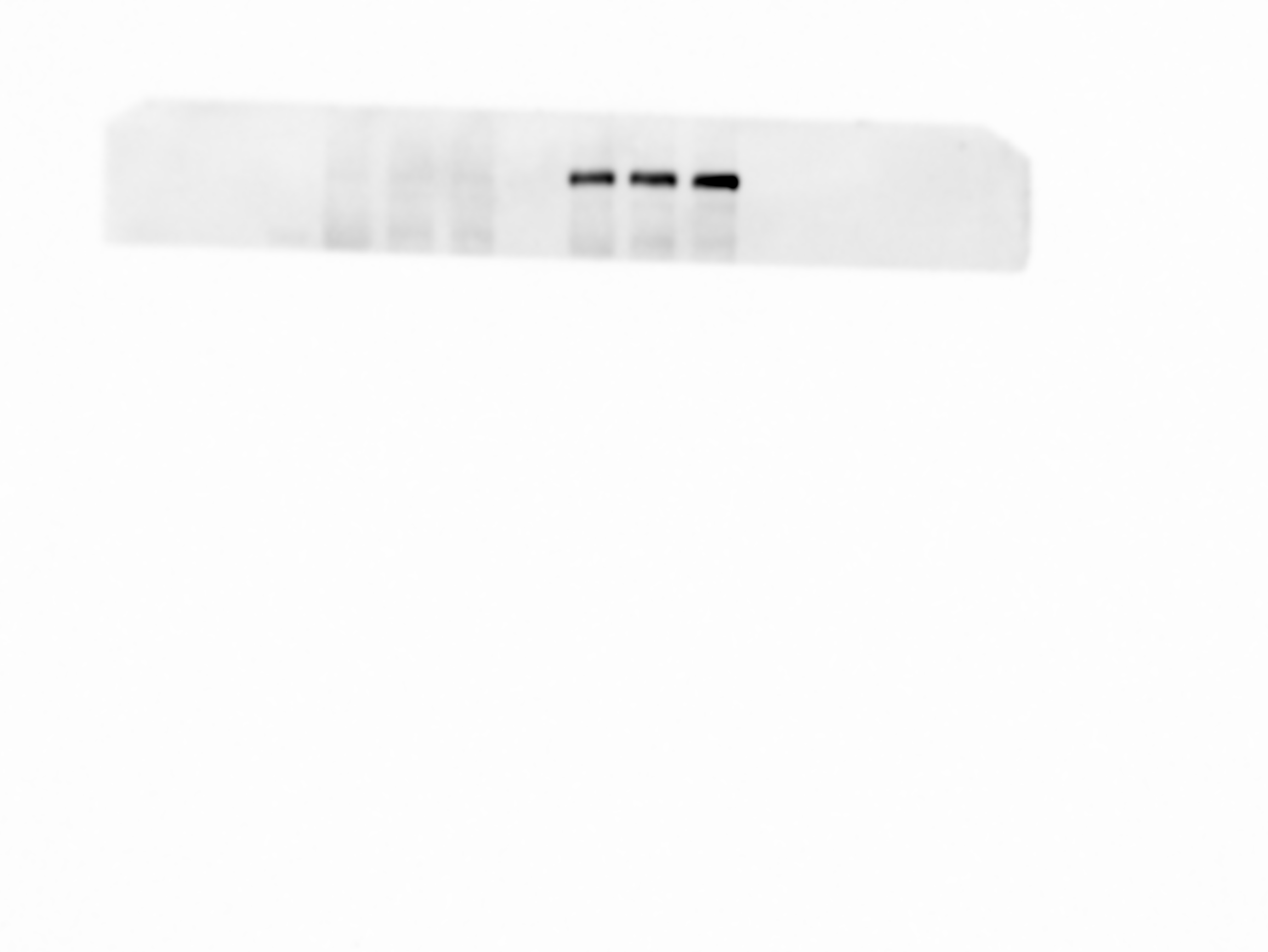

Supplement: Supplementary file 4 [file DataSheet2.zip › Fig4A Myc.tif]

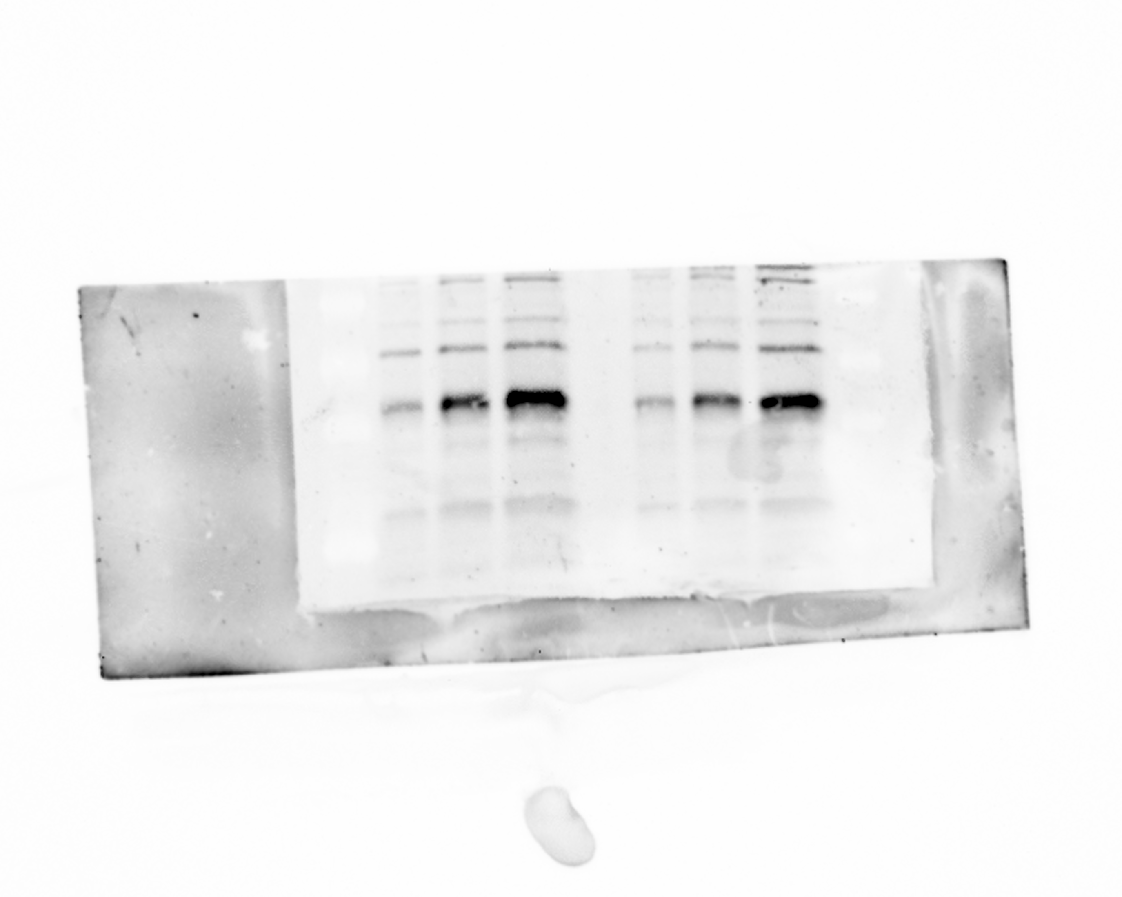

Supplement: Supplementary file 4 [file DataSheet2.zip › Fig4A p-IRF3.tif]

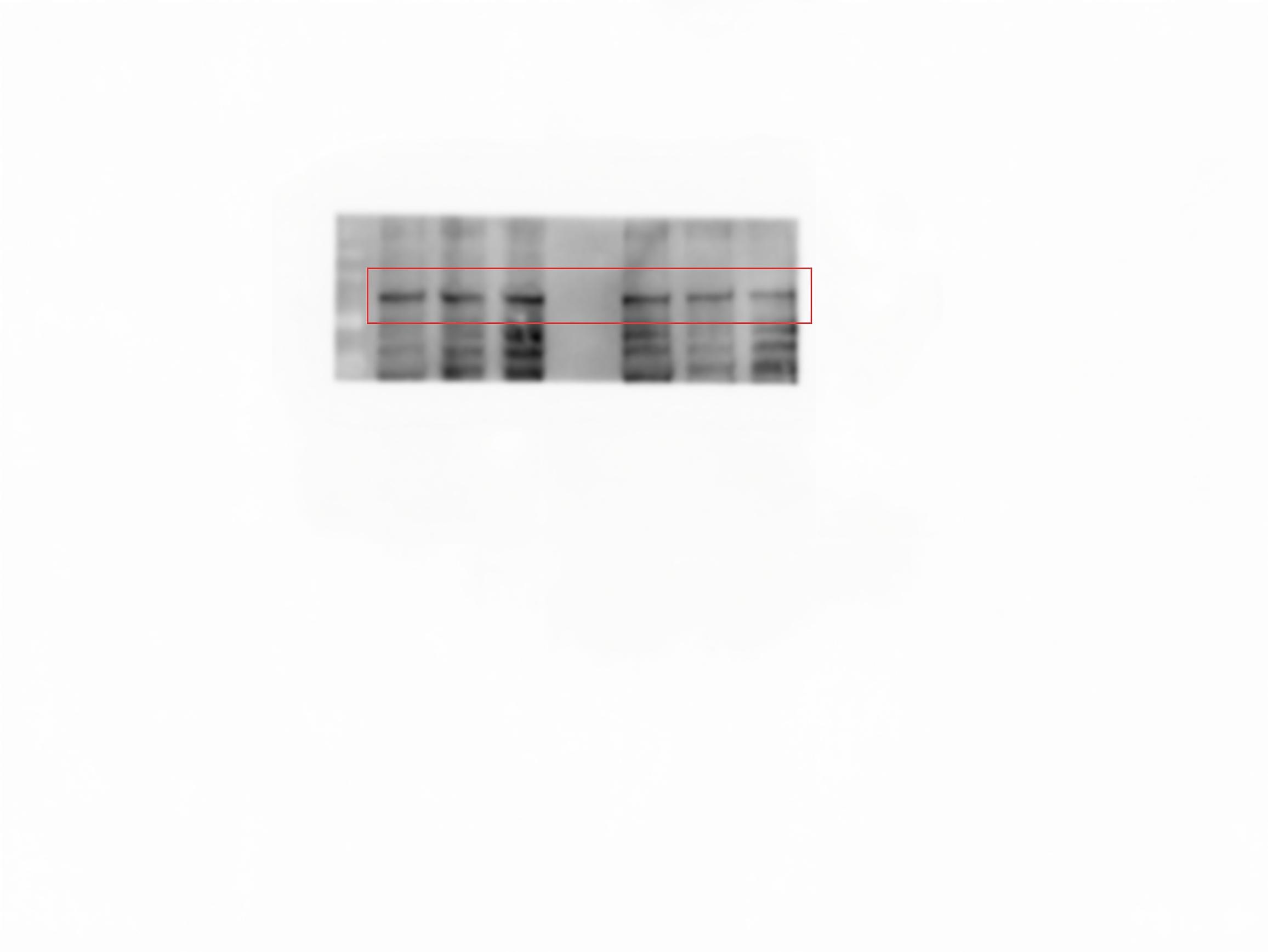

Supplement: Supplementary file 4 [file DataSheet2.zip › Fig4A p-TBK1 edited showing band.jpg]

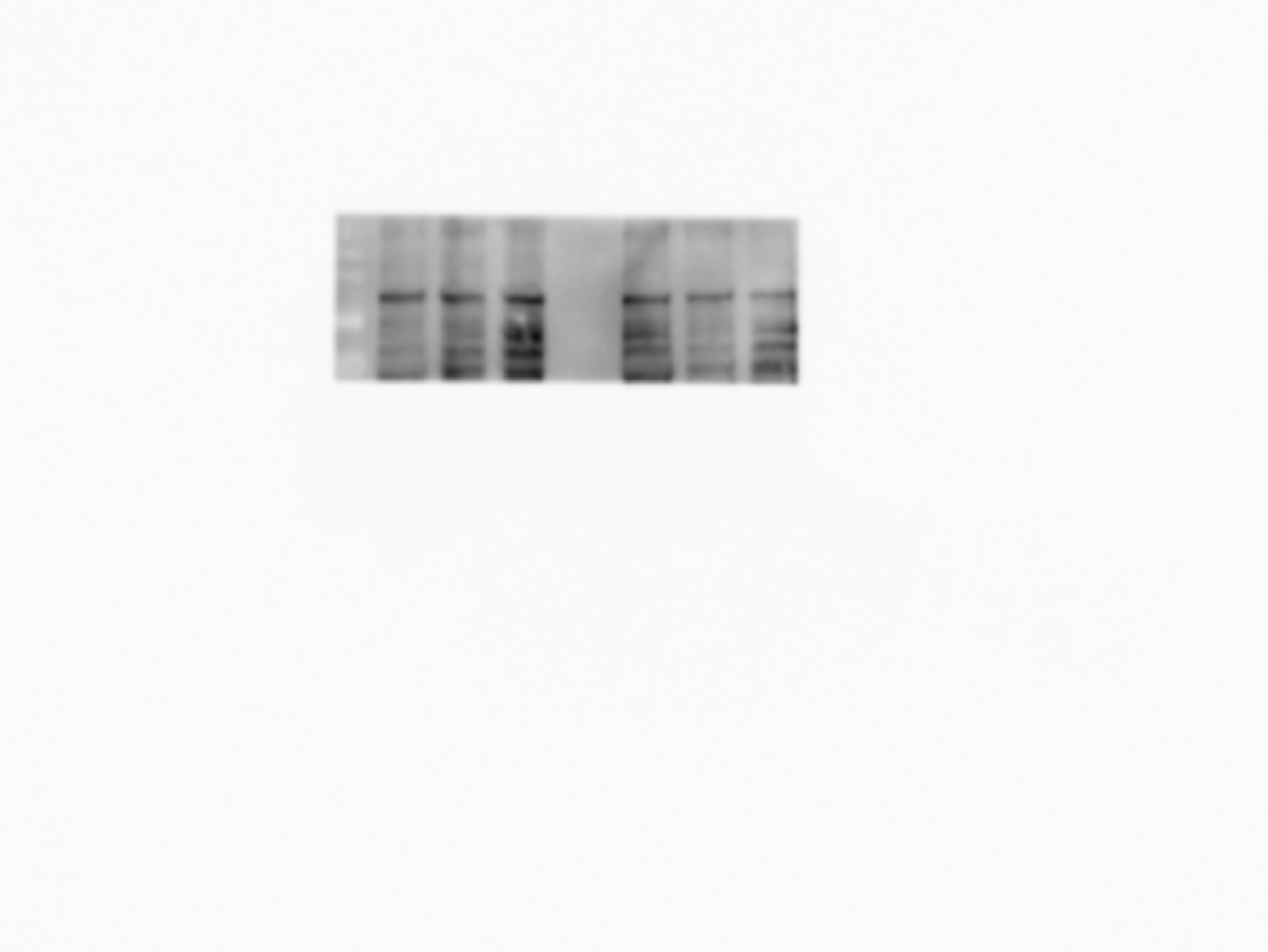

Supplement: Supplementary file 4 [file DataSheet2.zip › Fig4A p-TBK1.tif]

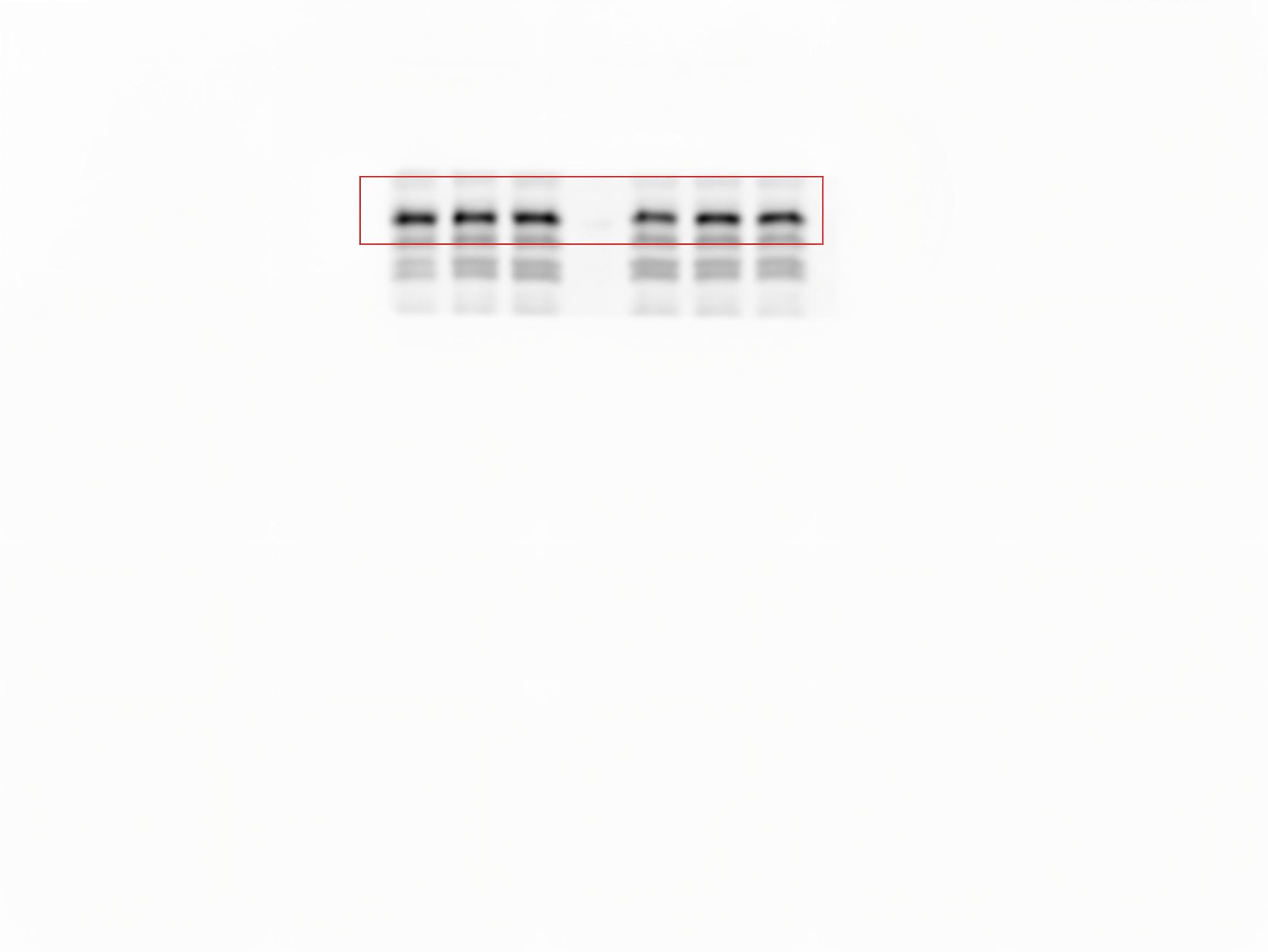

Supplement: Supplementary file 4 [file DataSheet2.zip › Fig4A TBK1 edited showing band.jpg]

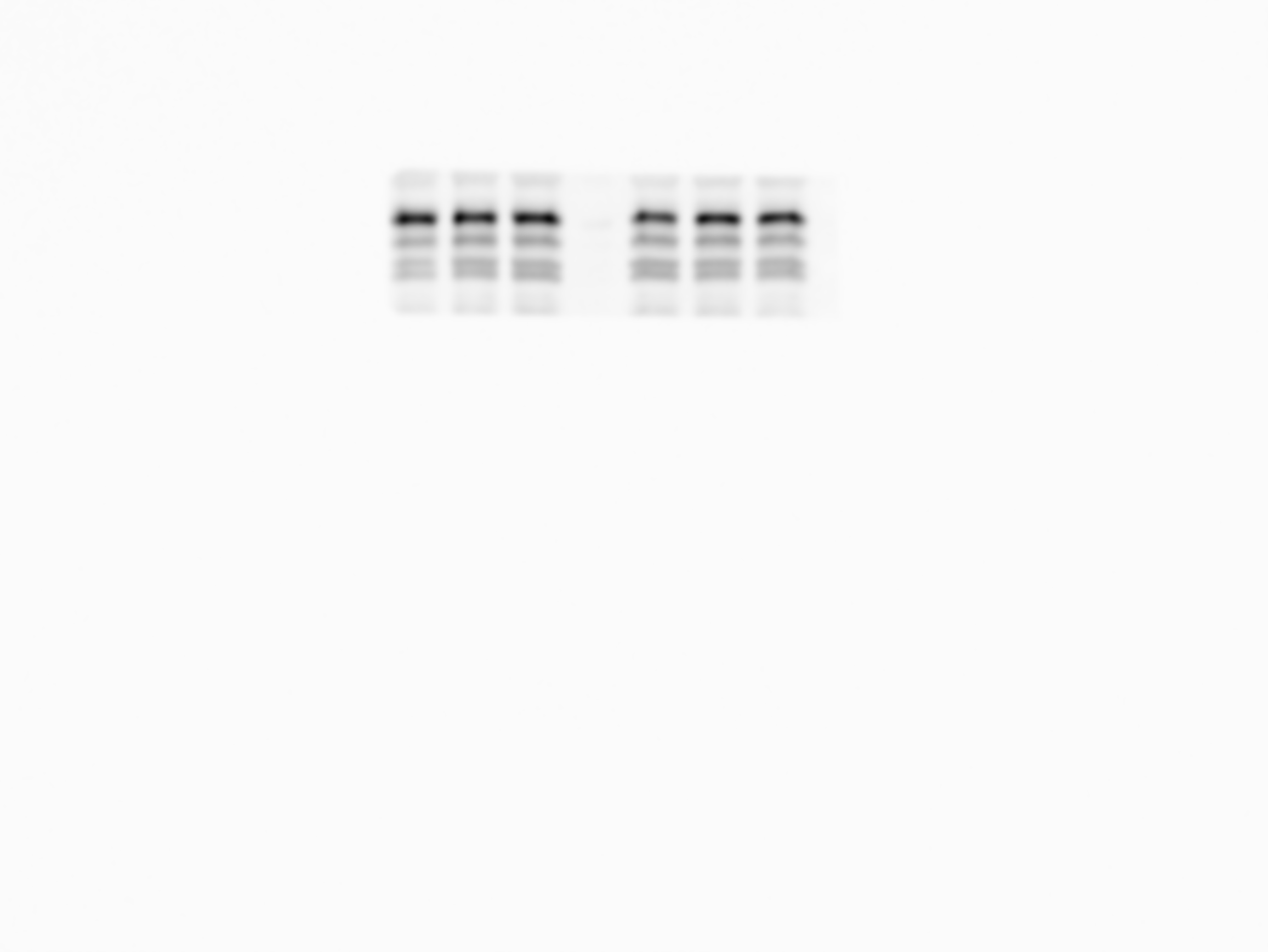

Supplement: Supplementary file 4 [file DataSheet2.zip › Fig4A TBK1.tif]

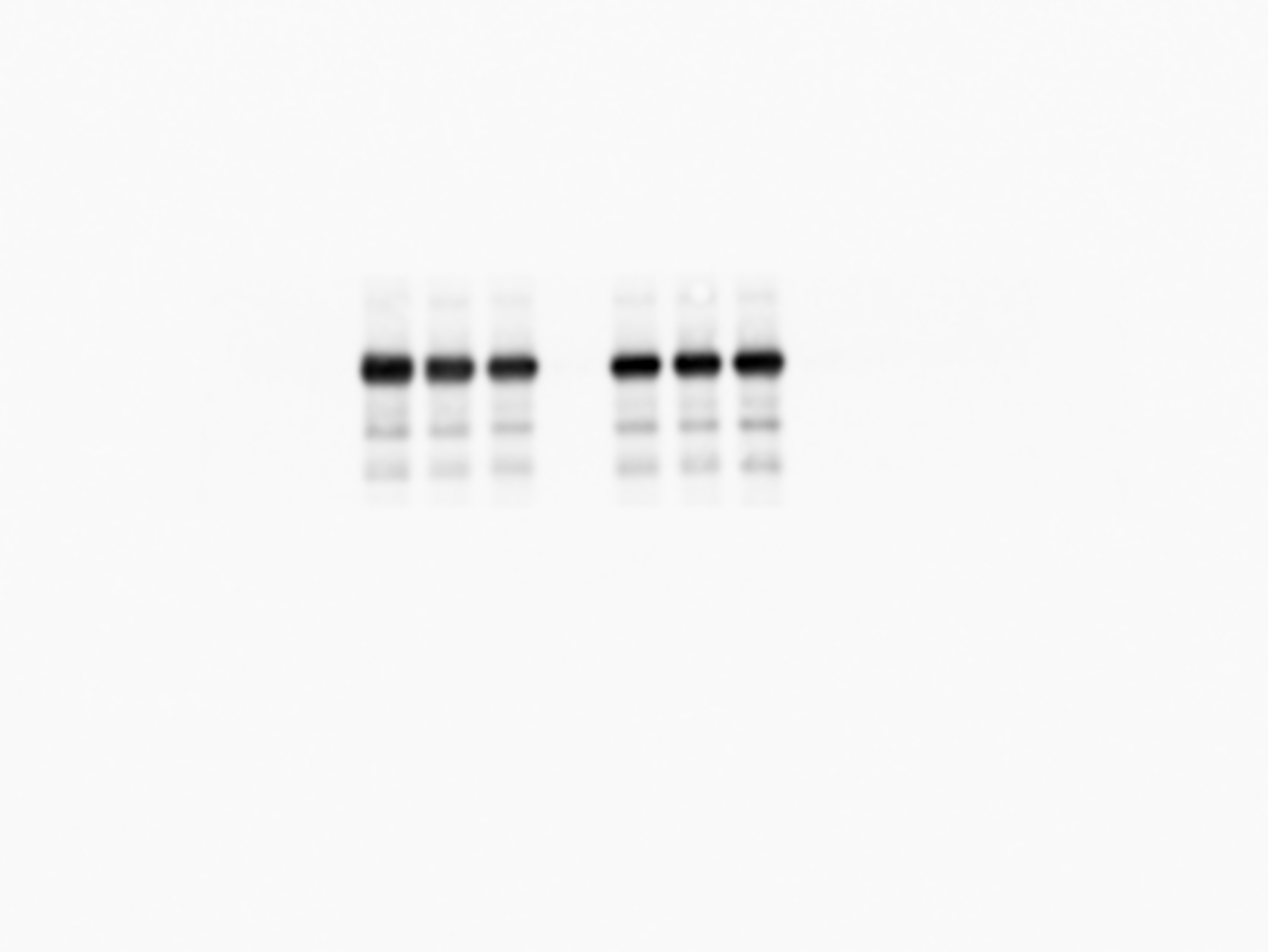

Supplement: Supplementary file 4 [file DataSheet2.zip › Fig4B Actin.tif]

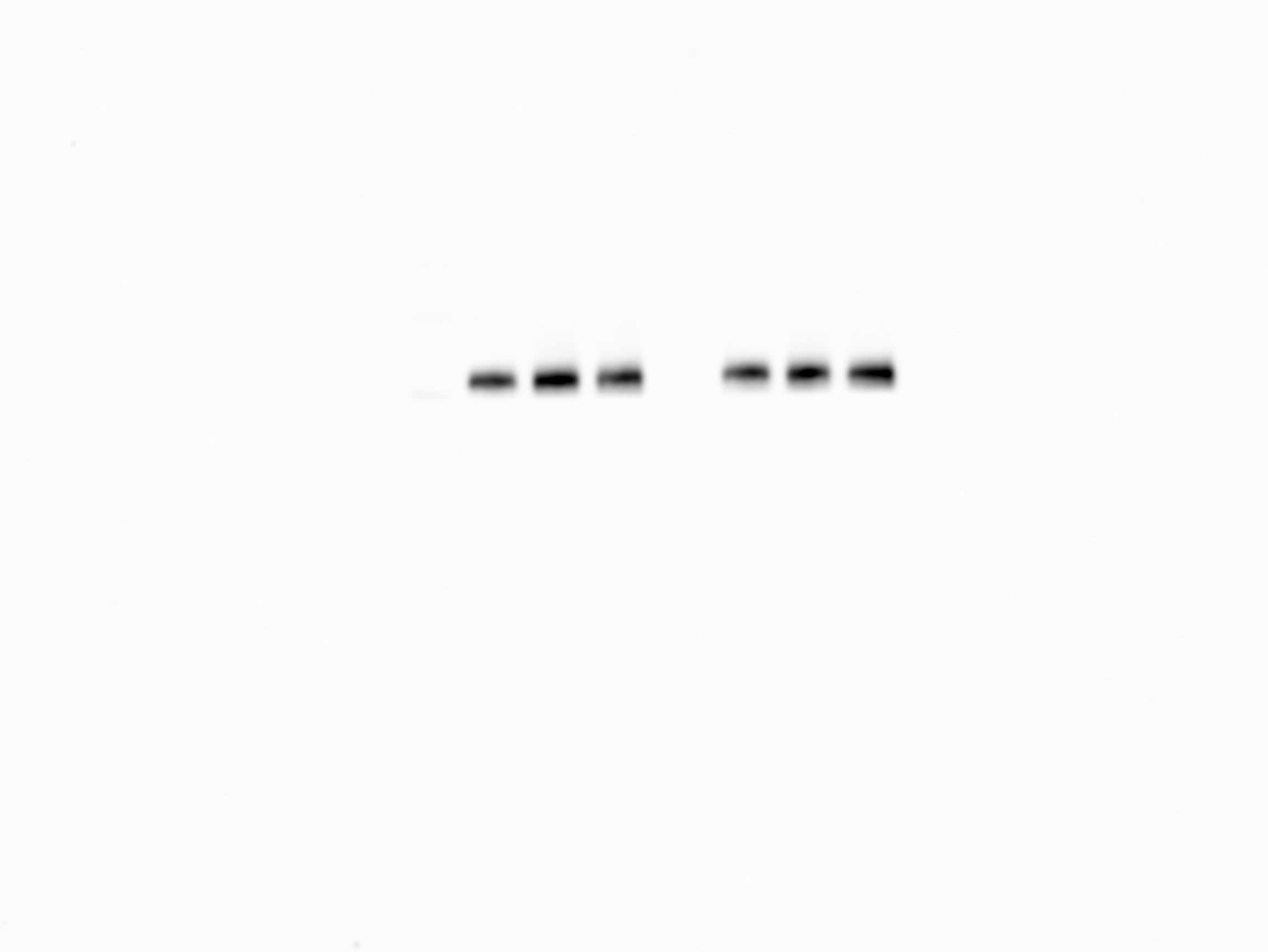

Supplement: Supplementary file 4 [file DataSheet2.zip › Fig4B IRF3.tif]

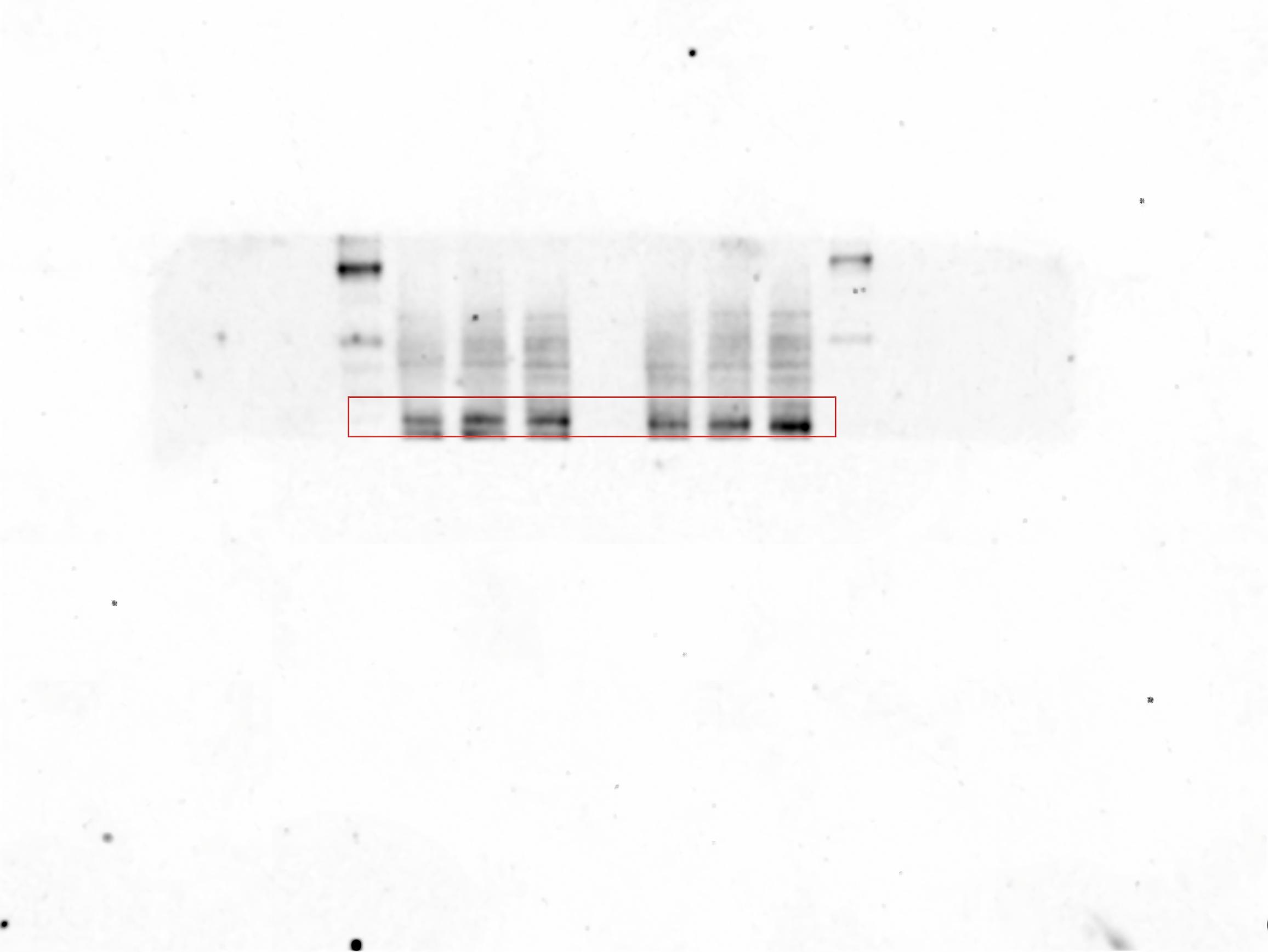

Supplement: Supplementary file 4 [file DataSheet2.zip › Fig4B p-IRF3 edited showing band.jpg]

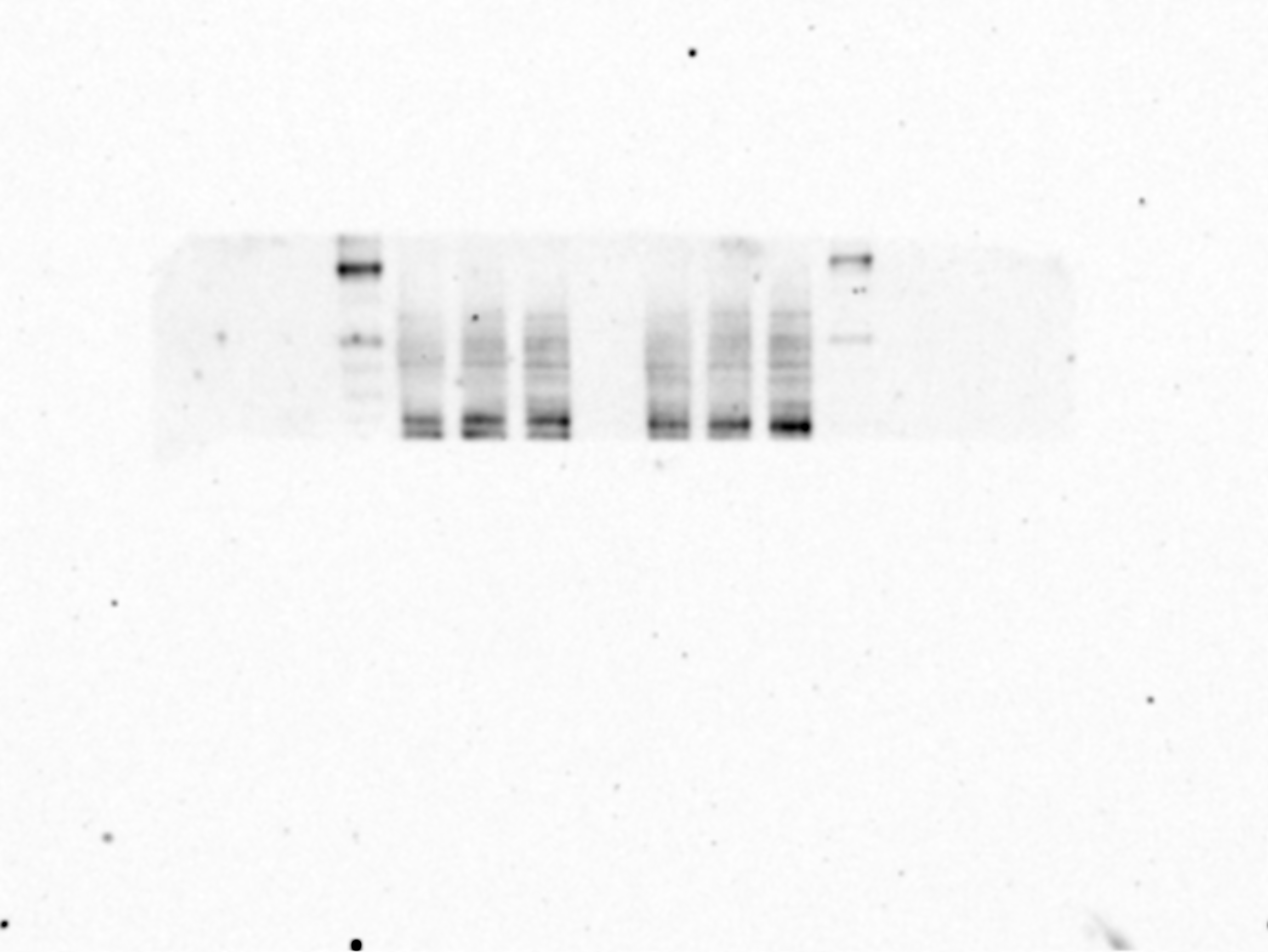

Supplement: Supplementary file 4 [file DataSheet2.zip › Fig4B p-IRF3.tif]

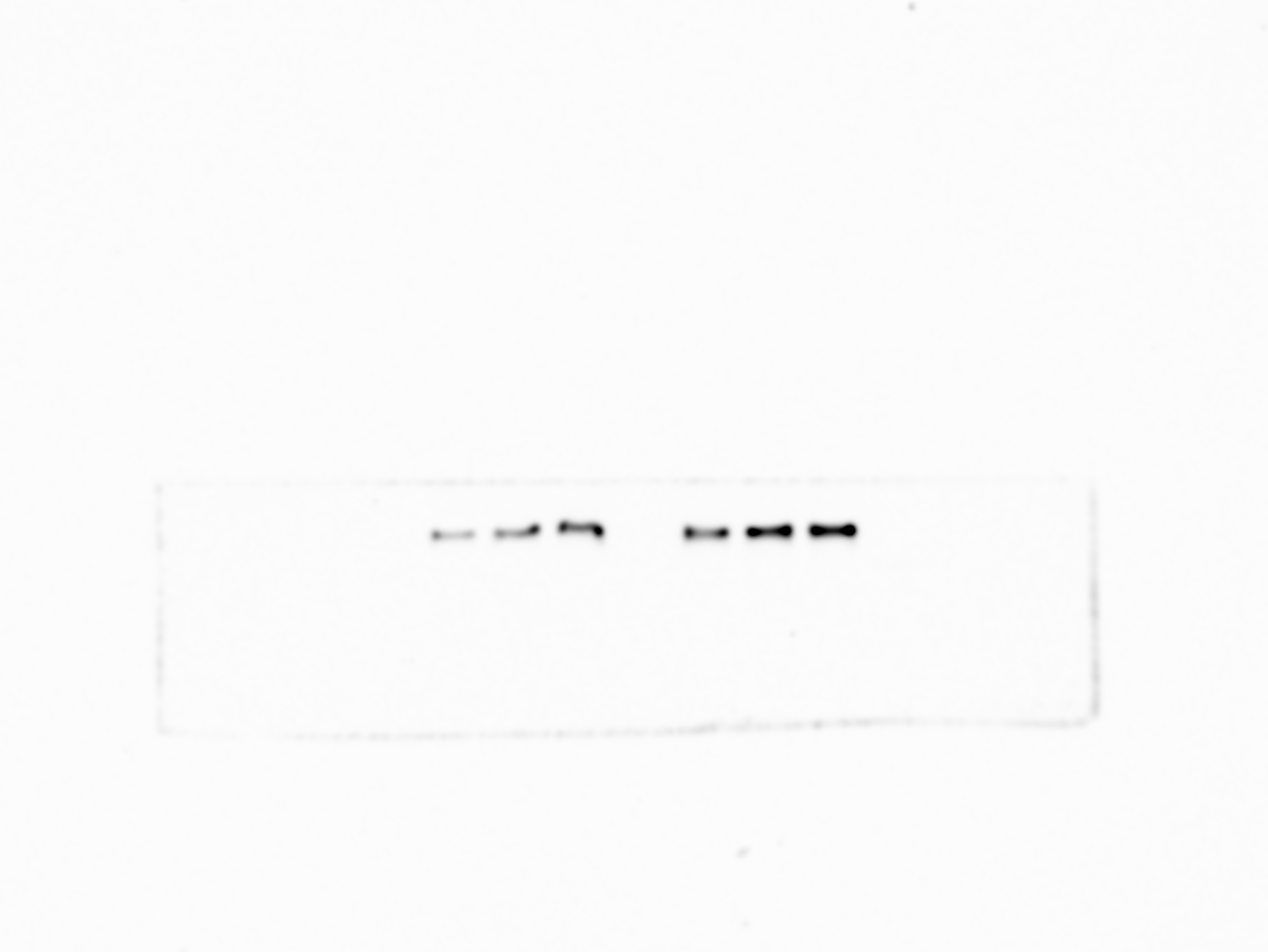

Supplement: Supplementary file 4 [file DataSheet2.zip › Fig4B p-TBK1.tif]

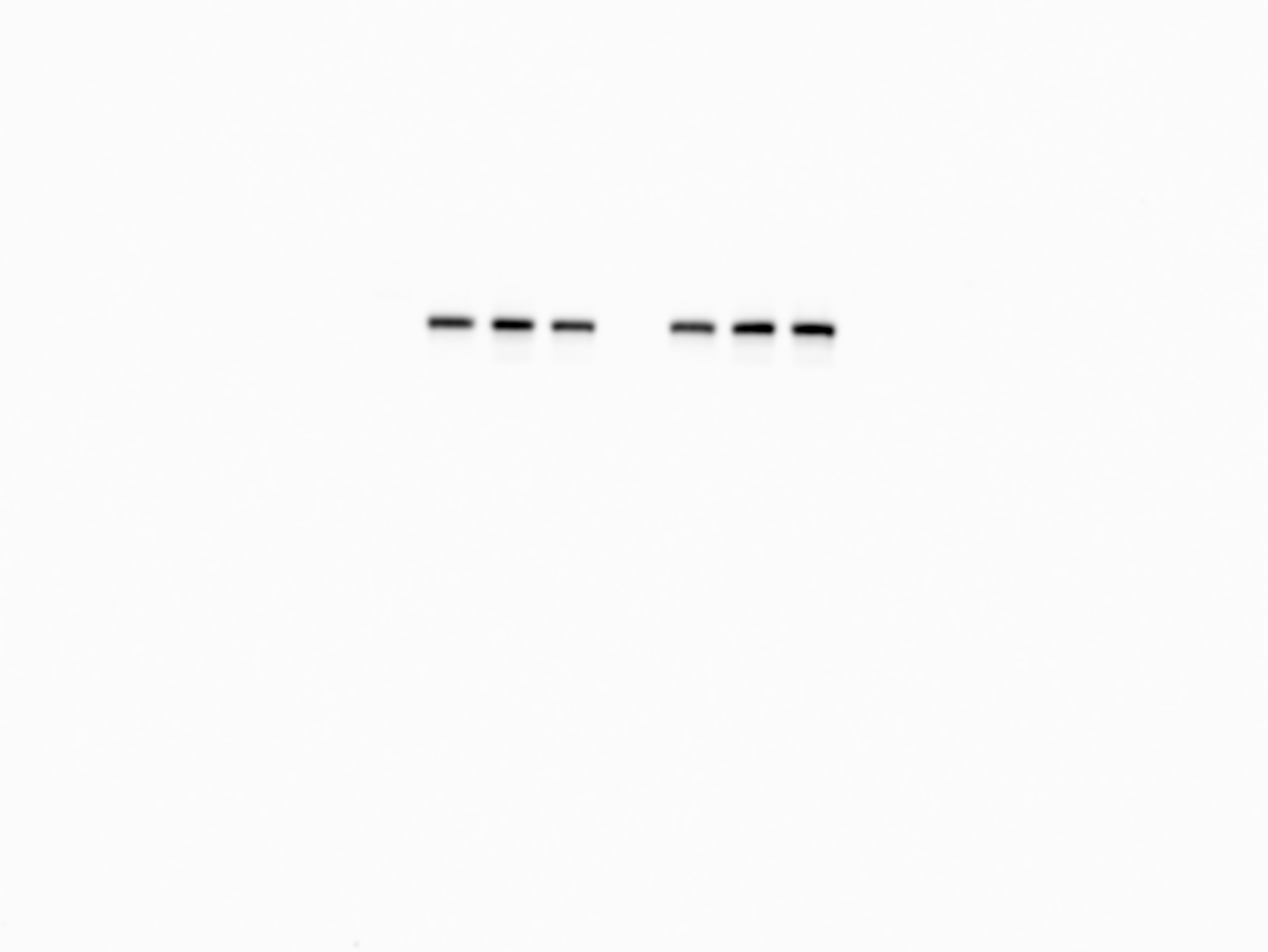

Supplement: Supplementary file 4 [file DataSheet2.zip › Fig4B TBK1.tif]

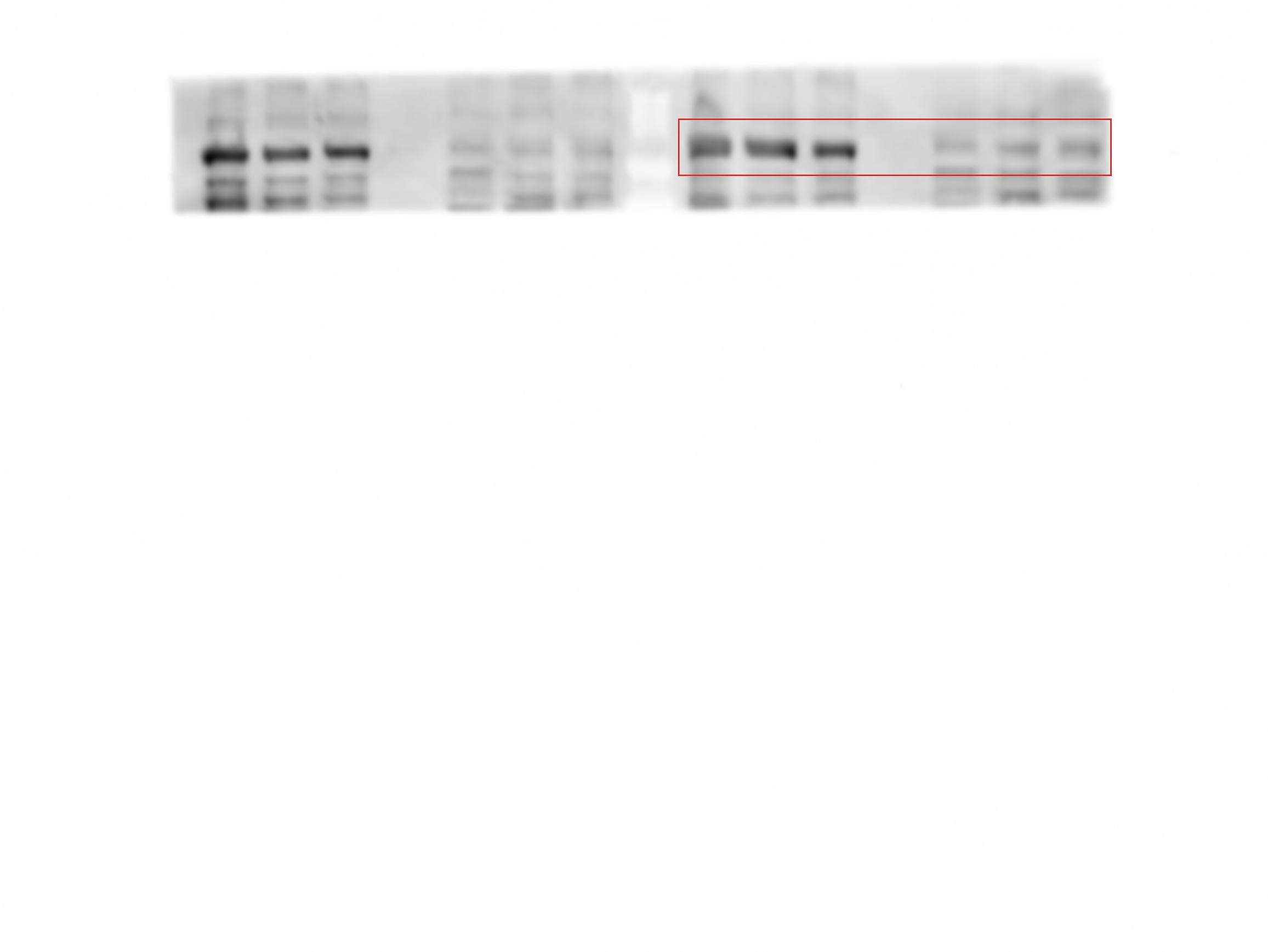

Supplement: Supplementary file 4 [file DataSheet2.zip › Fig4B TRIM28 edited showing band.jpg]

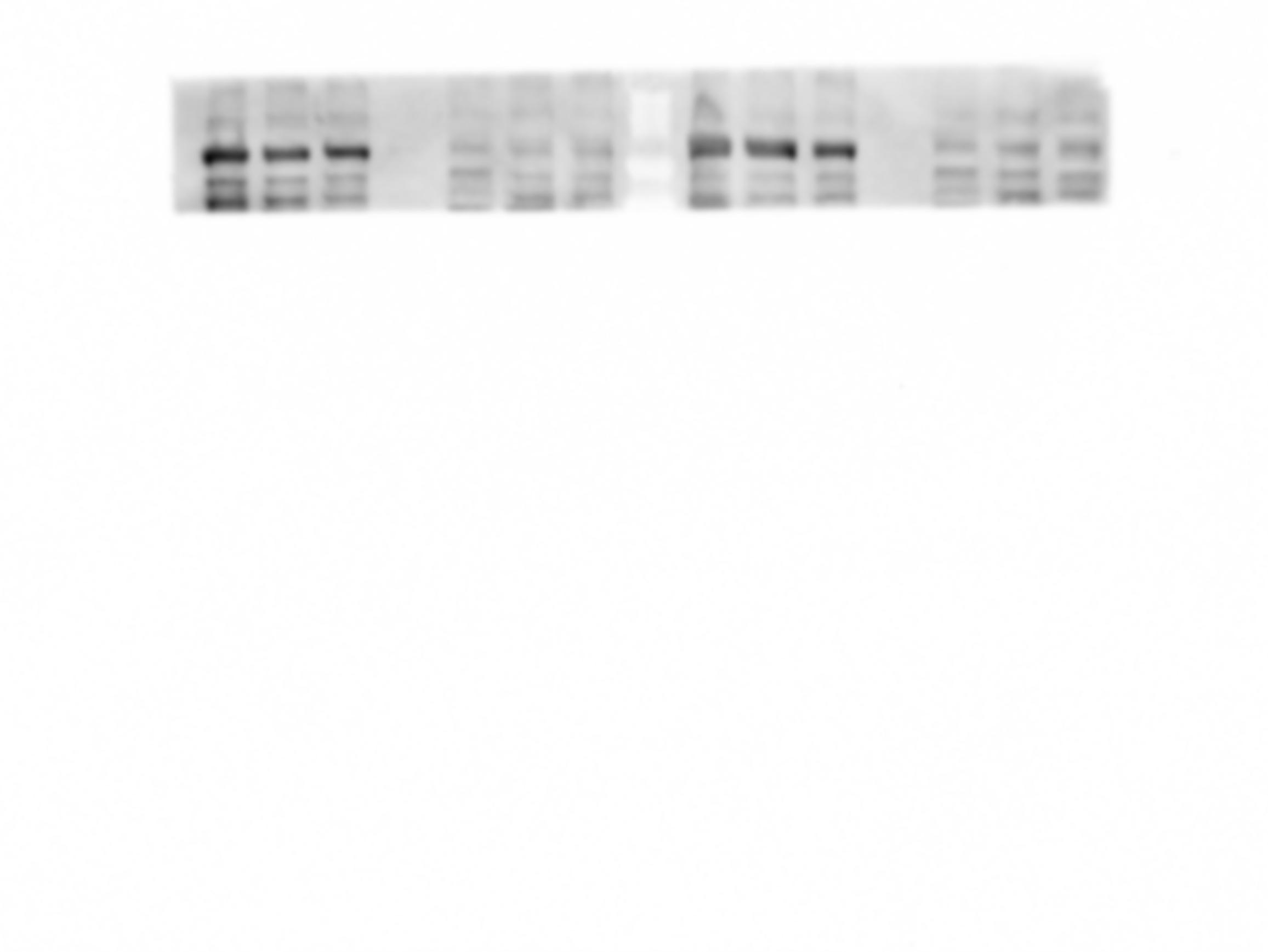

Supplement: Supplementary file 4 [file DataSheet2.zip › Fig4B TRIM28.jpg]

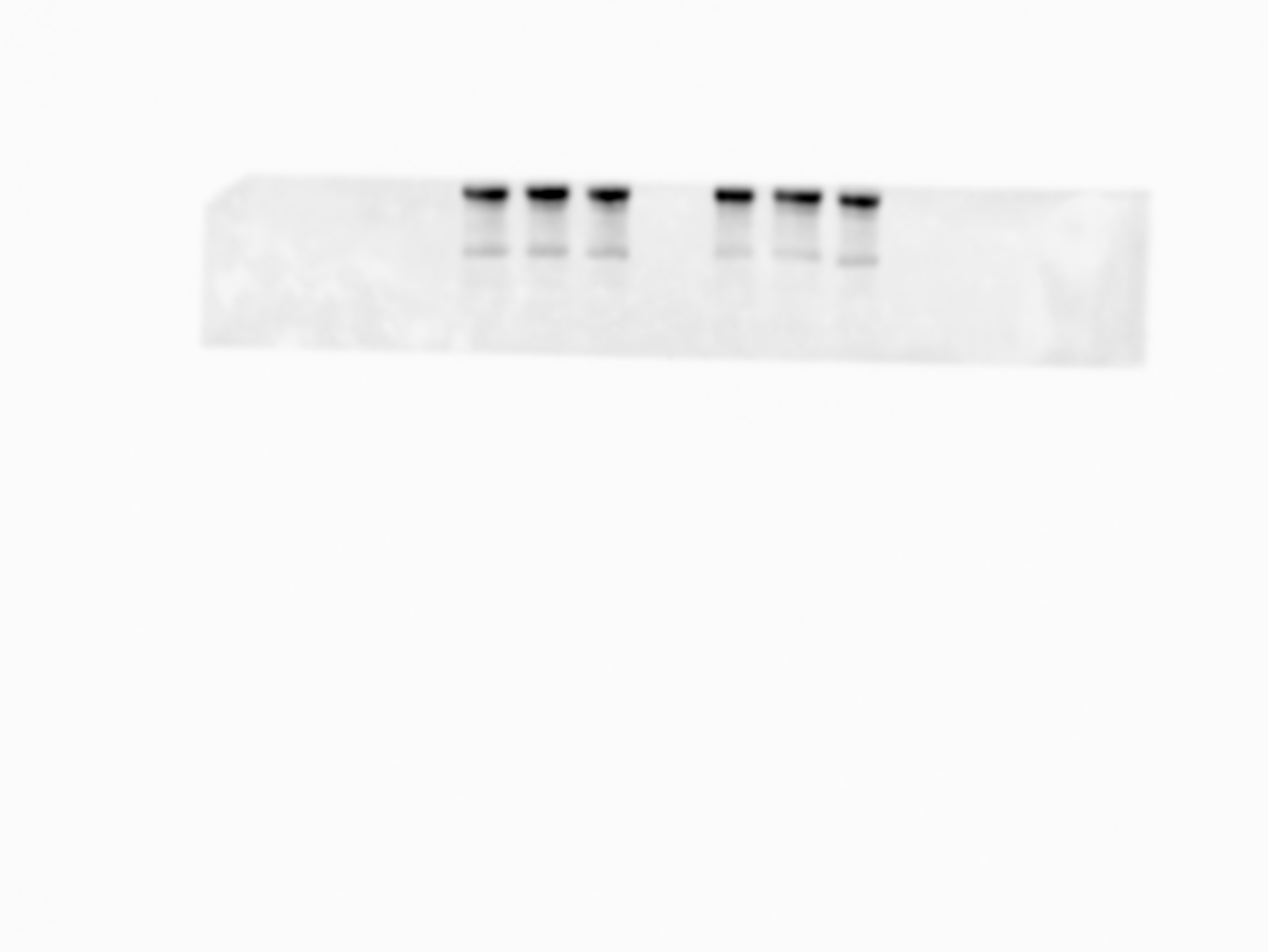

Supplement: Supplementary file 4 [file DataSheet2.zip › Fig4C GAPDH.tif]

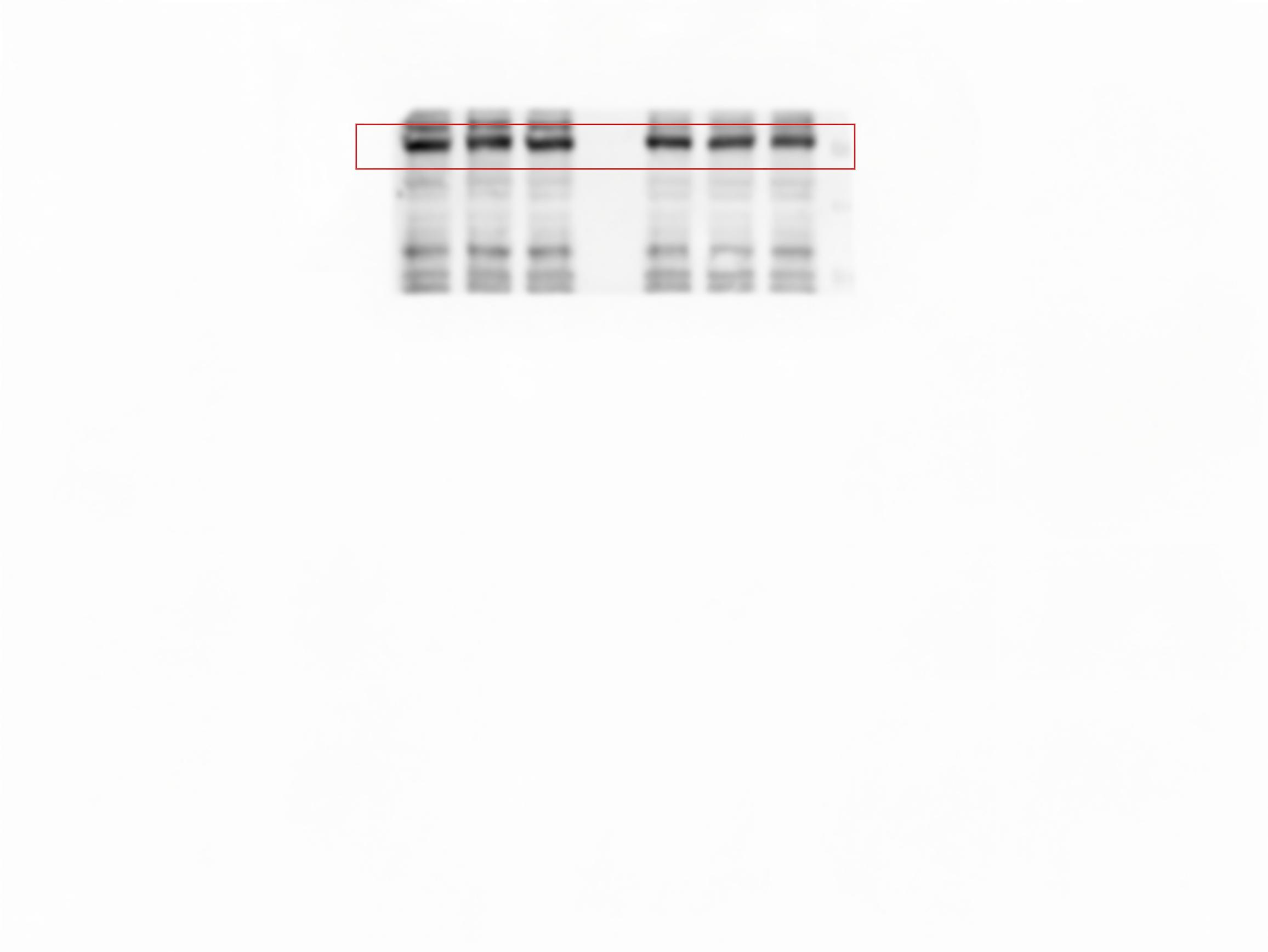

Supplement: Supplementary file 4 [file DataSheet2.zip › Fig4C IRF3 edited showing band.jpg]

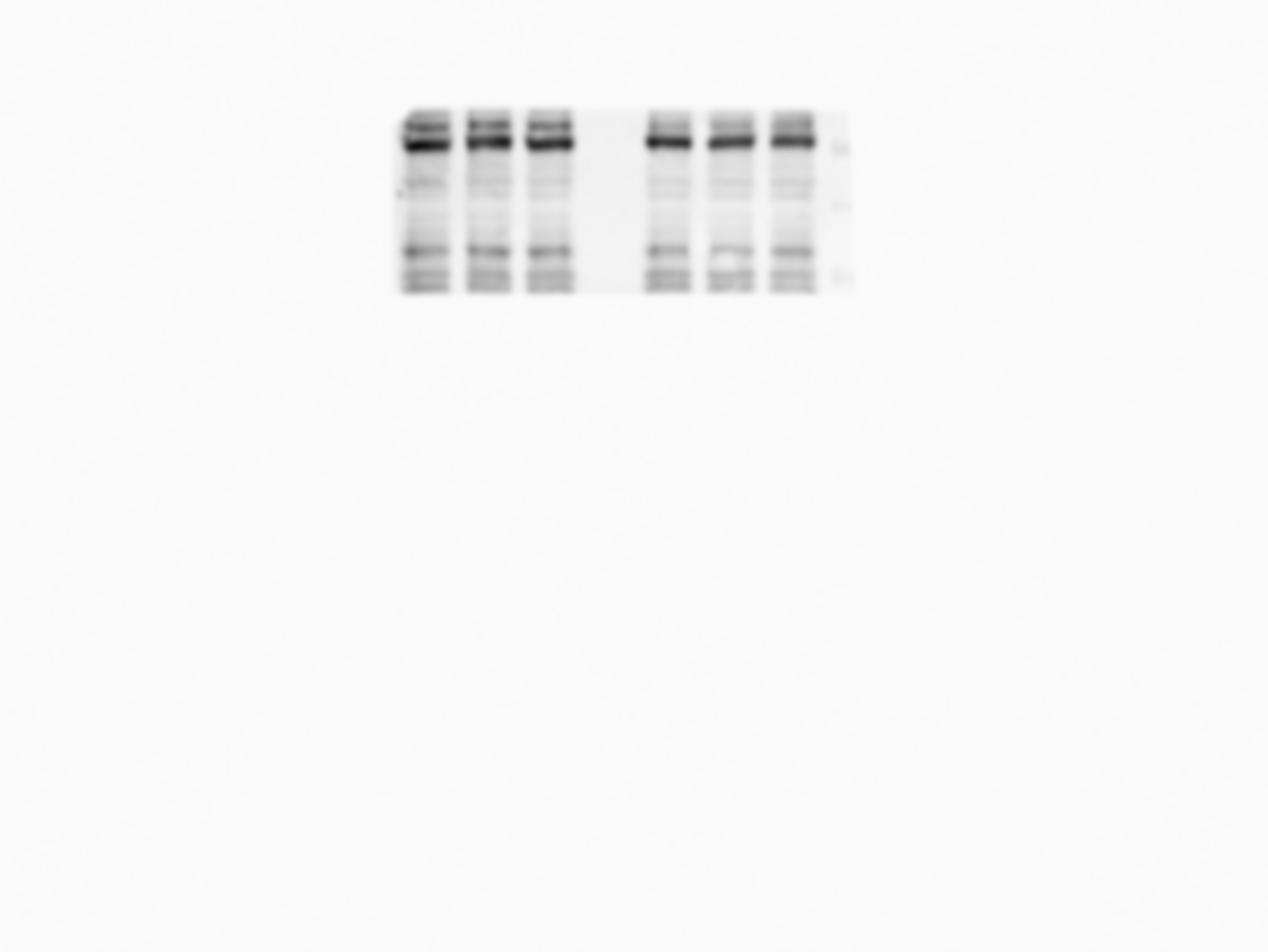

Supplement: Supplementary file 4 [file DataSheet2.zip › Fig4C IRF3.tif]

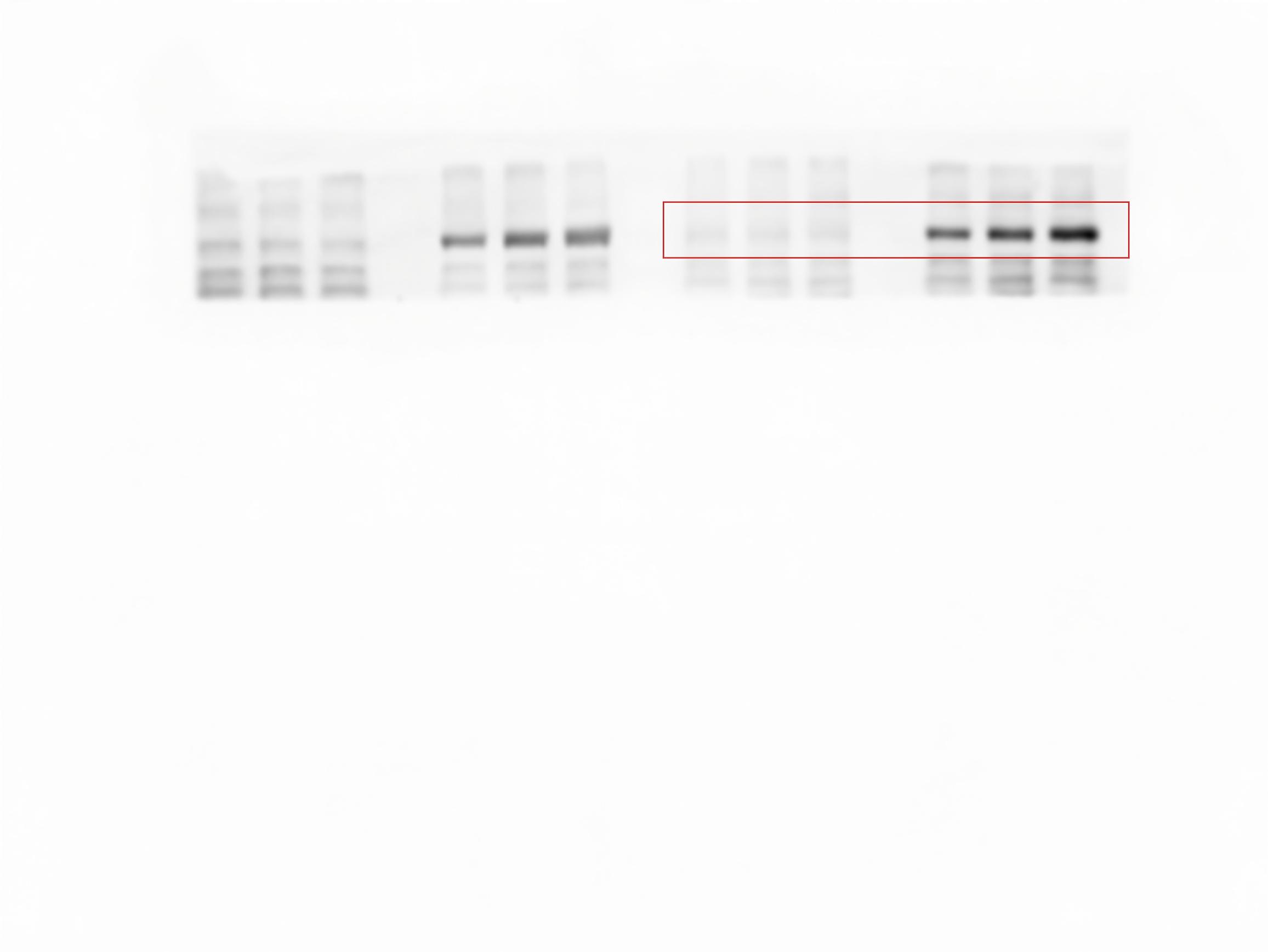

Supplement: Supplementary file 4 [file DataSheet2.zip › Fig4C Myc edited showing band.jpg]

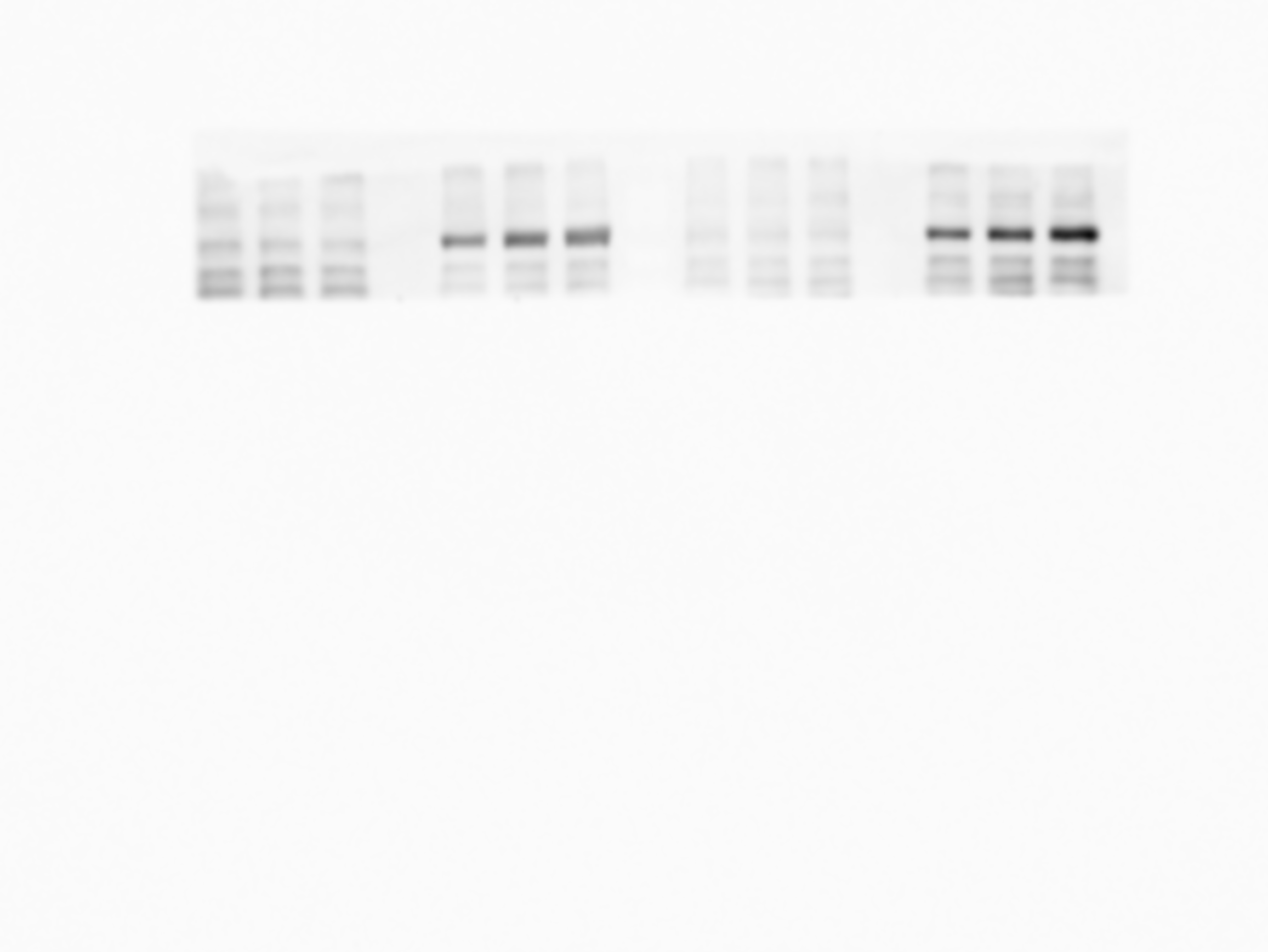

Supplement: Supplementary file 4 [file DataSheet2.zip › Fig4C Myc.tif]

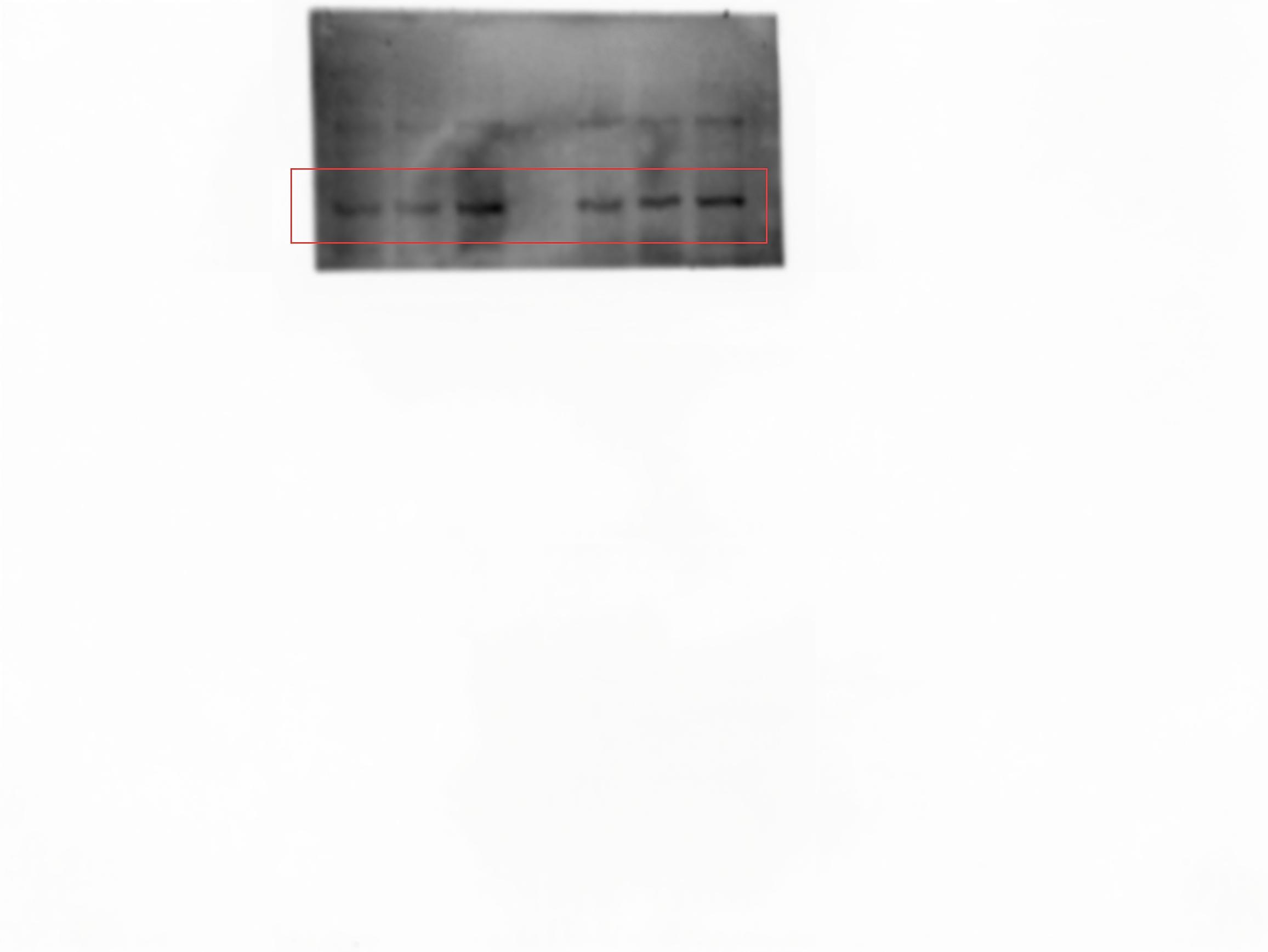

Supplement: Supplementary file 4 [file DataSheet2.zip › Fig4C p-IRF3 edited showing band.jpg]

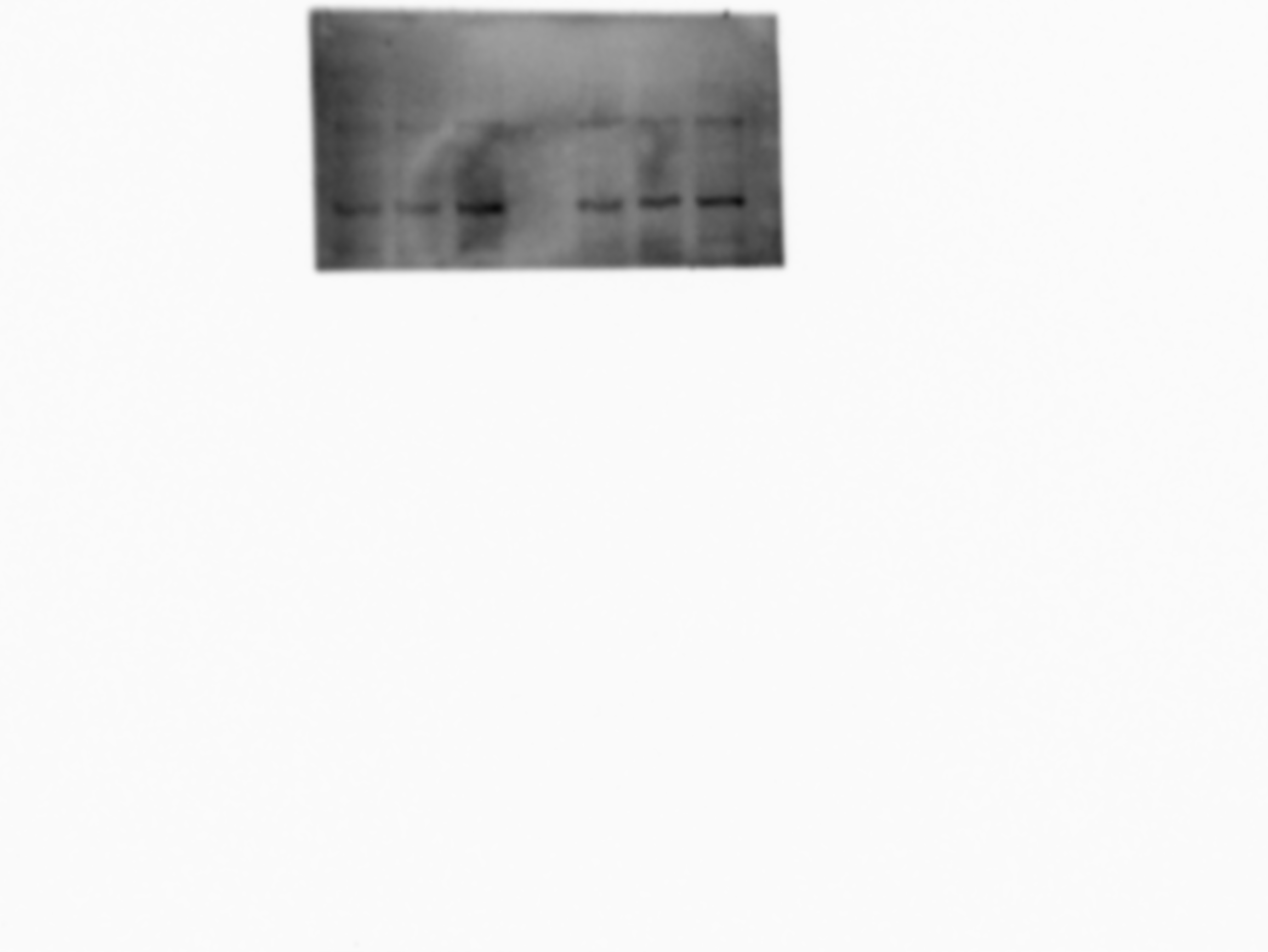

Supplement: Supplementary file 4 [file DataSheet2.zip › Fig4C p-IRF3.tif]

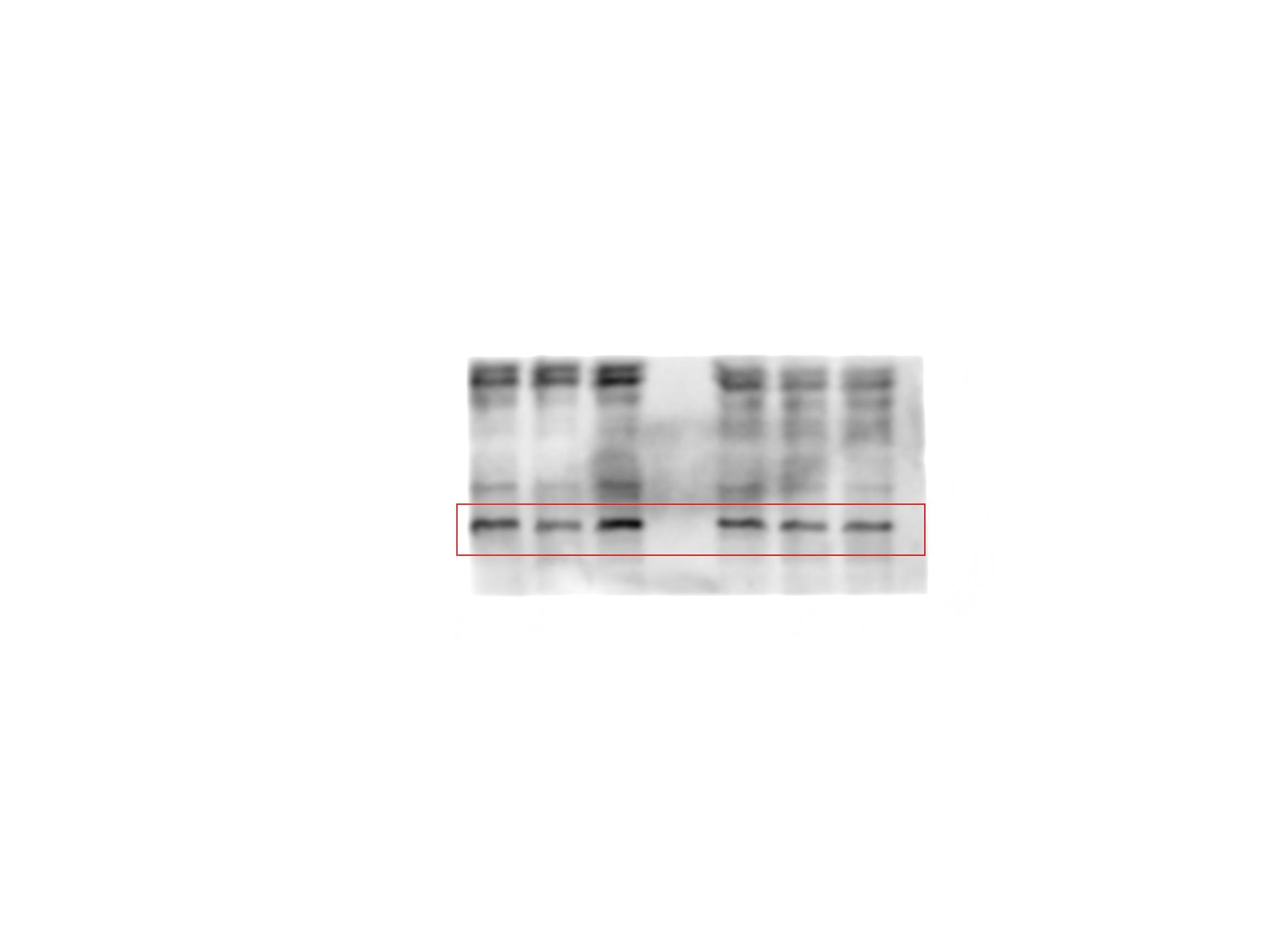

Supplement: Supplementary file 4 [file DataSheet2.zip › Fig4C p-TBK1 edited showing band.jpg]

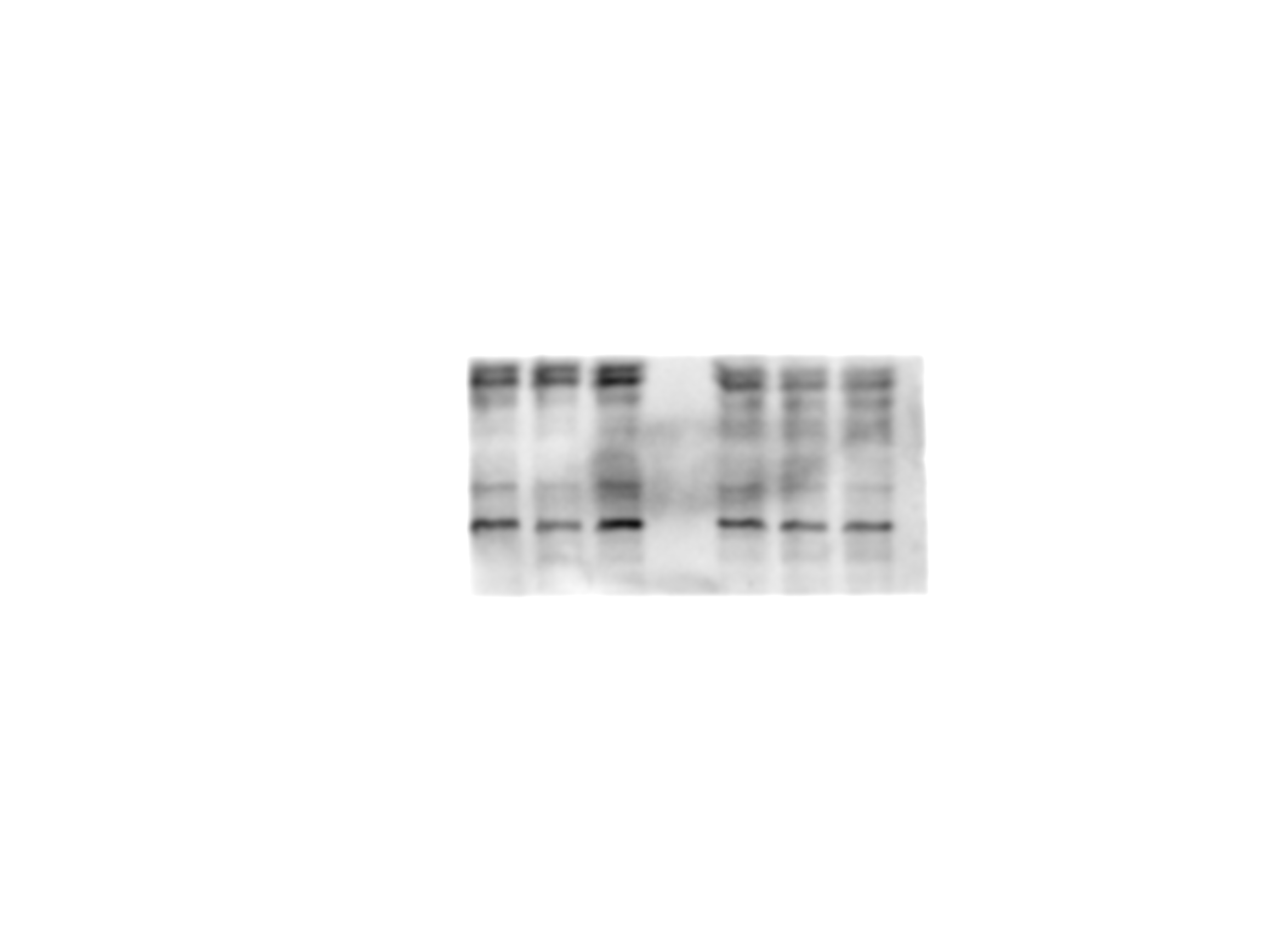

Supplement: Supplementary file 4 [file DataSheet2.zip › Fig4C p-TBK1.tif]

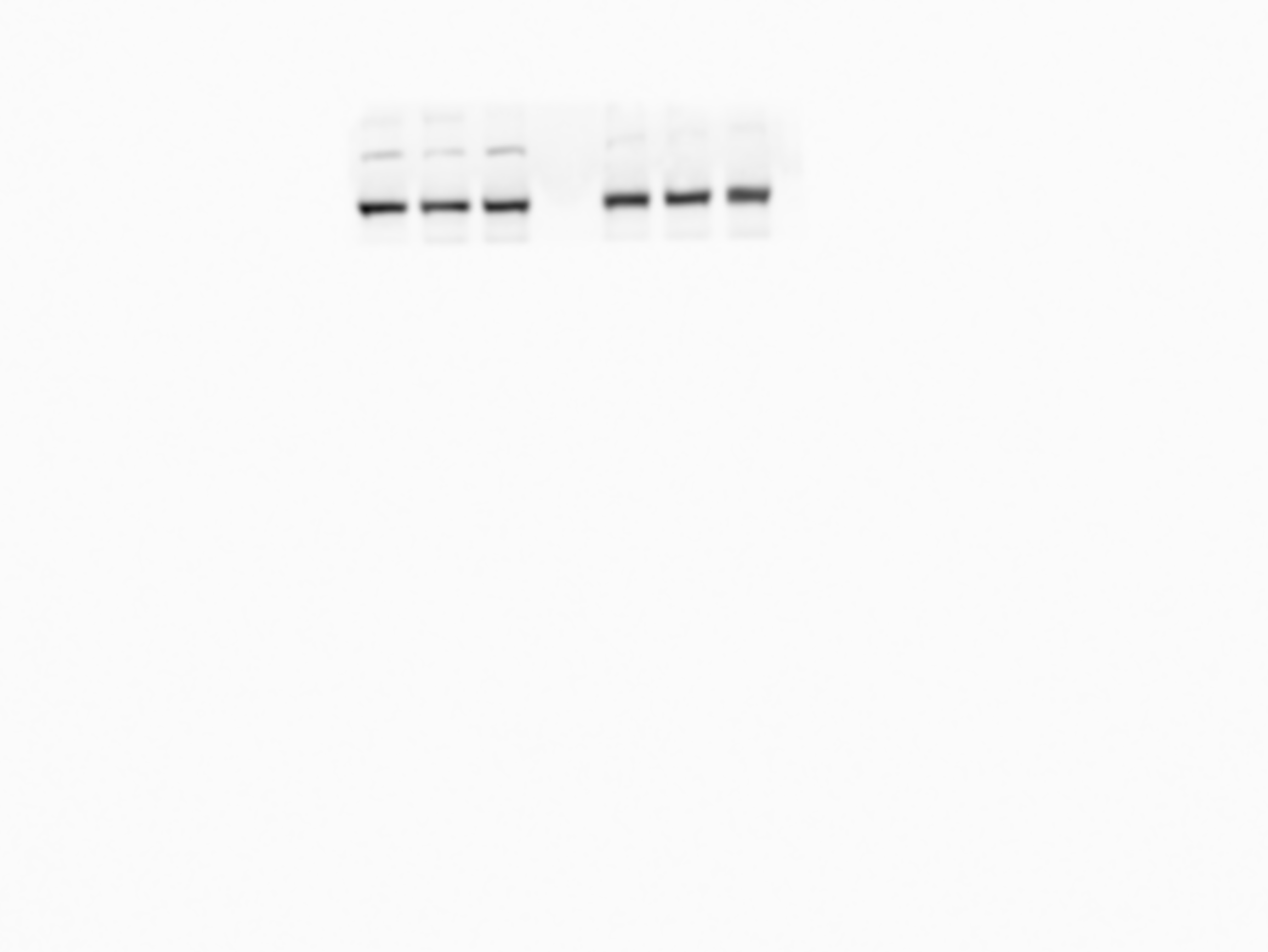

Supplement: Supplementary file 4 [file DataSheet2.zip › Fig4C TBK1.tif]

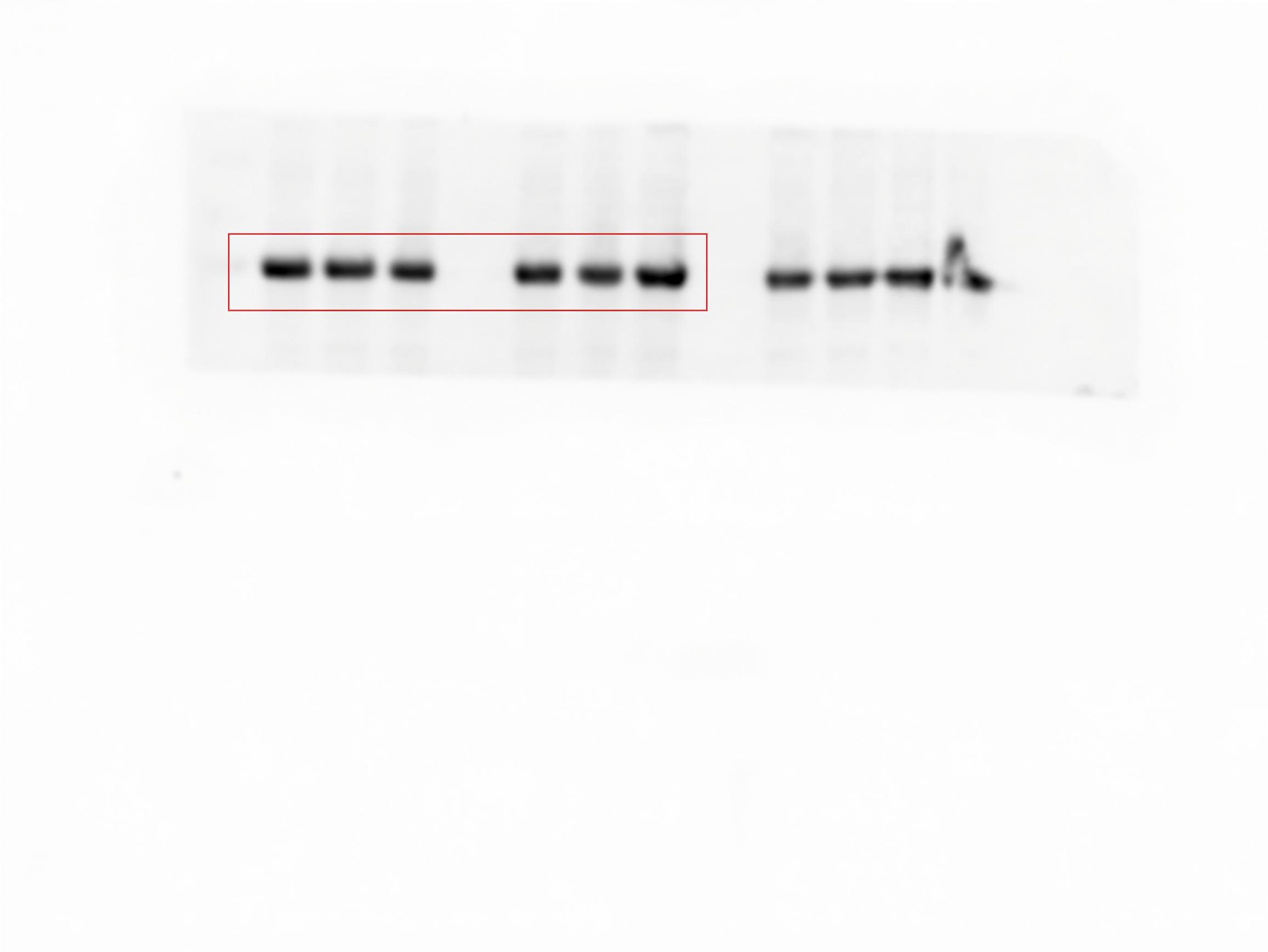

Supplement: Supplementary file 4 [file DataSheet2.zip › Fig4D Actin edited showing band.jpg]

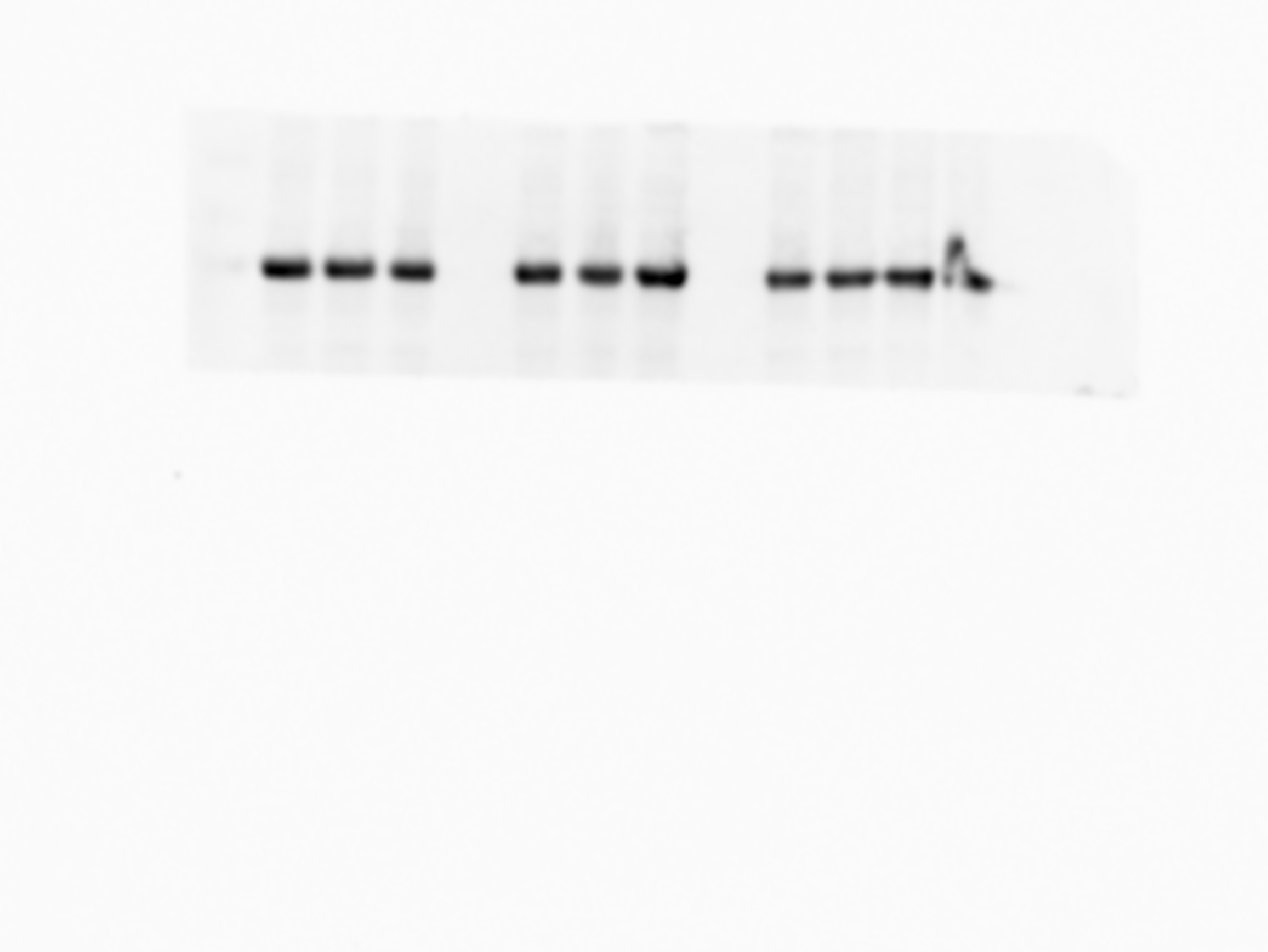

Supplement: Supplementary file 4 [file DataSheet2.zip › Fig4D Actin.tif]

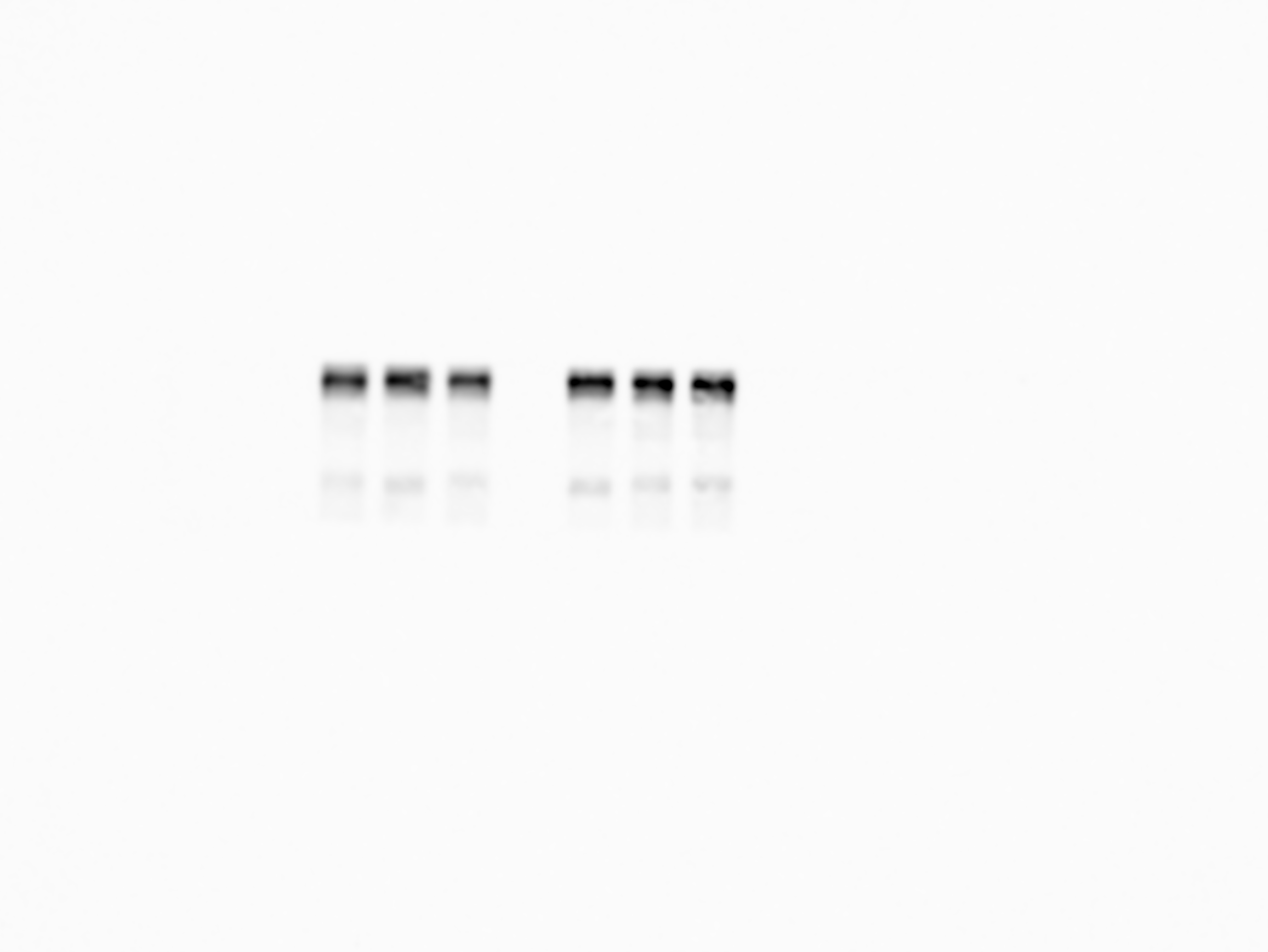

Supplement: Supplementary file 4 [file DataSheet2.zip › Fig4D IRF3.tif]

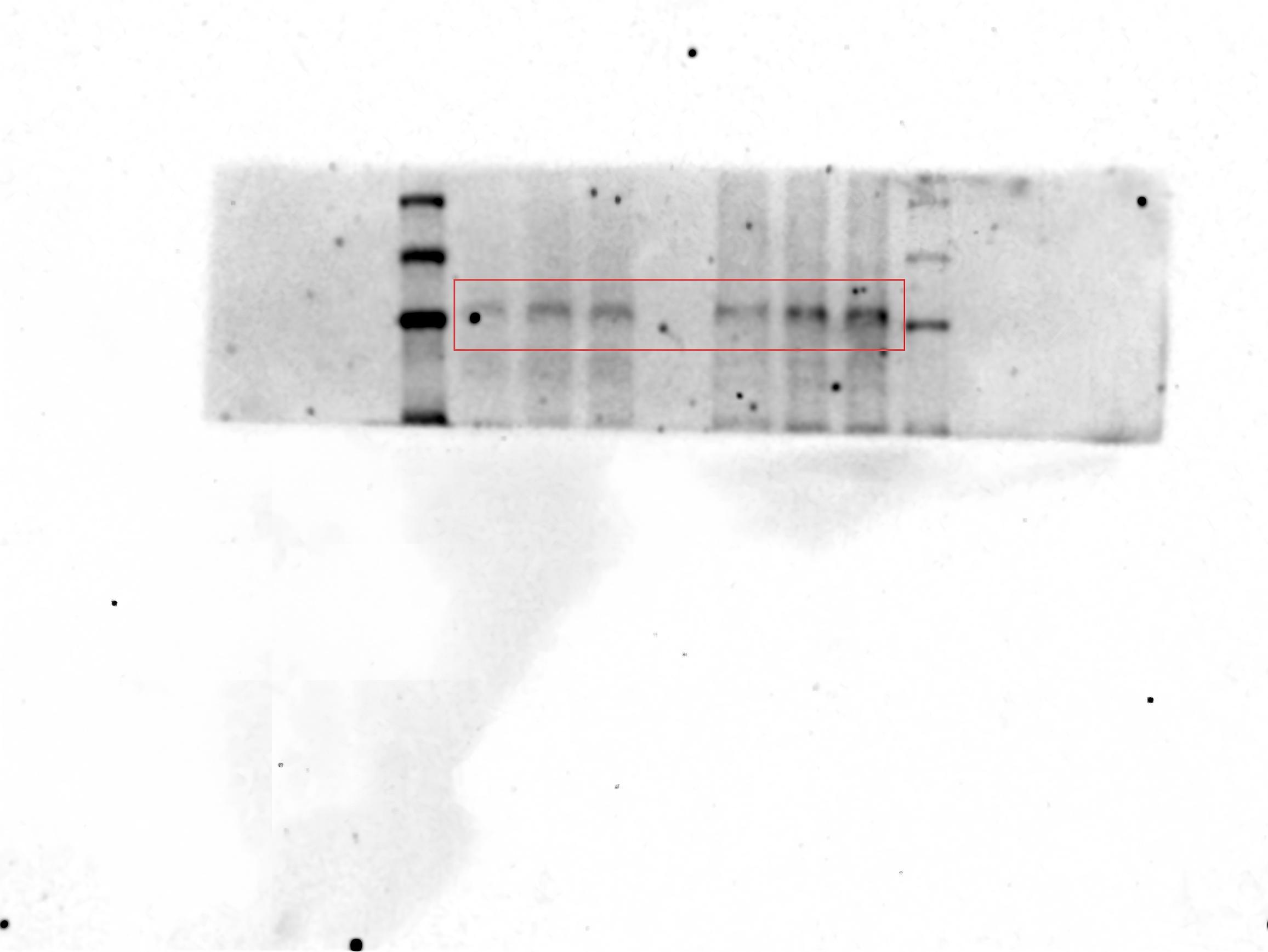

Supplement: Supplementary file 4 [file DataSheet2.zip › Fig4D p-IRF3 edited showing band.jpg]

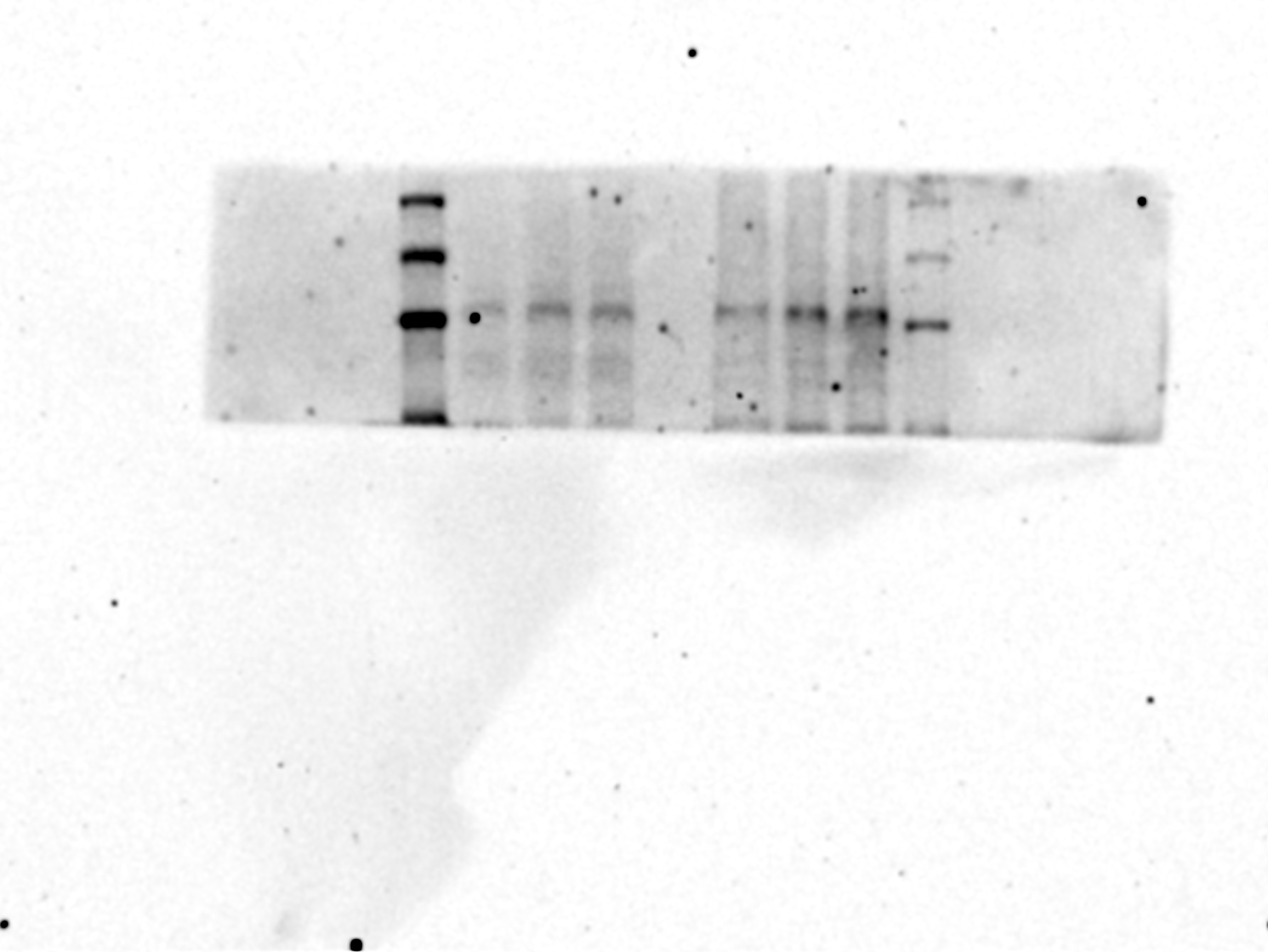

Supplement: Supplementary file 4 [file DataSheet2.zip › Fig4D p-IRF3.tif]

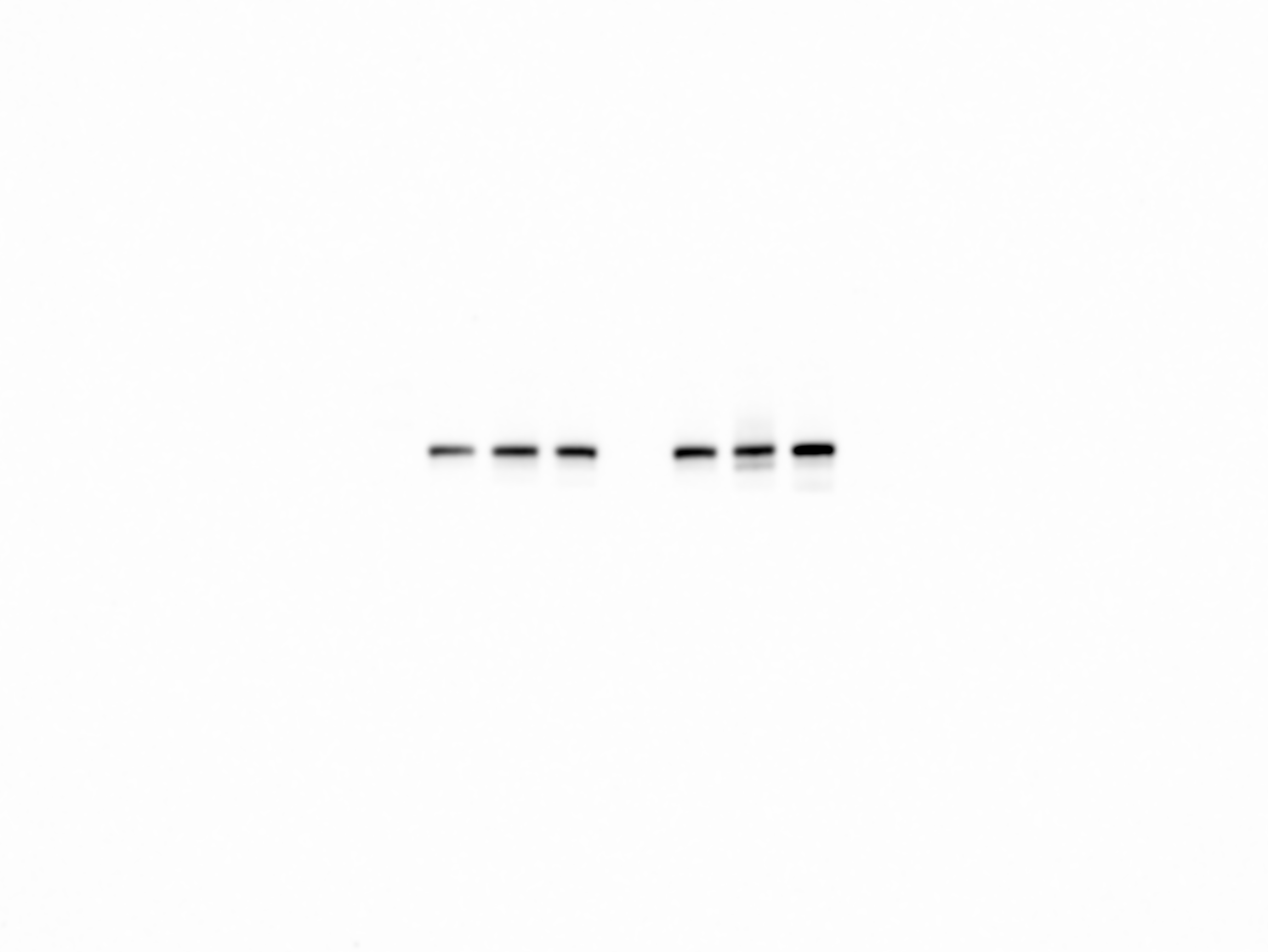

Supplement: Supplementary file 4 [file DataSheet2.zip › Fig4D p-TBK1.tif]

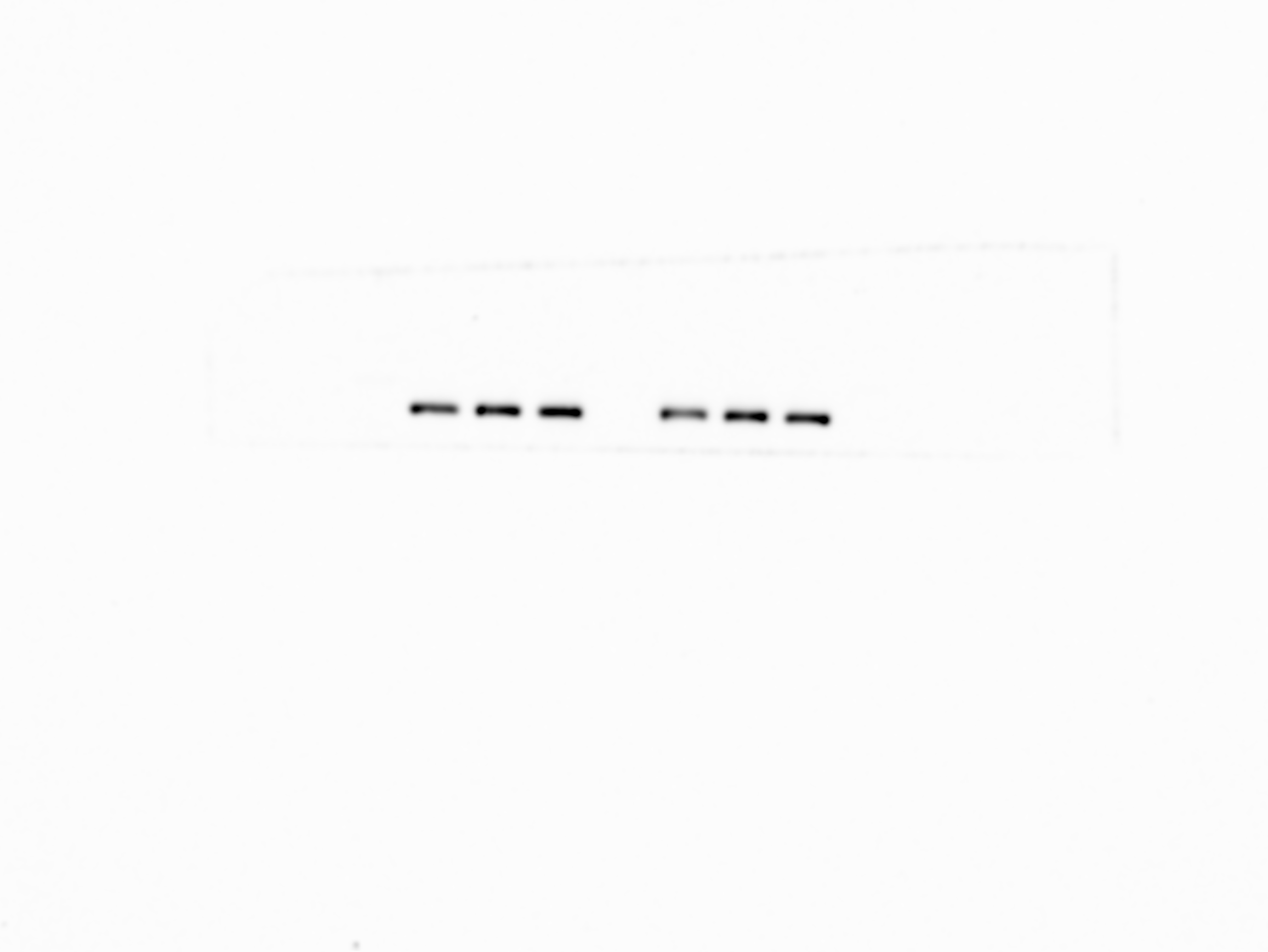

Supplement: Supplementary file 4 [file DataSheet2.zip › Fig4D TBK1.tif]

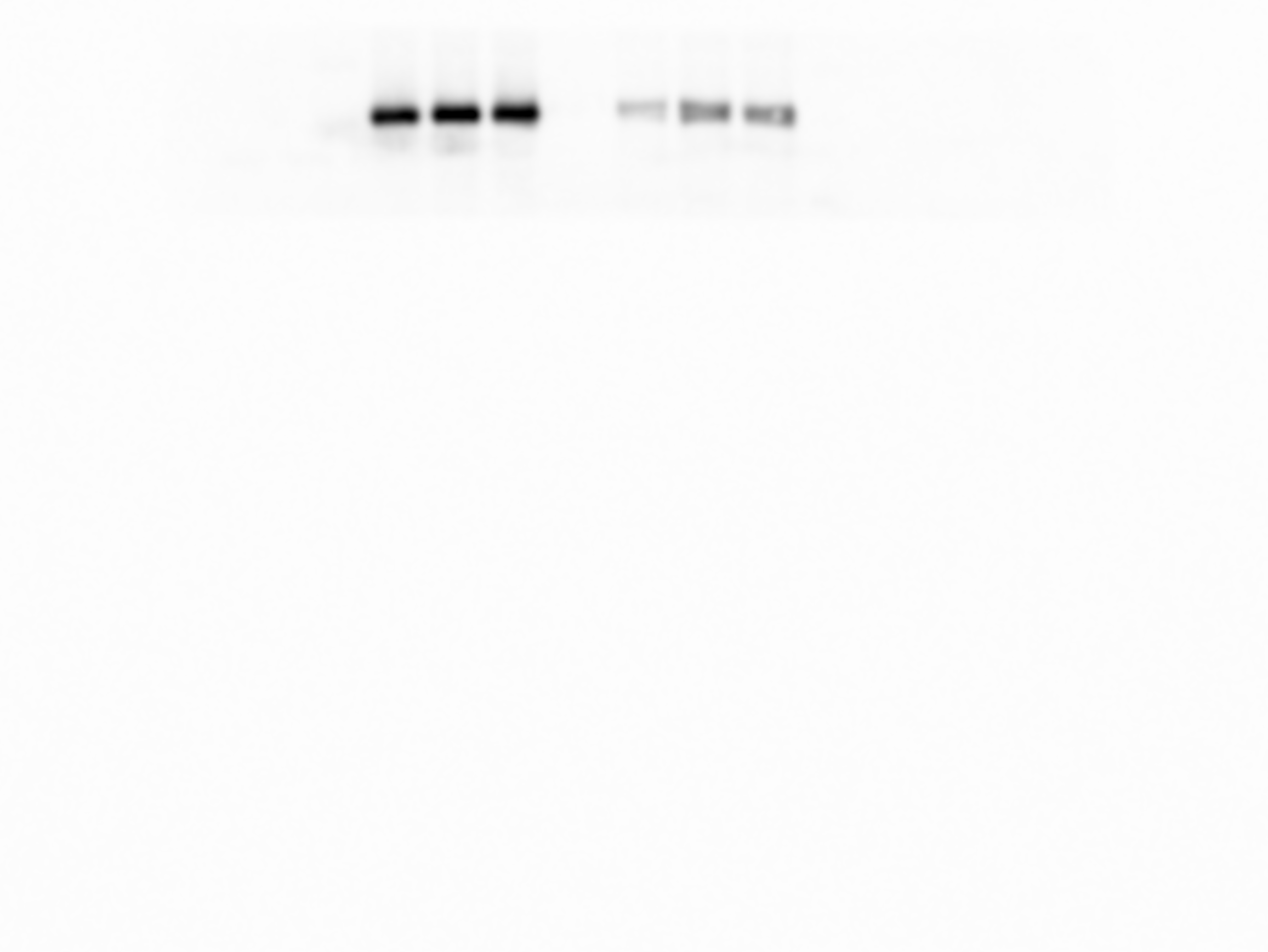

Supplement: Supplementary file 4 [file DataSheet2.zip › Fig4D TRIM28.tif]

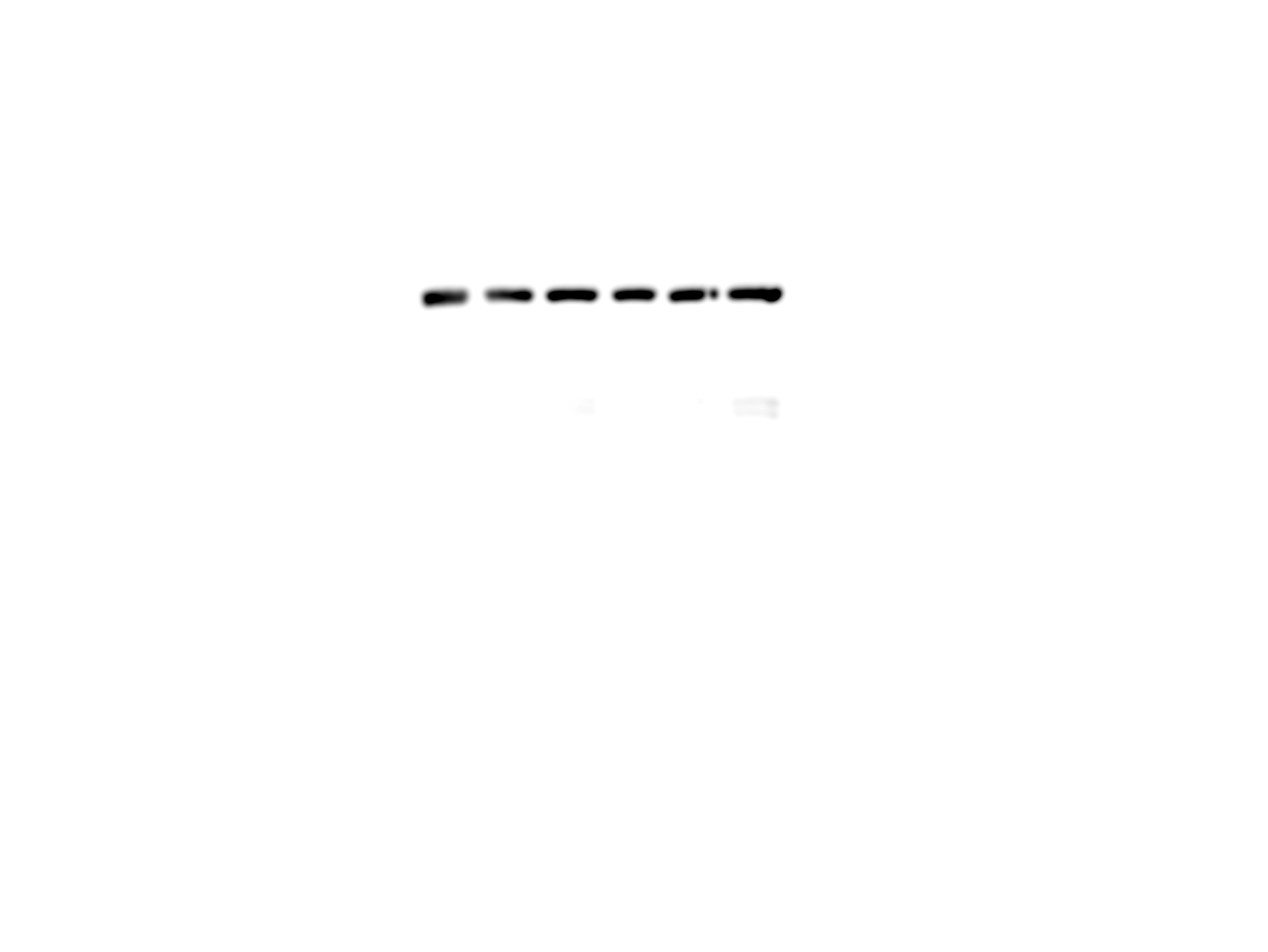

Supplement: Supplementary file 4 [file DataSheet2.zip › Fig4E CE GAPDH.tif]

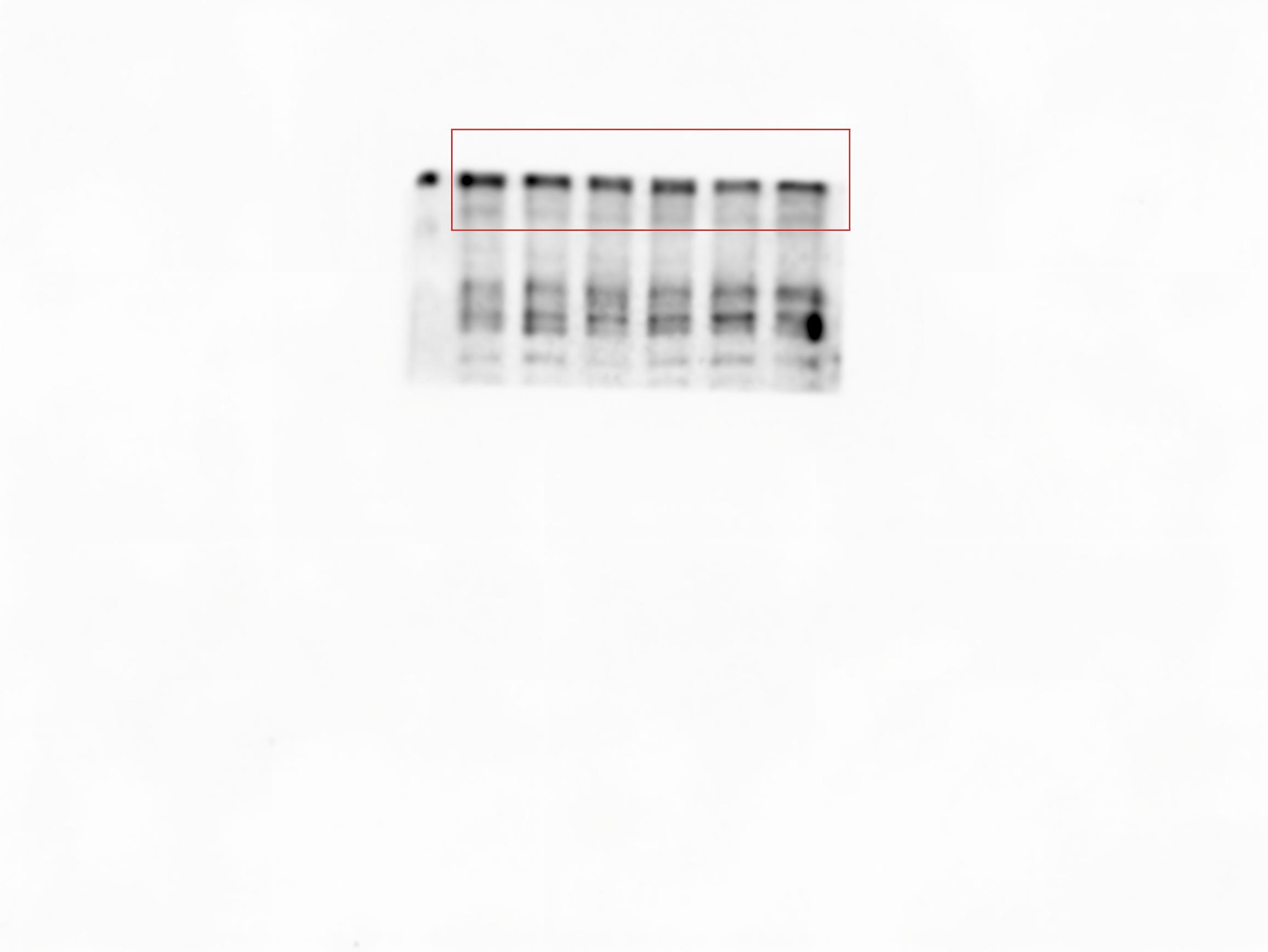

Supplement: Supplementary file 4 [file DataSheet2.zip › Fig4E CE IRF3 edited showing band.jpg]

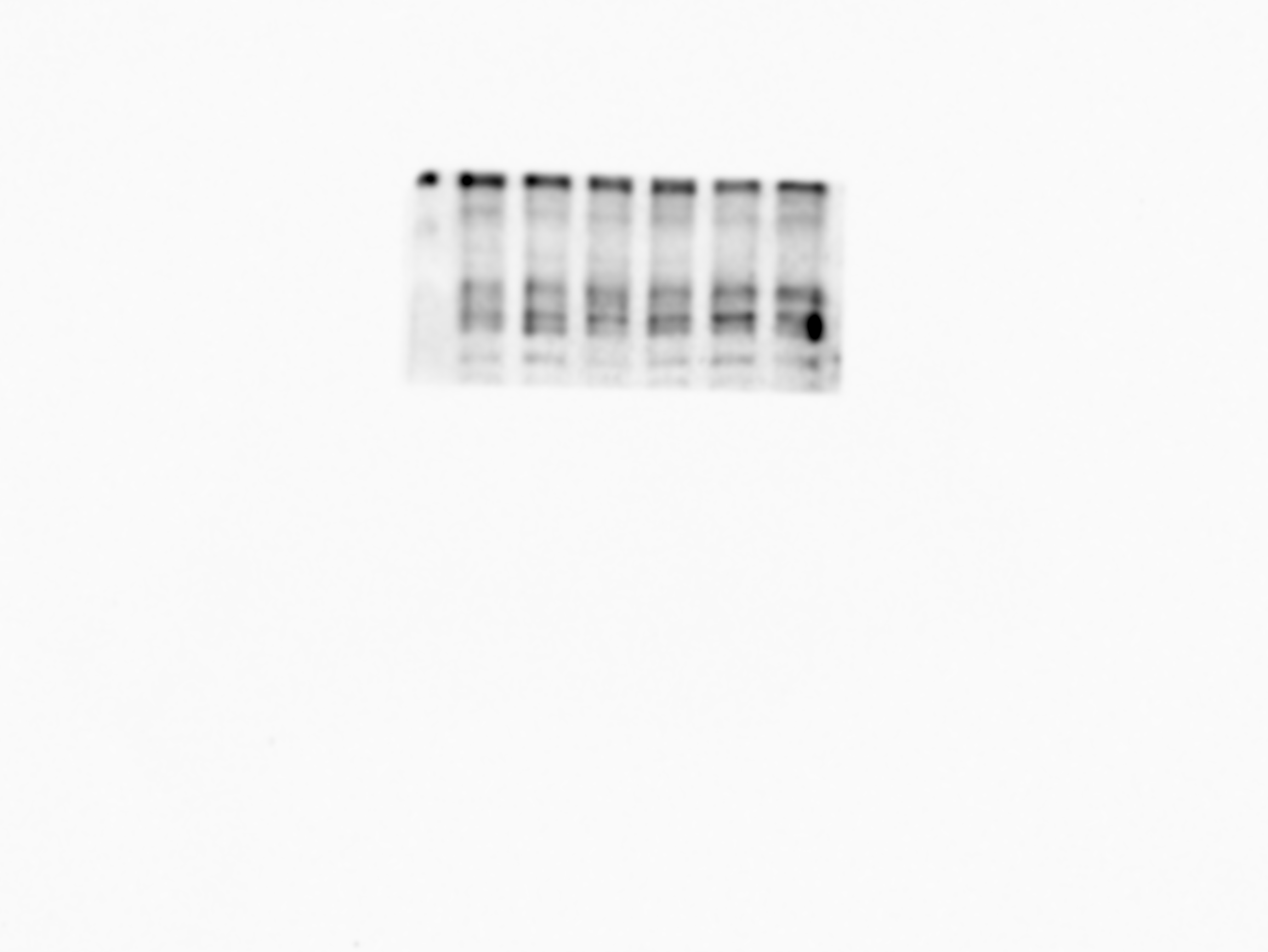

Supplement: Supplementary file 4 [file DataSheet2.zip › Fig4E CE IRF3.tif]

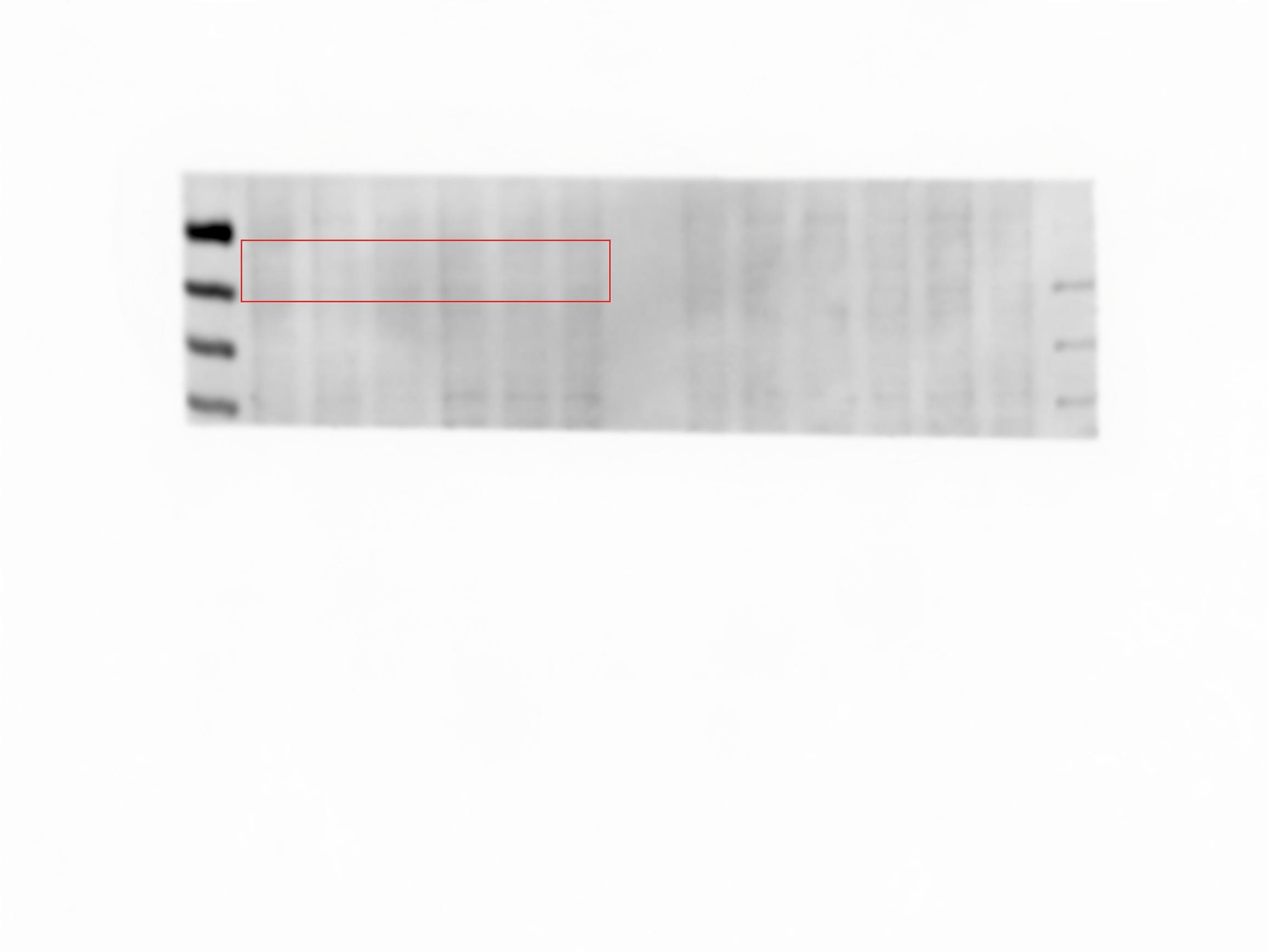

Supplement: Supplementary file 4 [file DataSheet2.zip › Fig4E CE Lamin B edited showing band.jpg]

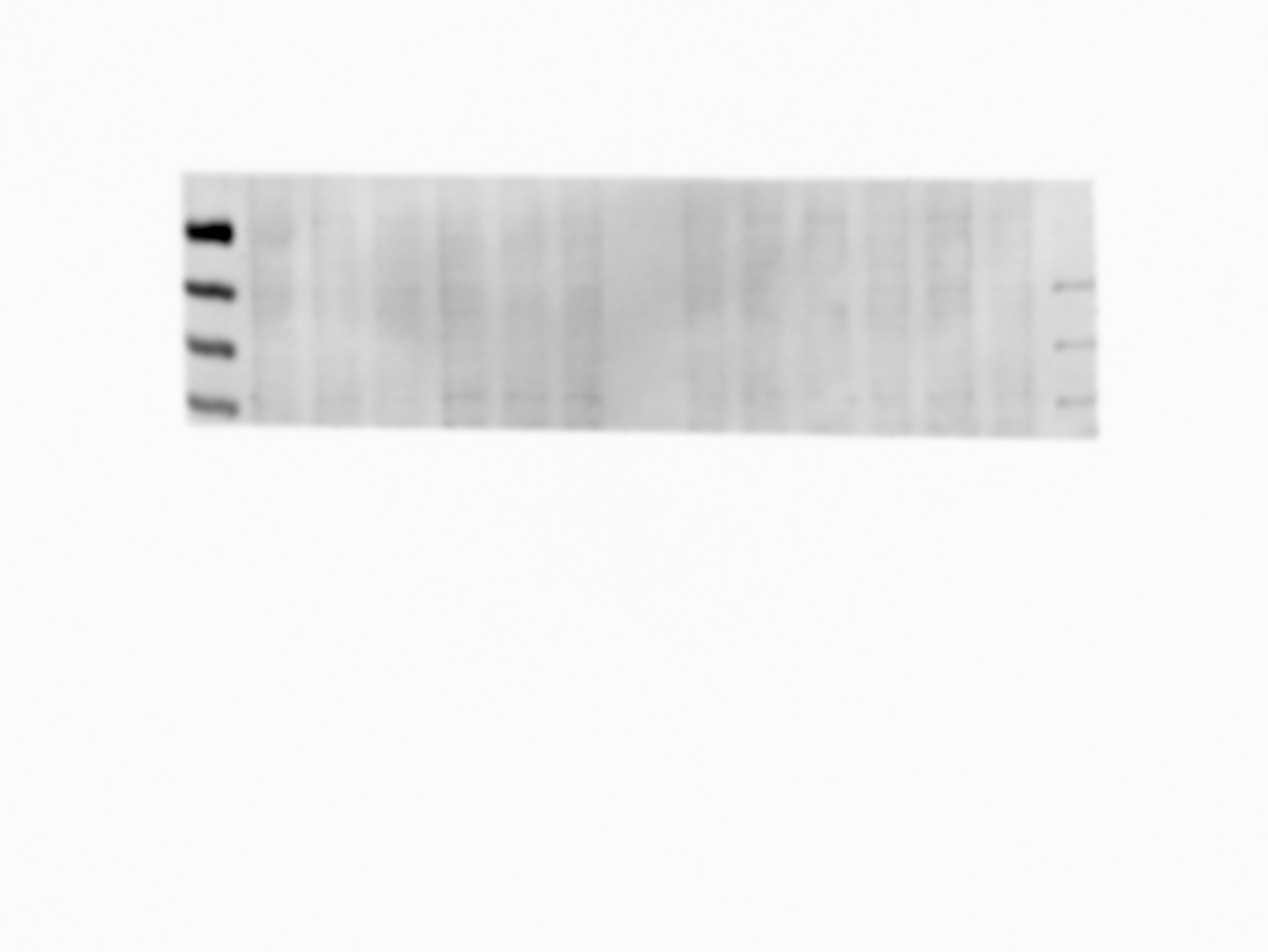

Supplement: Supplementary file 4 [file DataSheet2.zip › Fig4E CE Lamin B.tif]

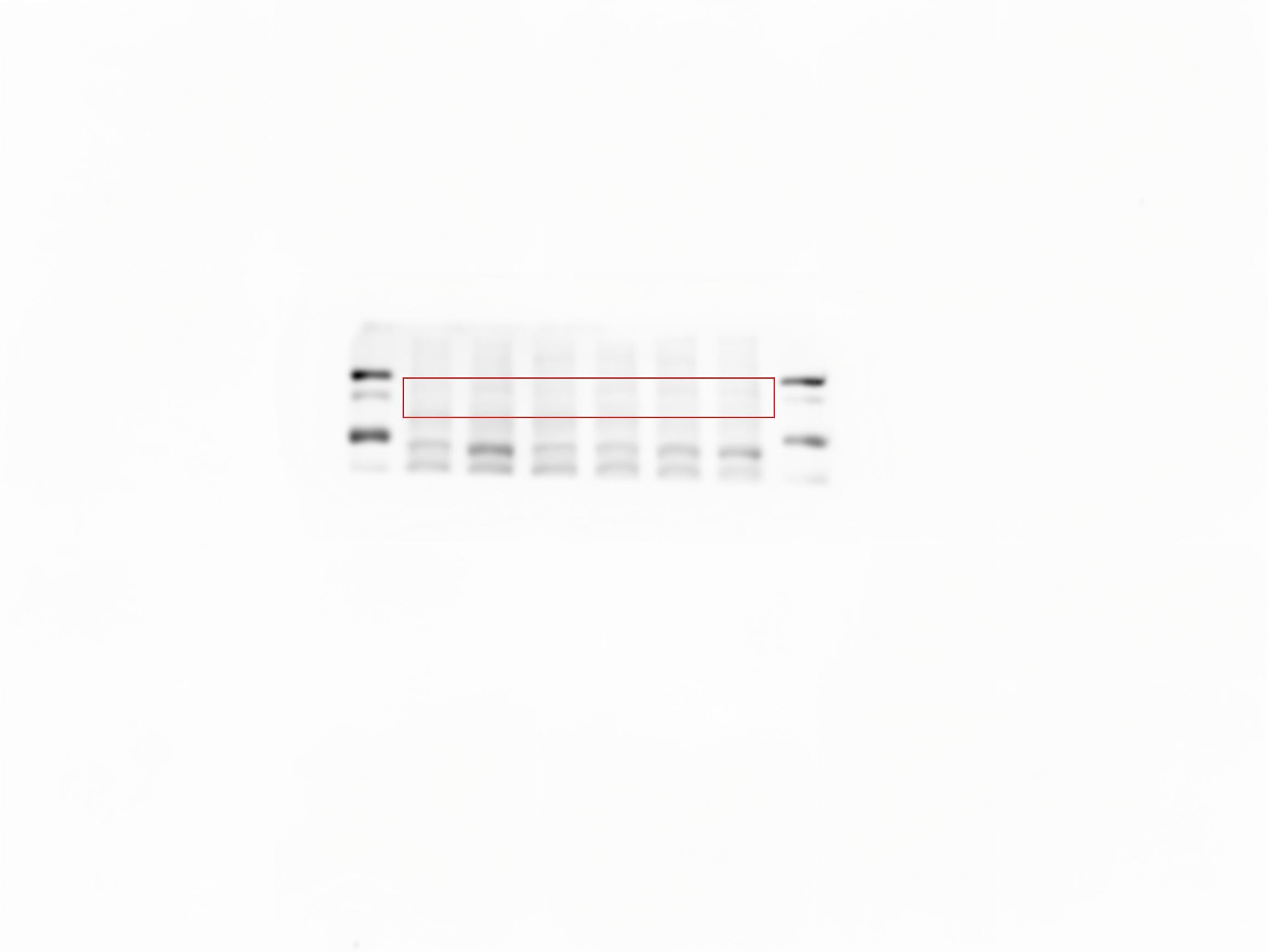

Supplement: Supplementary file 4 [file DataSheet2.zip › Fig4E NE GAPDH edited showing band.jpg]

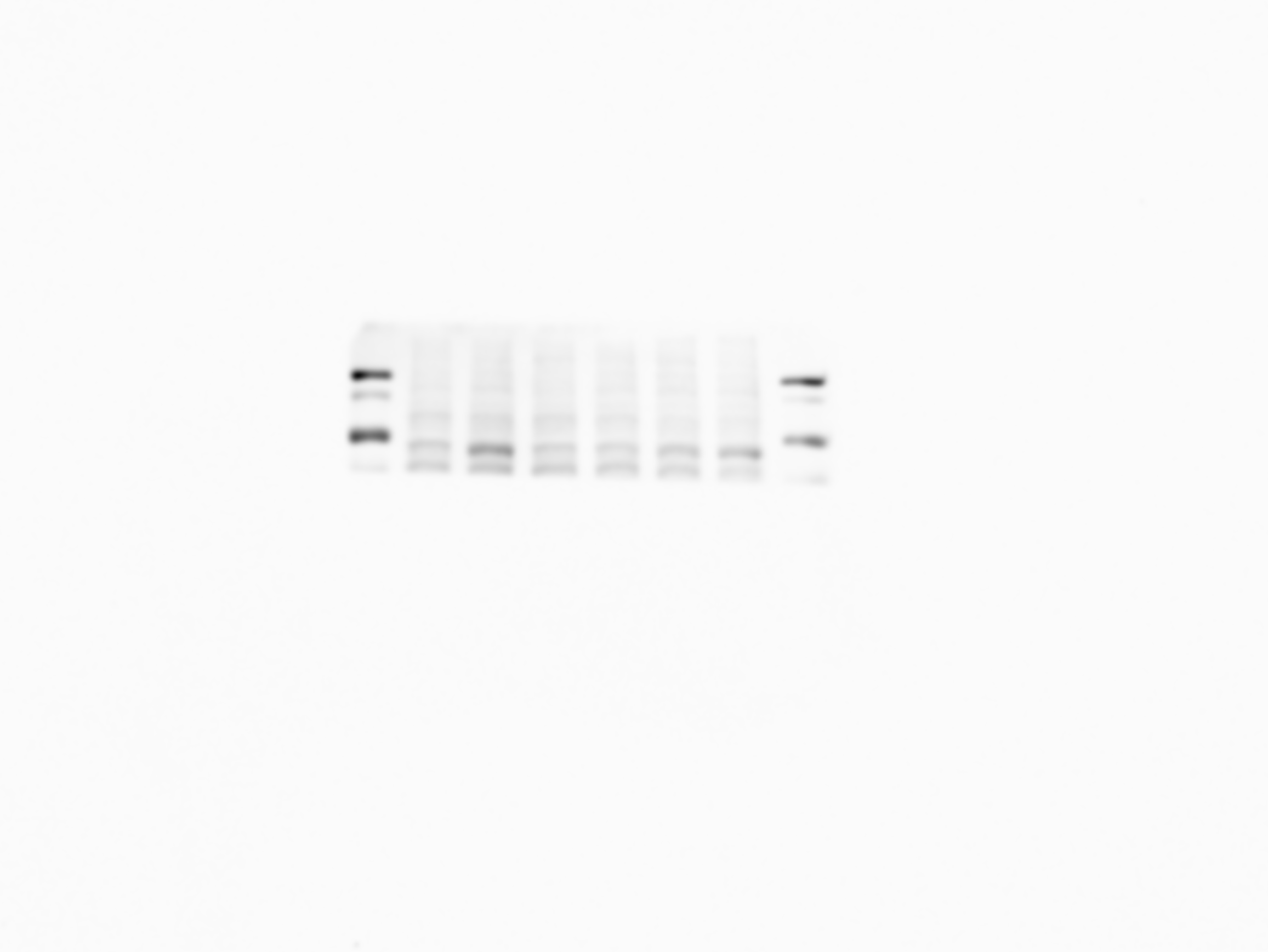

Supplement: Supplementary file 4 [file DataSheet2.zip › Fig4E NE GAPDH.tif]
