# Supplementary figures and images for: CRM1-dependent nuclear export of TRIM28 promotes MAVS K48-linked ubiquitination and suppresses RIG-I-mediated antiviral response (part 2 of 3)
Source: Front Immunol. 2026 Mar 24;17:1744833. doi: 10.3389/fimmu.2026.1744833 (PMC13053320; doi:10.3389/fimmu.2026.1744833)

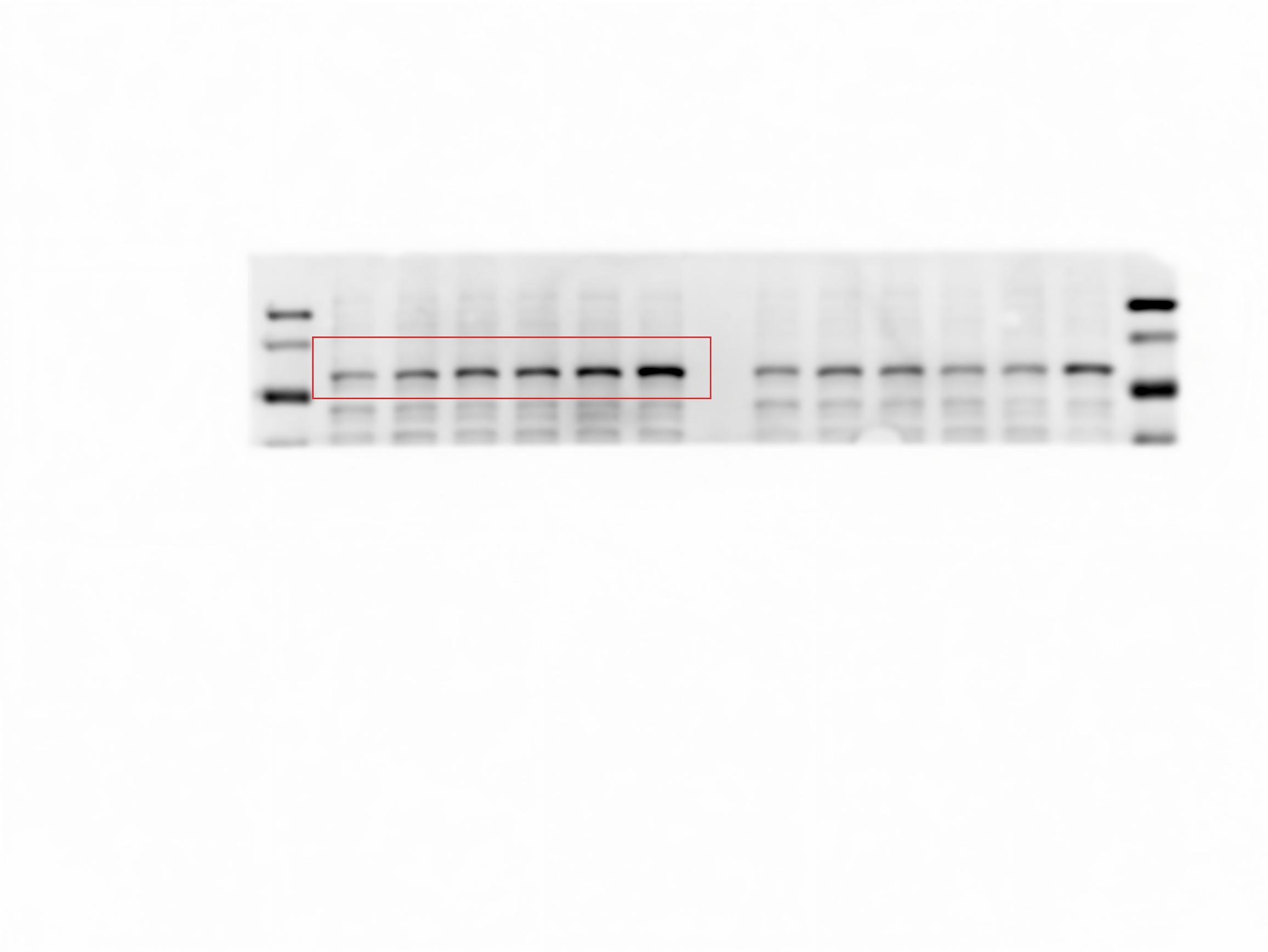

Supplement: Supplementary file 4 [file DataSheet2.zip › Fig4E NE IRF3 edited showing band.jpg]

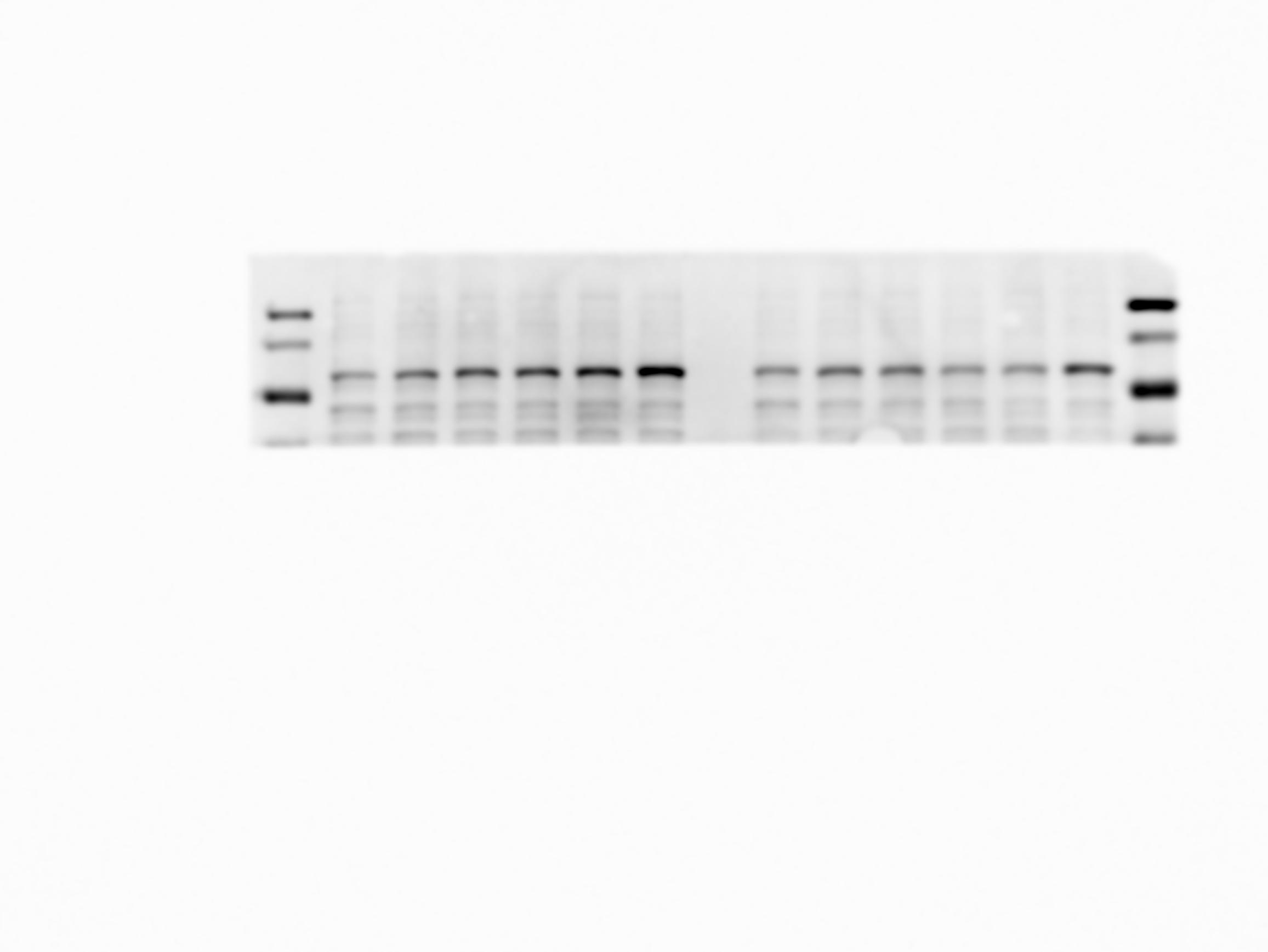

Supplement: Supplementary file 4 [file DataSheet2.zip › Fig4E NE IRF3.jpg]

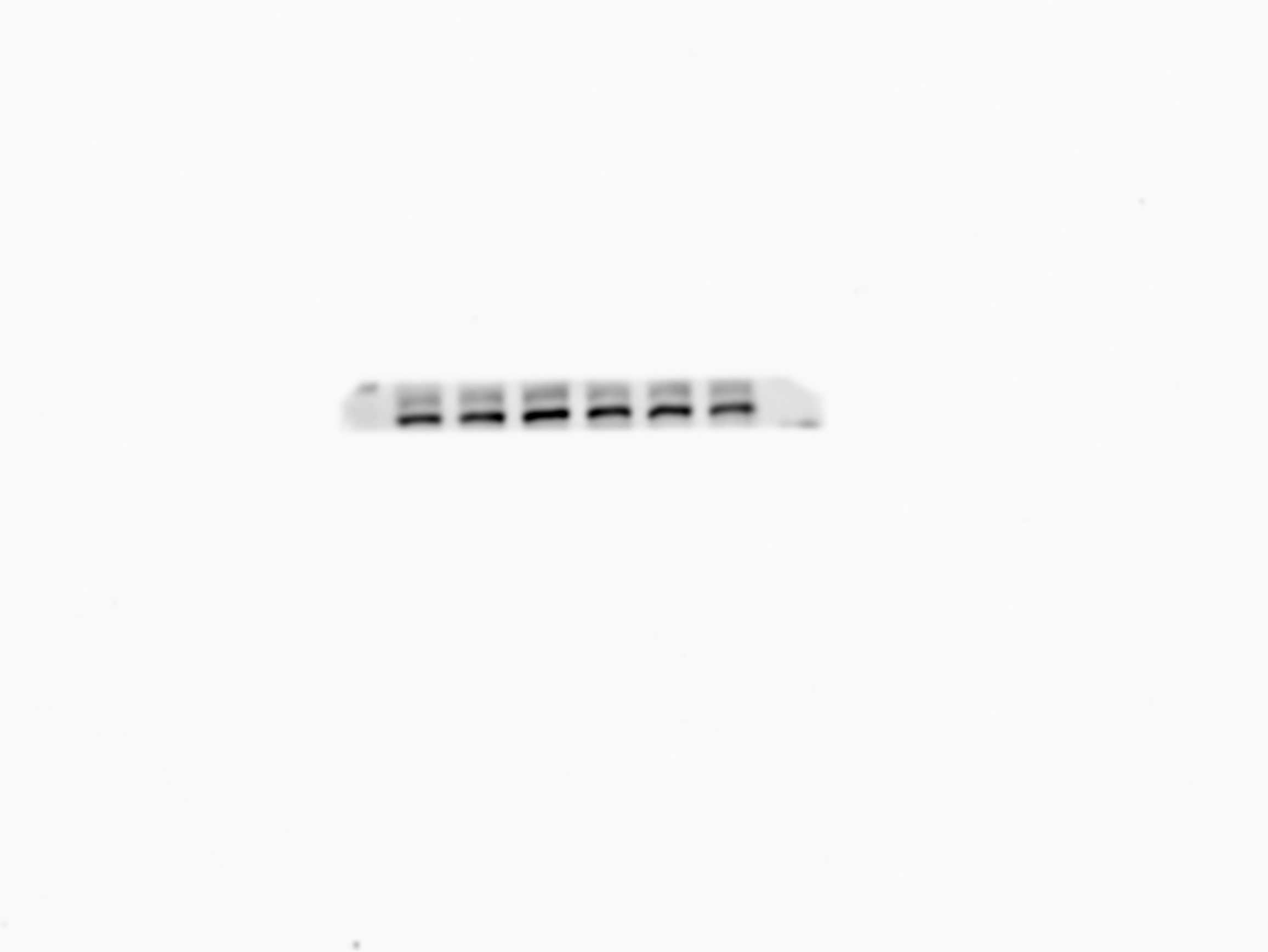

Supplement: Supplementary file 4 [file DataSheet2.zip › Fig4E NE lamin B.tif]

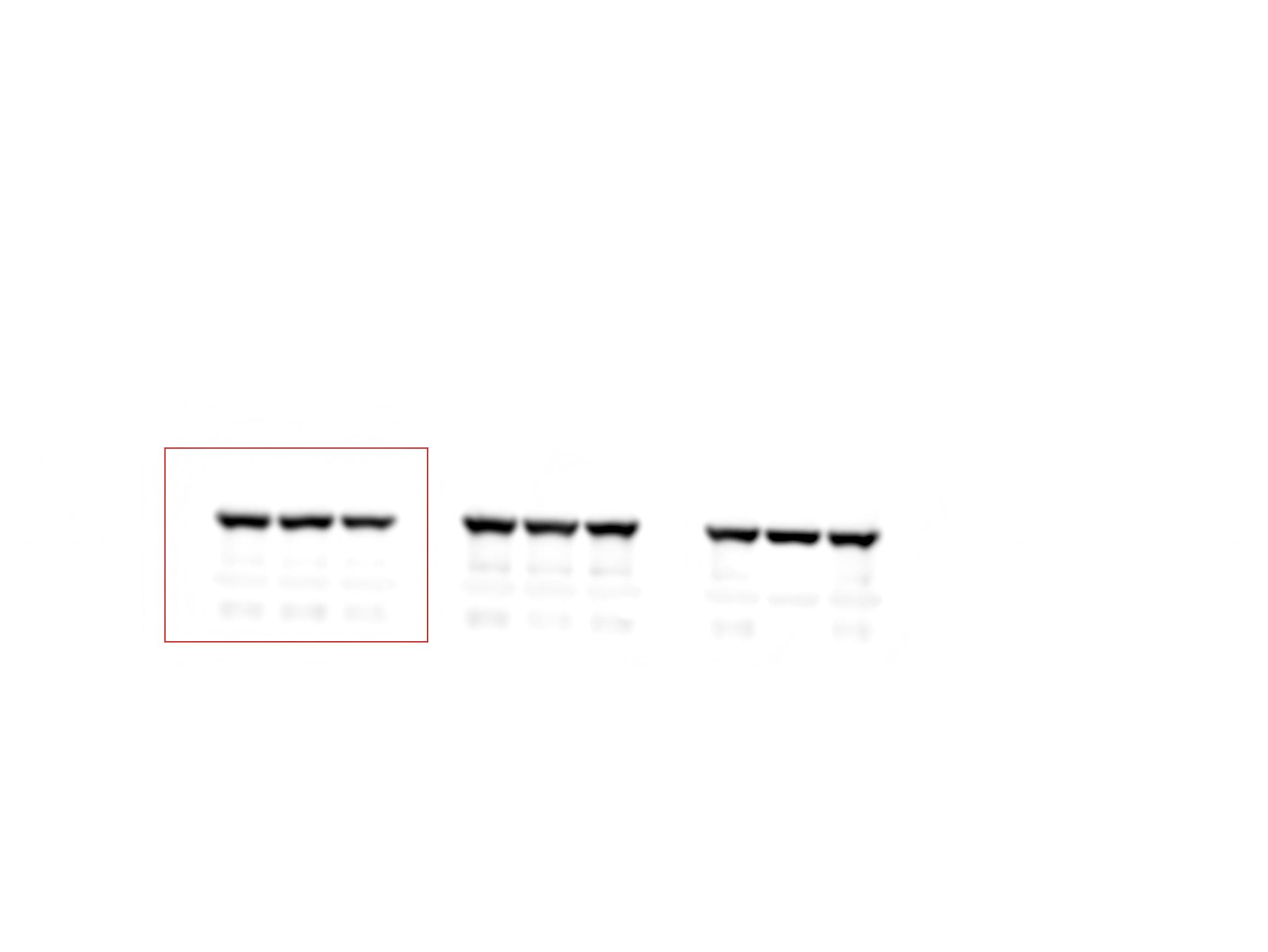

Supplement: Supplementary file 5 [file DataSheet3.zip › Fig5 D HA-RIG-I Input Actin edited showing band.jpg]

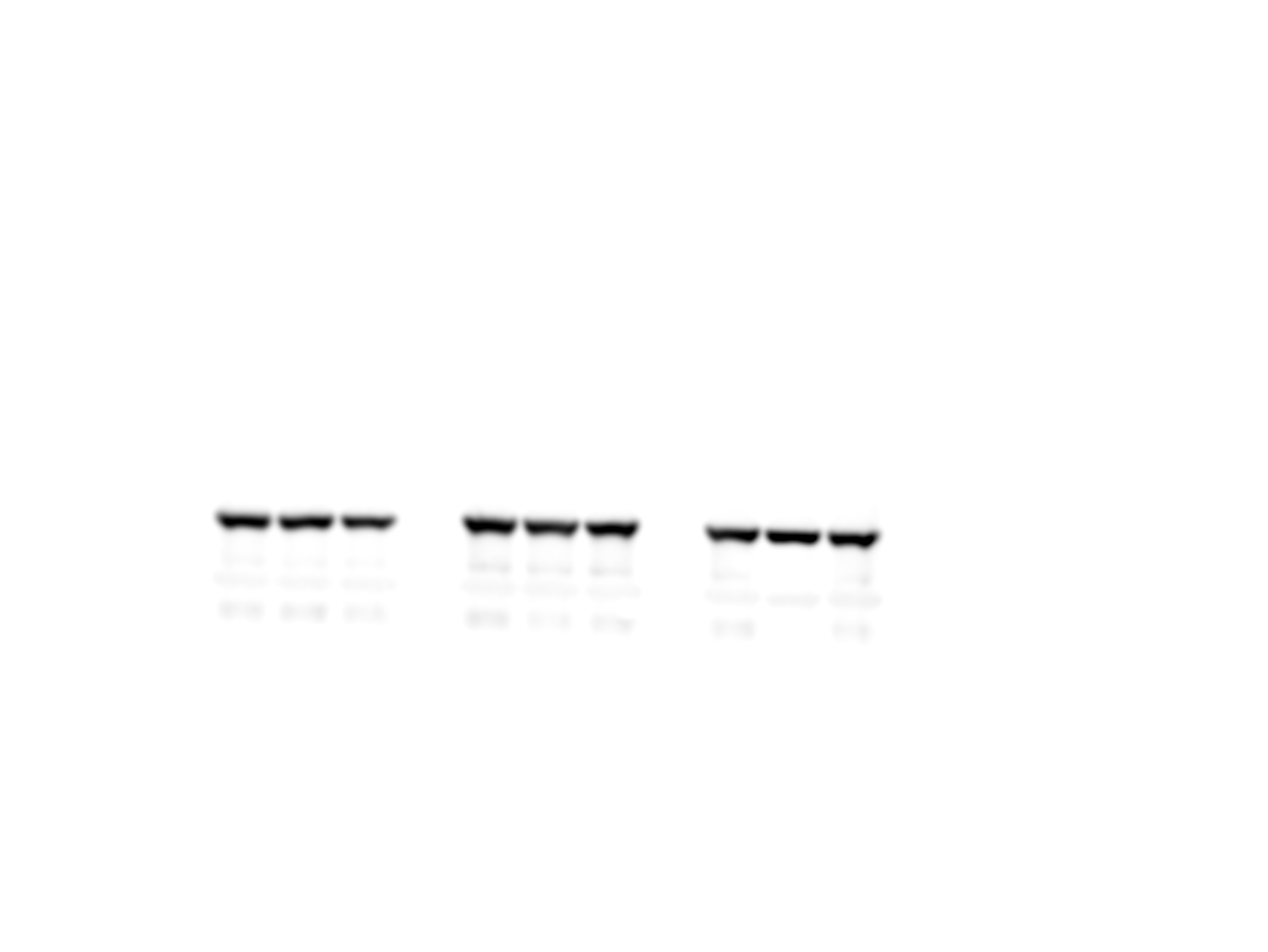

Supplement: Supplementary file 5 [file DataSheet3.zip › Fig5 D HA-RIG-I Input Actin.tif]

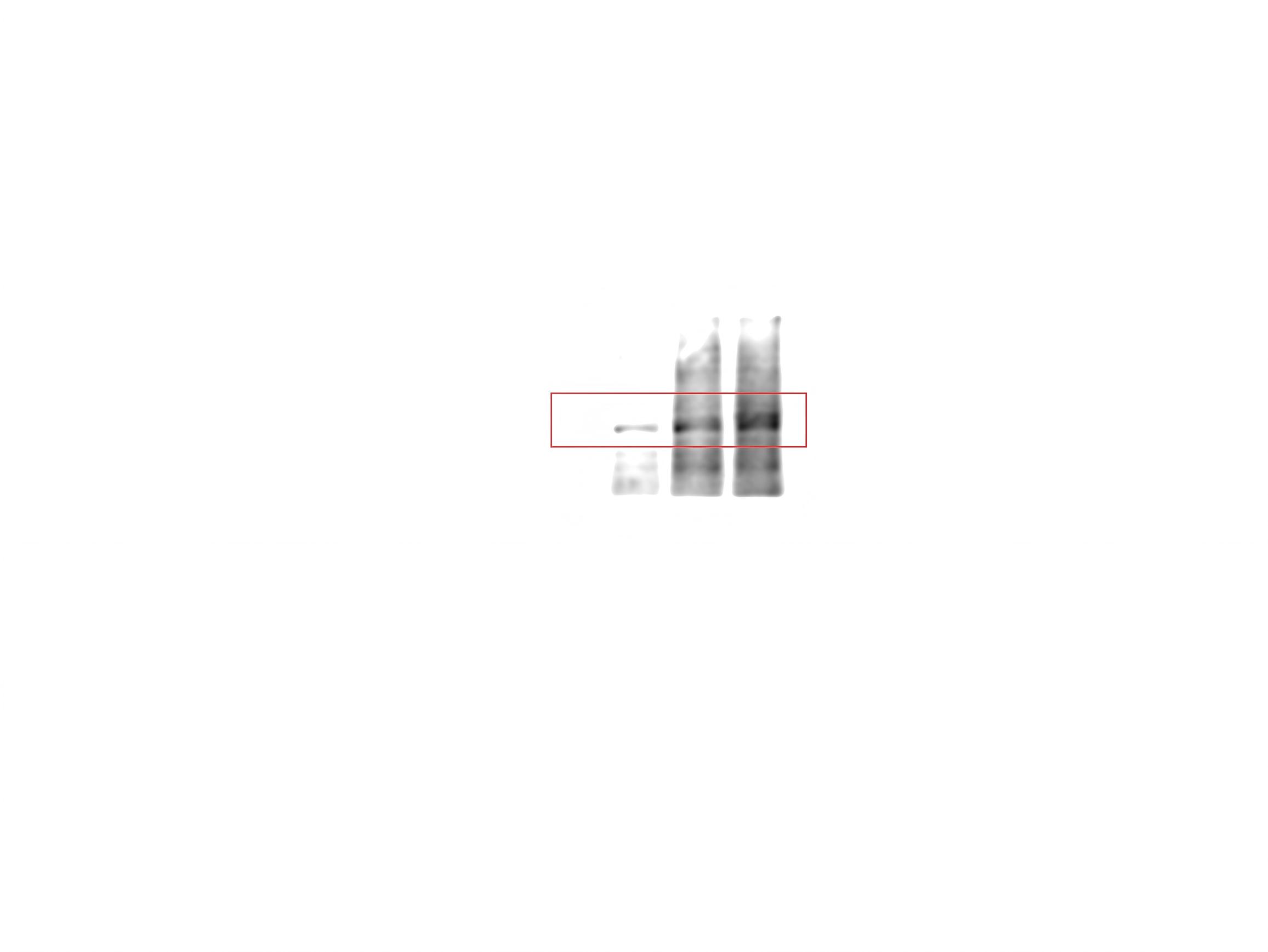

Supplement: Supplementary file 5 [file DataSheet3.zip › Fig5 D HA-RIG-I Input HA edited showing band.jpg]

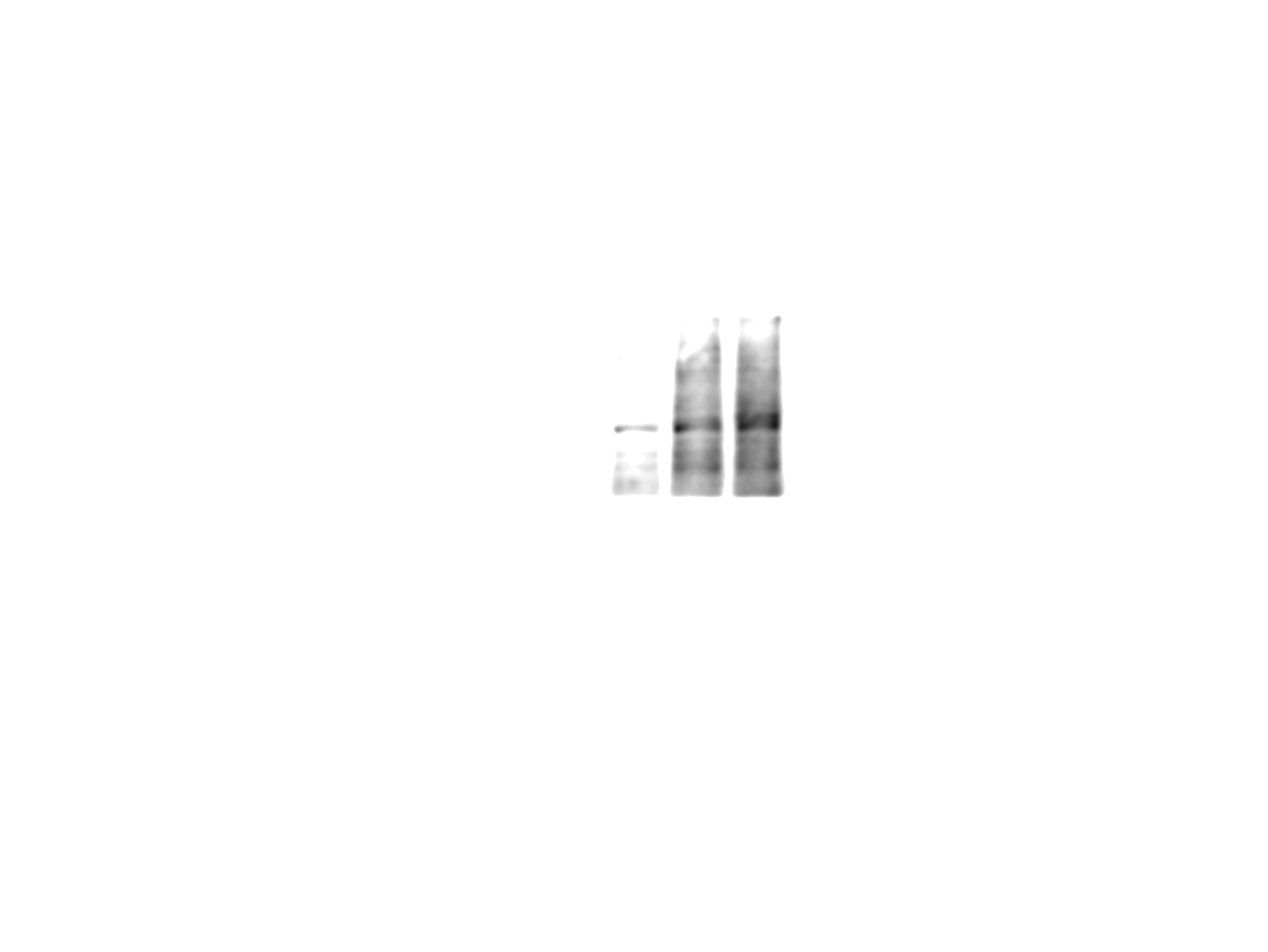

Supplement: Supplementary file 5 [file DataSheet3.zip › Fig5 D HA-RIG-I Input HA.tif]

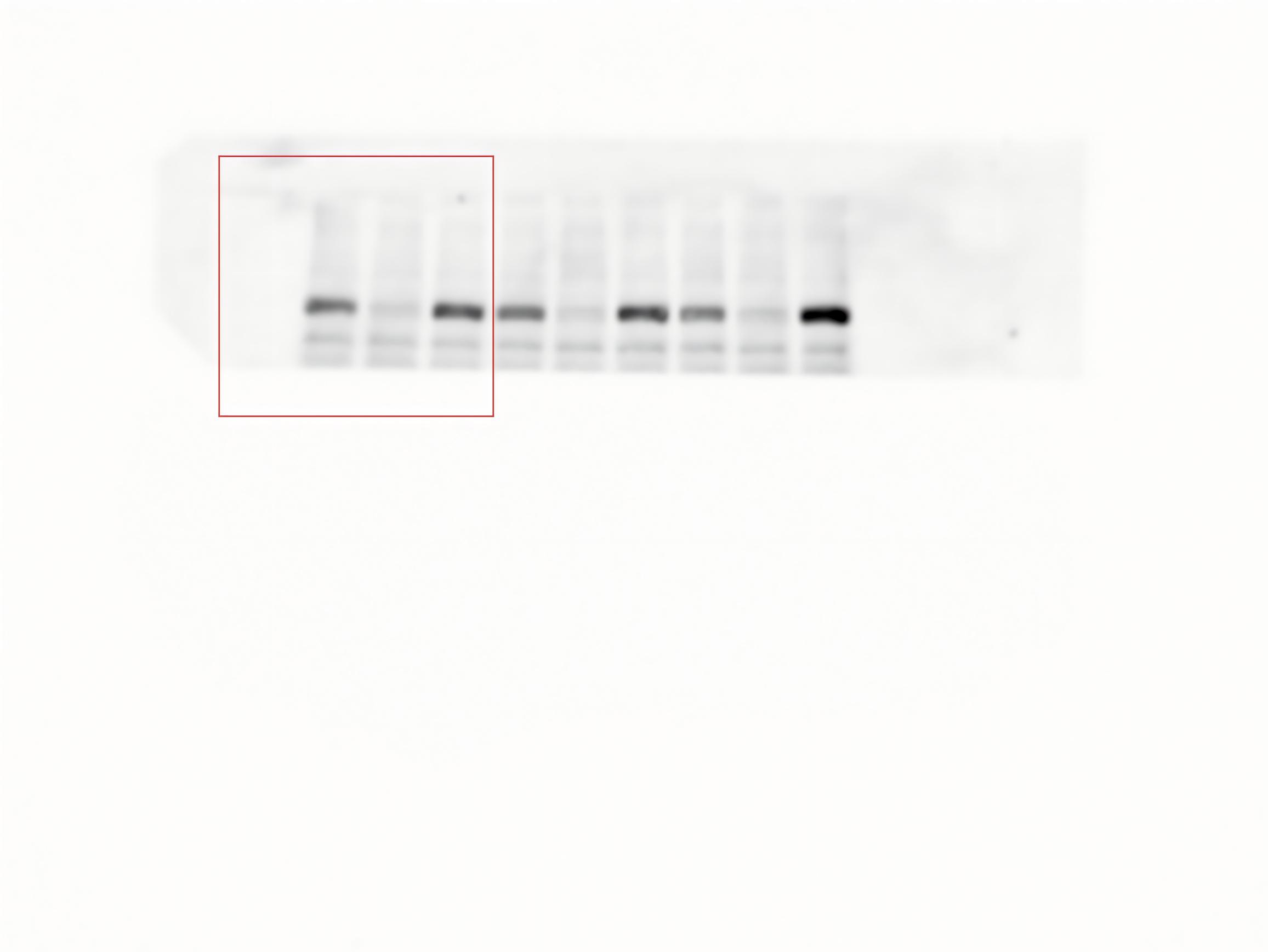

Supplement: Supplementary file 5 [file DataSheet3.zip › Fig5 D HA-RIG-I Input Myc edited showing band.jpg]

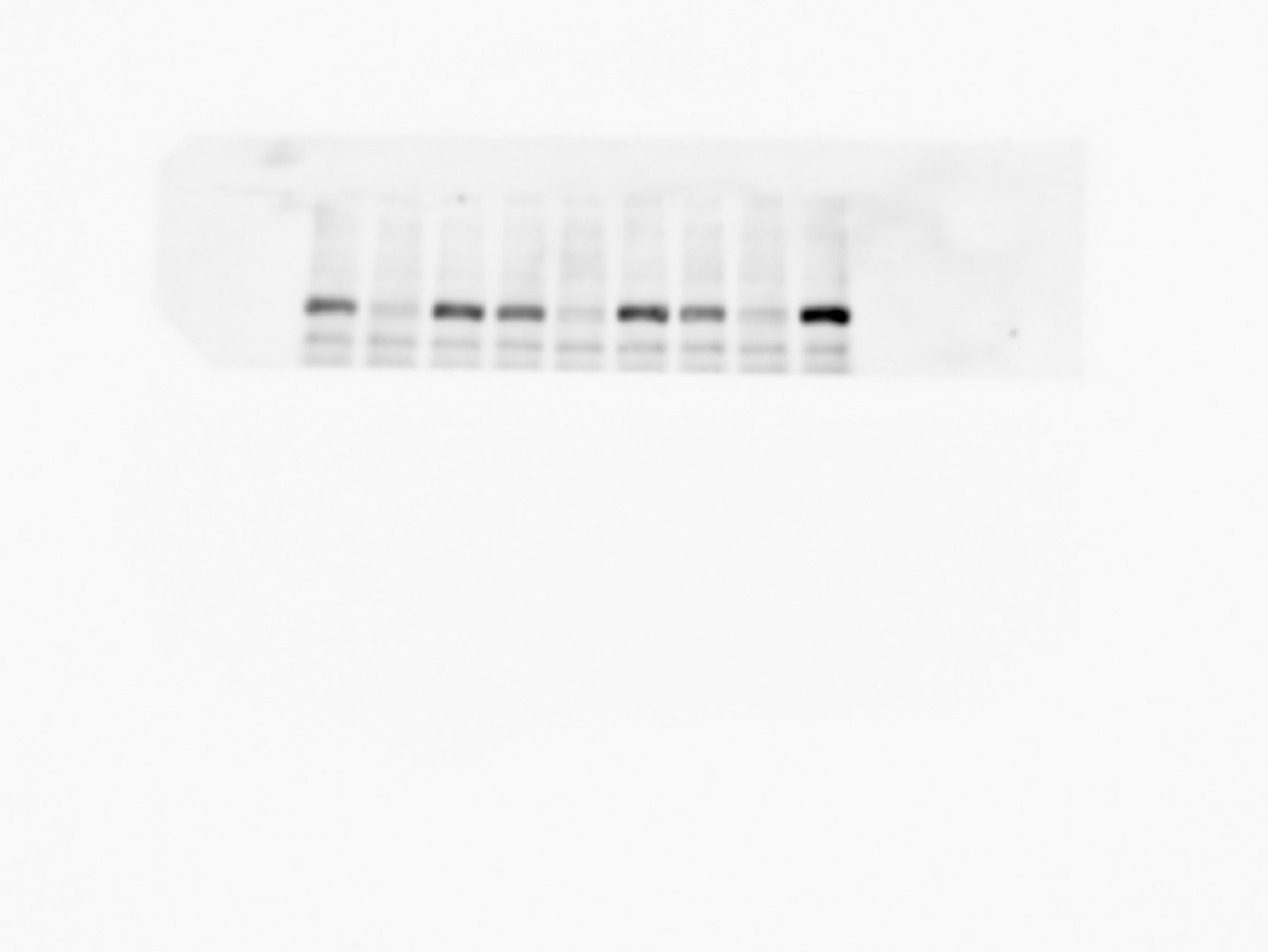

Supplement: Supplementary file 5 [file DataSheet3.zip › Fig5 D HA-RIG-I Input Myc.tif]

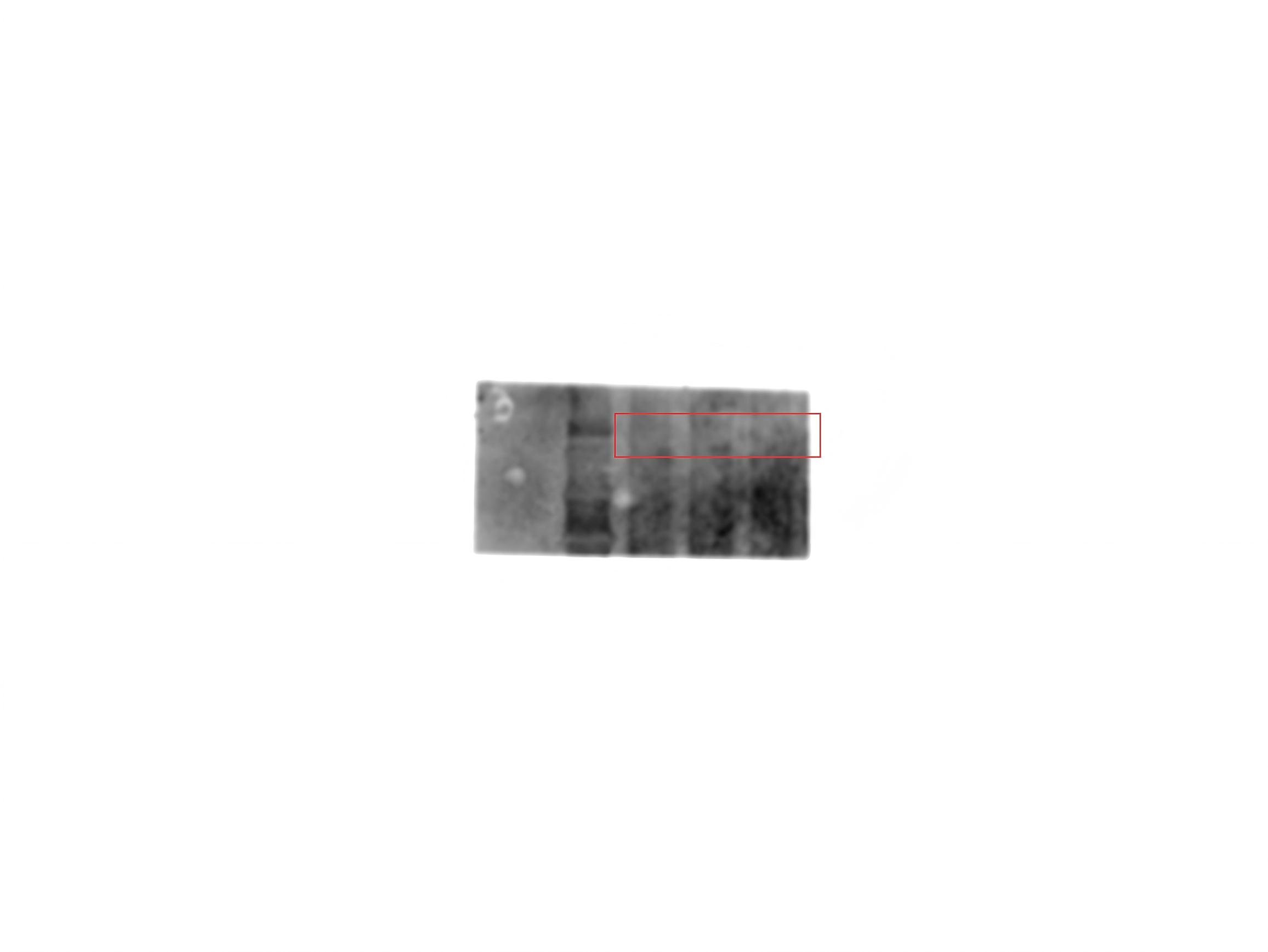

Supplement: Supplementary file 5 [file DataSheet3.zip › Fig5 D HA-RIG-I IP HA edited showing band.jpg]

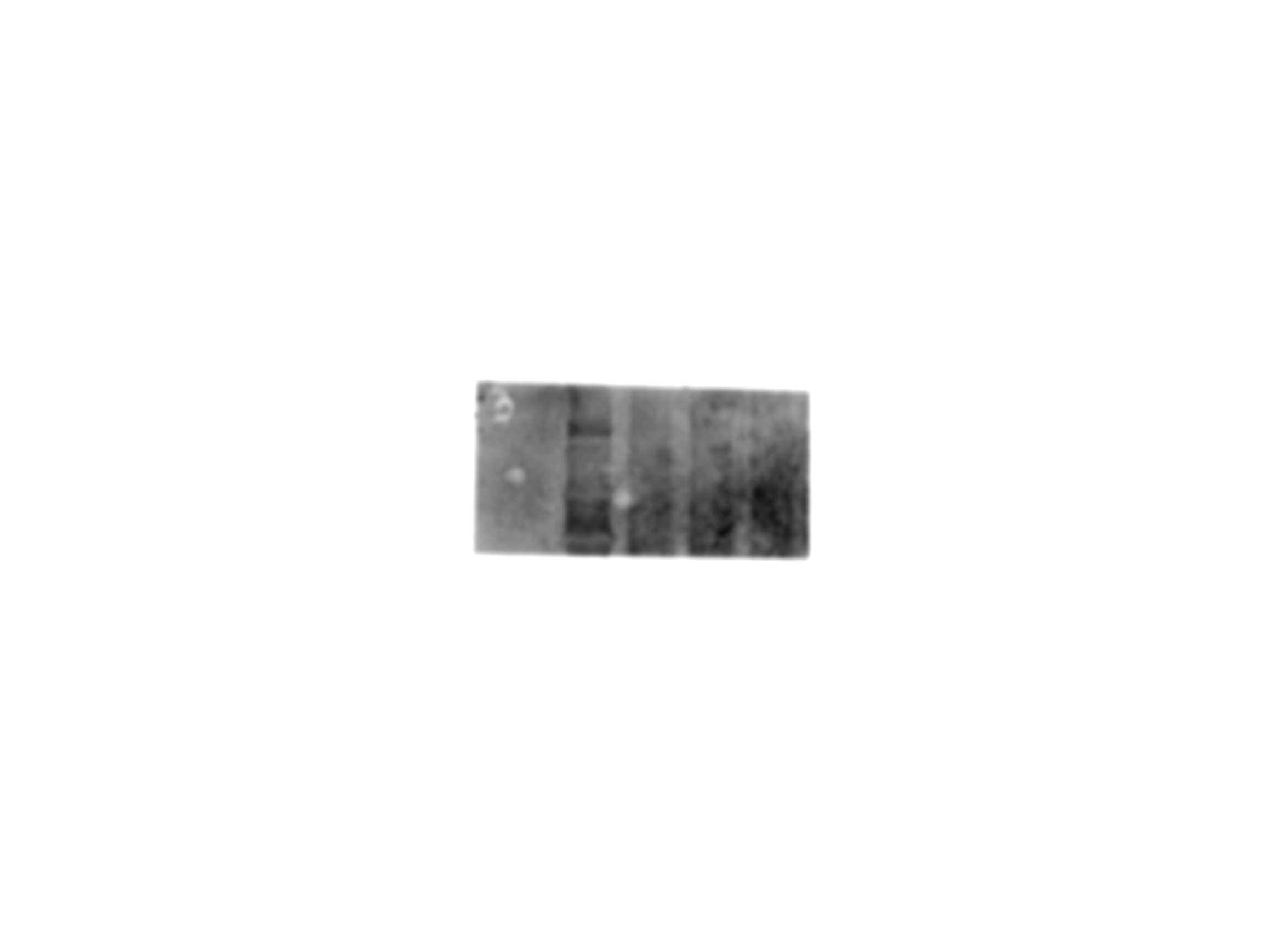

Supplement: Supplementary file 5 [file DataSheet3.zip › Fig5 D HA-RIG-I IP HA.tif]

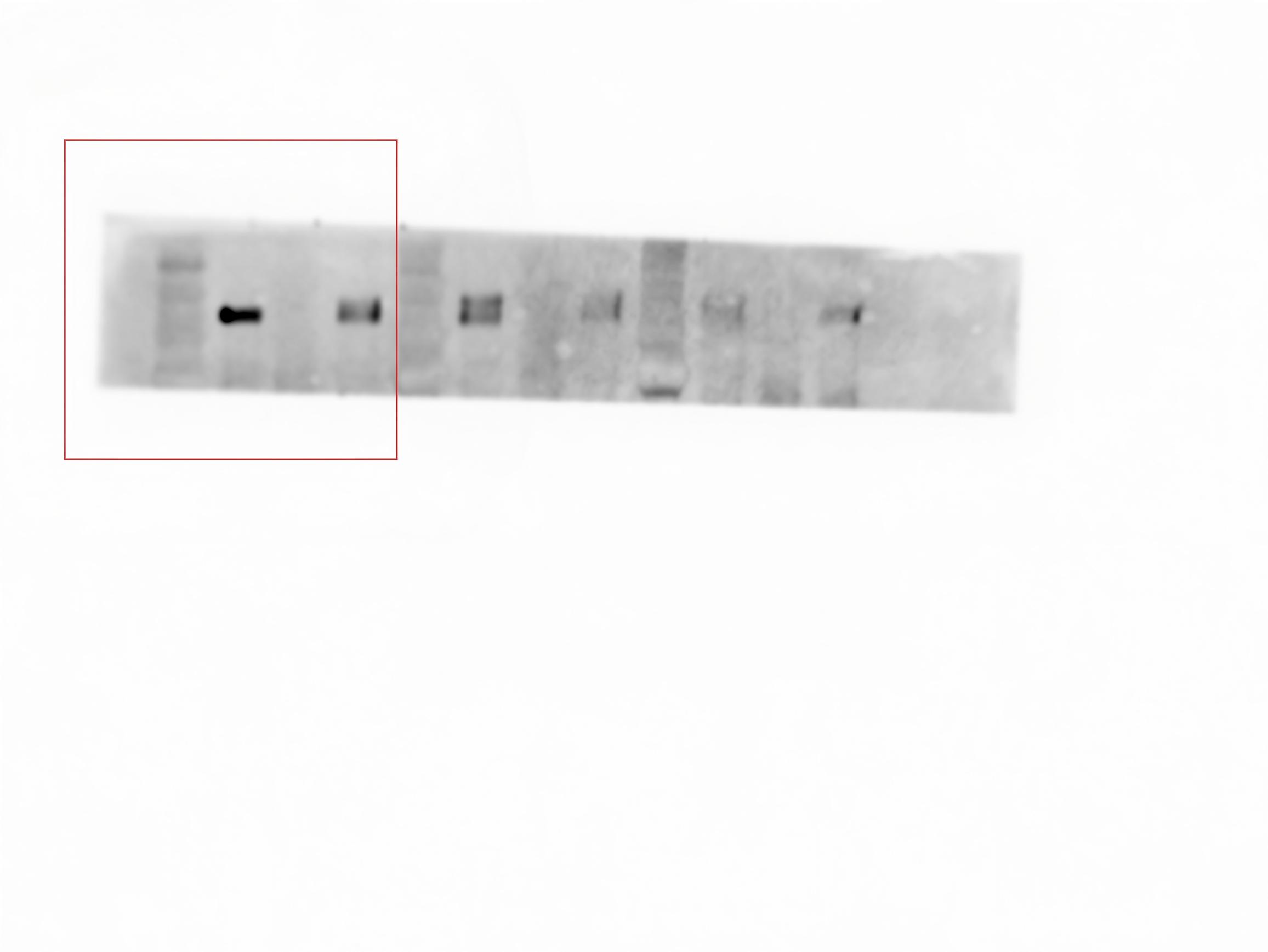

Supplement: Supplementary file 5 [file DataSheet3.zip › Fig5 D HA-RIG-I IP Myc edited showing band.jpg]

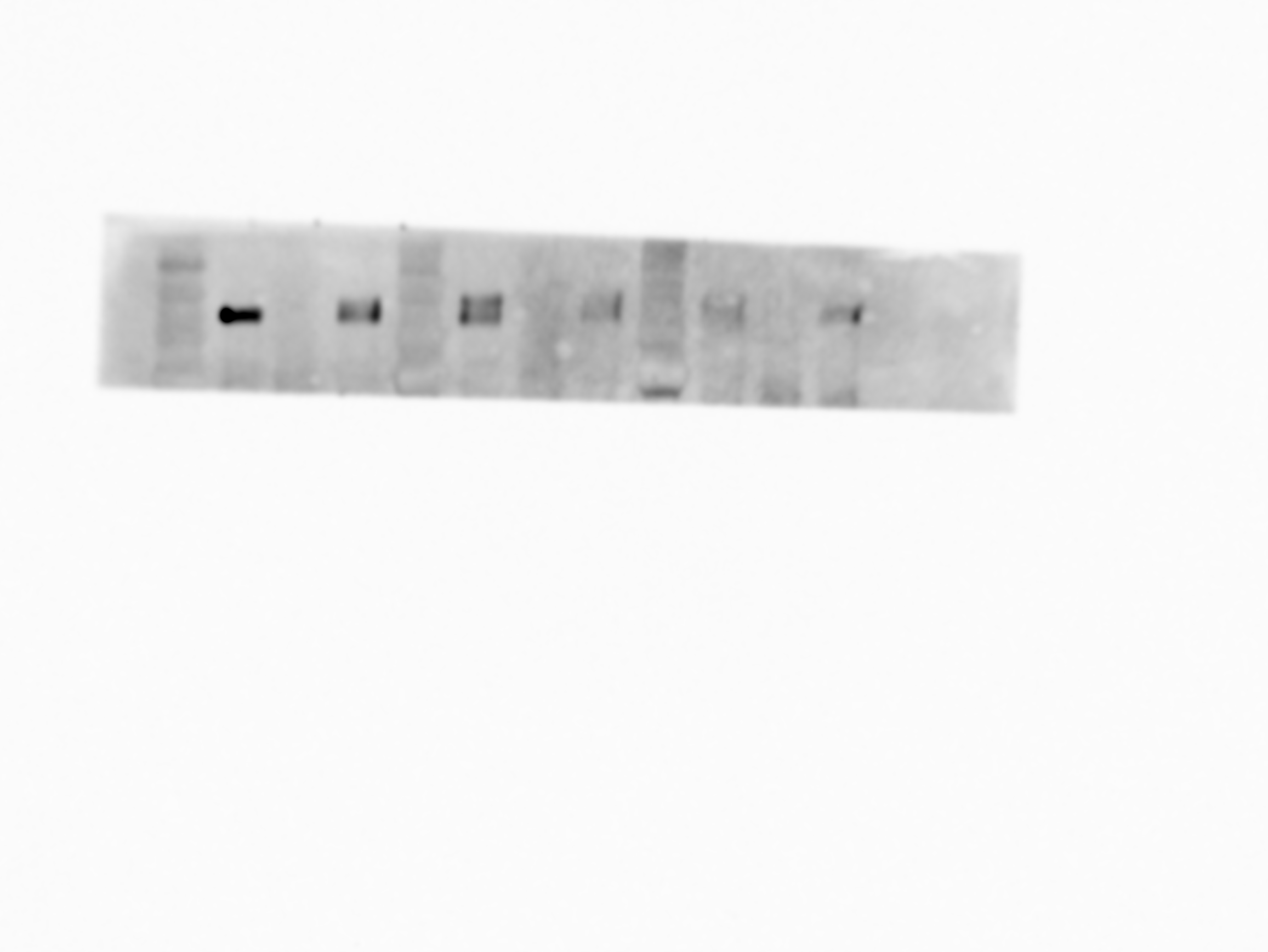

Supplement: Supplementary file 5 [file DataSheet3.zip › Fig5 D HA-RIG-I IP Myc.tif]

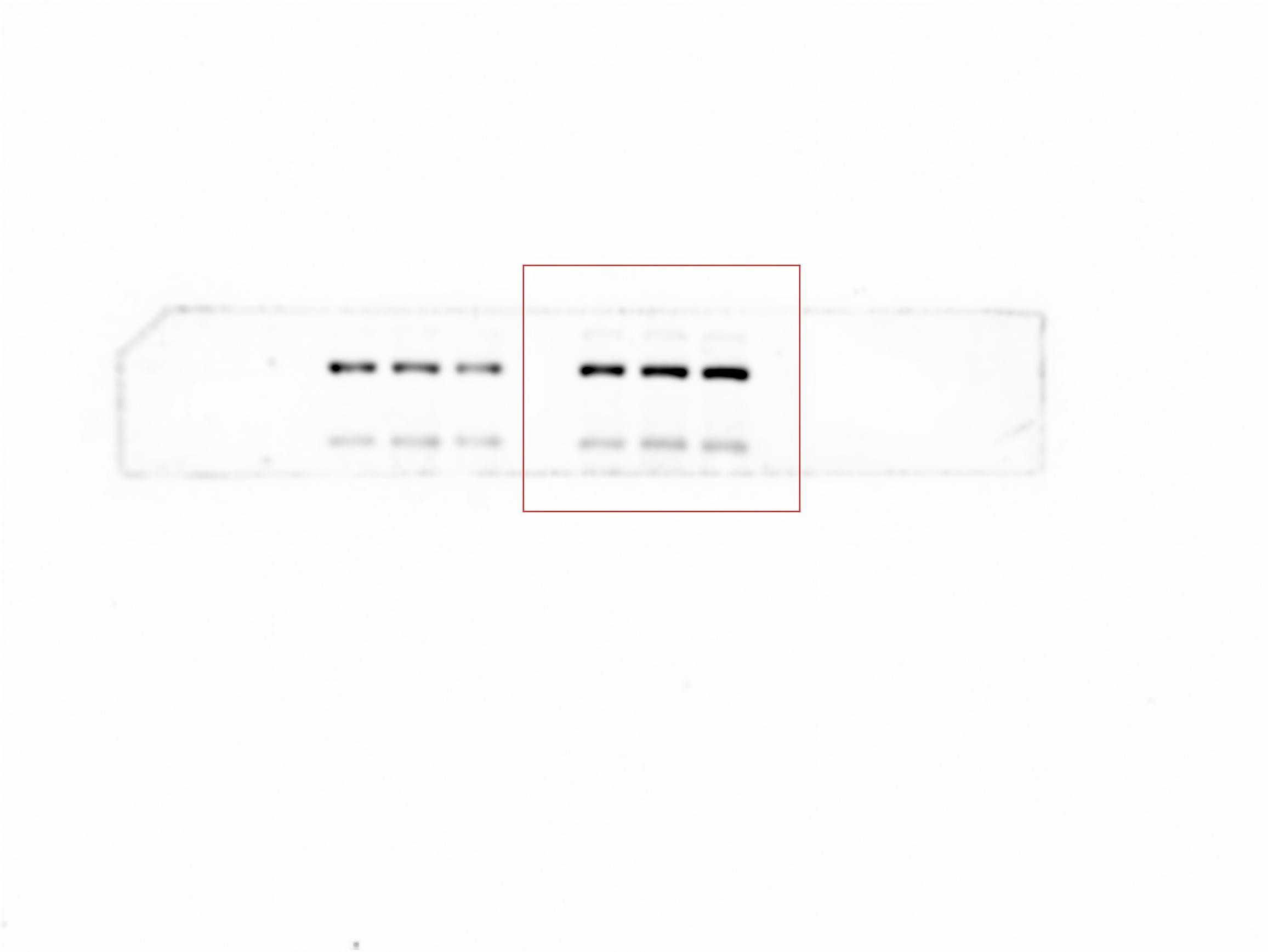

Supplement: Supplementary file 5 [file DataSheet3.zip › Fig5 D HA-TBK1 Input Actin edited showing band.jpg]

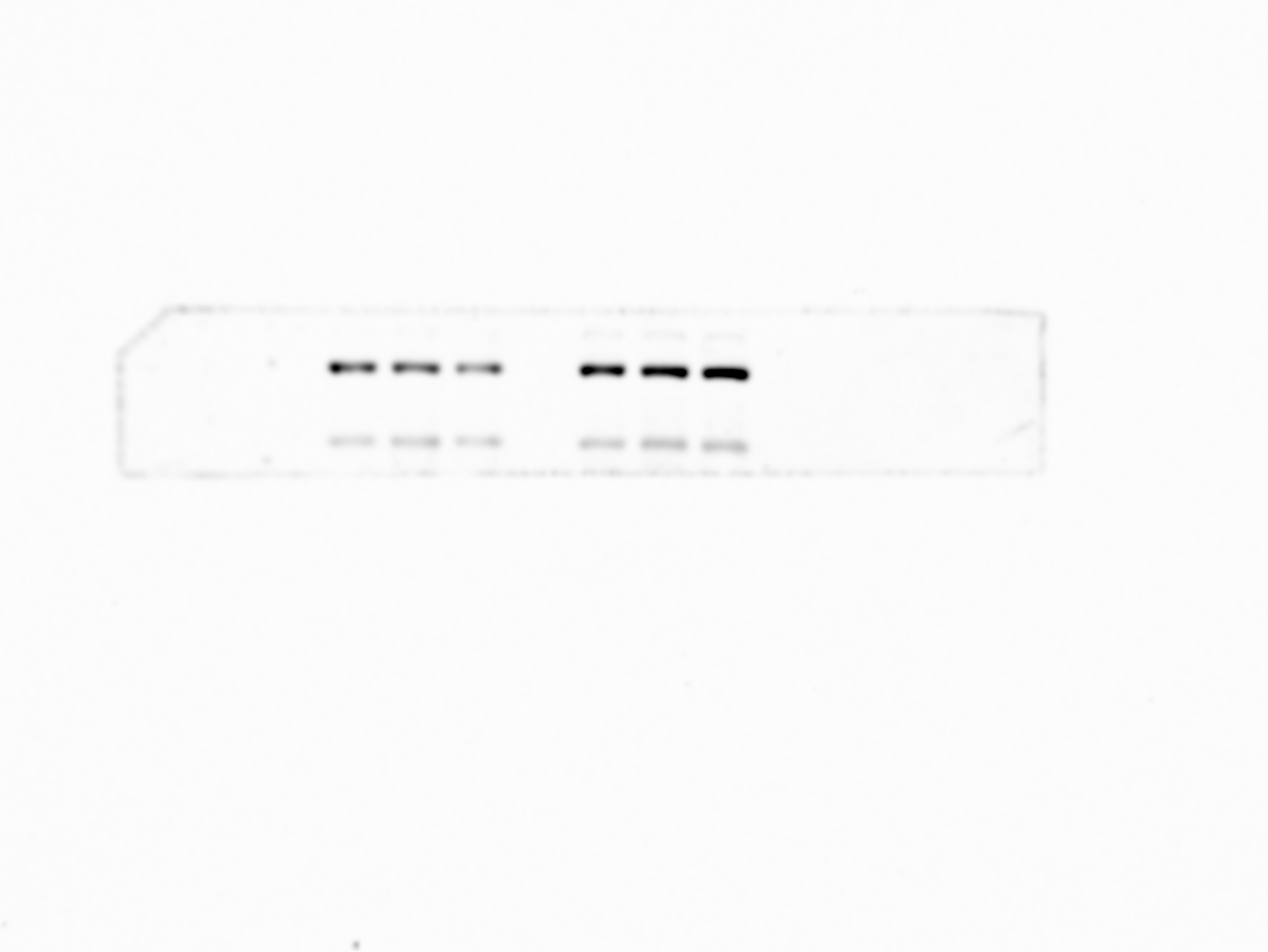

Supplement: Supplementary file 5 [file DataSheet3.zip › Fig5 D HA-TBK1 Input Actin.tif]

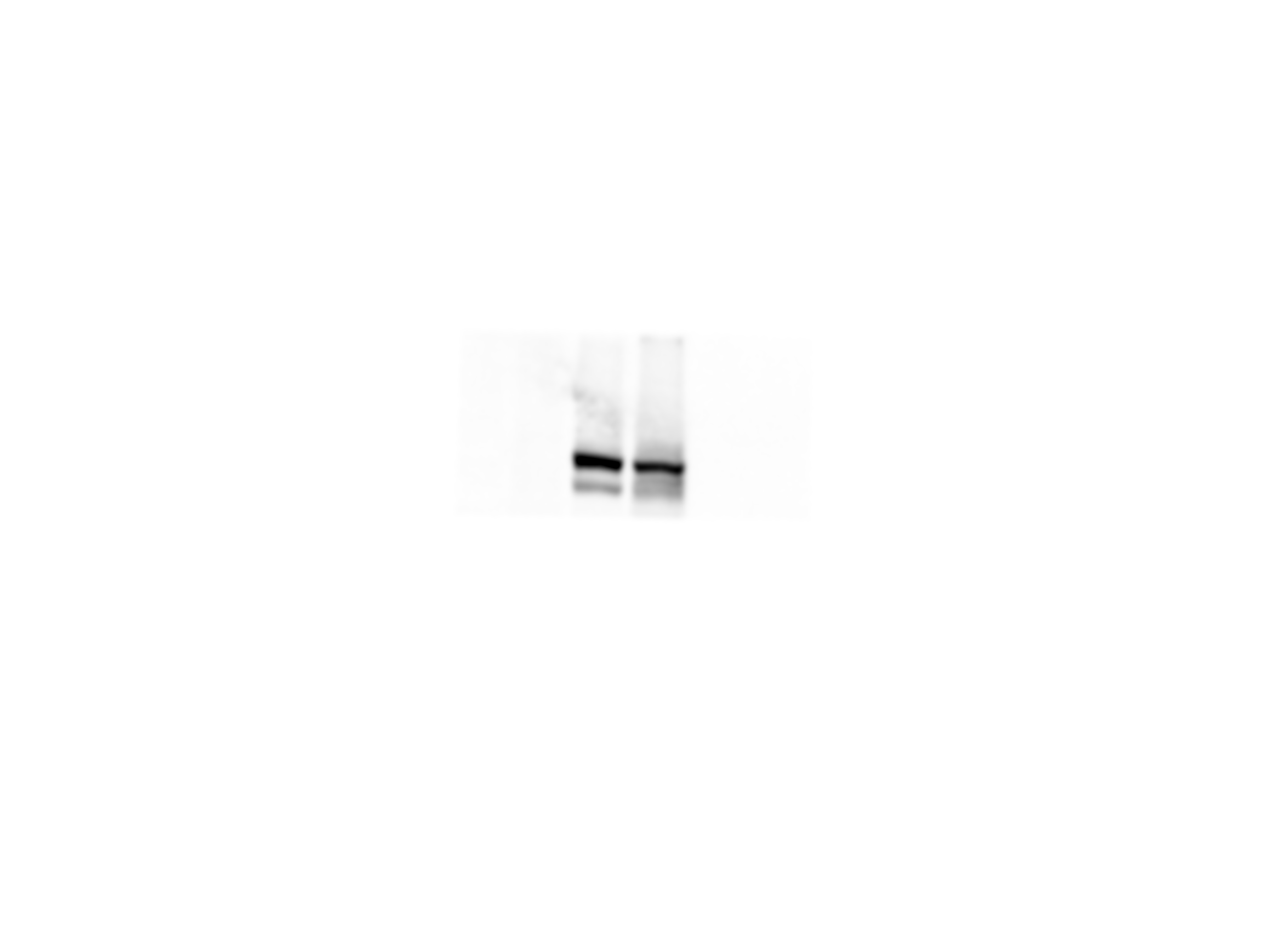

Supplement: Supplementary file 5 [file DataSheet3.zip › Fig5 D HA-TBK1 Input HA.tif]

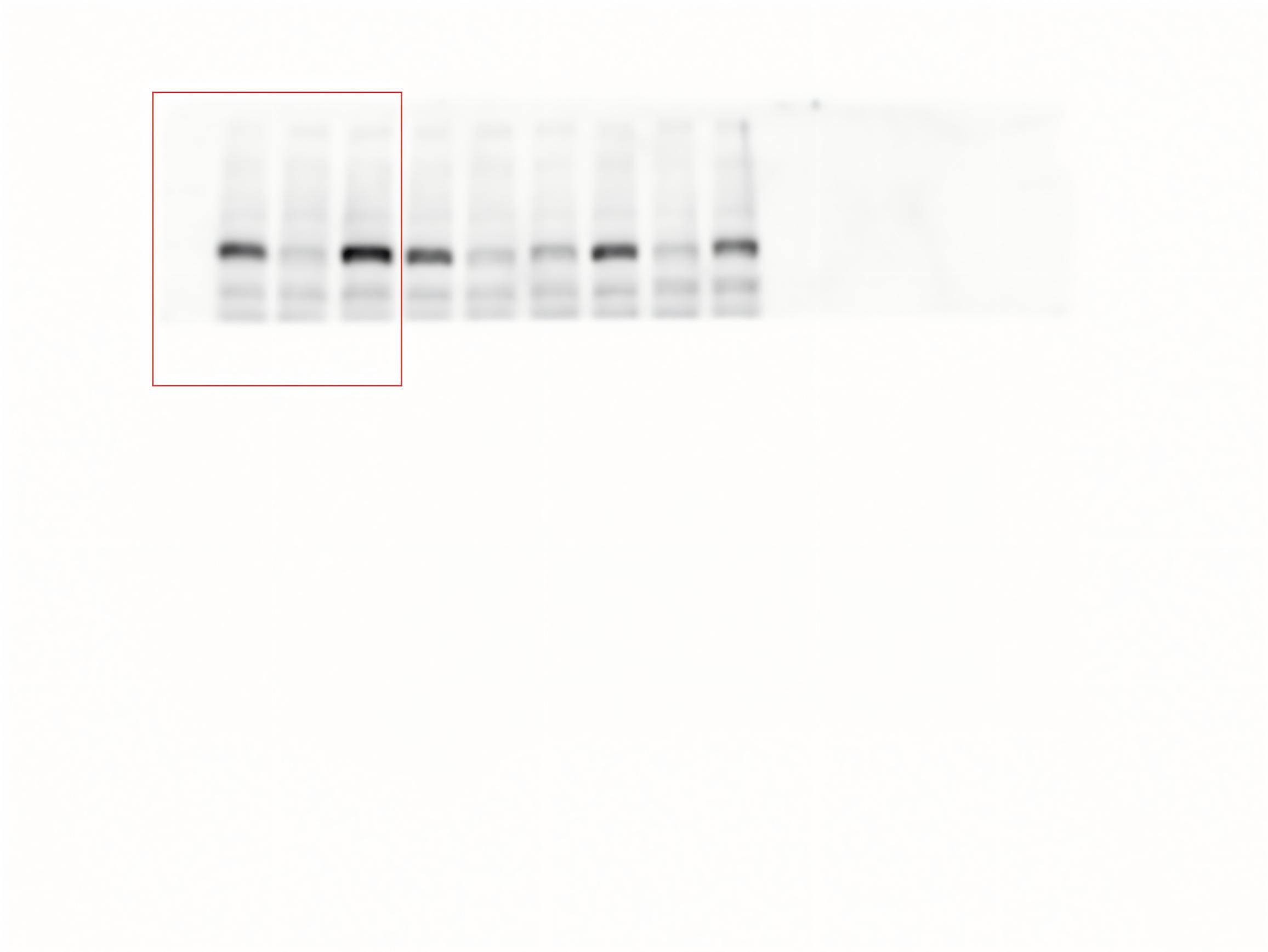

Supplement: Supplementary file 5 [file DataSheet3.zip › Fig5 D HA-TBK1 Input Myc edited showing band.jpg]

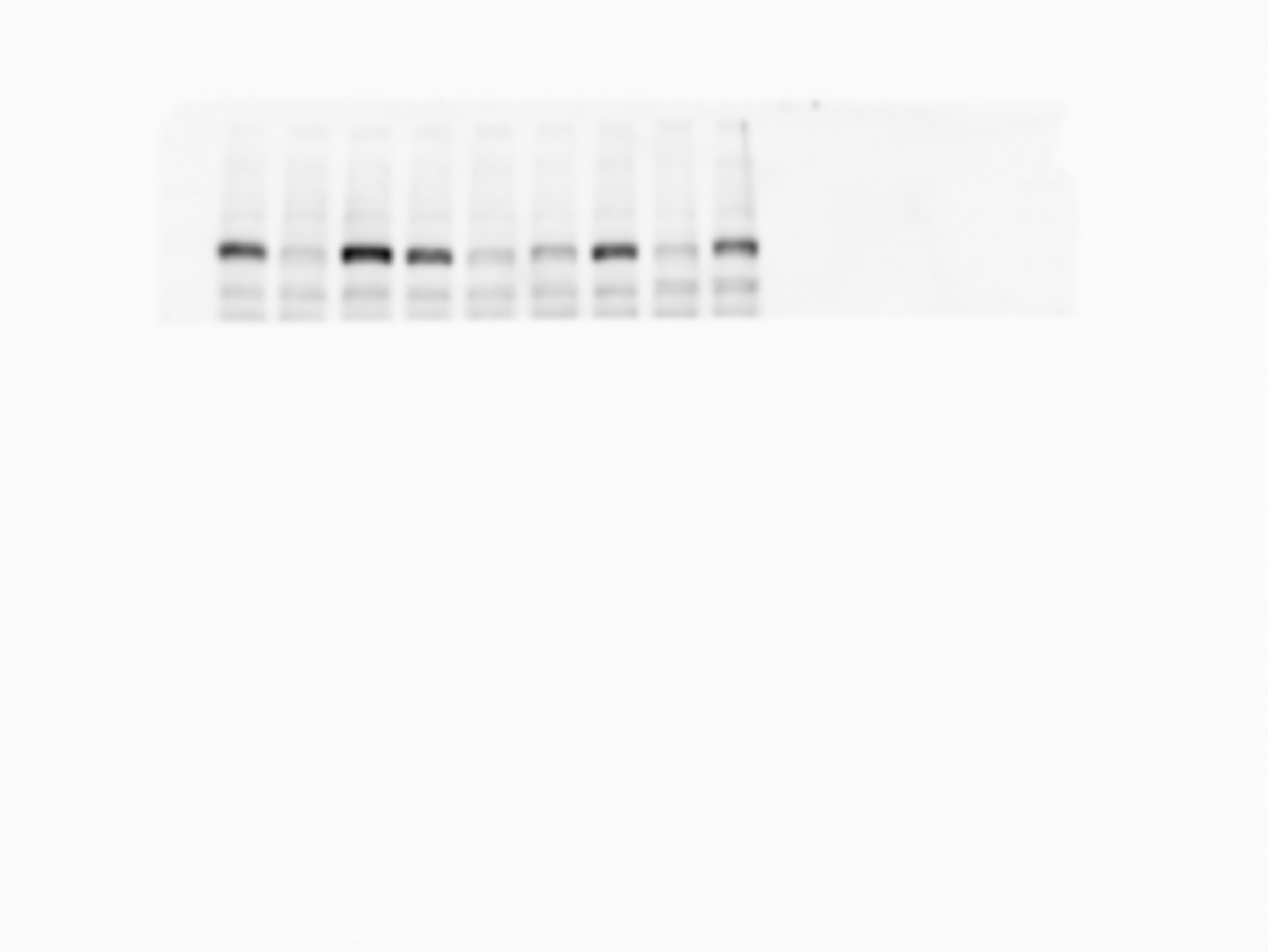

Supplement: Supplementary file 5 [file DataSheet3.zip › Fig5 D HA-TBK1 Input Myc.tif]

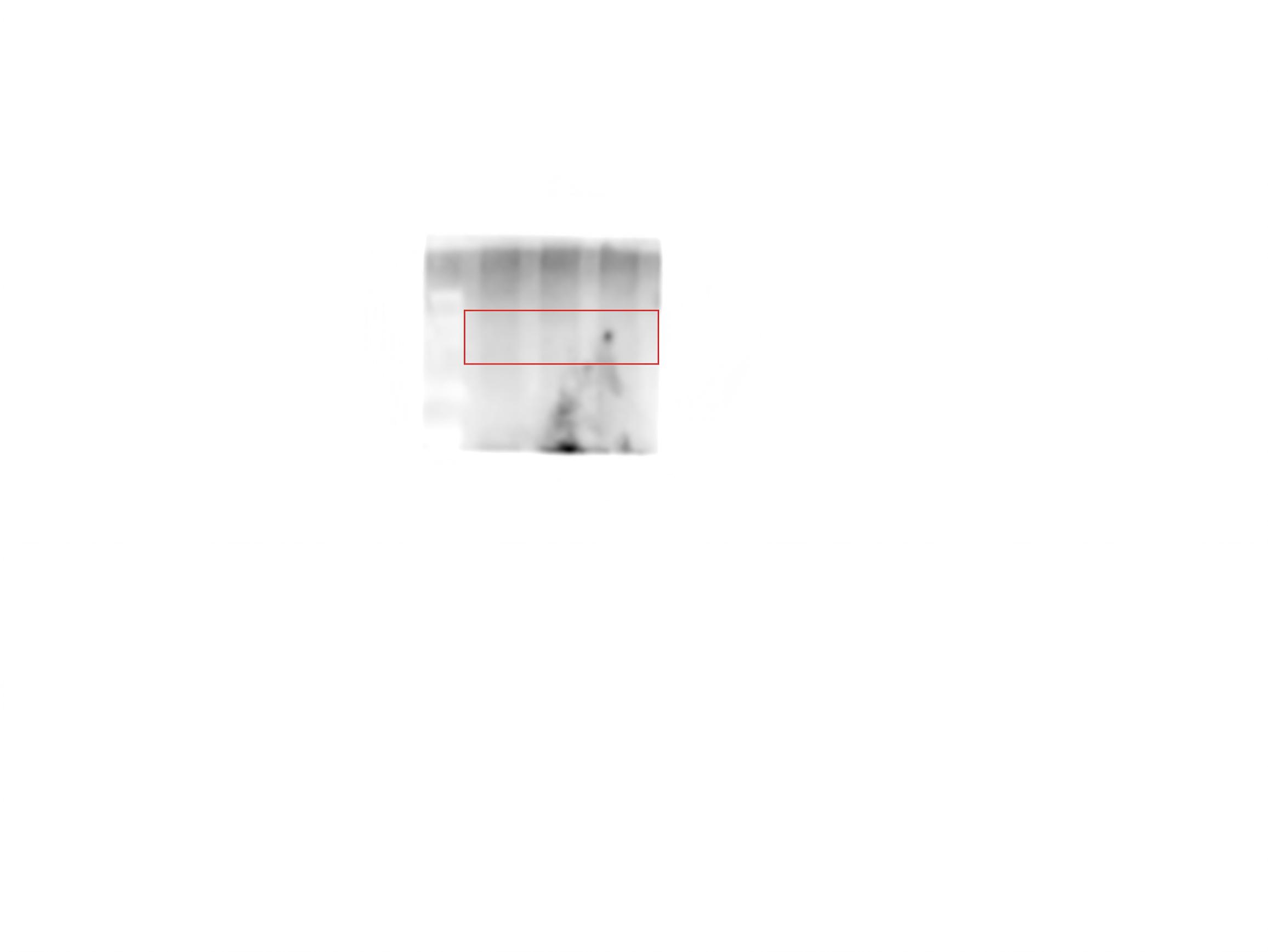

Supplement: Supplementary file 5 [file DataSheet3.zip › Fig5 D HA-TBK1 IP HA edited showing band.jpg]

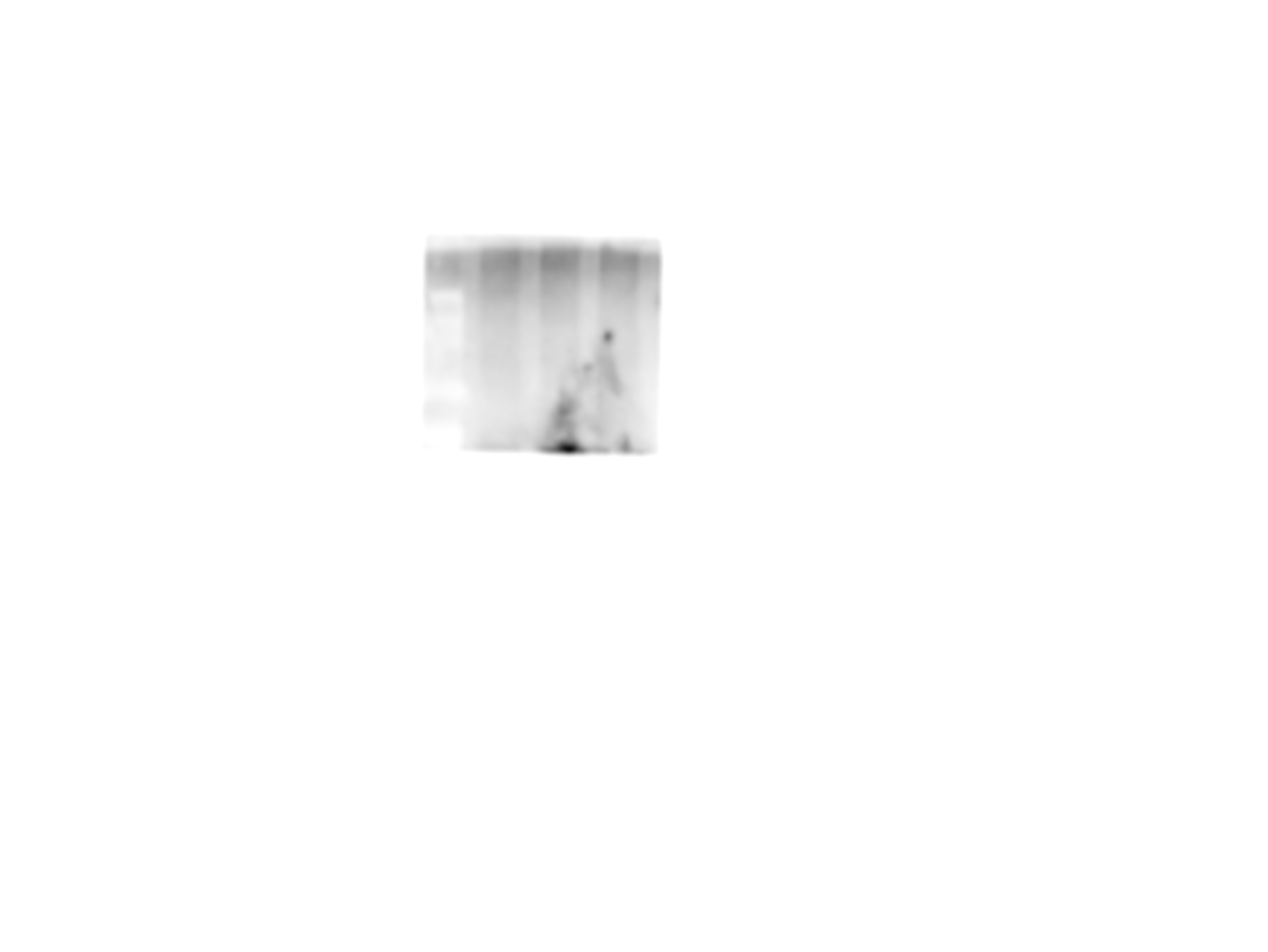

Supplement: Supplementary file 5 [file DataSheet3.zip › Fig5 D HA-TBK1 IP HA.tif]

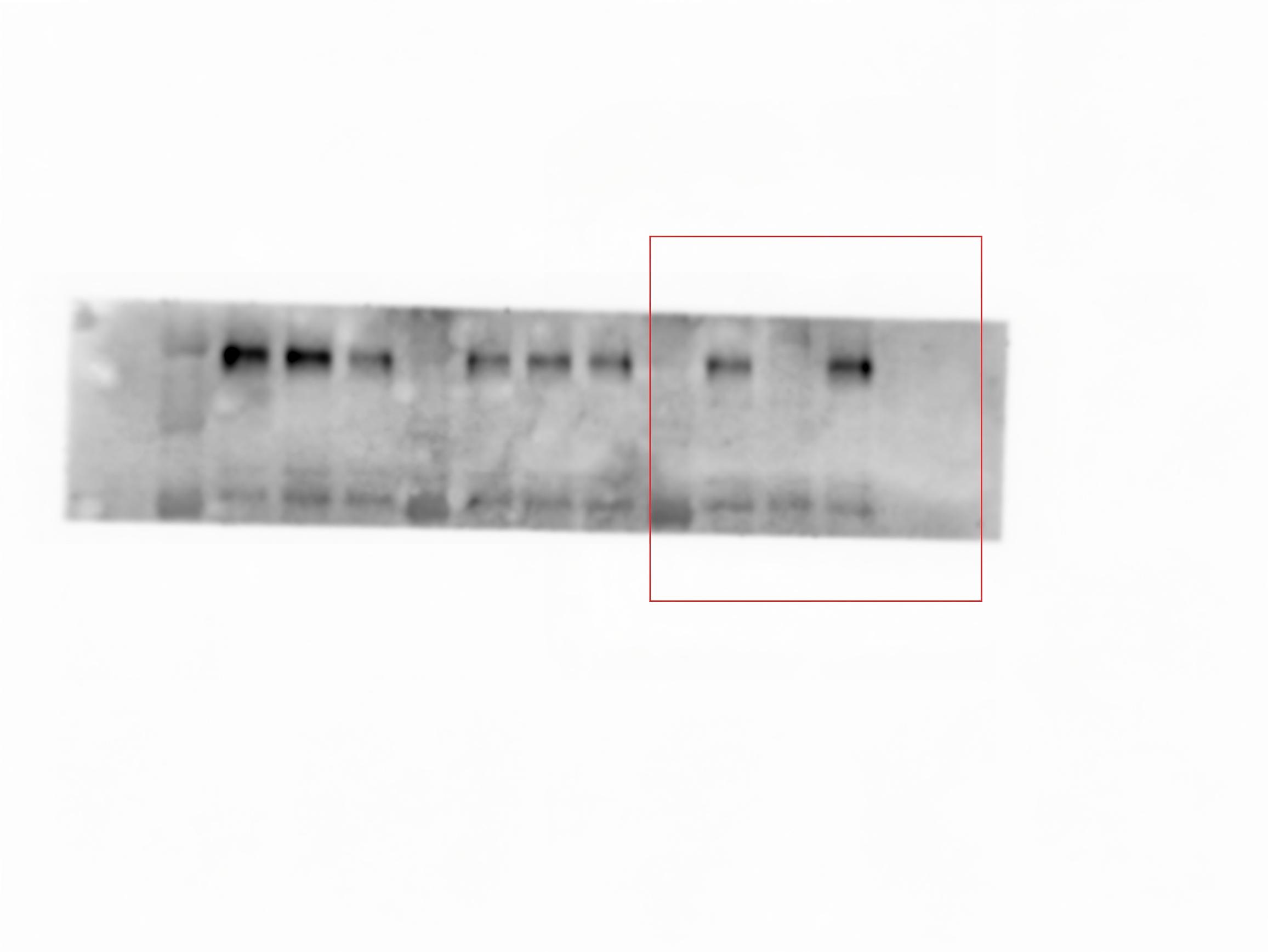

Supplement: Supplementary file 5 [file DataSheet3.zip › Fig5 D HA-TBK1 IP Myc edited showing band.jpg]

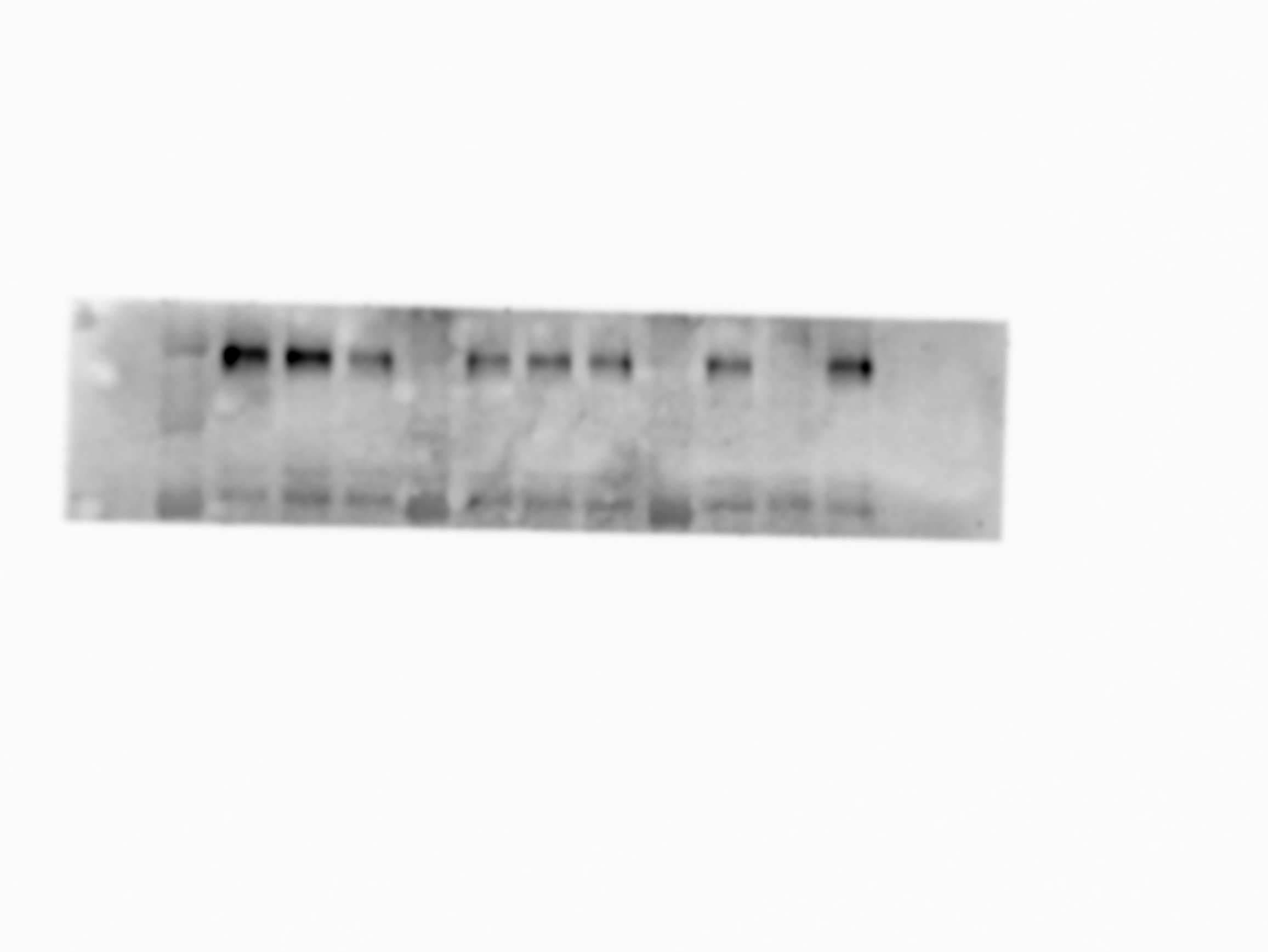

Supplement: Supplementary file 5 [file DataSheet3.zip › Fig5 D HA-TBK1 IP Myc.tif]

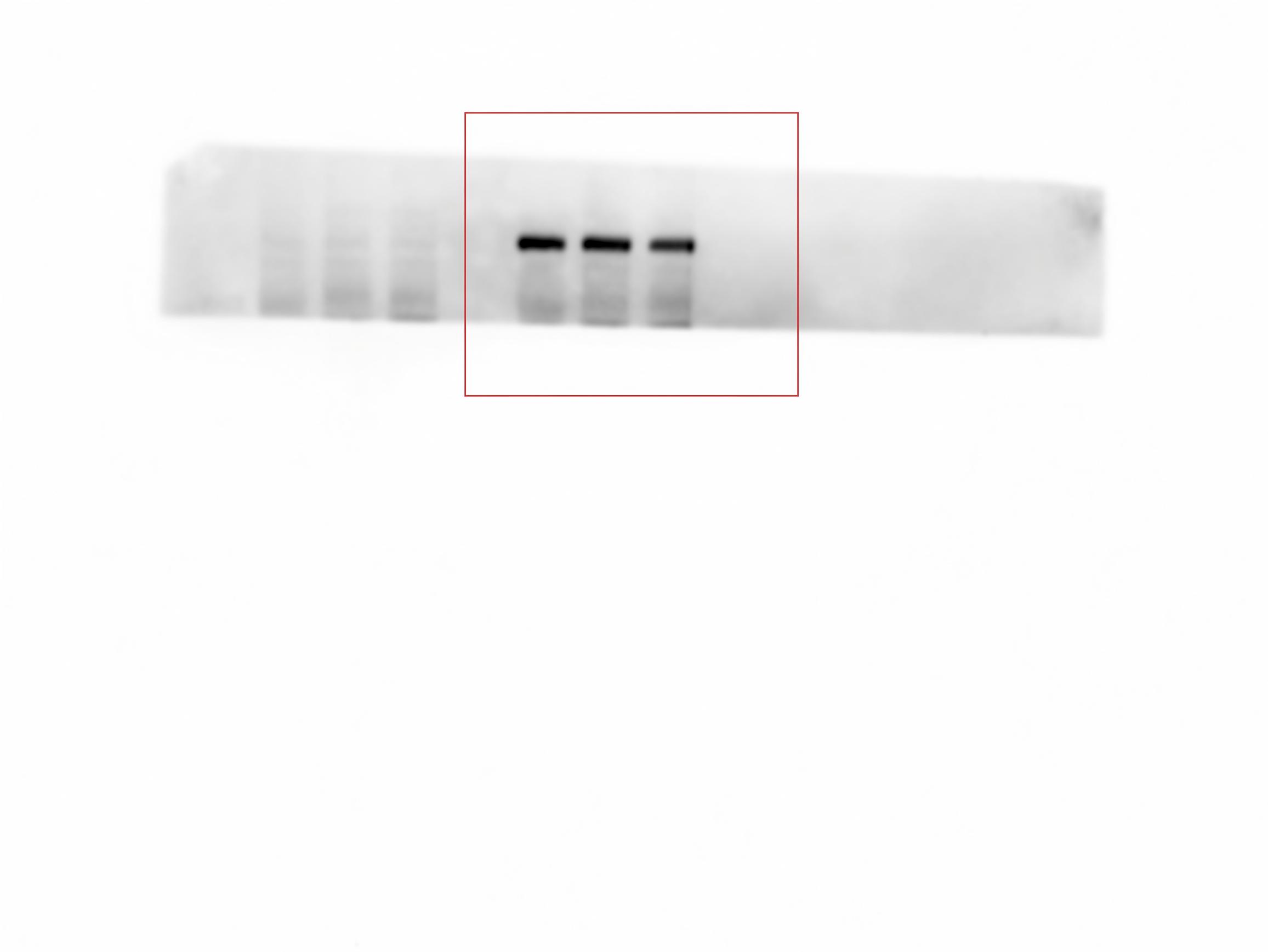

Supplement: Supplementary file 5 [file DataSheet3.zip › Fig5D HA-IRF3 Input Actin edited showing band.jpg]

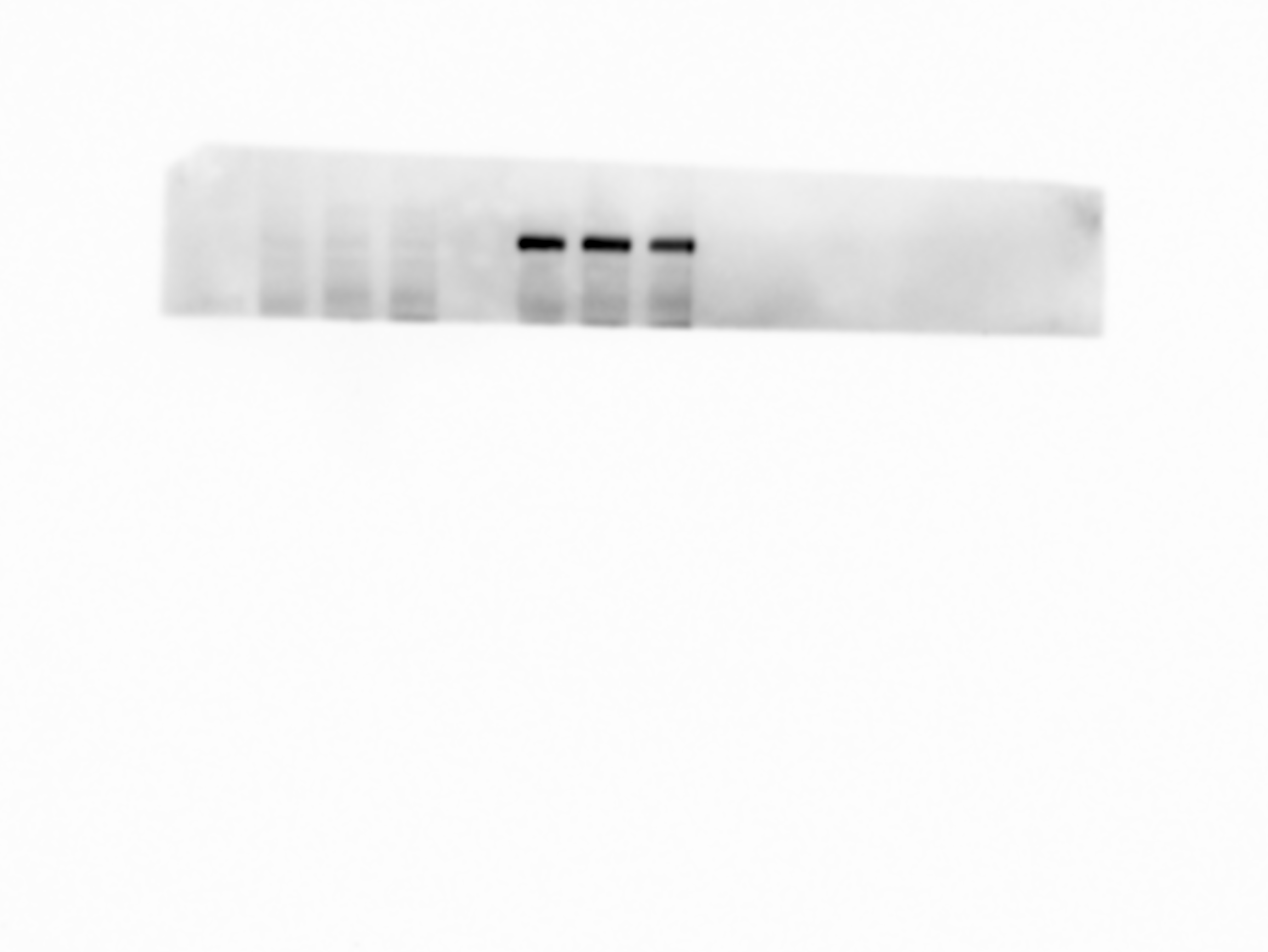

Supplement: Supplementary file 5 [file DataSheet3.zip › Fig5D HA-IRF3 Input Actin.tif]

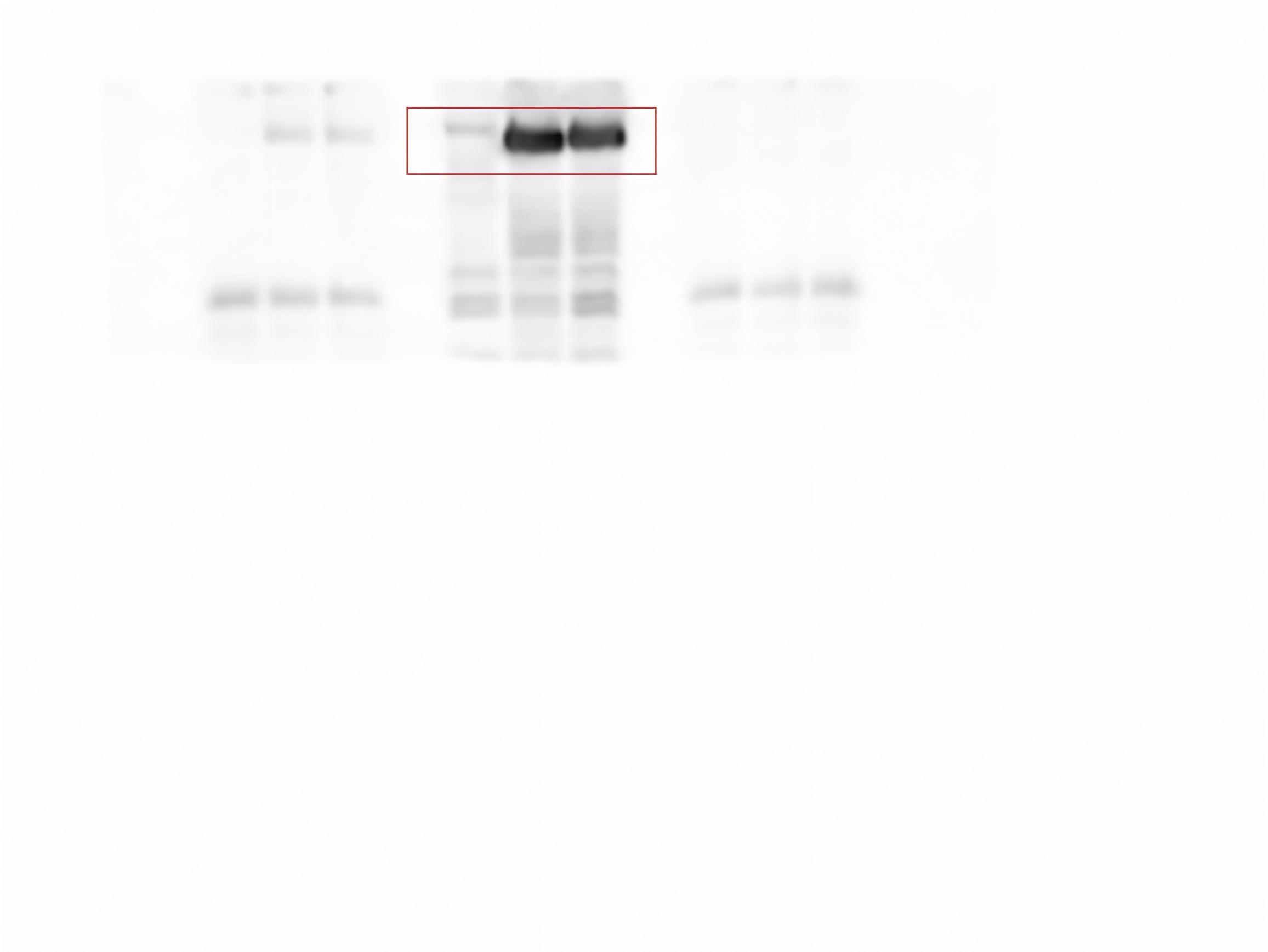

Supplement: Supplementary file 5 [file DataSheet3.zip › Fig5D HA-IRF3 Input HA edited showing band.jpg]

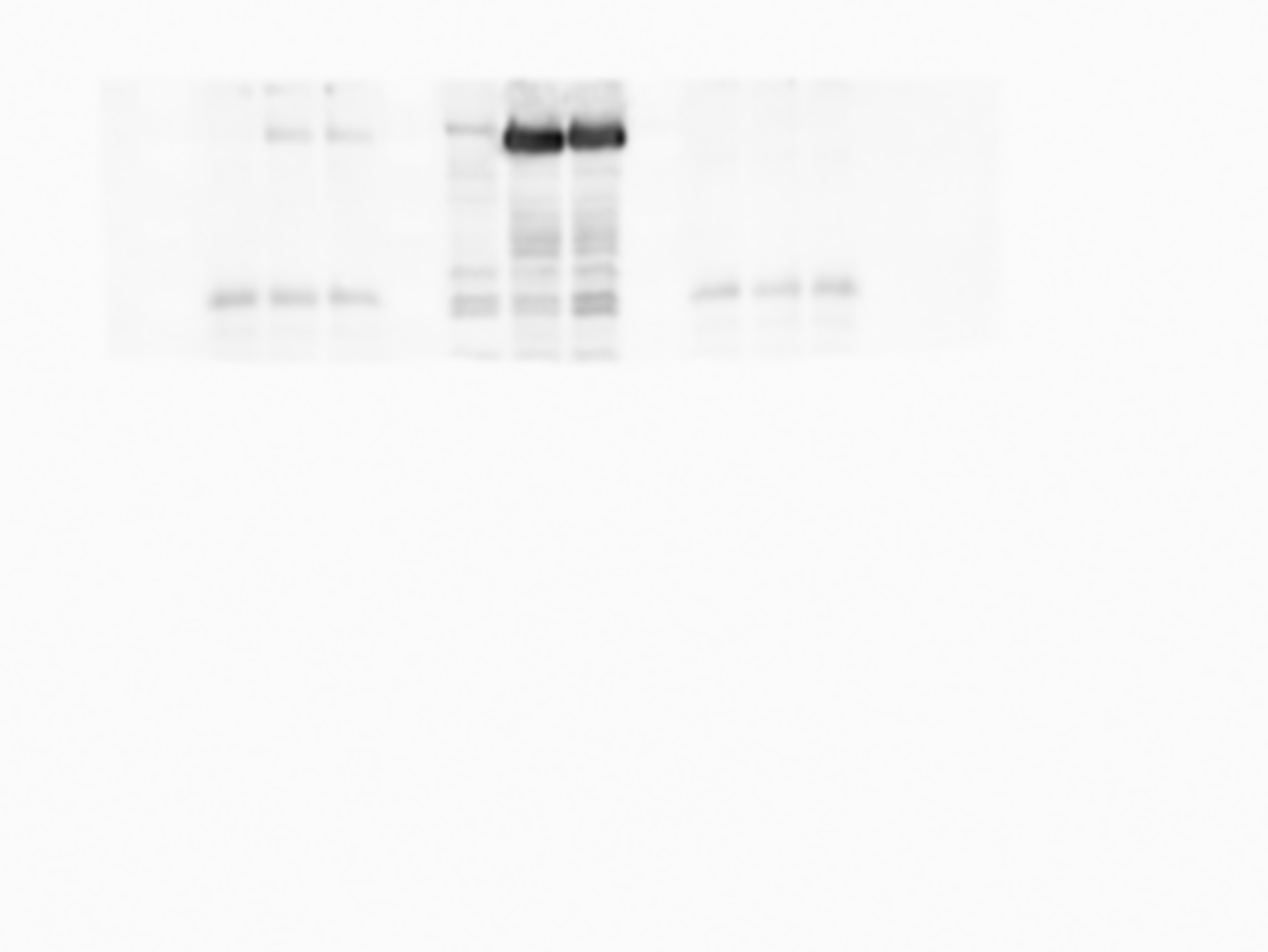

Supplement: Supplementary file 5 [file DataSheet3.zip › Fig5D HA-IRF3 Input HA.tif]

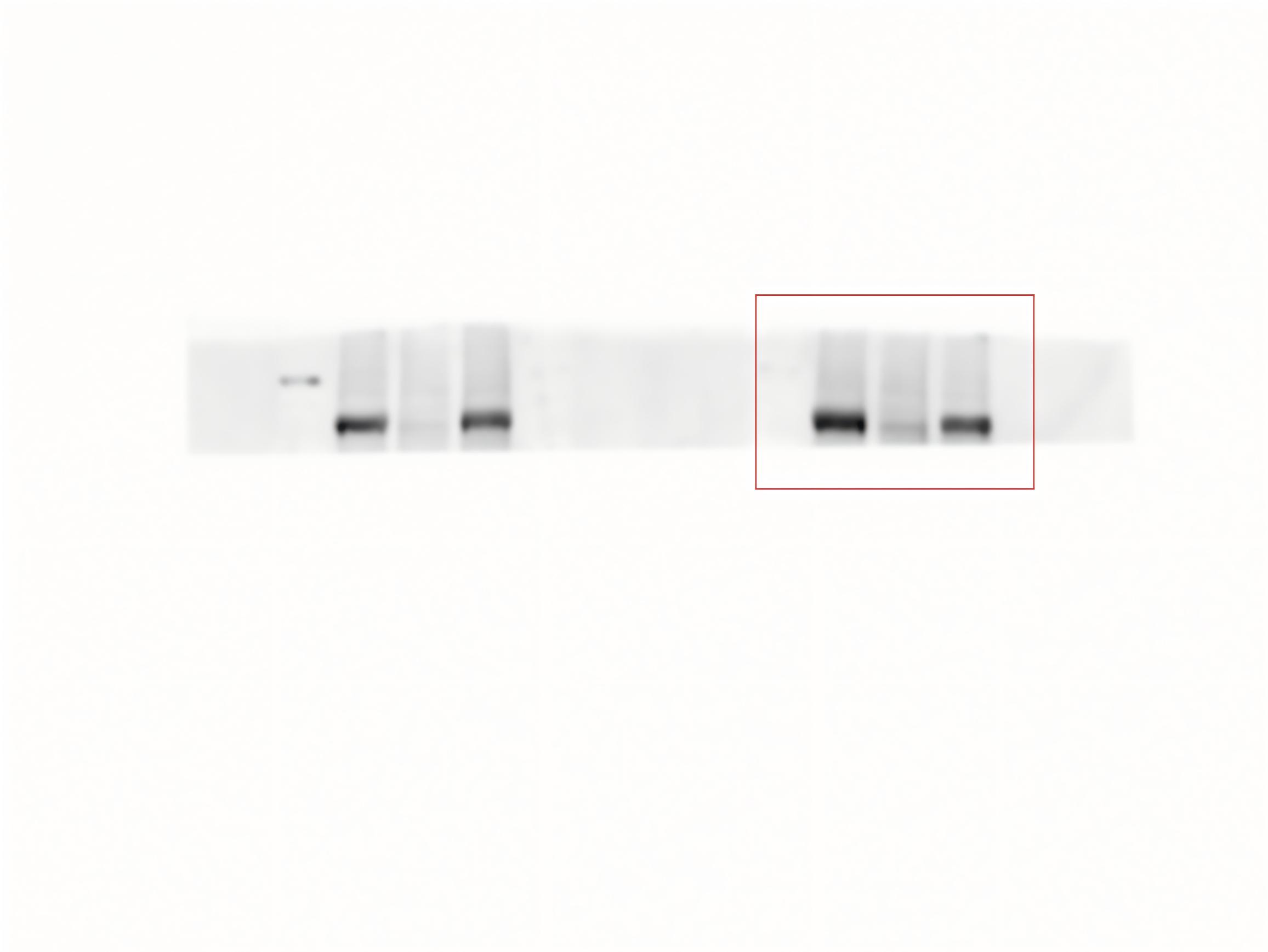

Supplement: Supplementary file 5 [file DataSheet3.zip › Fig5D HA-IRF3 Input Myc edited showing band.jpg]

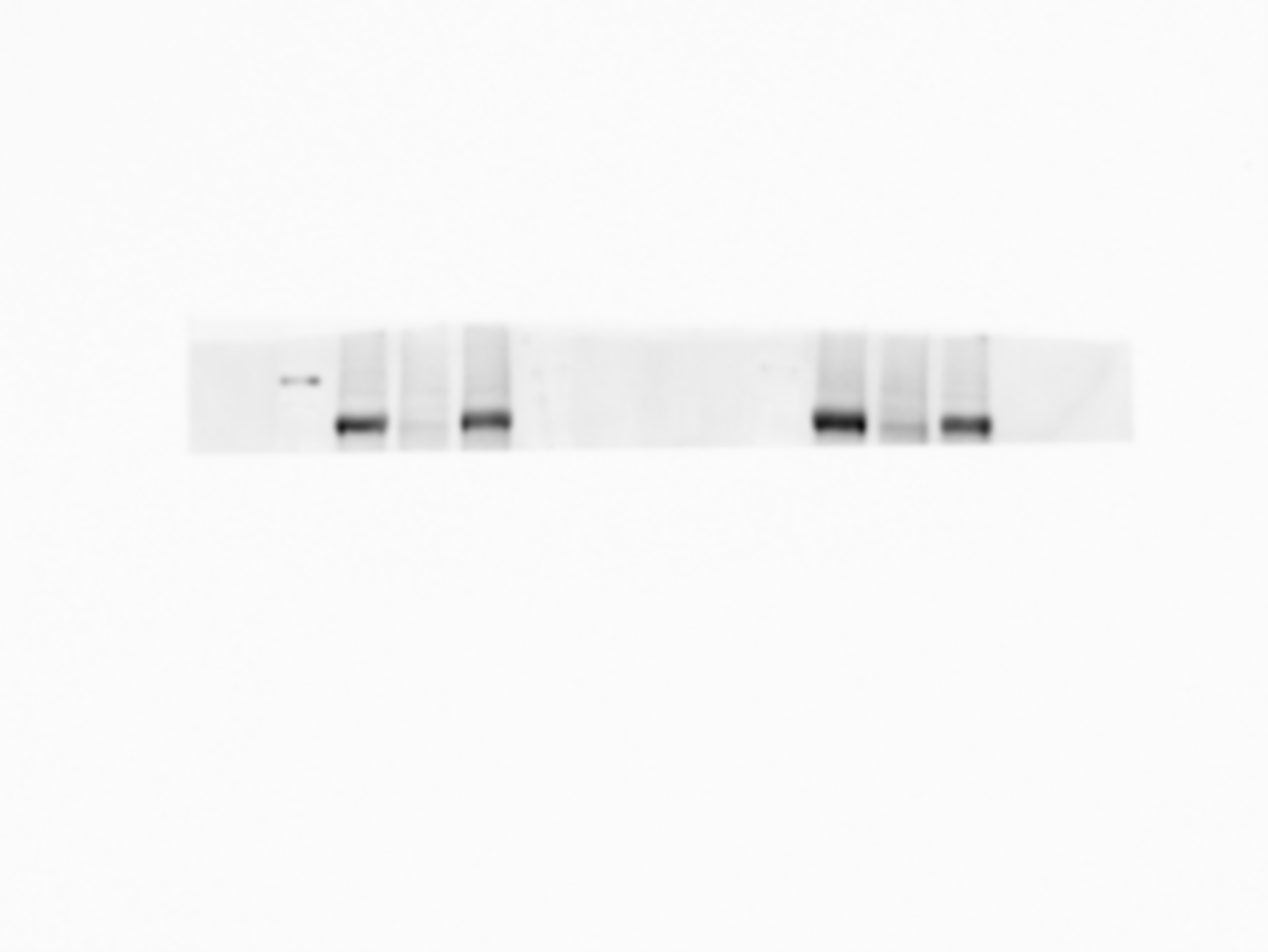

Supplement: Supplementary file 5 [file DataSheet3.zip › Fig5D HA-IRF3 Input Myc.tif]

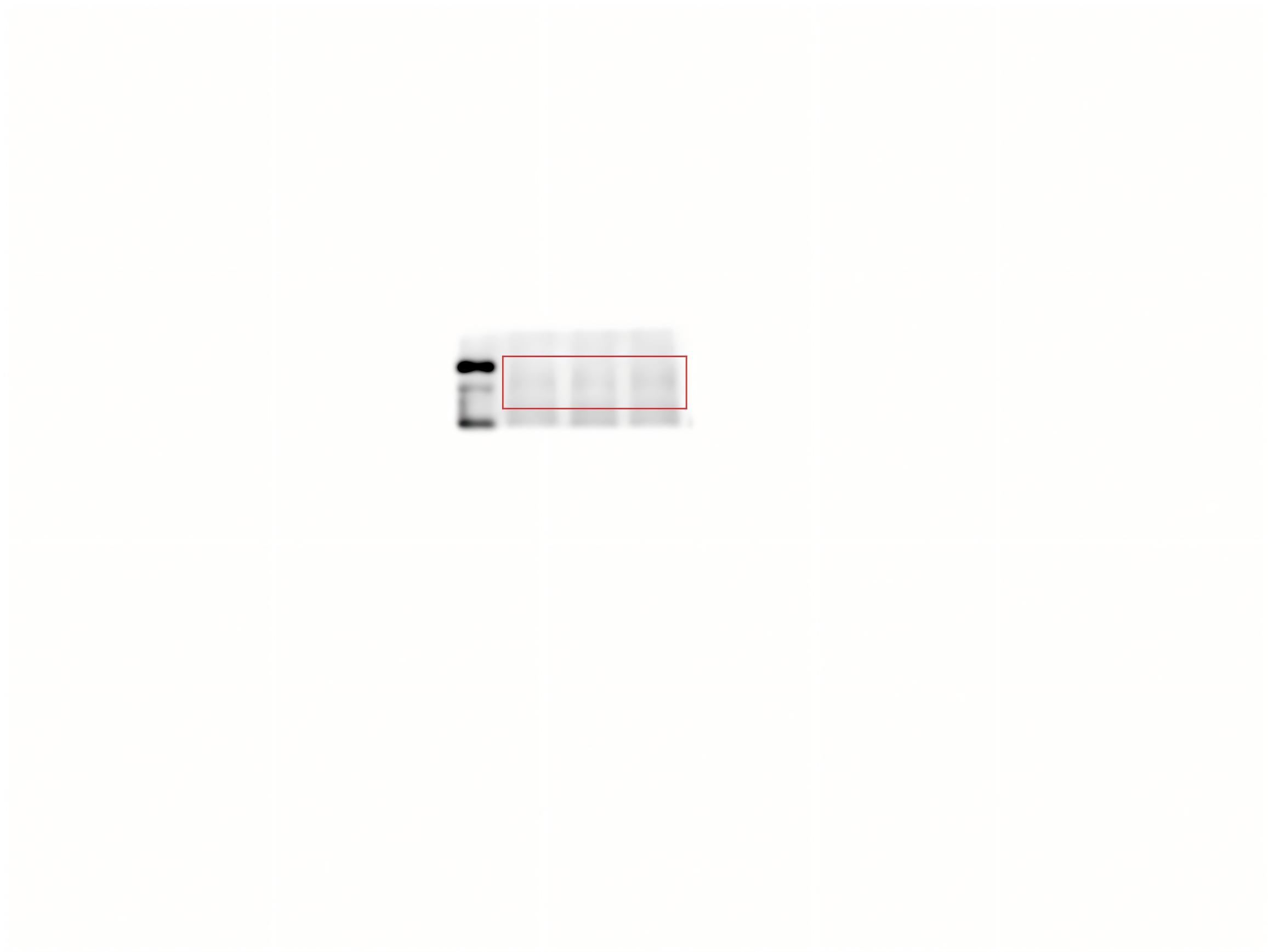

Supplement: Supplementary file 5 [file DataSheet3.zip › Fig5D HA-IRF3 IP HA edited showing band.jpg]

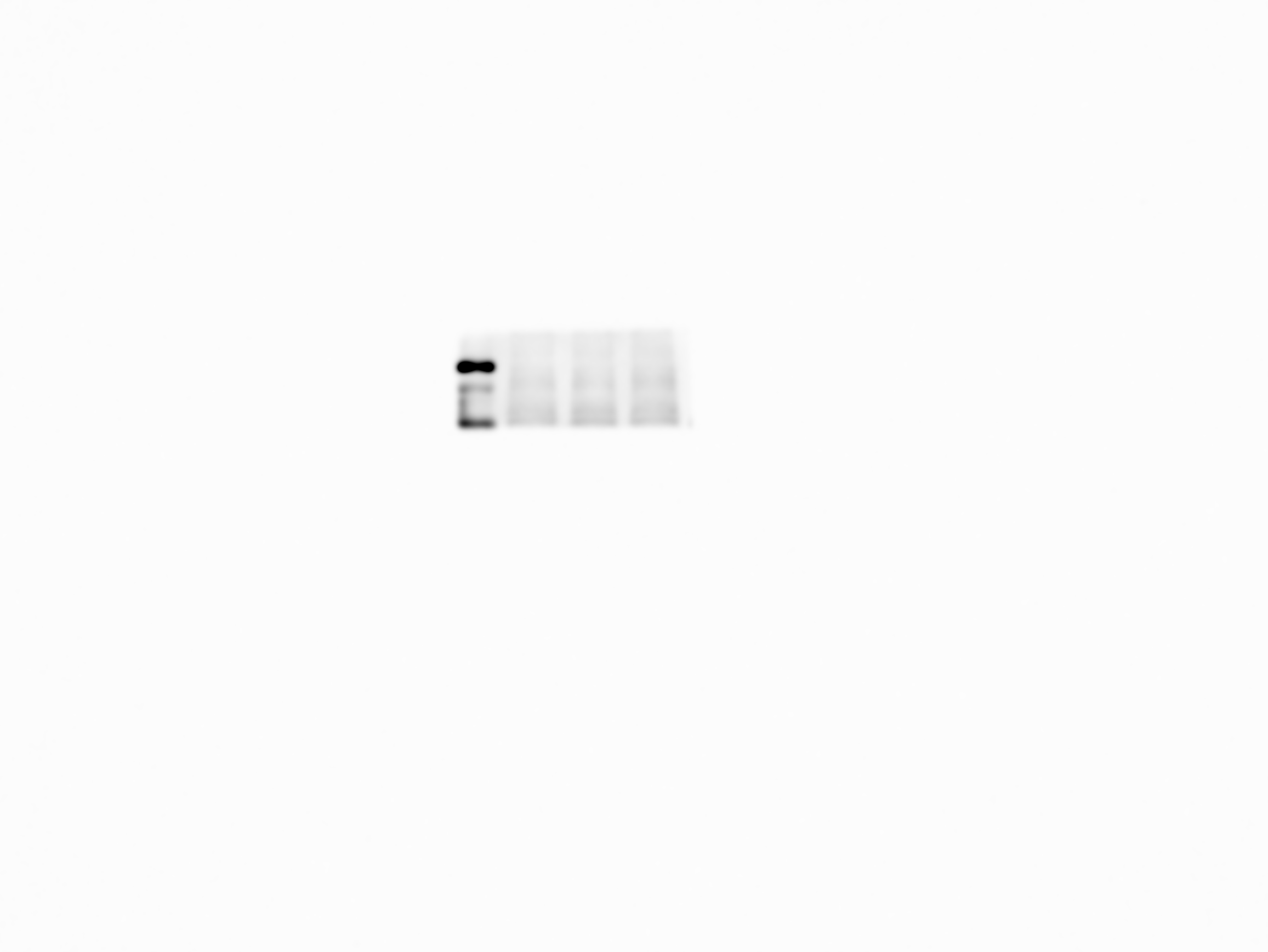

Supplement: Supplementary file 5 [file DataSheet3.zip › Fig5D HA-IRF3 IP HA.tif]

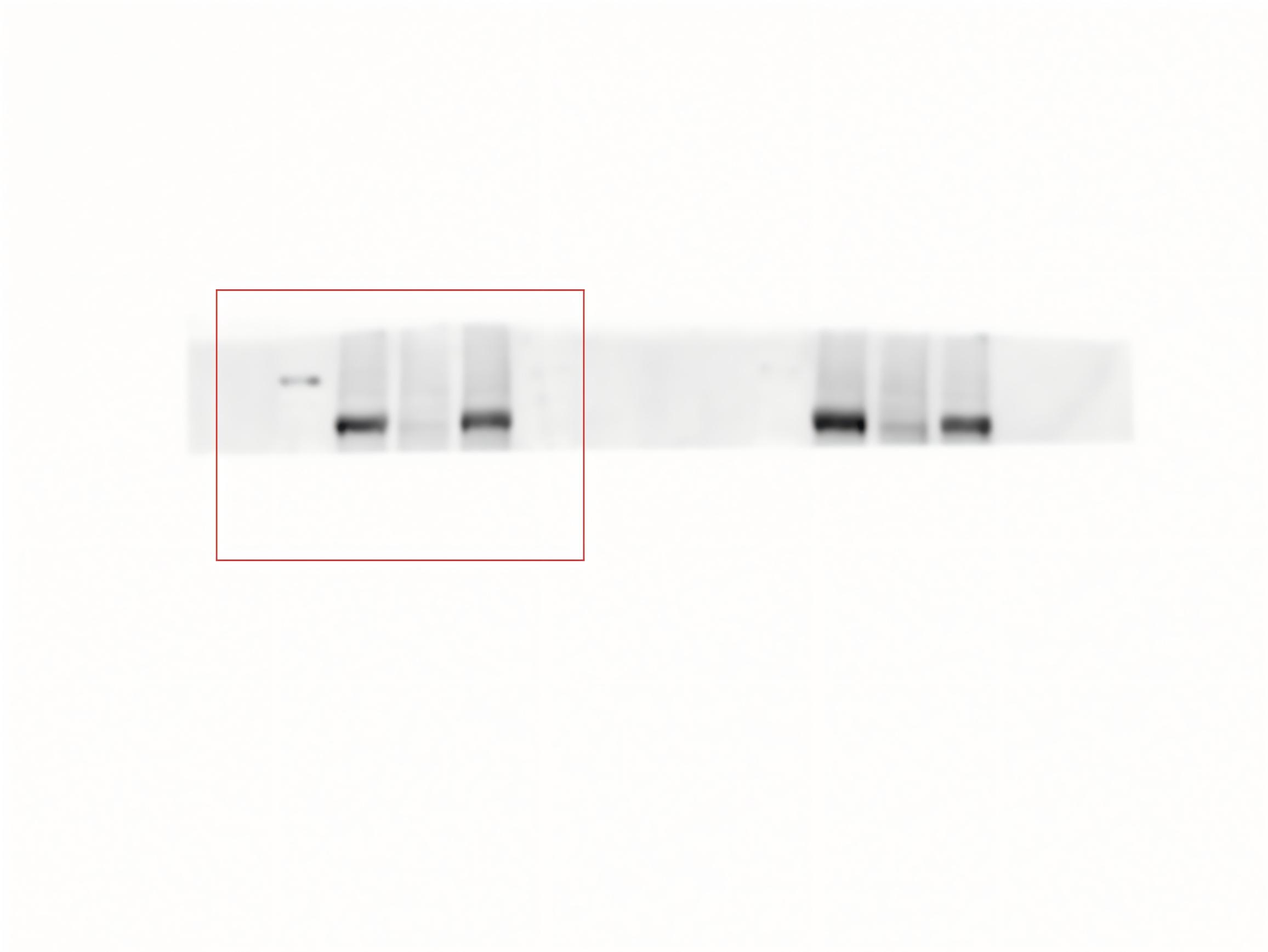

Supplement: Supplementary file 5 [file DataSheet3.zip › Fig5D HA-IRF3 IP Myc edited showing band.jpg]

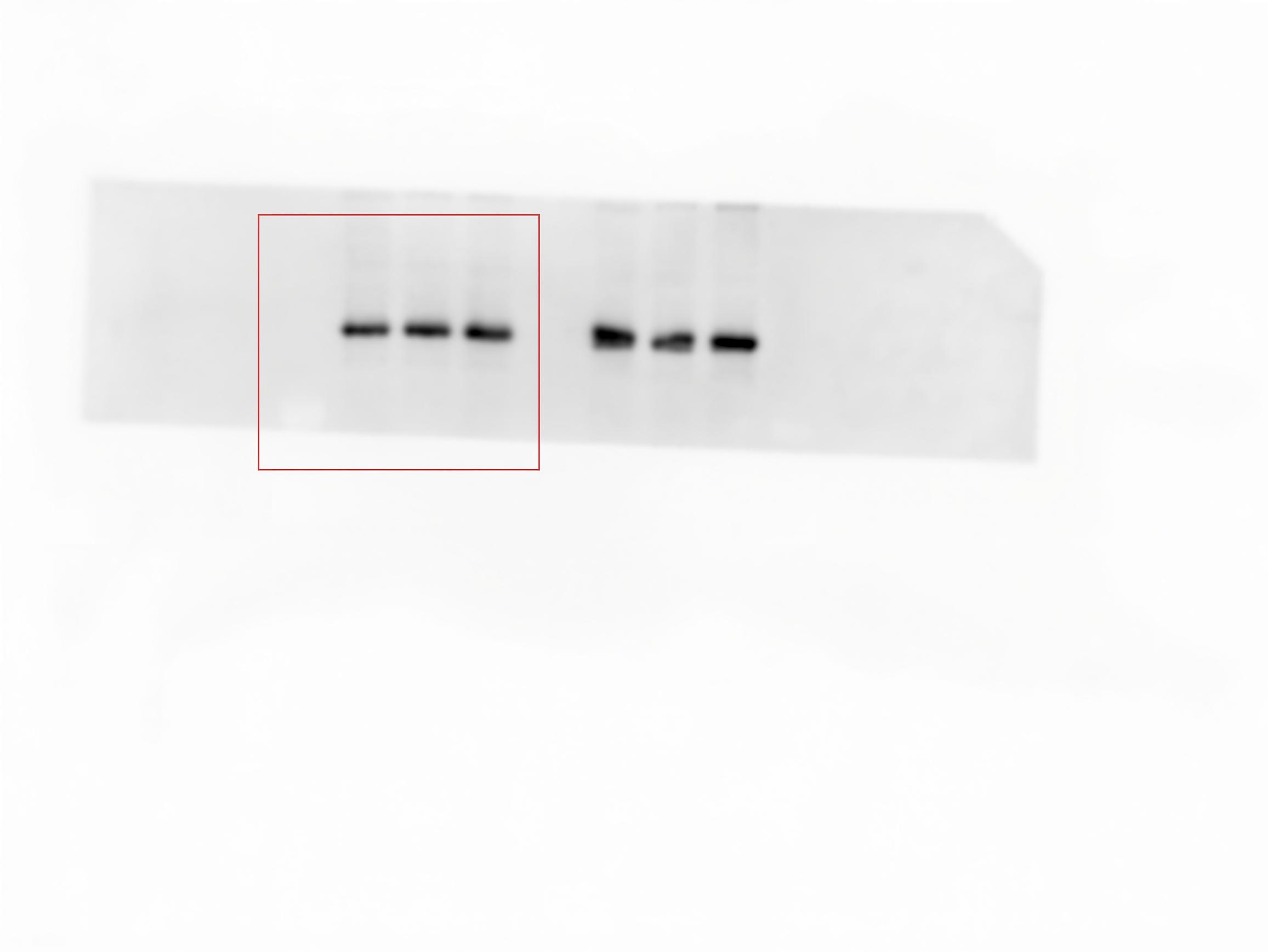

Supplement: Supplementary file 5 [file DataSheet3.zip › Fig5D HA-IRF7 Input Actin edited showing band.jpg]

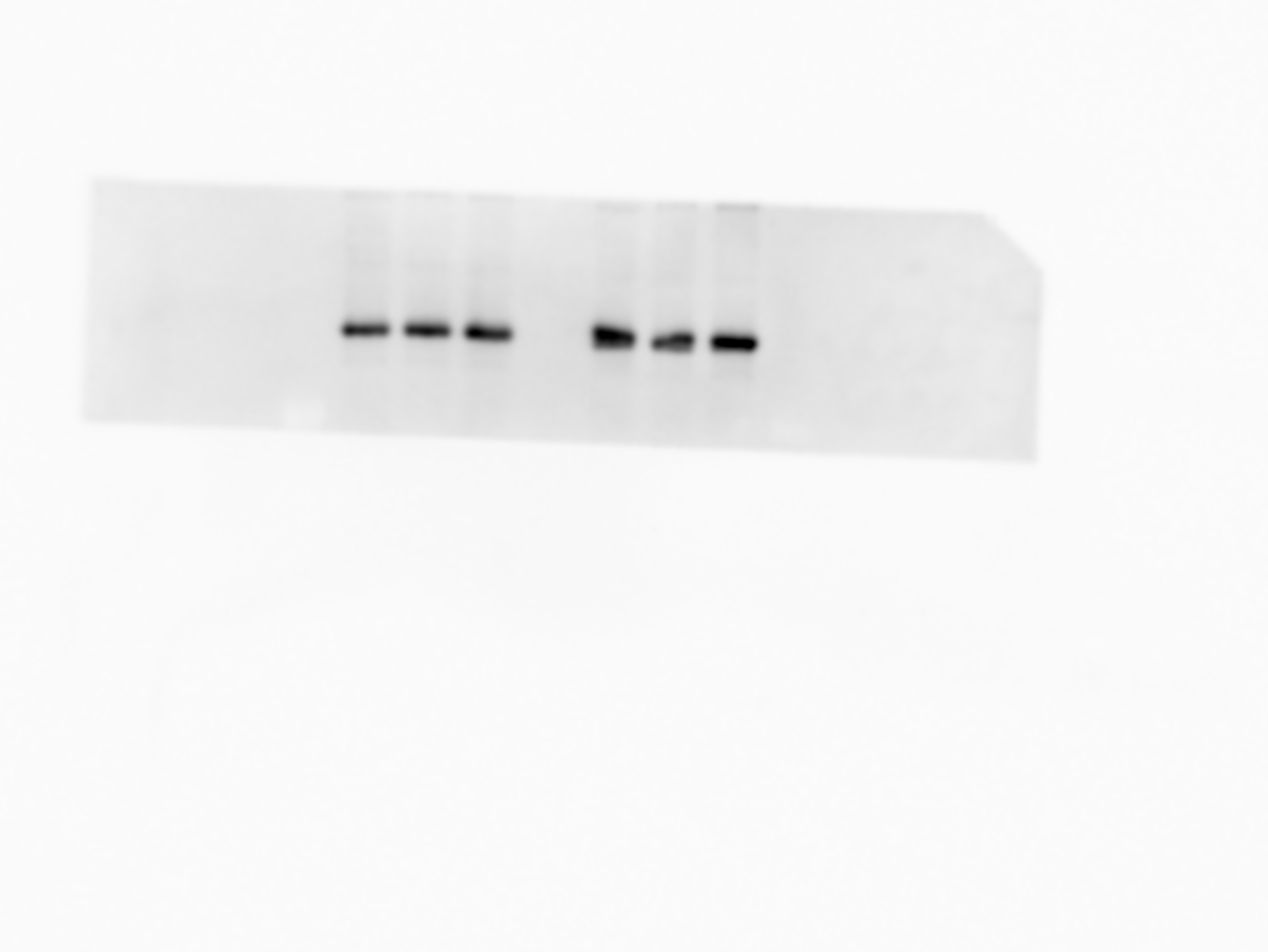

Supplement: Supplementary file 5 [file DataSheet3.zip › Fig5D HA-IRF7 Input Actin.tif]

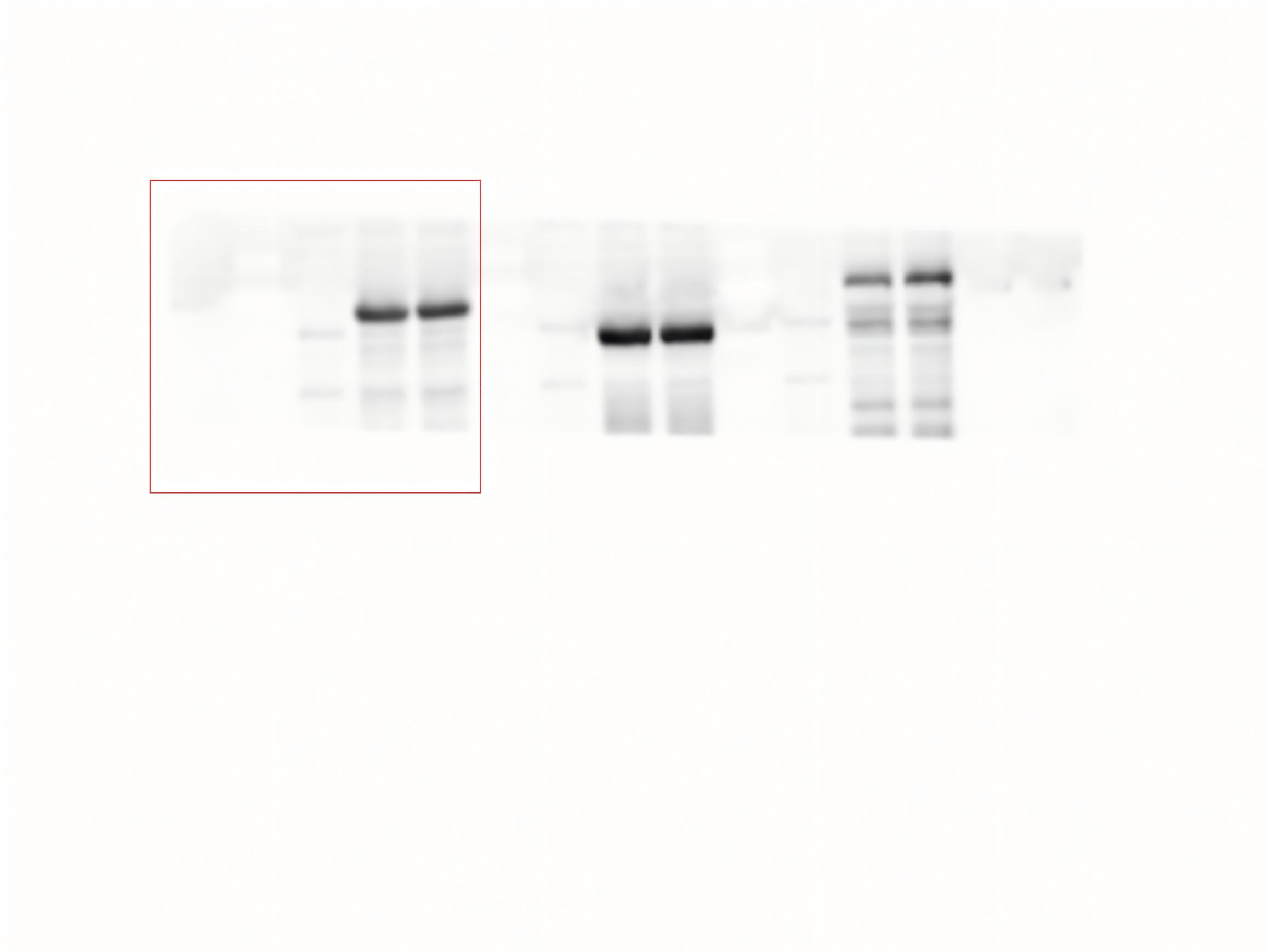

Supplement: Supplementary file 5 [file DataSheet3.zip › Fig5D HA-IRF7 Input HA edited showing band.jpg]

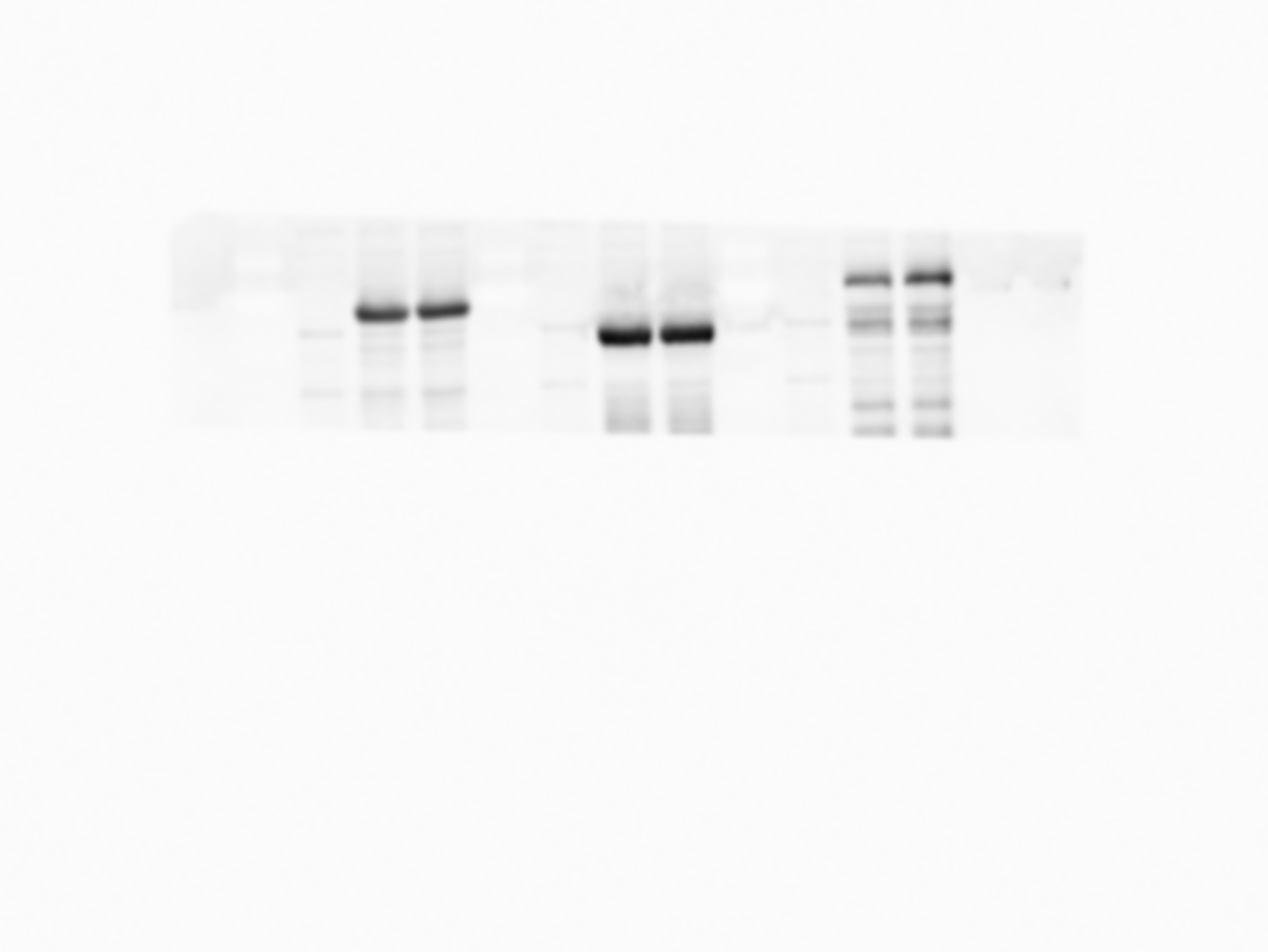

Supplement: Supplementary file 5 [file DataSheet3.zip › Fig5D HA-IRF7 Input HA.tif]

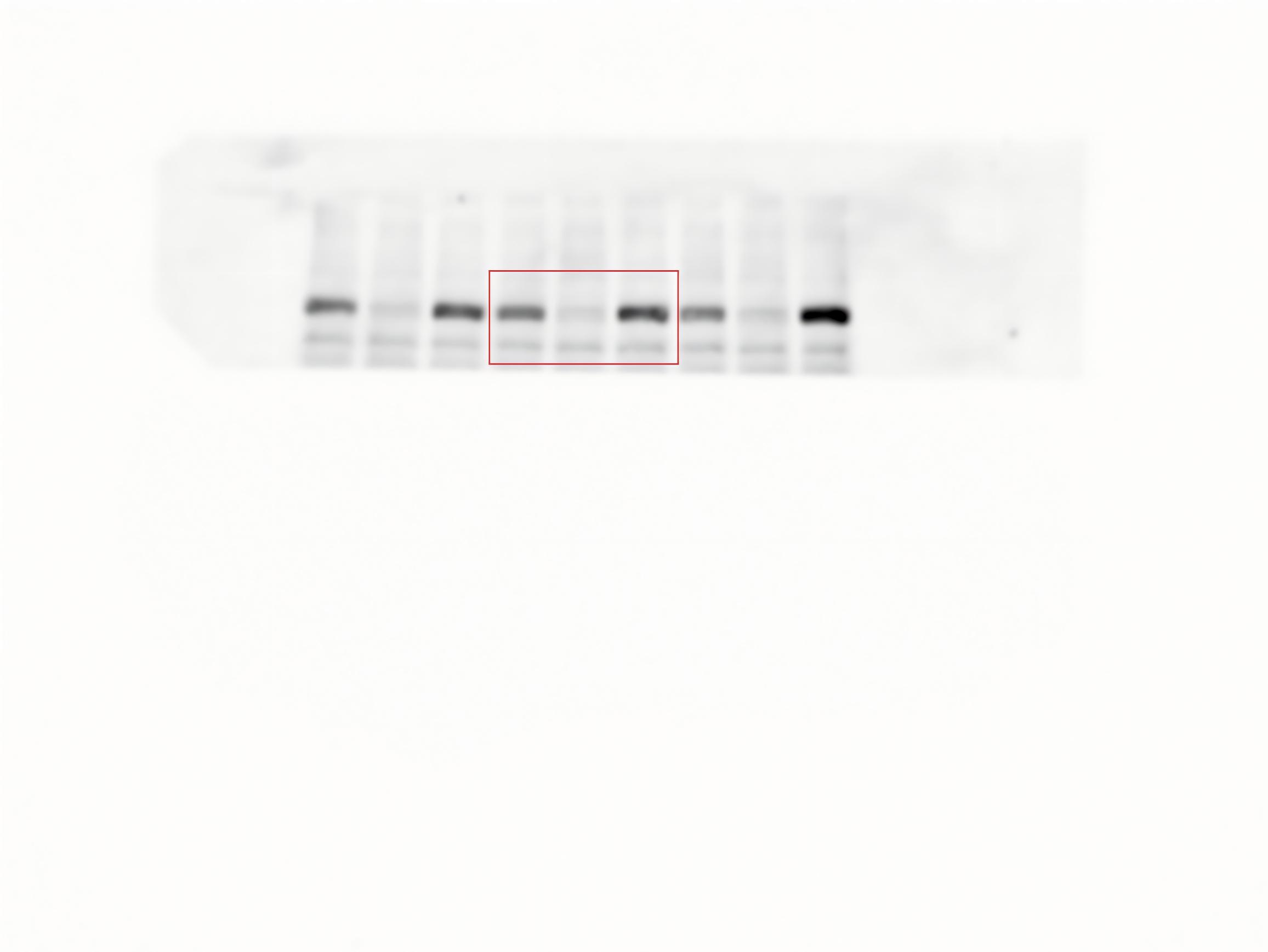

Supplement: Supplementary file 5 [file DataSheet3.zip › Fig5D HA-IRF7 Input Myc edited showing band.jpg]

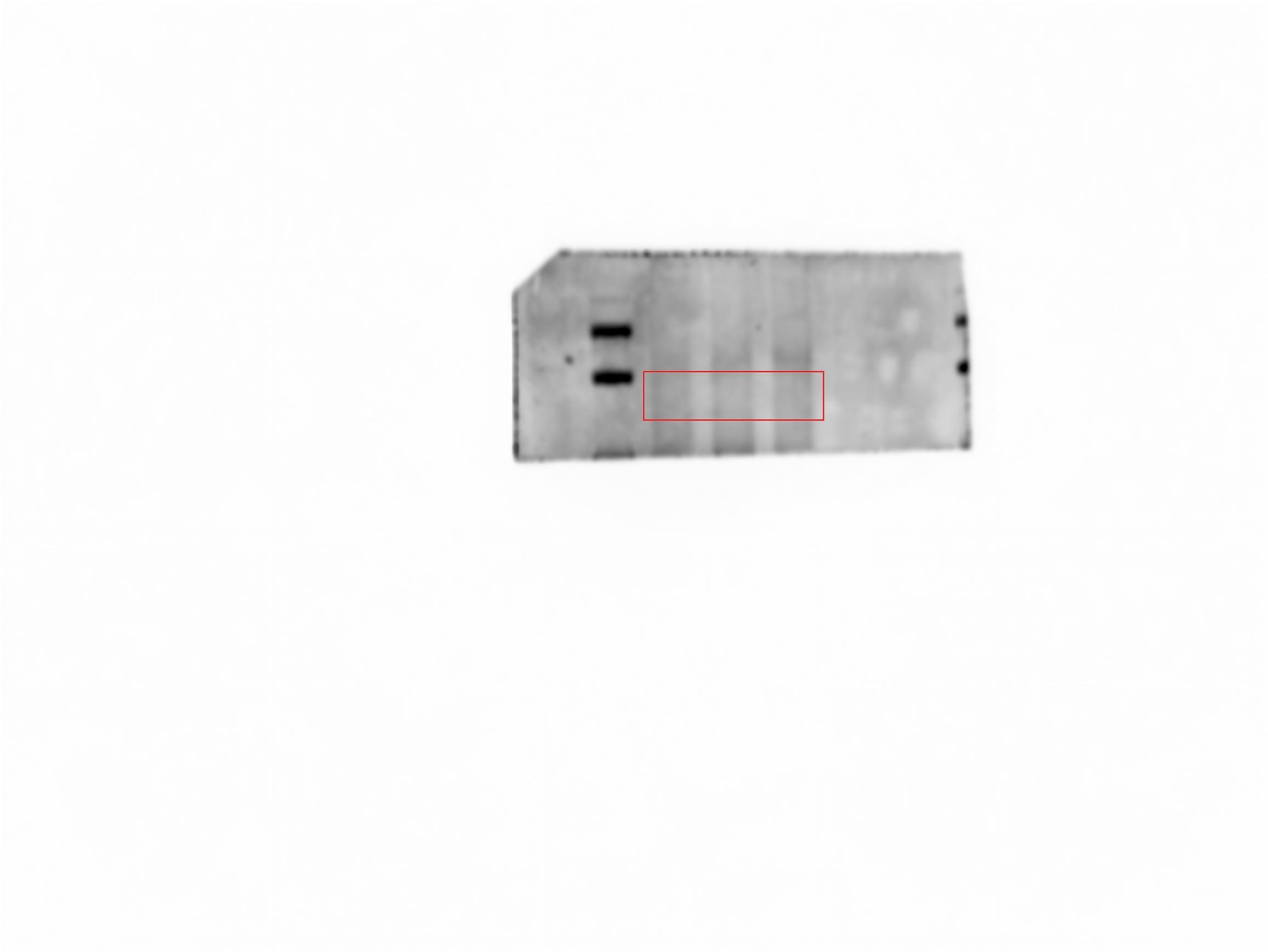

Supplement: Supplementary file 5 [file DataSheet3.zip › Fig5D HA-IRF7 IP HA edited showing band.jpg]

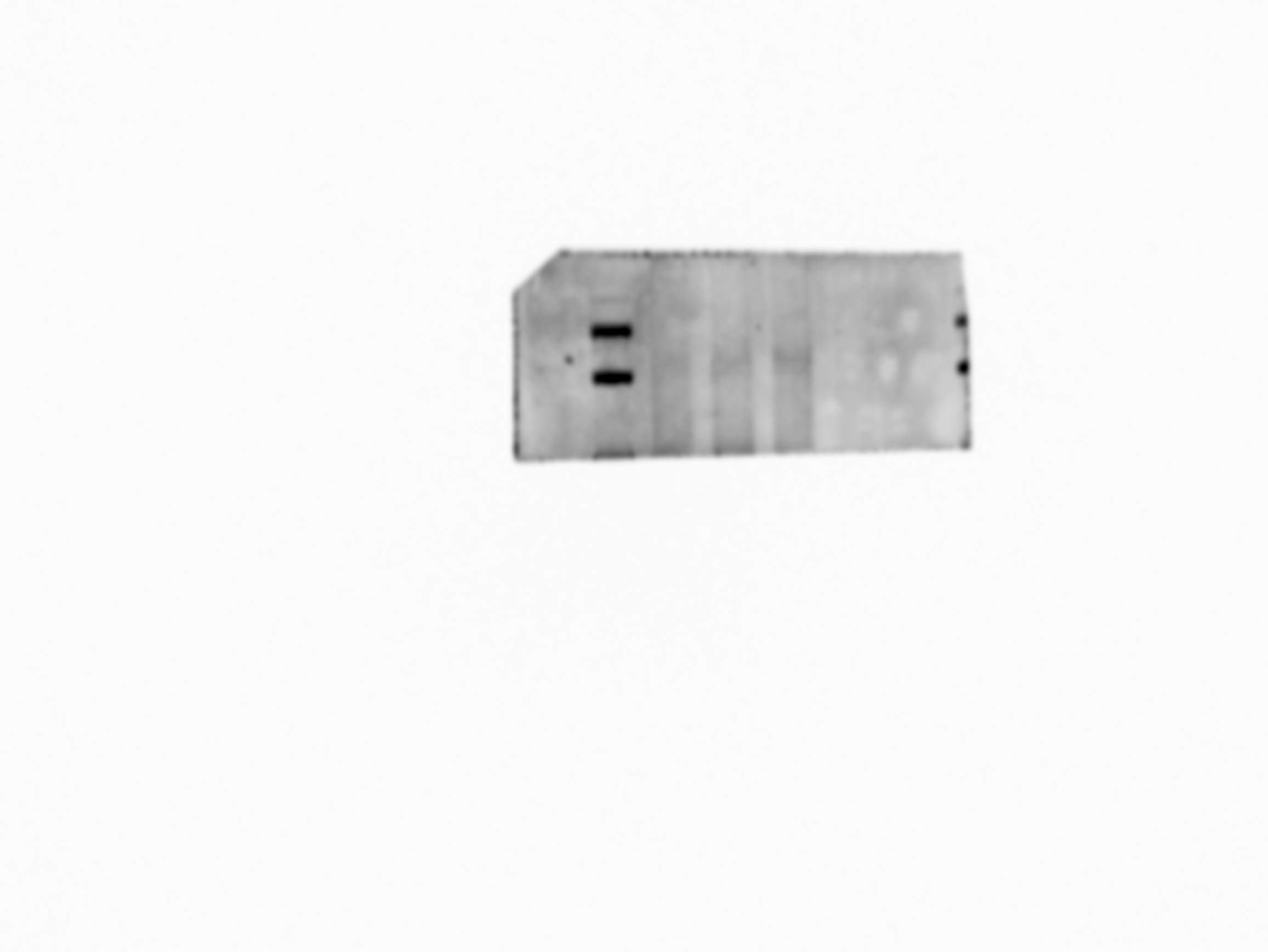

Supplement: Supplementary file 5 [file DataSheet3.zip › Fig5D HA-IRF7 IP HA.tif]

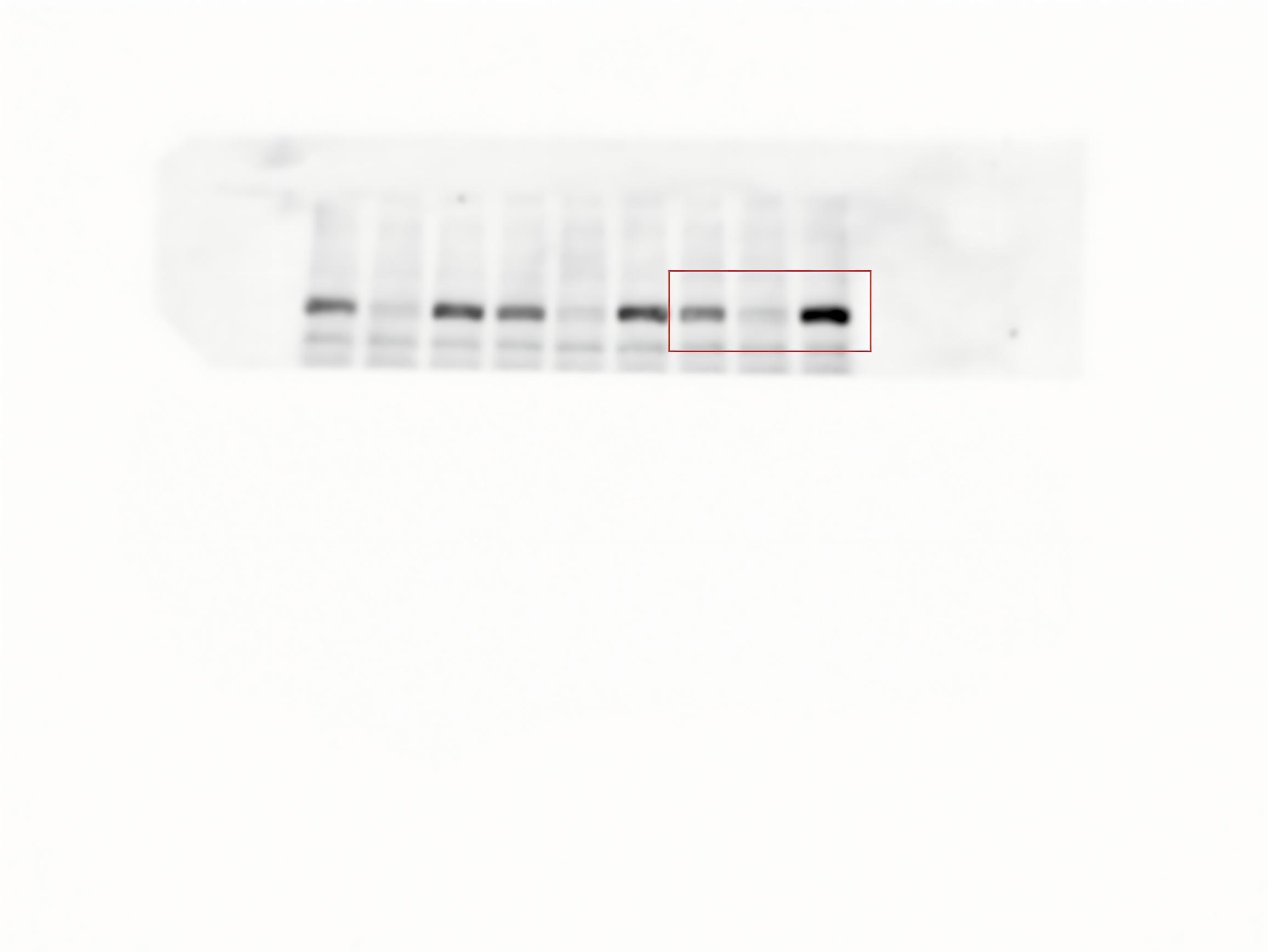

Supplement: Supplementary file 5 [file DataSheet3.zip › Fig5D HA-IRF7 IP Myc edited showing band.jpg]

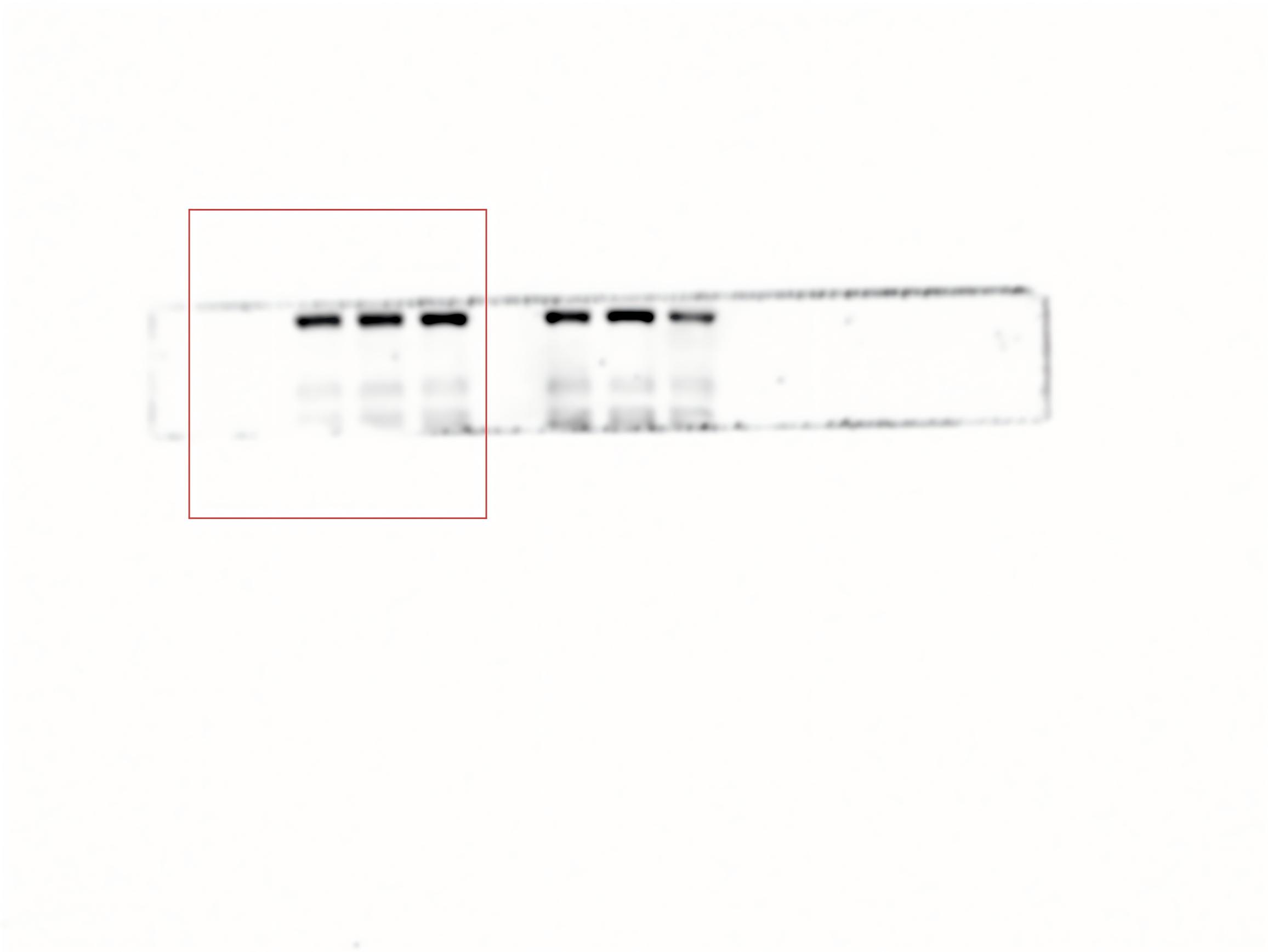

Supplement: Supplementary file 5 [file DataSheet3.zip › Fig5D HA-MDA5 Input Actin edited showing band.jpg]

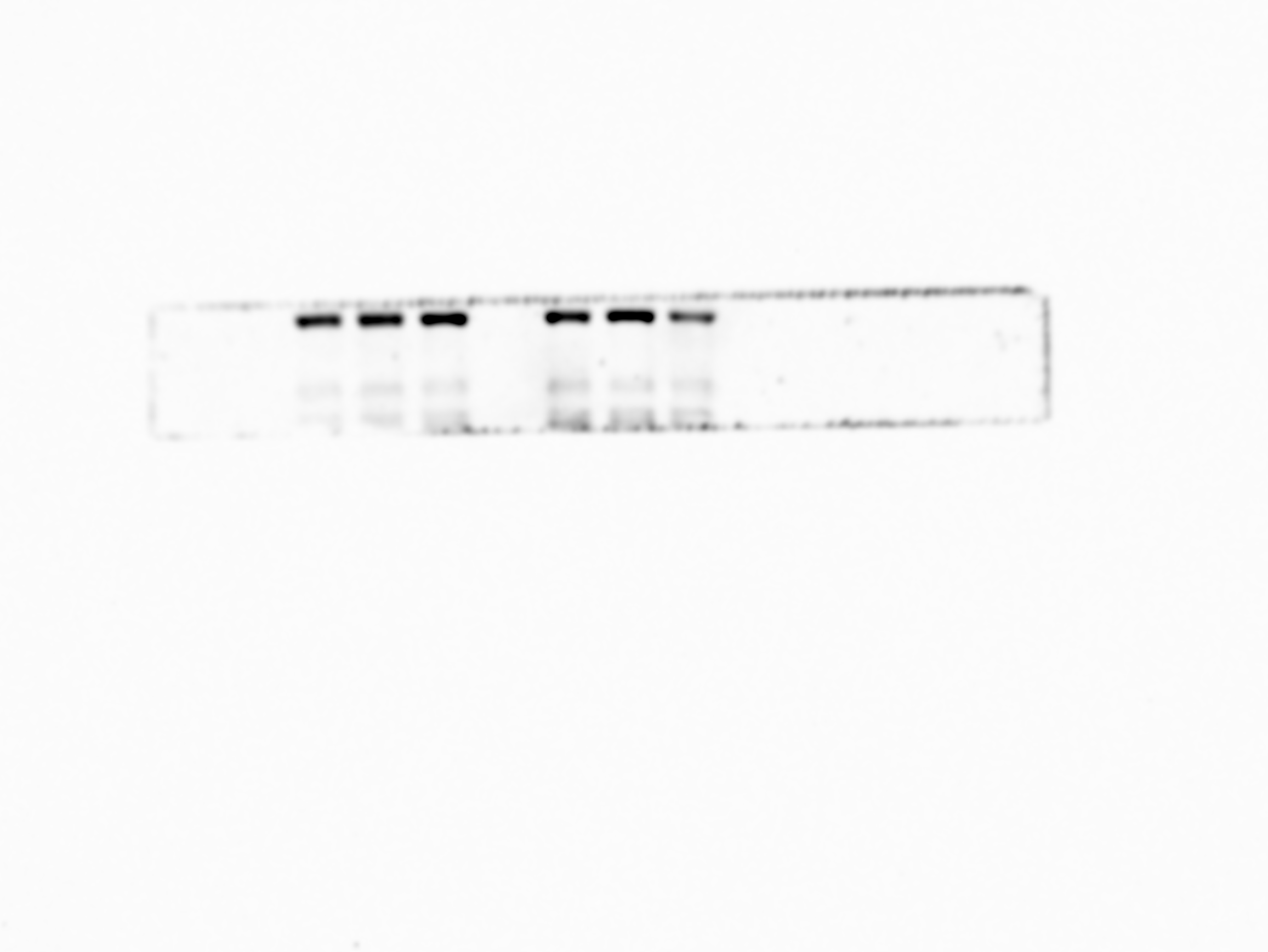

Supplement: Supplementary file 5 [file DataSheet3.zip › Fig5D HA-MDA5 Input Actin.tif]

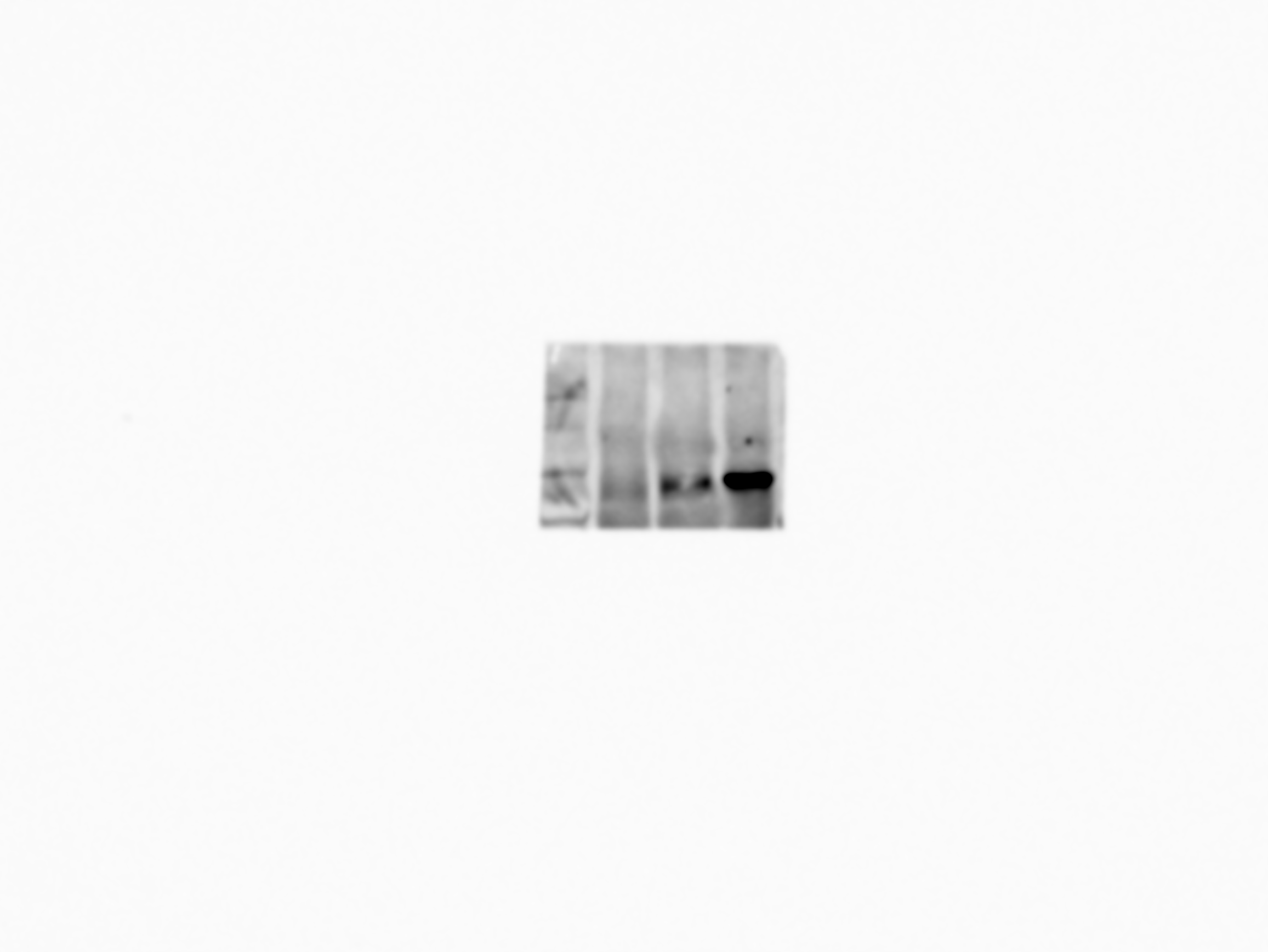

Supplement: Supplementary file 5 [file DataSheet3.zip › Fig5D HA-MDA5 Input HA.tif]

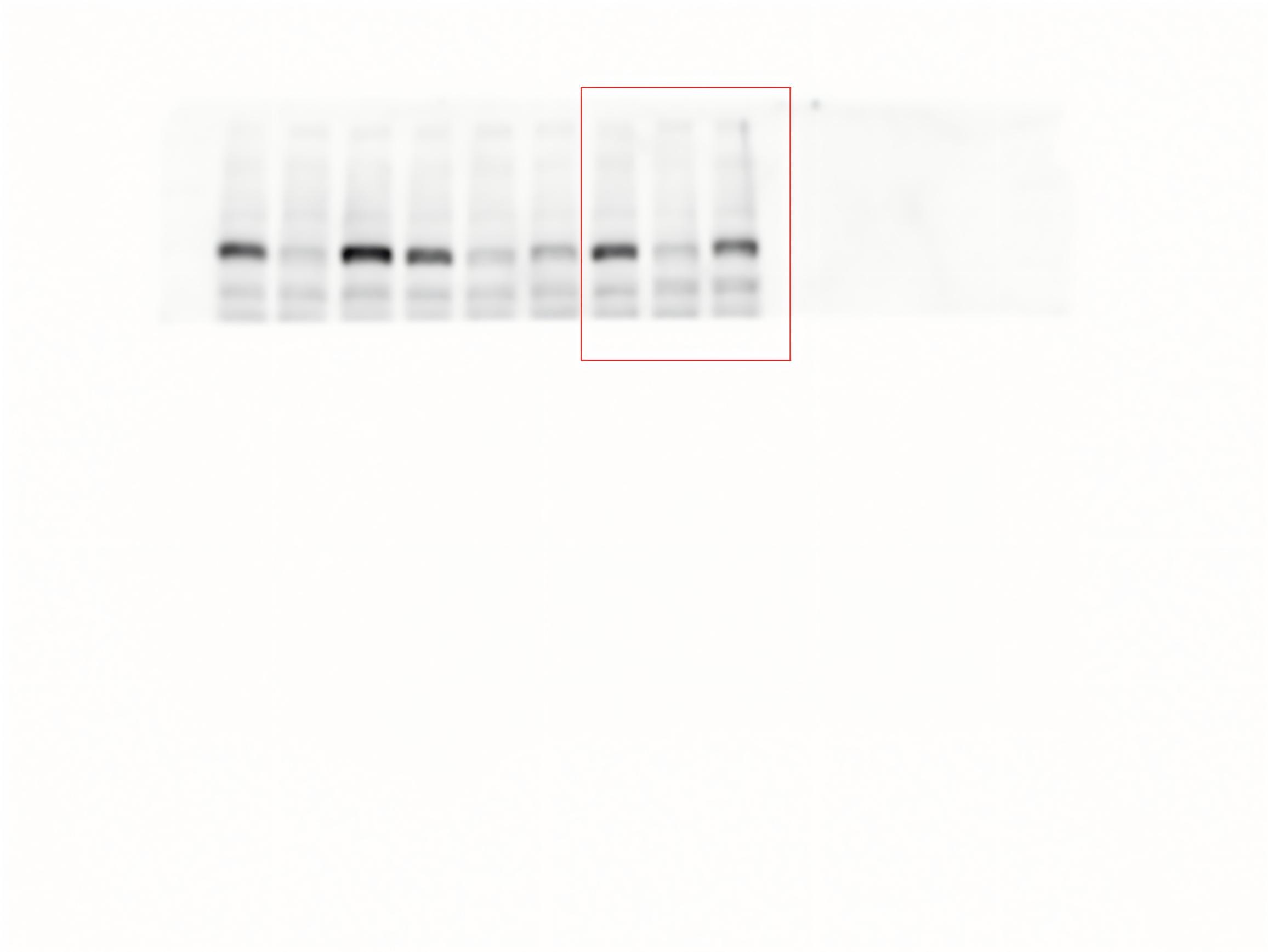

Supplement: Supplementary file 5 [file DataSheet3.zip › Fig5D HA-MDA5 Input Myc edited showing band.jpg]

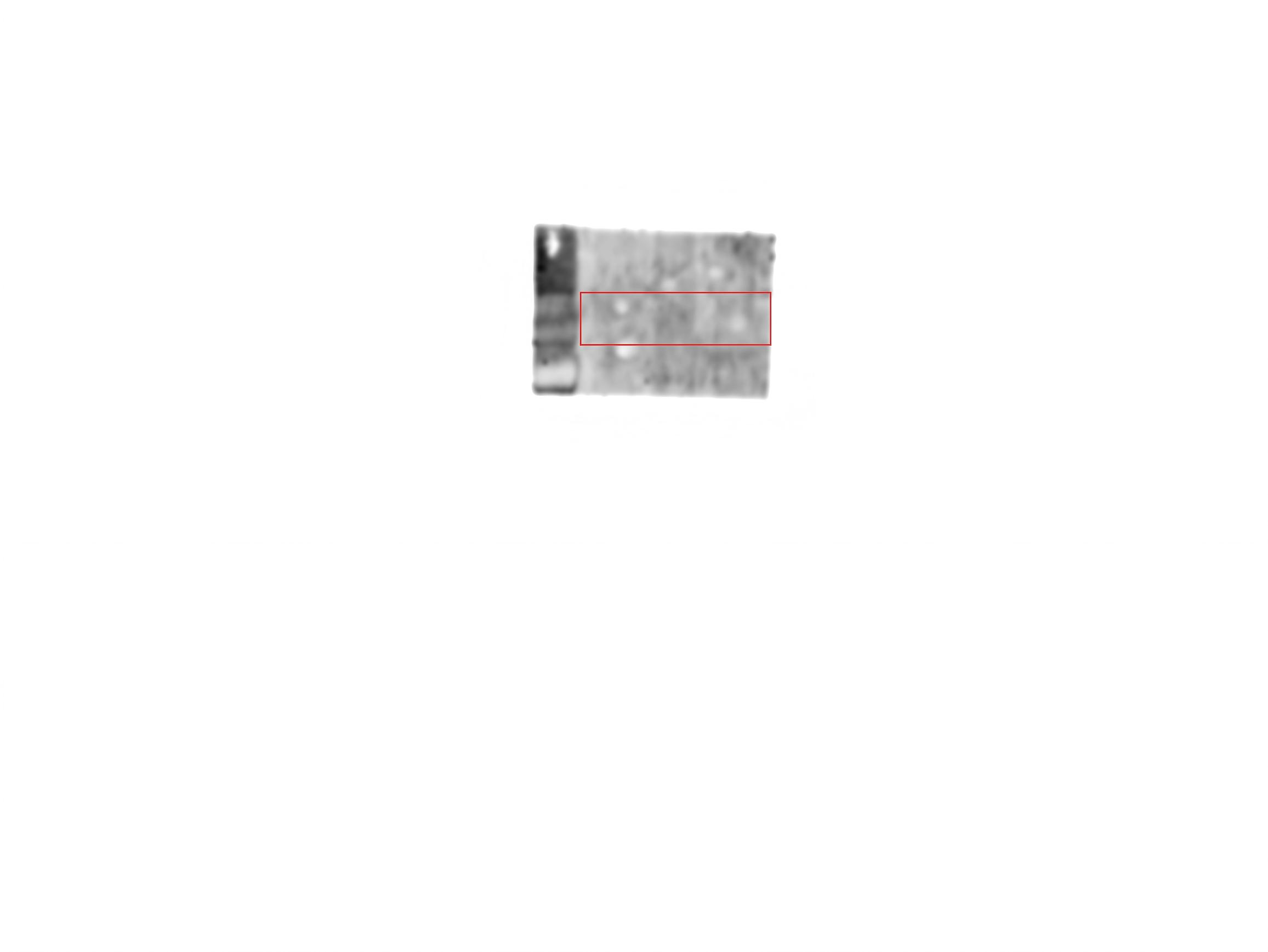

Supplement: Supplementary file 5 [file DataSheet3.zip › Fig5D HA-MDA5 IP HA edited showing band.jpg]

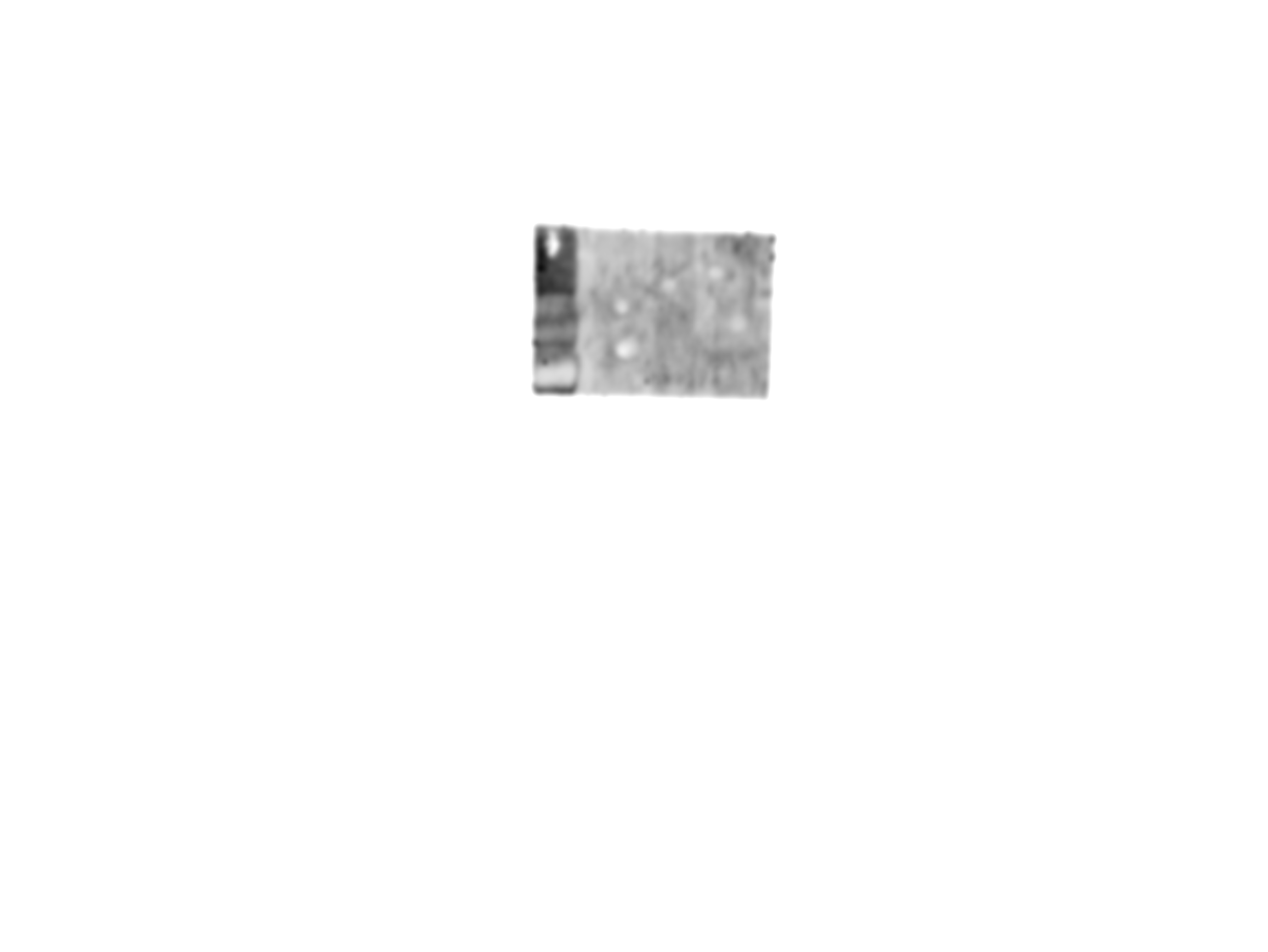

Supplement: Supplementary file 5 [file DataSheet3.zip › Fig5D HA-MDA5 IP HA.tif]

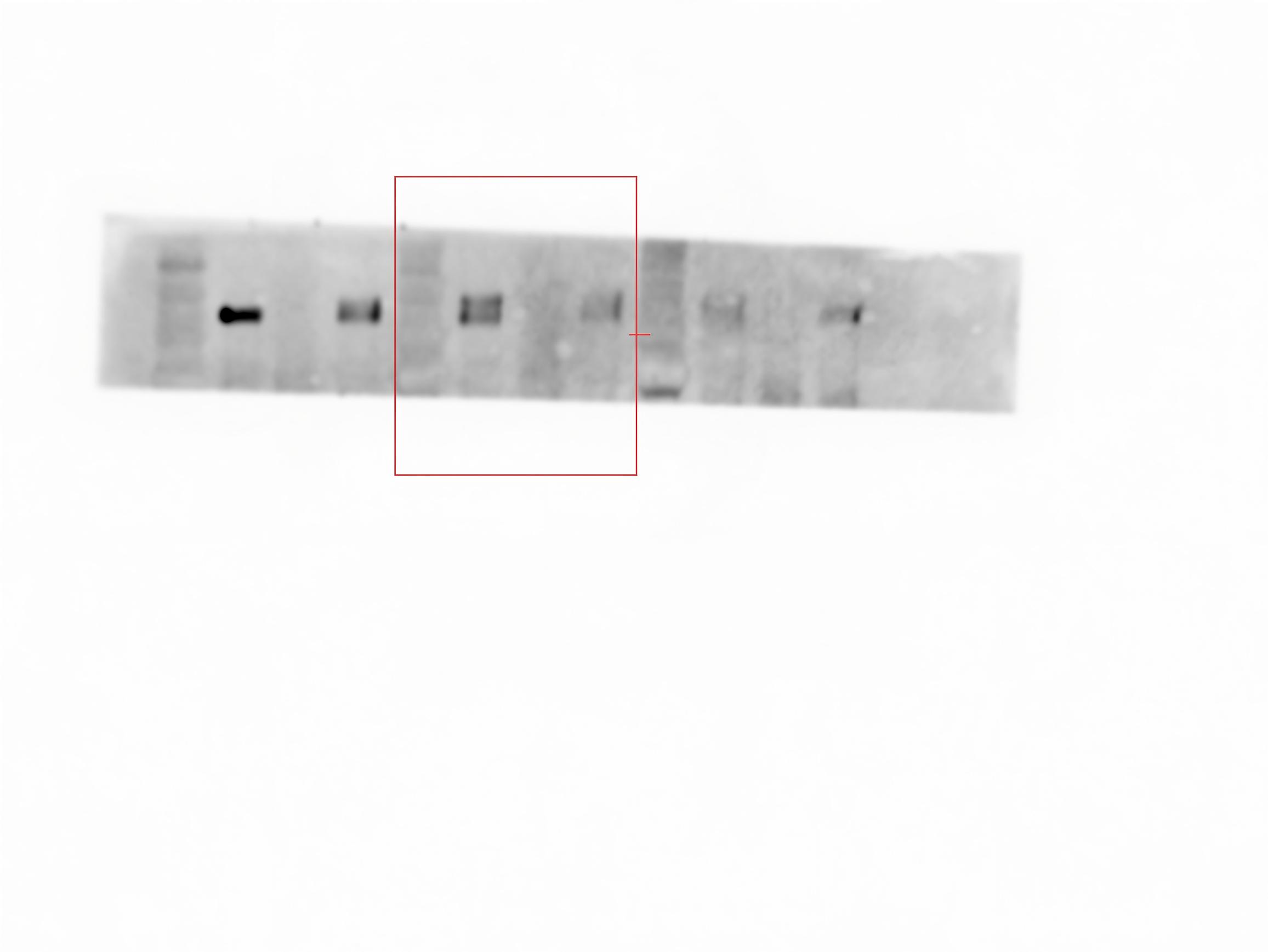

Supplement: Supplementary file 5 [file DataSheet3.zip › Fig5D HA-MDA5 IP Myc edited showing band.jpg]

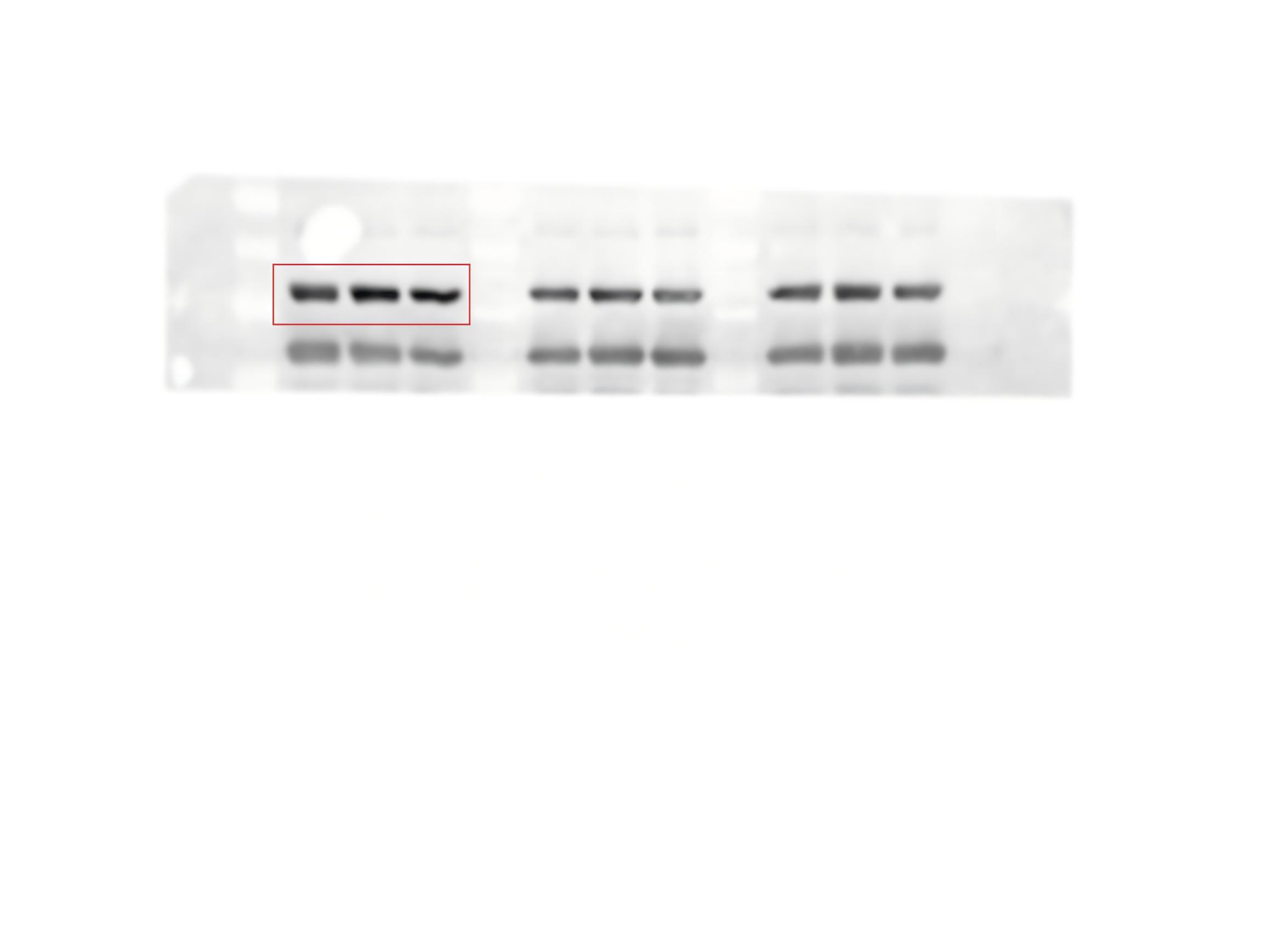

Supplement: Supplementary file 5 [file DataSheet3.zip › Fig5E left Input Actin edited showing band.jpg]

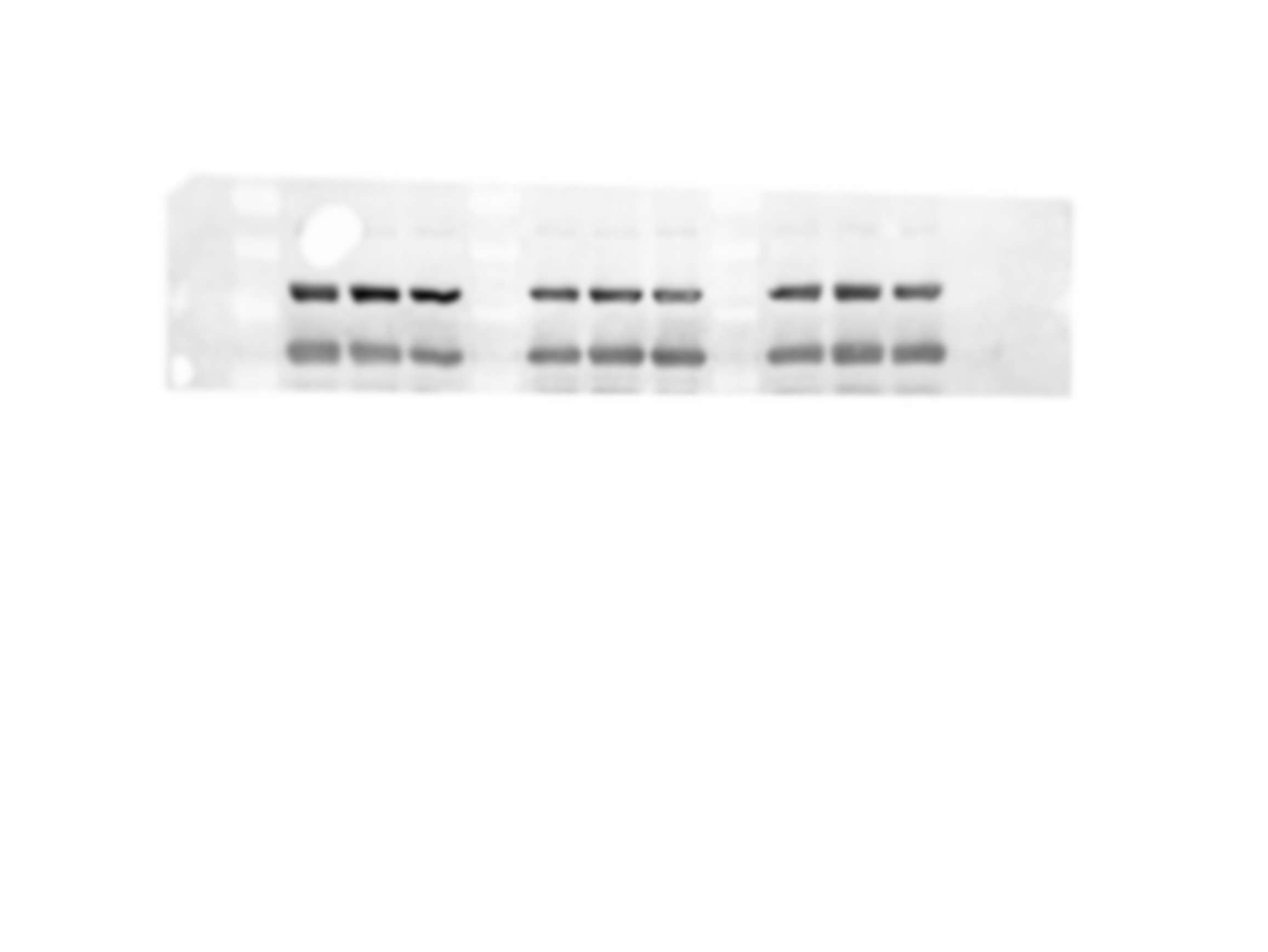

Supplement: Supplementary file 5 [file DataSheet3.zip › Fig5E left Input Actin.tif]

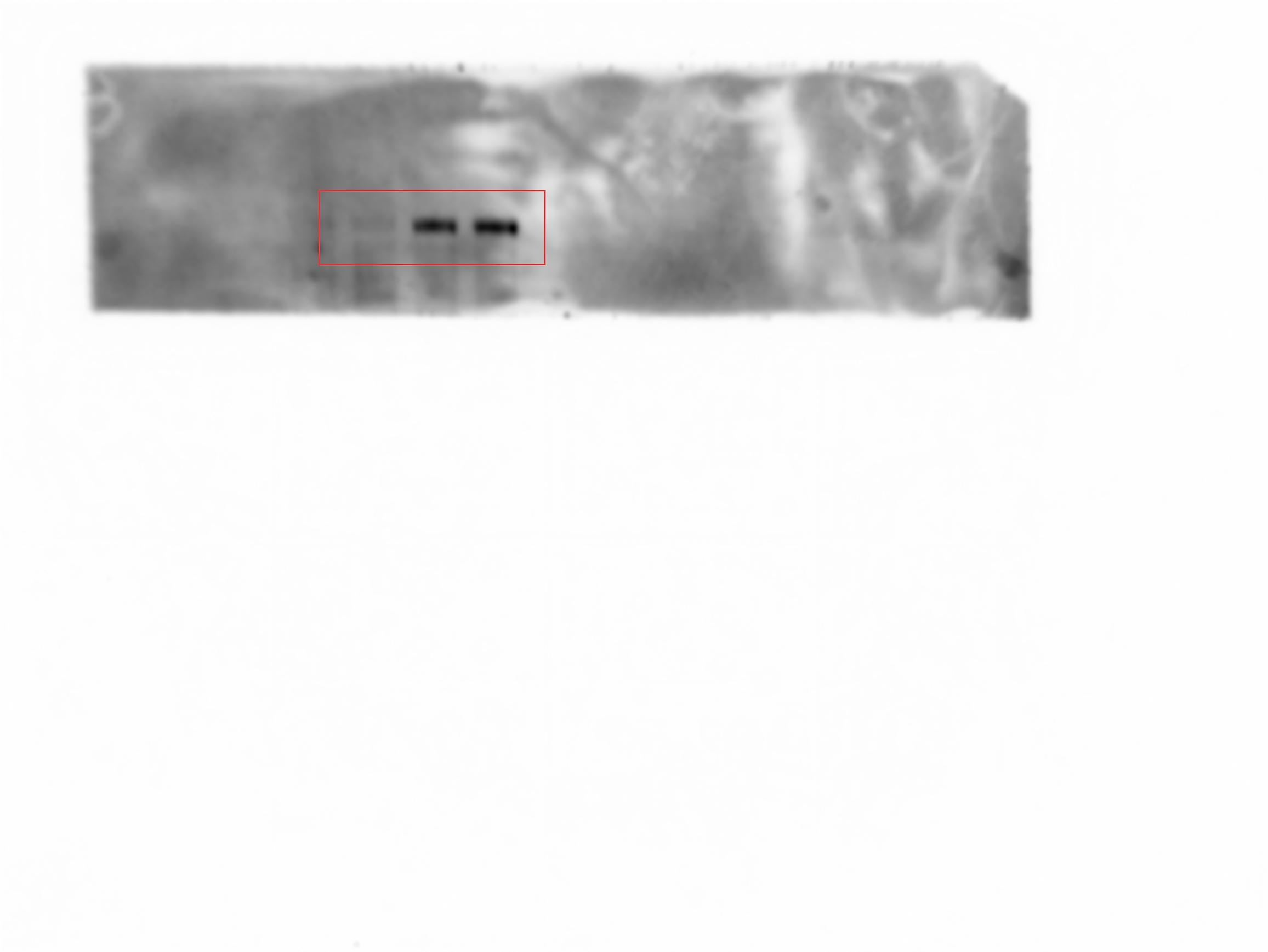

Supplement: Supplementary file 5 [file DataSheet3.zip › Fig5E left Input HA edited showing band.jpg]

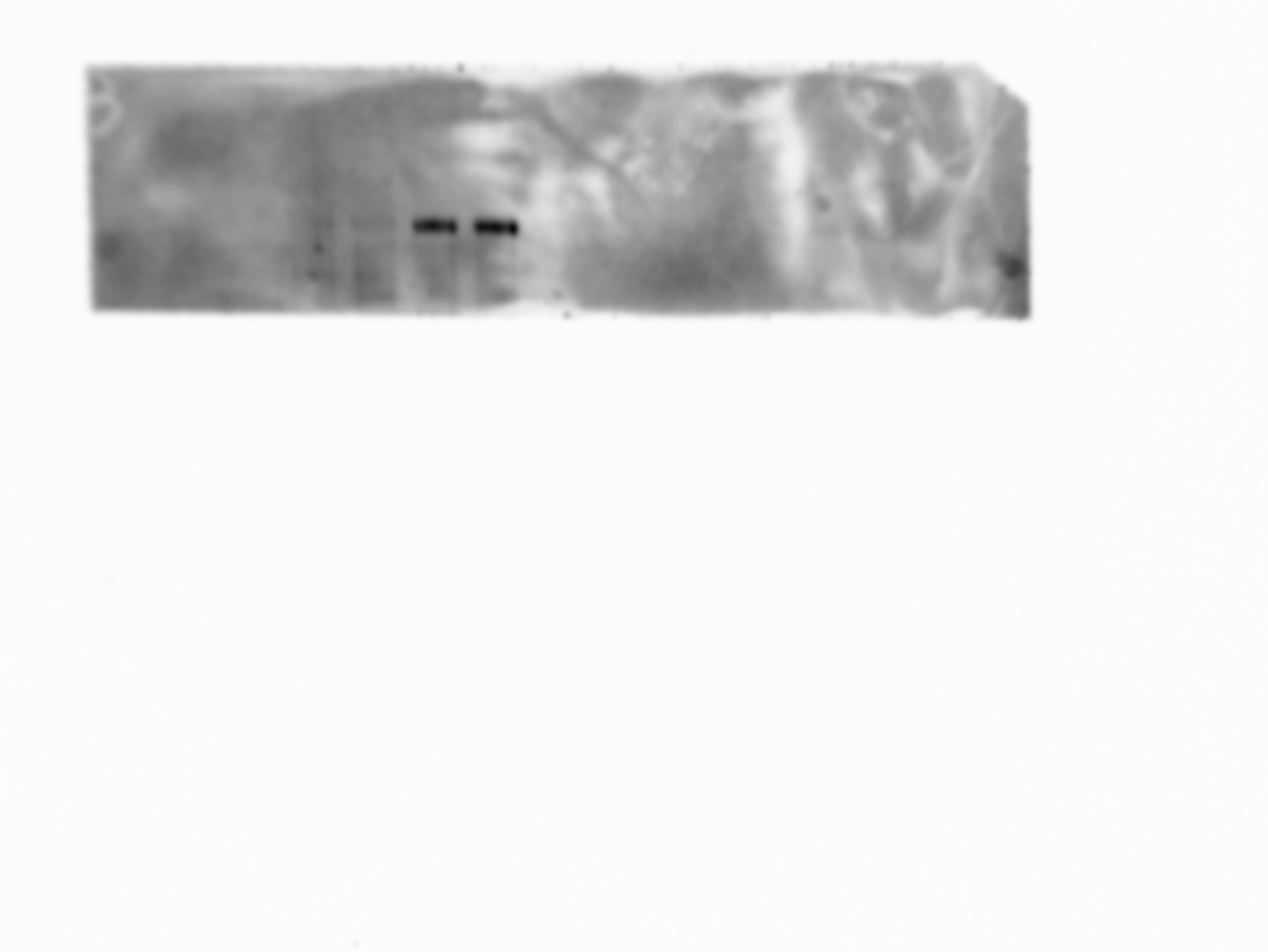

Supplement: Supplementary file 5 [file DataSheet3.zip › Fig5E left Input HA.tif]

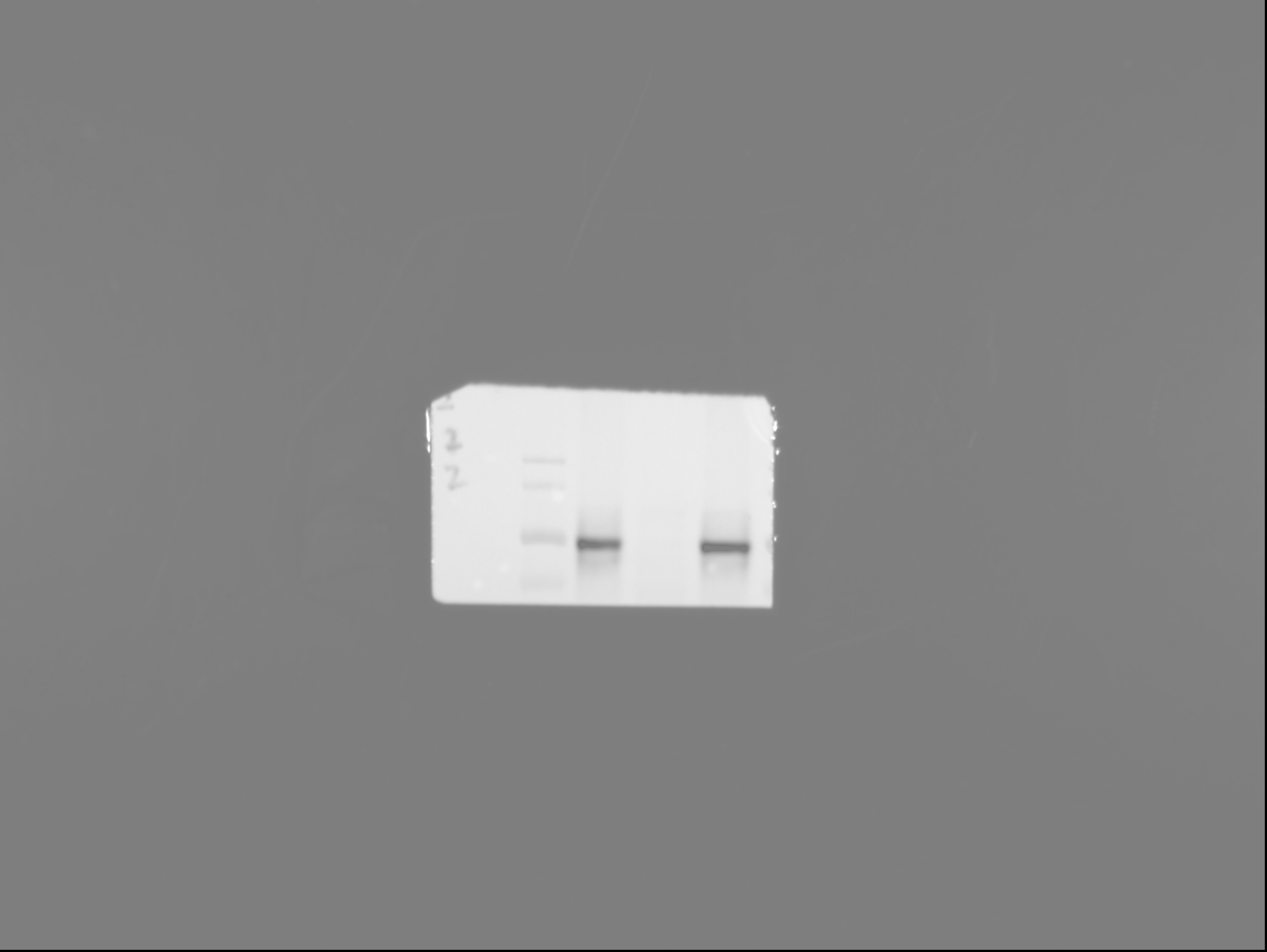

Supplement: Supplementary file 5 [file DataSheet3.zip › Fig5E left Input Myc.tif]

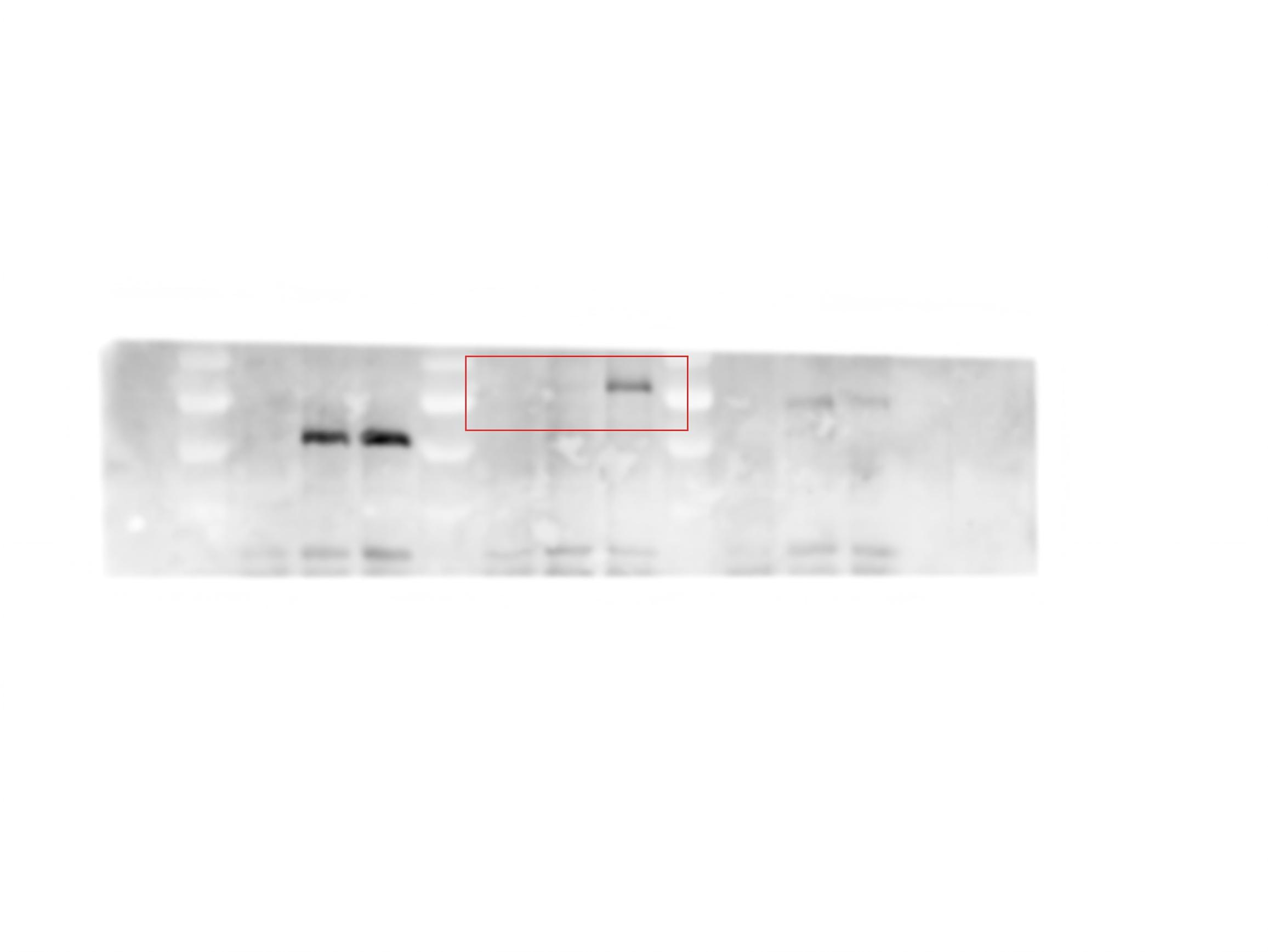

Supplement: Supplementary file 5 [file DataSheet3.zip › Fig5E left IP HA edited showing band.jpg]

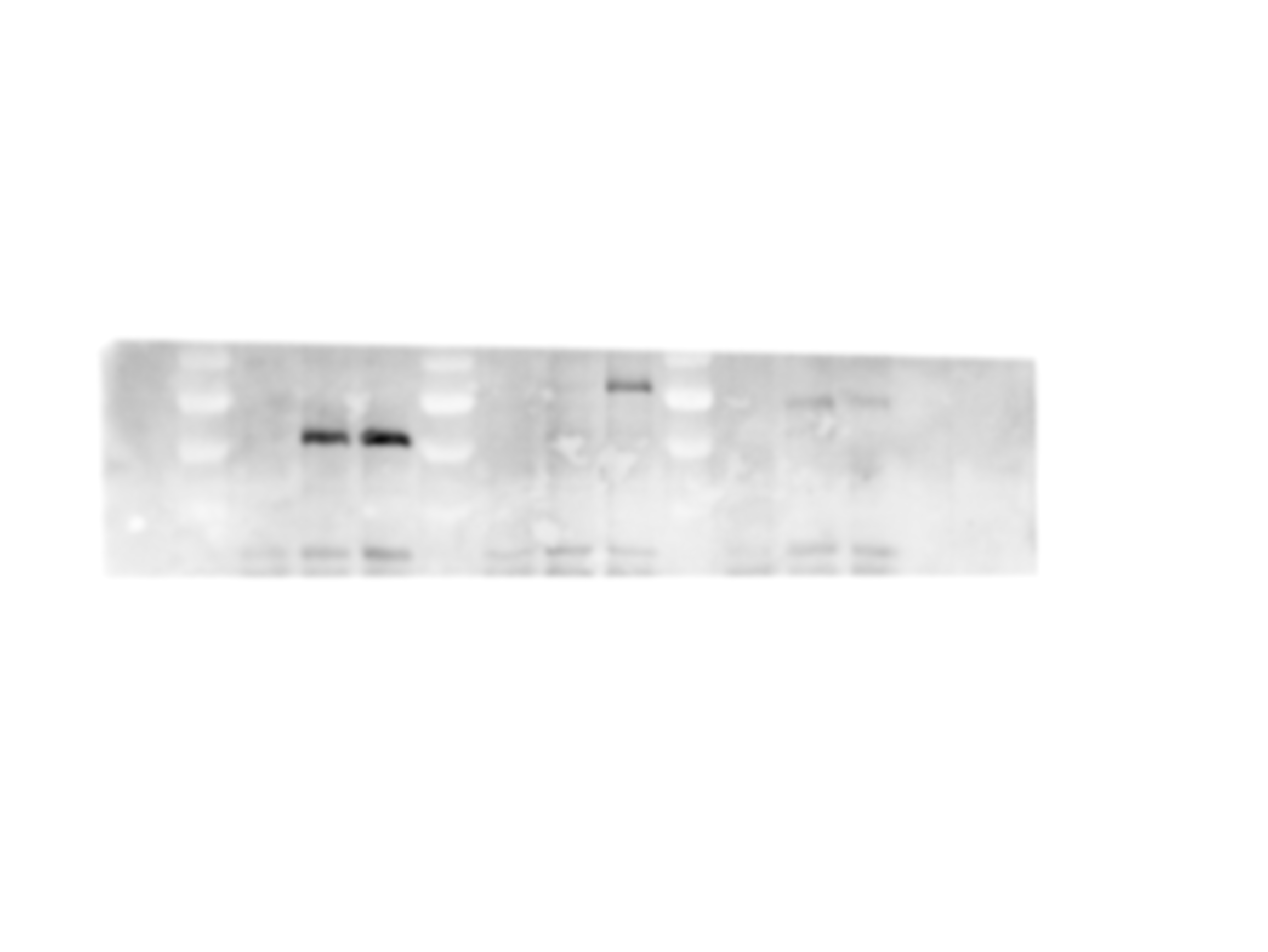

Supplement: Supplementary file 5 [file DataSheet3.zip › Fig5E left IP HA.tif]

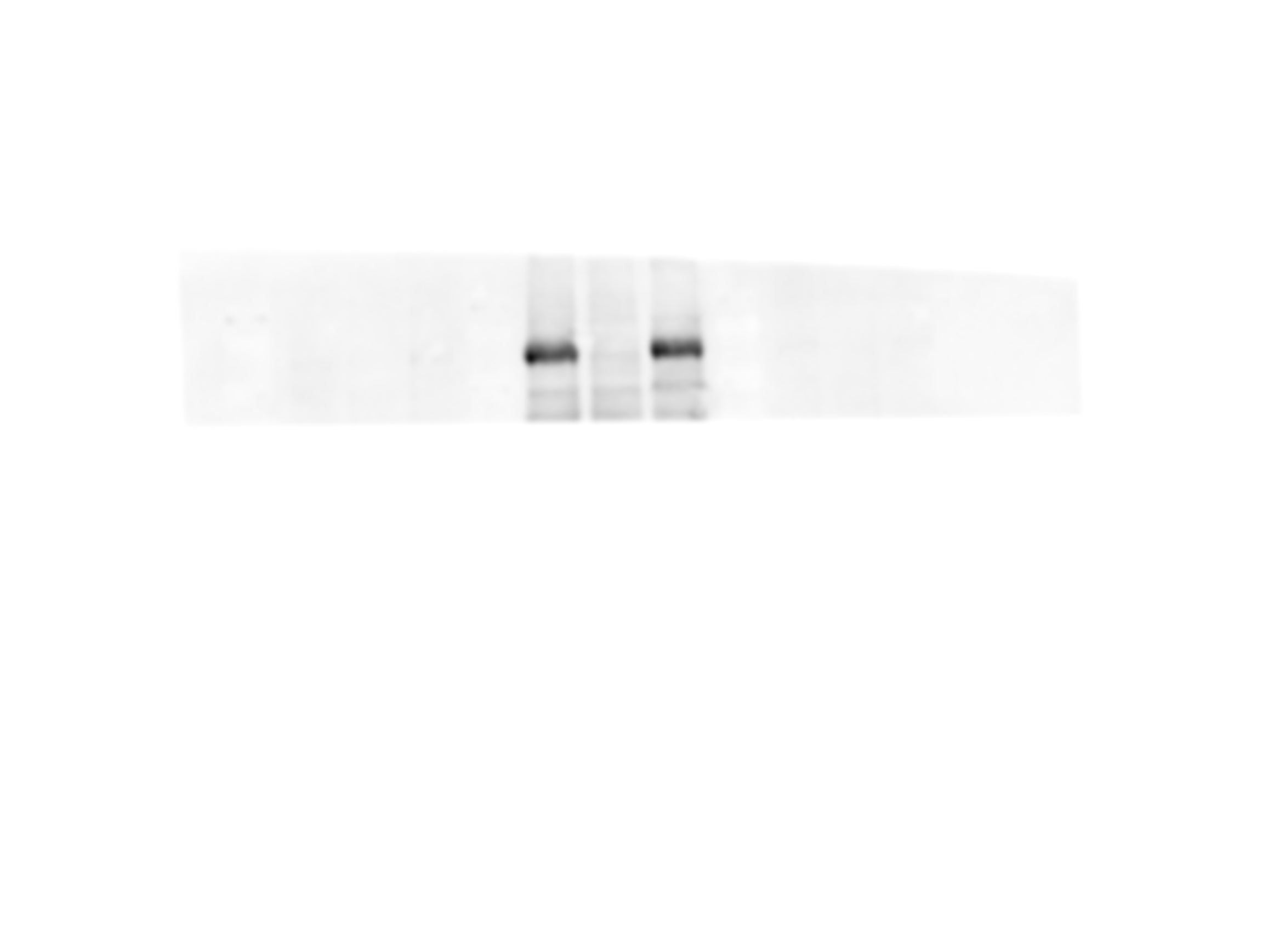

Supplement: Supplementary file 5 [file DataSheet3.zip › Fig5E left IP Myc.tif]

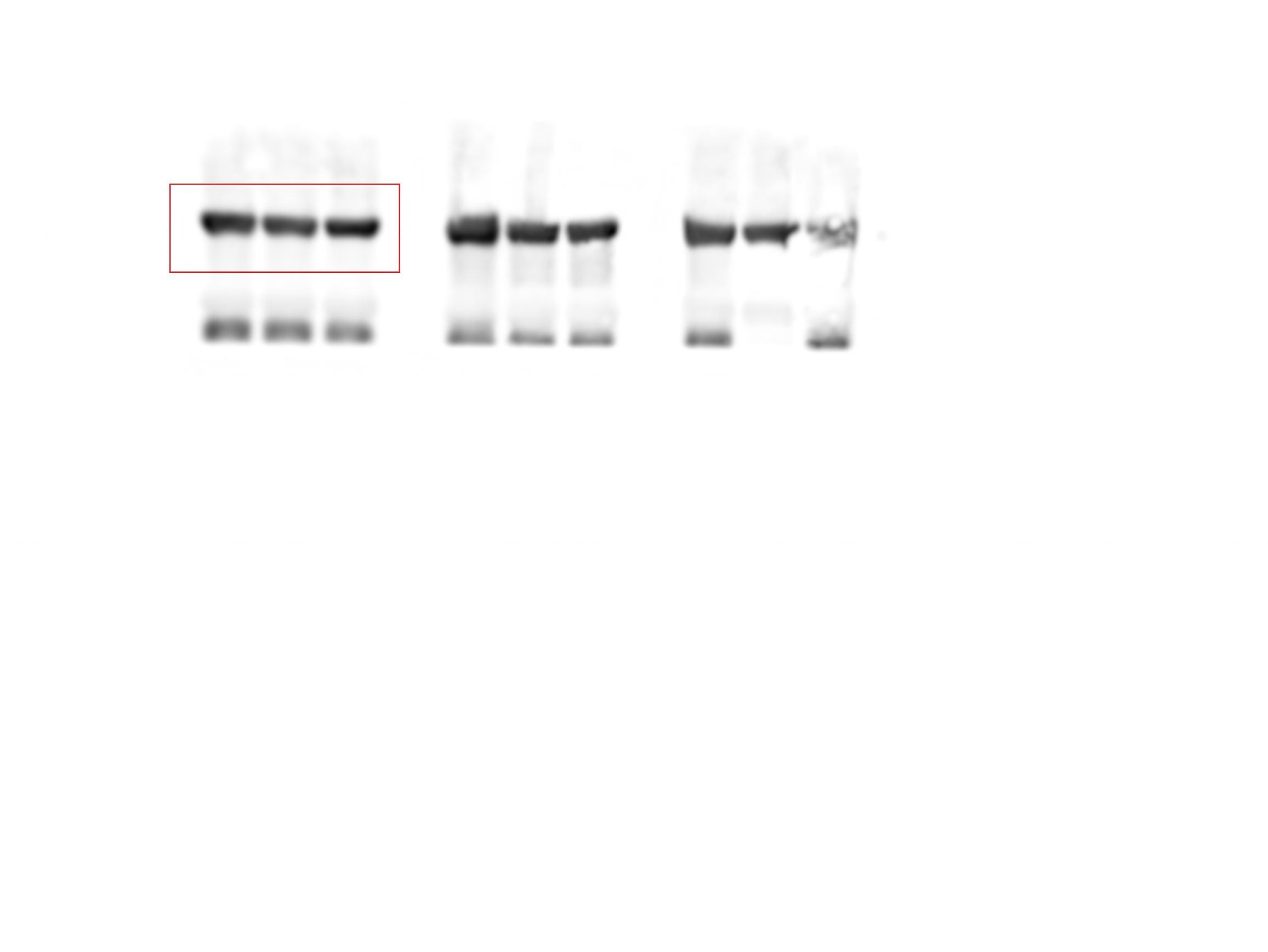

Supplement: Supplementary file 5 [file DataSheet3.zip › Fig5E right Input Actin edited showing band.jpg]

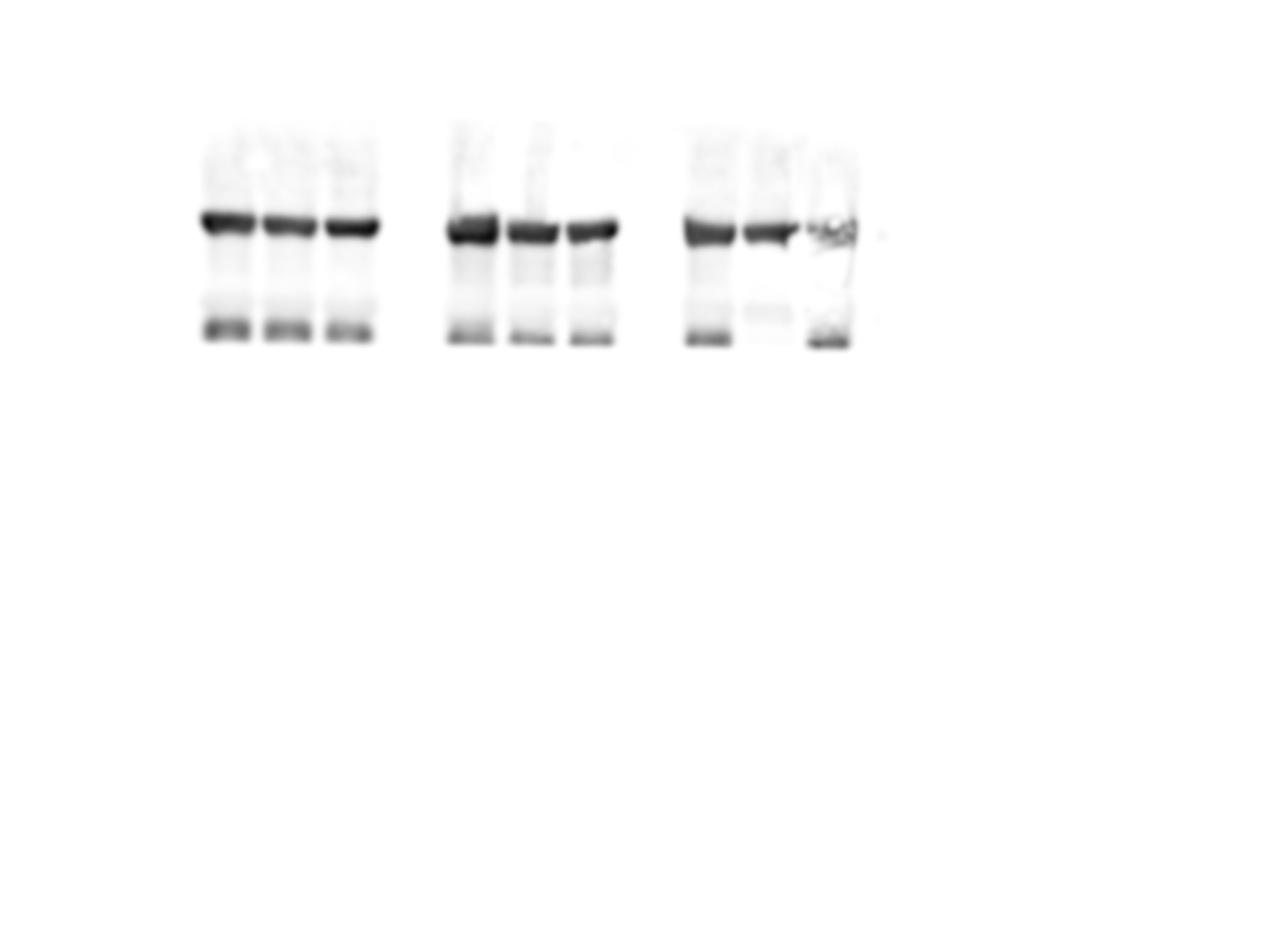

Supplement: Supplementary file 5 [file DataSheet3.zip › Fig5E right Input Actin.tif]

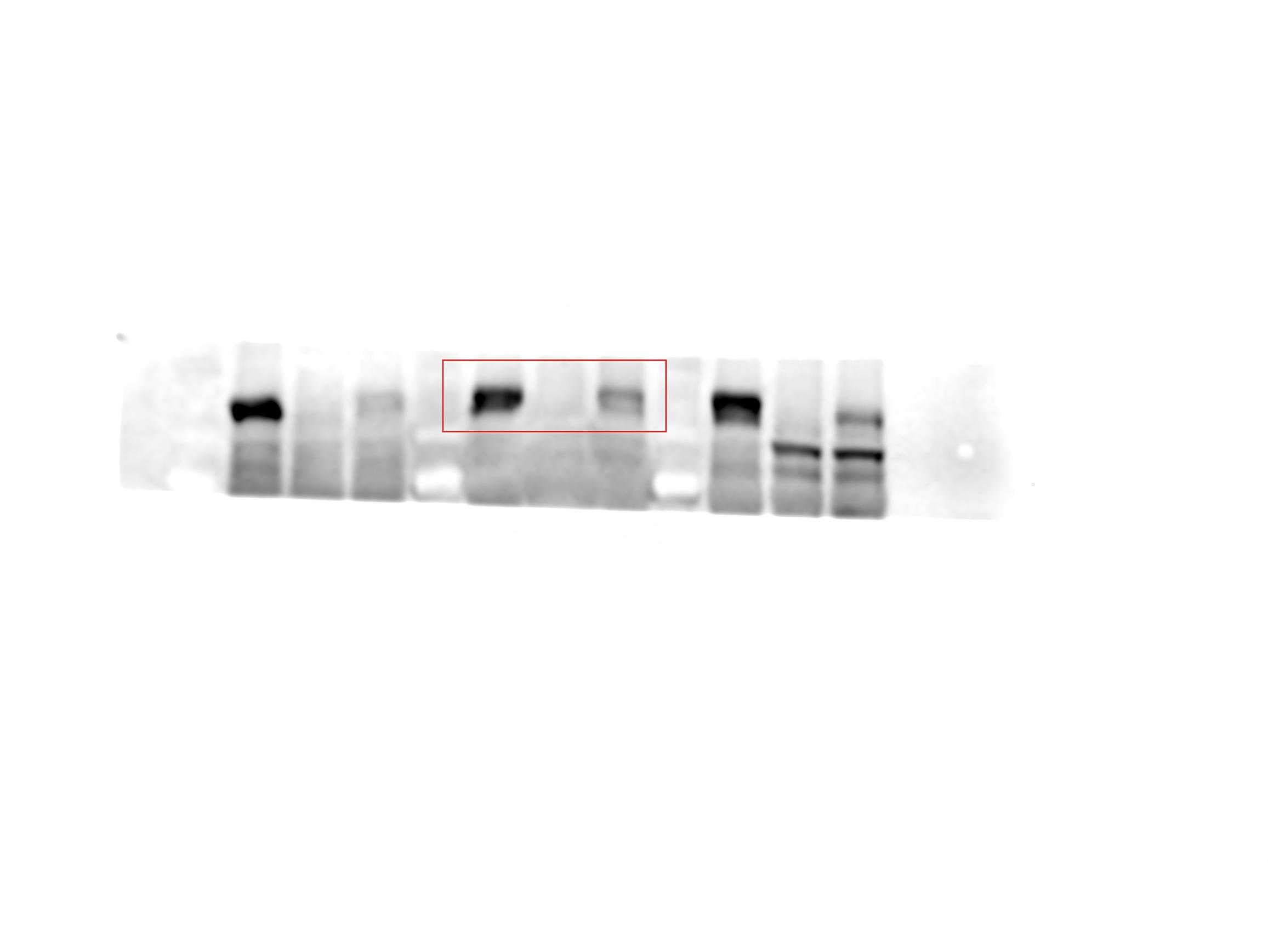

Supplement: Supplementary file 5 [file DataSheet3.zip › Fig5E right Input HA edited showing band.jpg]

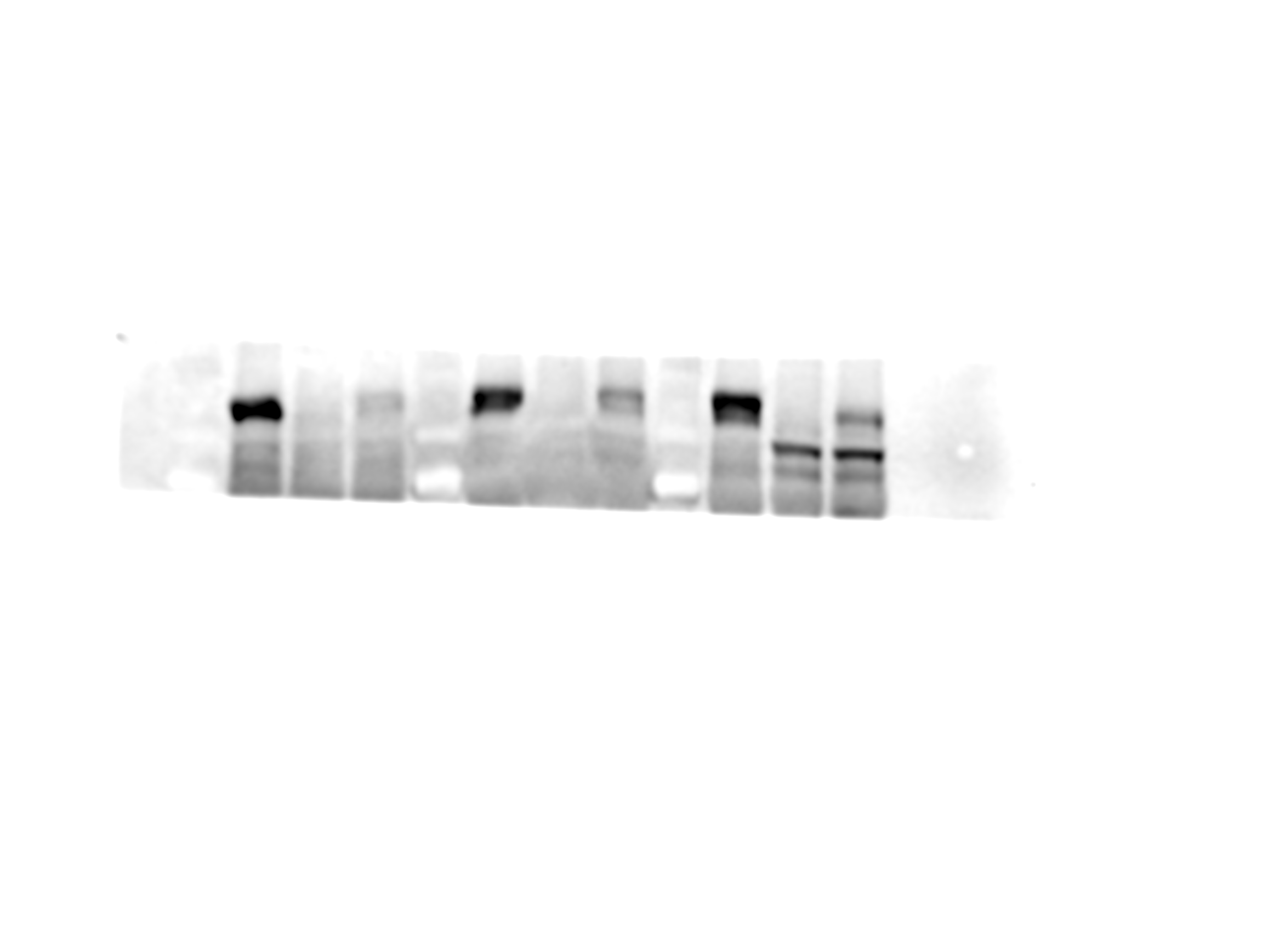

Supplement: Supplementary file 5 [file DataSheet3.zip › Fig5E right Input HA.tif]

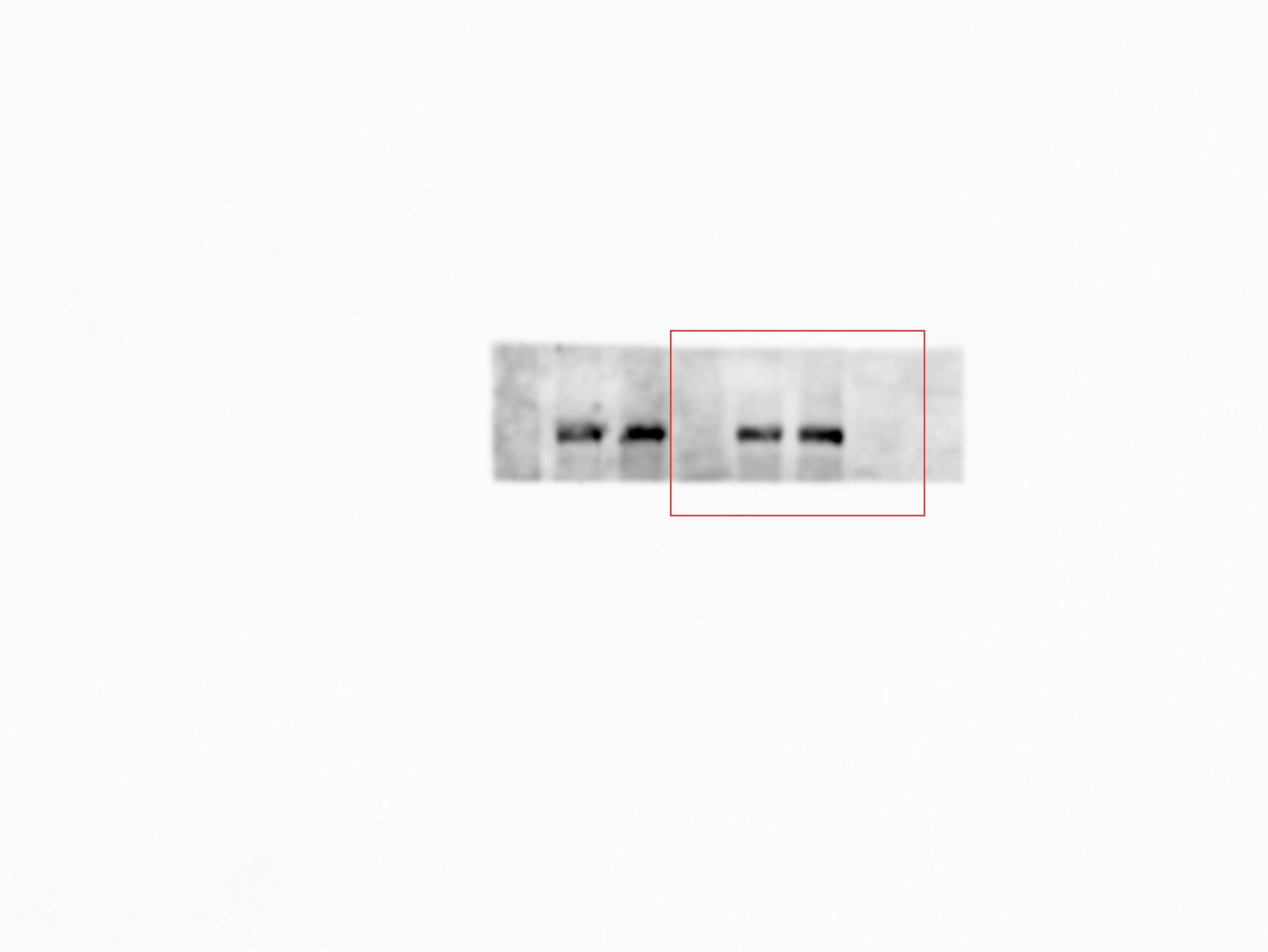

Supplement: Supplementary file 5 [file DataSheet3.zip › Fig5E right Input Myc edited showing band.jpg]

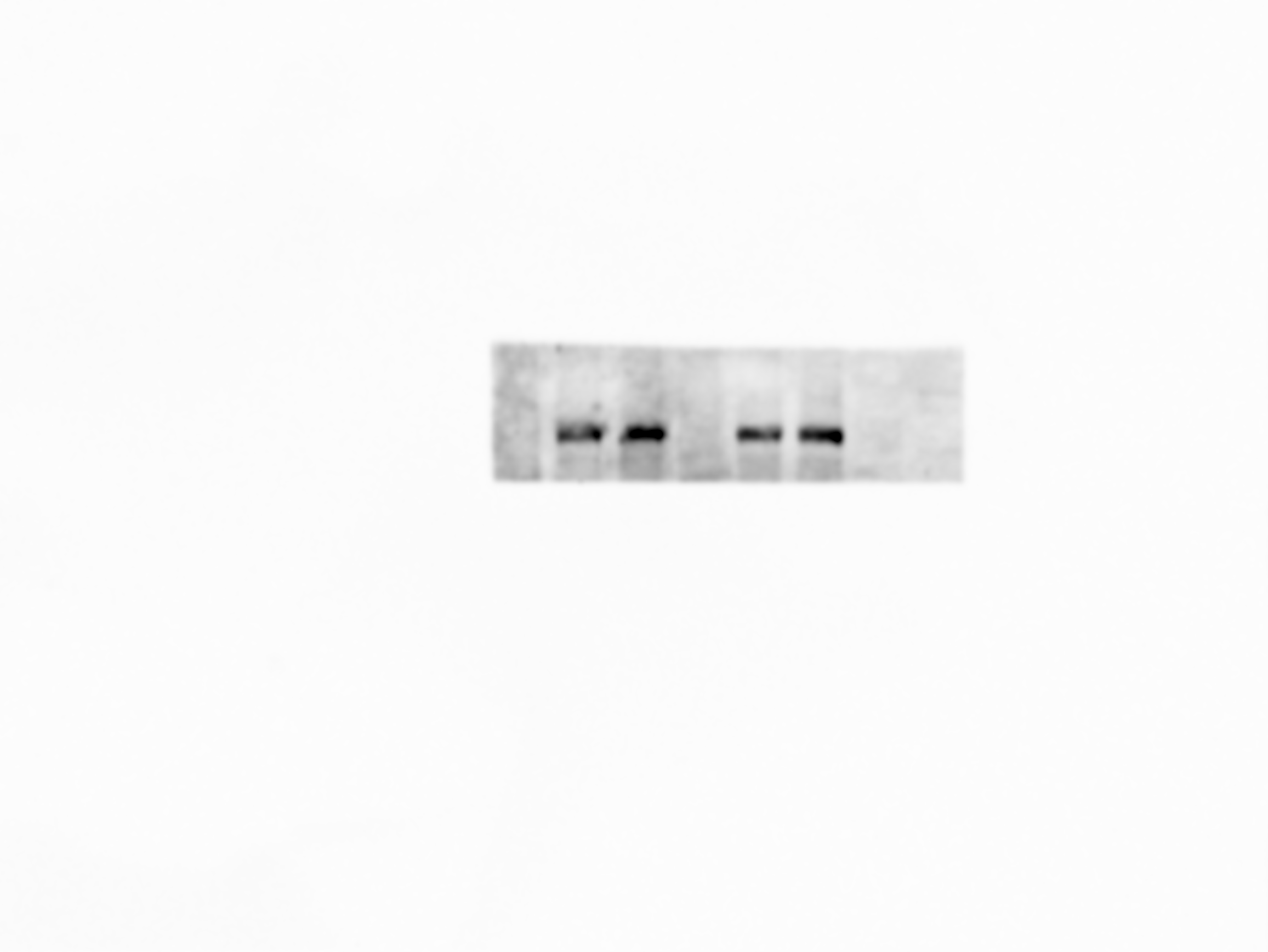

Supplement: Supplementary file 5 [file DataSheet3.zip › Fig5E right input Myc.tif]

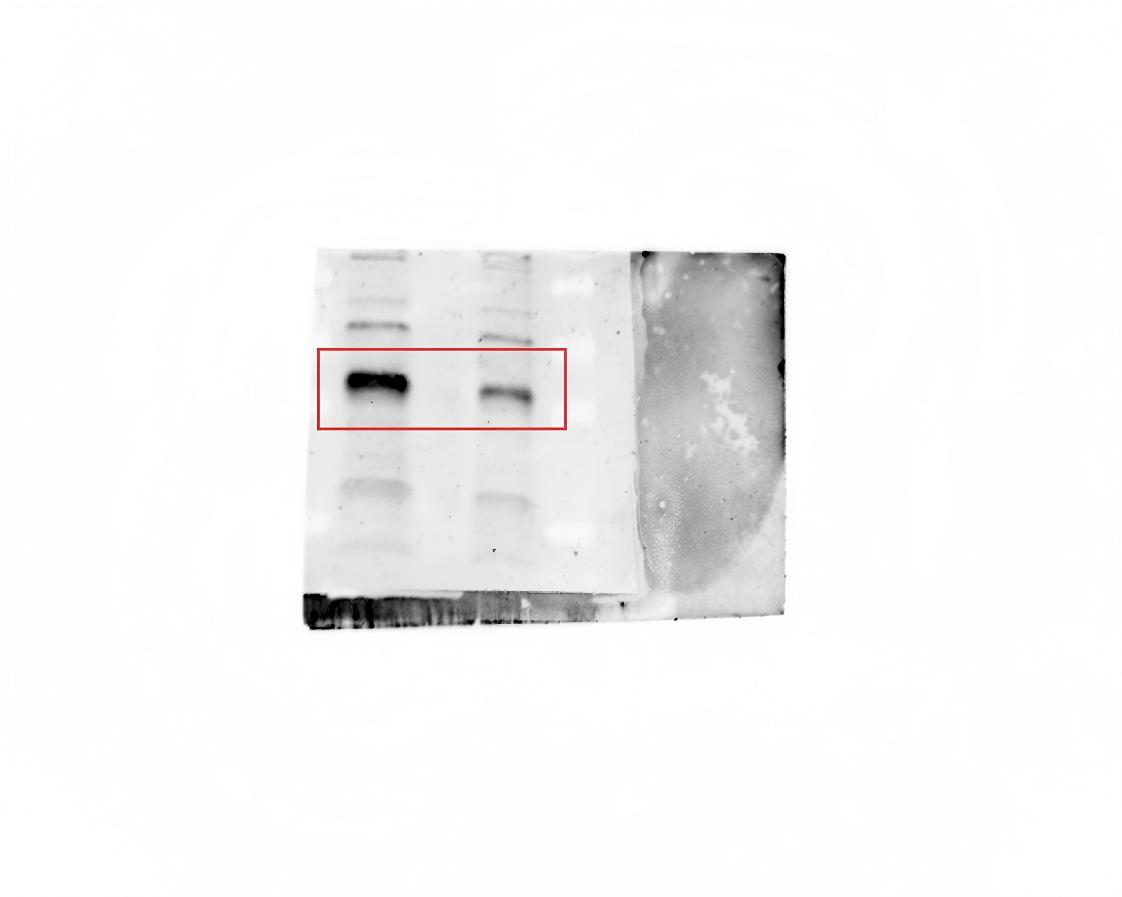

Supplement: Supplementary file 5 [file DataSheet3.zip › Fig5E right IP HA edited showing band.jpg]

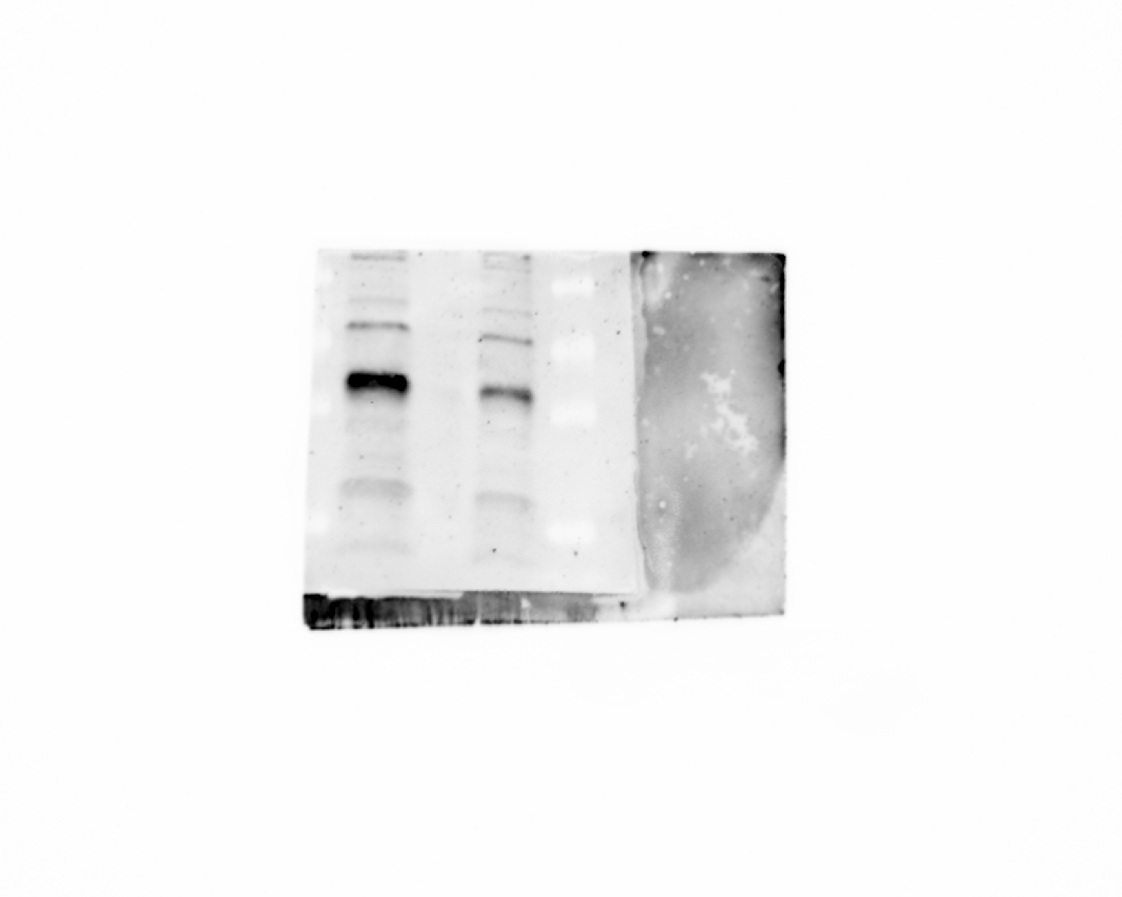

Supplement: Supplementary file 5 [file DataSheet3.zip › Fig5E right IP HA.jpg]

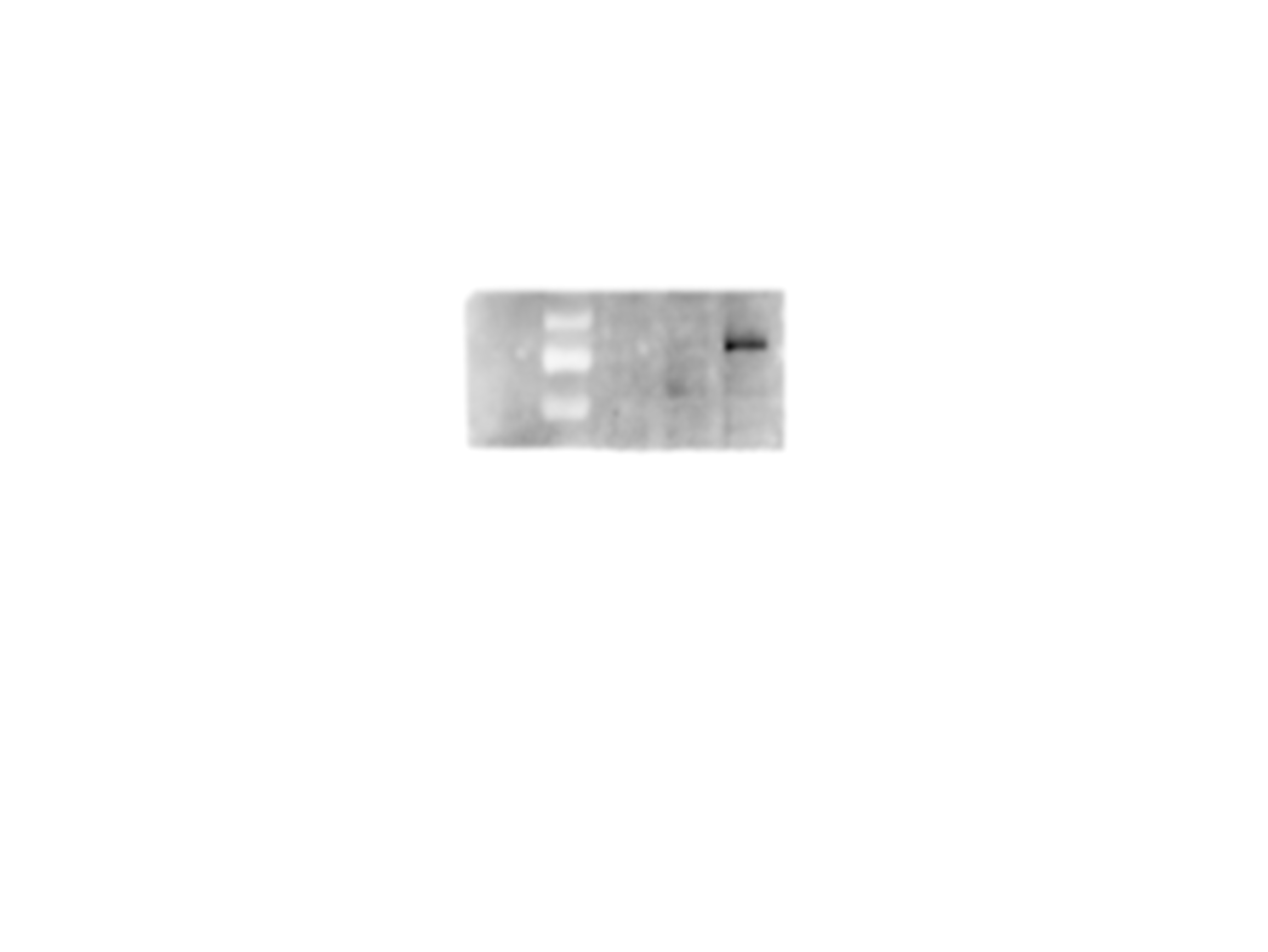

Supplement: Supplementary file 5 [file DataSheet3.zip › Fig5E right IP Myc edited showing band.tif]

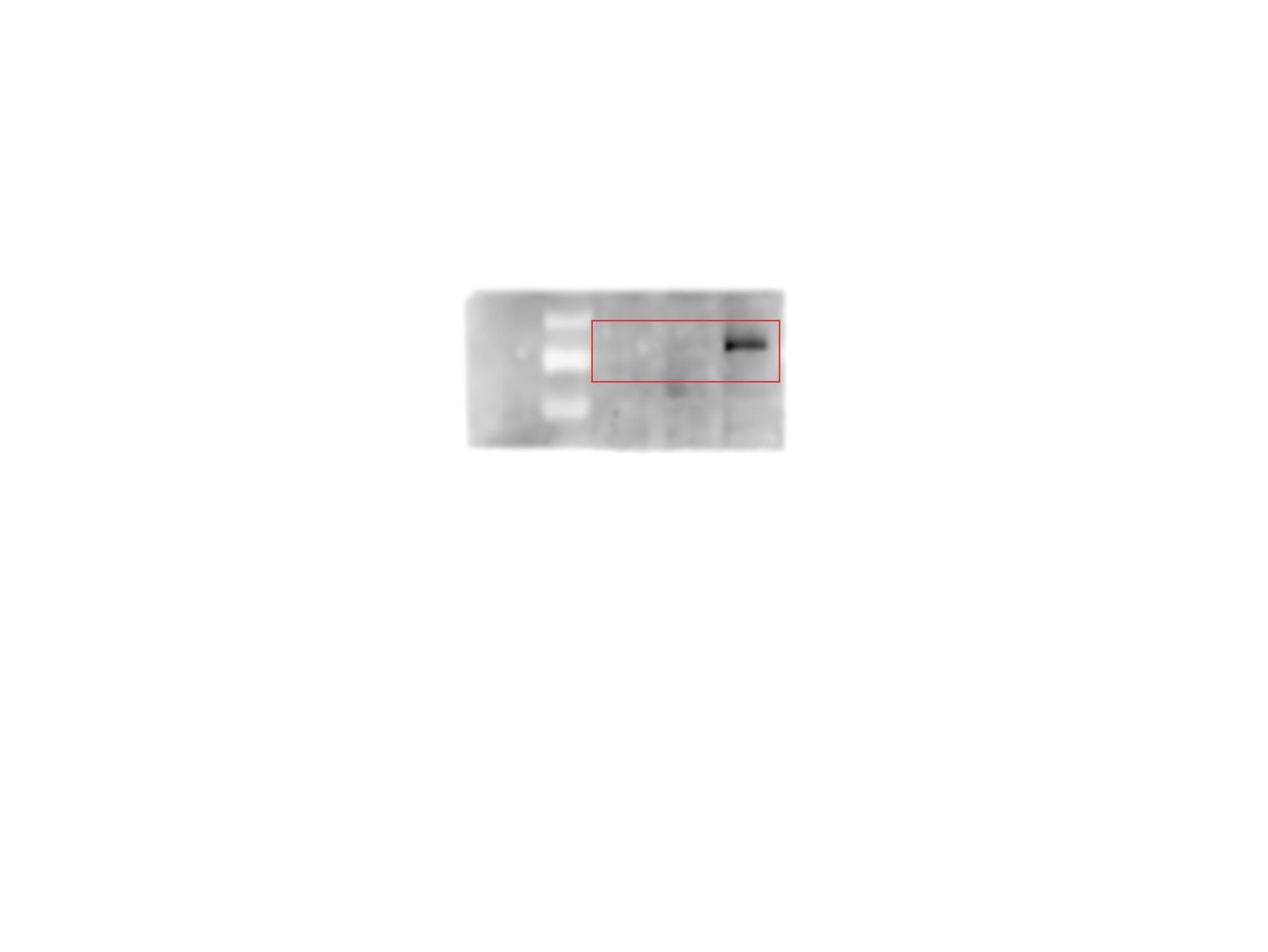

Supplement: Supplementary file 5 [file DataSheet3.zip › Fig5E right IP Myc.jpg]

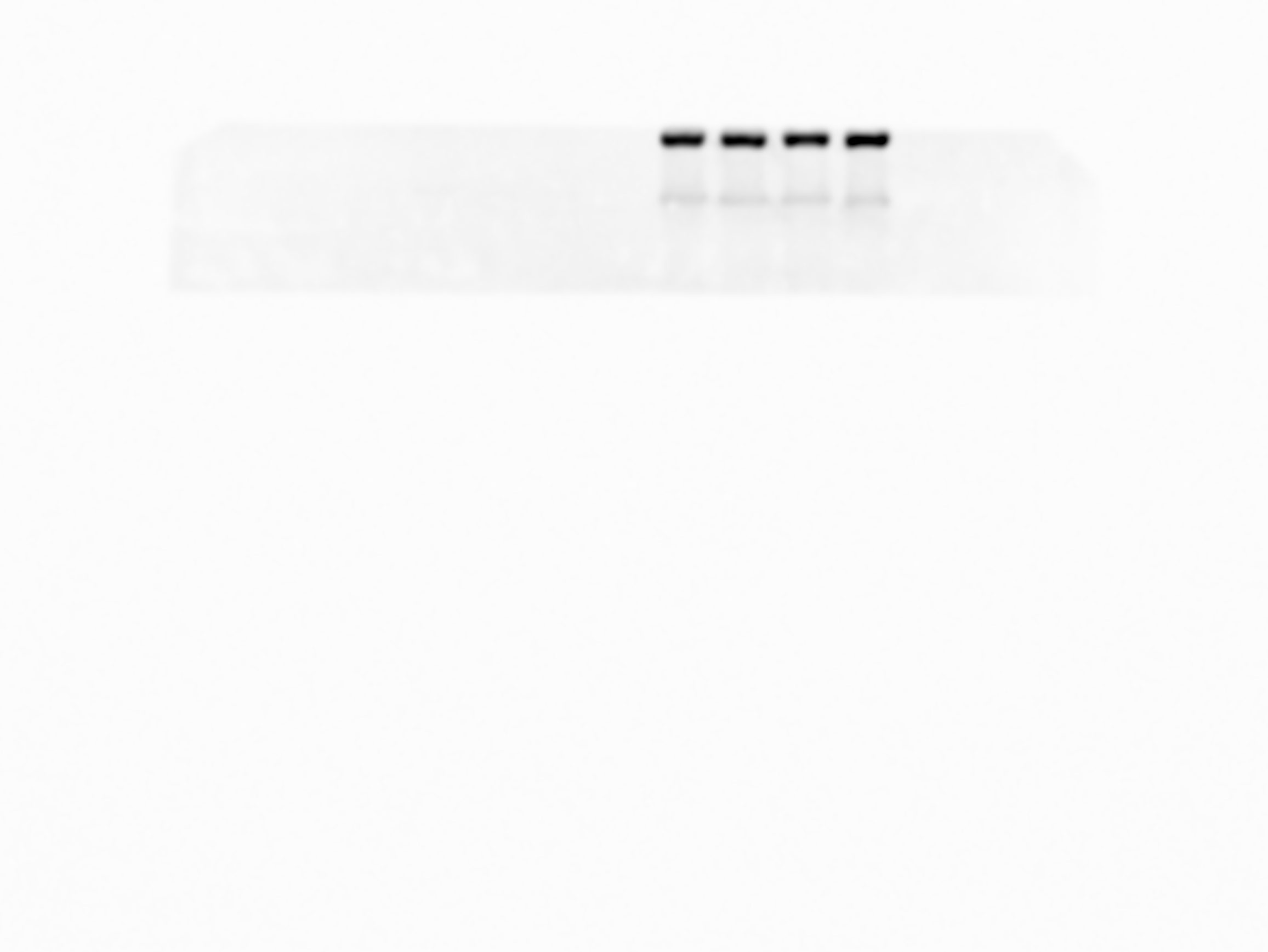

Supplement: Supplementary file 5 [file DataSheet3.zip › Fig5F Input Actin.tif]

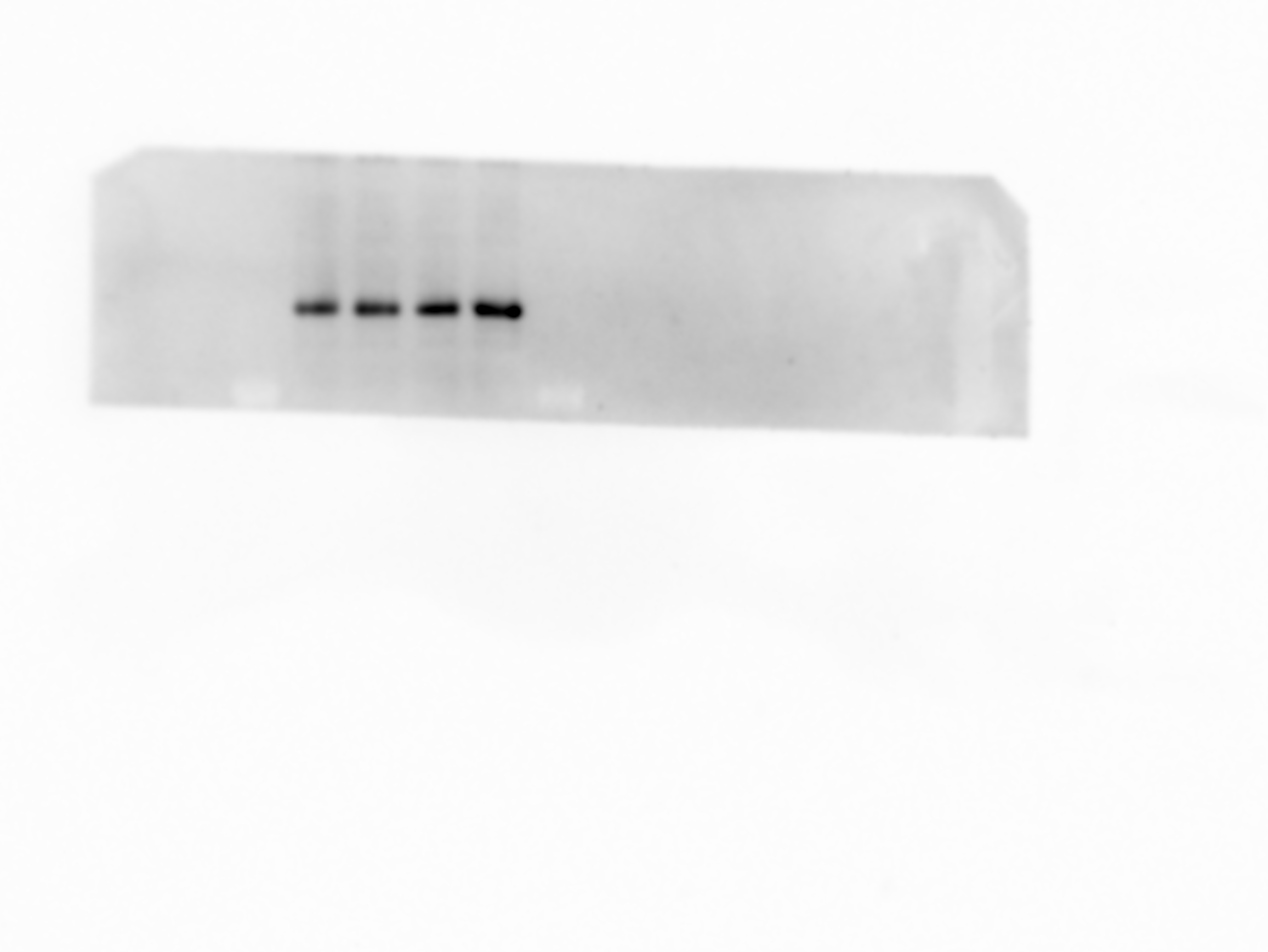

Supplement: Supplementary file 5 [file DataSheet3.zip › Fig5F Input MAVS.tif]

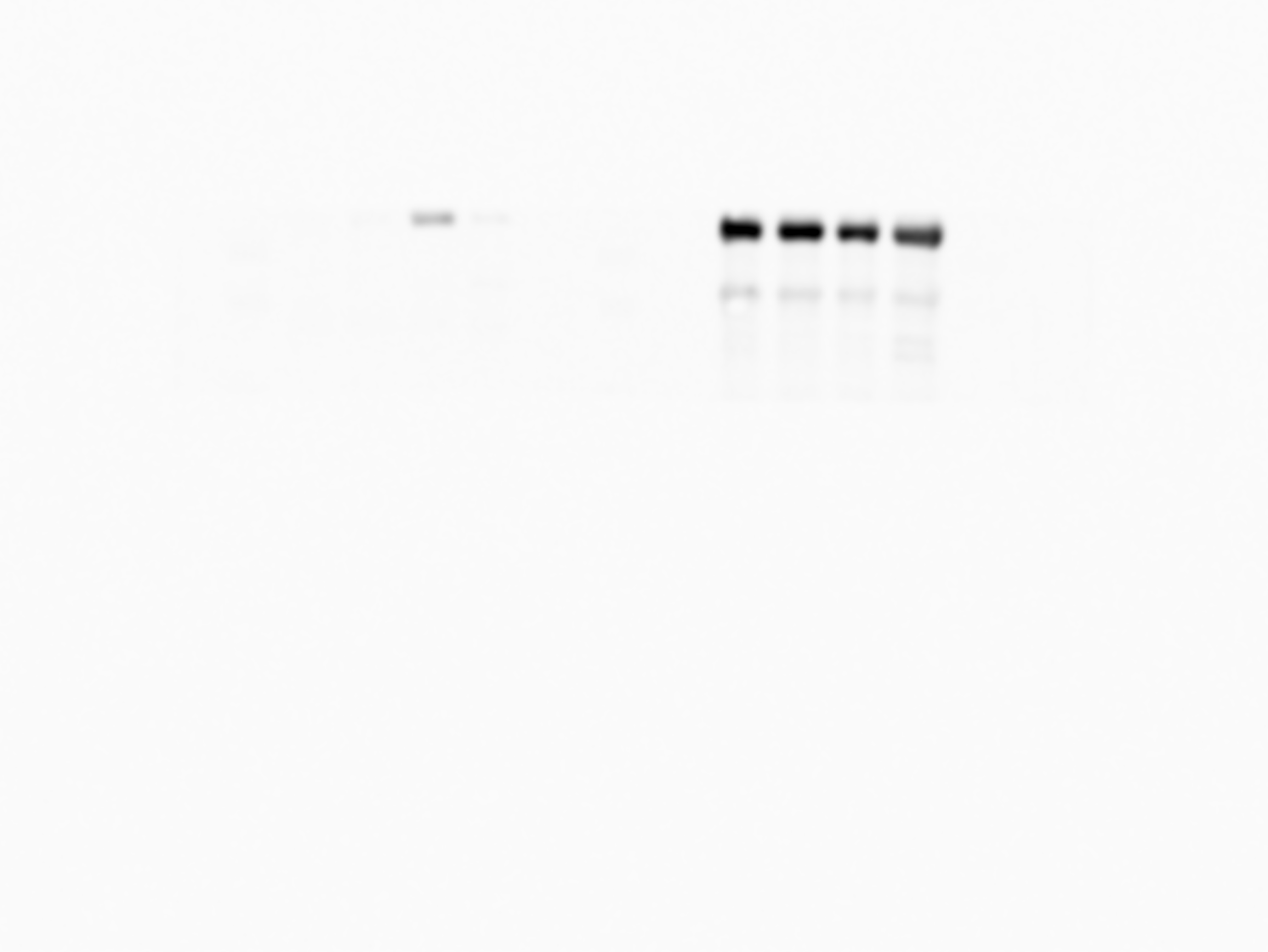

Supplement: Supplementary file 5 [file DataSheet3.zip › Fig5F Input TRIM28.tif]

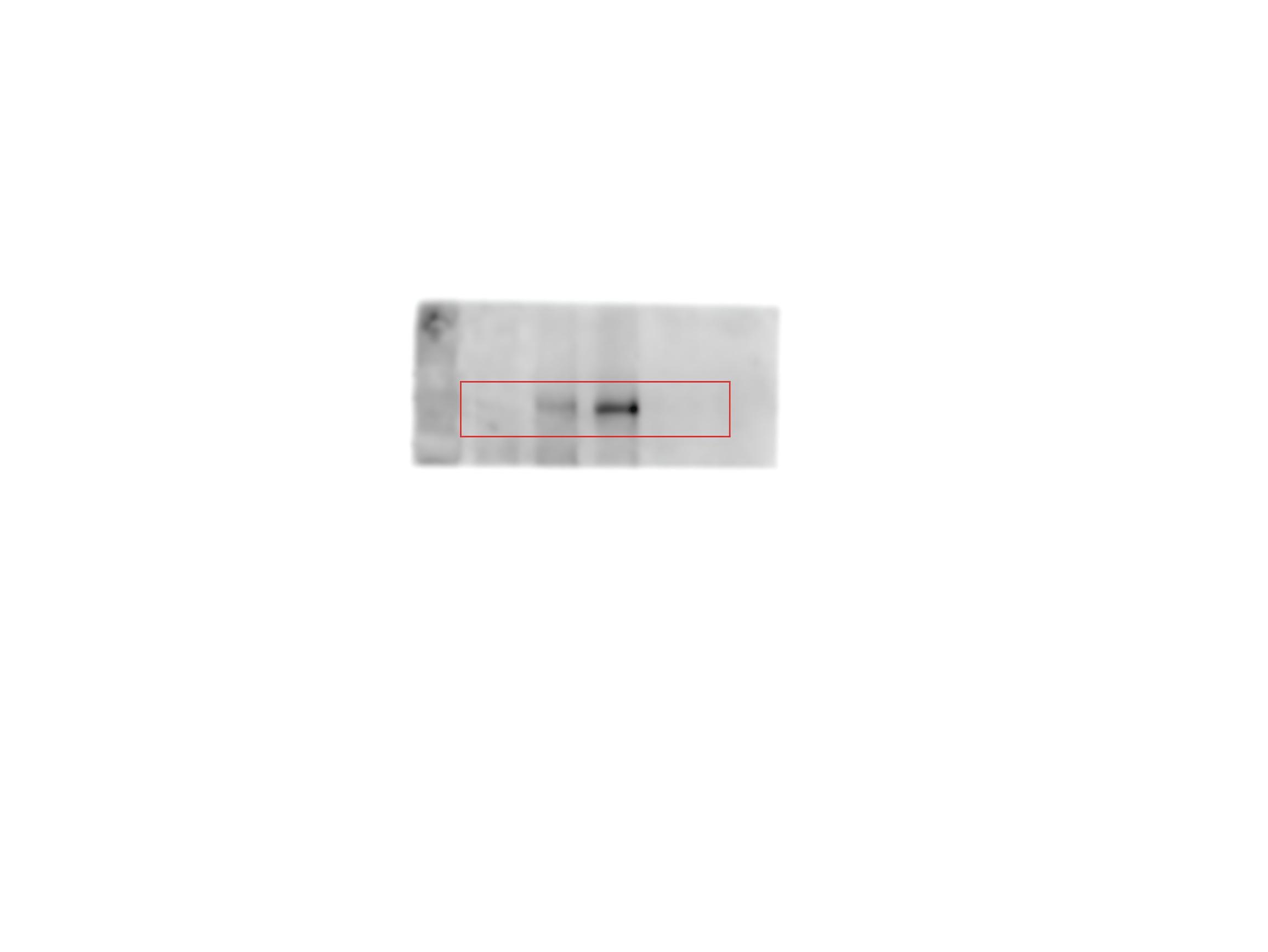

Supplement: Supplementary file 5 [file DataSheet3.zip › Fig5F IP MAVS edited showing band.jpg]

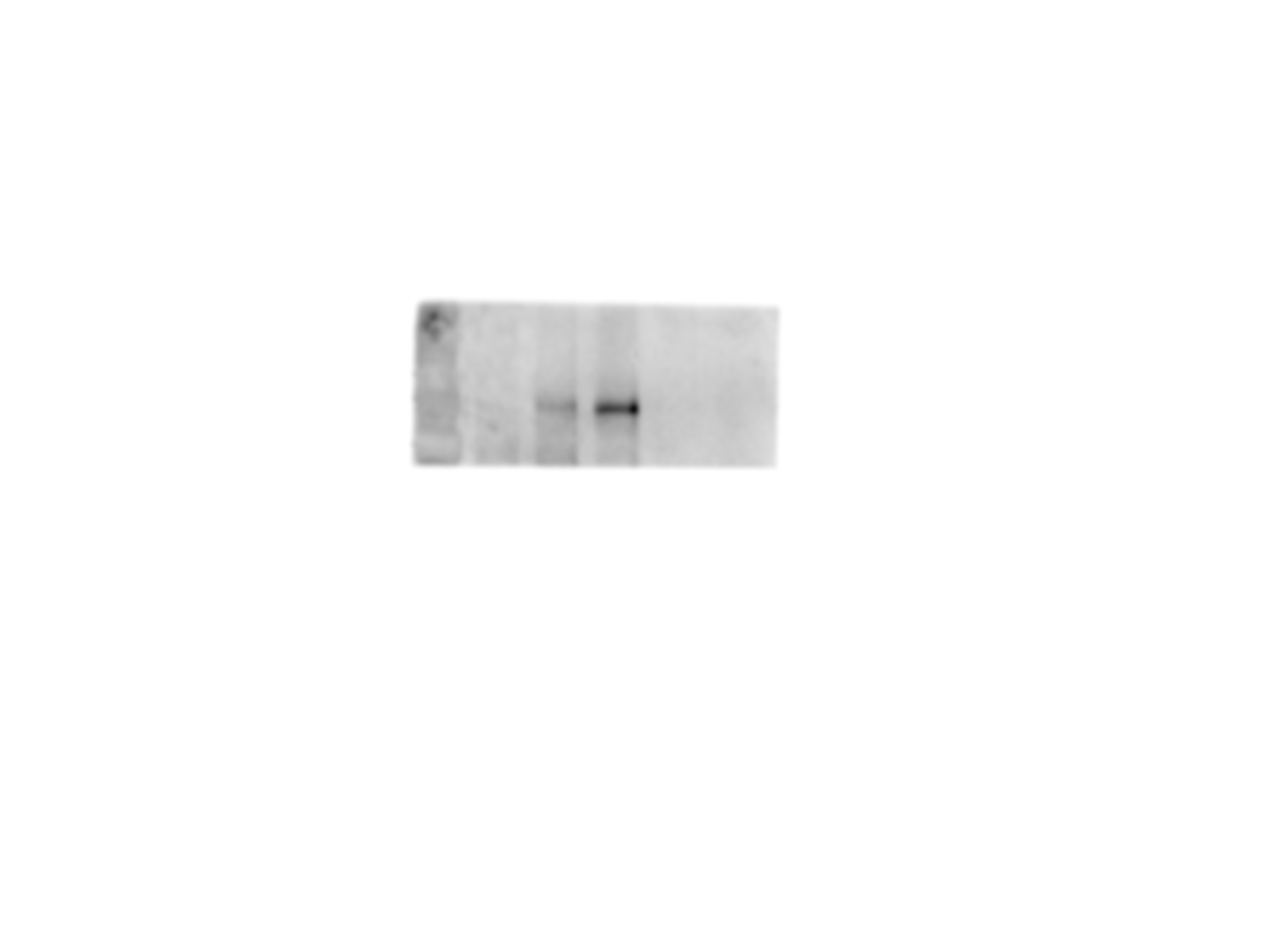

Supplement: Supplementary file 5 [file DataSheet3.zip › Fig5F IP MAVS.tif]

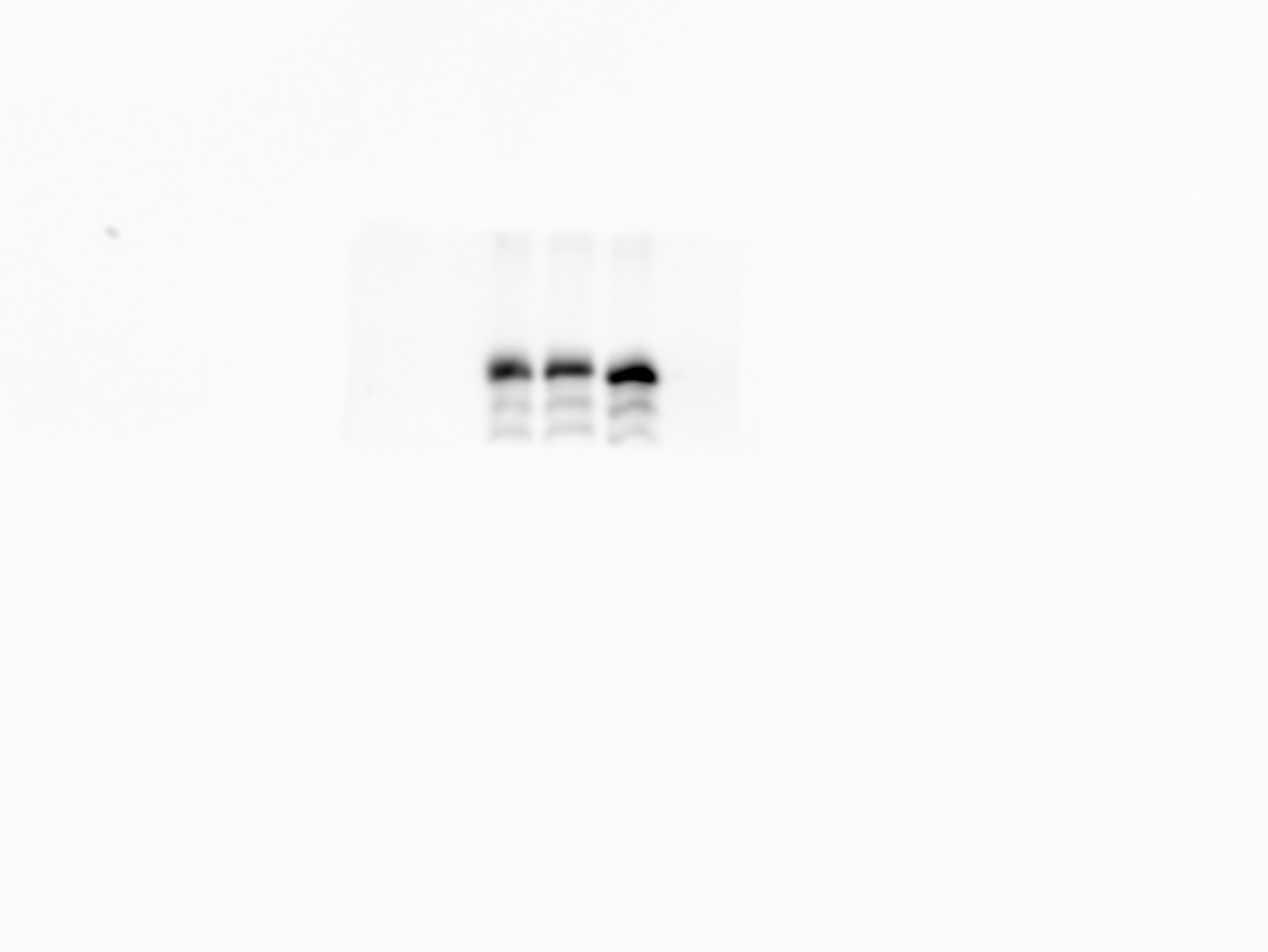

Supplement: Supplementary file 5 [file DataSheet3.zip › Fig5F IP TRIM28.tif]

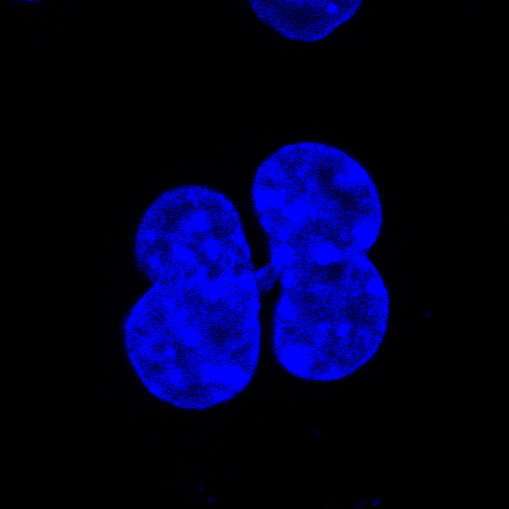

Supplement: Supplementary file 6 [file DataSheet4.zip › Fig6 A DAPI.tif]

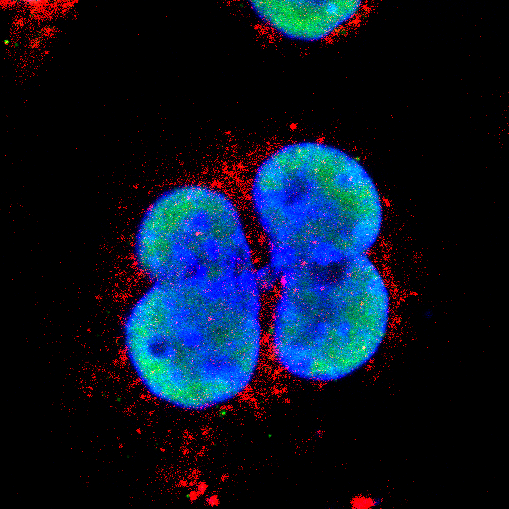

Supplement: Supplementary file 6 [file DataSheet4.zip › Fig6 A Merge1.tif]

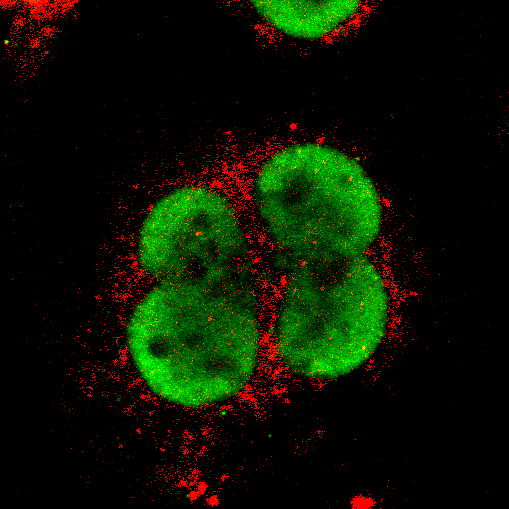

Supplement: Supplementary file 6 [file DataSheet4.zip › Fig6 A Merge2.tif]

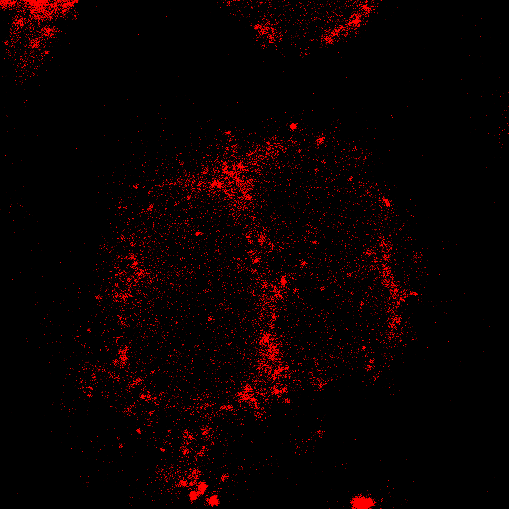

Supplement: Supplementary file 6 [file DataSheet4.zip › Fig6 A Mitotracker.tif]

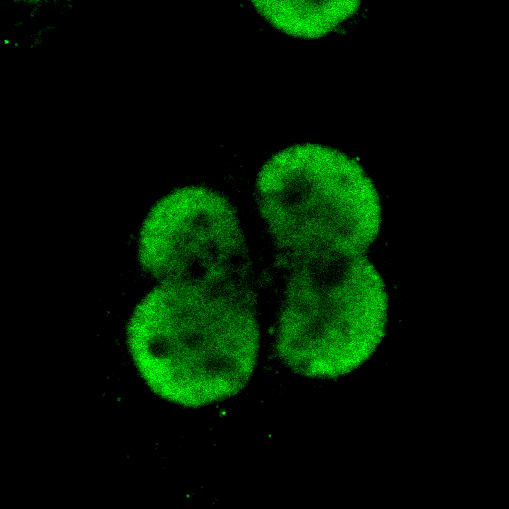

Supplement: Supplementary file 6 [file DataSheet4.zip › Fig6 A TRIM28.tif]

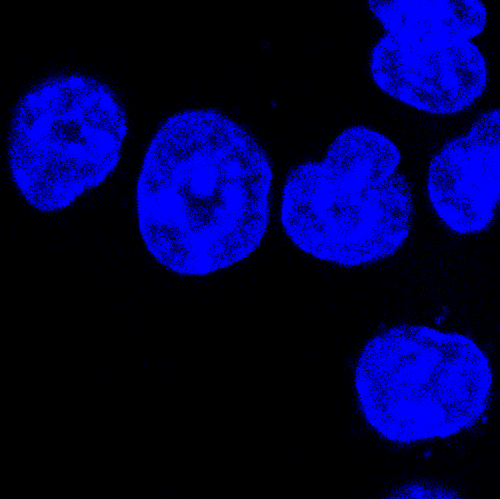

Supplement: Supplementary file 6 [file DataSheet4.zip › Fig6 E UI DAPI.tif]

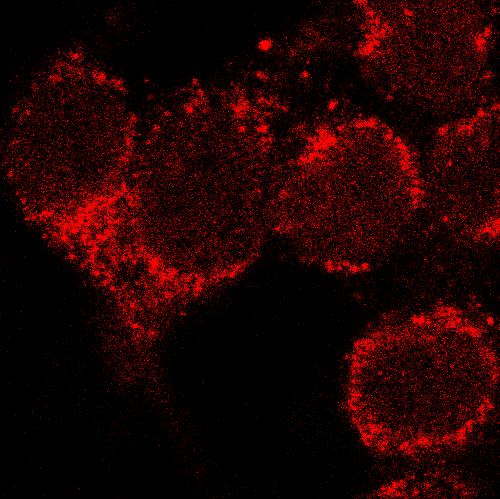

Supplement: Supplementary file 6 [file DataSheet4.zip › Fig6 E UI MAVS.tif]

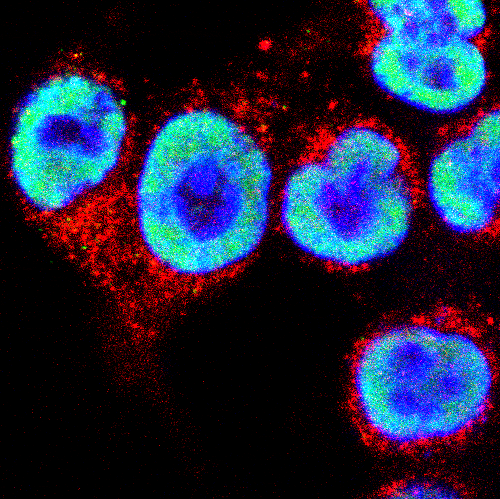

Supplement: Supplementary file 6 [file DataSheet4.zip › Fig6 E UI Merge.tif]

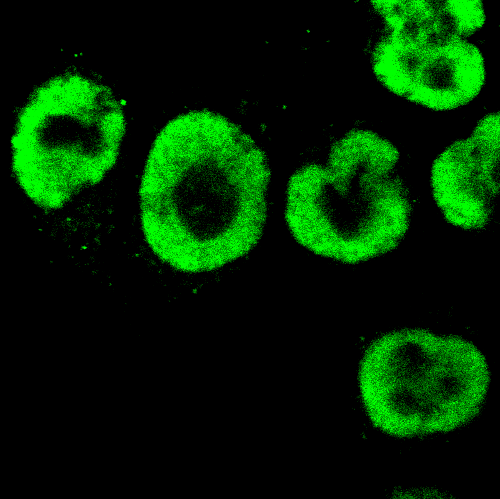

Supplement: Supplementary file 6 [file DataSheet4.zip › Fig6 E UI TRIM28.tif]

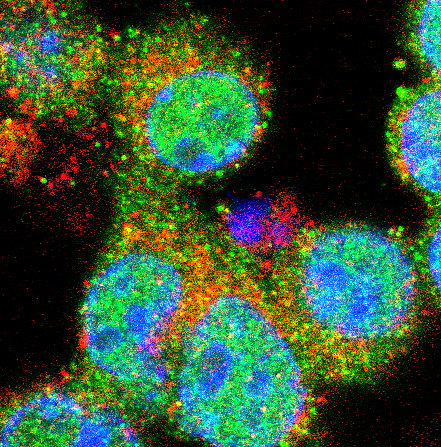

Supplement: Supplementary file 6 [file DataSheet4.zip › Fig6 E VAV Merge.tif]

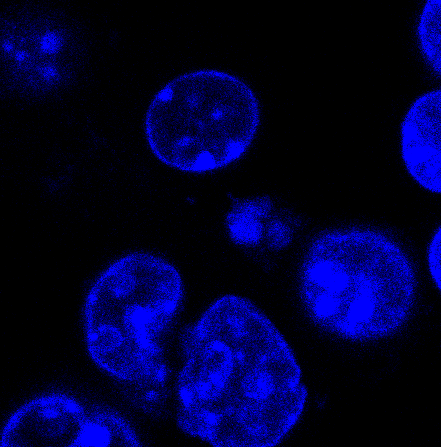

Supplement: Supplementary file 6 [file DataSheet4.zip › Fig6 E VSV DAPI.tif]

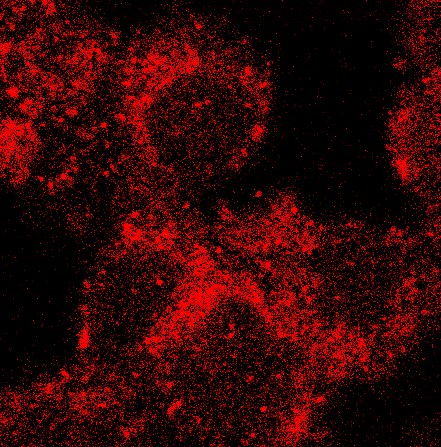

Supplement: Supplementary file 6 [file DataSheet4.zip › Fig6 E VSV MAVS.tif]

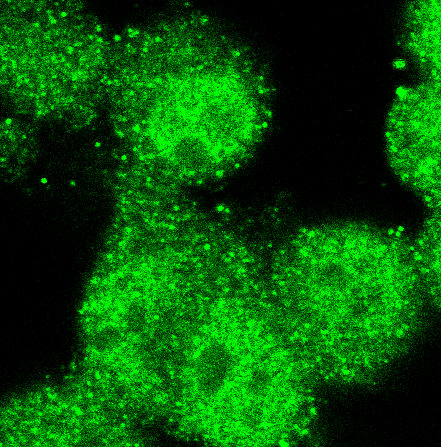

Supplement: Supplementary file 6 [file DataSheet4.zip › Fig6 E VSV TRIM28.tif]

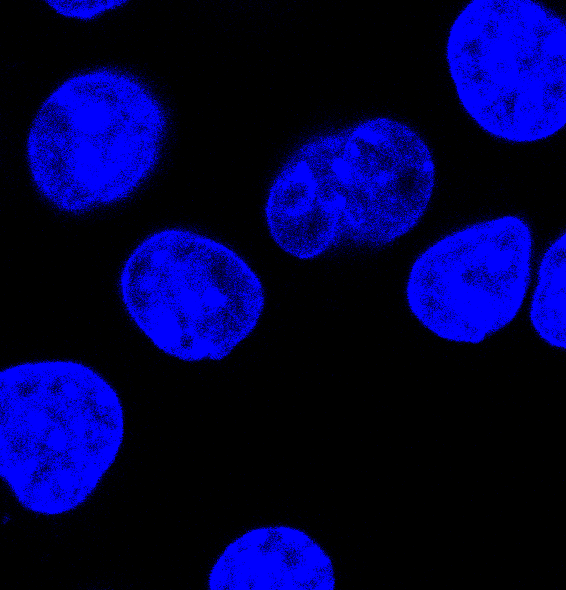

Supplement: Supplementary file 6 [file DataSheet4.zip › Fig6B UI DAPI.tif]

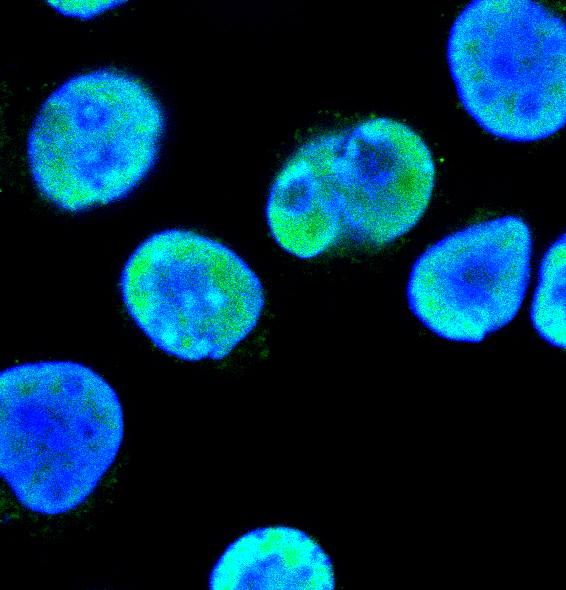

Supplement: Supplementary file 6 [file DataSheet4.zip › Fig6B UI Merge.tif]

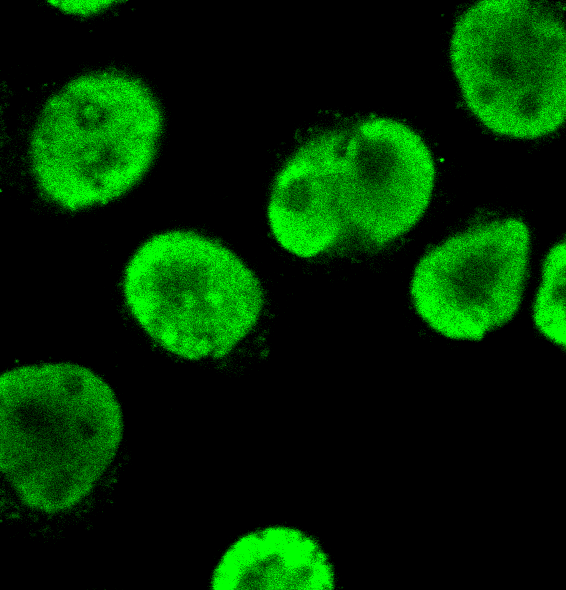

Supplement: Supplementary file 6 [file DataSheet4.zip › Fig6B UI TRIM28.tif]

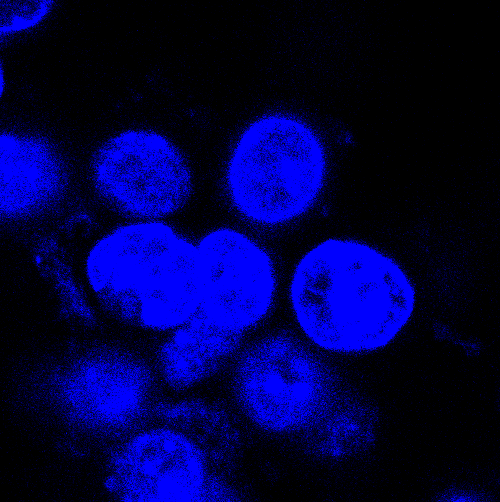

Supplement: Supplementary file 6 [file DataSheet4.zip › Fig6B VSV DAPI.tif]

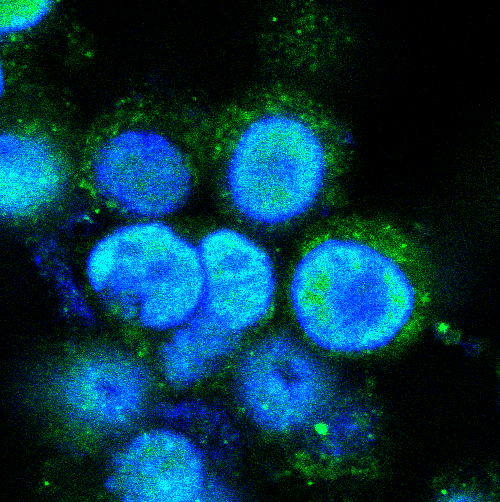

Supplement: Supplementary file 6 [file DataSheet4.zip › Fig6B VSV Merge.tif]

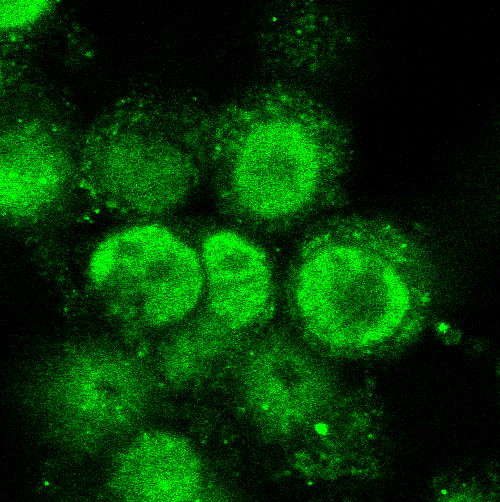

Supplement: Supplementary file 6 [file DataSheet4.zip › Fig6B VSV TRIM28.tif]

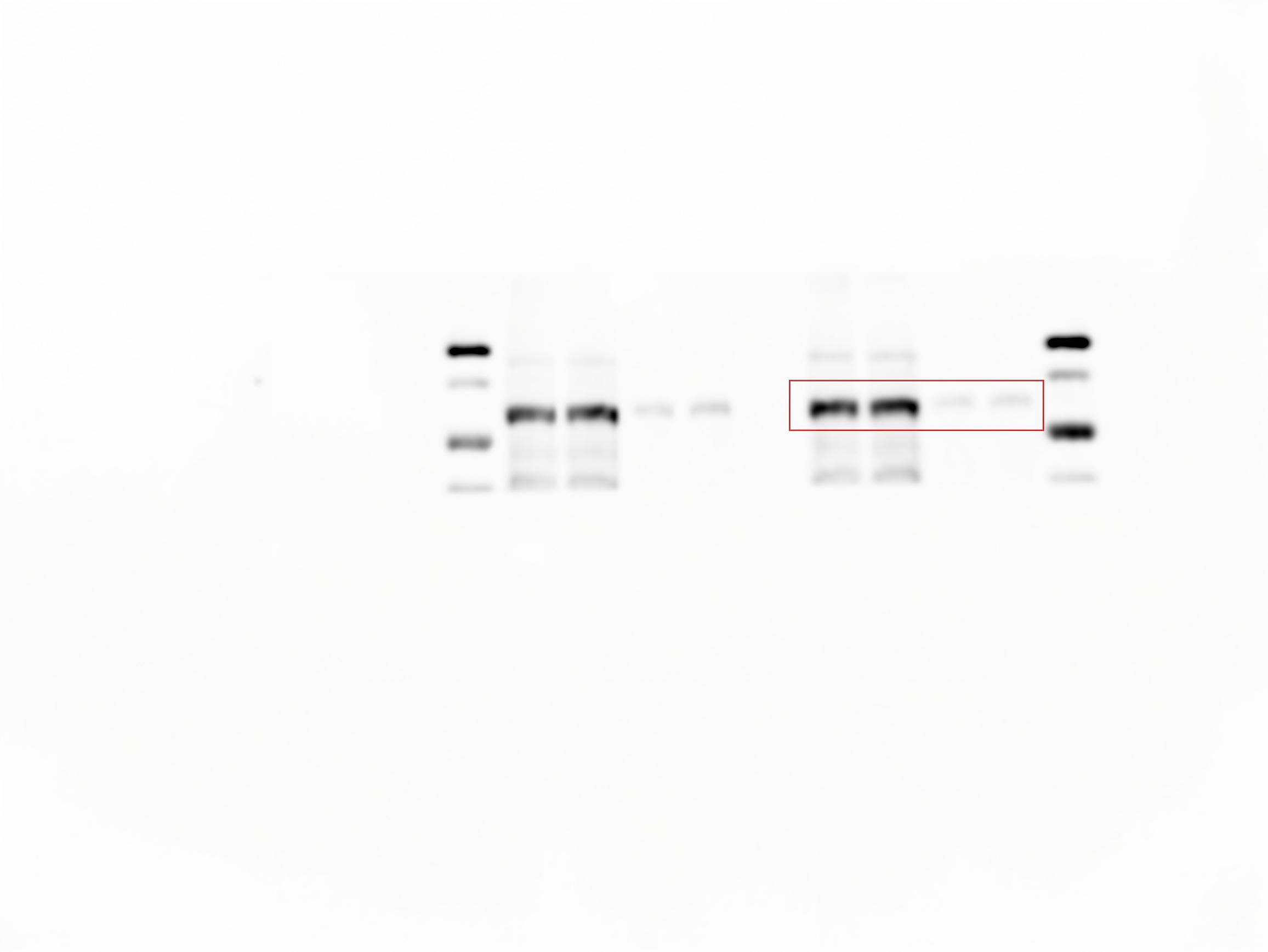

Supplement: Supplementary file 6 [file DataSheet4.zip › Fig6D Actin edited showing band.jpg]

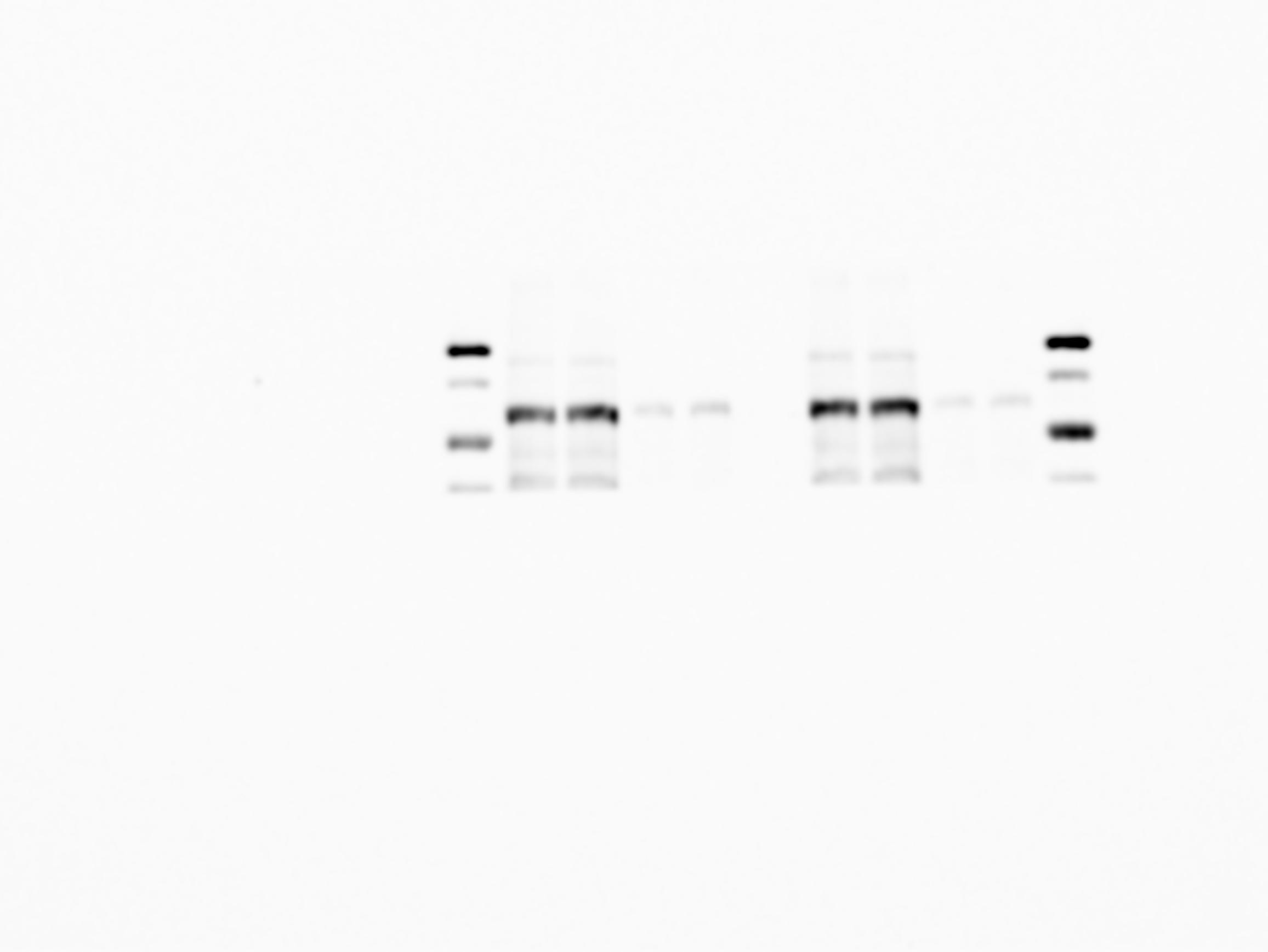

Supplement: Supplementary file 6 [file DataSheet4.zip › Fig6D Actin.jpg]

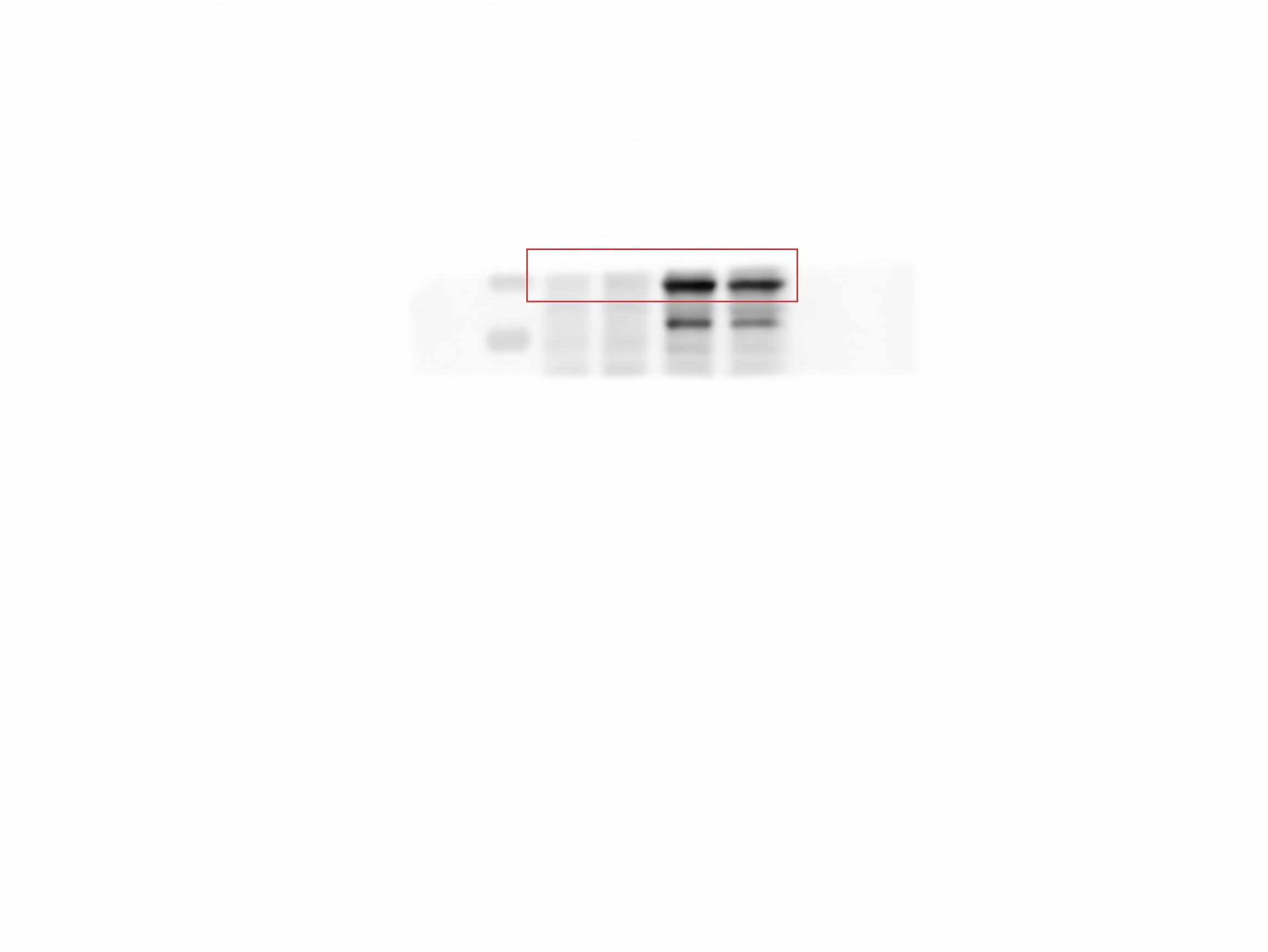

Supplement: Supplementary file 6 [file DataSheet4.zip › Fig6D Lamin B edited showing band.jpg]

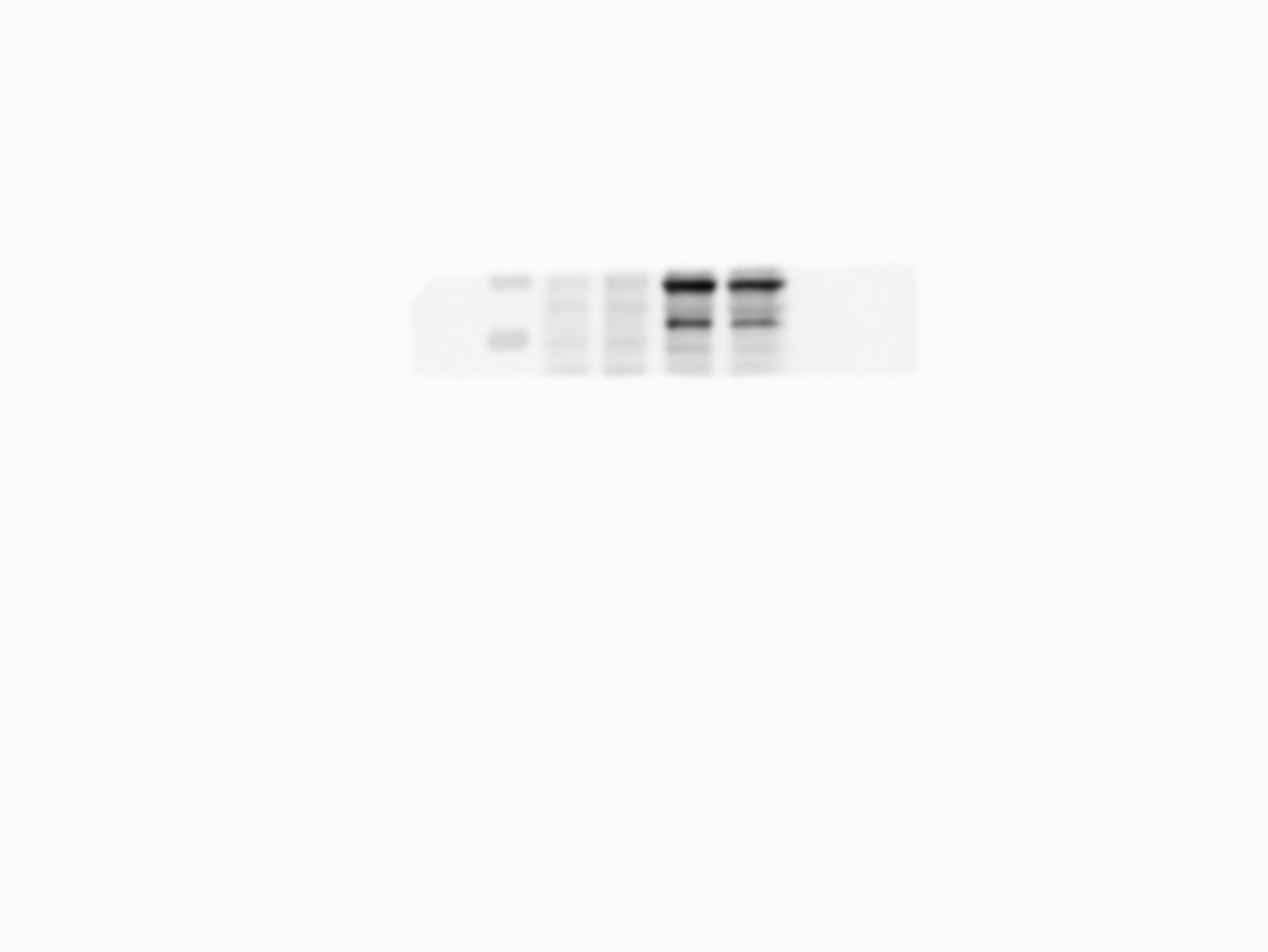

Supplement: Supplementary file 6 [file DataSheet4.zip › Fig6D Lamin B.tif]

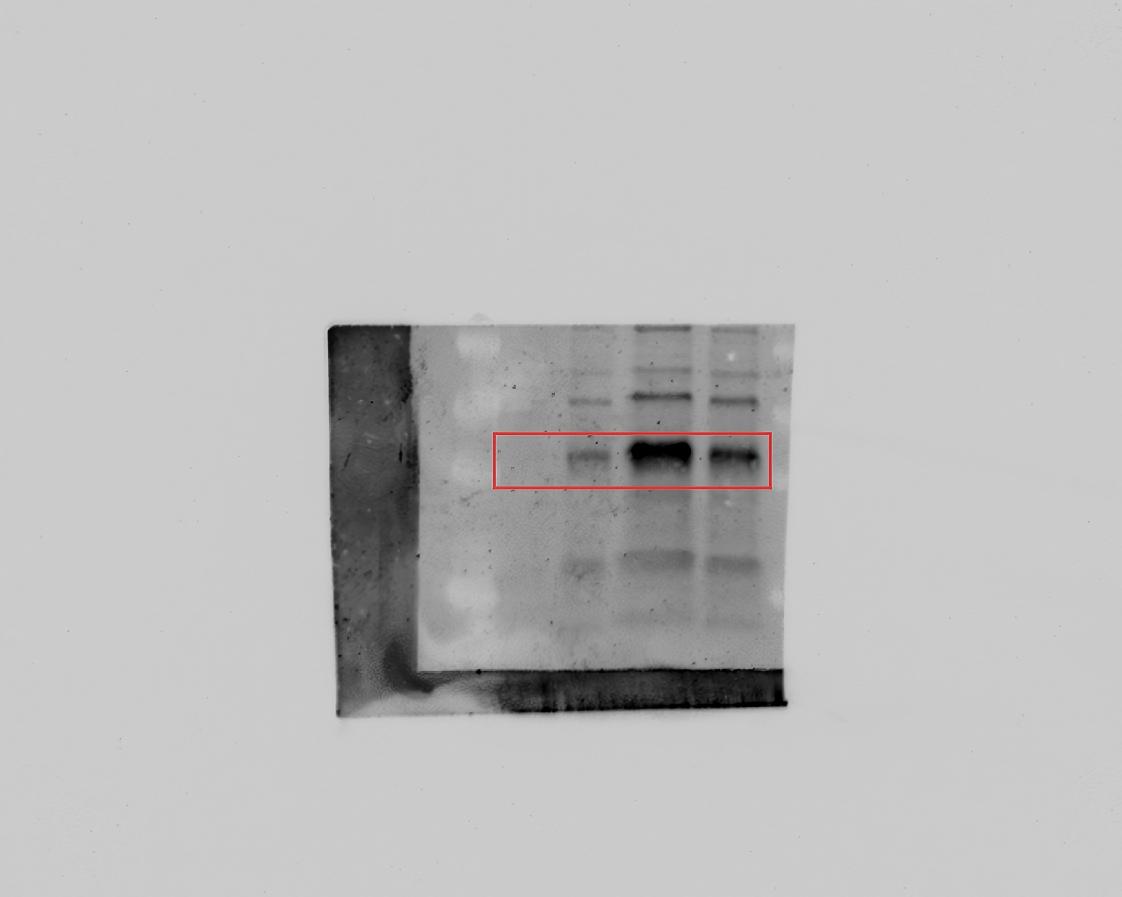

Supplement: Supplementary file 6 [file DataSheet4.zip › Fig6D TRIM28 edited showing band.jpg]

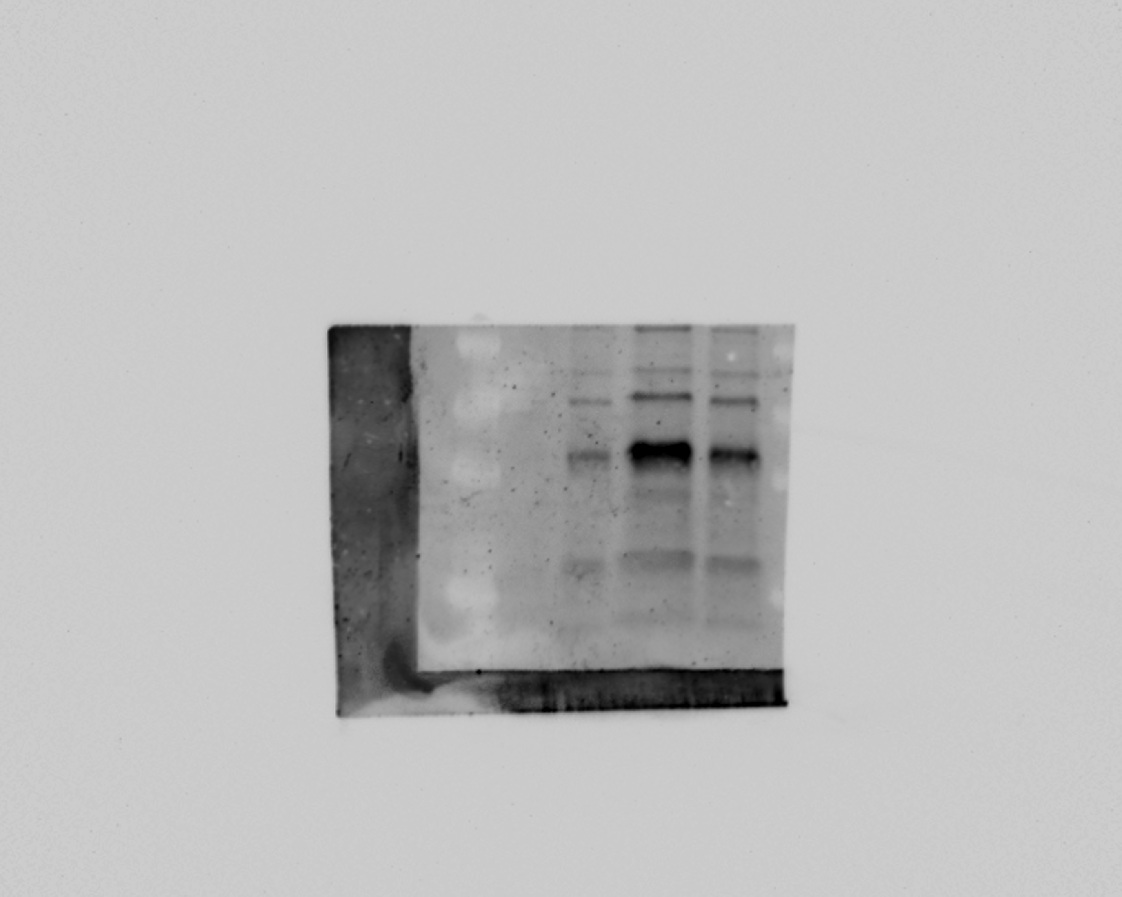

Supplement: Supplementary file 6 [file DataSheet4.zip › Fig6D TRIM28.jpg]

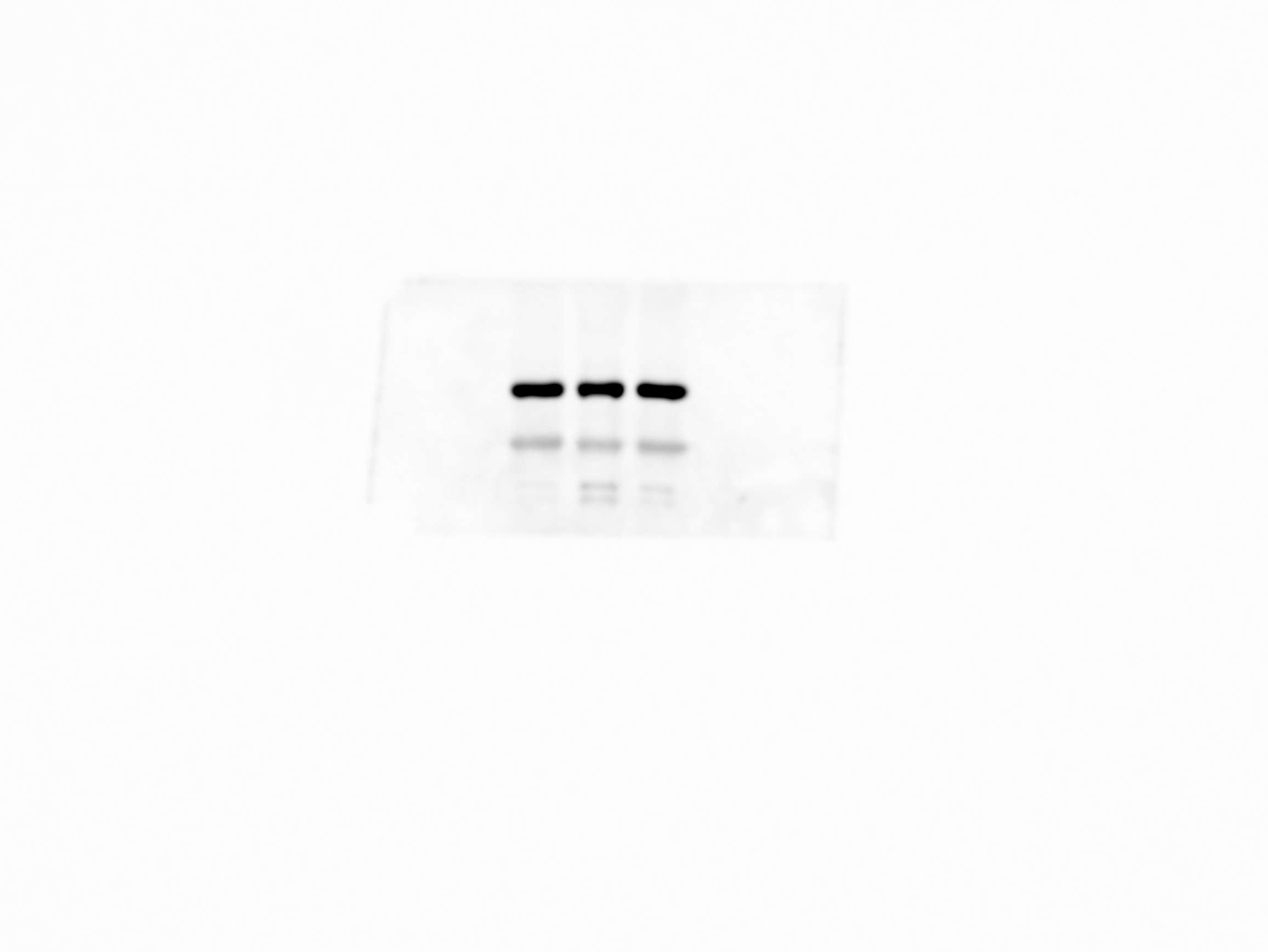

Supplement: Supplementary file 6 [file DataSheet4.zip › Fig6F Input Actin.tif]

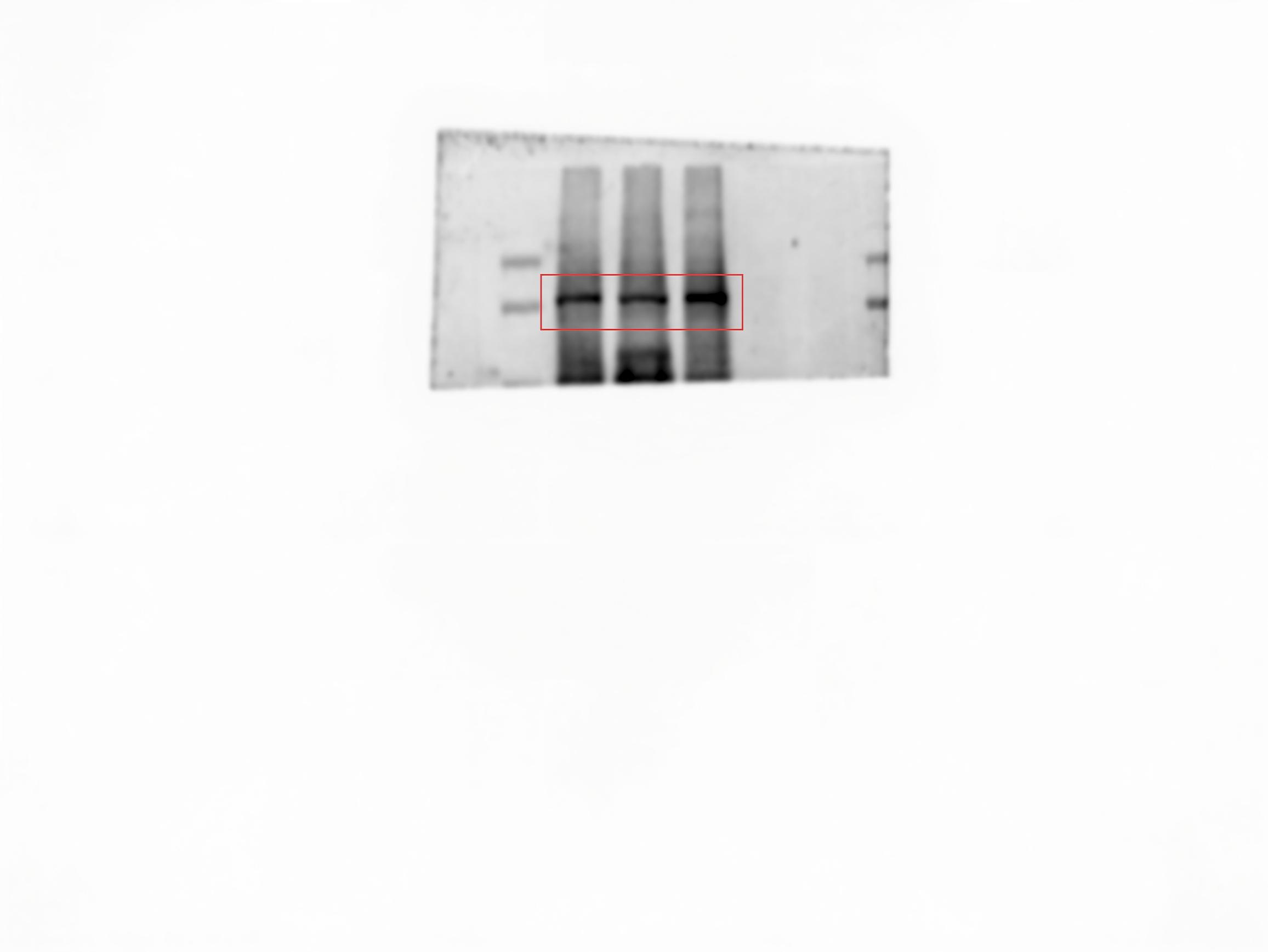

Supplement: Supplementary file 6 [file DataSheet4.zip › Fig6F Input CRM1 edited showing band.jpg]

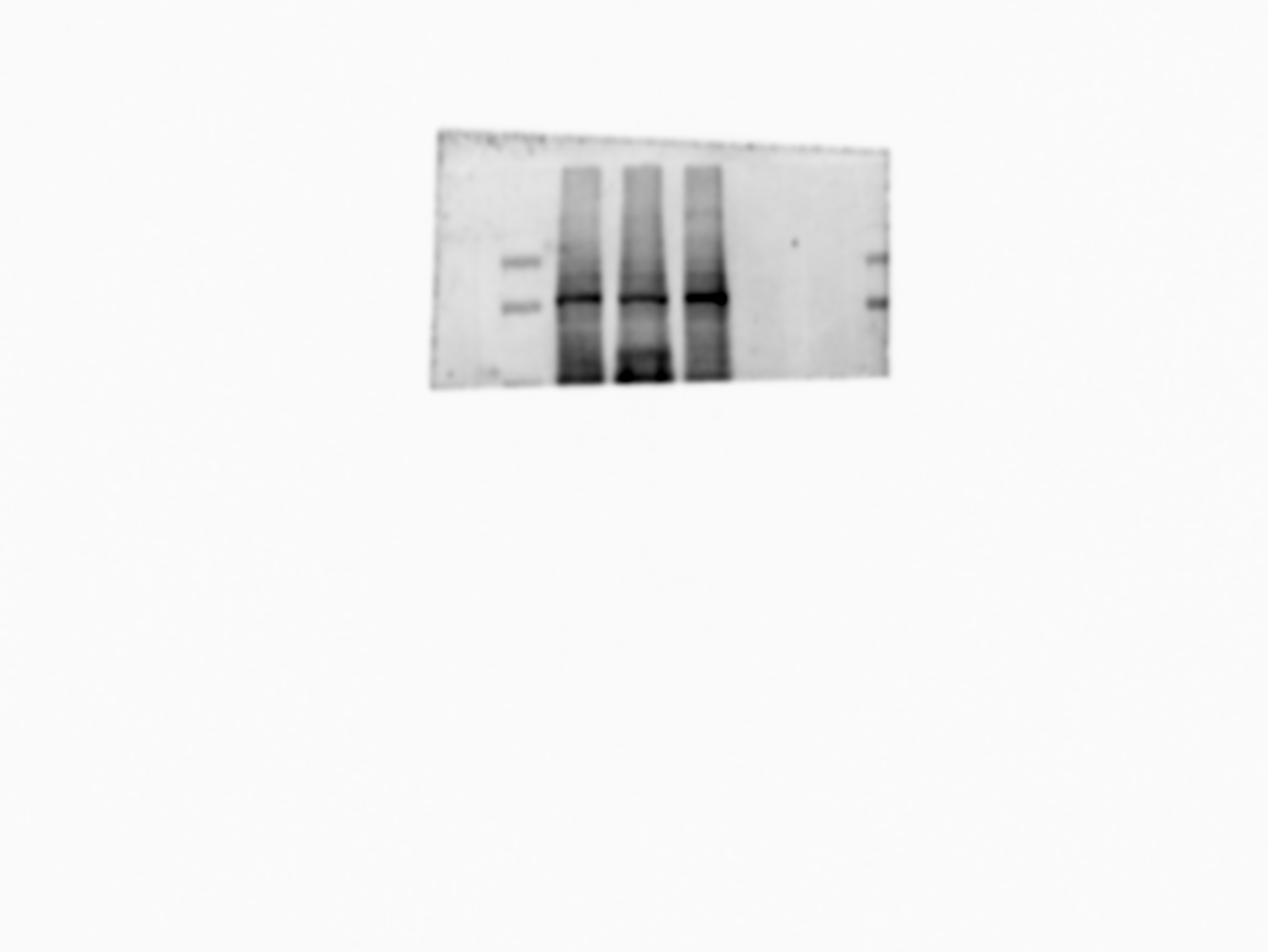

Supplement: Supplementary file 6 [file DataSheet4.zip › Fig6F Input CRM1.tif]

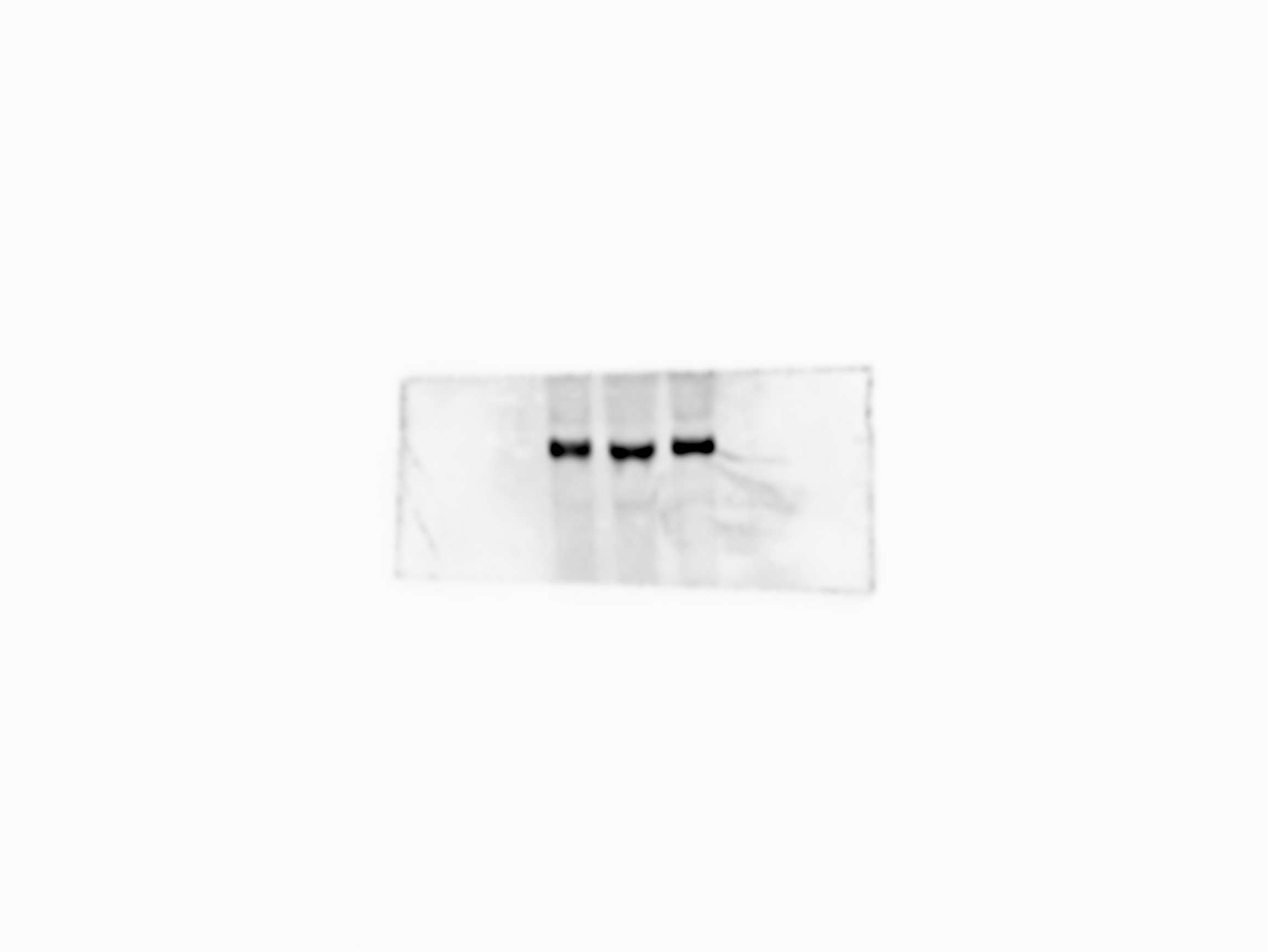

Supplement: Supplementary file 6 [file DataSheet4.zip › Fig6F Input TRIM28.tif]

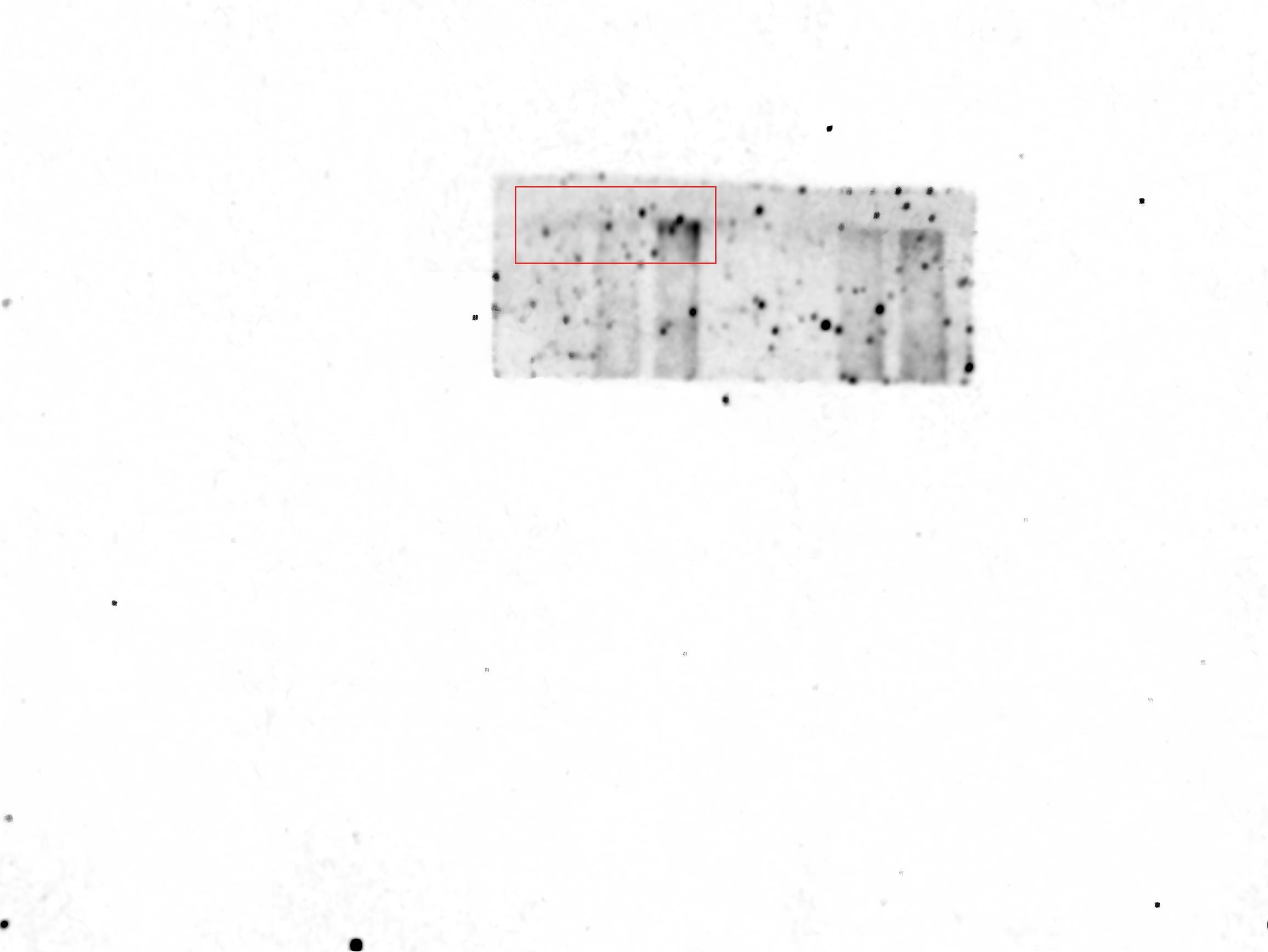

Supplement: Supplementary file 6 [file DataSheet4.zip › Fig6F IP CRM1 edited showing band.jpg]
