# Supplementary figures and images for: CRM1-dependent nuclear export of TRIM28 promotes MAVS K48-linked ubiquitination and suppresses RIG-I-mediated antiviral response (part 3 of 3)
Source: Front Immunol. 2026 Mar 24;17:1744833. doi: 10.3389/fimmu.2026.1744833 (PMC13053320; doi:10.3389/fimmu.2026.1744833)

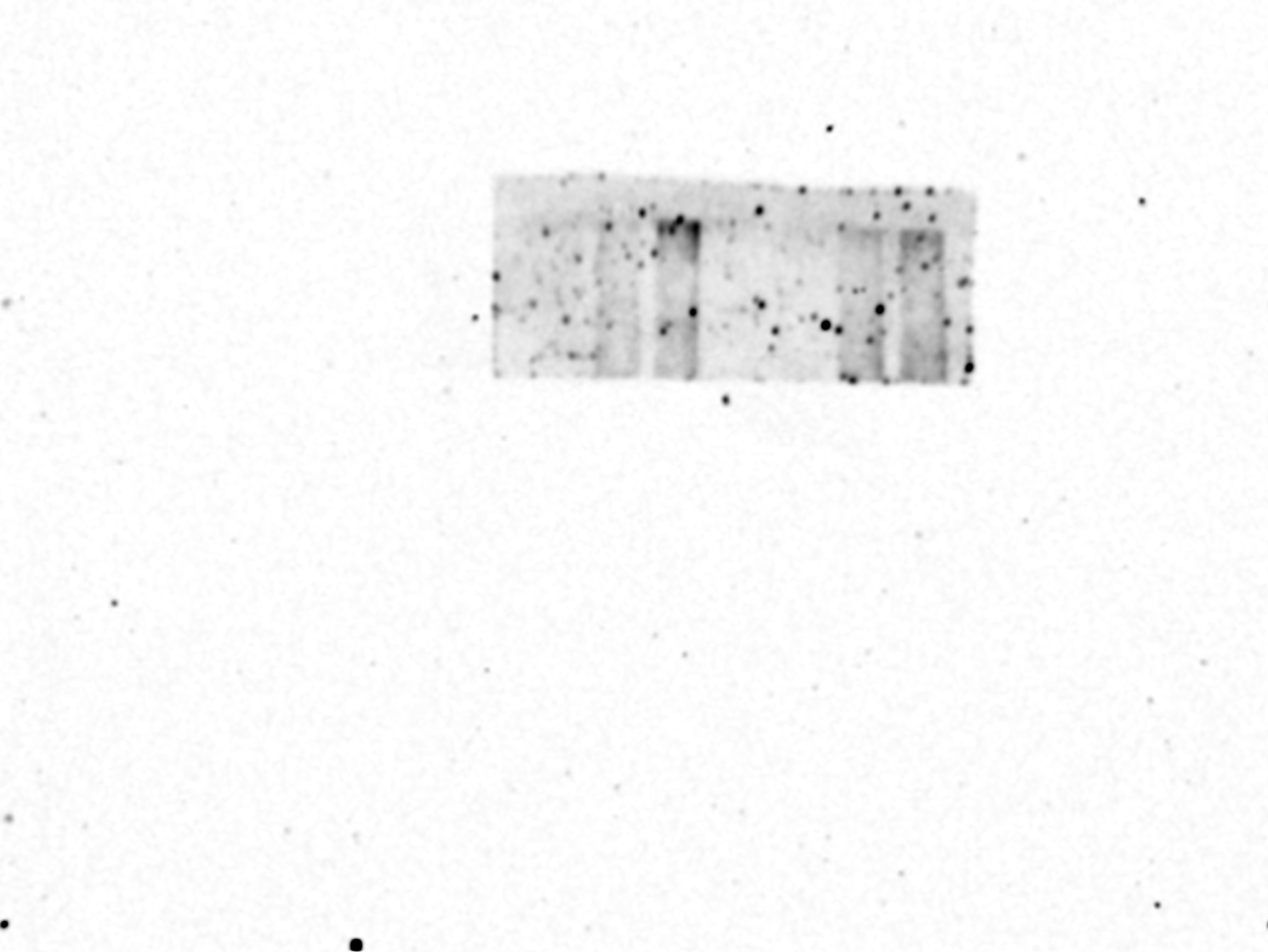

Supplement: Supplementary file 6 [file DataSheet4.zip › Fig6F IP CRM1.tif]

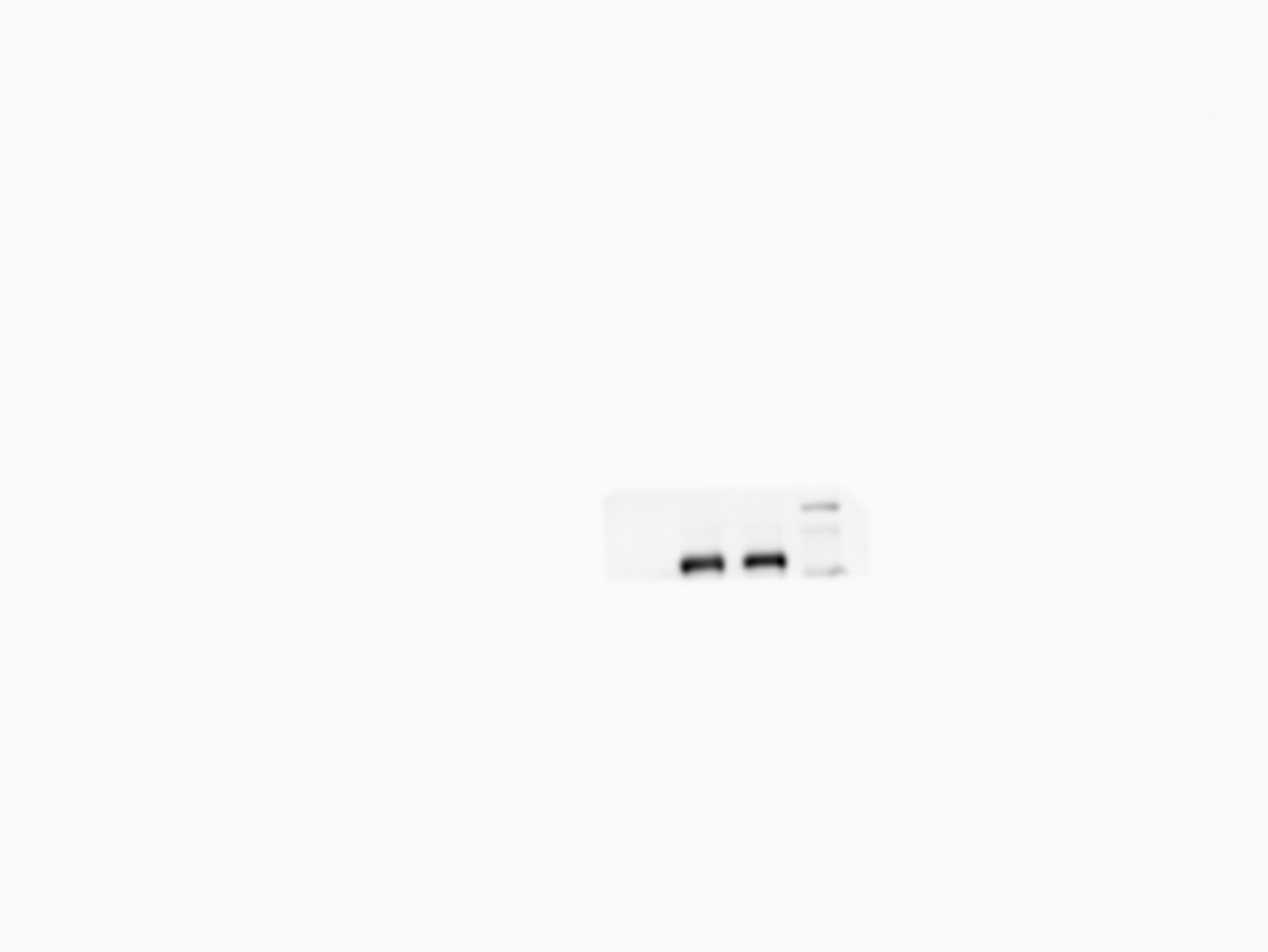

Supplement: Supplementary file 6 [file DataSheet4.zip › Fig6F IP TRIM28.jpg]

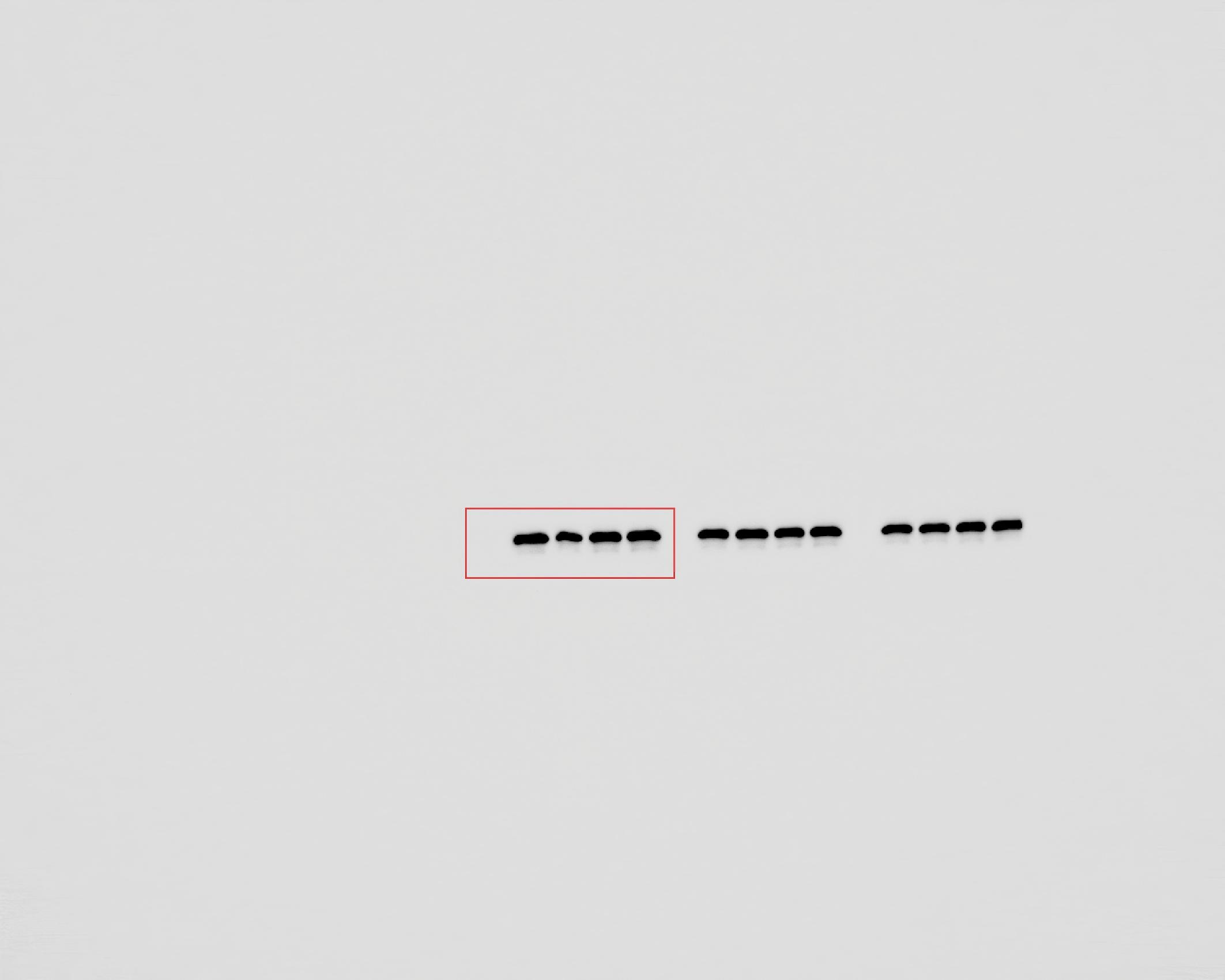

Supplement: Supplementary file 6 [file DataSheet4.zip › Fig6G CE Actin edited showing band.jpg]

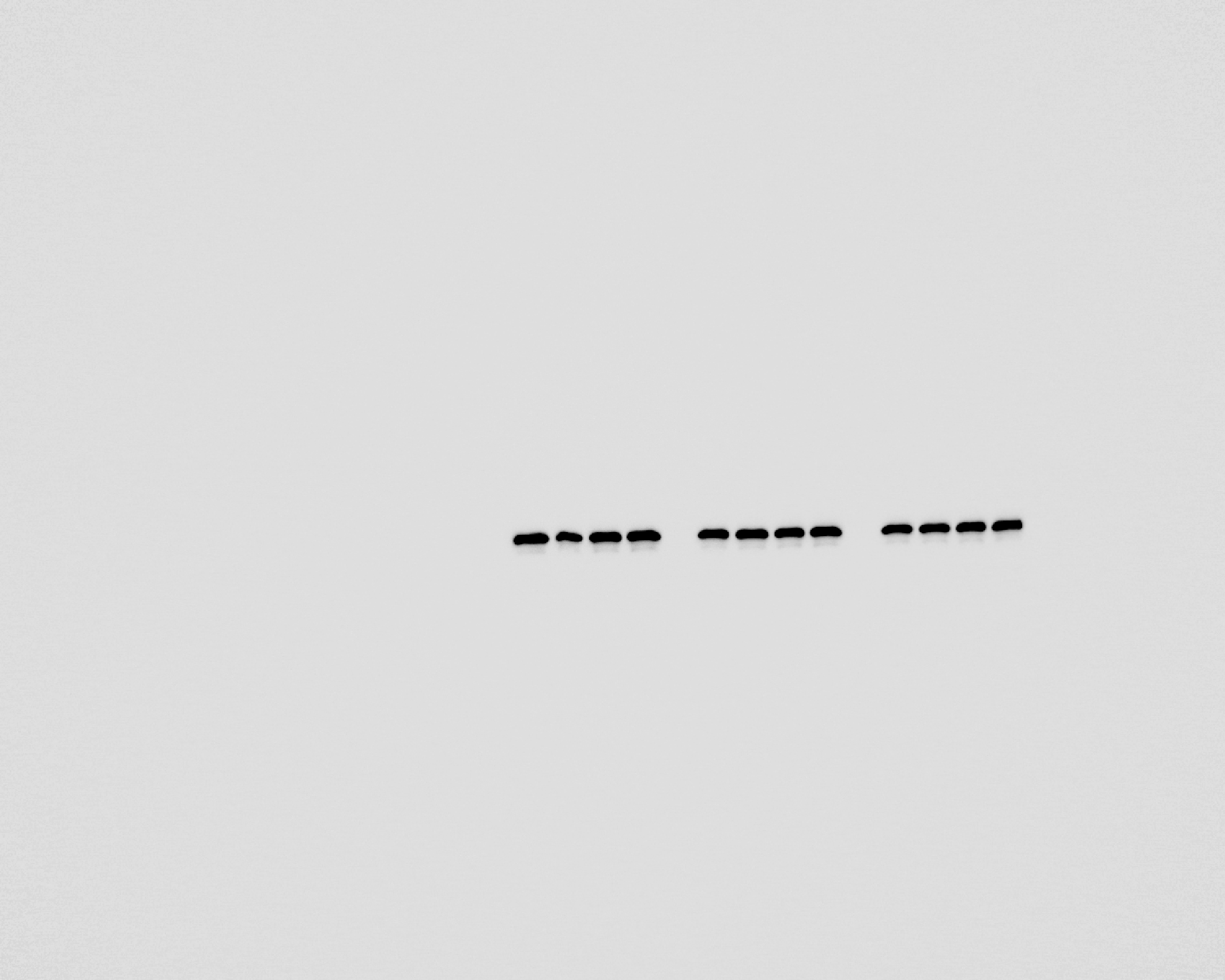

Supplement: Supplementary file 6 [file DataSheet4.zip › Fig6G CE Actin.tif]

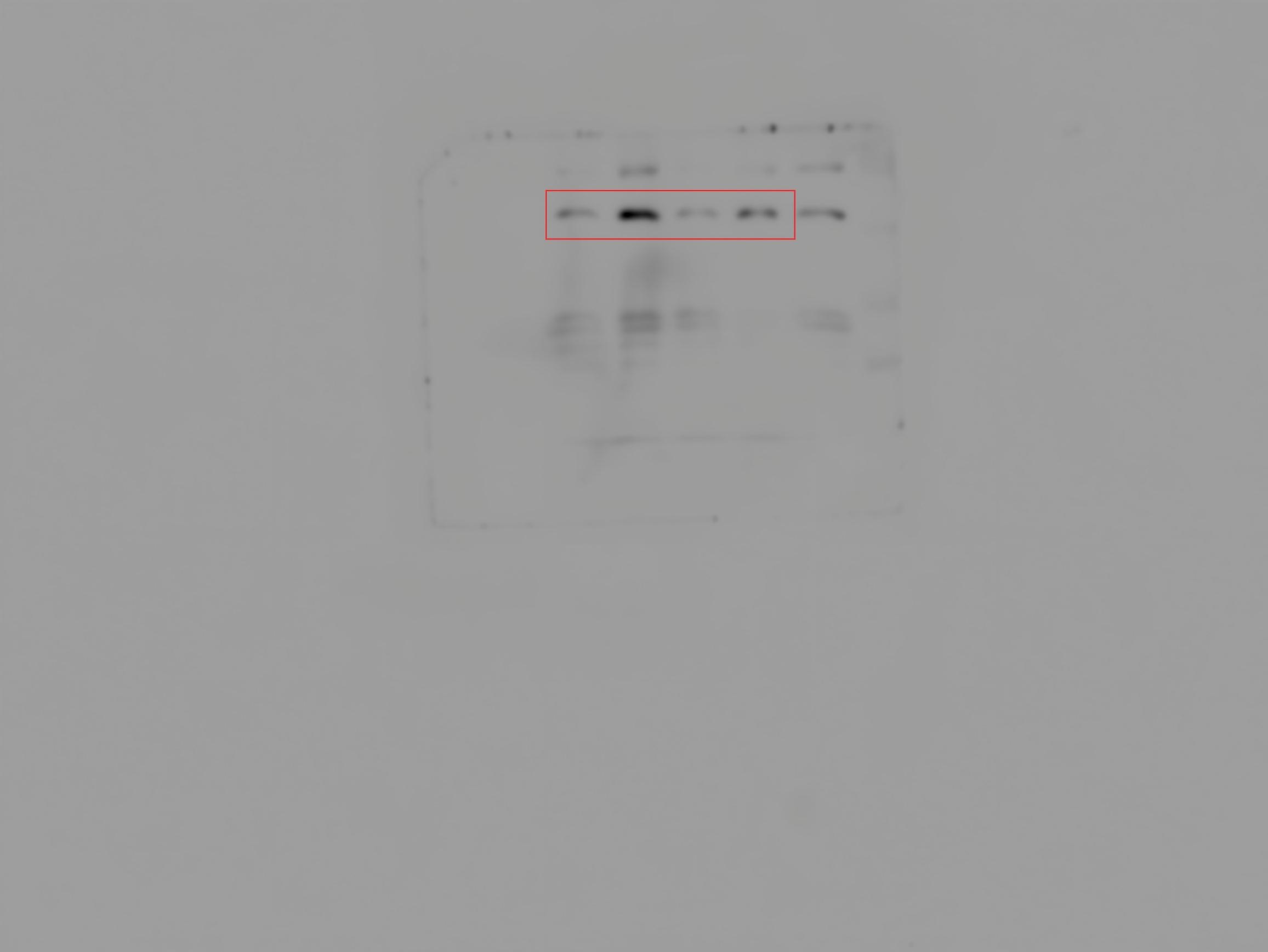

Supplement: Supplementary file 6 [file DataSheet4.zip › Fig6G CE TRIM28 edited showing band.jpg]

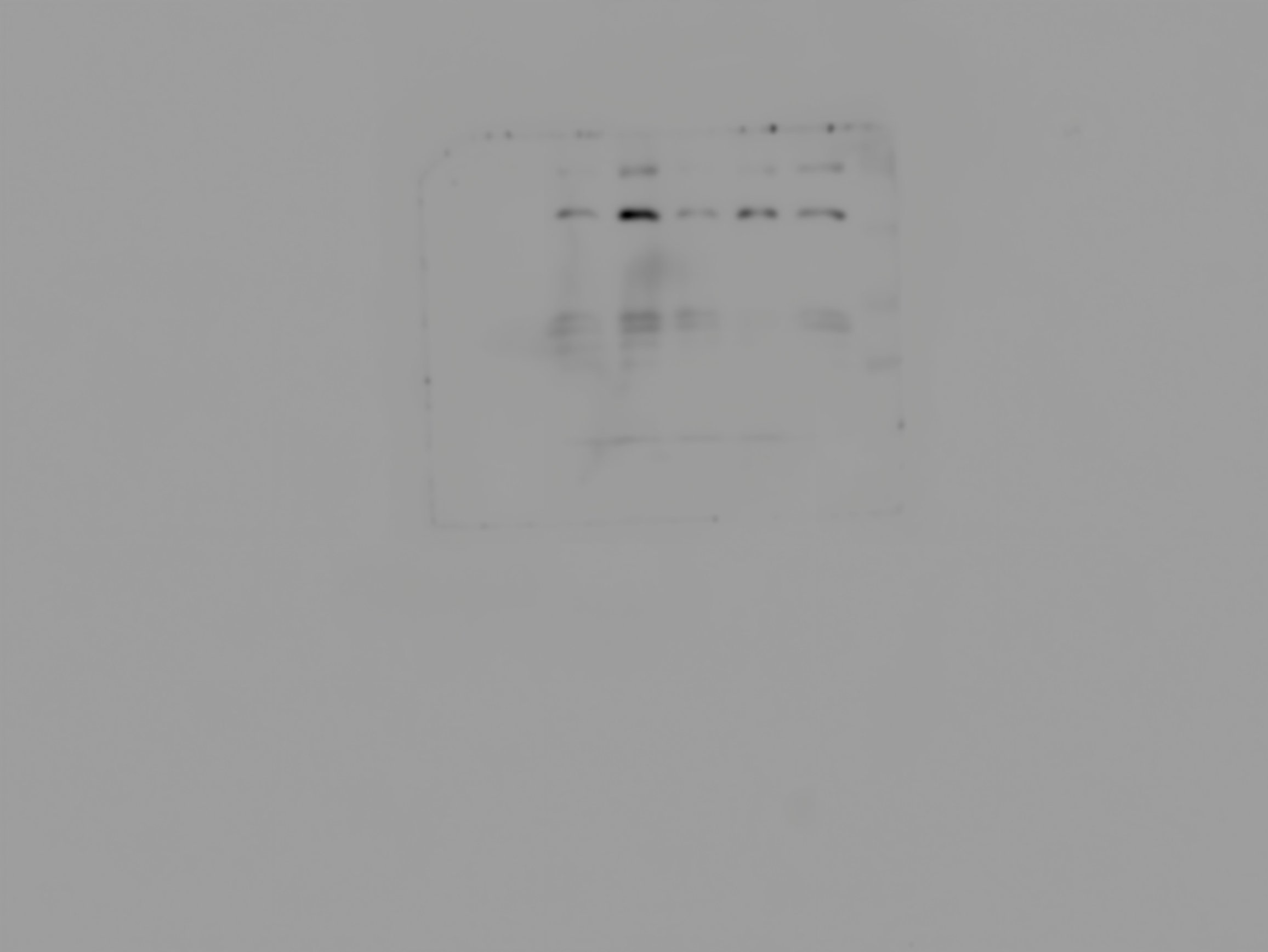

Supplement: Supplementary file 6 [file DataSheet4.zip › Fig6G CE TRIM28.png]

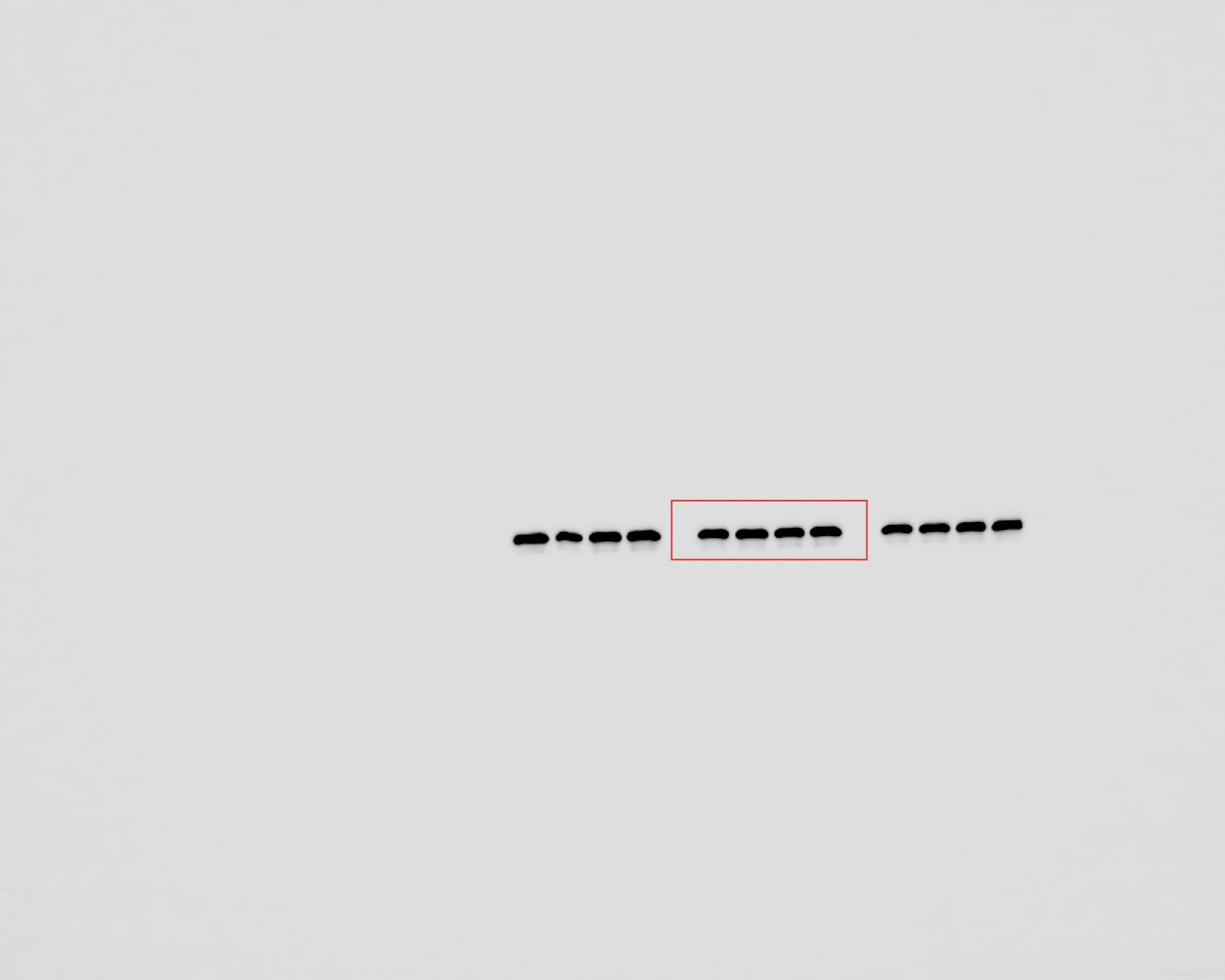

Supplement: Supplementary file 6 [file DataSheet4.zip › Fig6G WCL Actin edited showing band.jpg]

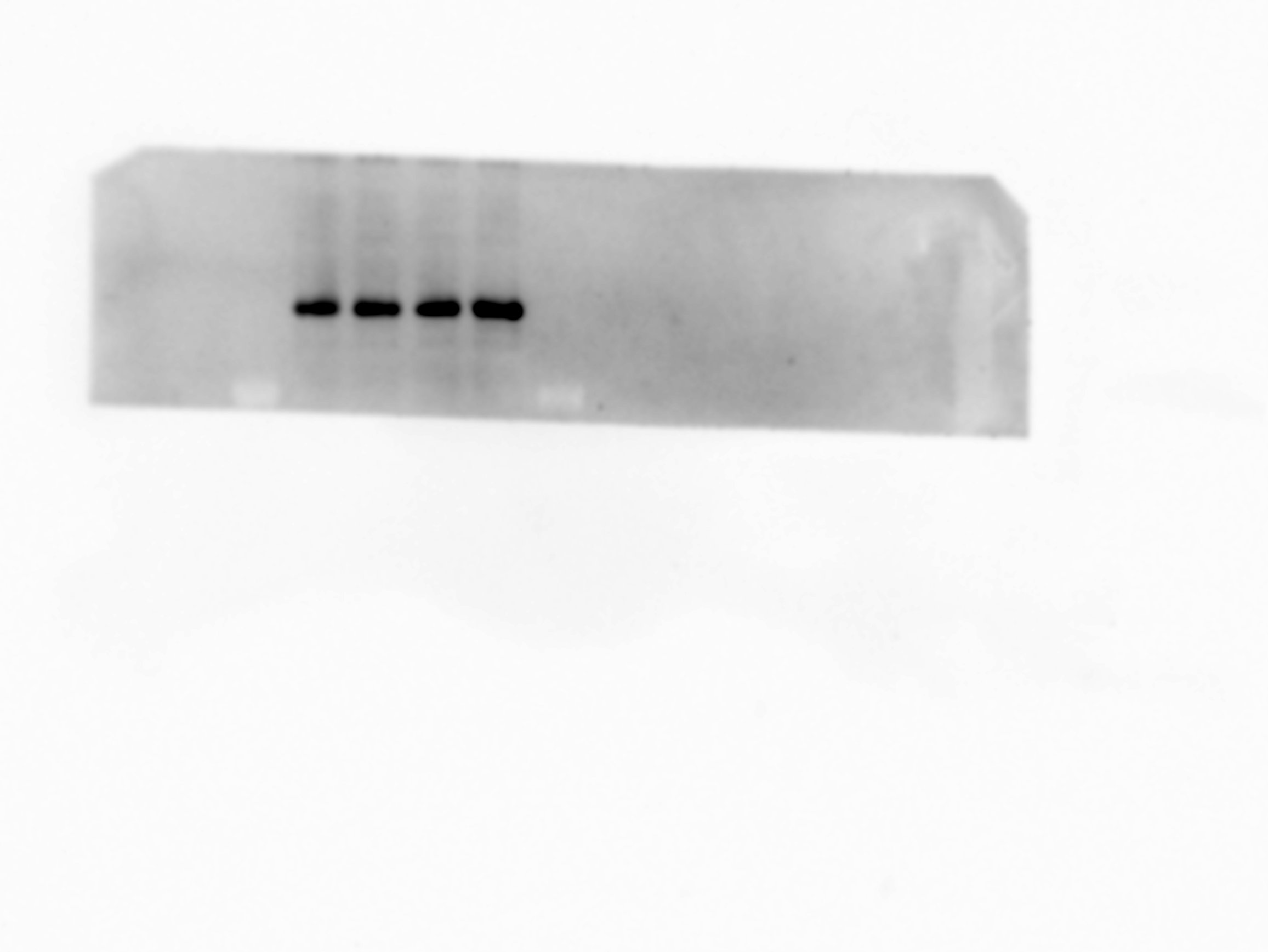

Supplement: Supplementary file 6 [file DataSheet4.zip › Fig6G WCL TRIM28.tif]

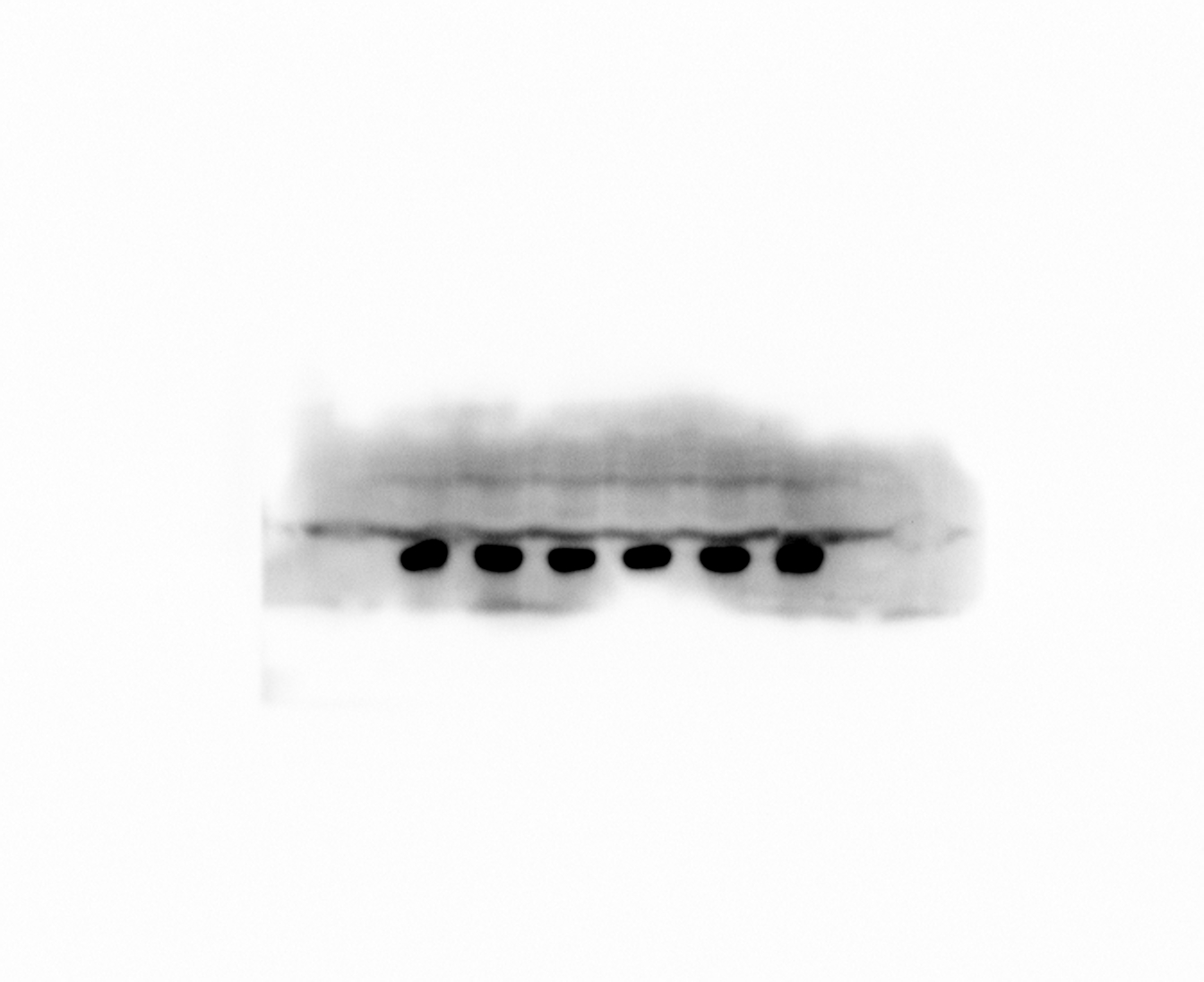

Supplement: Supplementary file 6 [file DataSheet4.zip › Fig7A Input Actin.jpg]

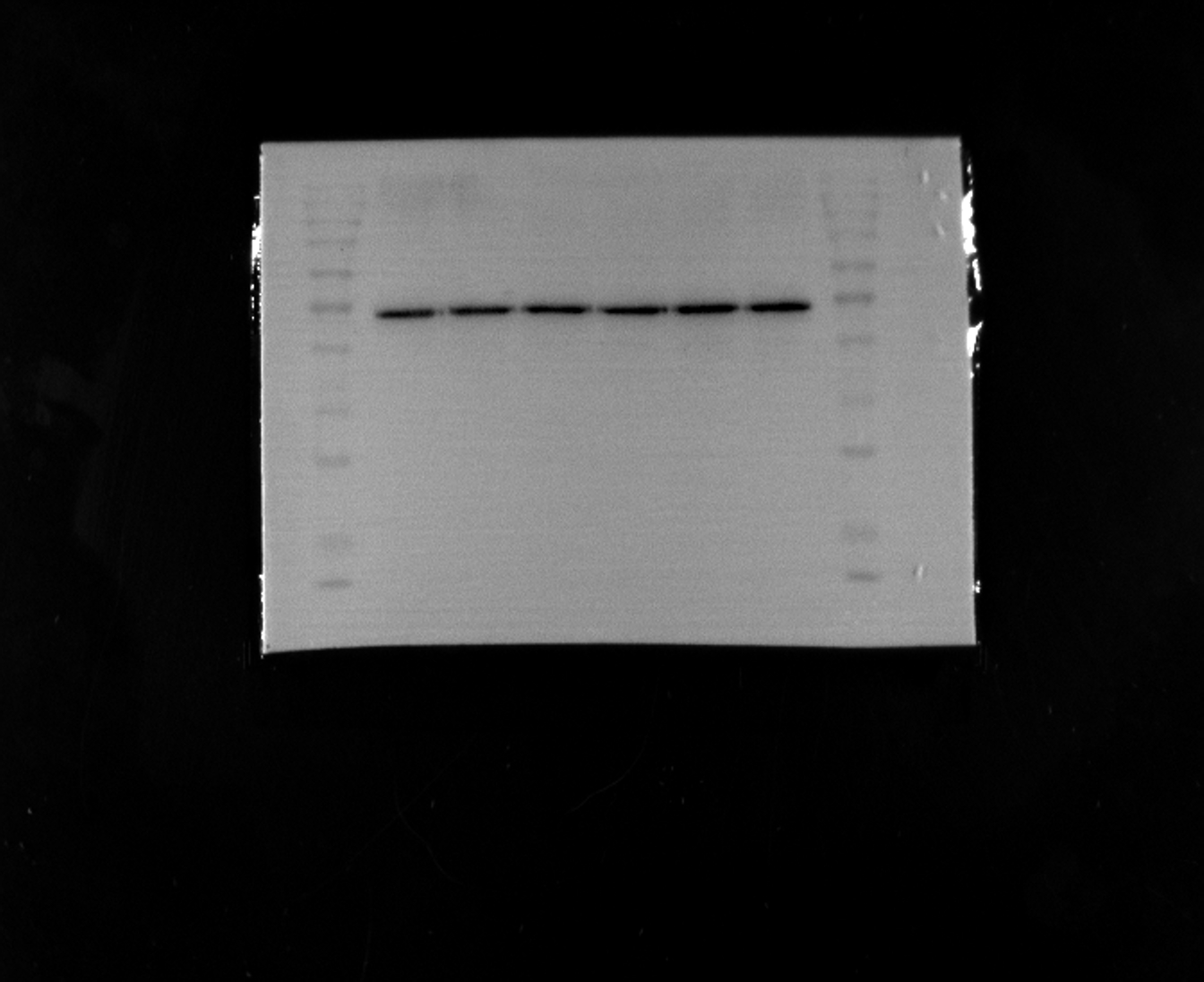

Supplement: Supplementary file 6 [file DataSheet4.zip › Fig7A Input HA.jpg]

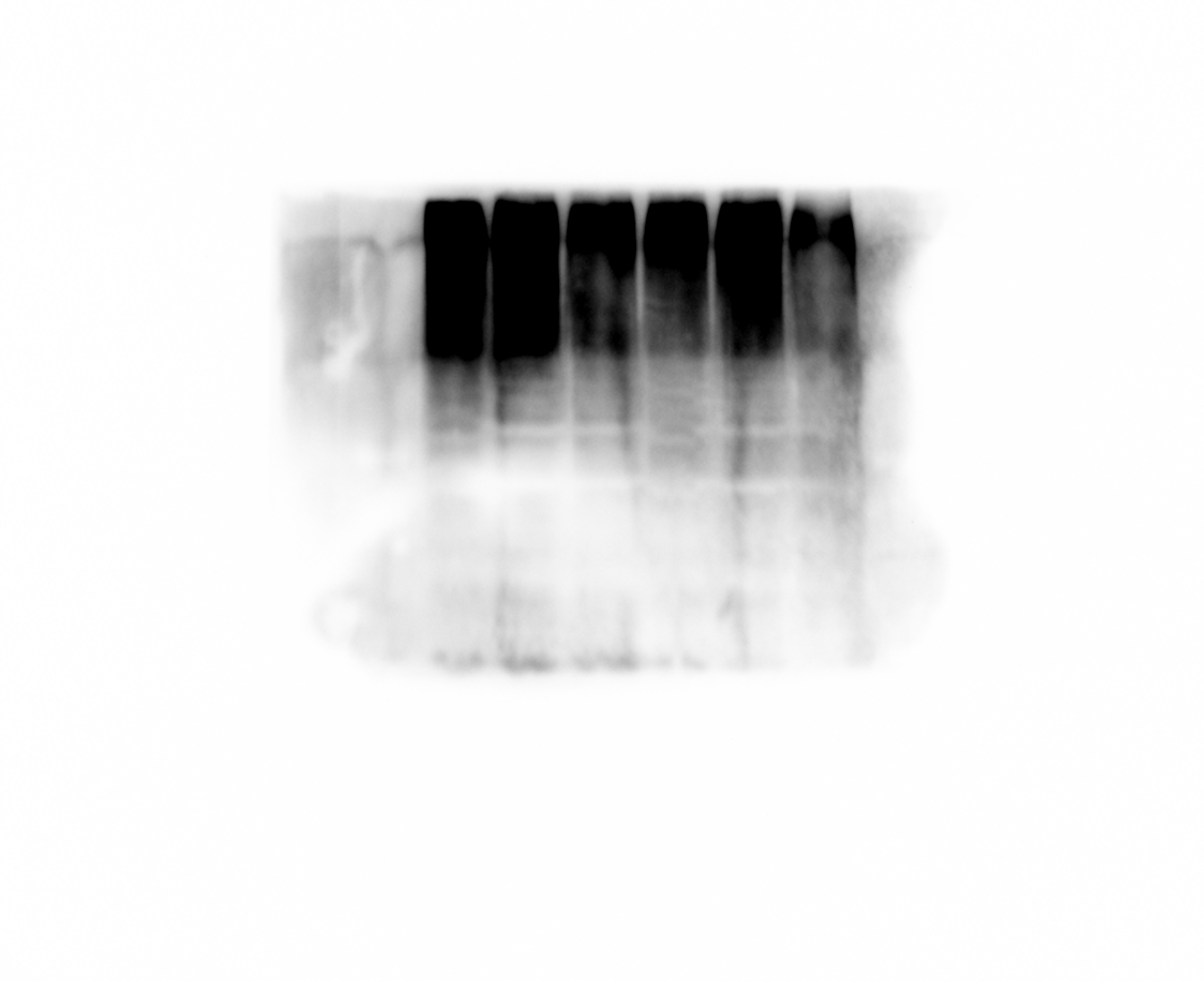

Supplement: Supplementary file 6 [file DataSheet4.zip › Fig7A Input His.jpg]

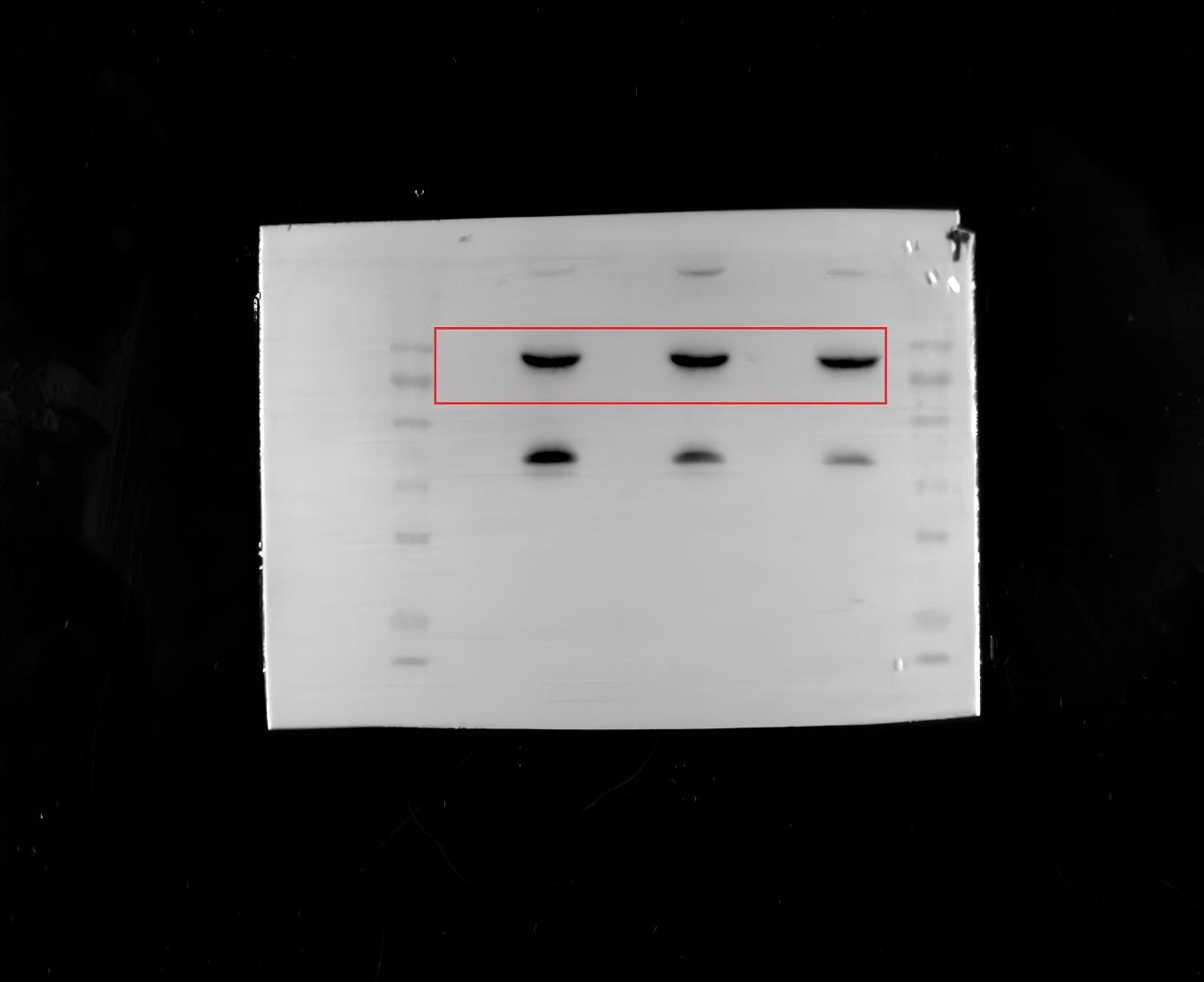

Supplement: Supplementary file 6 [file DataSheet4.zip › Fig7A Input Myc edited showing band.jpg]

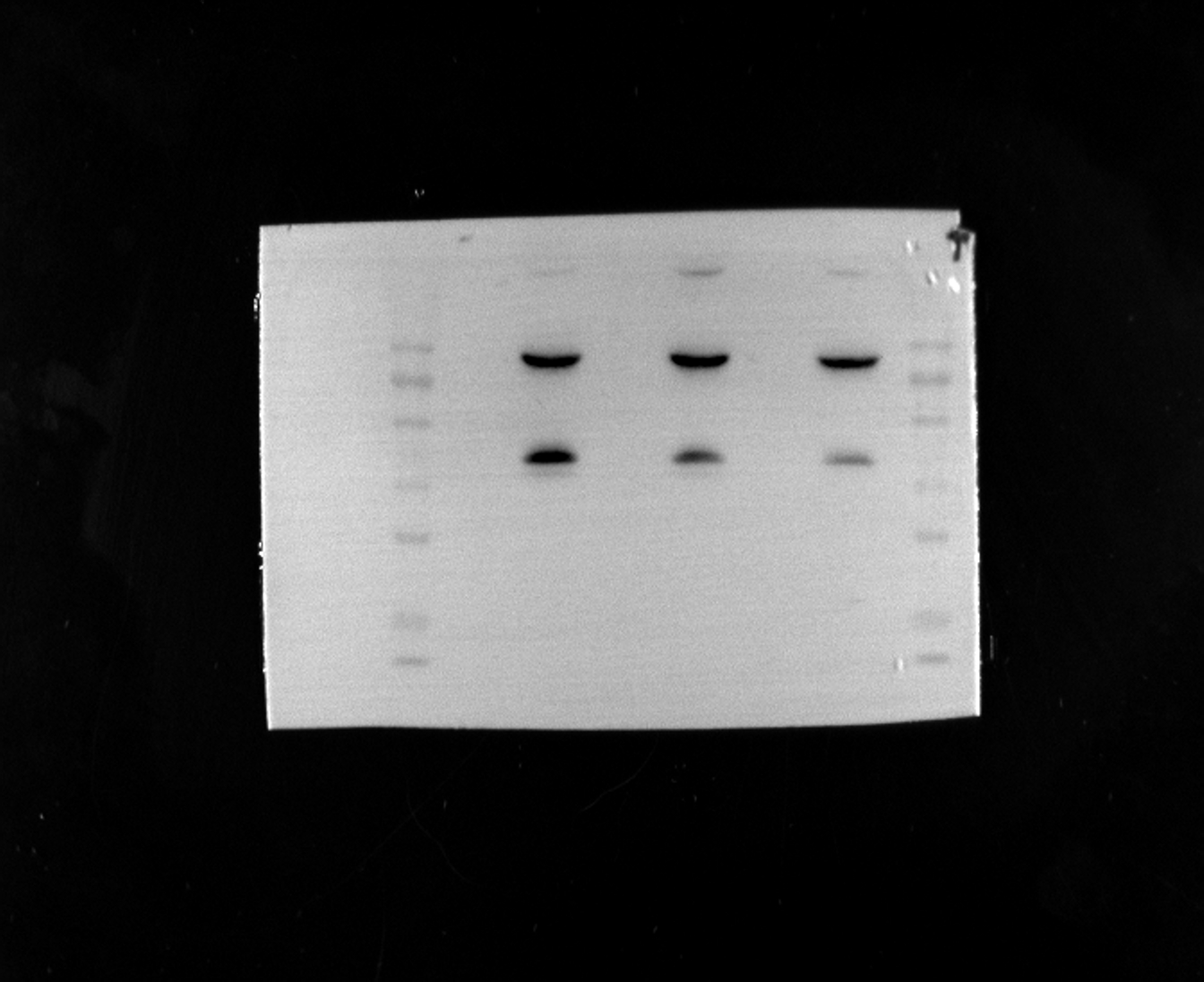

Supplement: Supplementary file 6 [file DataSheet4.zip › Fig7A Input Myc.jpg]

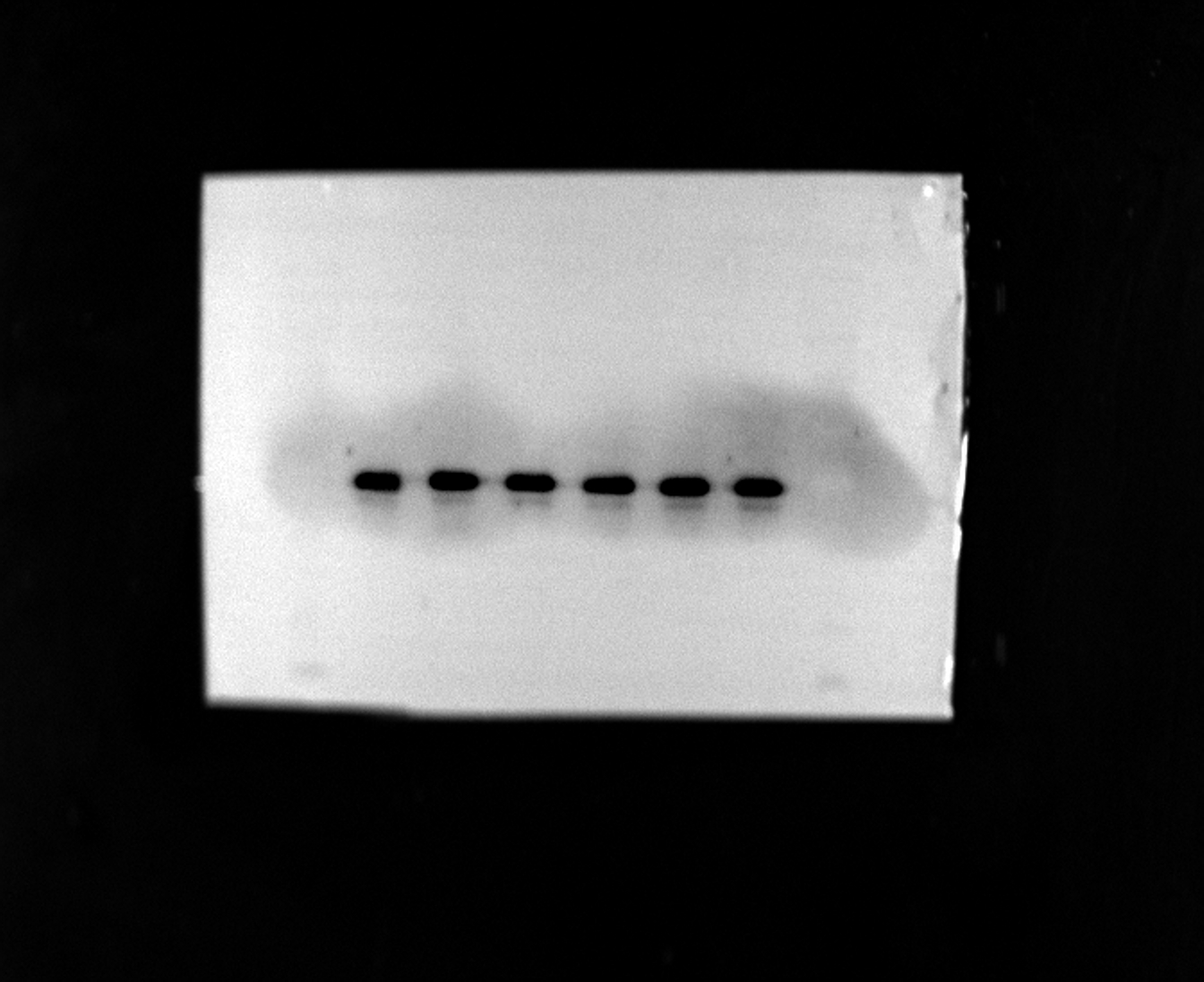

Supplement: Supplementary file 6 [file DataSheet4.zip › Fig7A IP HA.jpg]

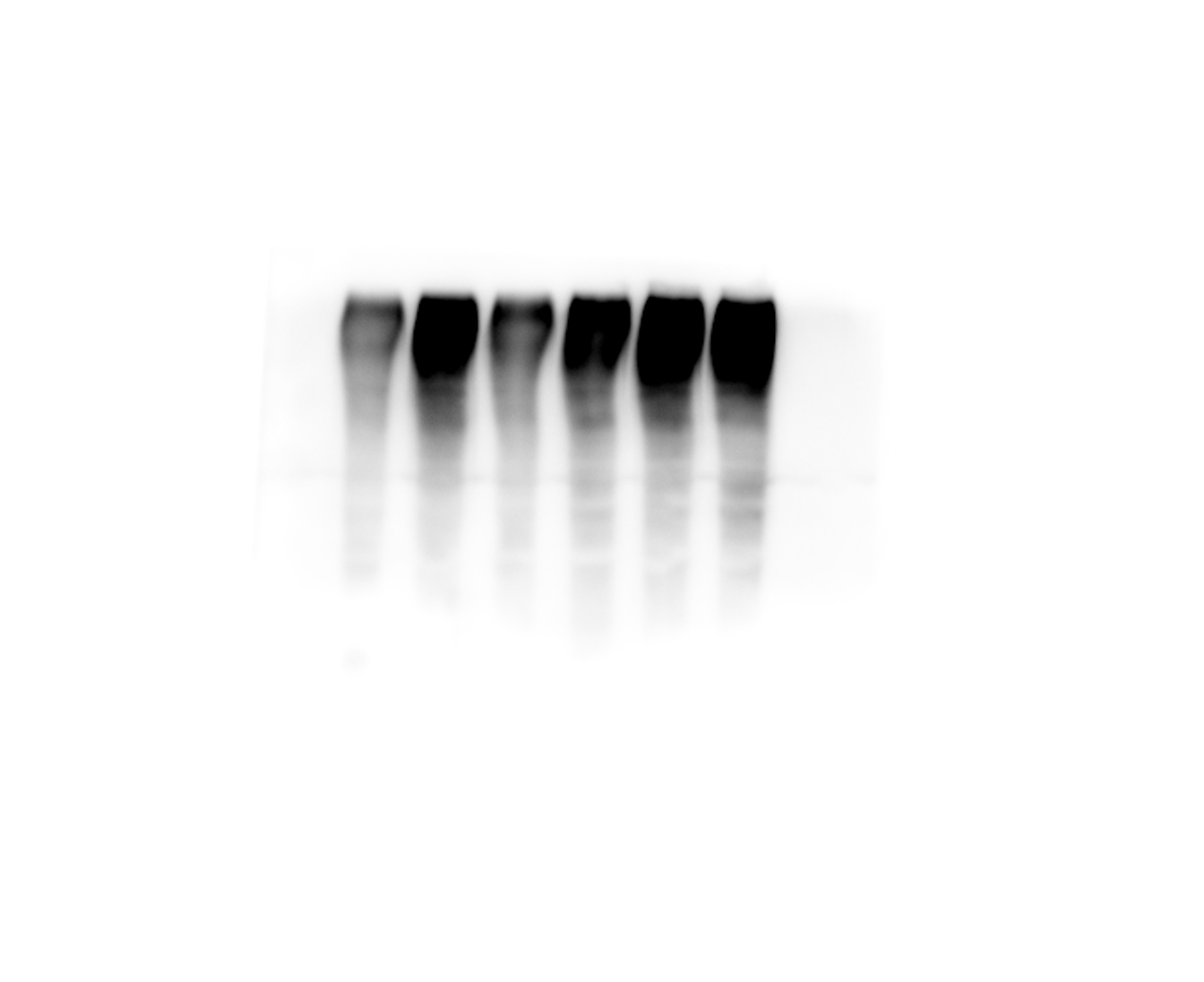

Supplement: Supplementary file 6 [file DataSheet4.zip › Fig7A IP His.jpg]

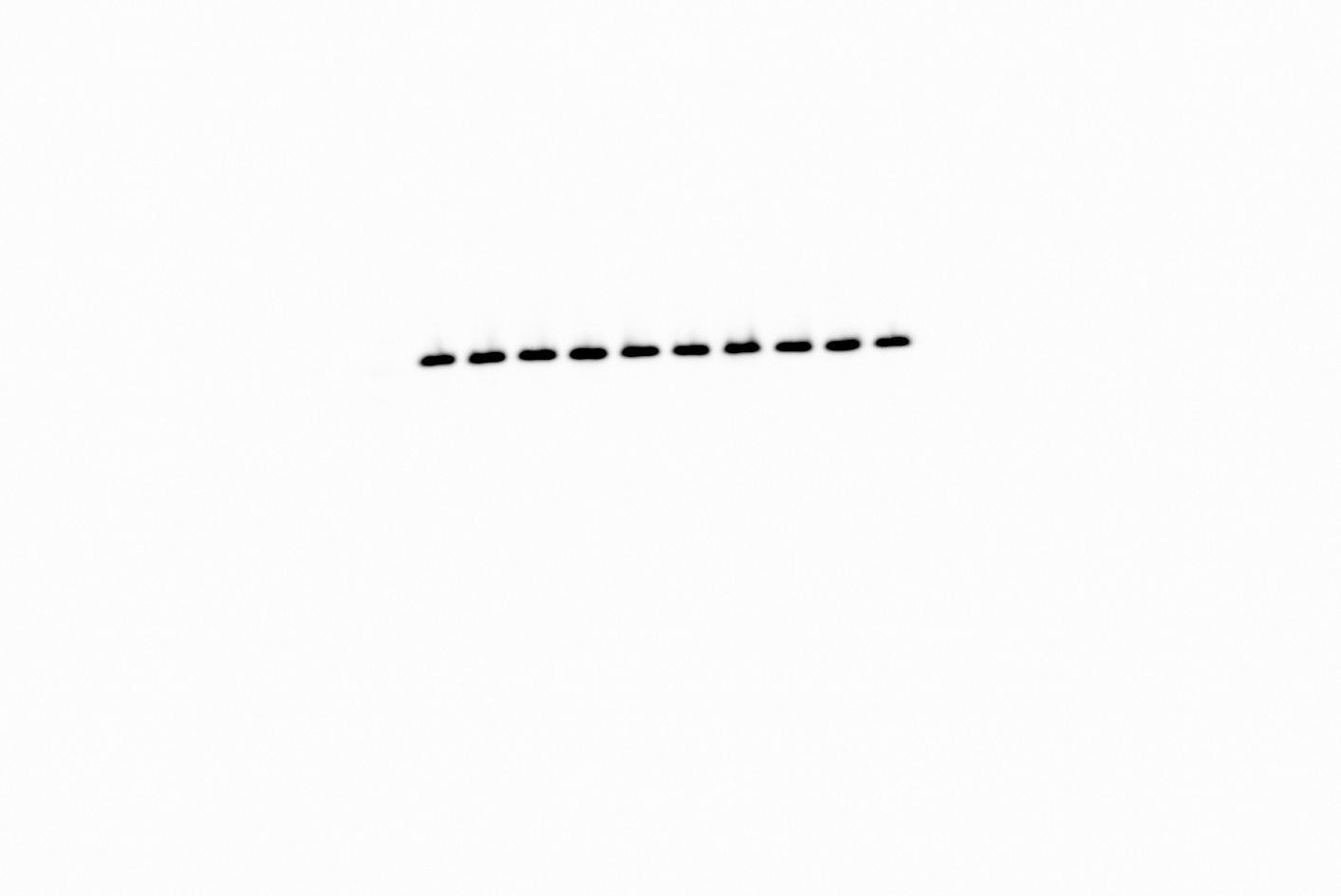

Supplement: Supplementary file 6 [file DataSheet4.zip › Fig7B Input HA.tif]

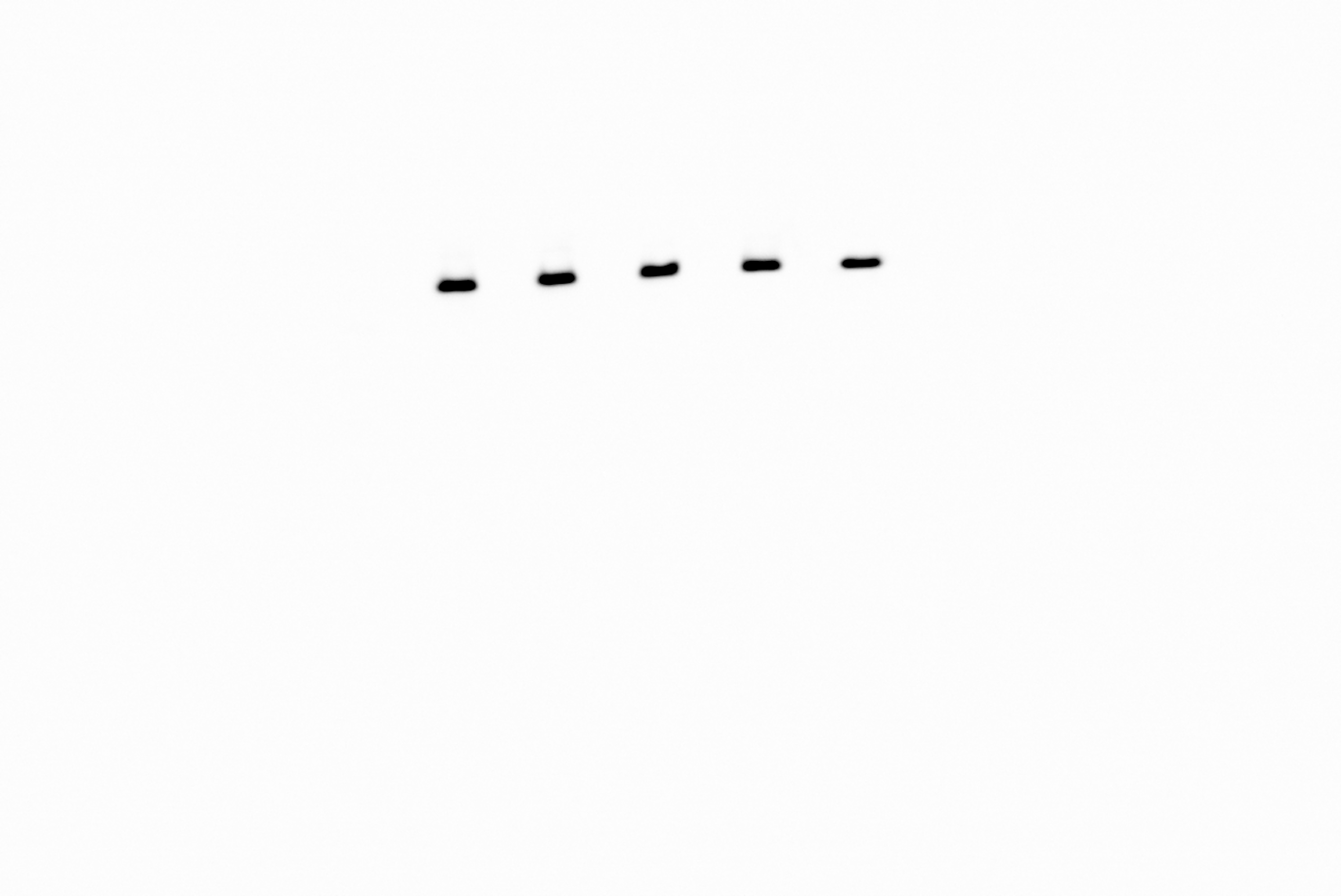

Supplement: Supplementary file 6 [file DataSheet4.zip › Fig7B Input Myc.tif]

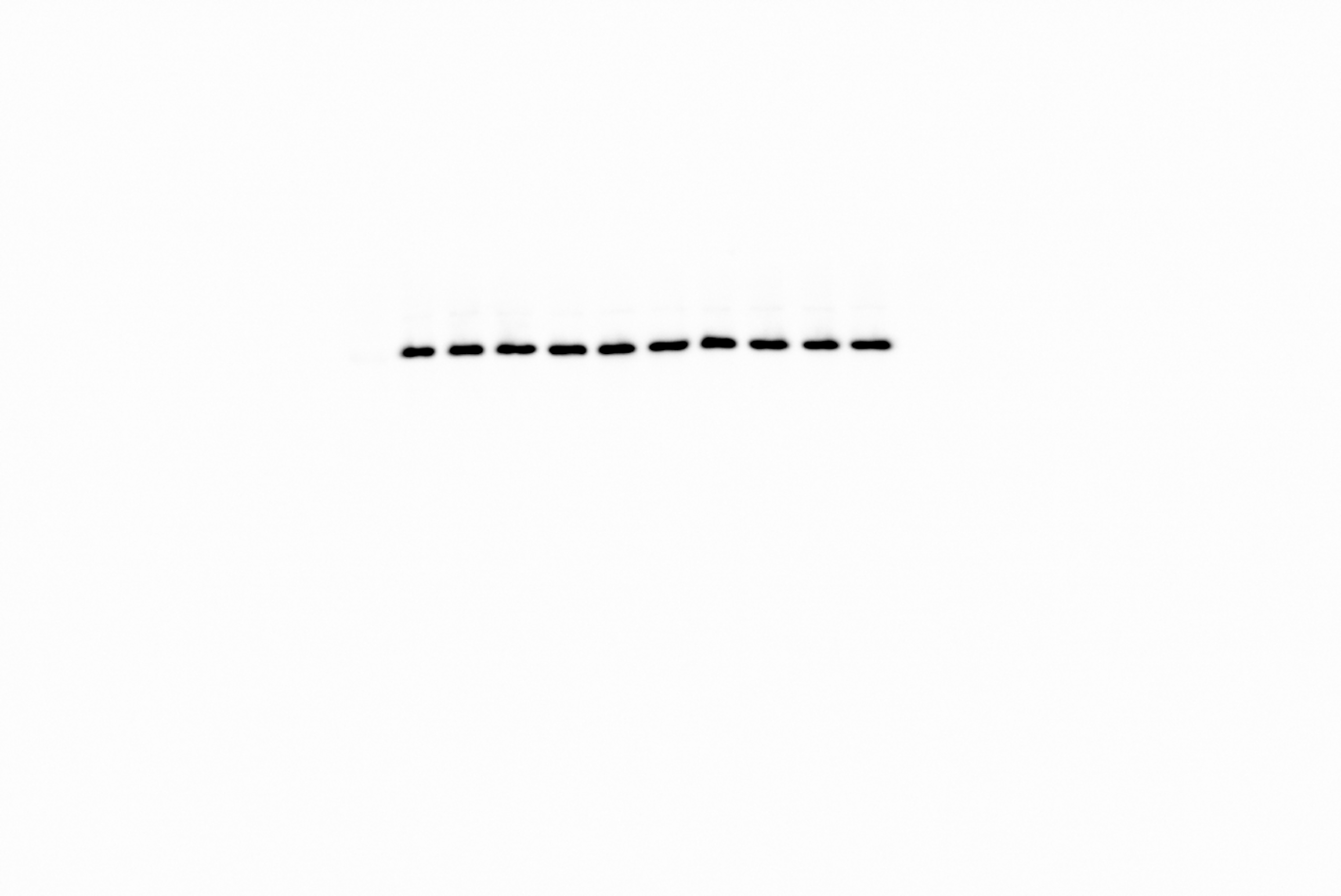

Supplement: Supplementary file 6 [file DataSheet4.zip › Fig7B Ip HA.tif]

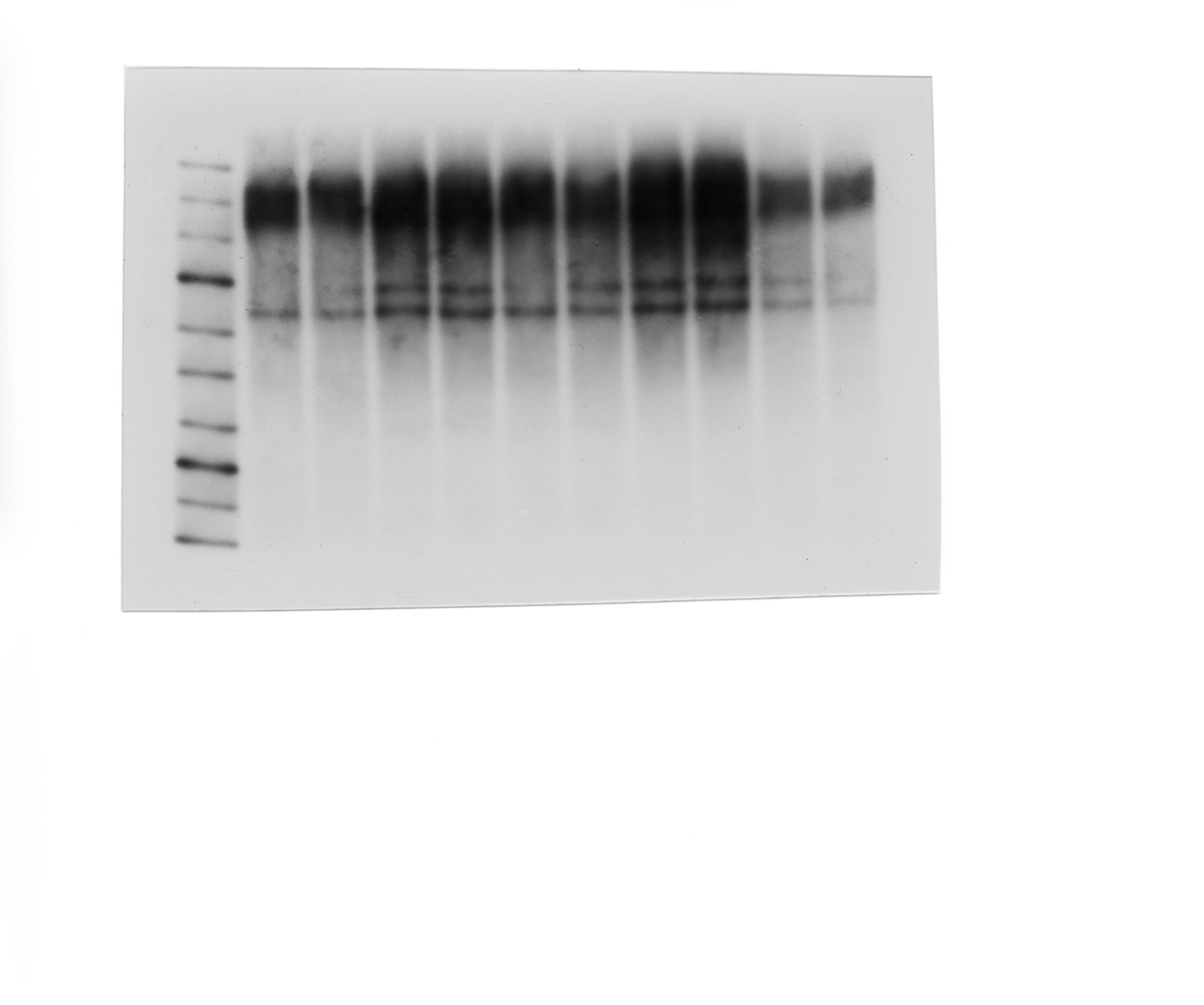

Supplement: Supplementary file 6 [file DataSheet4.zip › Fig7B Ip His.tif]

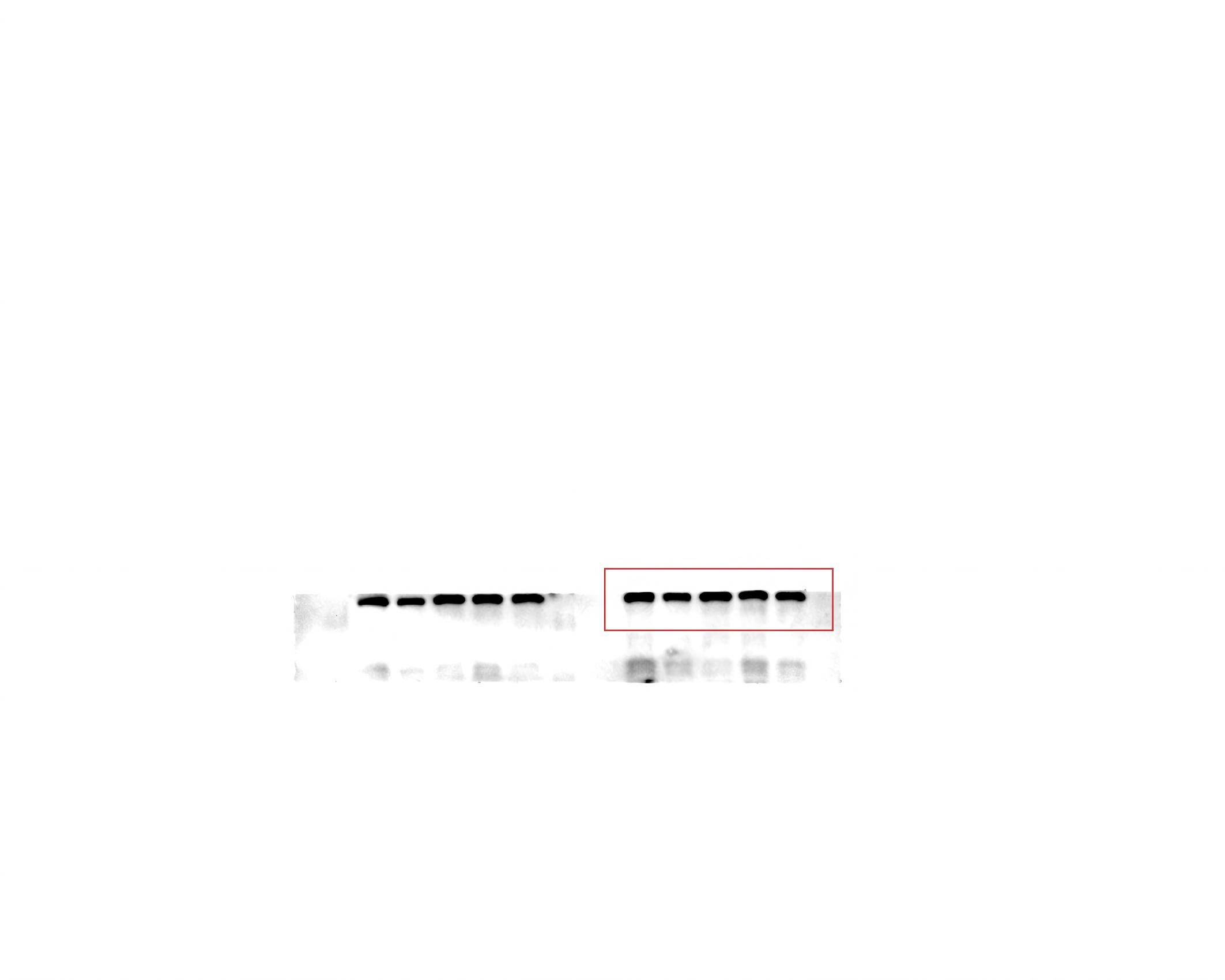

Supplement: Supplementary file 6 [file DataSheet4.zip › Fig7E Input Actin edited showing band.jpg]

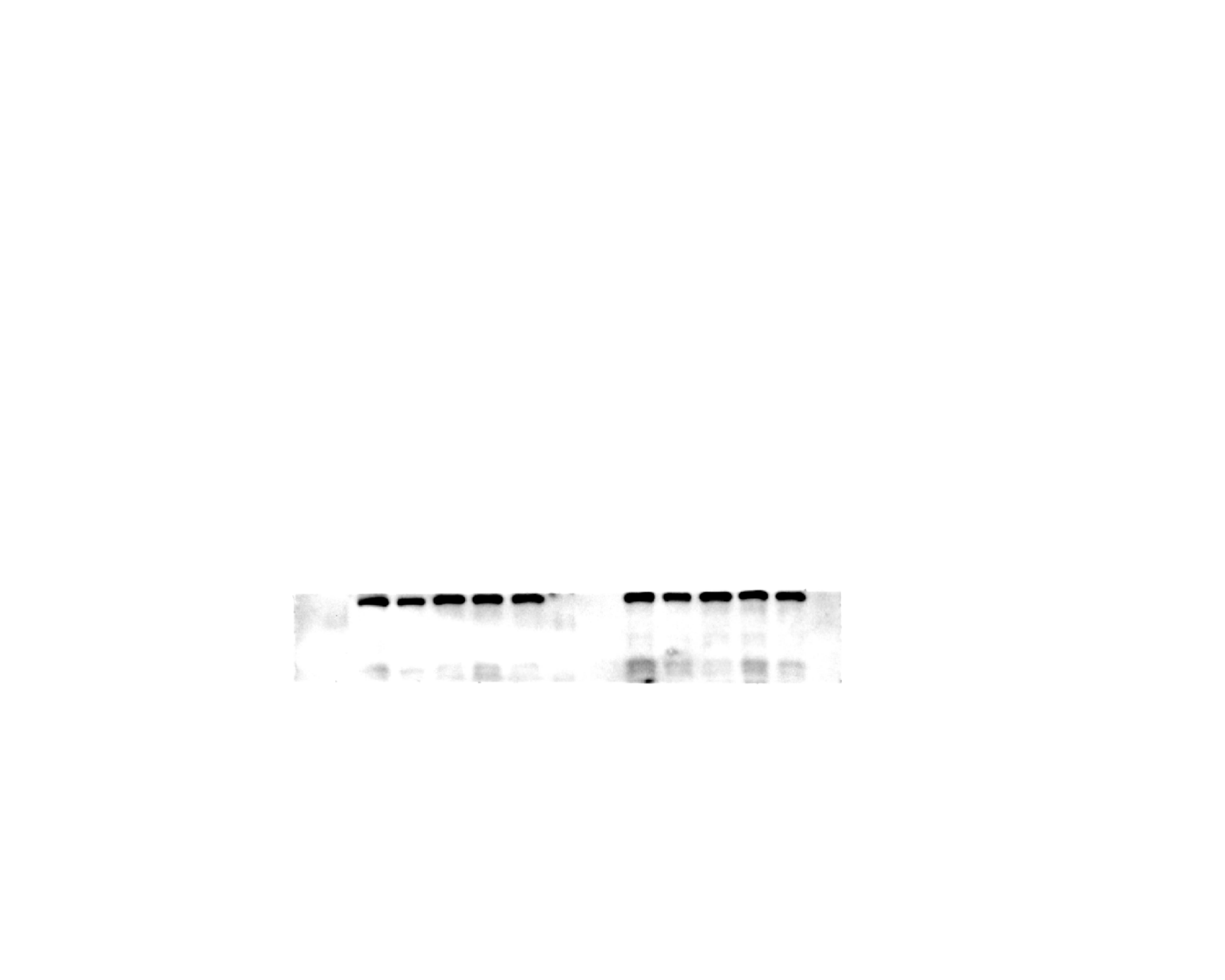

Supplement: Supplementary file 6 [file DataSheet4.zip › Fig7E Input Actin.png]

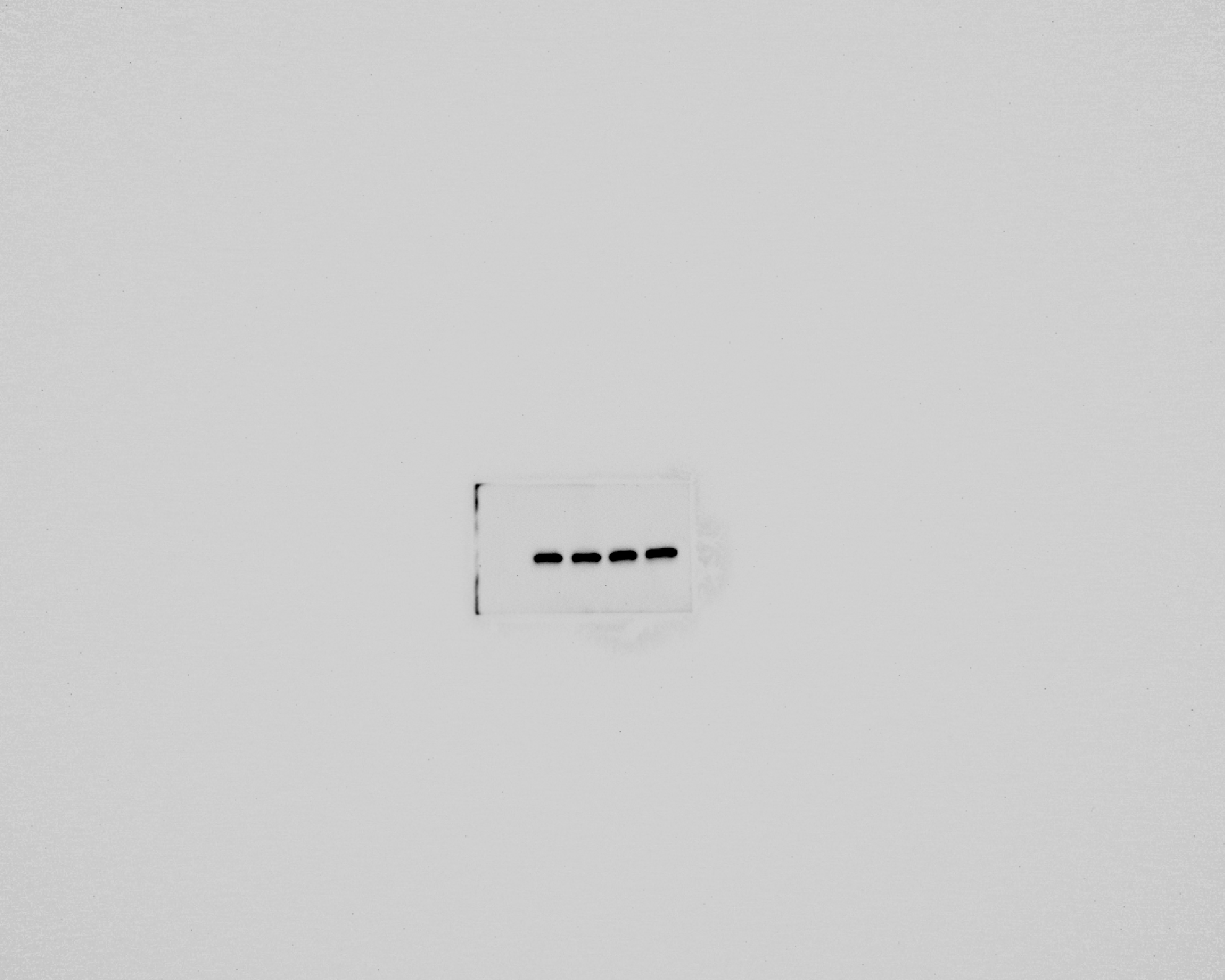

Supplement: Supplementary file 6 [file DataSheet4.zip › Fig7E Input HA.tif]

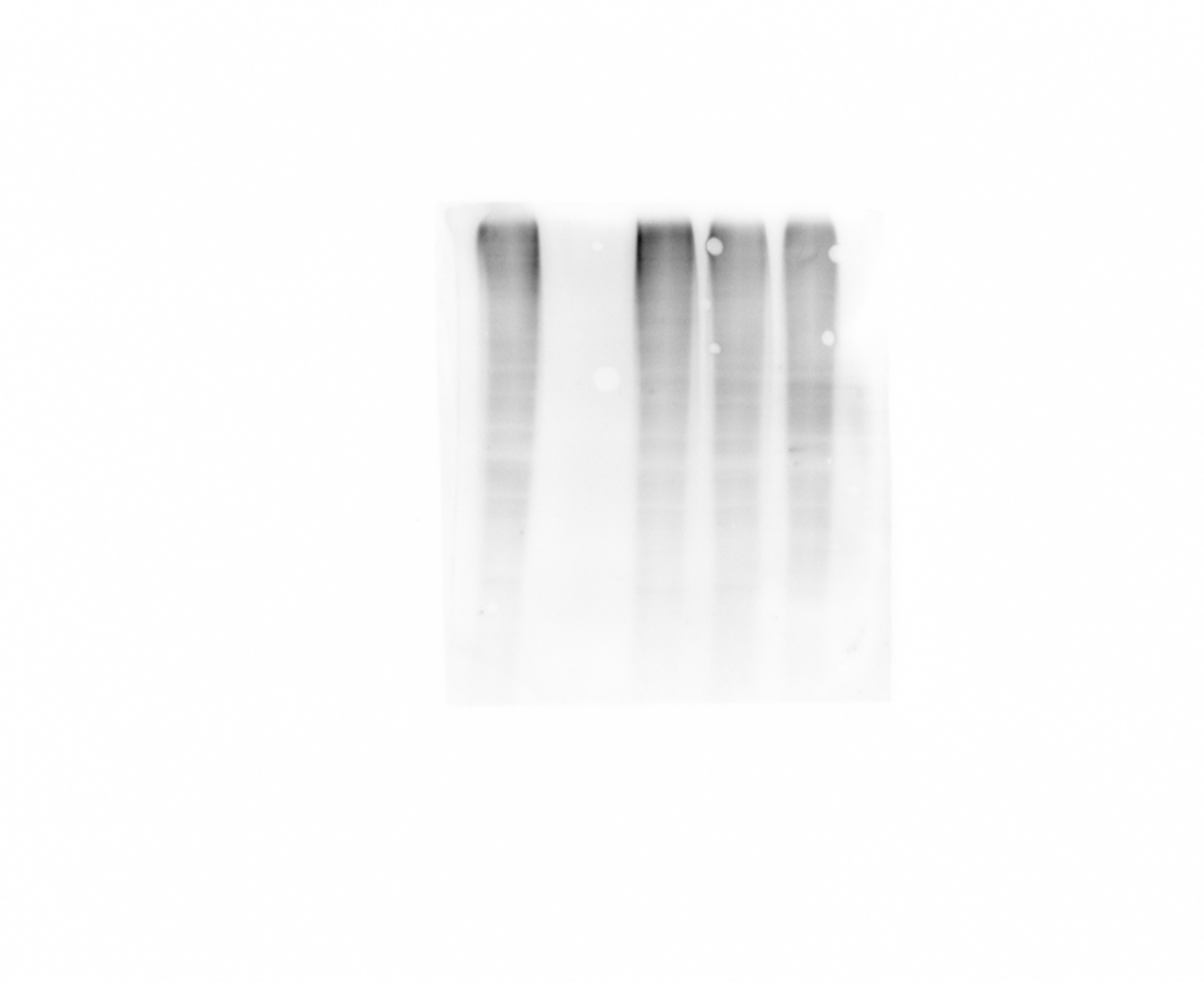

Supplement: Supplementary file 6 [file DataSheet4.zip › Fig7E Input His.jpg]

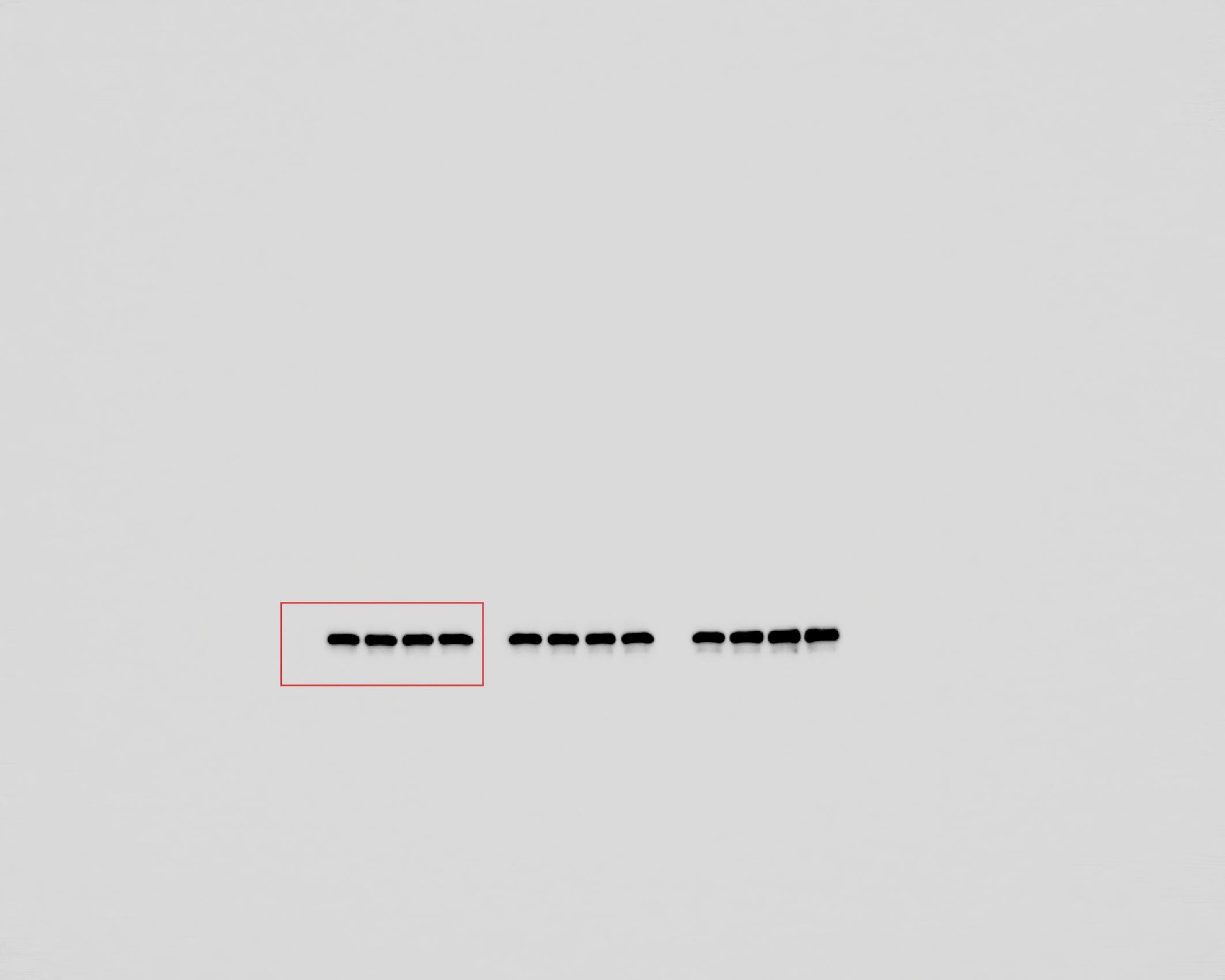

Supplement: Supplementary file 6 [file DataSheet4.zip › Fig7E IP HA edited showing band.jpg]

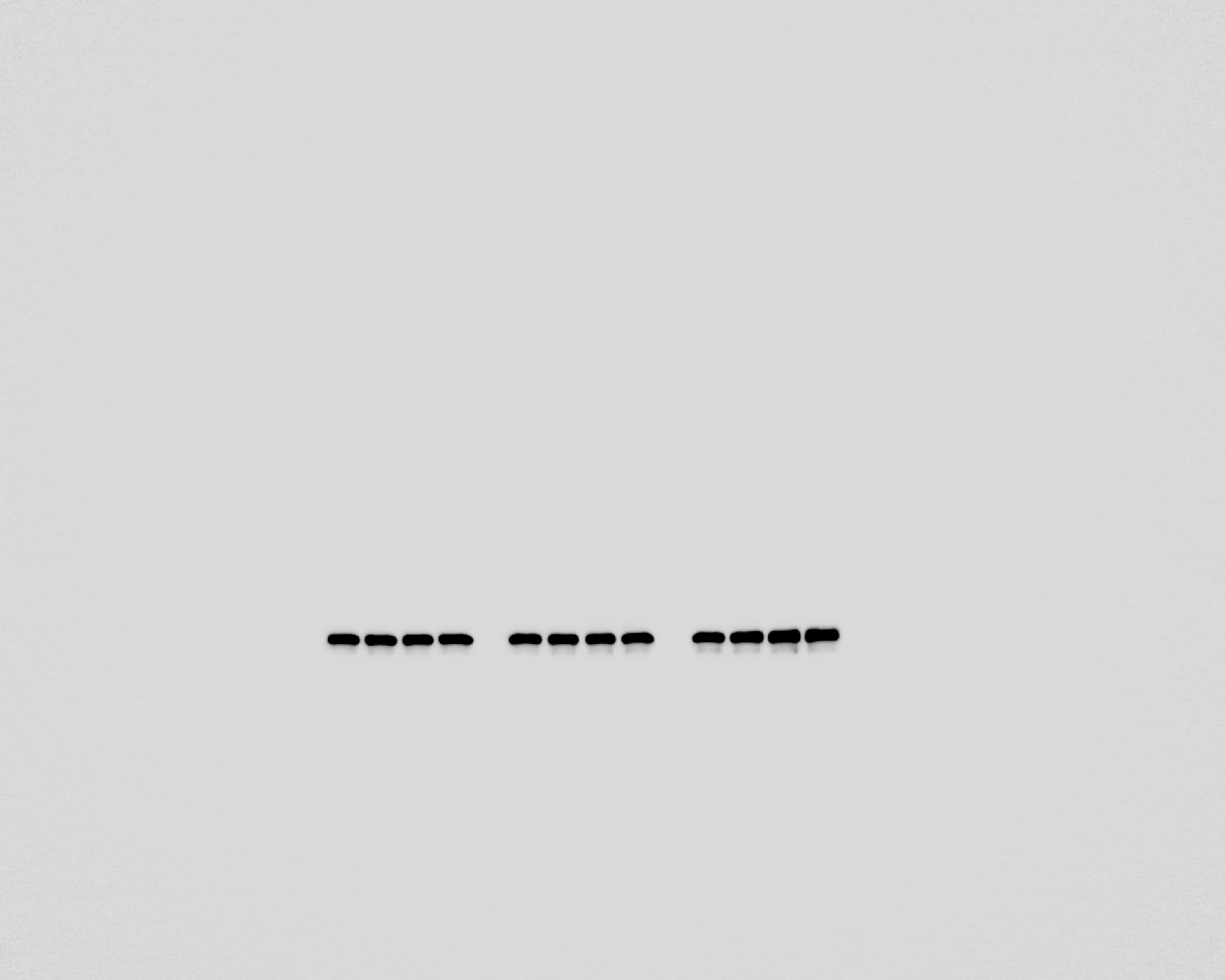

Supplement: Supplementary file 6 [file DataSheet4.zip › Fig7E IP HA.tif]

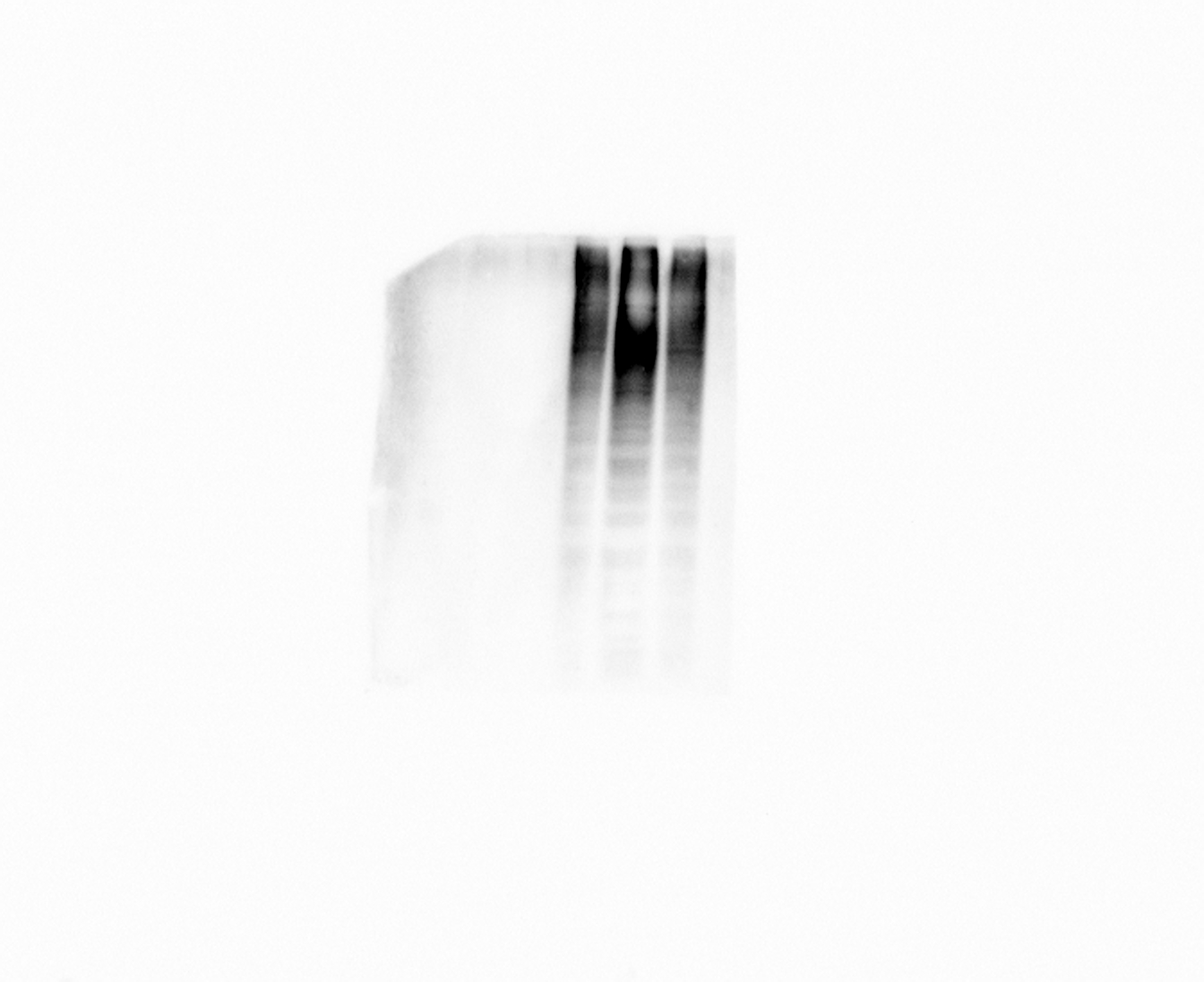

Supplement: Supplementary file 6 [file DataSheet4.zip › Fig7E IP His.jpg]

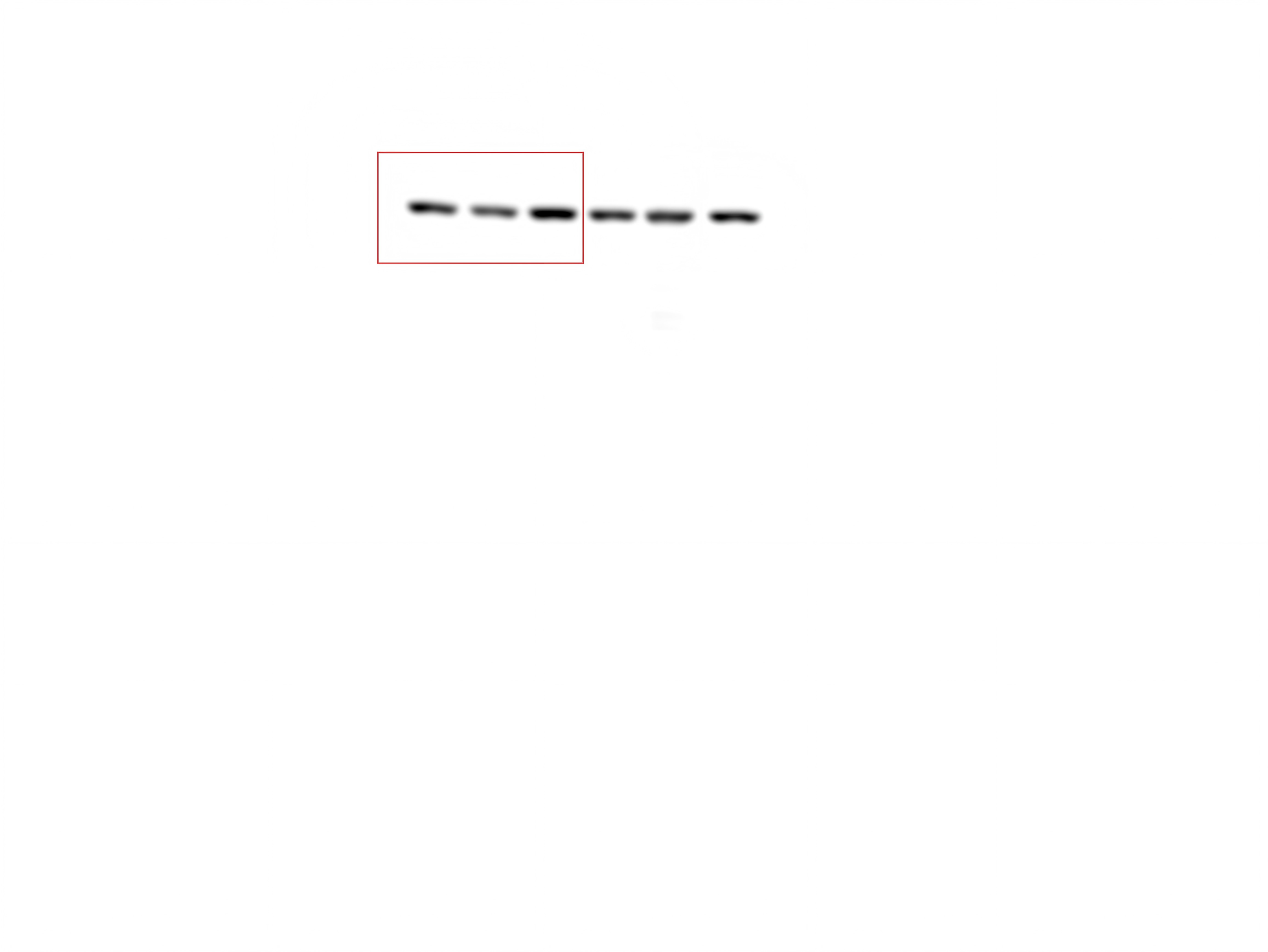

Supplement: Supplementary file 6 [file DataSheet4.zip › Fig8C Input Actin edited showing band.png]

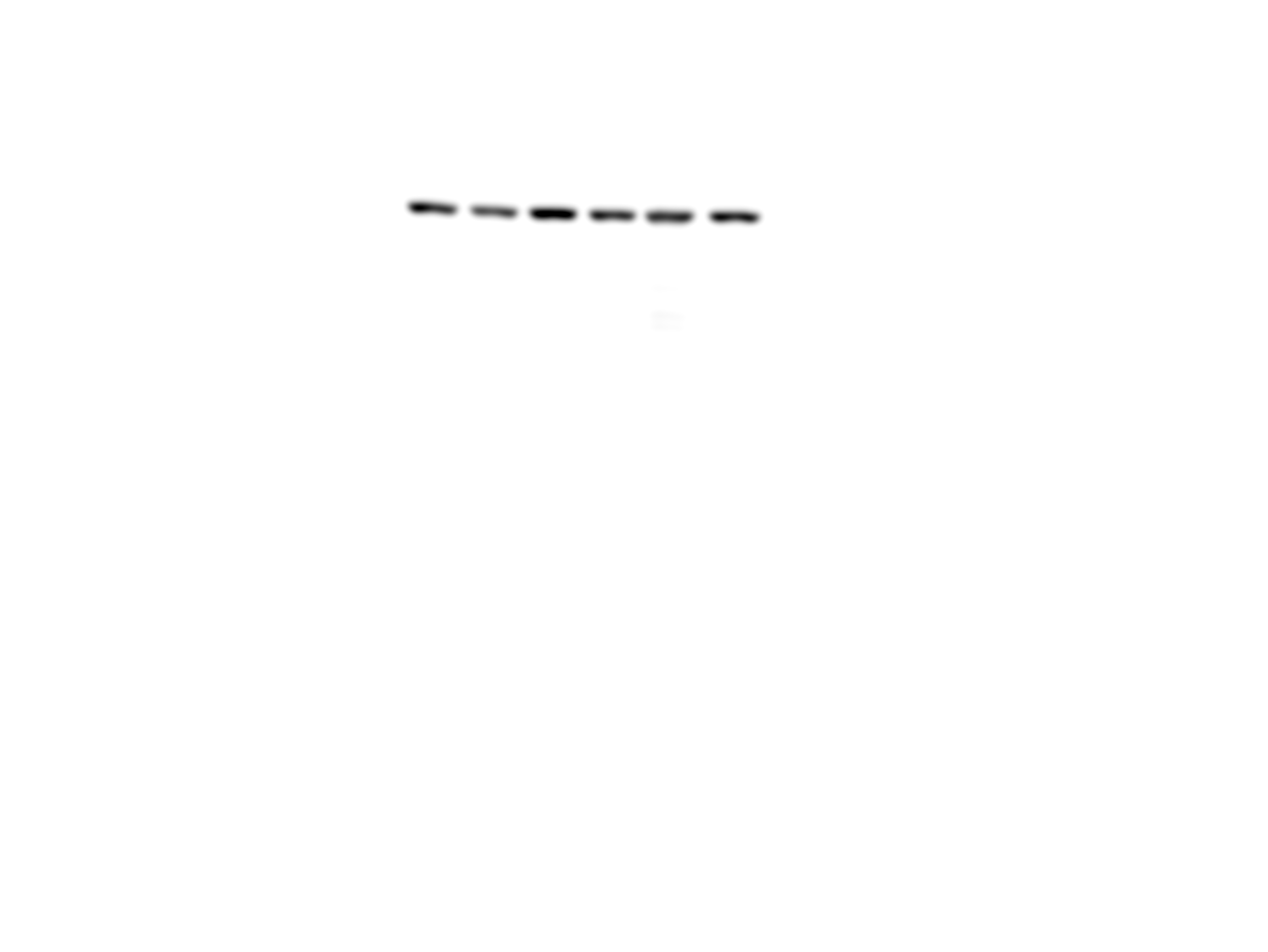

Supplement: Supplementary file 6 [file DataSheet4.zip › Fig8C Input Actin.tif]

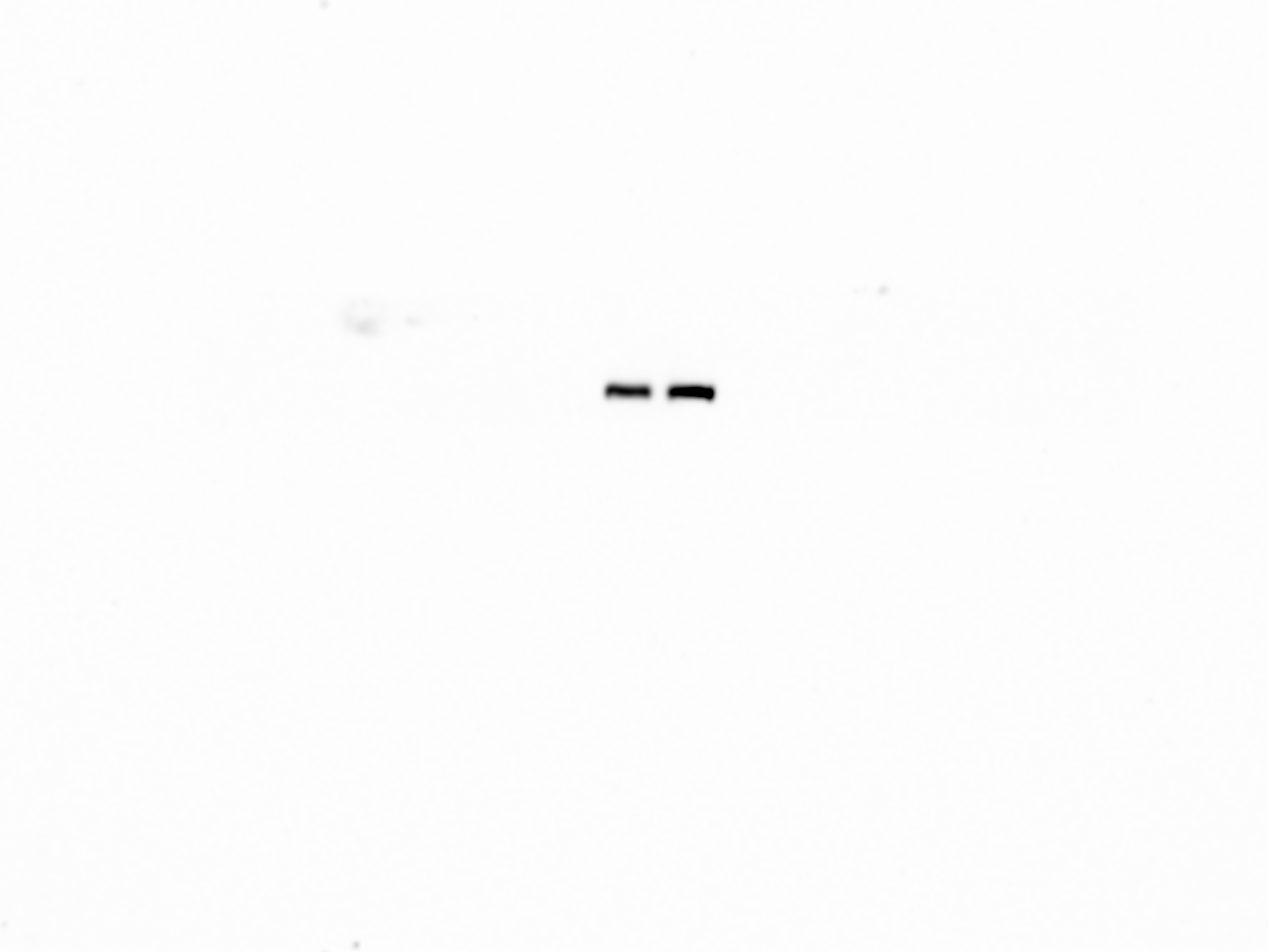

Supplement: Supplementary file 6 [file DataSheet4.zip › Fig8C Input Myc.tif]

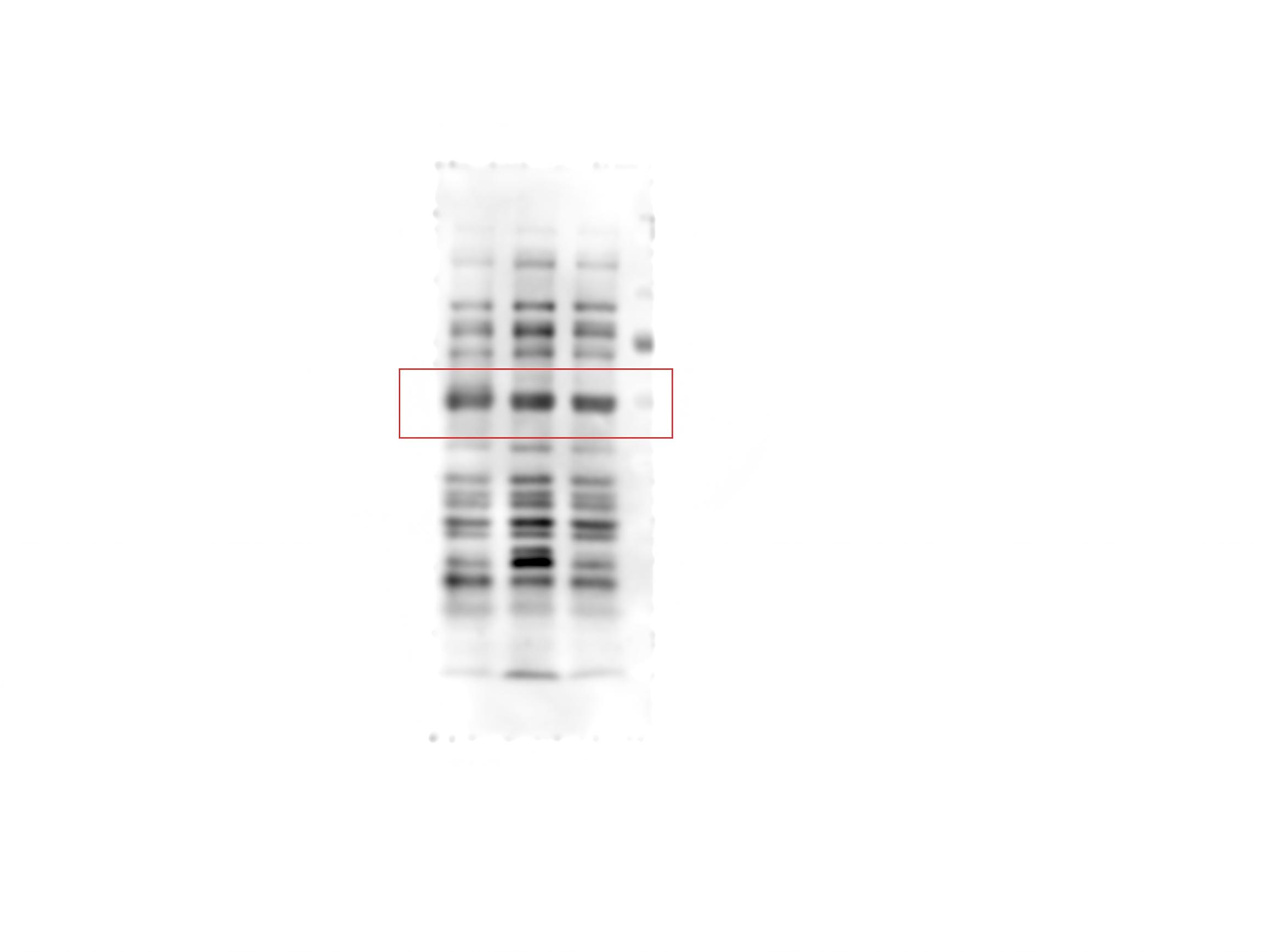

Supplement: Supplementary file 6 [file DataSheet4.zip › Fig8C Input-HA edited showing band.jpg]

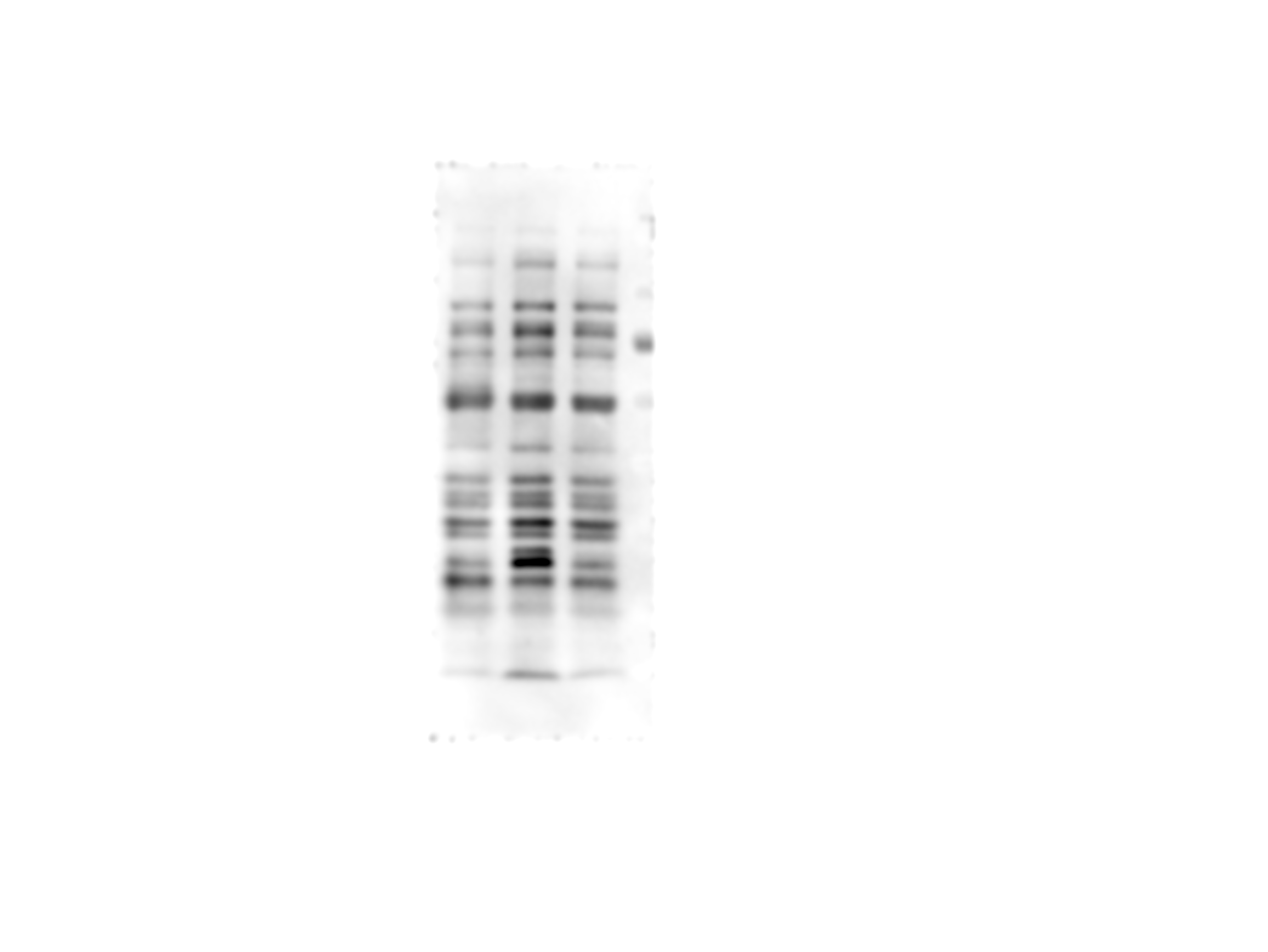

Supplement: Supplementary file 6 [file DataSheet4.zip › Fig8C Input-HA.tif]

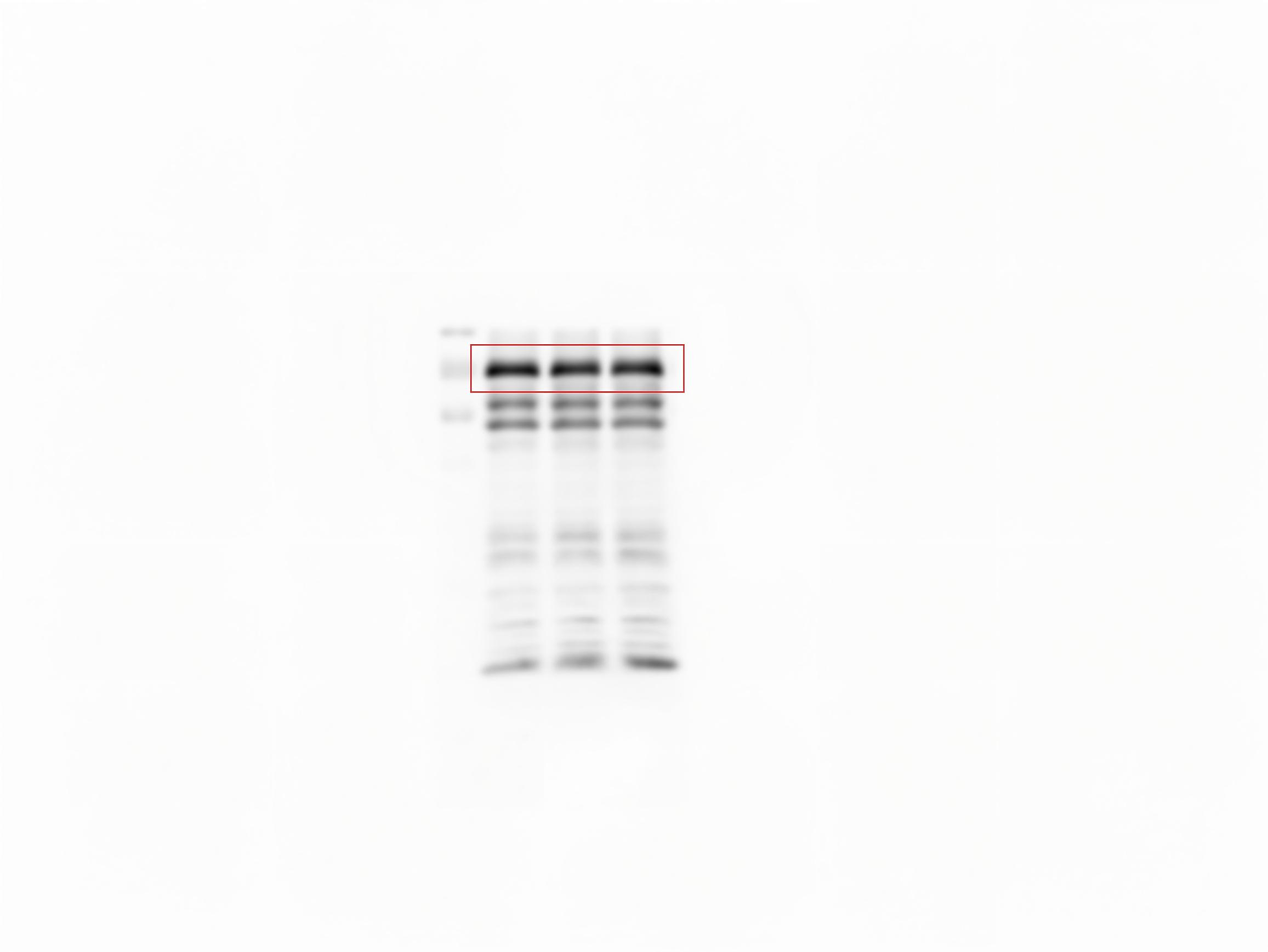

Supplement: Supplementary file 6 [file DataSheet4.zip › Fig8C IP-HA edited showing band.jpg]

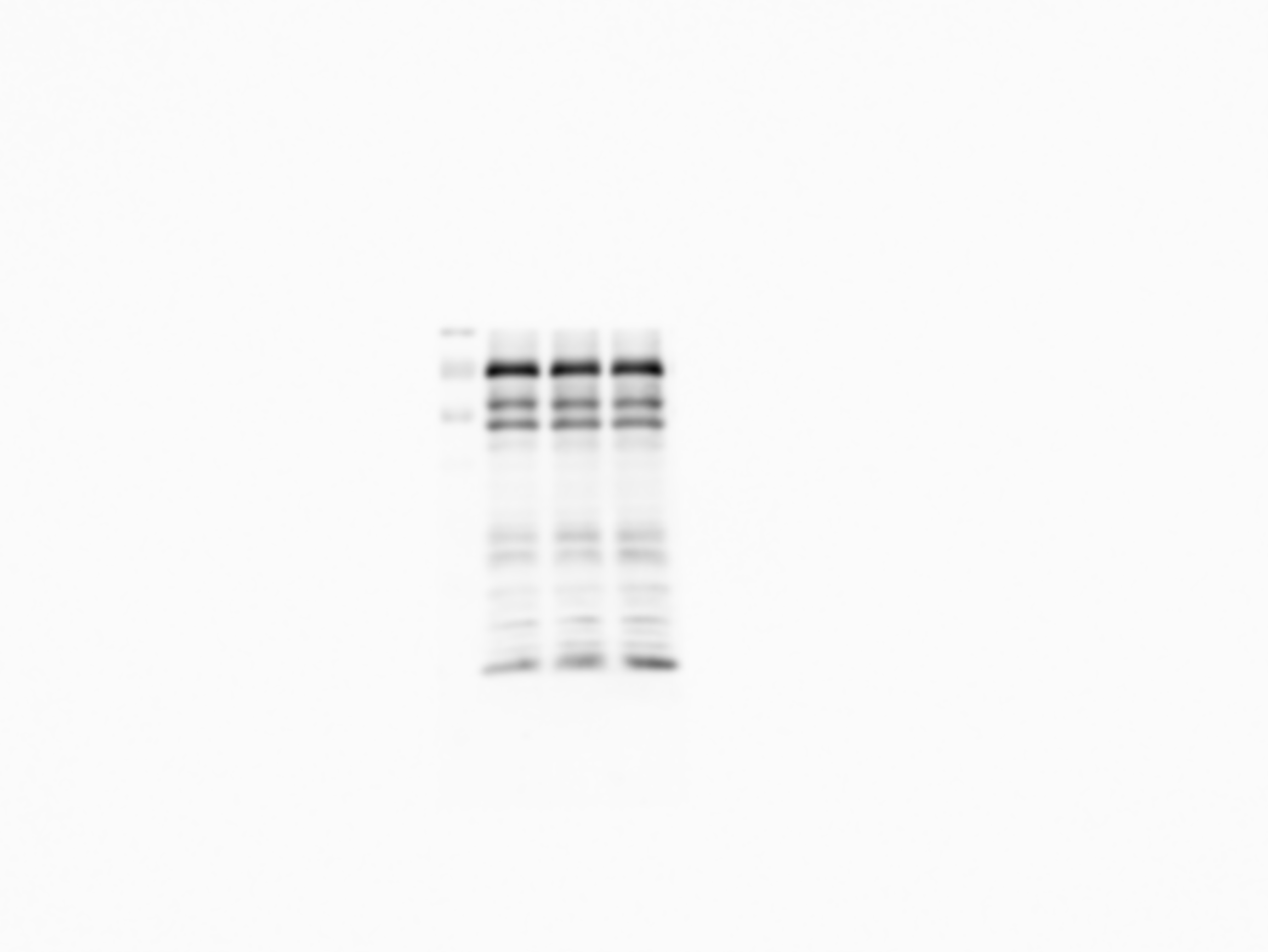

Supplement: Supplementary file 6 [file DataSheet4.zip › Fig8C IP-HA.tif]

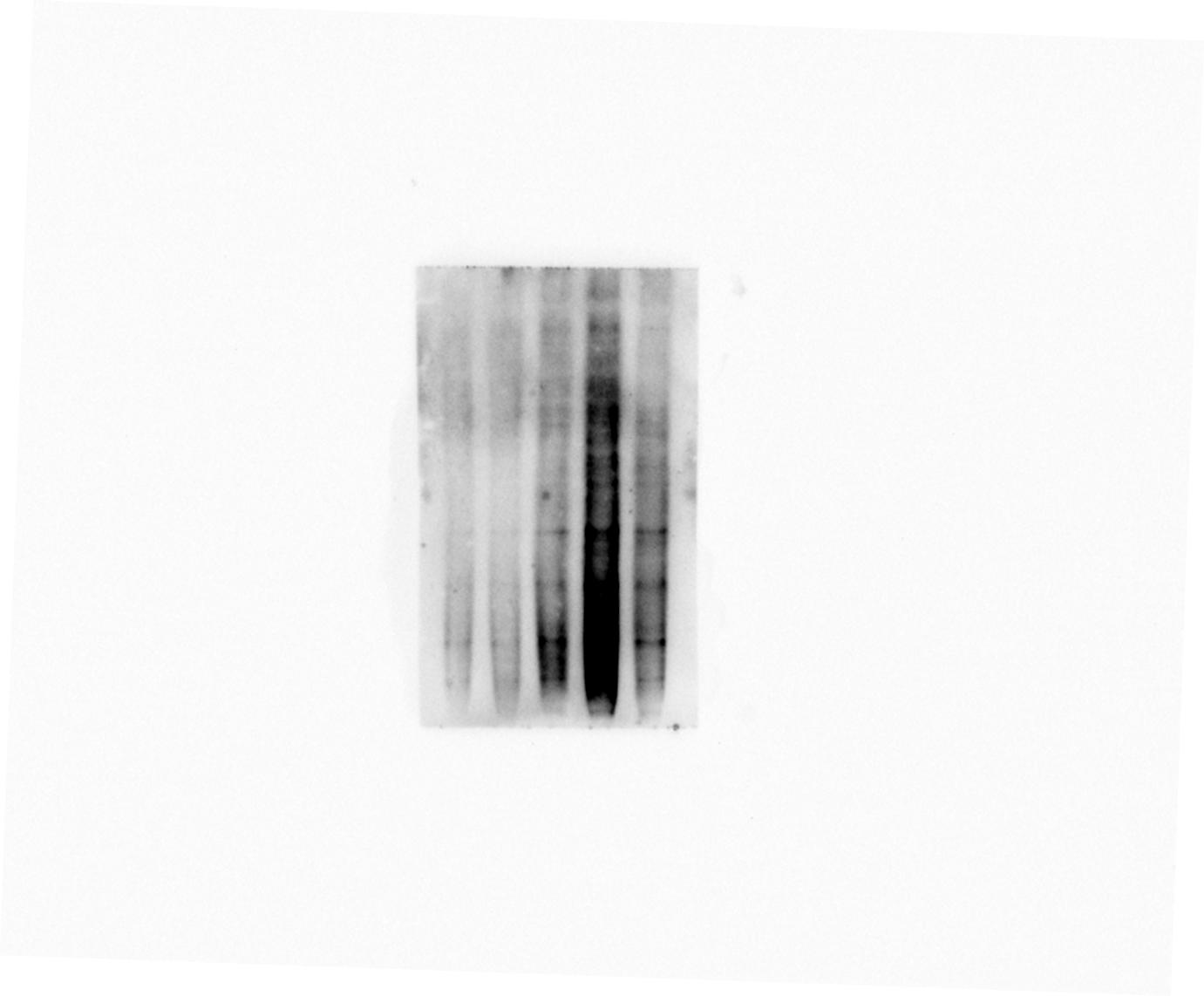

Supplement: Supplementary file 6 [file DataSheet4.zip › Fig8C IP-His.jpg]

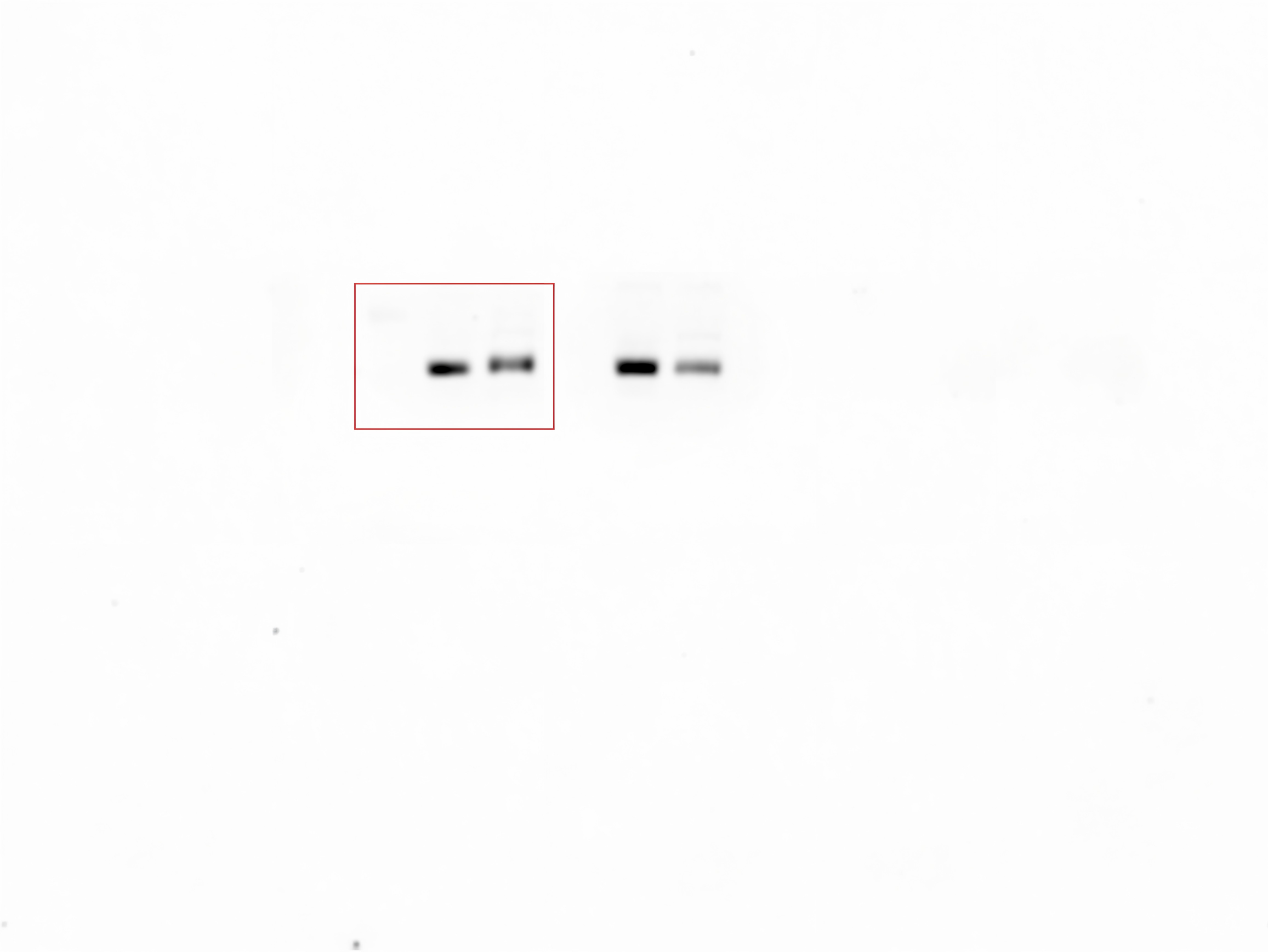

Supplement: Supplementary file 6 [file DataSheet4.zip › Fig8C IP-Myc edited showing band.png]

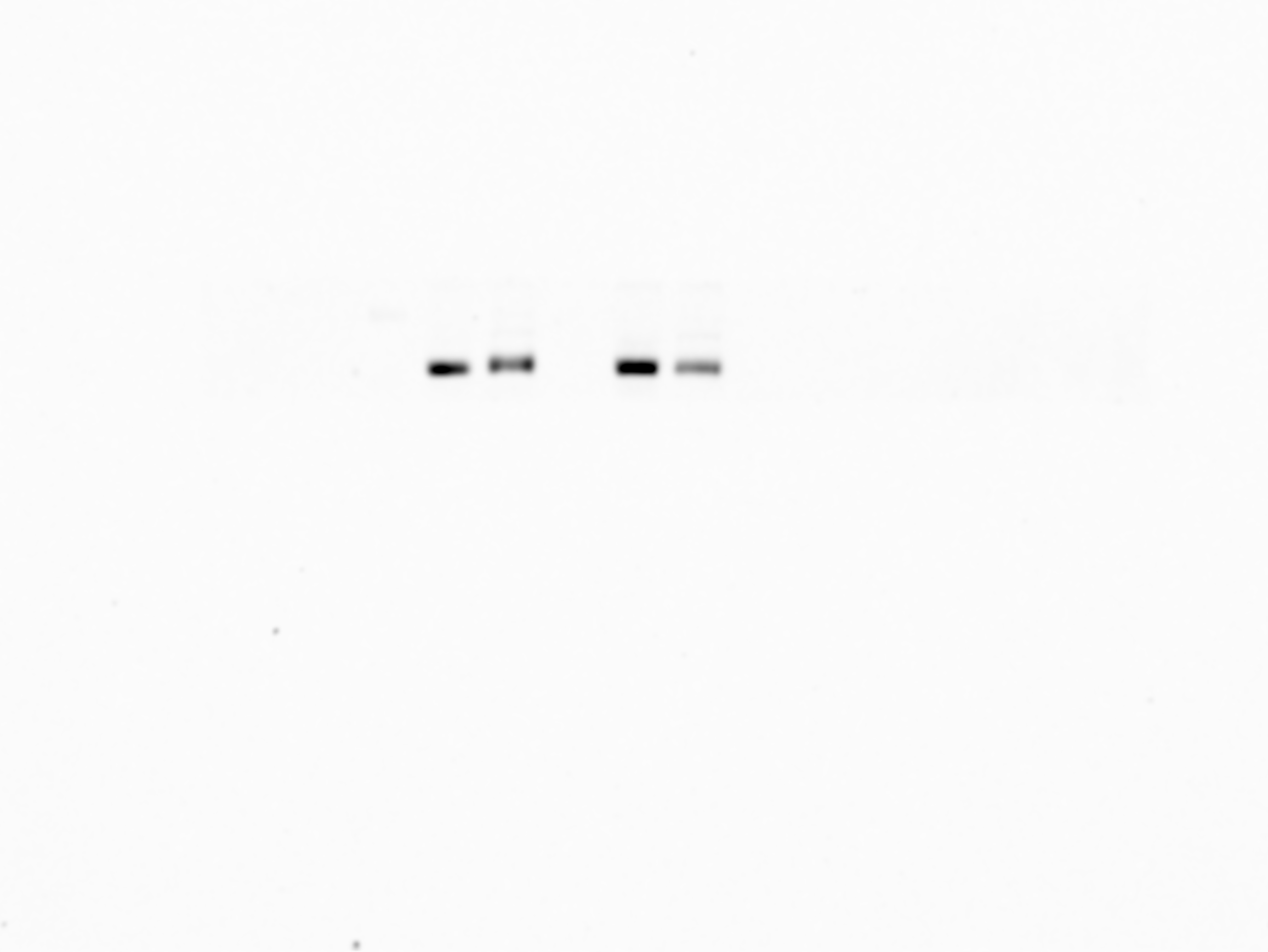

Supplement: Supplementary file 6 [file DataSheet4.zip › Fig8C IP-Myc.tif]

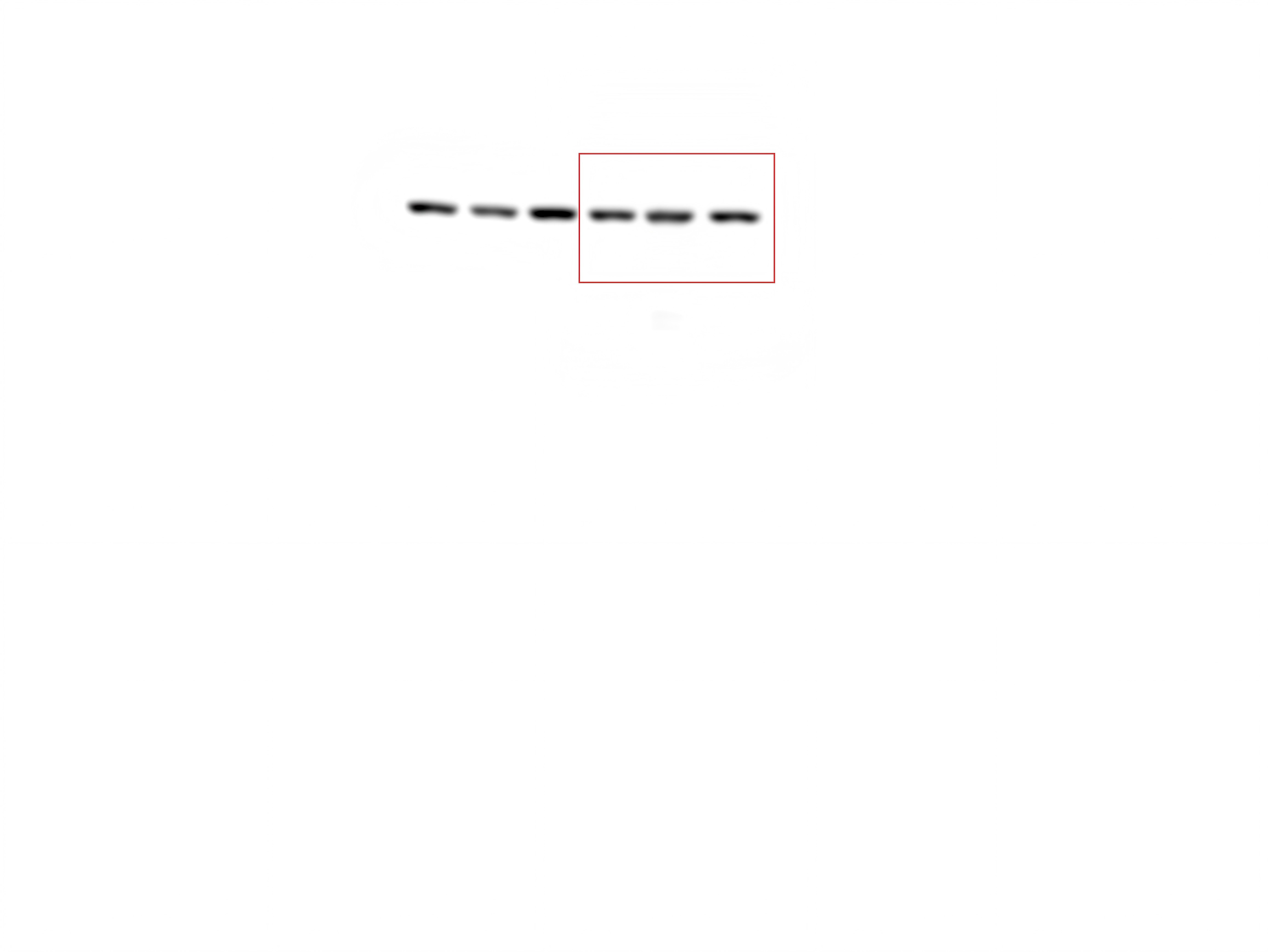

Supplement: Supplementary file 6 [file DataSheet4.zip › Fig8D Input Actin edited showing band.png]

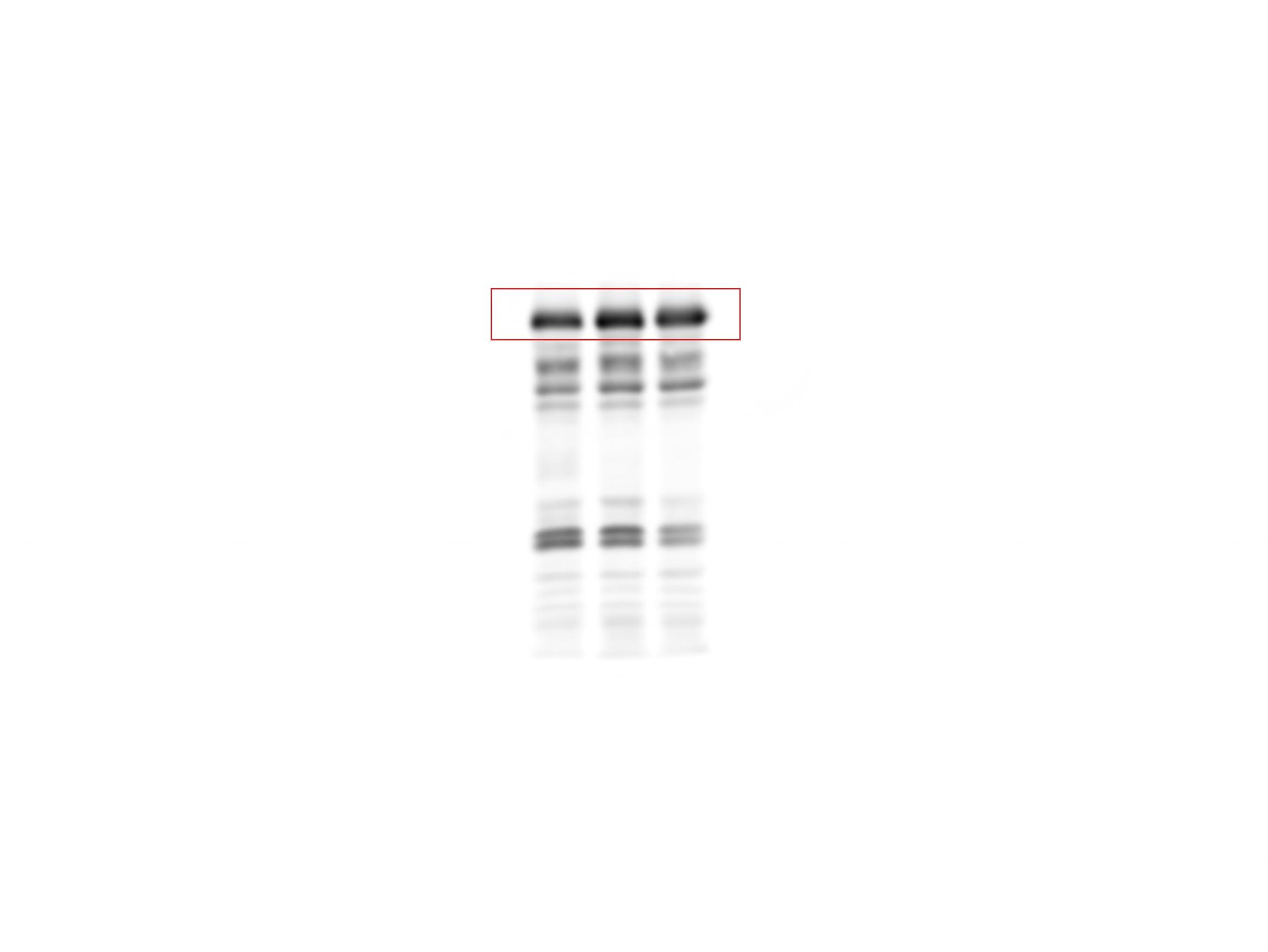

Supplement: Supplementary file 6 [file DataSheet4.zip › Fig8D Input HA edited showing band.jpg]

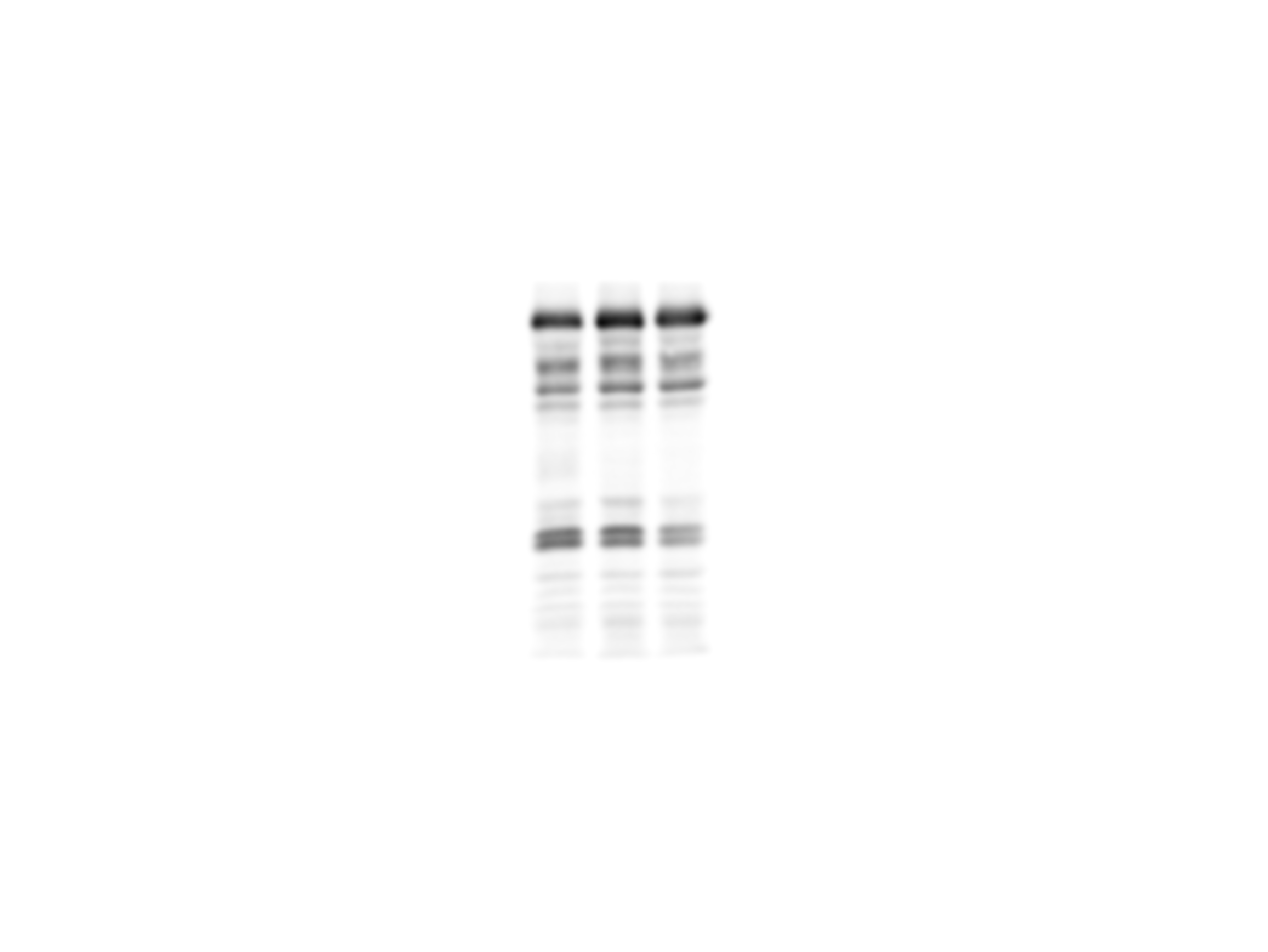

Supplement: Supplementary file 6 [file DataSheet4.zip › Fig8D Input HA.tif]

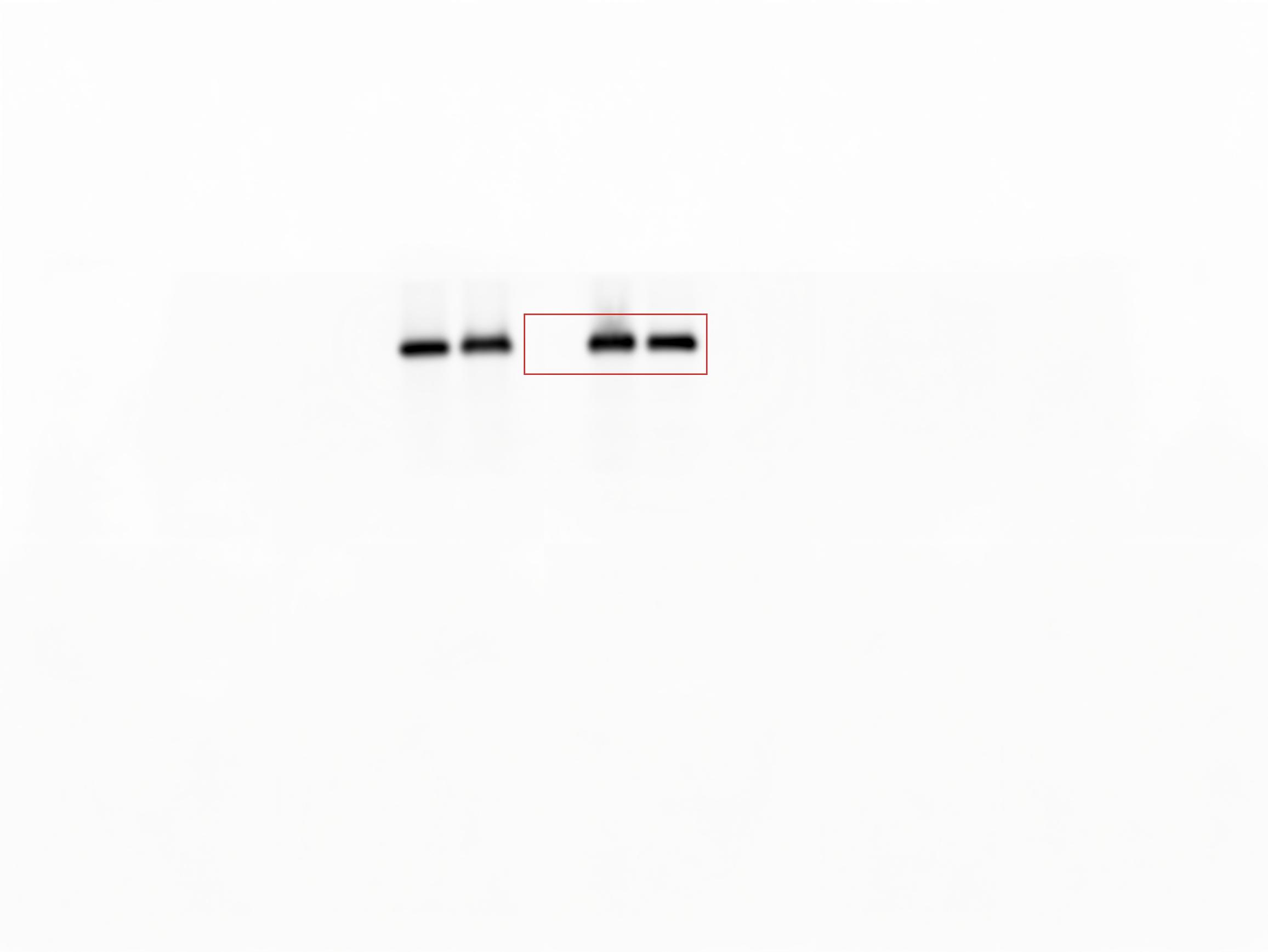

Supplement: Supplementary file 6 [file DataSheet4.zip › Fig8D Input Myc edited showing band.jpg]

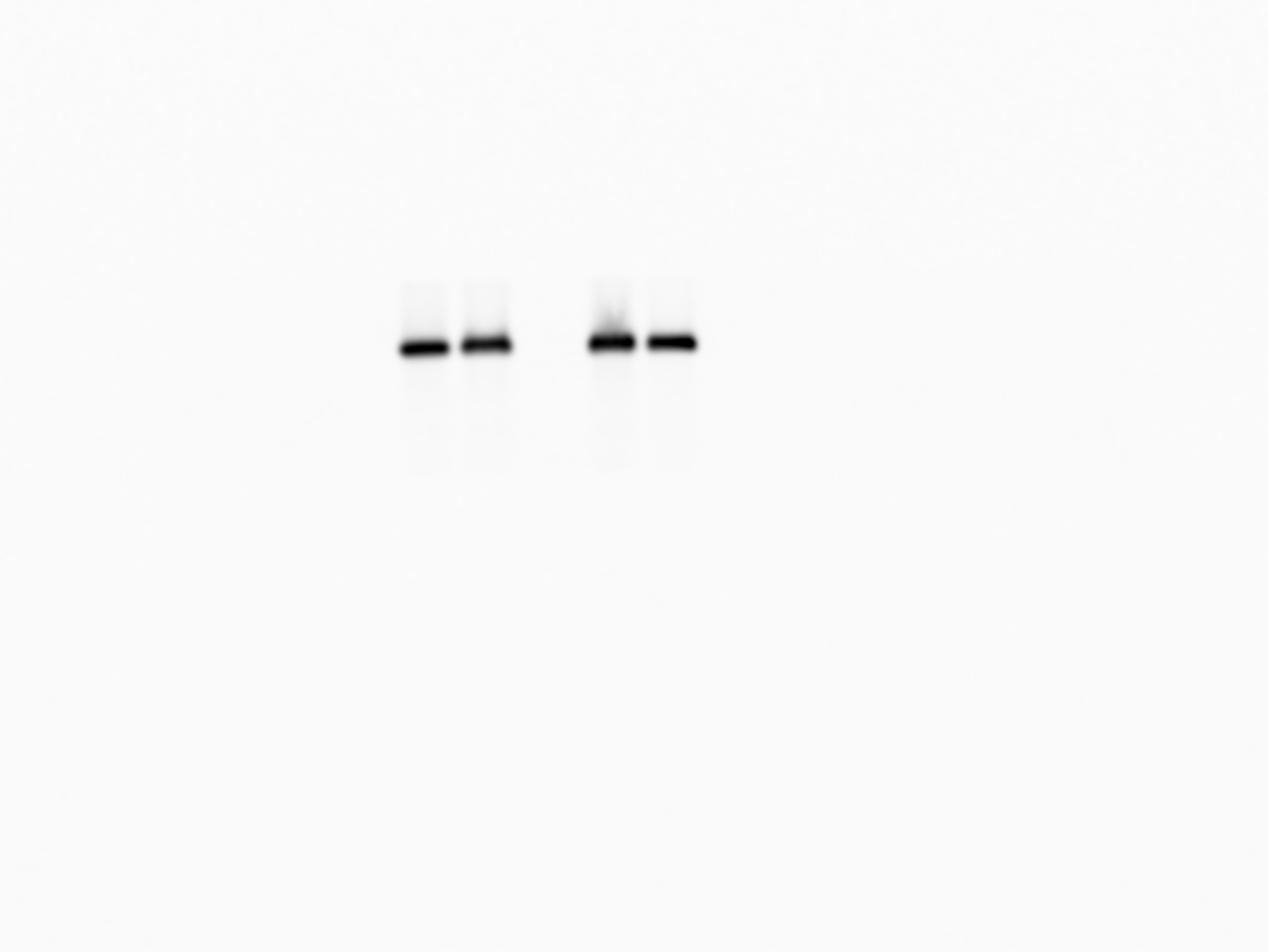

Supplement: Supplementary file 6 [file DataSheet4.zip › Fig8D Input Myc.tif]

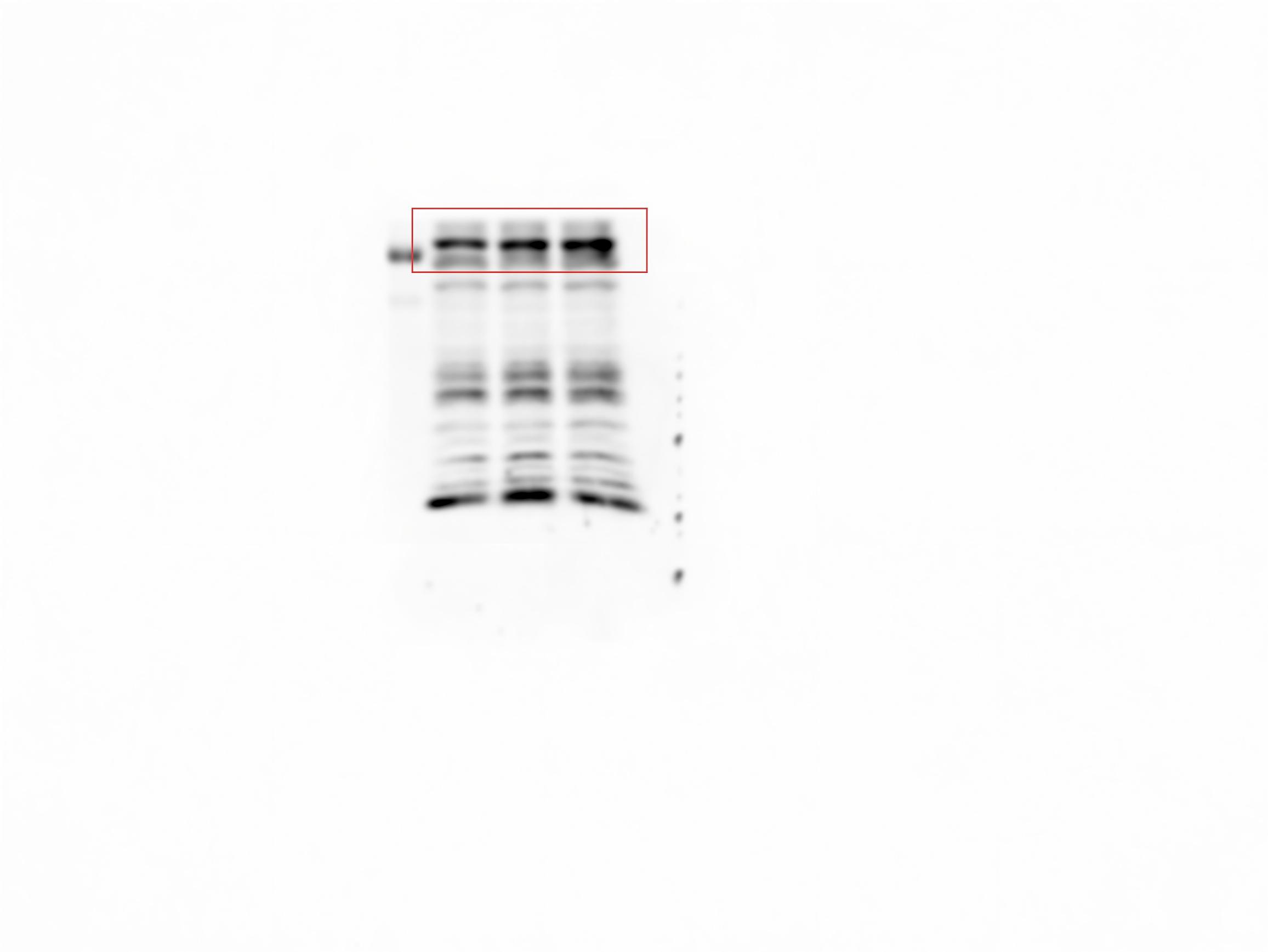

Supplement: Supplementary file 6 [file DataSheet4.zip › Fig8D IP HA edited showing band.jpg]

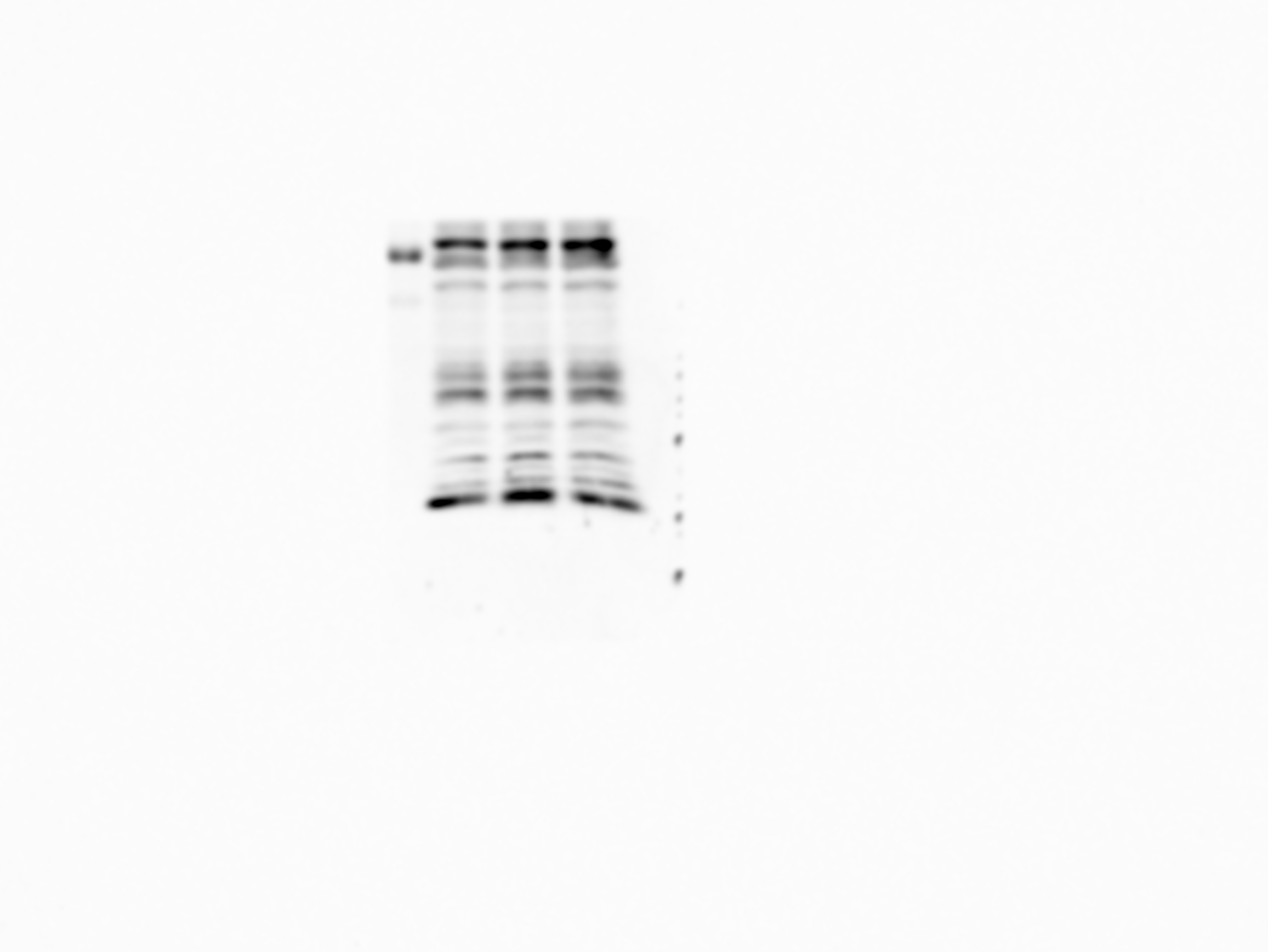

Supplement: Supplementary file 6 [file DataSheet4.zip › Fig8D IP HA.tif]

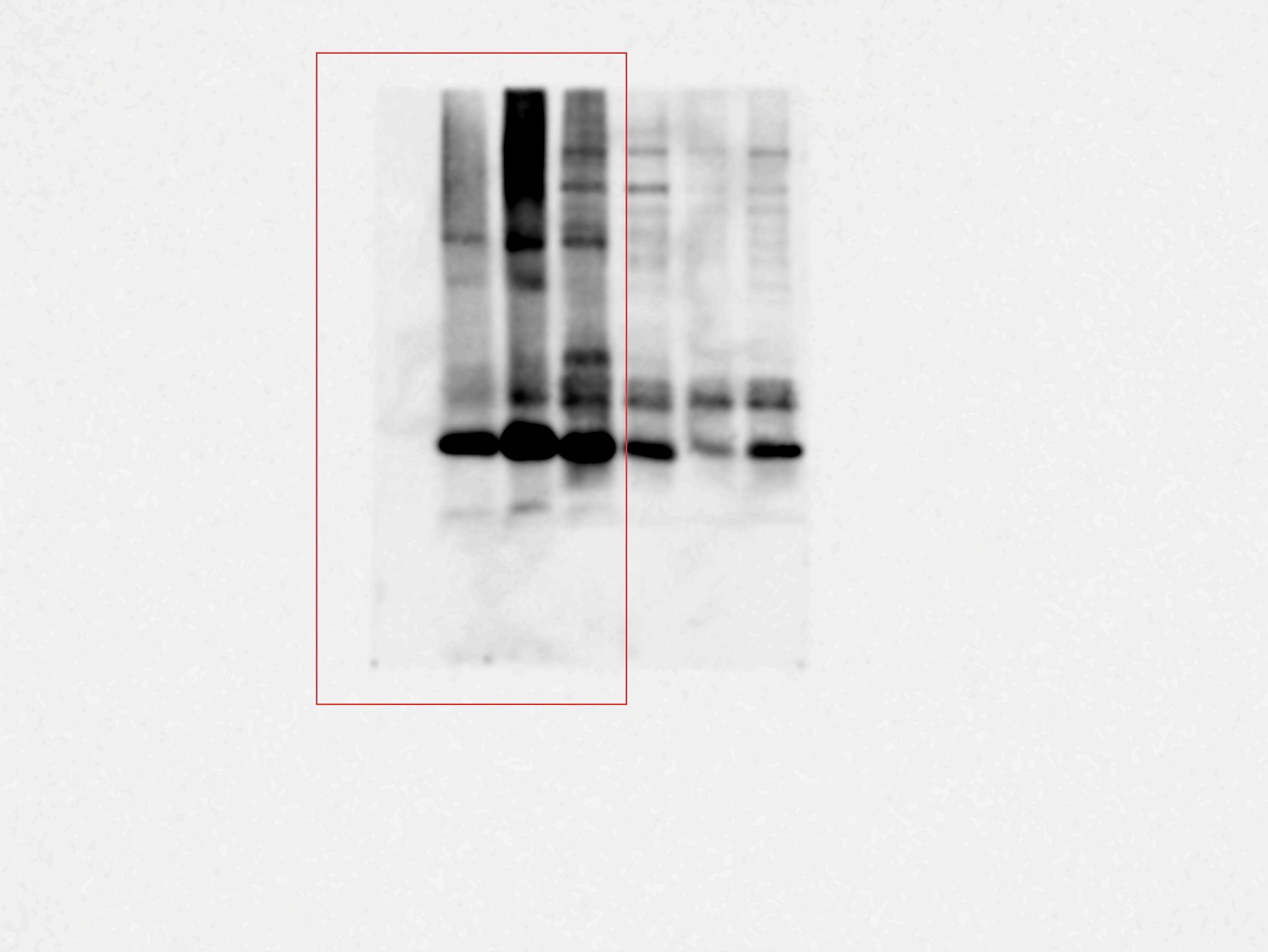

Supplement: Supplementary file 6 [file DataSheet4.zip › Fig8D IP His edited showing band.jpg]

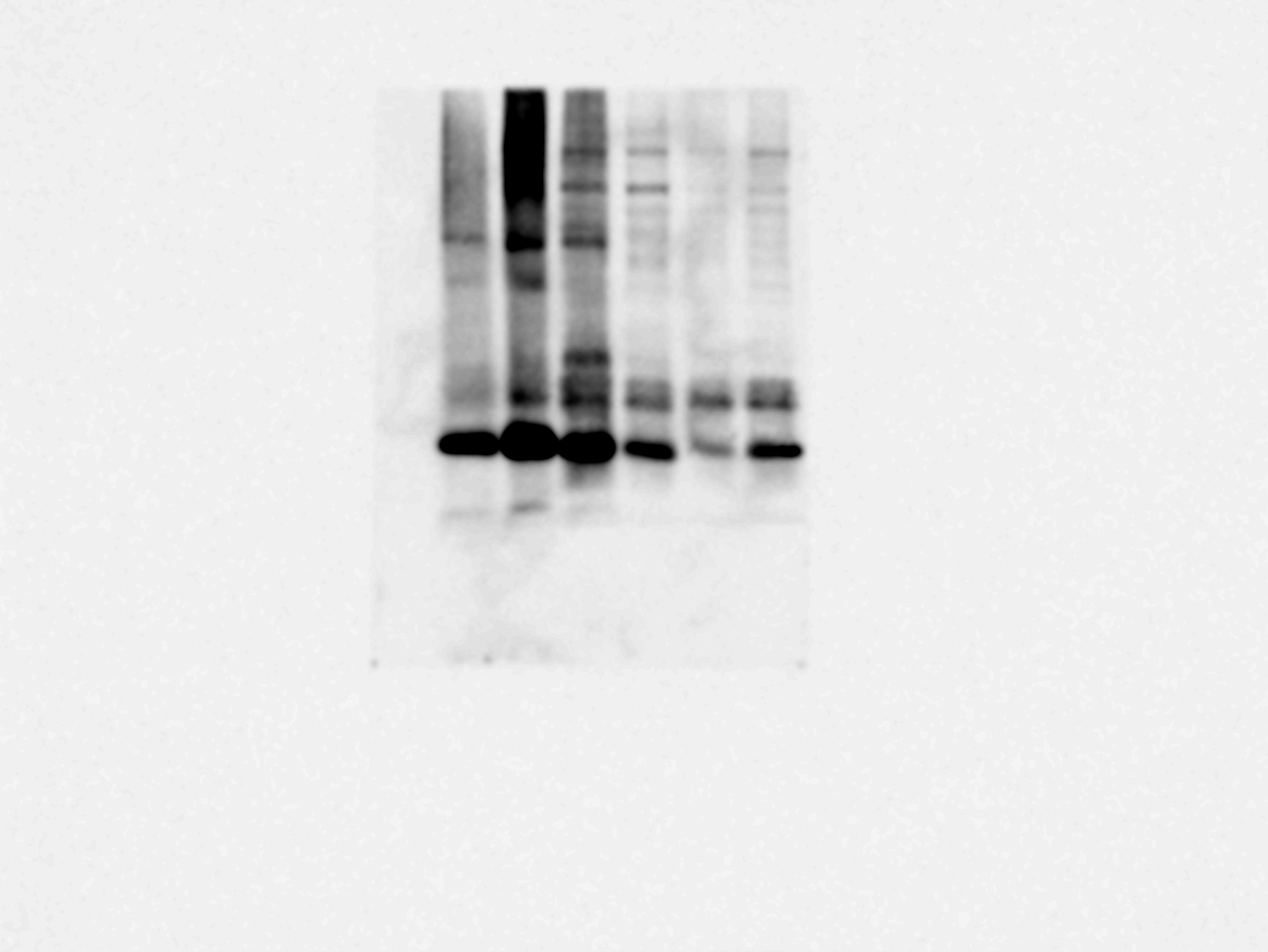

Supplement: Supplementary file 6 [file DataSheet4.zip › Fig8D IP His.tif]

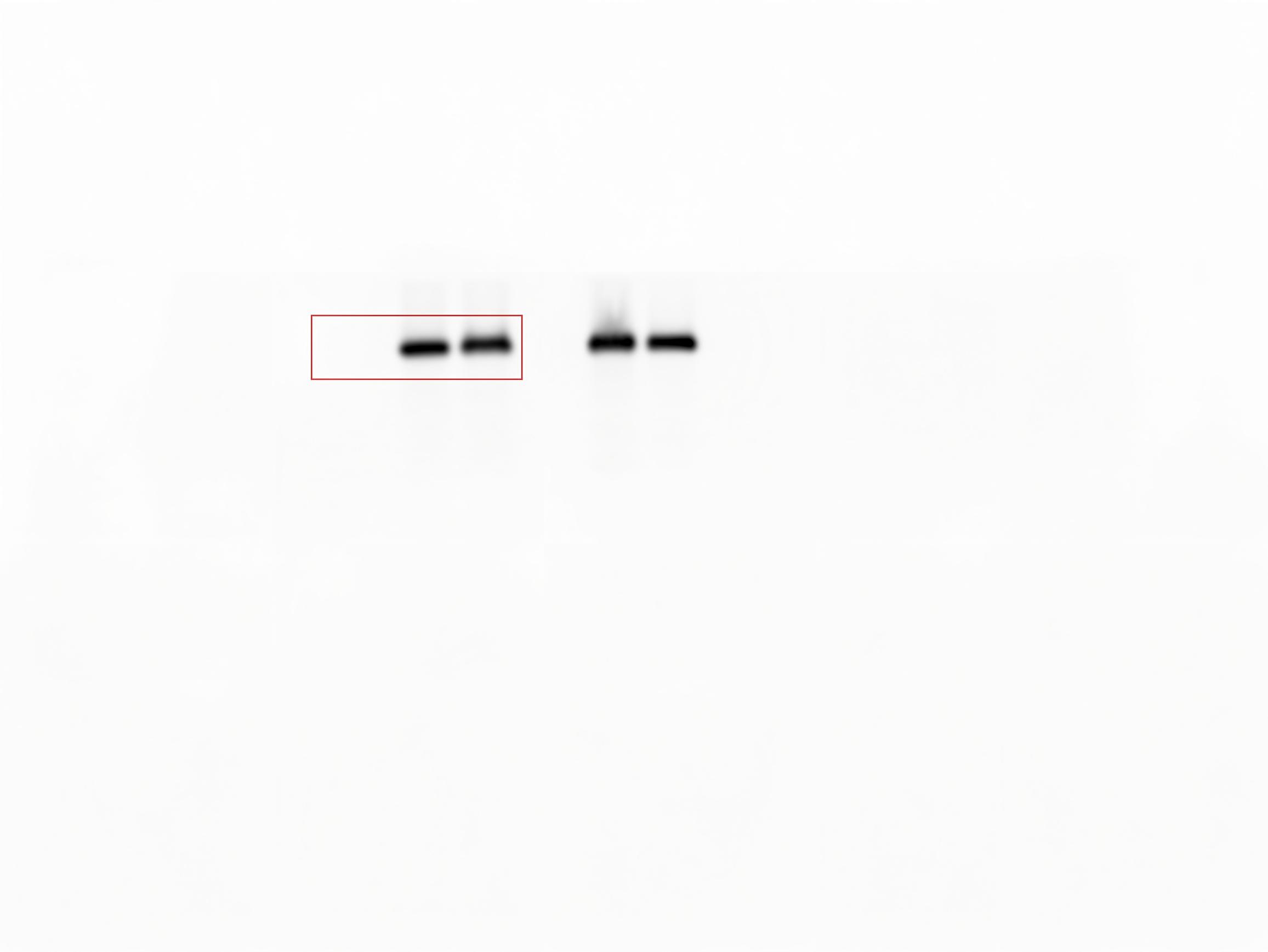

Supplement: Supplementary file 6 [file DataSheet4.zip › Fig8D IP Myc edited showing band.jpg]

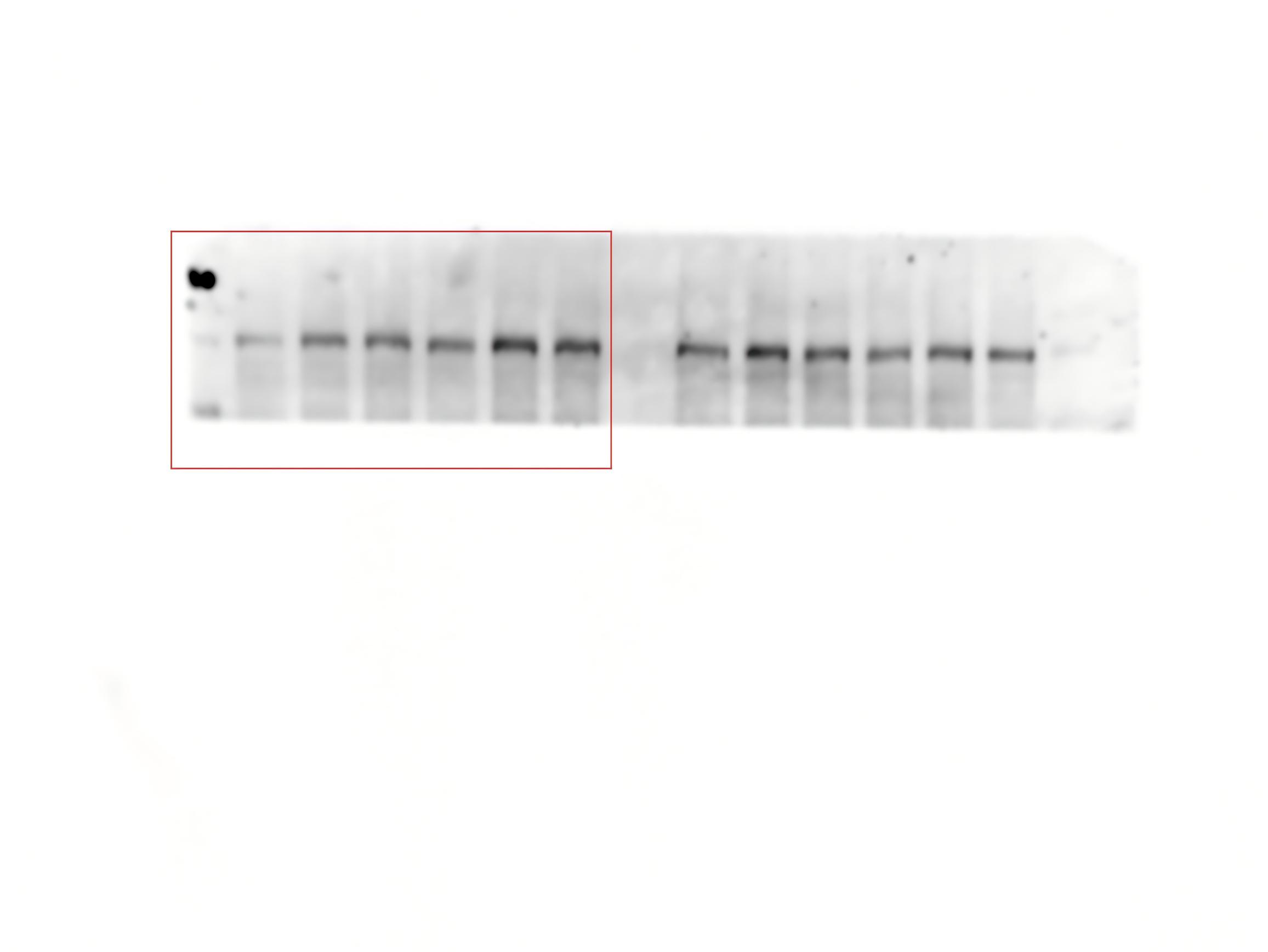

Supplement: Supplementary file 6 [file DataSheet4.zip › Supplementary Fig1 TRIM28.jpg]

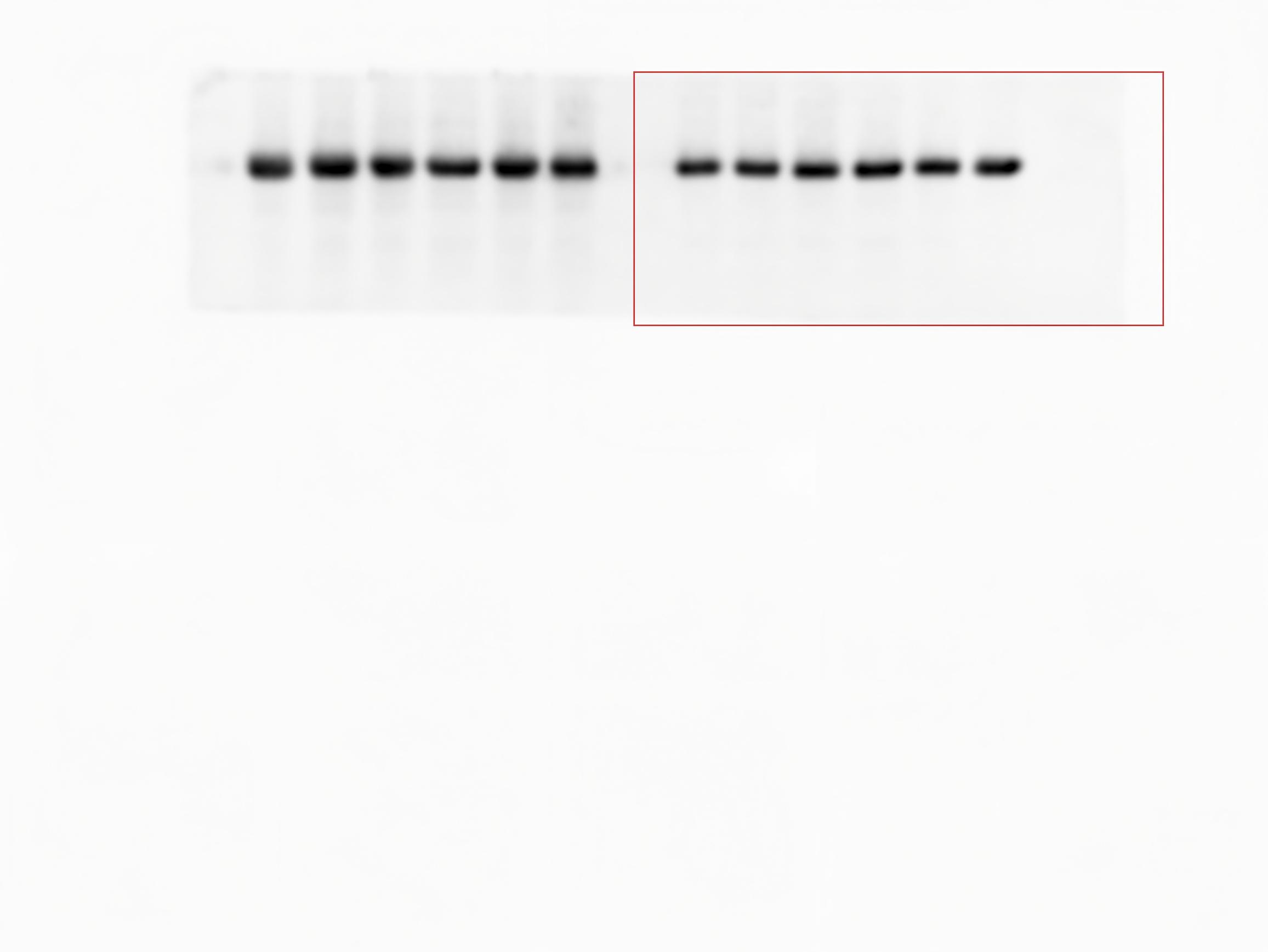

Supplement: Supplementary file 6 [file DataSheet4.zip › Supplementary Fig1D GAPDH.jpg]

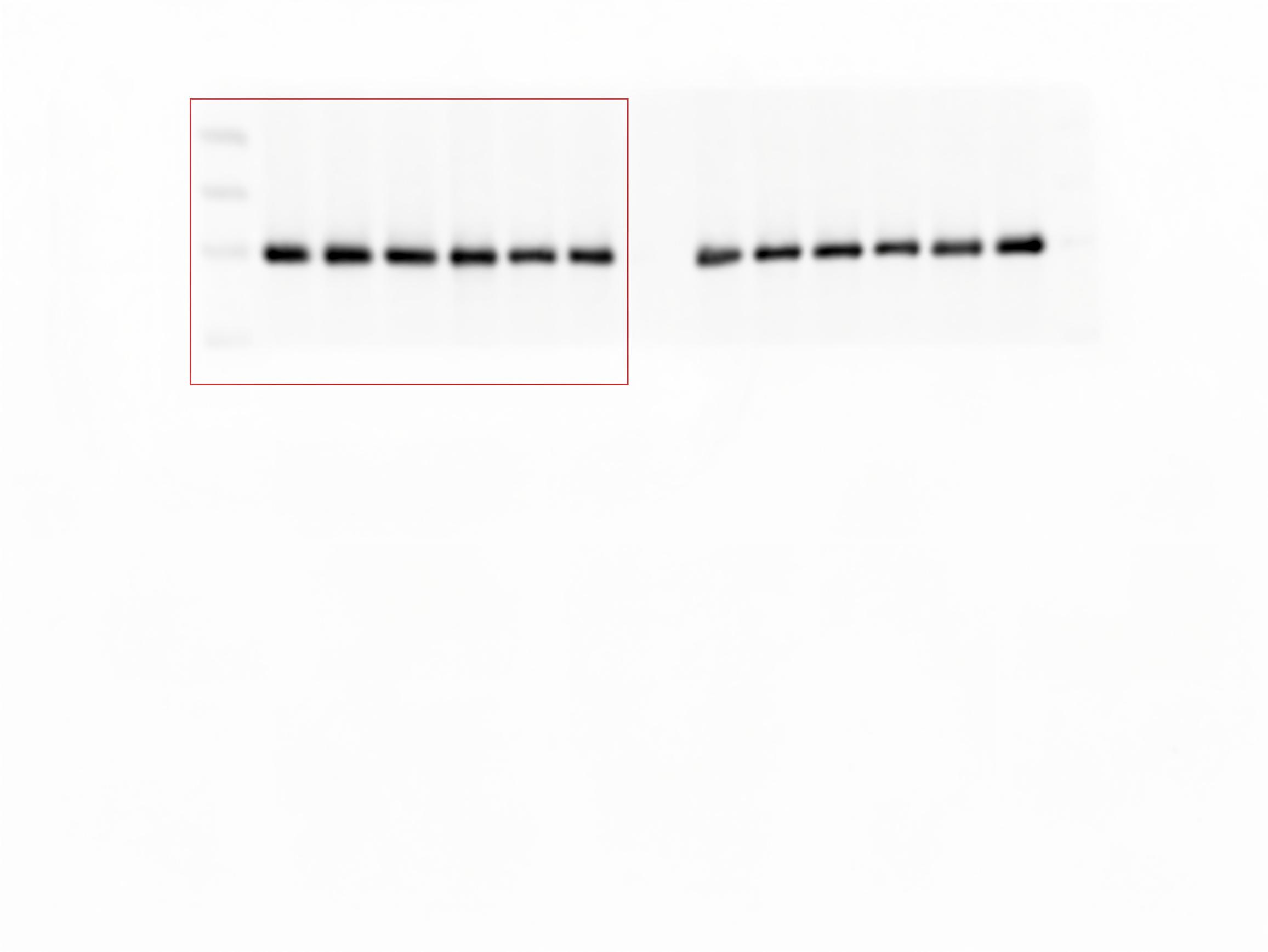

Supplement: Supplementary file 6 [file DataSheet4.zip › Supplementary Fig1F GAPDH.jpg]

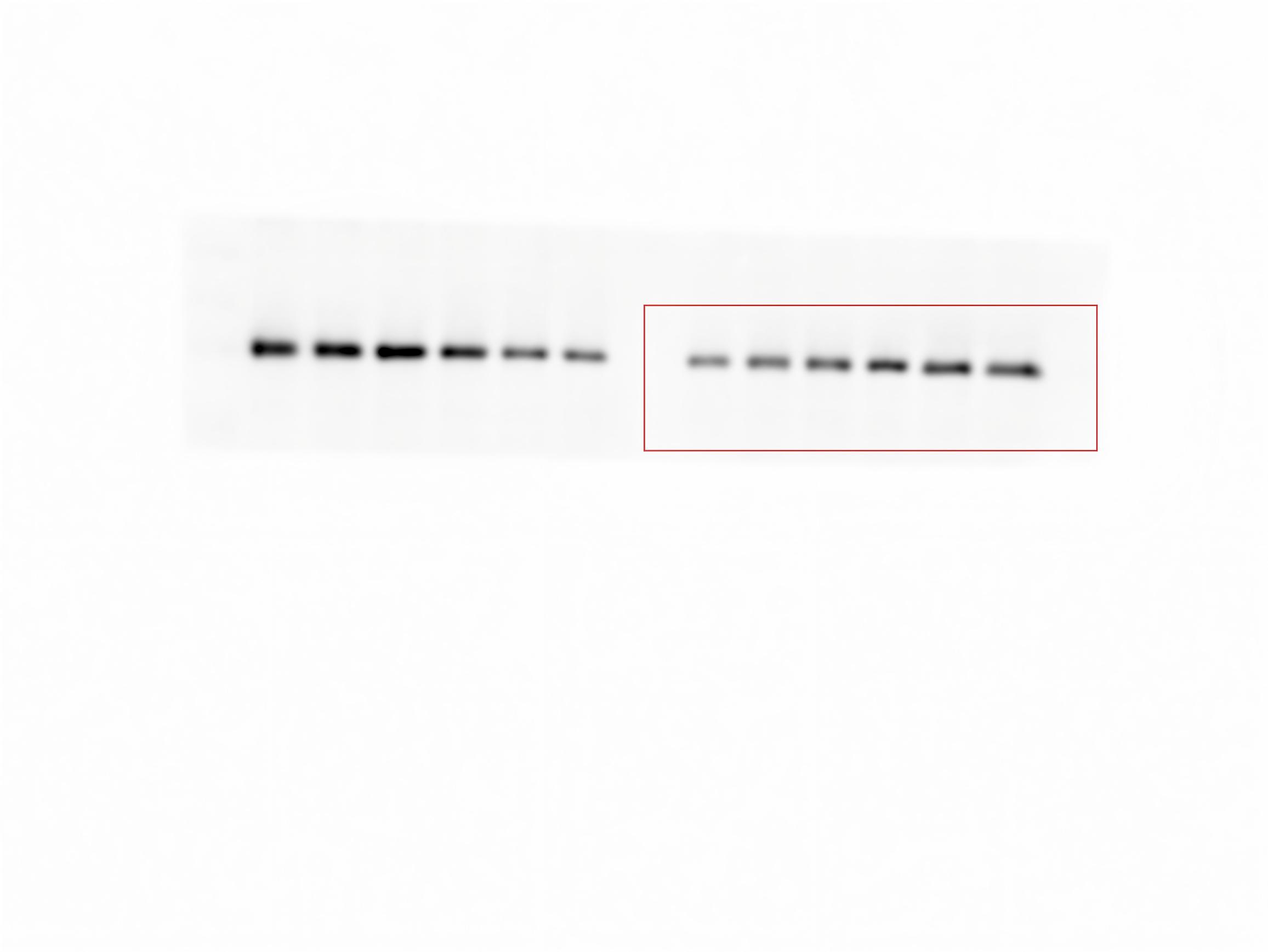

Supplement: Supplementary file 6 [file DataSheet4.zip › Supplementary Fig1F TRIM28.jpg]
